# The [3+2] Annulation of CF_3_-Ketimines by Re Catalysis: Access to CF_3_-Containing Amino Heterocycles and Polyamides

**DOI:** 10.1016/j.isci.2020.101705

**Published:** 2020-10-20

**Authors:** Saisai Zhang, Xun-Yong Liu, Zhenbang Chang, Xinxin Qiao, Heng-Ying Xiong, Guangwu Zhang

**Affiliations:** 1 Institute of Organic Functional Molecules, College of Chemistry and Chemical Engineering, Henan University, Kaifeng, 475004, P.R. China; 2 School of Chemistry and Materials Science, Ludong University, Yantai, 264025, P.R. China

**Keywords:** Chemistry, Molecular Inorganic Chemistry, Catalysis, Organic Synthesis

## Abstract

Transition metal catalyzed [3 + 2] annulation of imines with double bonds via directed C-H activation offers a direct access to amino cyclic motifs. However, owing to weak coordination and steric hindrance, trifluoromethylated ketimines have been an unaddressed challenge for TM-catalyzed annulations. Here, a rhenium-catalyzed [3 + 2] annulation of trifluoromethylated ketimines with isocyanates via C(sp^2^)-H activation has been disclosed. This approach provides an efficient platform for rapid access to a privileged library of CF_3_-containing iminoisoindolinones and polyamides by utilizing challenging CF_3_-ketimines as the annulation component. The capability of gram scale synthesis, the post-functionalization of the cyclization adduct, the derivation of complex natural molecules and the facile synthesis of polyamides highlight a diversity of synthetic potential of the current methodology.

## Highlights

• Challenging sp^2^ C-H activation
• Valuable CF_3_-containing heterocycles
• Variant synthetic applications
• Interesting fluorinated polyamides

## Graphical Abstract

**Figure:**
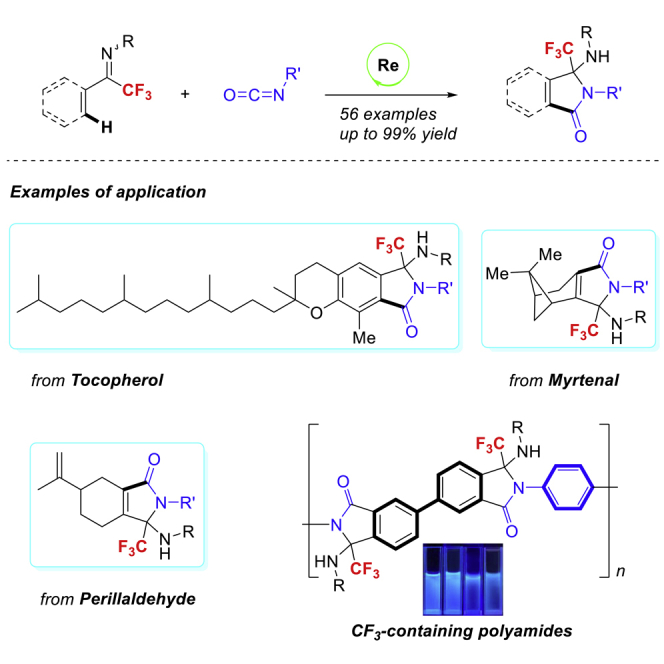

## Introduction

**Scheme 1:**
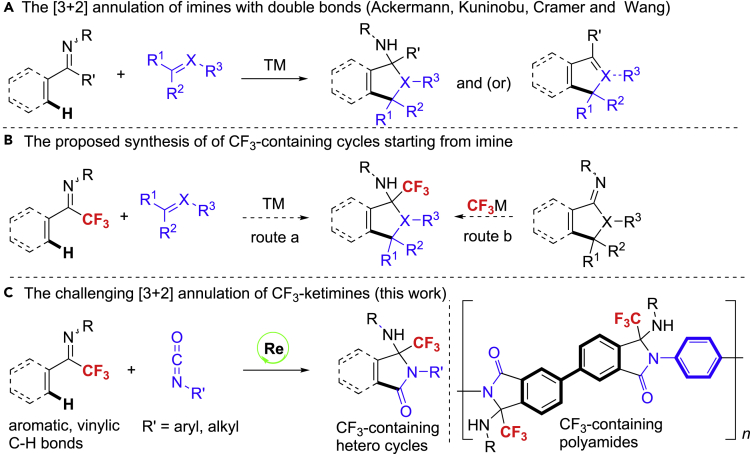
The Progresses of the [3 + 2] Annulation of Imines, the Strategies on the Synthesis of CF_3_-Containing Cycles from Imine and Current Work

**Figure 1:**
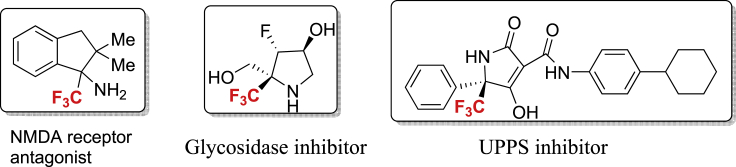
Selected Biologically Active CF_3_-Containing Amino (Hetero) Cycles

Besides serving as versatile and important organic intermediates for the synthesis of molecules with amine functionality (Layer, 1963; Bloch, 1998; Kobayashi and Ishitani, 1999; Martin, 2009; Blicke, 2011), imines are also eminent for the directing role in transition metal-catalyzed C-H functionalizations (Zhang et al., 2014a, 2014b; Chen et al., 2015; Sambiagio et al., 2018; Li and Shi, 2012; Rouquet and Chatani, 2013; Jiao et al., 2016; Gensch et al., 2016; Leitch and Frost, 2017; He et al., 2017; Yang et al., 2017; Hummel et al., 2017; Park et al., 2017; Dong et al., 2017; Gandeepan, et al., 2019). In particular, the directed ortho C(sp^2^)-H bond transformation of imines with an unsaturated bonds (Zhang et al., 2014a, 2014b; Yang and Huang, 2015) and the following cyclization process, a formal [3 + 2] annulation, which was first reported by Kuninobu (Kuninobu et al., 2005, 2006), has become a powerful route for the construction of amino carbon (hetero) cycles (Scheme 1A) (Kuninobu et al., 2010; Tran and Cramer, 2010, 2011; Zhang et al., 2013; Liu et al., 2015; Liang et al., 2017). However, trifluoromethylated ketimines remain a major challenge and have not been engaged in such an appealing procedure, which would give rise to biologically interesting and highly valuable CF_3_-containing amino carbon or hetero cyclic motifs with a quaternary carbon center (Figure 1) (Sani et al., 2007; Petrov, 2009; Satoshi et al., 2008; Hurley et al., 2009). The probable challenges could be ascribed to two aspects: (1) the weakened coordination potential of nitrogen atom by the strong electron withdrawing effect of CF_3_ group; (2) the increased steric hindrance by CF_3_ group compared with a common alkyl group (such as Me and Et) (Meanwell, 2018). Considering the fact that the electron-withdrawing character of CF_3_ group might enhance the reactivity of ketimines (Wang et al., 2006), the nucleophilic cyclization step would be likely assisted by CF_3_ group. To this end, we envisaged that, if the insertion of unsaturated bond into the C-H bond of CF_3_-ketimines could be accomplished by certain transition metal catalysis, it would also allow for the subsequent nucleophilic cyclization (Scheme 1B: route a). The proposed challenging [3 + 2] strategy would be more straightforward compared with the post-functionalization of a cyclic imine precursor with CF_3_-nucleophiles to deliver CF_3_-containing amino cycles (Scheme 1B: route b), and the latter approach may suffer from tedious synthetic steps of cyclic imine precursor and site selectivity issue during nucleophilic trifluoromethylation if other unsaturated bond presented in the imine structure.

Since the pioneering works of several groups (Chen and Hartwig, 1999; Kuninobu et al., 2005, 2006), rhenium-catalyzed C-H functionalization has become a promising implement for the rapid construction of new C-C and C-X bonds in an atom economic and environmentally benign manner (Kuninobu et al., 2008; Fukumoto et al., 2012; Sueki et al., 2013; Wang et al., 2013; Tang et al., 2013; Jin et al., 2013). Among these reactions, rhenium often exhibited exclusive catalytic activities over other transition metals, owing to the special properties of rhenium (Kuninobu and Takai, 2011). However, merging rhenium-catalyzed C-H activation with the synthesis of fluorinated molecules has been relatively underexploited. Herein, we describe an effective rhenium-catalyzed insertion of isocyanate into the C-H bond of CF_3_-ketimine/nucleophilic cyclization sequence for the rapid access CF_3_-substituted iminoisoindolinones (Scheme 1C). In this reaction, the imino group has been preserved in the absence of leaving group.

## Results and Discussion

### Reaction Optimization

We commenced our studies by using 2,2,2-trifluoro-N,1-diphenylethan-1-imine (**1a**) as the mode substrate to react with p-tolyl isocyanate (**2a**). After systematic optimization of the various reaction parameters, the expected 3-amino-3-(trifluoromethyl)isoindolin-1-one was obtained in 82% yield (Table 1, entry 1). In a control reaction that Re_2_(CO)_10_ was omitted, no desired product was detected (entry 2). Re salts other than Re_2_(CO)_10_ also mediated the formation of the product albeit in slightly lower yields (entries 3–4). A range of other metal carbonyls showed no catalytic activities on this annulation (entries 5–11). In addition, the use of common Pd, Cu, or Rh catalysts, which were successfully implemented in C-H functionalization of imines, resulted in no conversion of the staring material (entries 12–15). The nature of solvent also plays a key role in reaction efficiency. After the replacement of o-xylene with PhCl or toluene, the comparable results were achieved (entries 16–17). The use of polar solvents gave low conversions (entries 18–20), whereas very polar solvents such as DMSO totally impeded the formation of the desired product (entry 21). At last, no reaction occurred at lower temperature (entry 22).

**Table 1:** Influence of Reaction Parameters on [3 + 2] Annulation


Entry	Change from the Standard Conditions	Yield (%)^a^
1	None	82
2	In the absence of Re_2_(CO)_10_	_
3	ReBr(CO)_5_ instead of Re_2_(CO)_10_	47
4	ReCl(CO)_5_ instead of Re_2_(CO)_10_	66
5	Ru_3_(CO)_10_ instead of Re_2_(CO)_10_	_
6	Cr(CO)_6_ instead of Re_2_(CO)_10_	_
7	Fe_2_(CO)_9_ instead of Re_2_(CO)_10_	_
8	Co_2_(CO)_8_ instead of Re_2_(CO)_10_	_
9	Mn_2_(CO)_10_ instead of Re_2_(CO)_10_	_
10	W(CO)_6_ instead of Re_2_(CO)_10_	_
11	Mo(CO)_6_ instead of Re_2_(CO)_10_	_
12	Pd(OAc)_2_ instead of Re_2_(CO)_10_	_
13	Cu(OAc)_2_ instead of Re_2_(CO)_10_	_
14	[Rh(OAc)_2_]_2_ instead of Re_2_(CO)_10_	_
15	Rh(PPh_3_)_3_Cl instead of Re_2_(CO)_10_	_
16	PhCl instead of o-xylene	75
17	Toluene instead of o-xylene	78
18	THF instead of o-xylene	18
19	EtOAc instead of o-xylene	20
20	DCE instead of o-xylene	16
21	DMSO instead of o-xylene	_
22	At 130°C	_

Reaction conditions: Ketimine **1a** (0.3 mmol, 1 equiv.), isocyanate **2a** (0.6 mmol, 2 equiv.), catalyst (0.03 mmol, 0.1 equiv.), in solvent (3 mL) under Ar at 150 °C for 60 h.

a Isolated yield.

### Substrate Scope regarding CF_3_-Ketimines

**Scheme 2:**
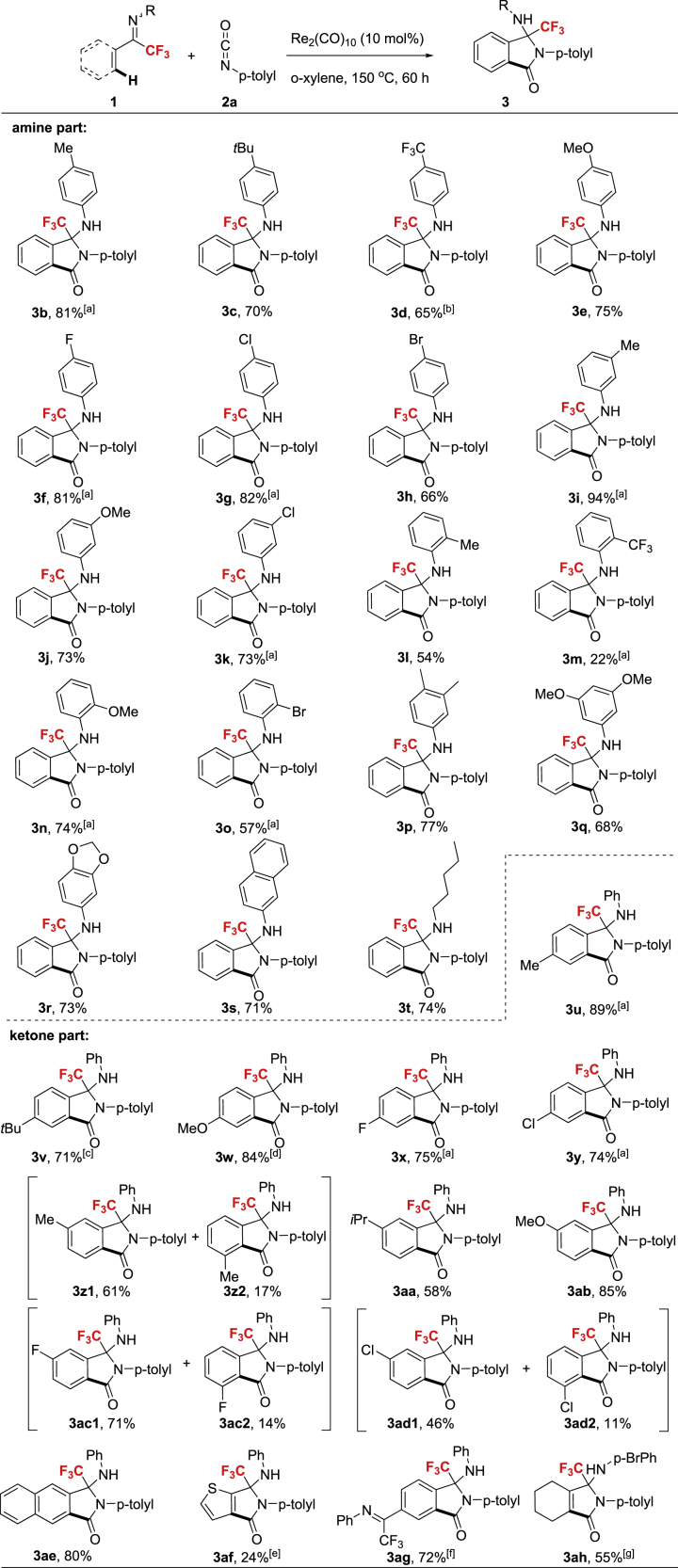
Scope of the Ketimines Reaction conditions: Ketimine **1** (0.3 mmol, 1 equiv.), isocyanate **2** (0.6 mmol, 2 equiv.), catalyst (0.03 mmol, 0.1 equiv.), in o-xylene (3 mL), under Ar at 150 °C for 60 h, isolated yield. ^a^in PhCl for 48 h. ^b^at 160 °C. ^c^at 140 °C. ^d^at 130 °C. ^e^at 110 °C. ^f^on 0.15 mmol scale, at 160 °C for 72 h. ^g^on 0.2 mmol scale.

The Re-catalyzed insertion/cyclization process can be applied to a series of CF_3_-ketimines with different substitution patterns on amines or ketones (Scheme 2). For the amine part, the reactions of imines with either electron-deficient or electron-rich groups on para, meta, or ortho position on anilines were conducted to furnish **3** in decent to excellent yields. In general, more electron-deficient aniline derived substrates gave slightly lower yields, and the ortho-substituted ones were converted in low to moderate yields probably owing to the steric hindrance during the cyclization step. A series of functional groups such as F, Cl, Br, methyl ethers, dioxolyl, and CF_3_ on anilines were compatible under the optimal conditions. It is noticed that 2-naphthylamine and amylamine were well tolerated to give corresponding cyclization products in good yields (**3s**, **3t**). In regard of substitutions on ketone part, there was no obvious electronic effect on reaction outcomes in which substrates with either electron-deficient or electron-rich substituents on aromatic ring all afforded desired products in moderate to good yields. In addition, owing to regioselectivity, a mixture of two regioisomers was obtained from the meta-substituted ketone (**3z**, **3ac**, **3ad**). Furthermore, the reaction of 2-thienyl ketone derived CF_3_-ketimine **1af** proceeded to afford the expected product **3af** in low yield, probably owing to the instability of the substrate under standard conditions. Diimine **1ag** gave the mono-annulation adduct in 72% yield, while delivering the di-annulation product in trace amount. Gratefully, vinylic CF_3_-ketimine **1ah** smoothly underwent the [3 + 2] sequence to give the desired cyclization product in moderate yield (**3ah**).

### Substrate Scope regarding Isocyanates

**Scheme 3:**
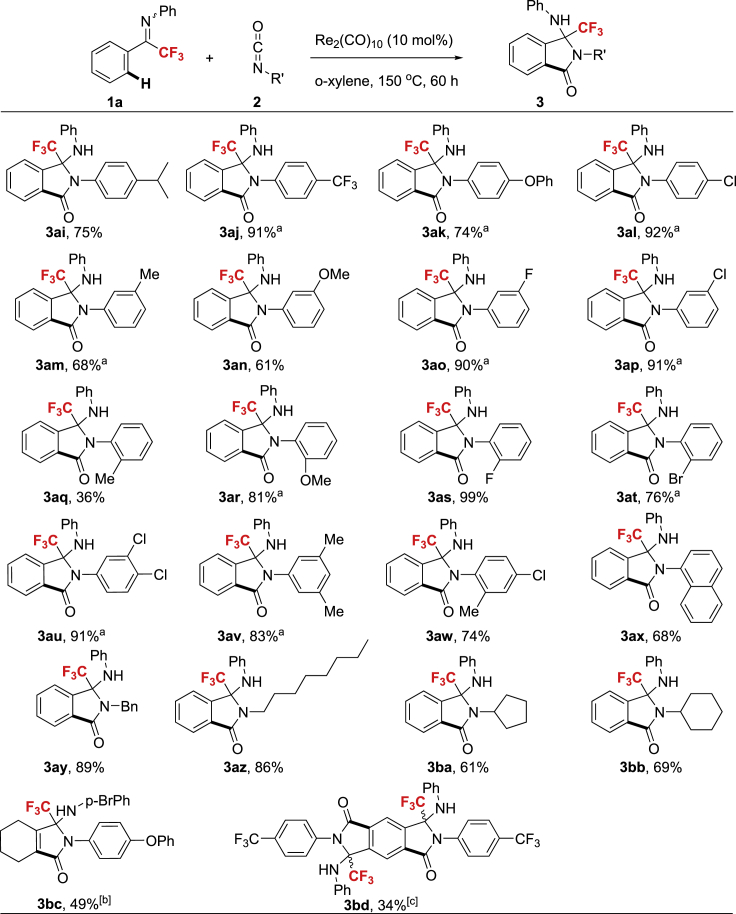
Scope of the Isocyanates Reaction conditions: Ketimine **1** (0.3 mmol, 1 equiv.), isocyanate **2** (0.6 mmol, 2 equiv.), catalyst (0.03 mmol, 0.1 equiv.), in o-xylene (3 mL), under Ar at 150 °C for 60 h, isolated yield. ^a^In PhCl for 48 h. ^b^On 0.2 mmol scale. ^c^On 0.15 mmol scale, at 160 °C for 72 h.

The reaction scope was then tested on the isocyanate side (Scheme 3). Aryl isocyanates with either an electron-donating or electron-withdrawing group smoothly underwent the cyclization reaction to give the desired product in moderate to excellent yield, implying no significant electronic effect on the aromatic ring of isocyanate. Interestingly, in the case of the ortho-CH_3_ substituted isocyanate, the reaction outcome was moderate (**3aq**). Functional groups such as F, Cl, Br, methyl ethers, phenyl ether, and CF_3_ were amenable to the optimal reaction conditions. In addition, naphthalenyl isocyanate was transformed to corresponding product in good yield (**3ax**). Furthermore, aliphatic isocyanates with linear or cyclic chains smoothly proceeded to generate the desired products in good to high yield (**3ay**-**bb**). The vinylic C-H bond of ketimine **1ah** could be further functionalized with substituted isocyanate (**2d**), affording the desired annulation adduct in moderate yield. Upon the treatment of diimine **1ag** with the highly electron-deficient isocyanate **2c**, the di-annulation adduct was isolated as the main product.

**Figure 2:**
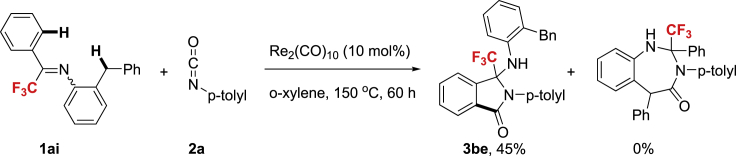
Intramolecular Competitive sp^2^ and sp^3^ C-H Bond Activation

With aromatic sp^2^ and benzylic sp^3^ C-H bonds installed in the same molecule, the sp^2^ C-H bond activation was preferred over sp^3^ C-H bond when **1ai** was subjected to standard reaction conditions, indicating the highly selective profile of the protocol (Figure 2).

### Mechanistic Investigations

**Scheme 4:**
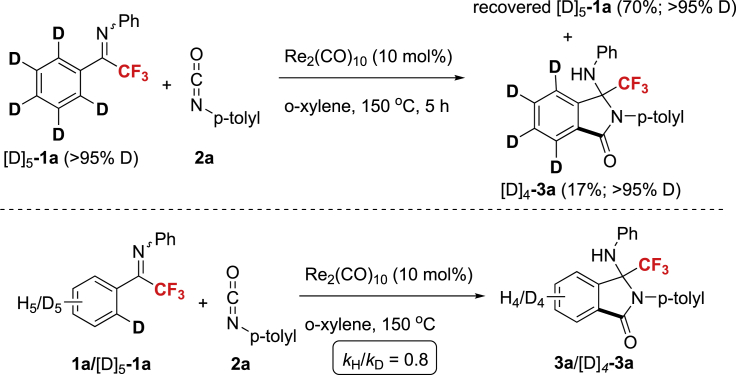
The D–H Exchange Experiment and the Kinetic Isotope Effect Experiments

To gain some mechanistic information on this reaction, the deuterium-labeling experiments were conducted (Scheme 4). With the deuterium-labeled compound [D]_5_-**1a**, D-H exchange was observed with neither the substrate nor the product (Scheme 4, top). Based on the initial rates of parallel reactions, the intermolecular kinetic isotope effect (KIE) of the rhenium-catalyzed C-H functionalization was measured to be k_H_/k_D_ = 0.8, demonstrating that the C–H bond cleavage of imine **1a** might not be the kinetically relevant step (Scheme 4, bottom).

**Scheme 5:**
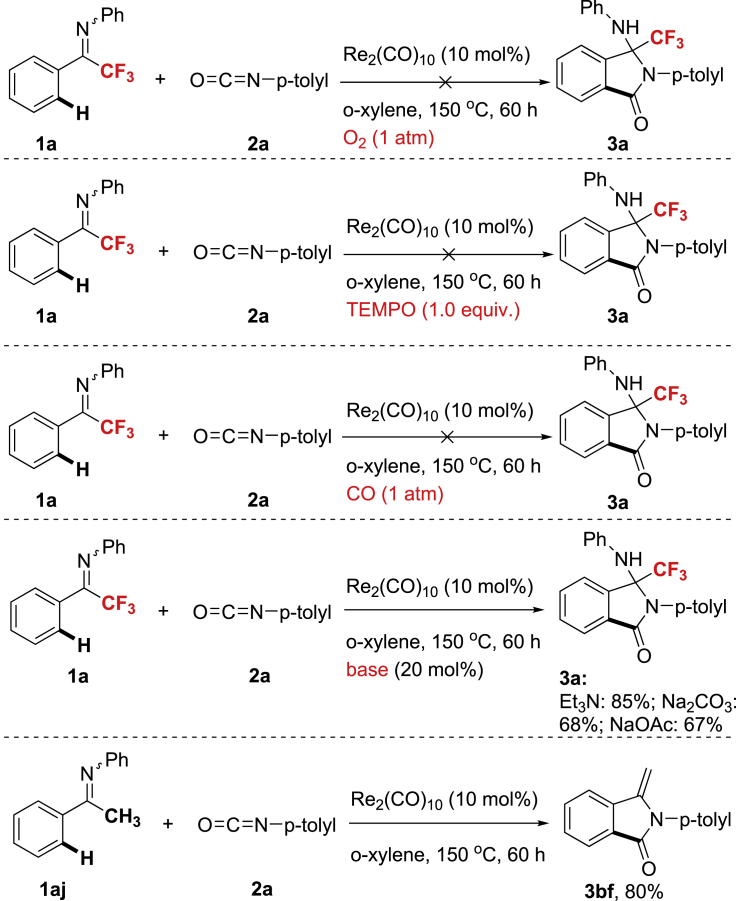
Control Experiments

Several control experiments were performed for further mechanistic studies (Scheme 5). Under O_2_, no desired annulations product was observed, suggesting low-valent Re catalyst is essential for the reaction since rhenium could be oxidized to a high oxidation state by O_2_ (Ivin and Mol, 1997). In the presence of radical scavenger TEMPO or under CO, the annulation was impeded, indicating CO dissociation and Re(CO)_5_ radicals formation likely involved in annulations process (Reynolds et al., 2001). In the presence of catalytic amount of base, the reaction efficiency varied. It was observed that Et_3_N gave slightly higher yield, whereas inorganic bases such as Na_2_CO_3_ and NaOAc exhibited reduced performance on reaction outcomes. Interestingly, after replacement of CF_3_ with CH_3_ on imine structure, the deaminative annulation product **3bf** was generated in good yield (80%) under standard reaction conditions (Hou et al., 2013), indicating the role of CF_3_ group for stabilizing the resulting amino heterocycles.

**Scheme 6:**
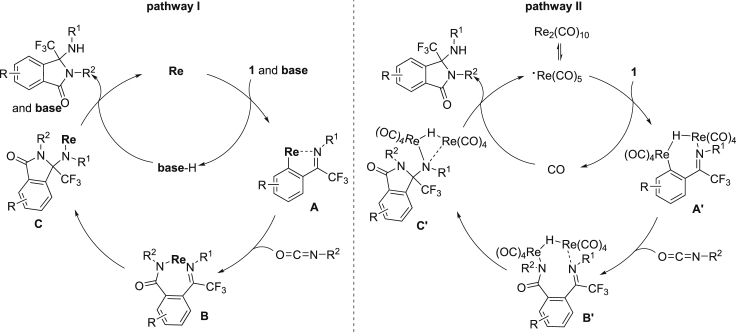
Plausible Reaction Mechanism

On the basis of these mechanistic investigations and previous reports (Chen and Hartwig, 1999; Kuninobu et al., 2005, 2006, 2008; Fukumoto et al., 2012; Sueki et al., 2013; Wang et al., 2013; Tang et al., 2013; Jin et al., 2013; Reynolds et al., 2001; Lapointe and Fagnou, 2010; Ackermann, 2011; Engle et al., 2012), we then presumed two catalytic pathways for the annulation of CF_3_-ketimines with isocyanates (Scheme 6). In pathway **I**, with the assistance of base, the initial C-H metalation of CF_3_-ketimine **1** formed rhenacycle **A** after the coordination of imine with rhenium catalyst, which was followed by the insertion of isocyanate to generate the aminated rhenium species **B**. The further intramolecular nucleophilic amination and proto-demetalation of the aminated rhenium species **C** furnished the desired cyclization product and regenerated the active rhenium catalyst. In pathway **II**, the homolytic Re-Re bond cleavage of Re_2_(CO)_10_ produced Re(CO)_5_ radicals, which reacted with CF_3_-ketimine **1** to form dinuclear Re complexes **A′** through CO dissociation and C-H bond cleavage. The followed insertion of isocyanate generated the aminated dinuclear rhenium species **B′**, which further underwent intramolecular nucleophilic amination to give species **C’**. The reductive elimination of **C′** furnished the desired cyclization product and regenerated the active rhenium catalyst.

### Synthetic Applications

**Scheme 7:**
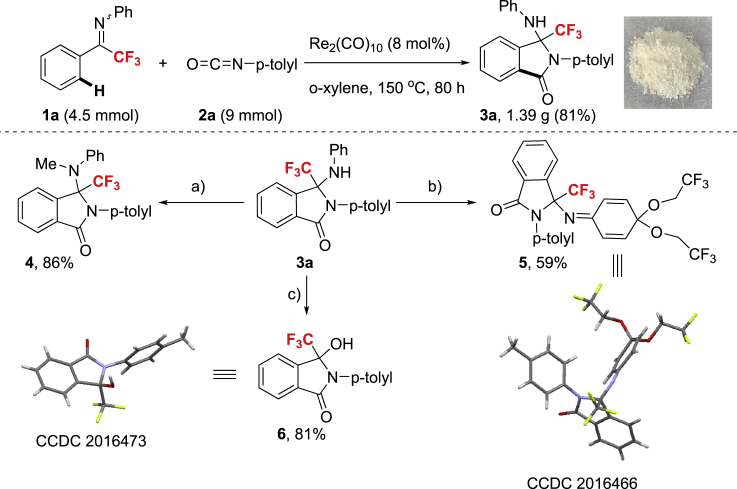
Synthetic Applications Reaction conditions: (a) LiHMDS (8.0 equiv.), MeI (8.0 equiv.), in THF under Ar, refluxing for 24 h; (b) PIDA (4.0 equiv.), Cs_2_CO_3_ (1.5 equiv.), in TFE, at 70 °C for 6 h; (c) BF_3_⋅Et_2_O (3.0 equiv.), in CH_3_CN, under Ar, at 80 °C for 24 h.

Next, the synthetic utility of this approach was explored (Scheme 7). The annulation of **1a** and **2a** was conducted on 4.5 mmol scale with reduced catalyst loading (8 mol%) and prolonged reaction time, furnishing **3a** in 81% yield (1.39 g), which is comparable with the aforementioned result on small scale. The methylation of **3a** with MeI as the alkylation reagent smoothly afforded **4** in 86% yield. Surprisingly, the treatment of **3a** with PIDA as the oxidant in TFE delivered trifluoroethyl ketal moiety **5** in 59% yield, arising from the oxidation of the aniline part of **3a**. Upon the treatment of **3a** with stoichiometric amount of strong Lewis acid BF_3_·Et_2_O, the unexpected C-N bond cleavage was observed to give CF_3_-tertiary alcohol and the OH group likely came from trace water. Notably, the structures of **5** and **6** were further unambiguously confirmed by X-ray crystallography and the latter two transformations constitute novel discoveries that relevant processes have been scarcely reported in the literature.

**Scheme 8:**
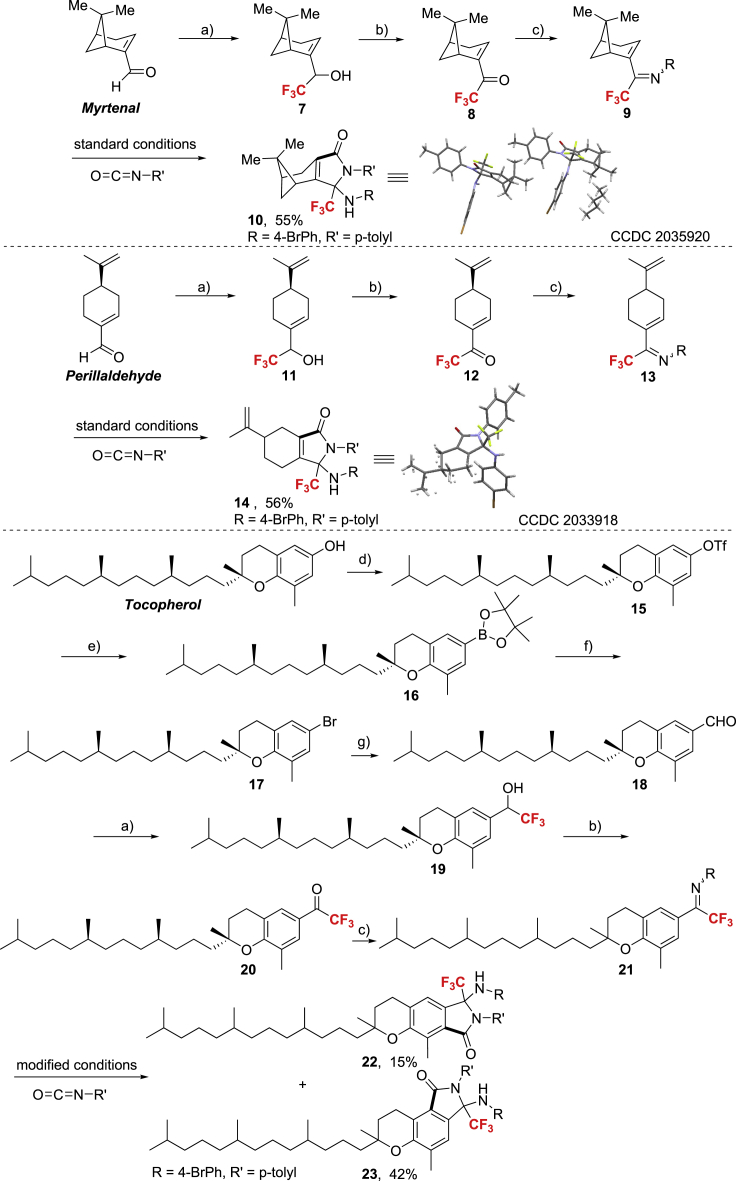
Derivation of Complex Molecules Reaction conditions: (a) TMSCF_3_ (2.2 equiv.), TBAF (0.5 equiv.), in THF under Ar, -40 °C to rt; (b) DMP (1.2 equiv.), in DCM, rt for 30 min; (c) 4-Br-aniline (2.0 equiv.), TsOH⋅H_2_O (0.2 equiv.), in toluene, at 140 °C for 48 h; (d) Tf_2_O (1.3 equiv.), Et_3_N (2.5 equiv.), in DCM, at 0 °C for 30 min; (e) B_2_pin_2_ (2.0 equiv.), PdCl_2_(dppf) (10 mol%), Et_3_N (3.0 equiv.), in dioxane, at 100 °C for 4 h; (f) CuBr_2_ (3.0 equiv.), in MeOH, at 90 °C for 72 h; (g) n-BuLi (2.0 equiv.), DMF (5.0 equiv.) in THF, at -78°C.

The applicability of this Re-catalyzed annulations was further examined by selected derivation of several natural products (Scheme 8). For example, by nucleophilic trifluoromethylation of *Myrtenal* or *Perillaldehyde* with TMSCF_3_/TBAF system, the subsequent oxidation with DMP, and the followed condensation with aniline, the desired *Myrtenal* or *Perillaldehyde* derived CF_3_-ketimine **9** or **13** was formed in decent yield, which was converted to the corresponding CF_3_-containing amino hetero cycles in good yield under standard conditions. It was established that the strained four-member ring in **9** was intact during the annulations process and the structures of **10** and **14** were further unambiguously confirmed by X-ray crystallography. Following the similar procedure after conversion of *Tocopherol* to its aldehyde moiety through a subsequent four-step procedure, the expected CF_3_-ketimine **21** was generated in decent yield, which was subjected to slightly modified annulations conditions to furnish two regioisomers **22** and **23** as the cyclization adduct.

**Figure 3:**
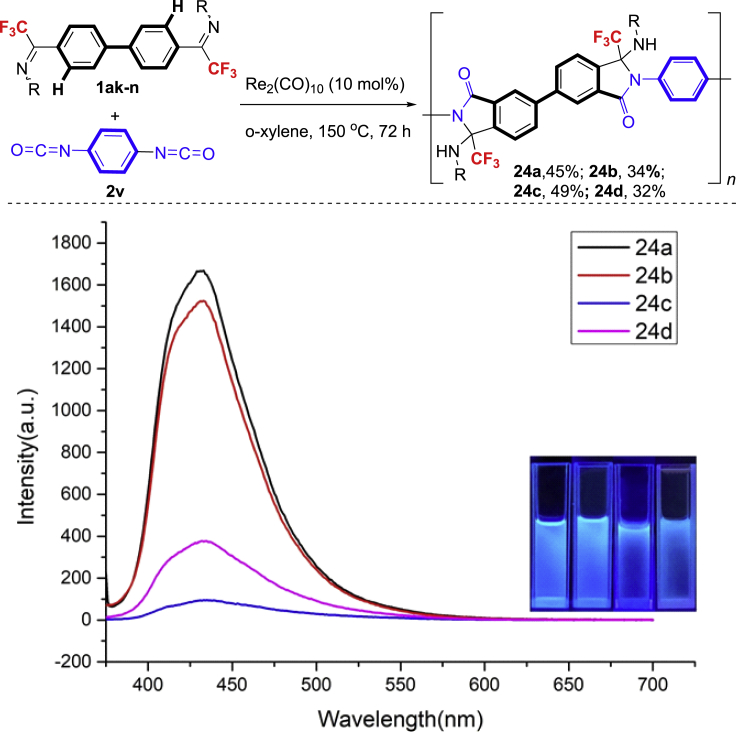
The Synthesis of CF_3_-Containing Polyamides and Optical Fluorescence Spectra of 24a-d in DCM Solution (1 mg/mL) (Insets Show the Respective Photographs under UV Illumination)

Inspired by the success of double annulation reactions of the CF_3_-diimine (**1ag**) and Kuninobu's leading work in the synthesis of polyimides (Sueki et al., 2013), we then attempted to explore the possibility for the synthesis of important trifluoromethylated polyamides bearing potential unique properties such as enhanced stability, solubility, and low surface energy (Wang, et al., 2004; Tsuchiya et al., 2006) through Re-catalyzed [3 + 2] annulations via C-H activation (Suraru et al., 2016; Yang et al., 2018; Blaskovits and Leclerc, 2019). Finally, trifluoromethylateddiimines with a diphenyl backbone were proved to be suitable annulation partners with phenyl diisocyanate, affording the trifluoromethylated polyamides in moderate yields with Mw/Mn from 1.6 to 1.8 and good solubility in organic solvents such as dichloromethane, chloroform, and THF (Figure 3, top). The preliminary study of optical properties of these polymers was performed. The UV spectra of **24a**−**d** display maximum absorption bands at 255–265 nm in CH_2_Cl_2_, which is ascribed to the π−π∗ transition of arenes (see Supplementary Information). The **24a**-**d** solutions show strong blue emissions with the emission peaks around 438 nm (Figure 3, bottom), which make them suitable as host materials for blue organic light-emitting devices.

### Conclusion

In conclusion, we have presented an unprecedented [3 + 2] annulation of CF_3_-ketimines with isocyanates via rhenium-catalyzed C-H activation. This approach demonstrated good functional group tolerance and broad substrate scope both on ketimines and isocyanates. A series of novel CF_3_-containing iminoisoindolinones were constructed in decent to excellent yield. This is the first example on functionalization of unactivated sp^2^ C-H bonds of CF_3_-ketimines, leading to the simultaneous formation of new C-C and C-N bonds by one simple operation. The imino group being intact during the annulations process in the absence of leaving group highlights the ability for trifluoromethylated amine synthesis of the catalytic protocol. The preliminary mechanistic studies indicated that Re(CO)_5_ radicals and dinuclear rhenium species were likely involved in the annulation process. Furthermore, the capability for gram scale synthesis, the diverse transformations of the annulation adduct, the derivation of the natural products and the ability for the construction of polyamides show the cases for synthetic applications of current strategy. Further employment of CF_3_-ketimines as the annulations partner with other unsaturated bonds and the systematic mechanistic study are ongoing in our laboratory.

### Limitations of the Study

The catalyst loading was a little high compared with previously reported Re systems, and lower catalyst loading (<10 mol%) was detrimental for reaction efficiency.

### Resource Availability

#### Lead Contact

Further information and requests for resources and reagents should be directed to and will be fulfilled by the Lead Contact, Guangwu Zhang (gw.zhangchem@hotmail.com).

#### Materials Availability

All unique/stable reagents generated in this study are available from the Lead Contact without restriction.

#### Data and Code Availability

The structure of 3-((4,4-bis(2,2,2-trifluoroethoxy)cyclohexa-2,5-dien-1-ylidene)amino)-2-(p-tolyl)-3-(trifluoromethyl)isoindolin-1-one (**5**, CCDC, 2016466), 3-hydroxy-2-(p-tolyl)-3-(trifluoromethyl)isoindolin-1-one (**6**, CCDC, 2016473), 3-((4-bromophenyl)amino)-5,5-dimethyl-2-(p-tolyl)-3-(trifluoromethyl)-2,3,4,5,6,7-hexahydro-1H-4,6-methanoisoindol-1-one (**10**, CCDC, 2035920), 3-((4-bromophenyl)amino)-6-(prop-1-en-2-yl)-2-(p-tolyl)-3-(trifluoromethyl)-2,3,4,5,6,7-hexahydro-1H-isoindol-1-one (**14**, CCDC, 2033918) in this article have been deposited in the Cambridge Crystallographic Data Center.

## Methods

All methods can be found in the accompanying Transparent Methods supplemental file.

## Supplemental Information

Document S1. Transparent Methods, Figures S1–S346, Schemes S1–S19, and Table S1

## Footnotes

Supplemental Information can be found online at https://doi.org/10.1016/j.isci.2020.101705.
