# Supplementary material for: The [3+2] Annulation of CF3-Ketimines by Re Catalysis: Access to CF3-Containing Amino Heterocycles and Polyamides
Source: iScience. 2020 Oct 20;23(11):101705. doi: 10.1016/j.isci.2020.101705 (PMC7644752; doi:10.1016/j.isci.2020.101705)
Supplement: Document S1. Transparent Methods, Figures S1–S346, Schemes S1–S19, and Table S1 [file mmc1.pdf]

iScience, Volume *23*

Supplemental Information

The [3+2] Annulation of CF_3-Ketimines by Re Catalysis: Access to CF_3-Containing Amino Heterocycles and Polyamides

Saisai Zhang, Xun-Yong Liu, Zhenbang Chang, Xinxin Qiao, Heng-Ying Xiong, and Guangwu Zhang

Supporting Information

The [3+2] Annulation of CF_3-Ketimines by Re Catalysis: Access to CF_3-Containing Amino Heterocycles and Polyamides

Saisai Zhang, Xun-Yong Liu, Zhenbang Chang, Xinxin Qiao, Heng-Ying Xiong and Guangwu Zhang

Supplementary Figures

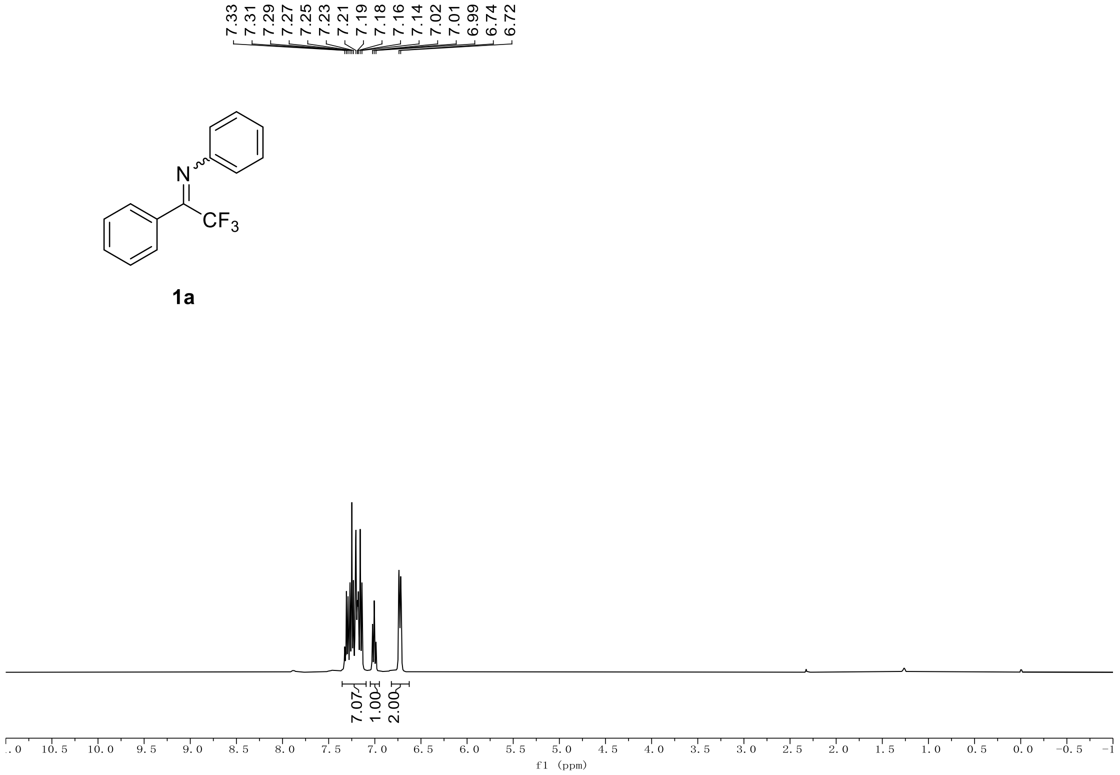

Figure S1. ^{1}H NMR (400 MHz, $CDCl_3$) spectrum of compound 1a, related to Table 1

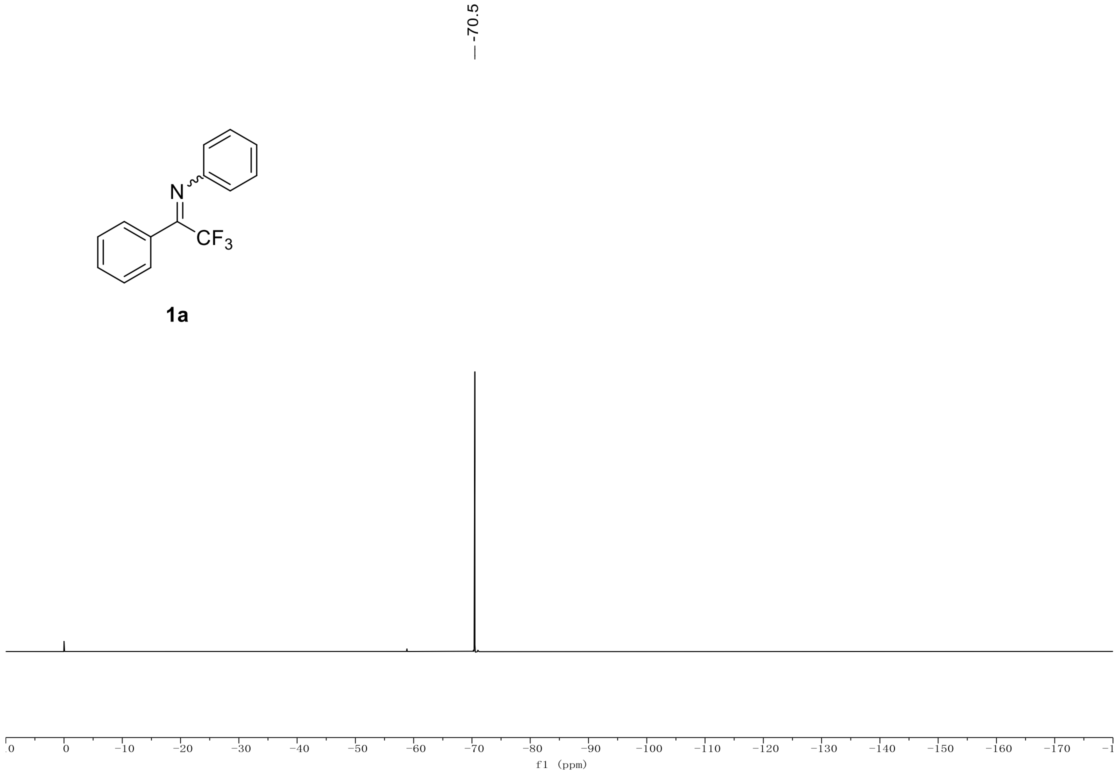

Figure S2. ^{19}F NMR (376 MHz, $CDCl_3$) spectrum of compound 1a, related to Table 1

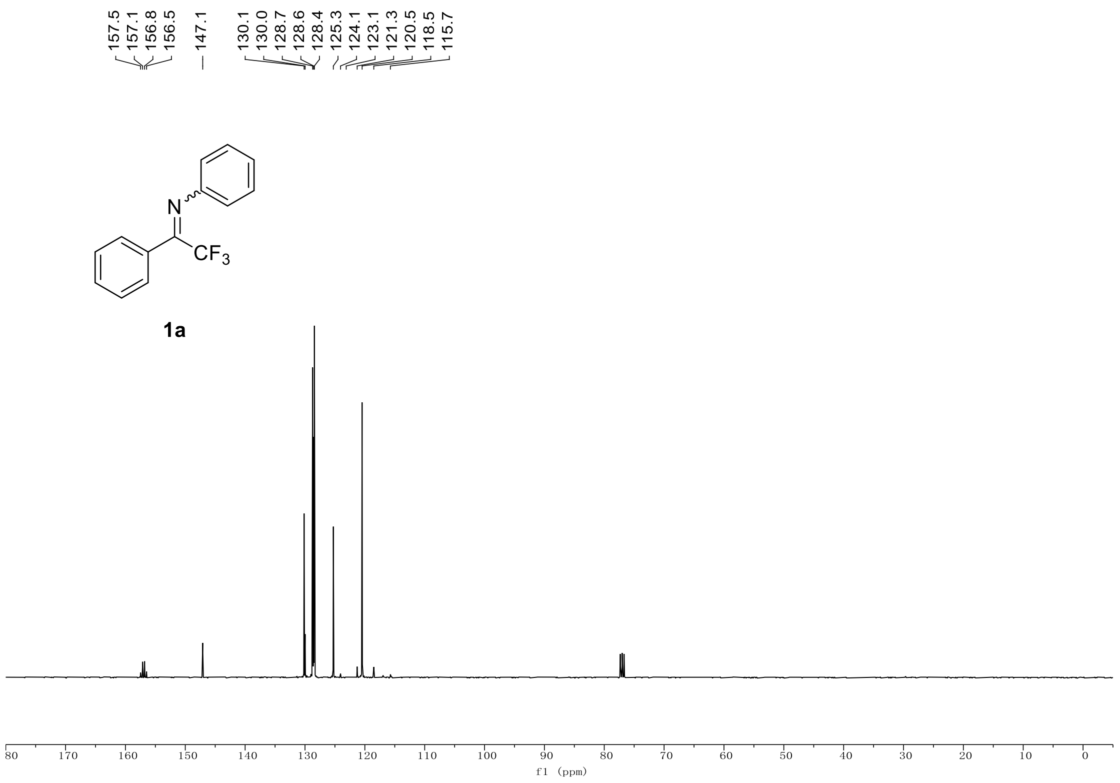

Figure S3. ^{13}C NMR (101 MHz, $CDCl_3$) spectrum of compound 1a, related to Table 1

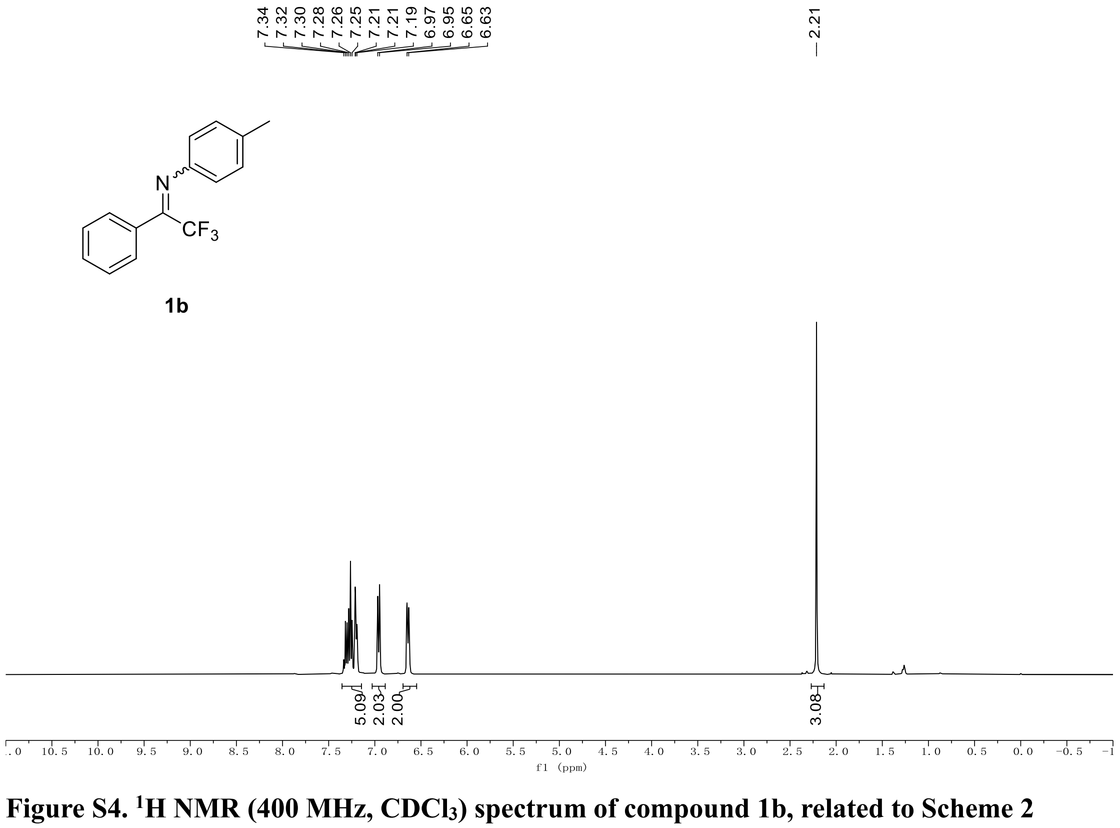

Figure S4. ^{1}H NMR (400 MHz, $CDCl_3$) spectrum of compound 1b, related to Scheme 2

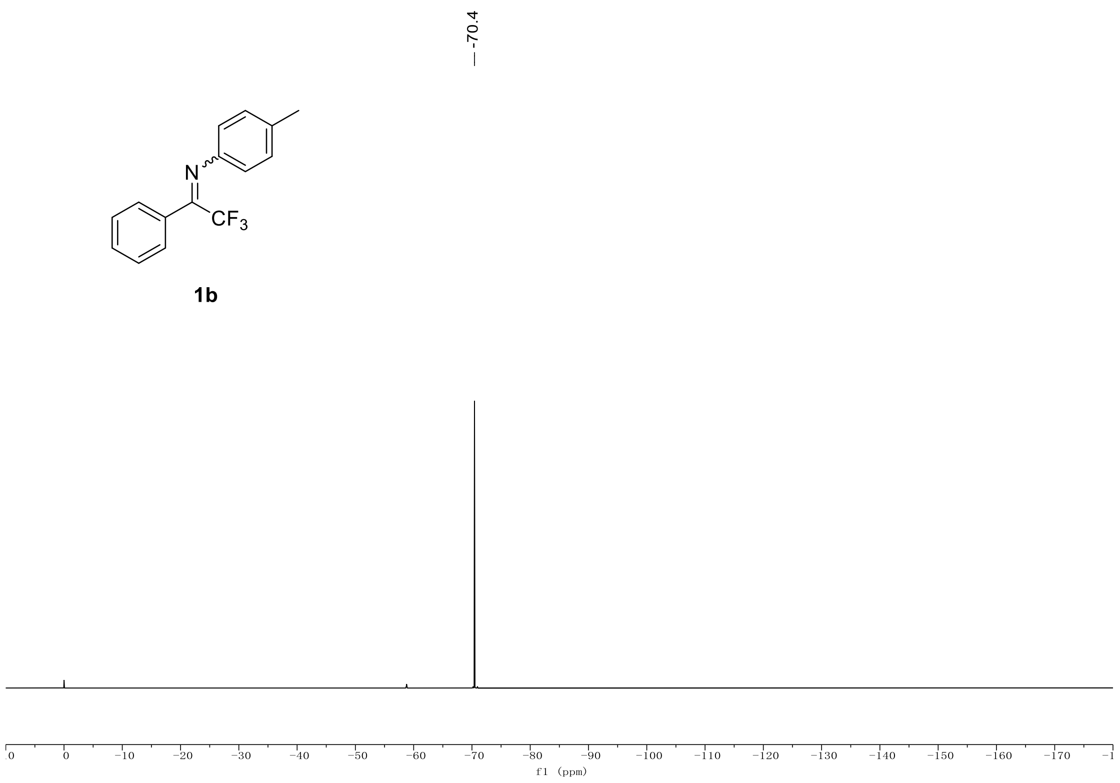

Figure S5. ^{19}F NMR (376 MHz, $CDCl_3$) spectrum of compound 1b, related to Scheme 2

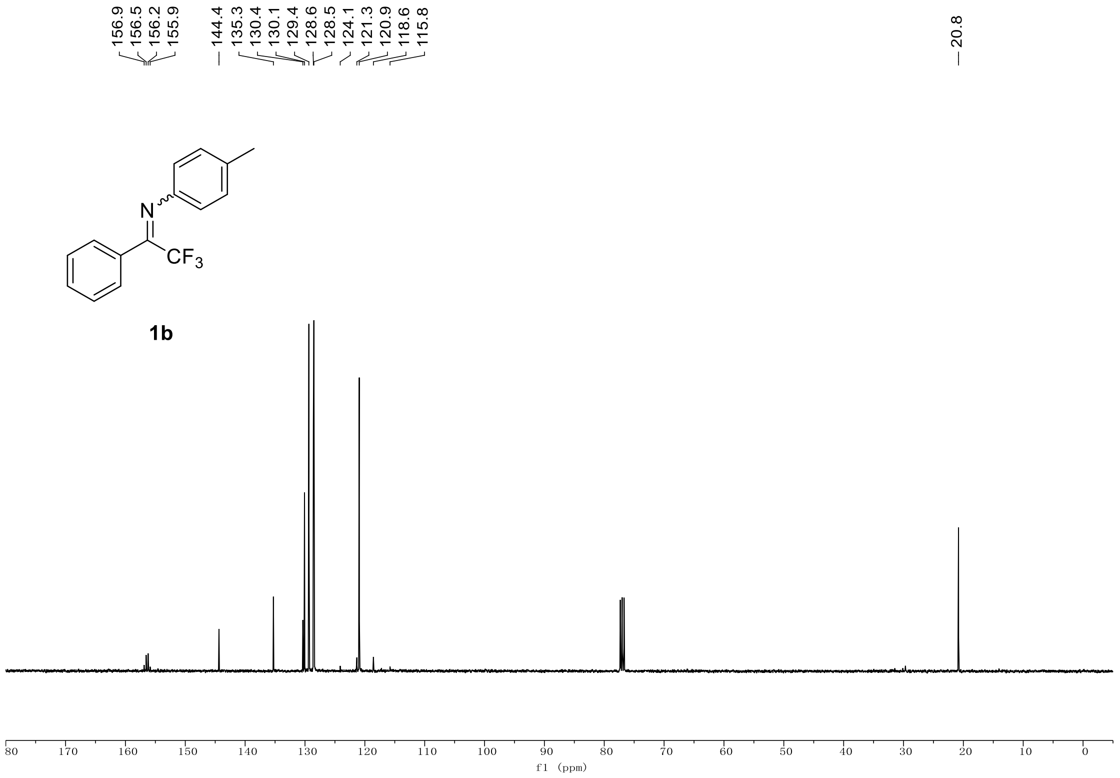

Figure S6. ^{13}C NMR (101 MHz, $CDCl_3$) spectrum of compound 1b, related to Scheme 2

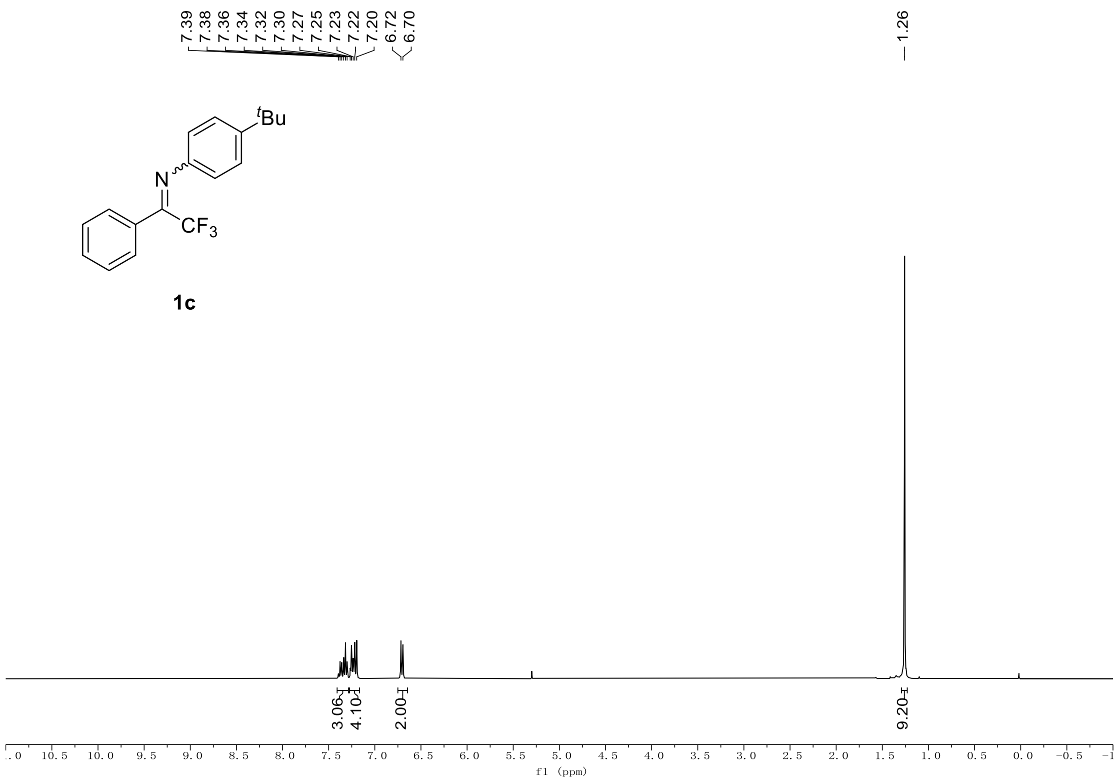

Figure S7. ^{1}H NMR (400 MHz, $CDCl_3$) spectrum of compound 1c, related to Scheme 2

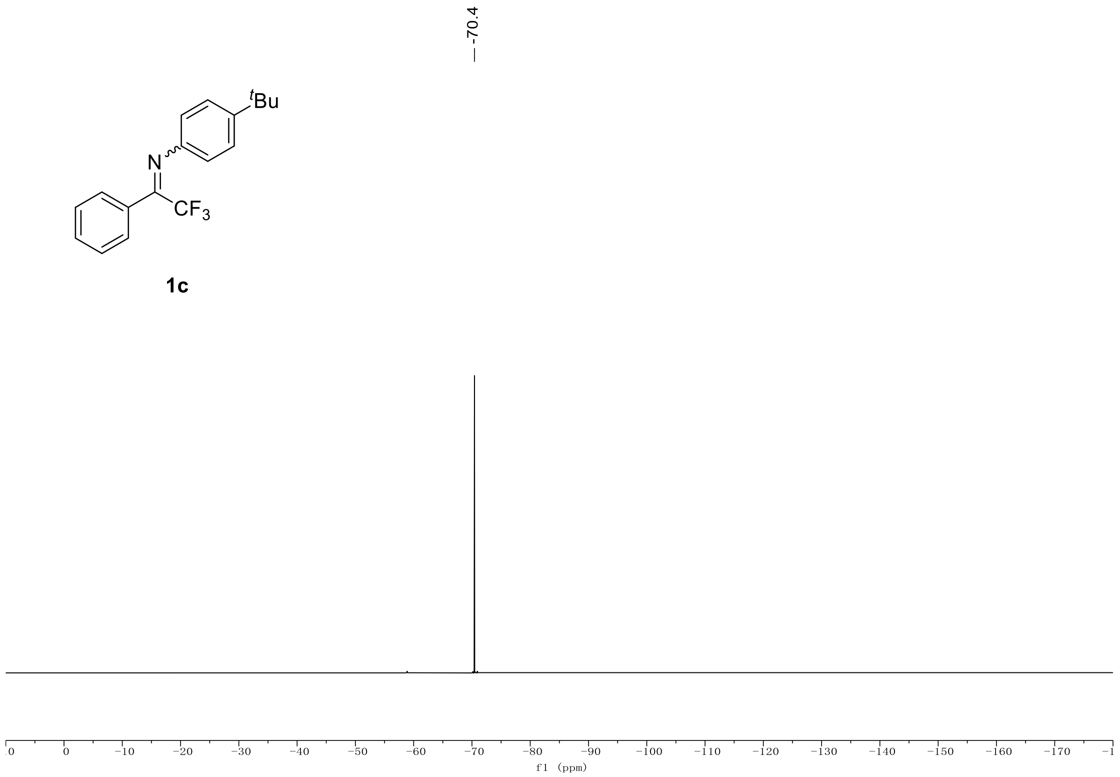

Figure S8. ^{19}F NMR (376 MHz, $CDCl_3$) spectrum of compound 1c, related to Scheme 2

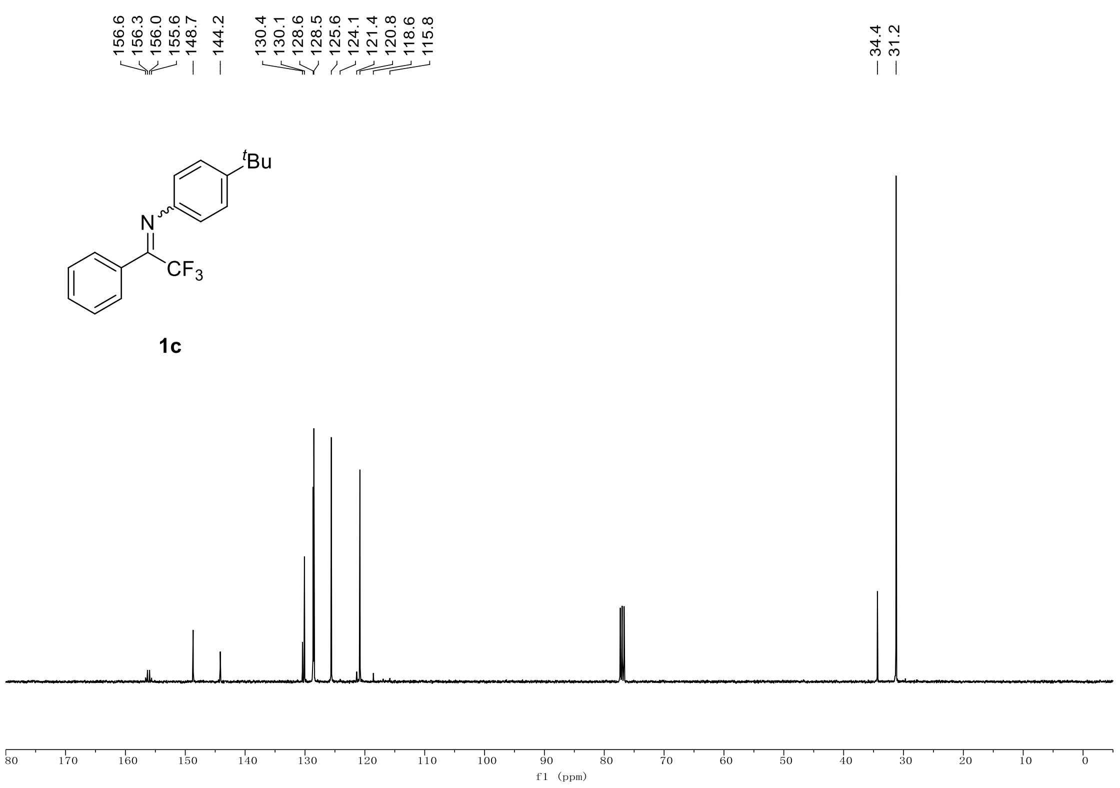

Figure S9. ^{13}C **NMR (101 MHz,** $CDCl_3$**) spectrum of compound 1c, related to Scheme 2**

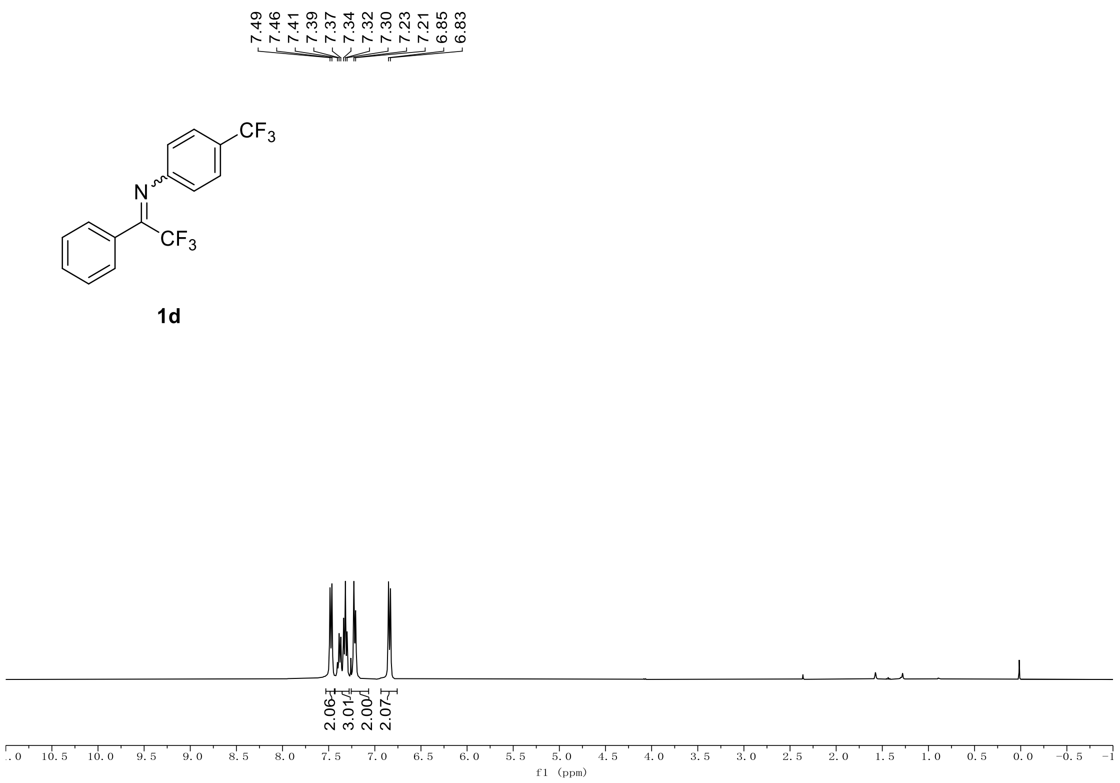

Figure S10. ^{1}H **NMR (400 MHz,** $CDCl_3$**) spectrum of compound 1d, related to Scheme 2**

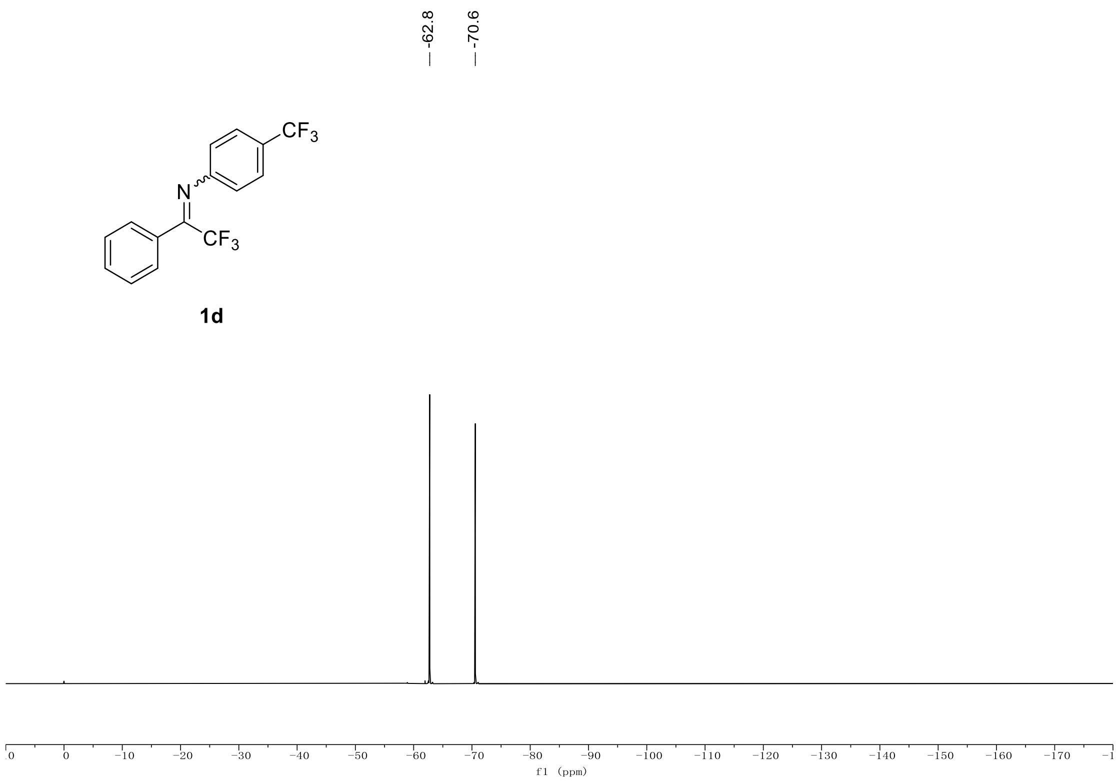

Figure S11. ^{19}F NMR (376 MHz, $CDCl_3$) spectrum of compound 1d, related to Scheme 2

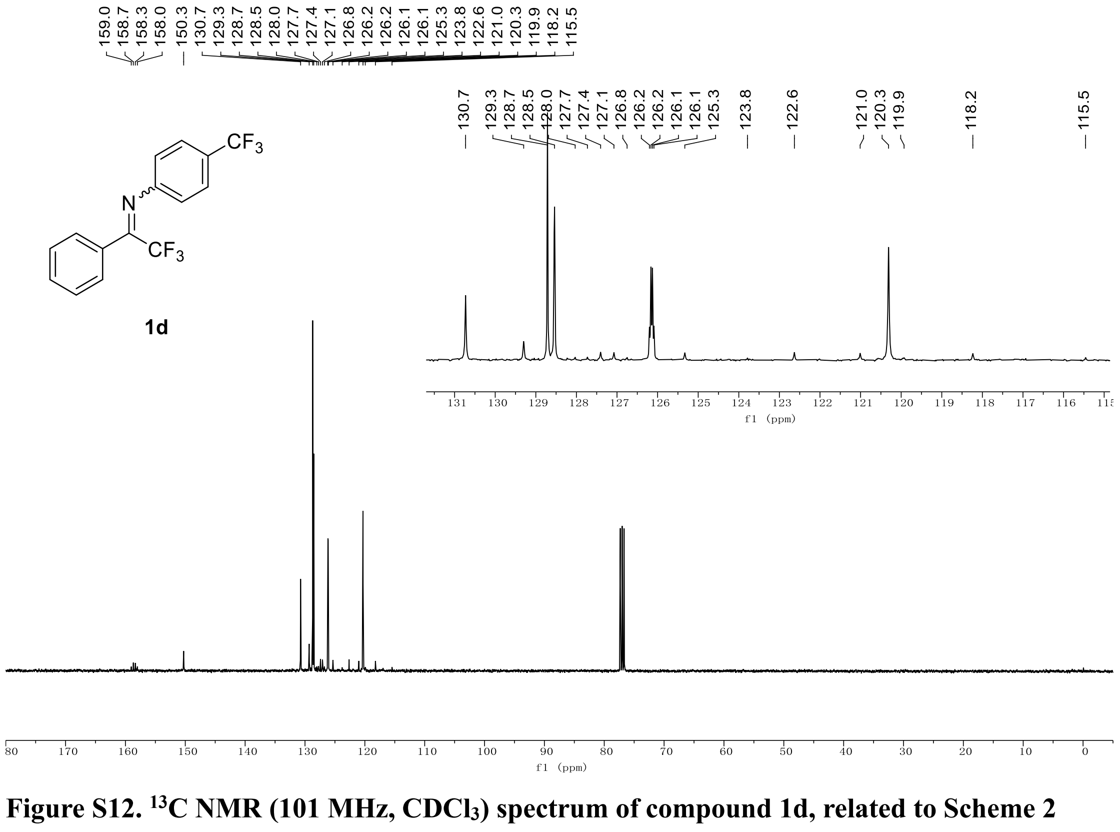

Figure S12. ^{13}C NMR (101 MHz, $CDCl_3$) spectrum of compound 1d, related to Scheme 2

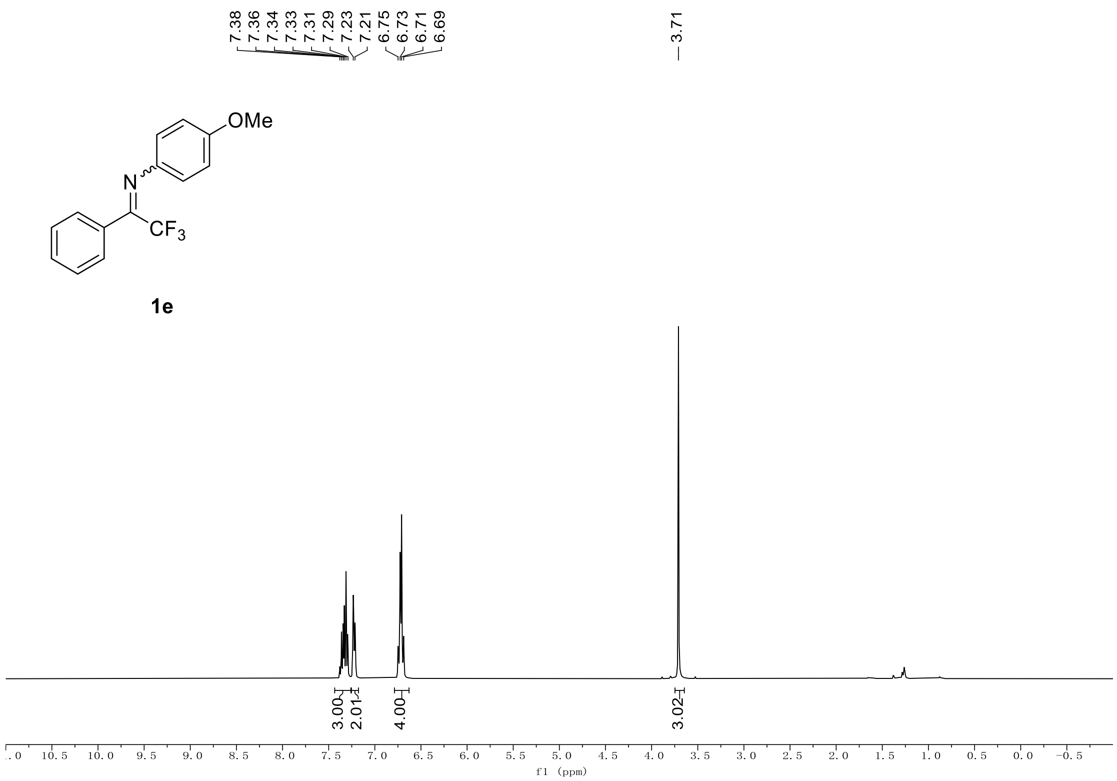

Figure S13. ^{1}H NMR (400 MHz, $CDCl_3$) spectrum of compound 1e, related to Scheme 2

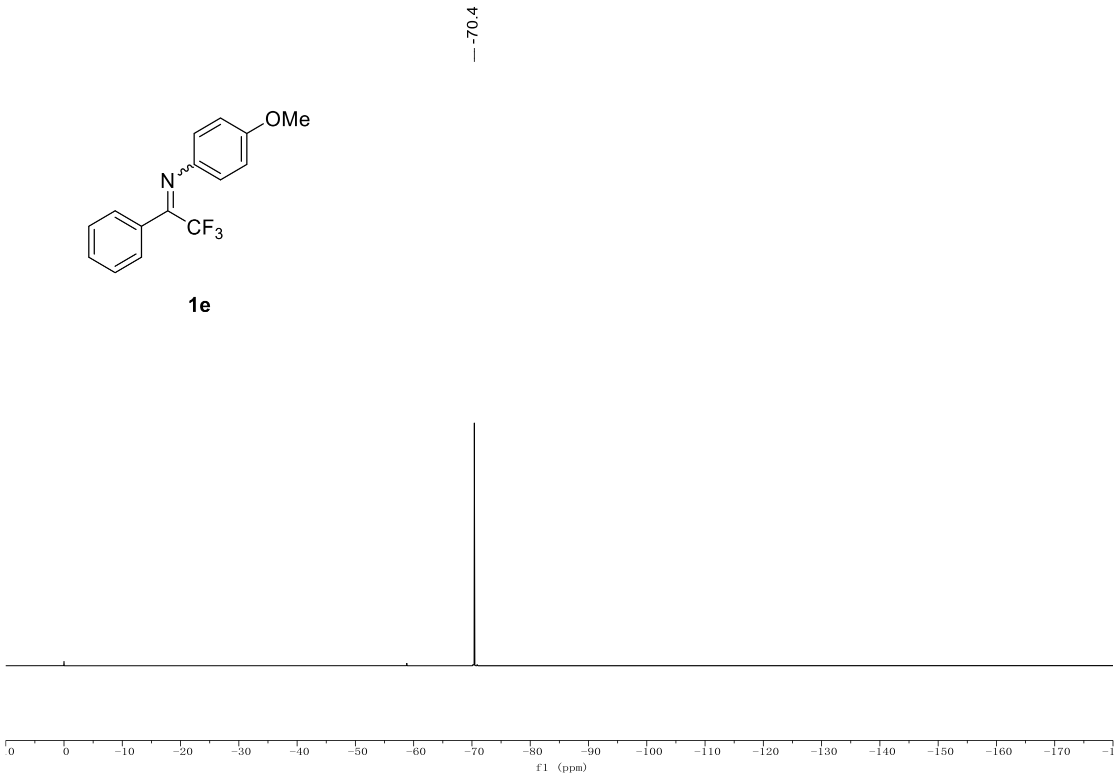

Figure S14. ^{19}F NMR (376 MHz, $CDCl_3$) spectrum of compound 1e, related to Scheme 2

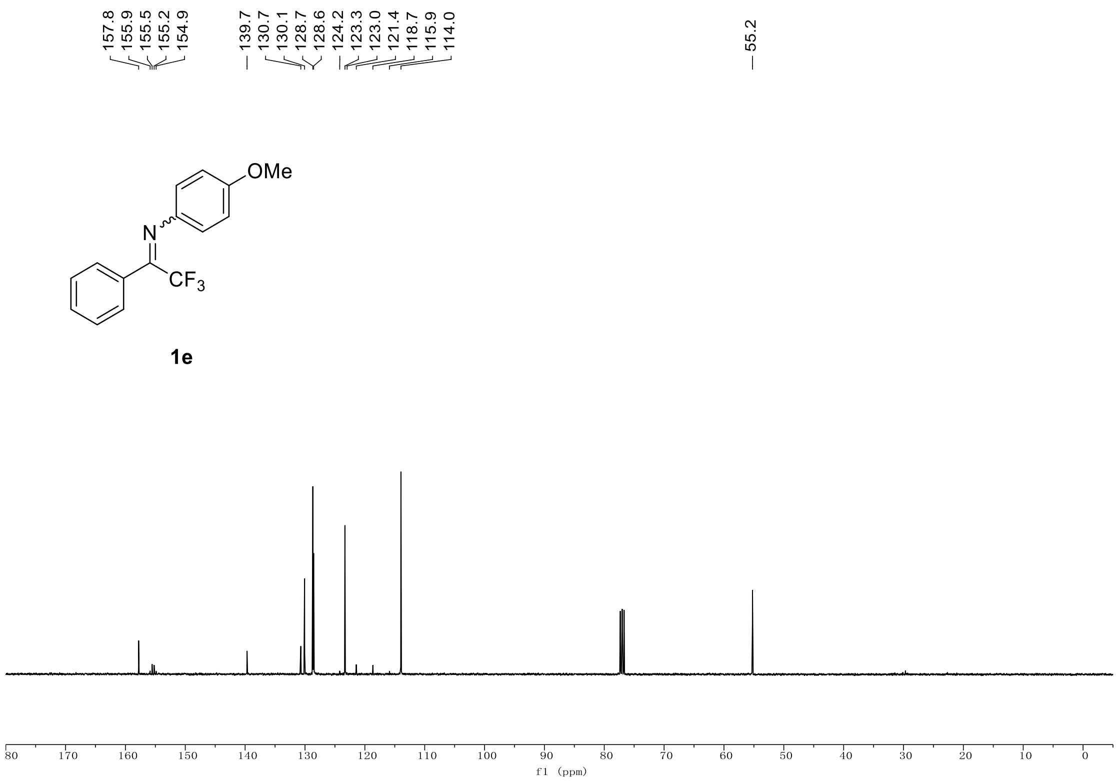

Figure S15. ^{13}C NMR (101 MHz, $CDCl_3$) spectrum of compound 1e, related to Scheme 2

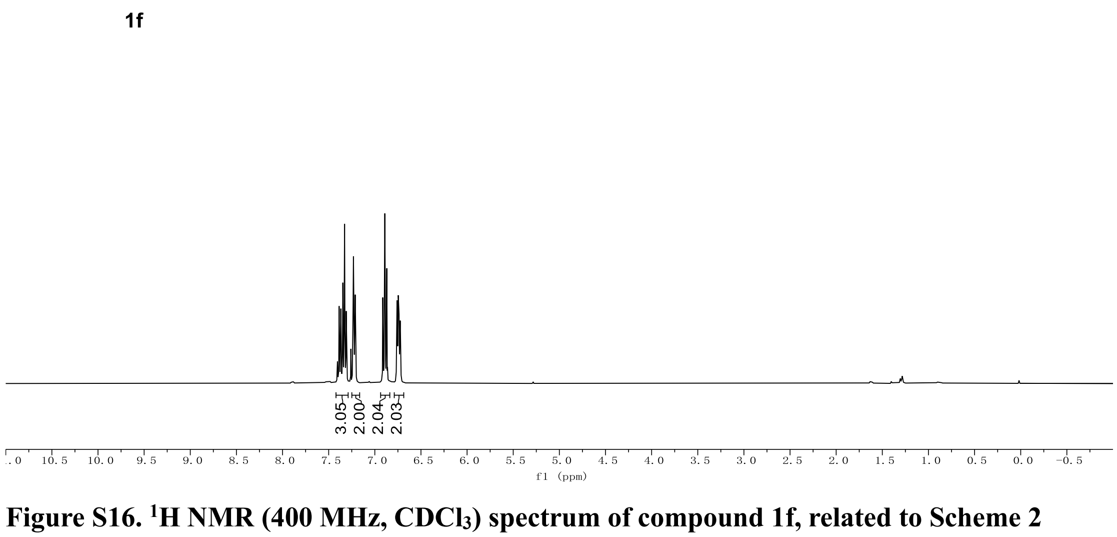

Figure S16. ^{1}H NMR (400 MHz, $CDCl_3$) spectrum of compound 1f, related to Scheme 2

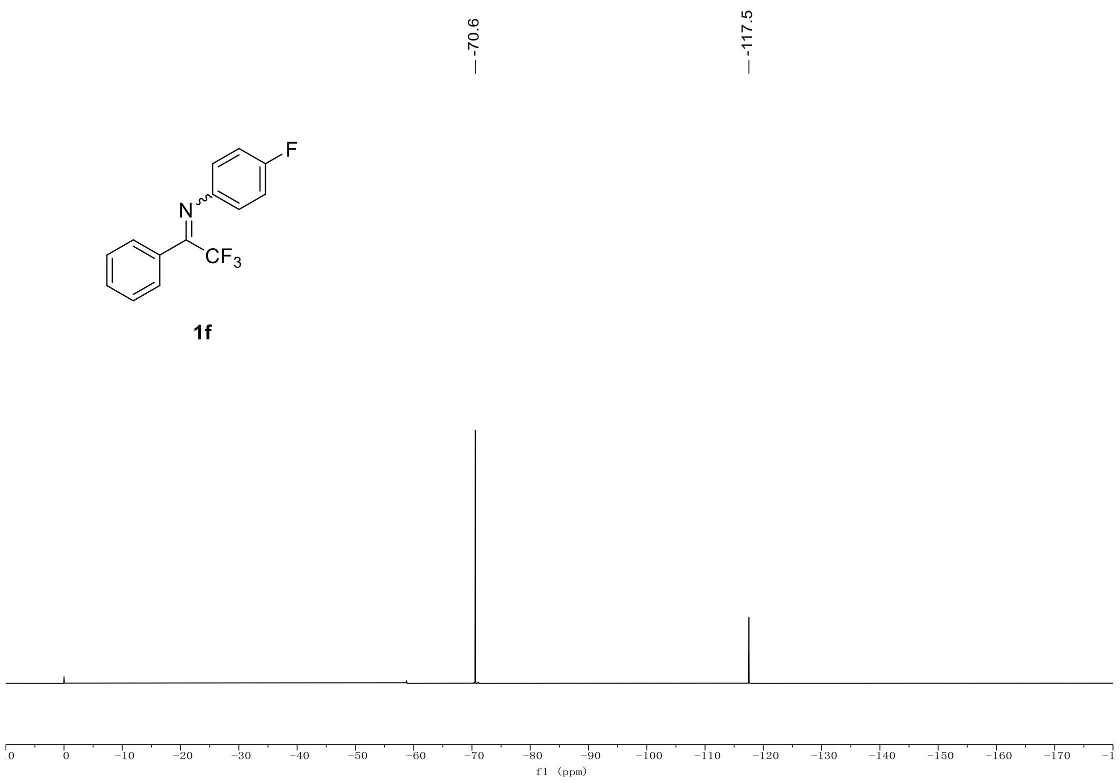

Figure S17. ^{19}F **NMR (376 MHz,** $CDCl_3$**) spectrum of compound 1f, related to Scheme 2**

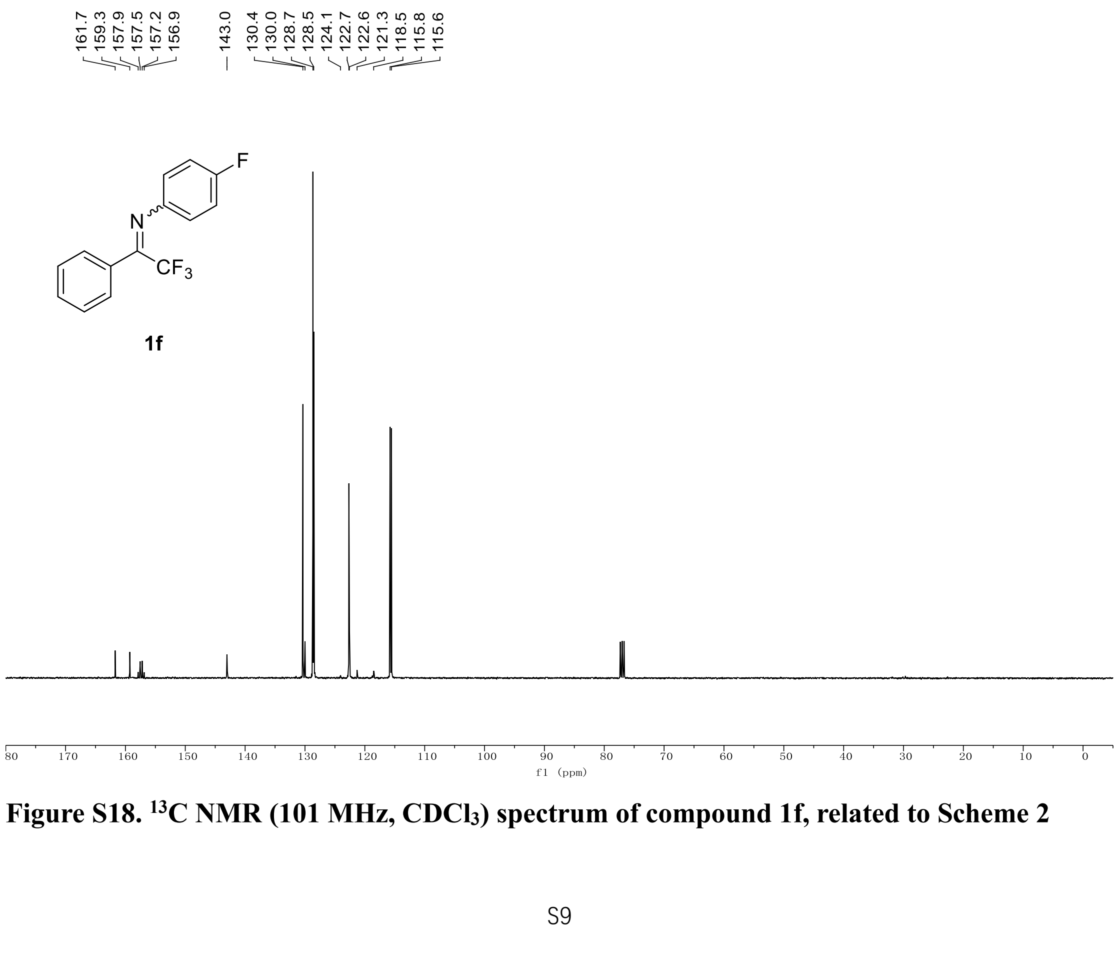

Figure S18. ^{13}C **NMR (101 MHz,** $CDCl_3$**) spectrum of compound 1f, related to Scheme 2**

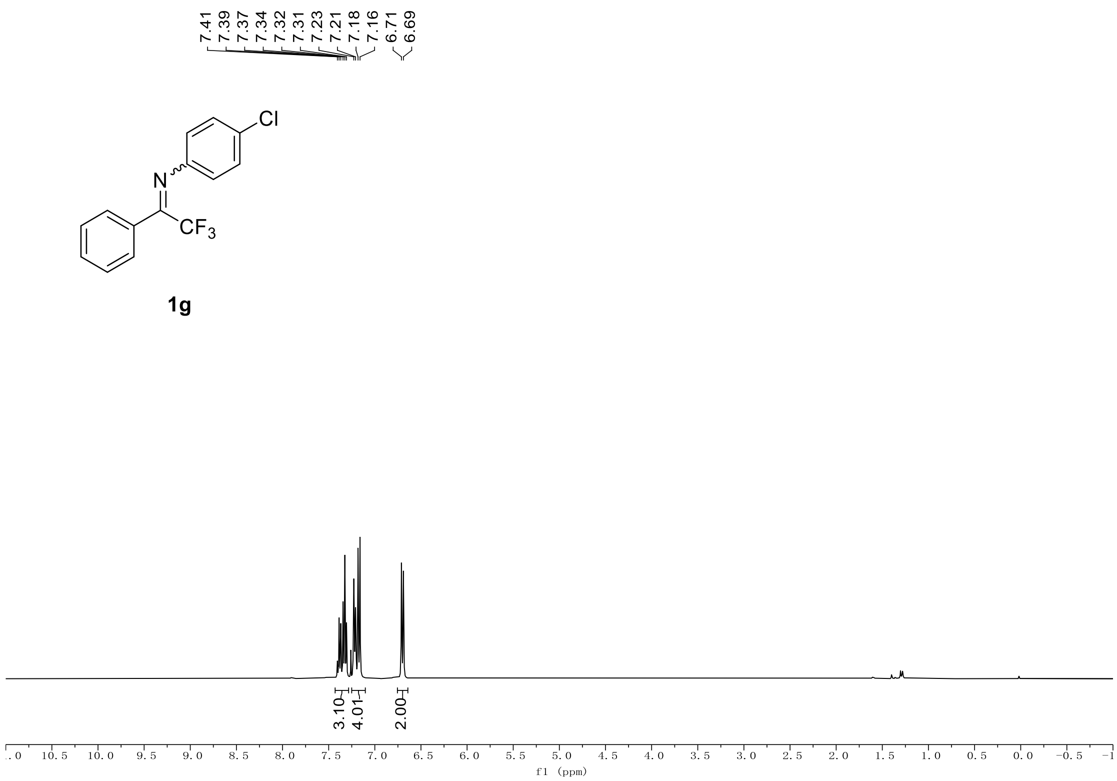

Figure S19. ^{1}H NMR (400 MHz, $CDCl_3$) spectrum of compound 1g, related to Scheme 2

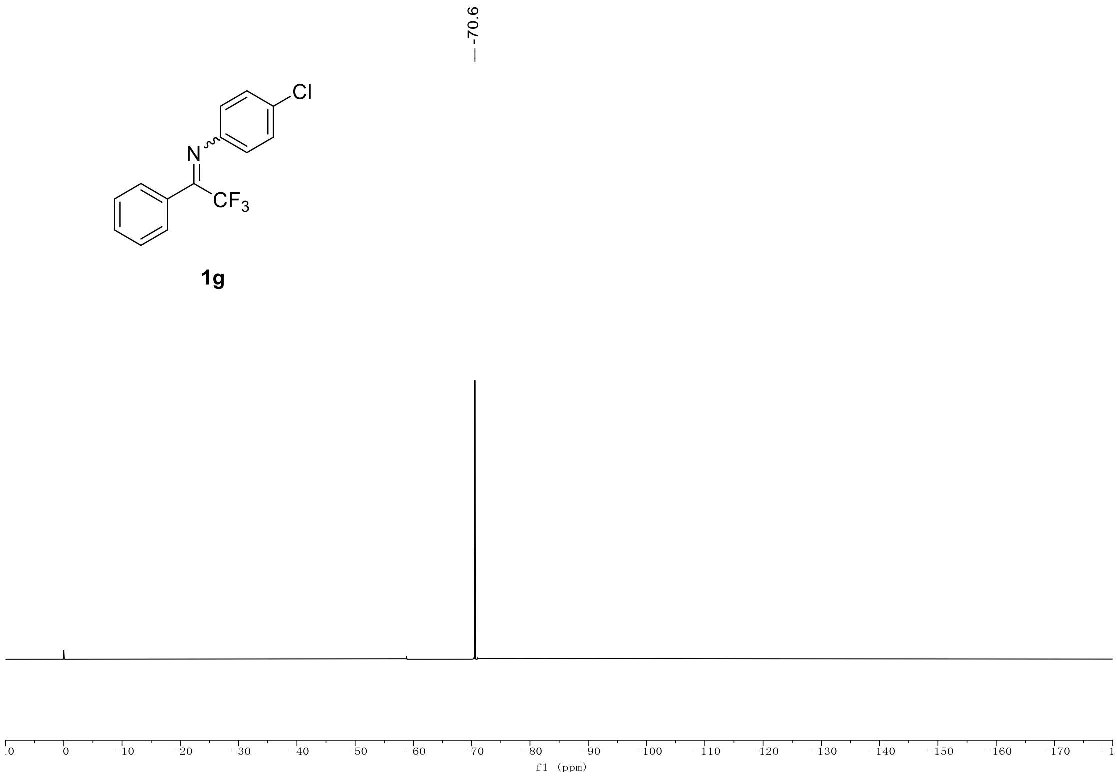

Figure S20. ^{19}F NMR (376 MHz, $CDCl_3$) spectrum of compound 1g, related to Scheme 2

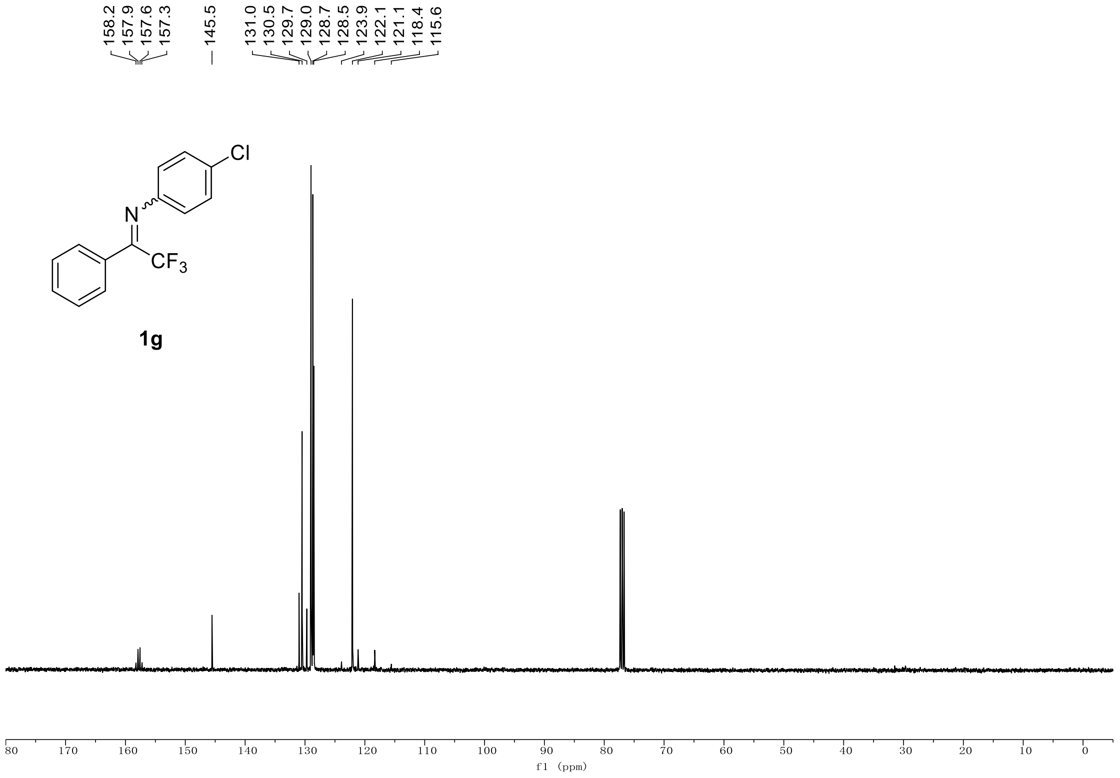

Figure S21. ^{13}**C NMR (101 MHz, CDCl$_3$) spectrum of compound 1g, related to Scheme 2**

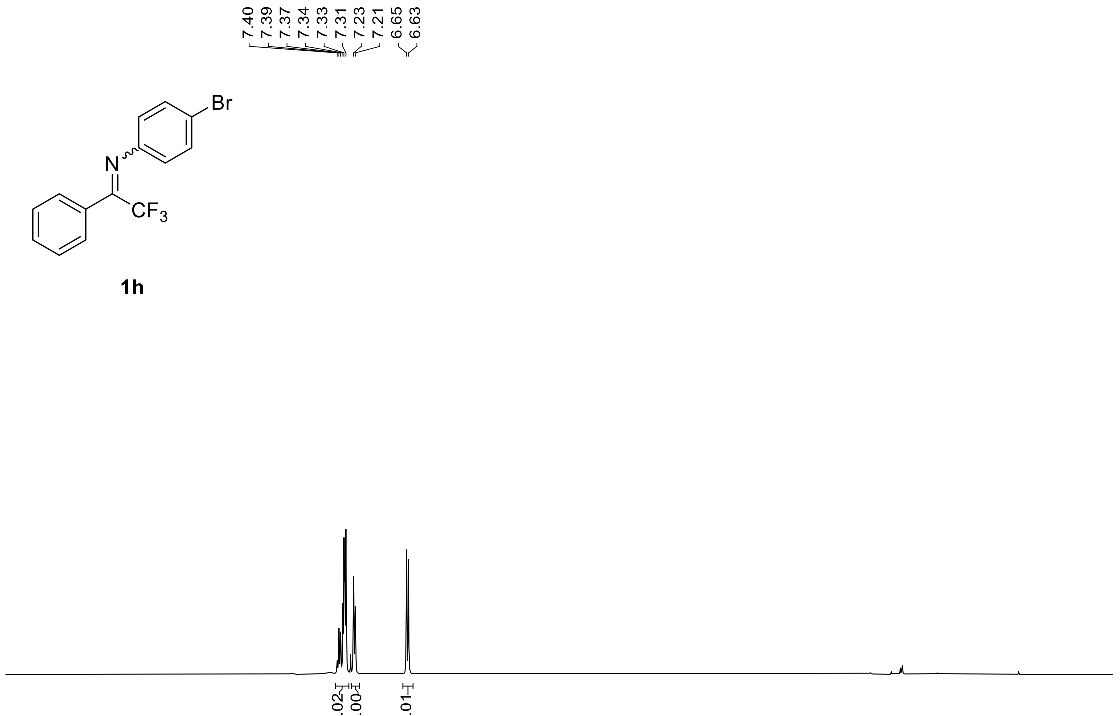

Figure S22. ^{1}**H NMR (400 MHz, CDCl$_3$) spectrum of compound 1h, related to Scheme 2**

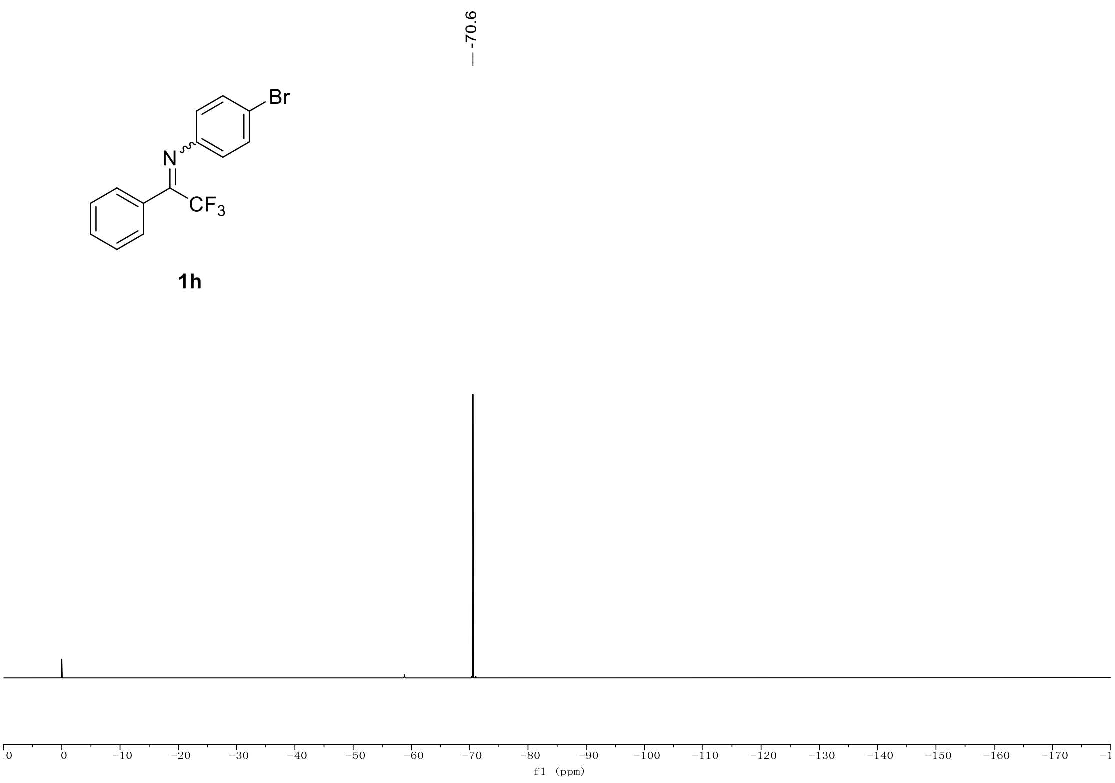

Figure S23. ^{19}F NMR (376 MHz, $CDCl_3$) spectrum of compound 1h, related to Scheme 2

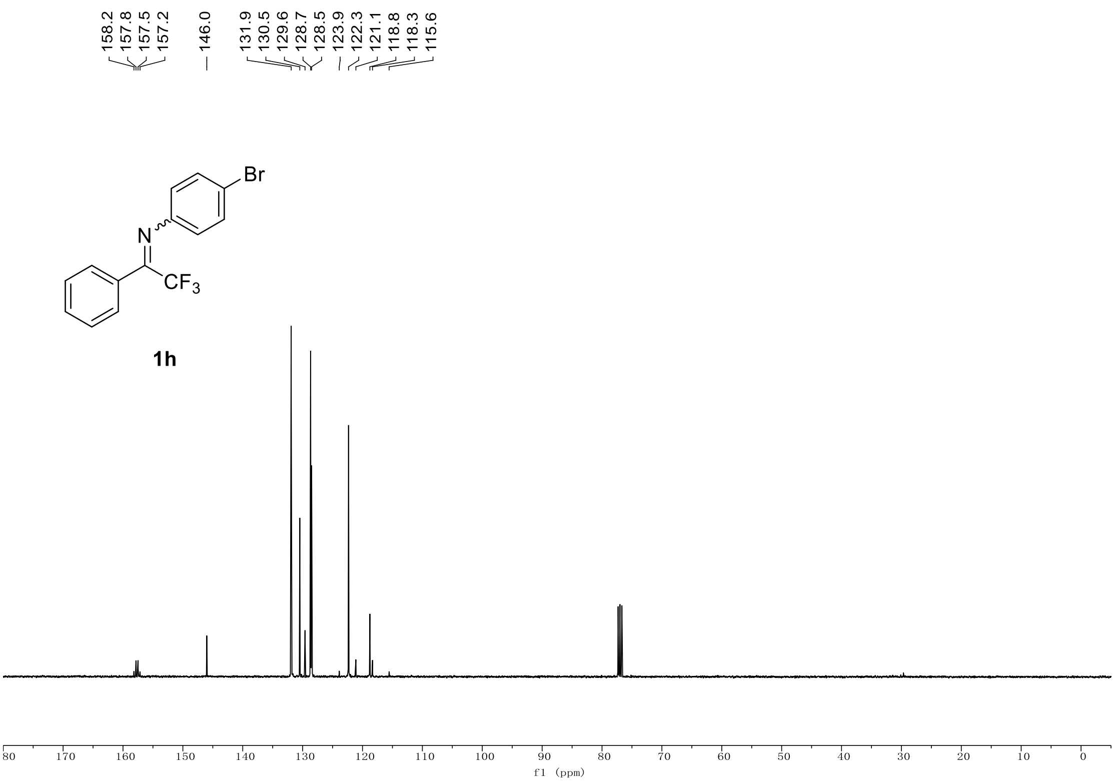

Figure S24. ^{13}C NMR (101 MHz, $CDCl_3$) spectrum of compound 1h, related to Scheme 2

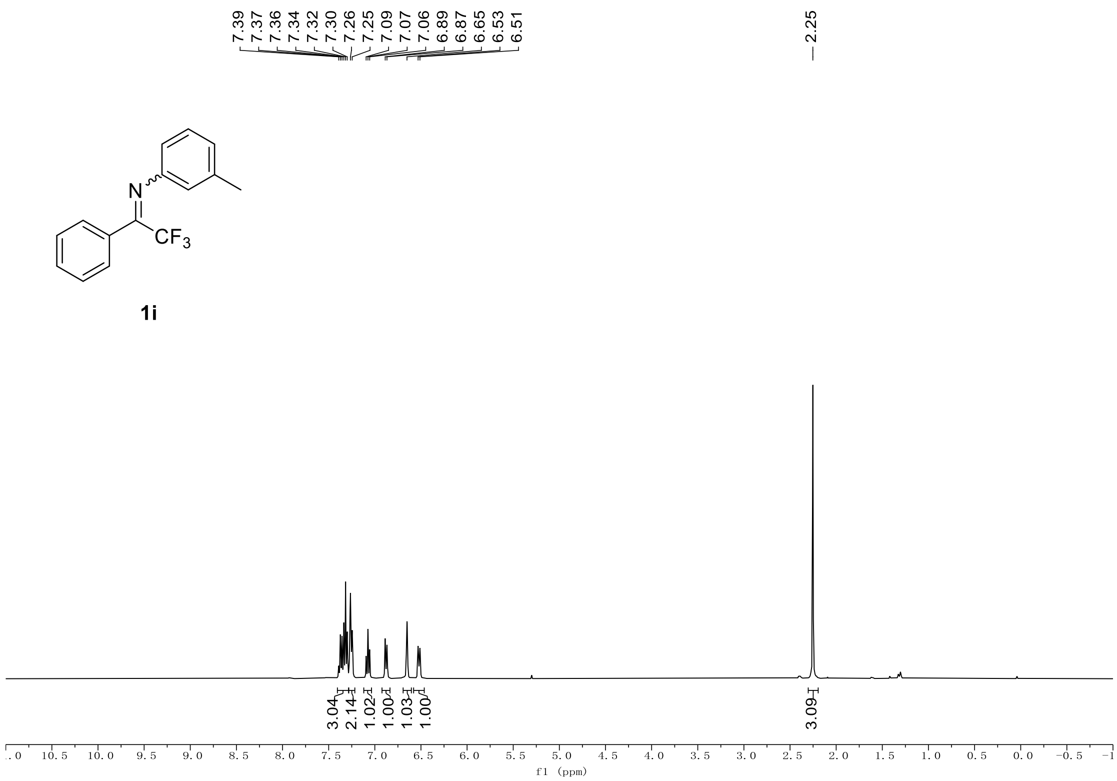

Figure S25. ^{1}H NMR (400 MHz, $CDCl_3$) spectrum of compound 1i, related to Scheme 2

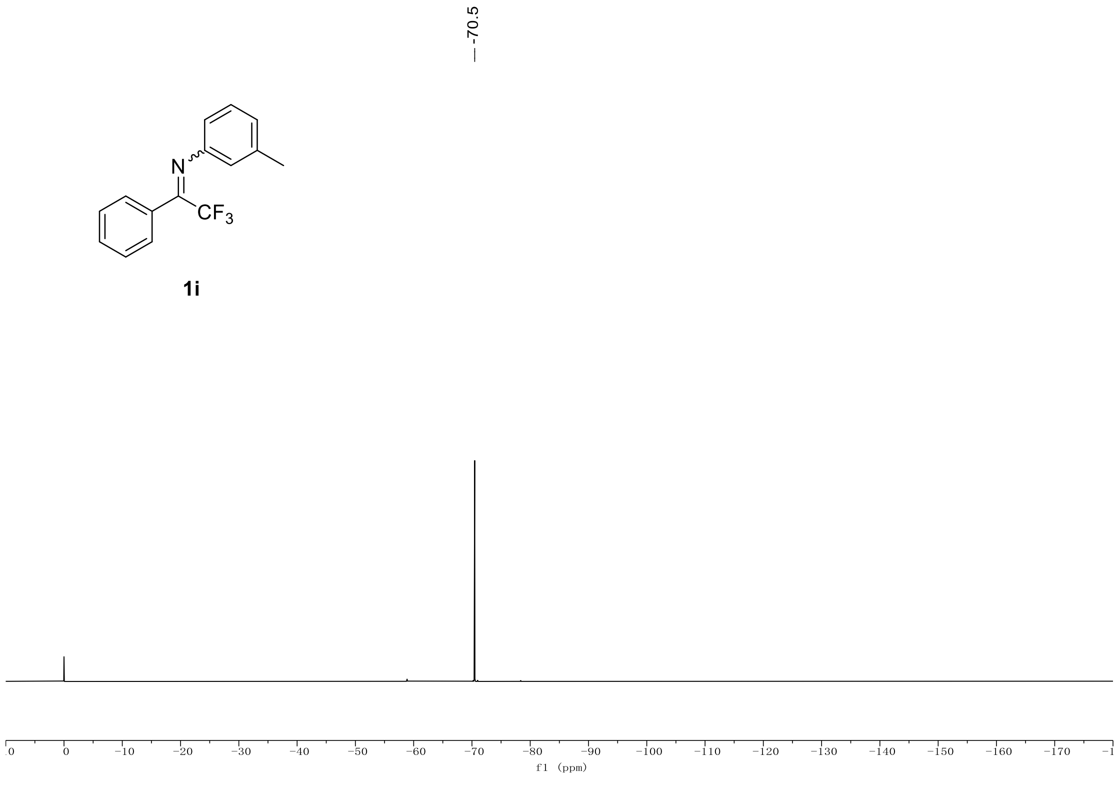

Figure S26. ^{19}F NMR (376 MHz, $CDCl_3$) spectrum of compound 1i, related to Scheme 2

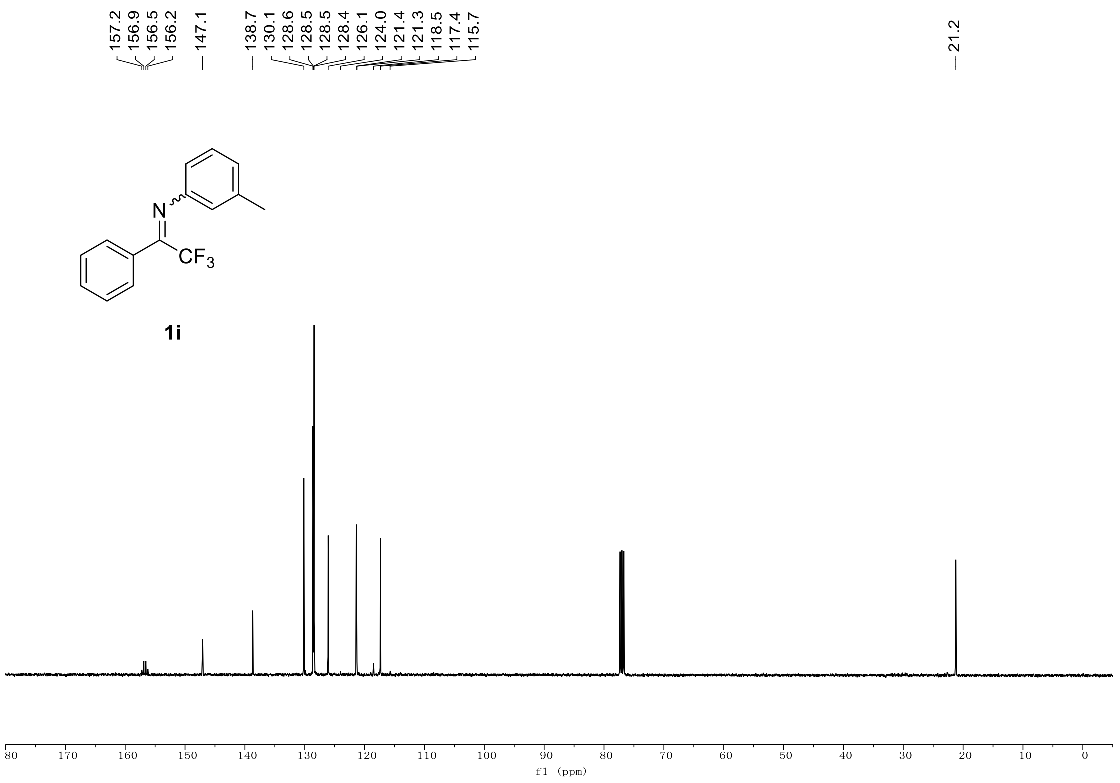

Figure S27. ^{13}C NMR (101 MHz, $CDCl_3$) spectrum of compound 1i, related to Scheme 2

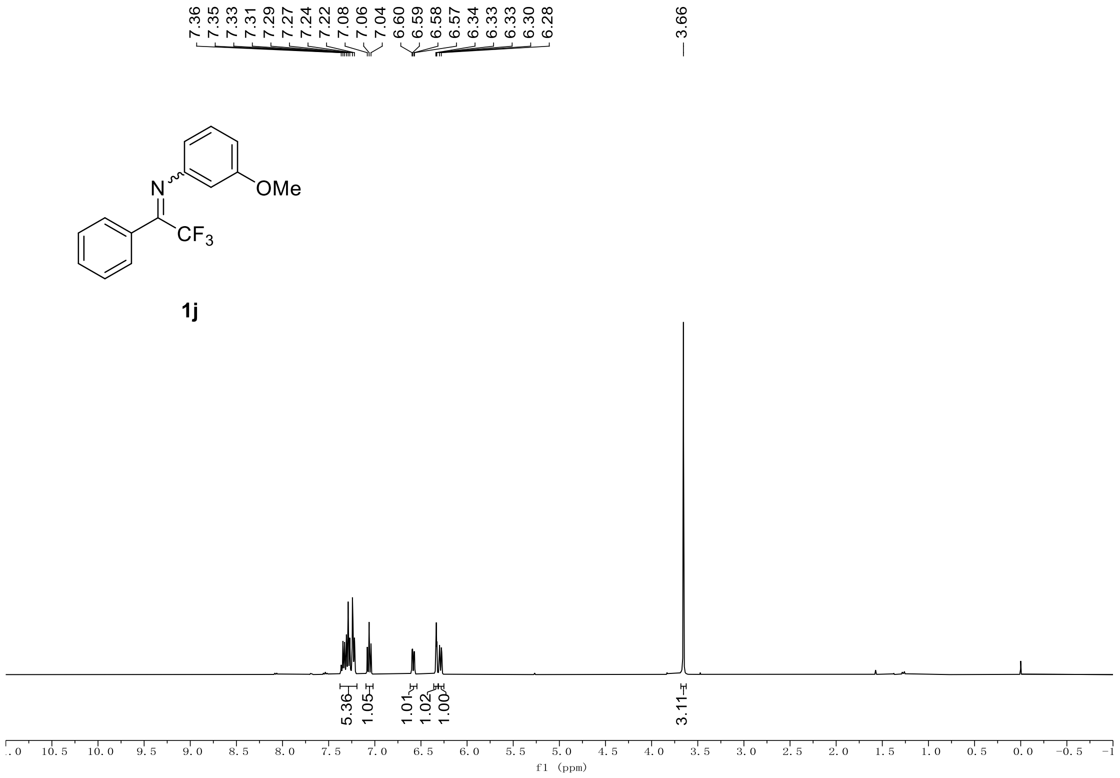

Figure S28. ^{1}H NMR (400 MHz, $CDCl_3$) spectrum of compound 1j, related to Scheme 2

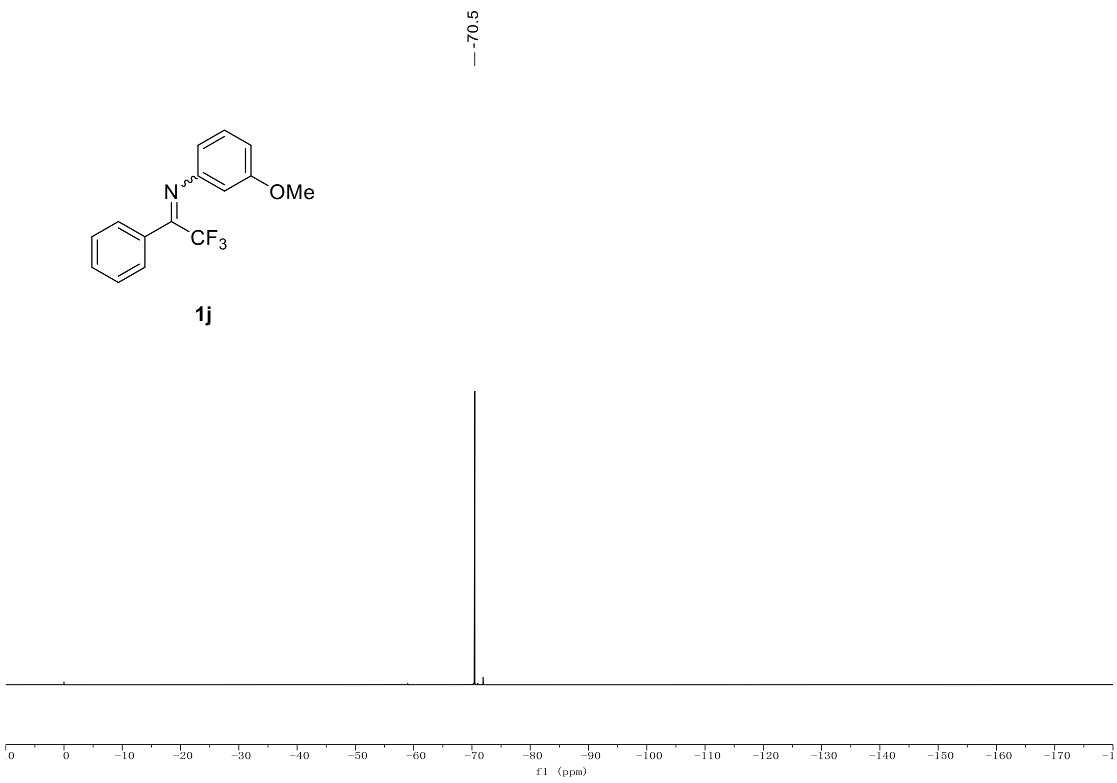

Figure S29. ^{19}F NMR (376 MHz, $CDCl_3$) spectrum of compound 1j, related to Scheme 2

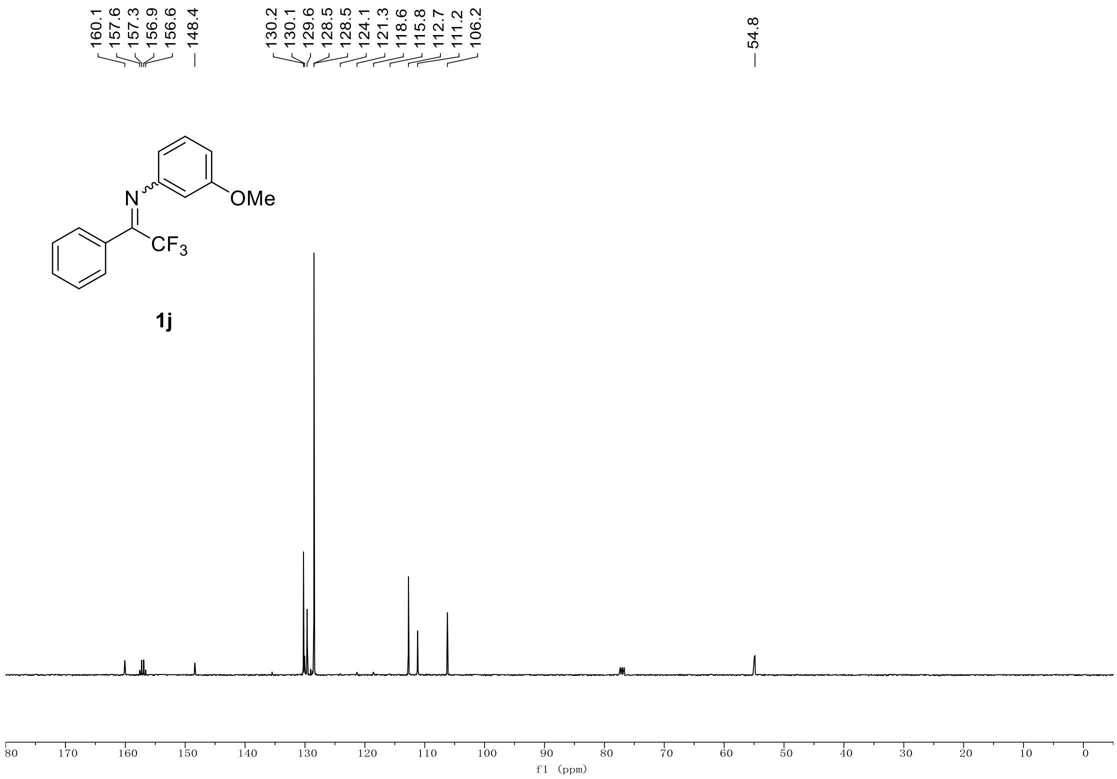

Figure S30. ^{13}C NMR (101 MHz, $CDCl_3$) spectrum of compound 1j, related to Scheme 2

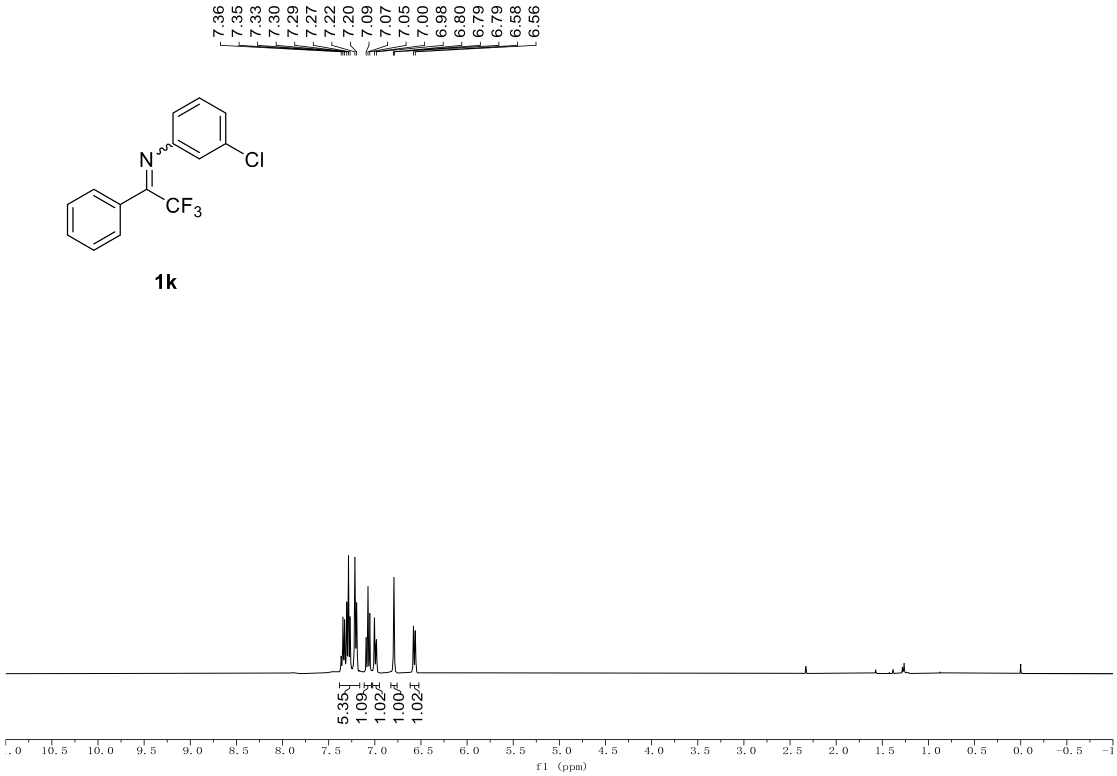

Figure S31. ^{1}H NMR (400 MHz, $CDCl_3$) spectrum of compound 1k, related to Scheme 2

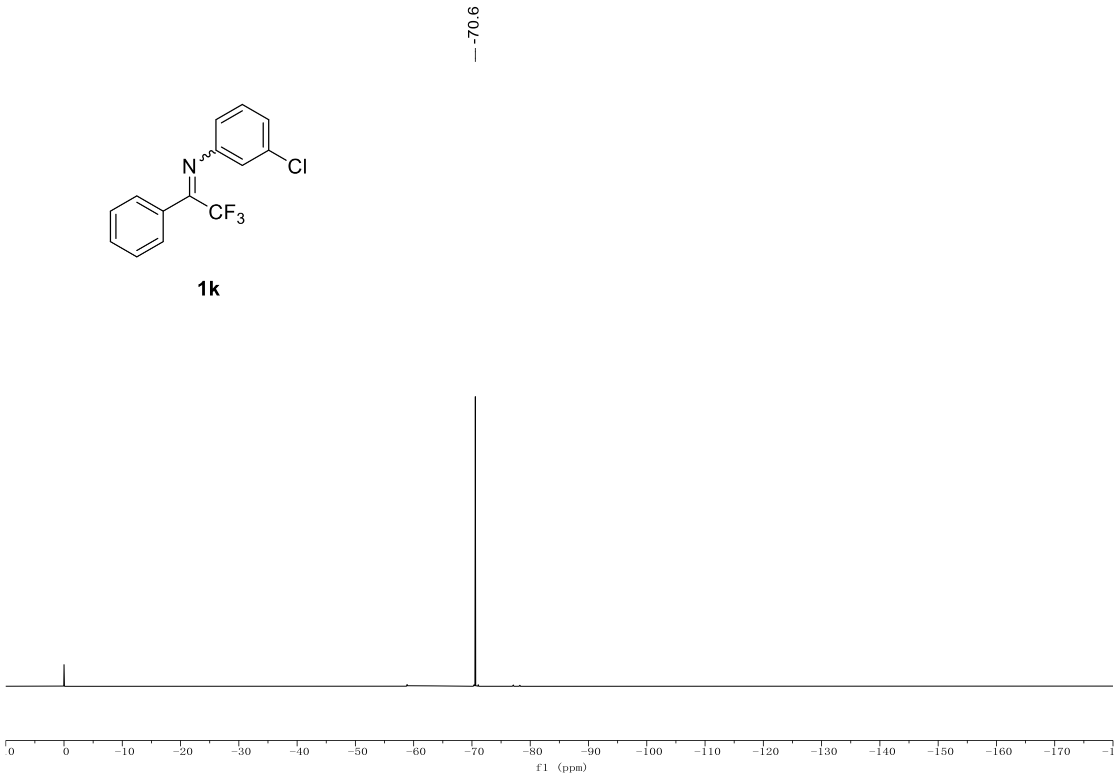

Figure S32. ^{19}F NMR (376 MHz, $CDCl_3$) spectrum of compound 1k, related to Scheme 2

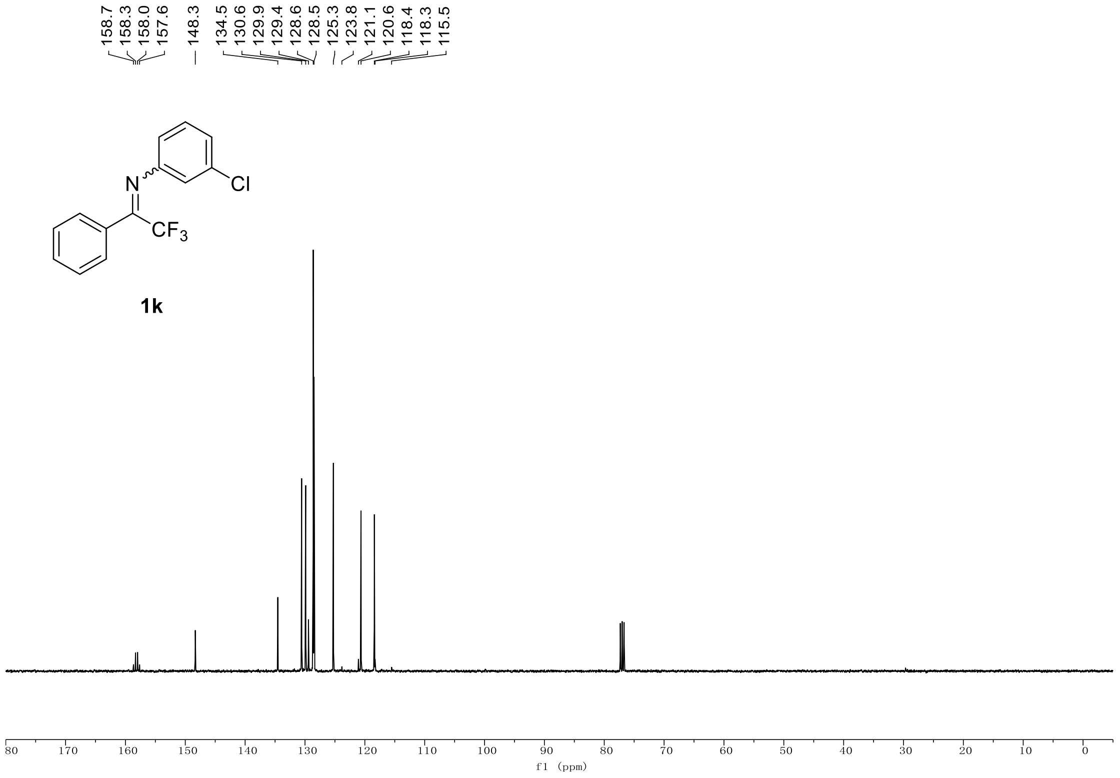

Figure S33. ^{13}C NMR (101 MHz, $CDCl_3$) spectrum of compound 1k, related to Scheme 2

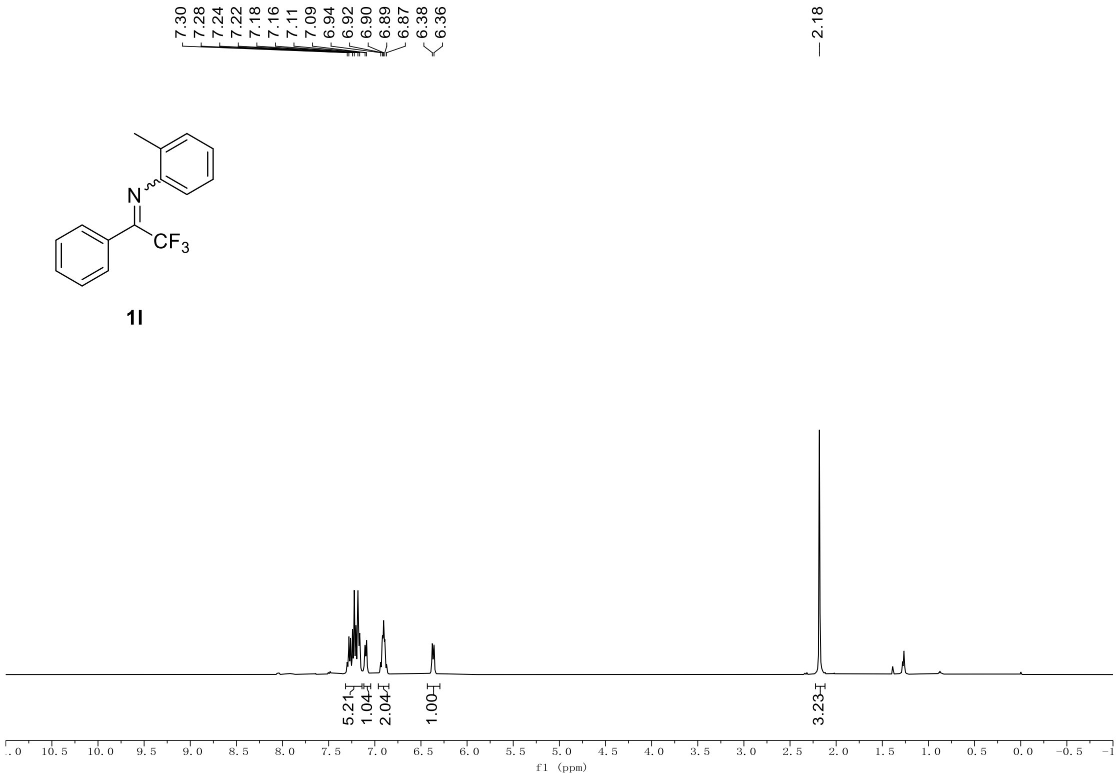

Figure S34. ^{1}H NMR (400 MHz, $CDCl_3$) spectrum of compound 1l, related to Scheme 2

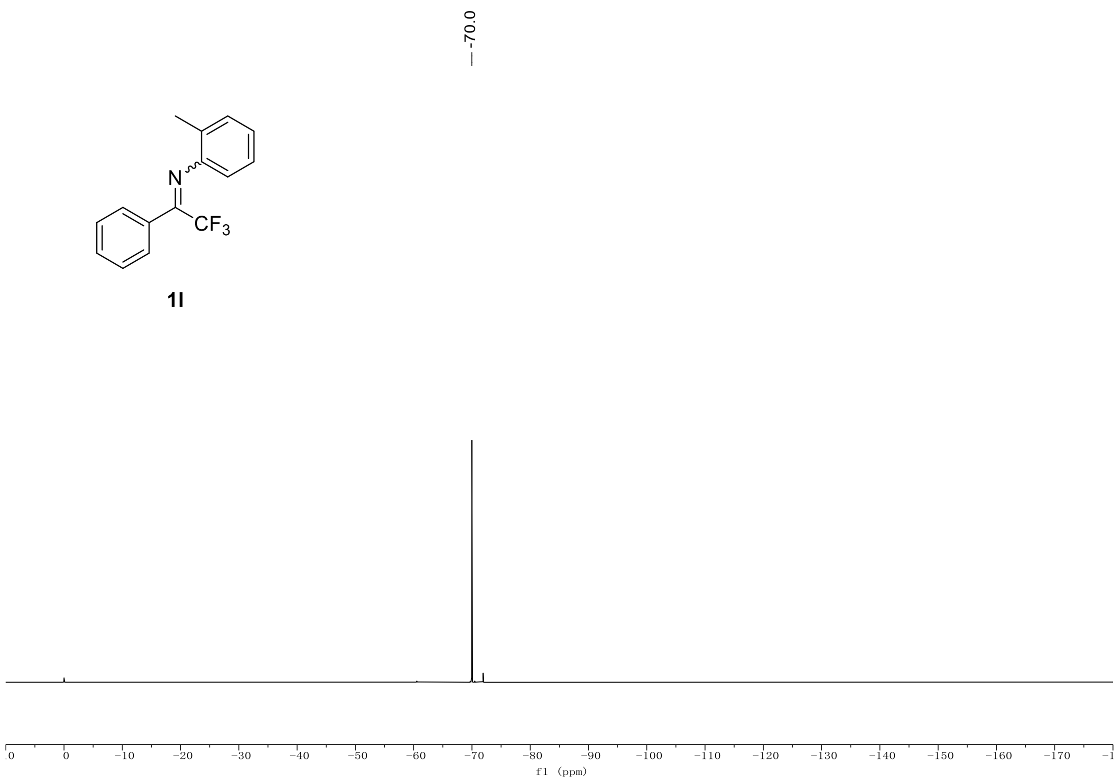

Figure S35. ^{19}F NMR (376 MHz, $CDCl_3$) spectrum of compound 1l, related to Scheme 2

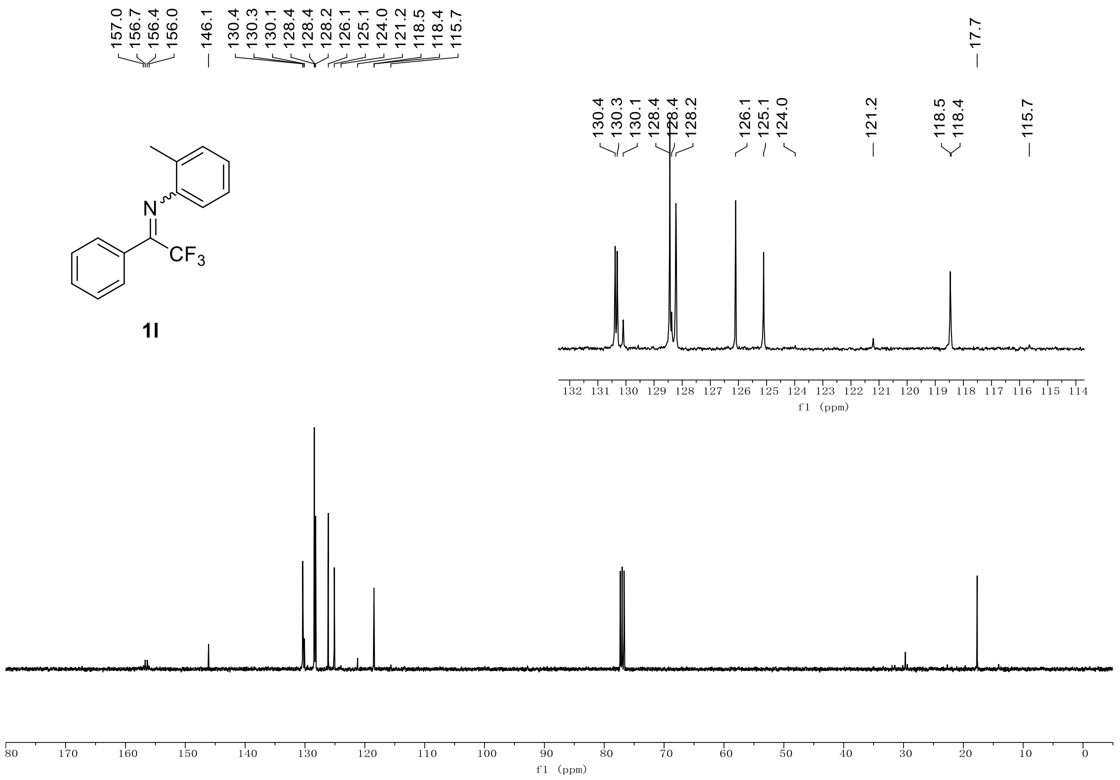

Figure S36. ^{13}C NMR (101 MHz, $CDCl_3$) spectrum of compound 1l, related to Scheme 2

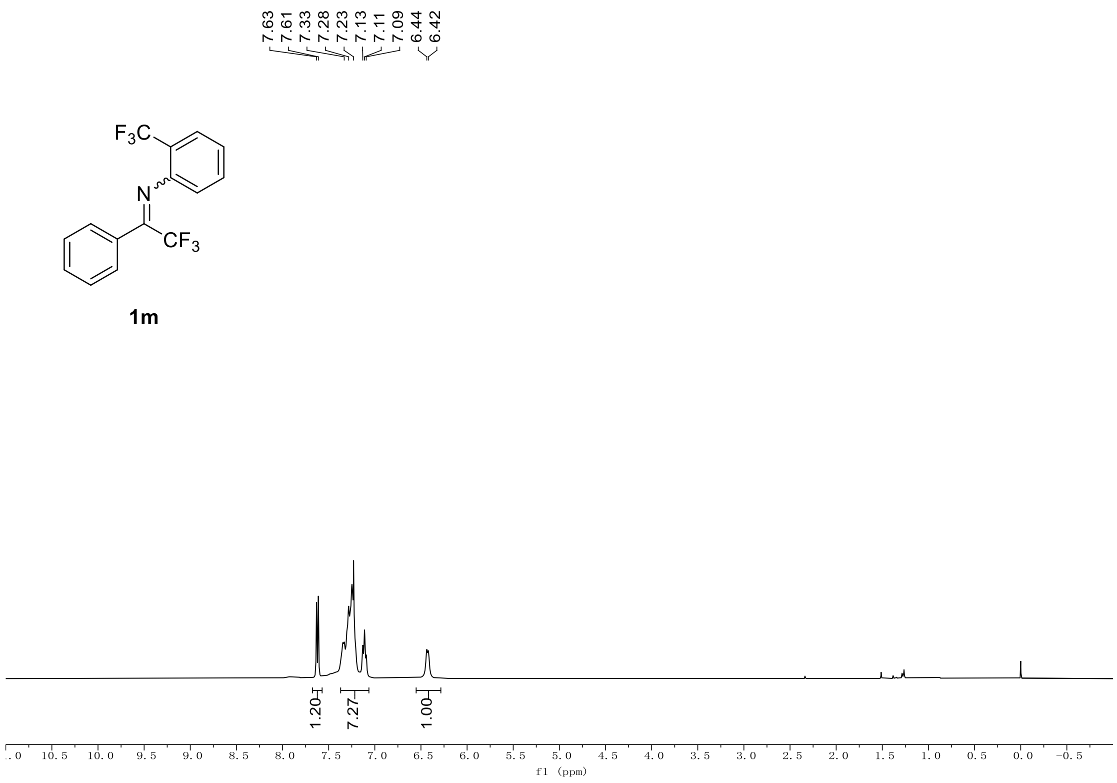

Figure S37. **^{1}H NMR (400 MHz, $CDCl_3$) spectrum of compound 1m, related to Scheme 2**

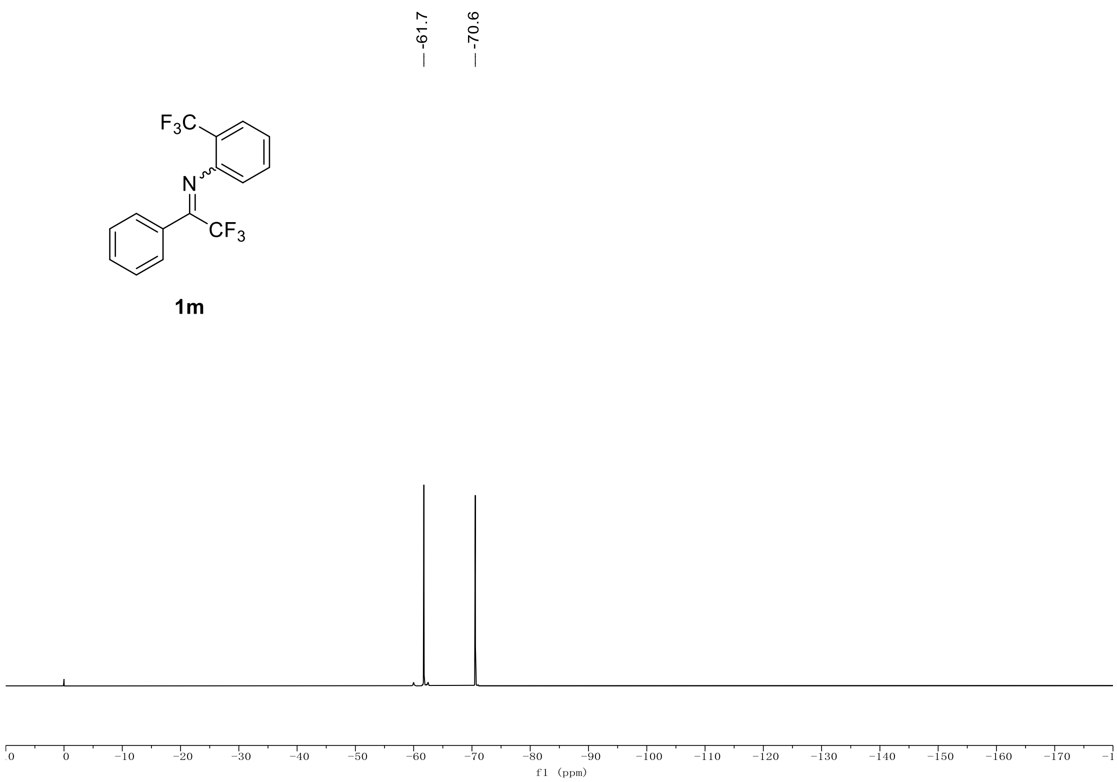

Figure S38. **^{19}F NMR (376 MHz, $CDCl_3$) spectrum of compound 1m, related to Scheme 2**

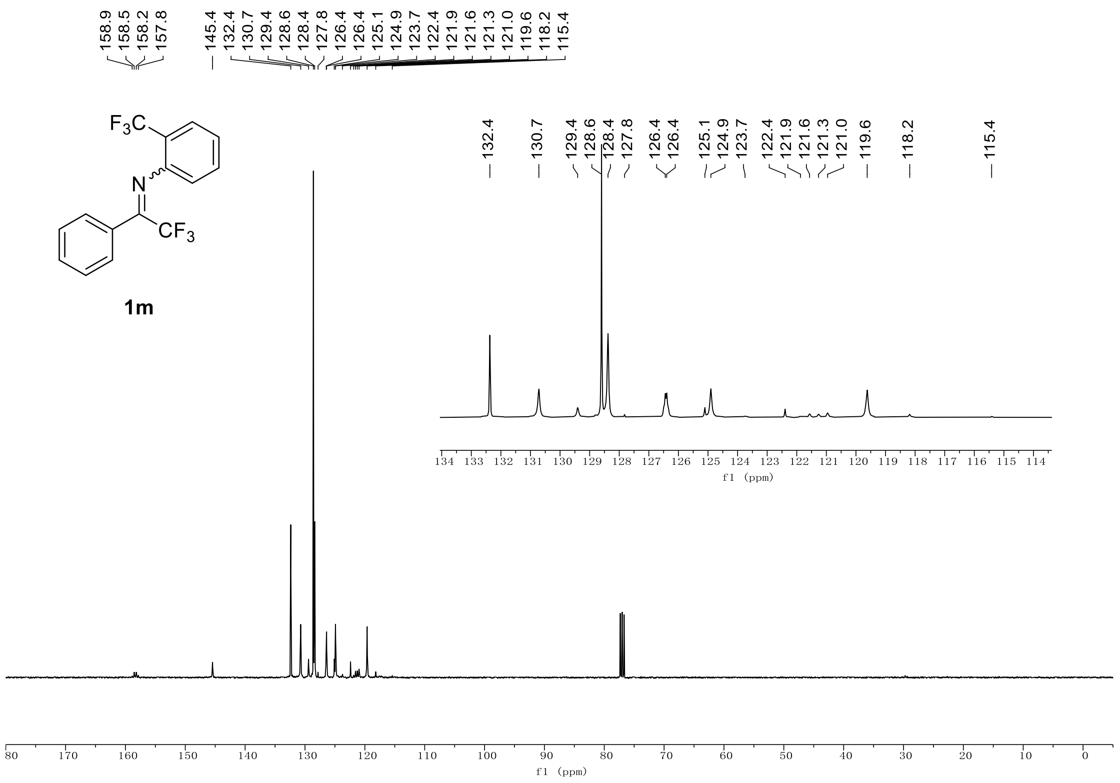

Figure S39. ^{13}C NMR (101 MHz, $CDCl_3$) spectrum of compound 1m, related to Scheme 2

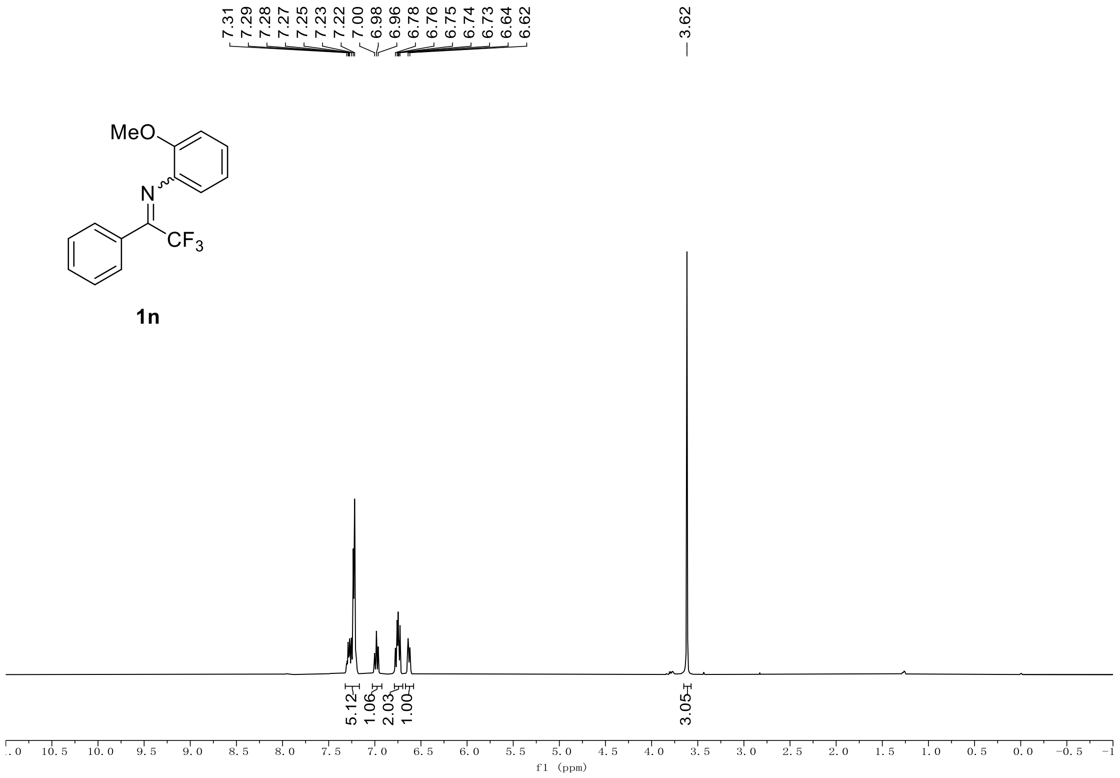

Figure S40. ^{1}H NMR (400 MHz, $CDCl_3$) spectrum of compound 1n, related to Scheme 2

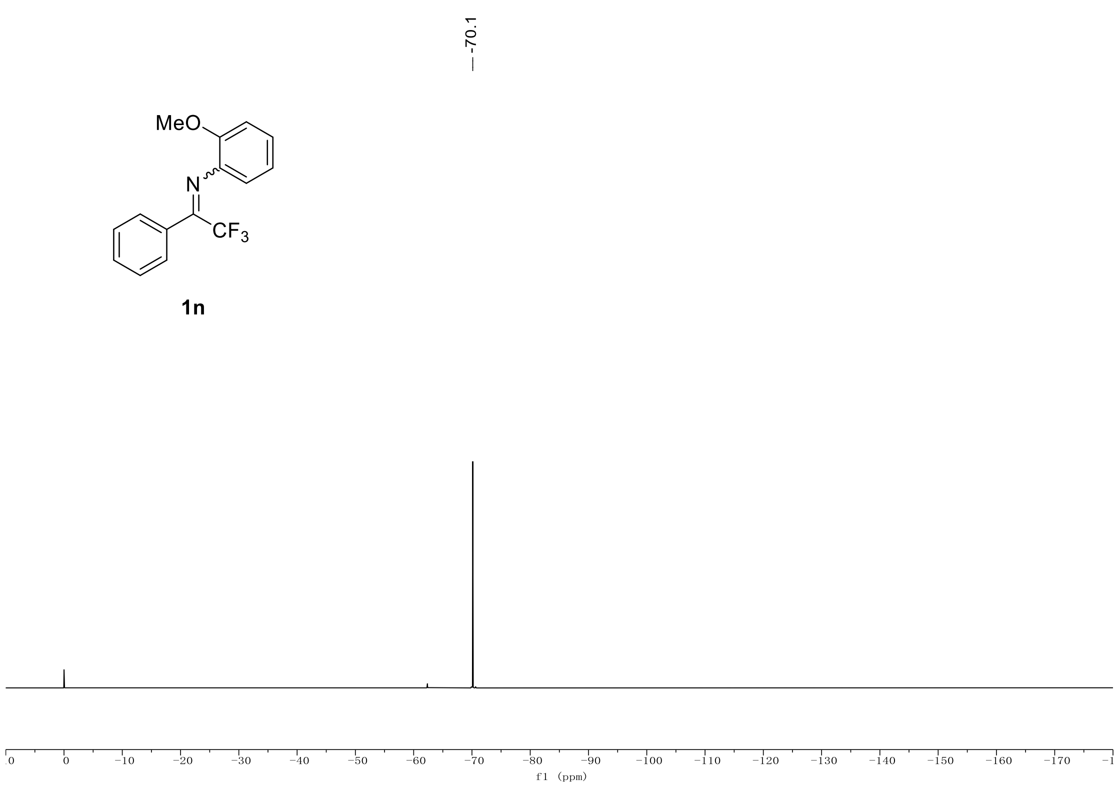

Figure S41. ^{19}F NMR (376 MHz, $CDCl_3$) spectrum of compound 1n, related to Scheme 2

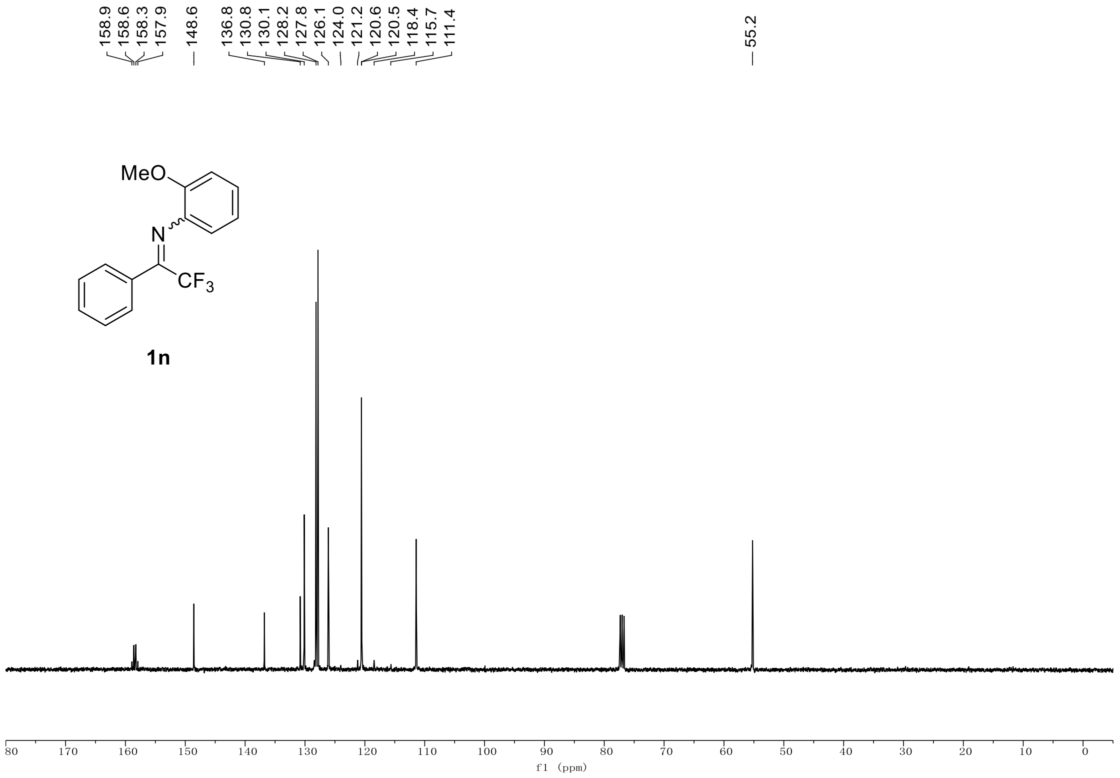

Figure S42. ^{13}C NMR (101 MHz, $CDCl_3$) spectrum of compound 1n, related to Scheme 2

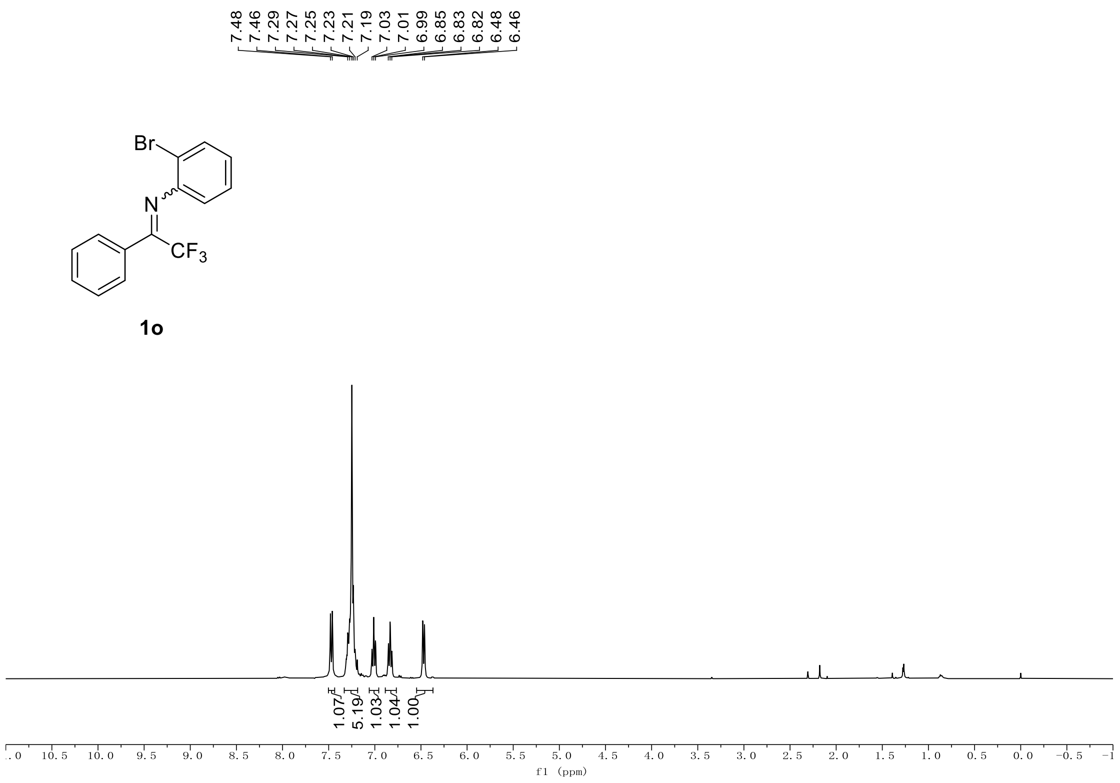

Figure S43. ^{1}H NMR (400 MHz, $CDCl_3$) spectrum of compound 1o, related to Scheme 2

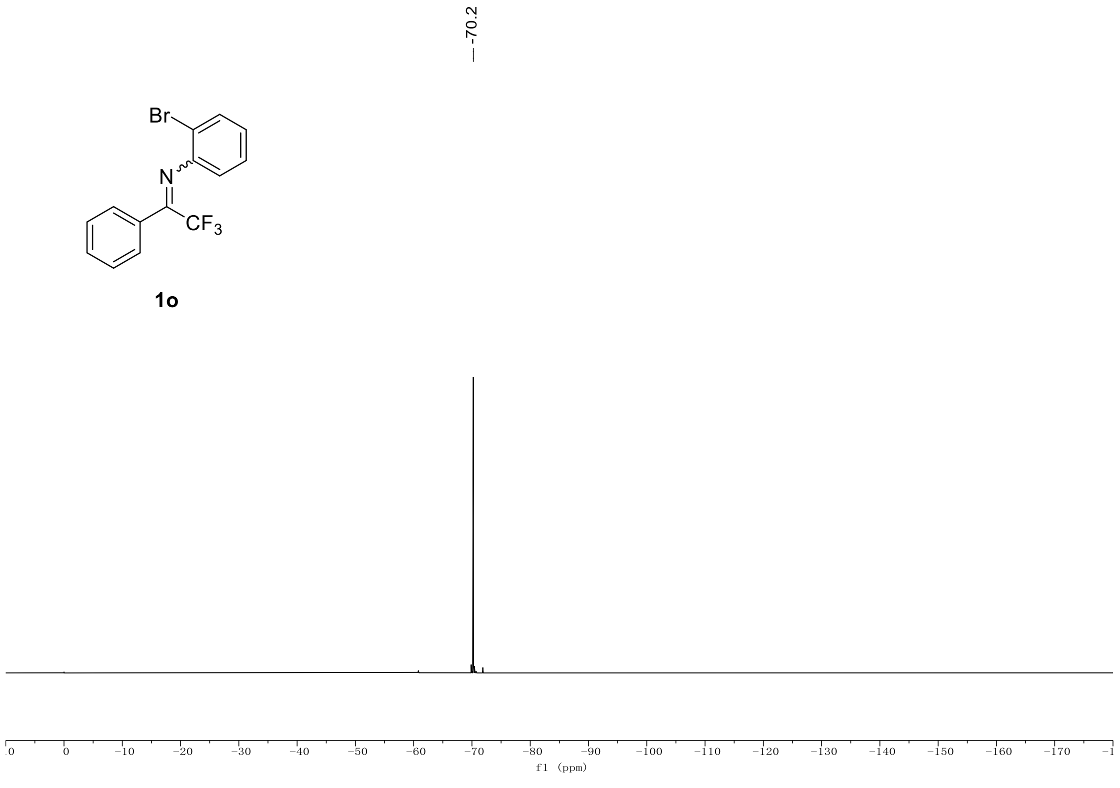

Figure S44. ^{19}F NMR (376 MHz, $CDCl_3$) spectrum of compound 1o, related to Scheme 2

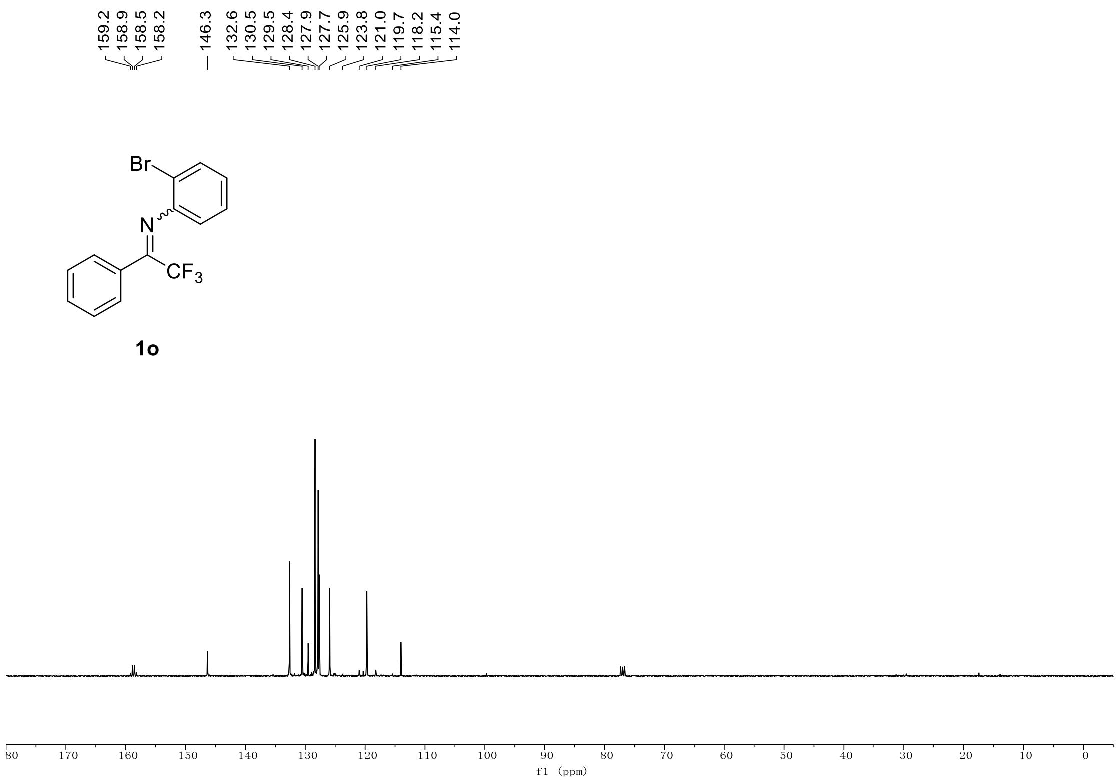

Figure S45. ^{13}C NMR (101 MHz, $CDCl_3$) spectrum of compound 1o, related to Scheme 2

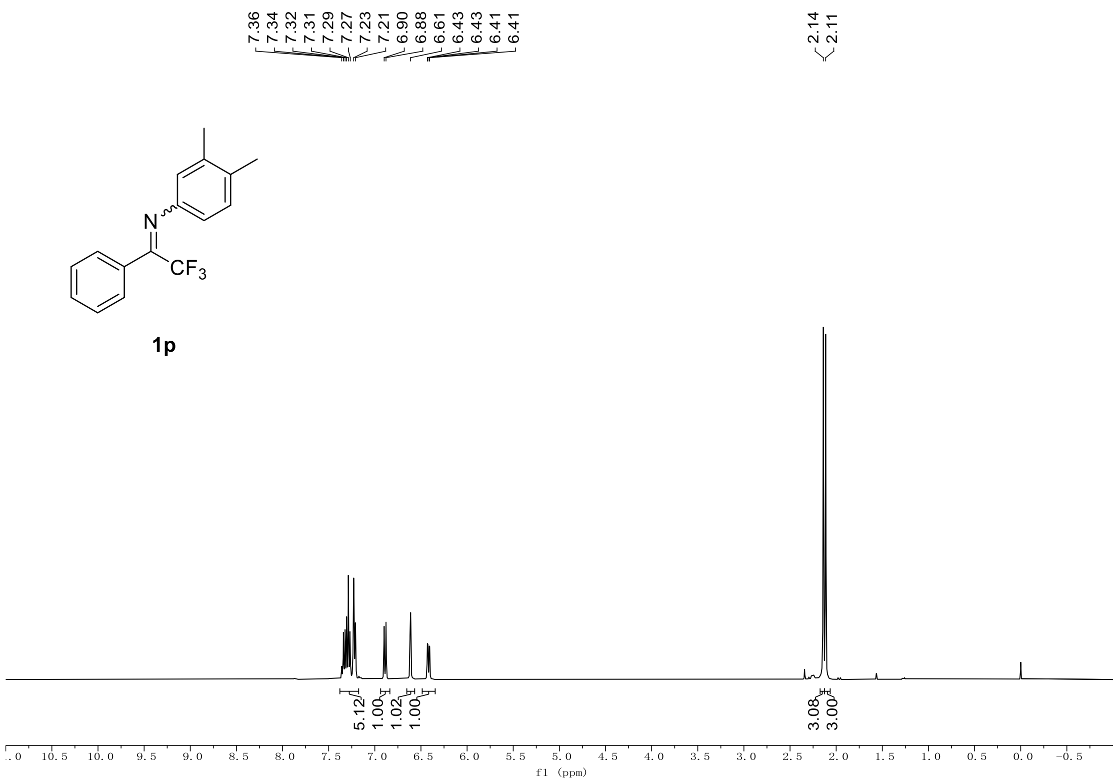

Figure S46. ^{1}H NMR (400 MHz, $CDCl_3$) spectrum of compound 1p, related to Scheme 2

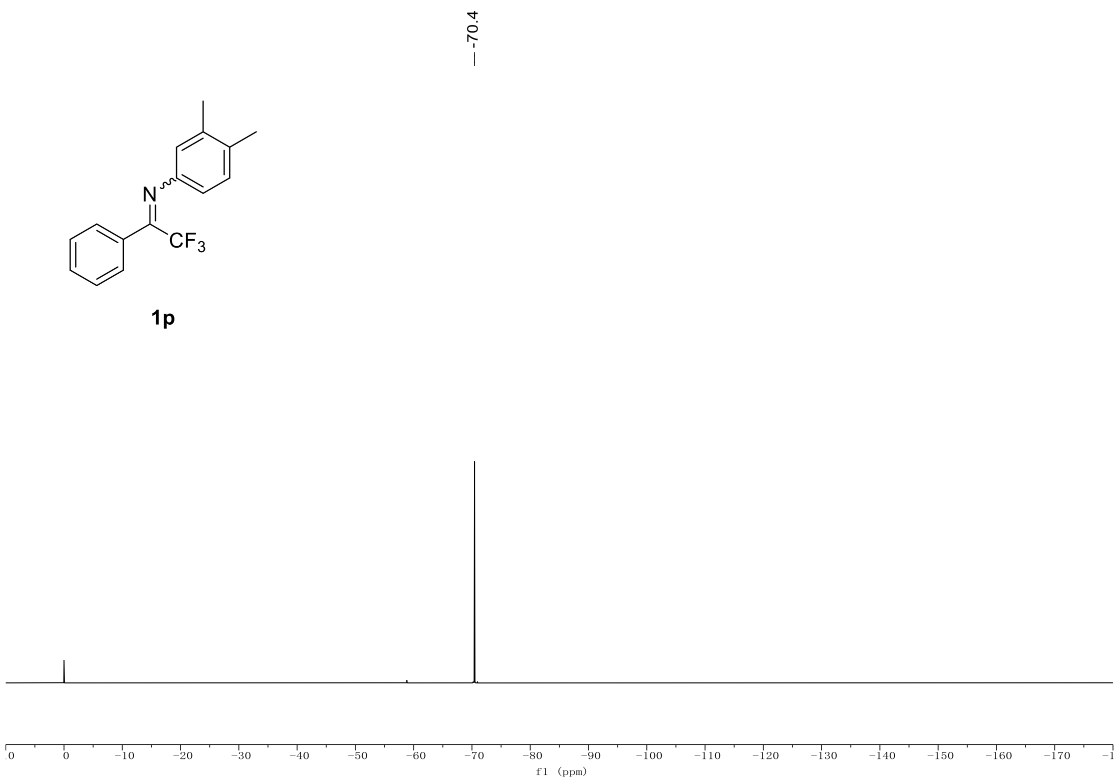

Figure S47. ^{19}F NMR (376 MHz, $CDCl_3$) spectrum of compound 1p, related to Scheme 2

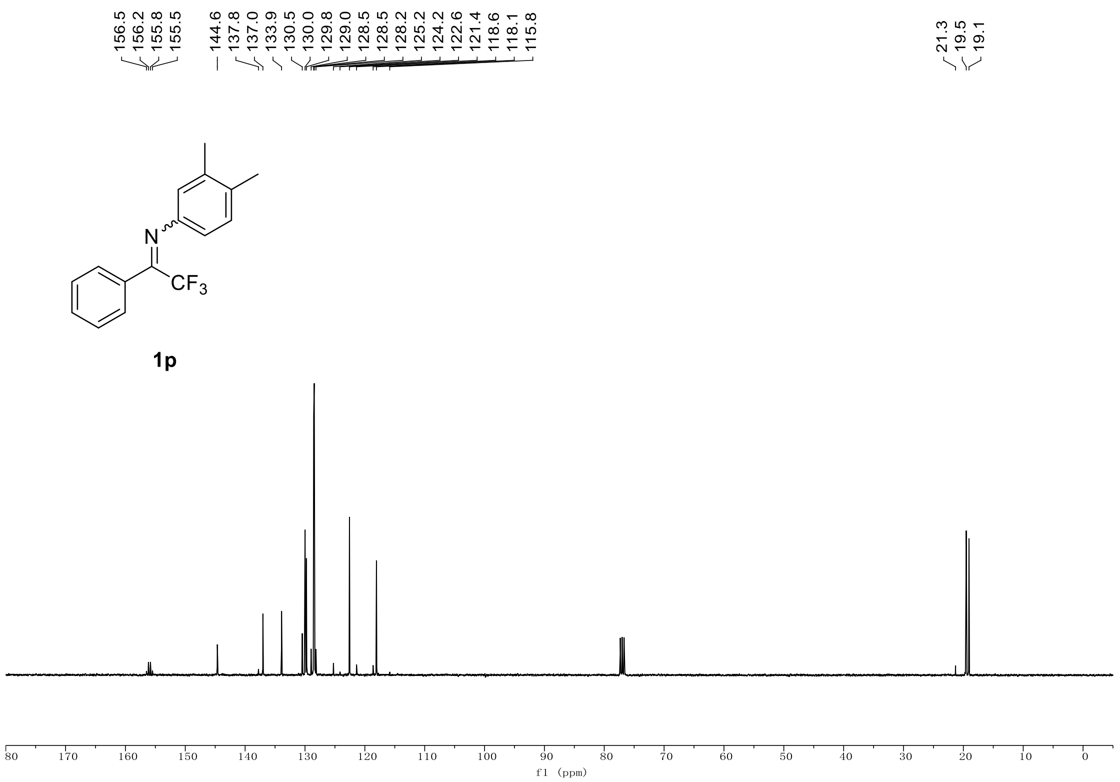

Figure S48. ^{13}C NMR (101 MHz, $CDCl_3$) spectrum of compound 1p, related to Scheme 2

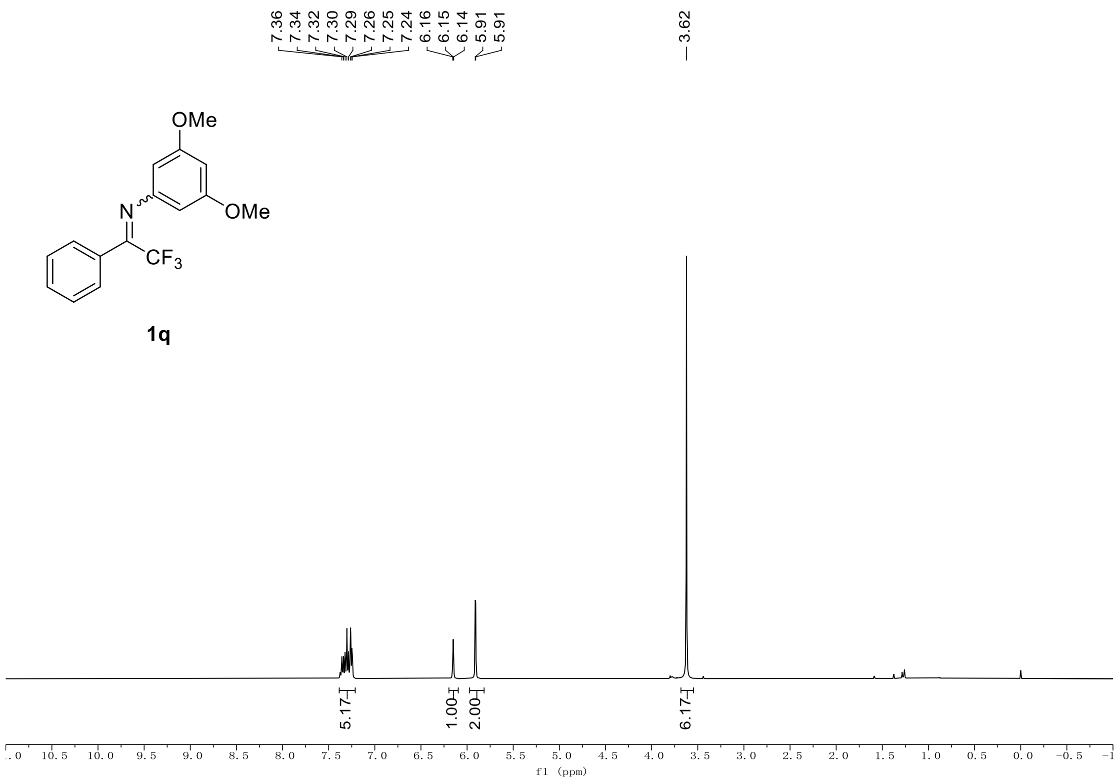

Figure S49. ^{1}H NMR (400 MHz, $CDCl_3$) spectrum of compound 1q, related to Scheme 2

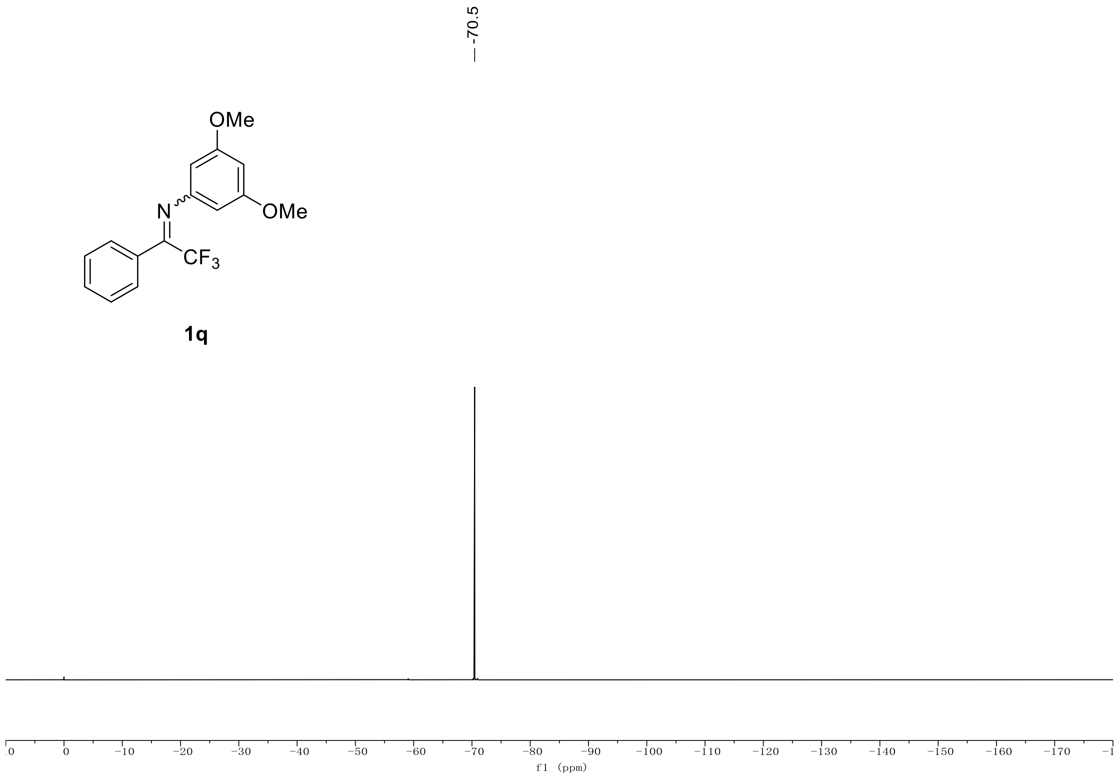

Figure S50. ^{19}F NMR (376 MHz, $CDCl_3$) spectrum of compound 1q, related to Scheme 2

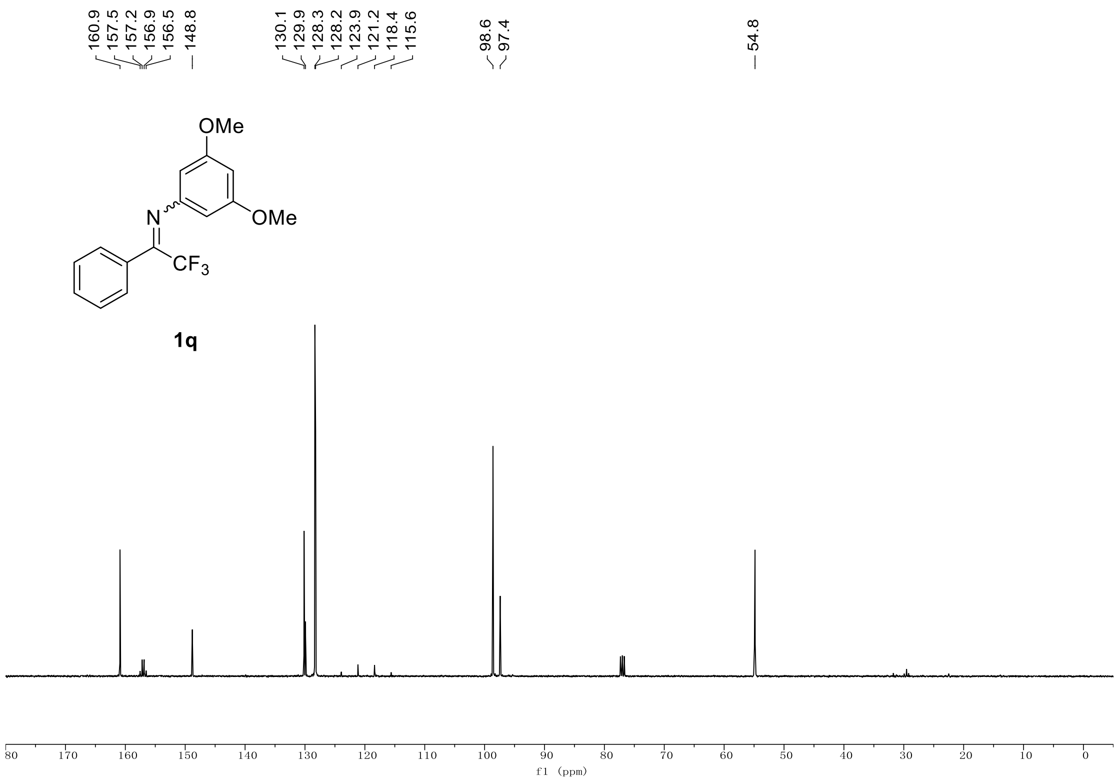

Figure S51. ^{13}**C NMR (101 MHz,** $CDCl_3$**) spectrum of compound 1q, related to Scheme 2**

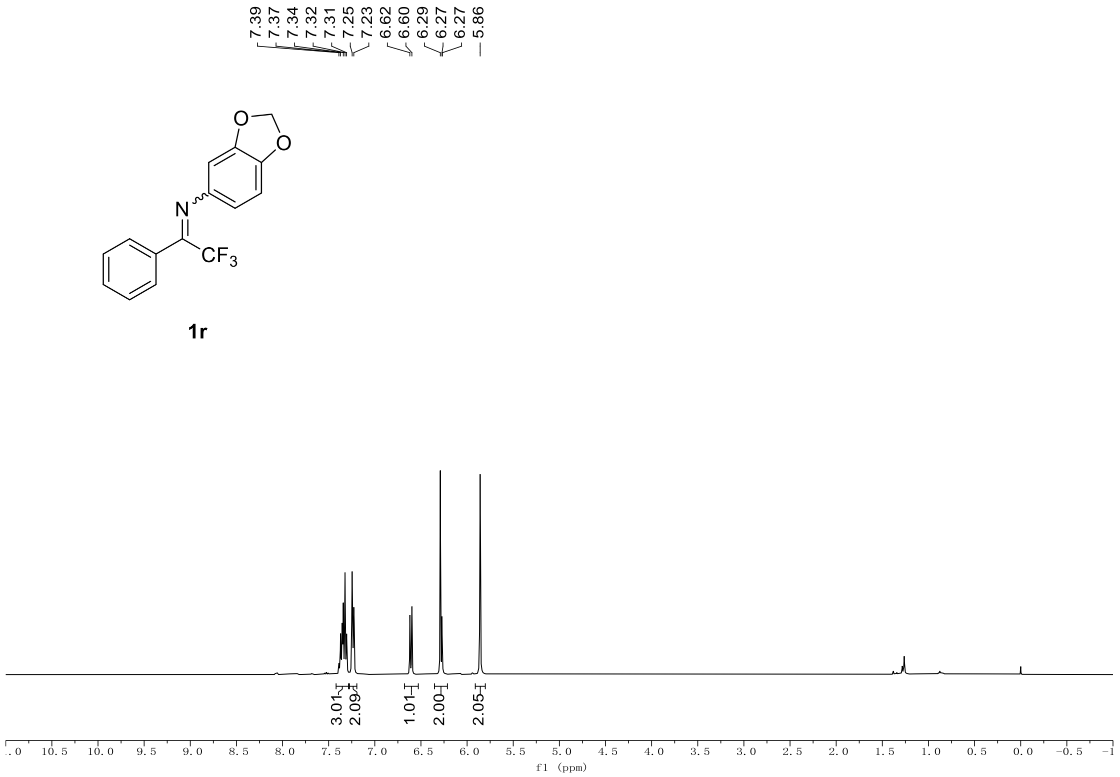

Figure S52. ^{1}**H NMR (400 MHz,** $CDCl_3$**) spectrum of compound 1r, related to Scheme 2**

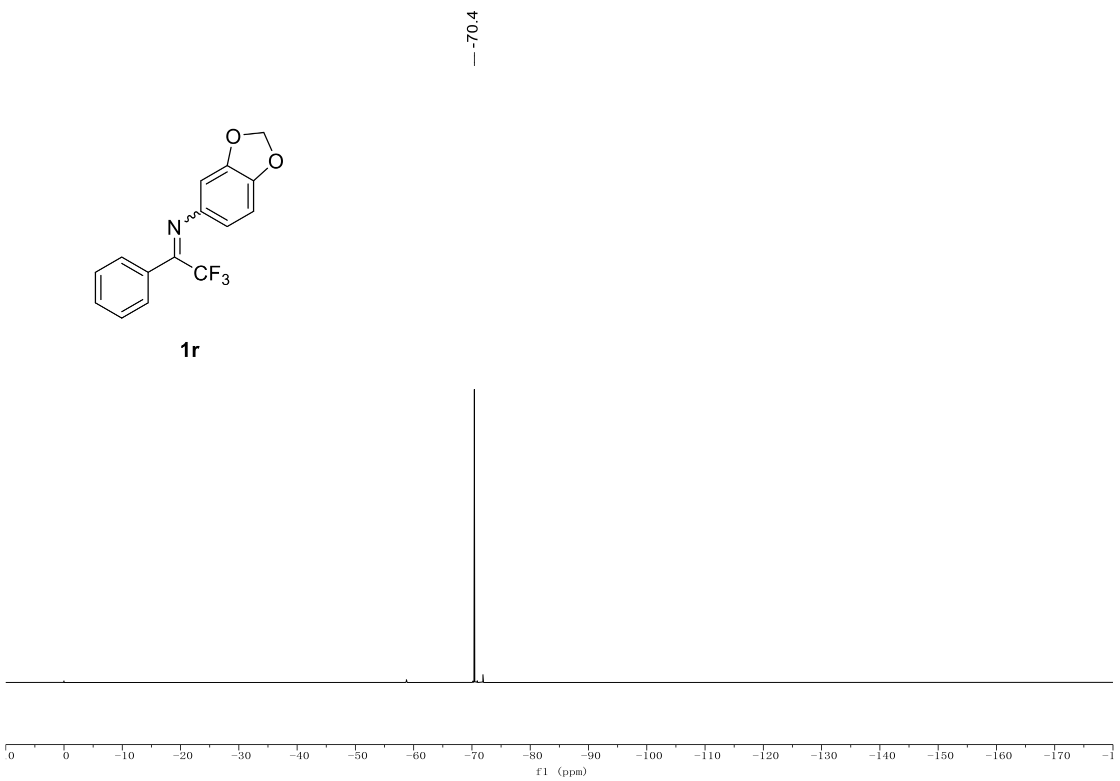

Figure S53. ^{19}**F NMR (376 MHz, CDCl$_3$) spectrum of compound 1r, related to Scheme 2**

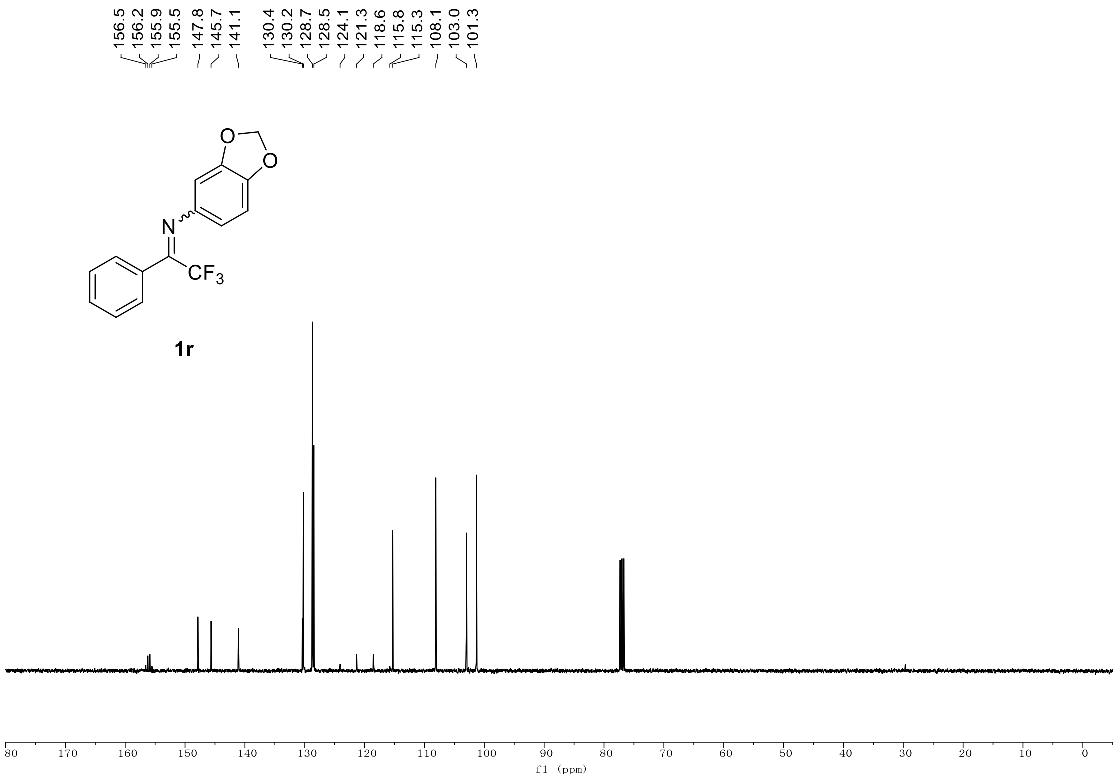

Figure S54. ^{13}**C NMR (101 MHz, CDCl$_3$) spectrum of compound 1r, related to Scheme 2**

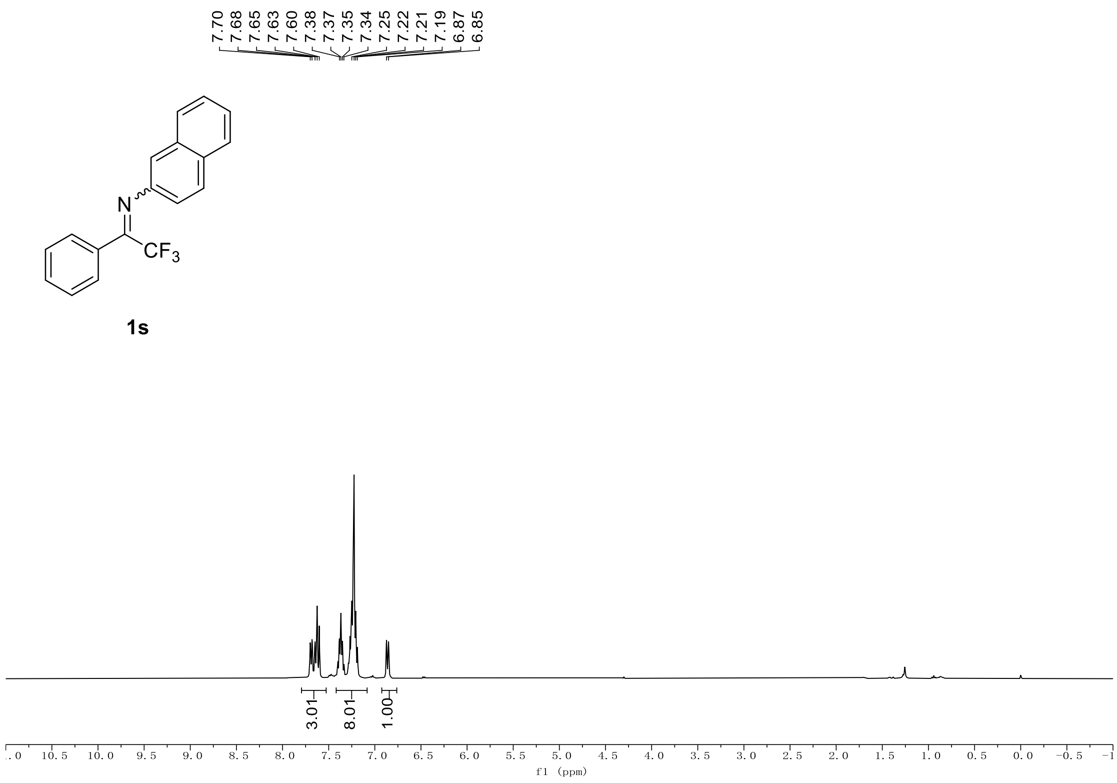

Figure S55. ^{1}H NMR (400 MHz, $CDCl_3$) spectrum of compound 1s, related to Scheme 2

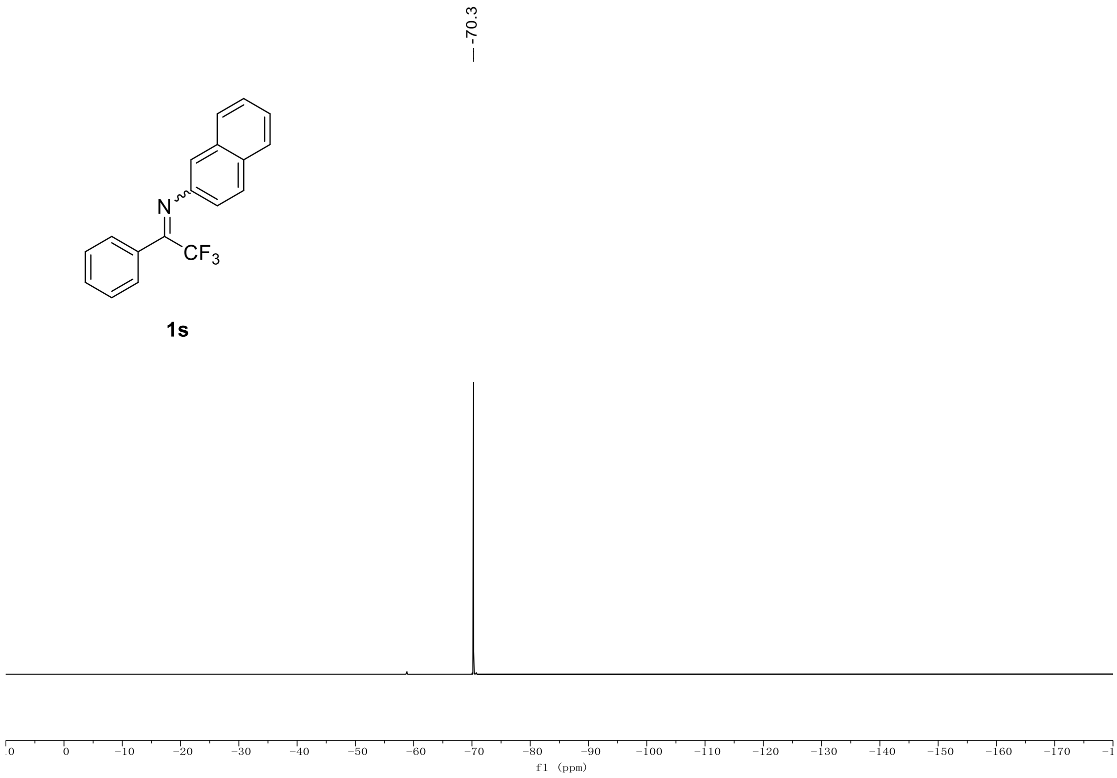

Figure S56. ^{19}F NMR (376 MHz, $CDCl_3$) spectrum of compound 1s, related to Scheme 2

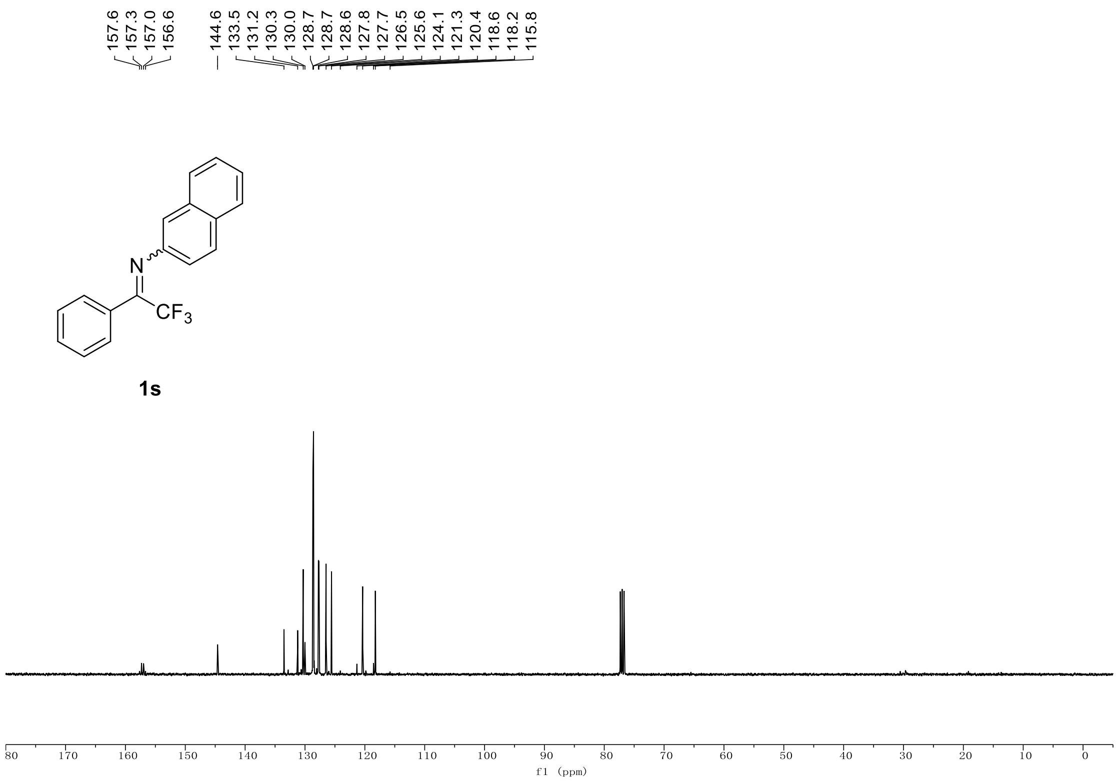

Figure S57. ^{13}C NMR (101 MHz, $CDCl_3$) spectrum of compound 1s, related to Scheme 2

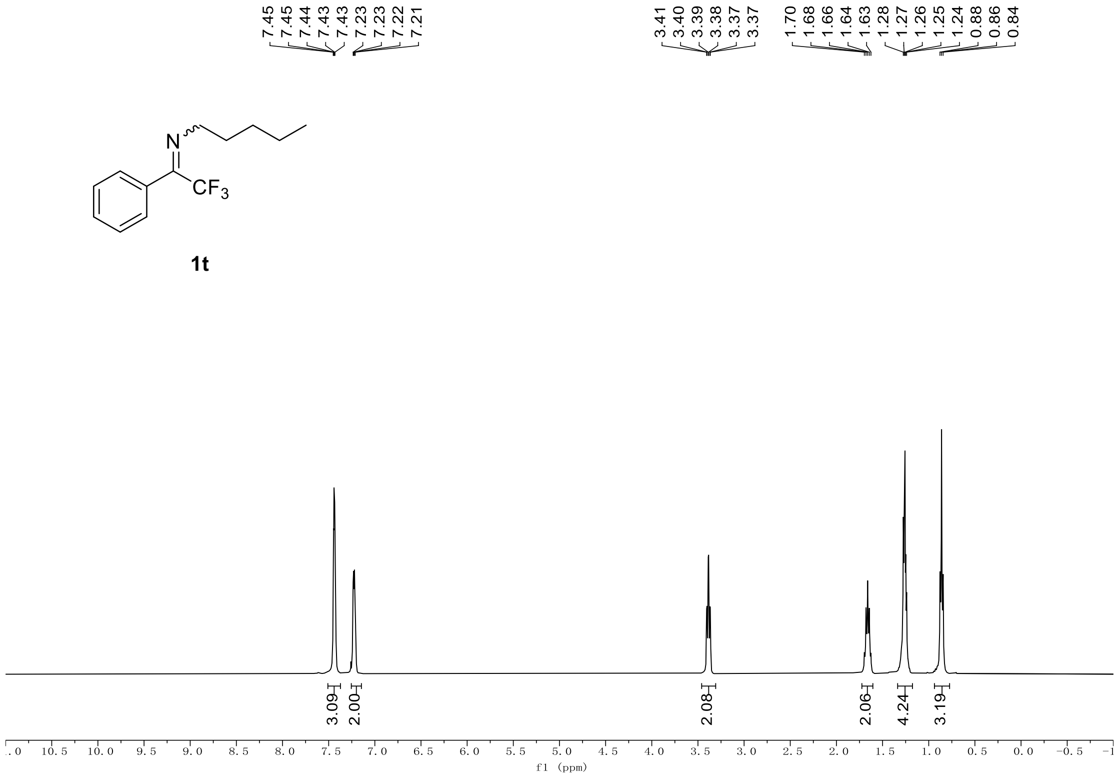

Figure S58. ^{1}H NMR (400 MHz, $CDCl_3$) spectrum of compound 1t, related to Scheme 2

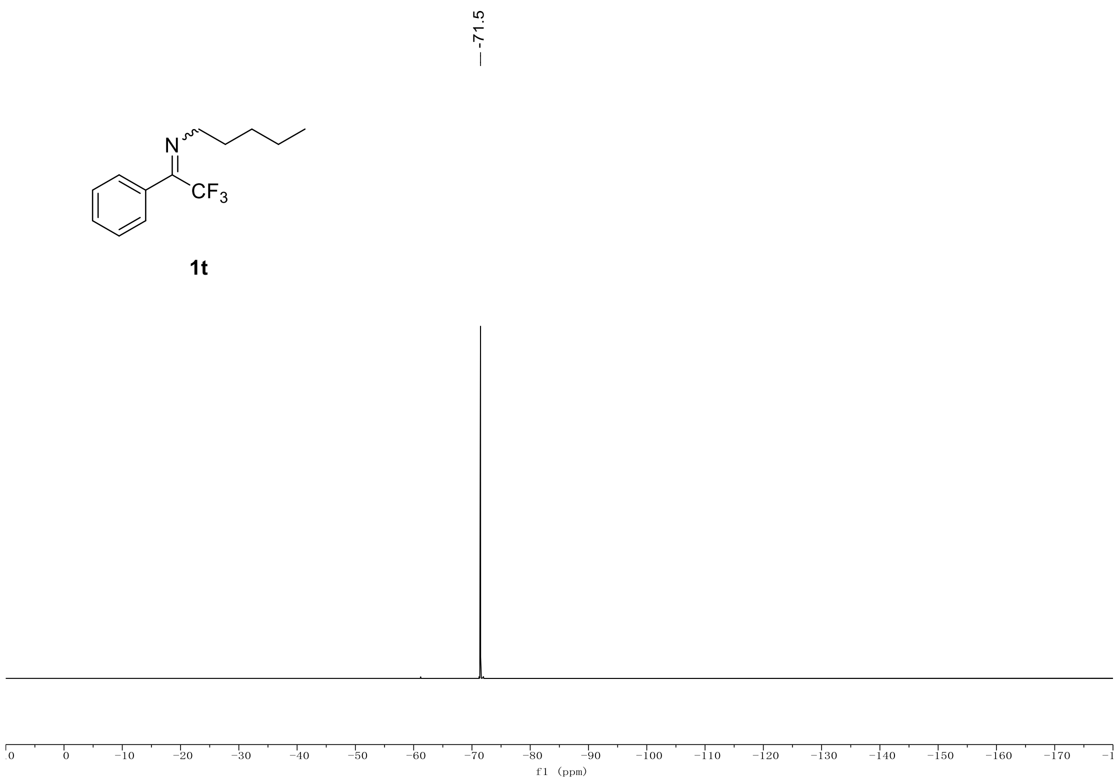

Figure S59. ^{19}F **NMR (376 MHz, CDCl$_3$) spectrum of compound 1t, related to Scheme 2**

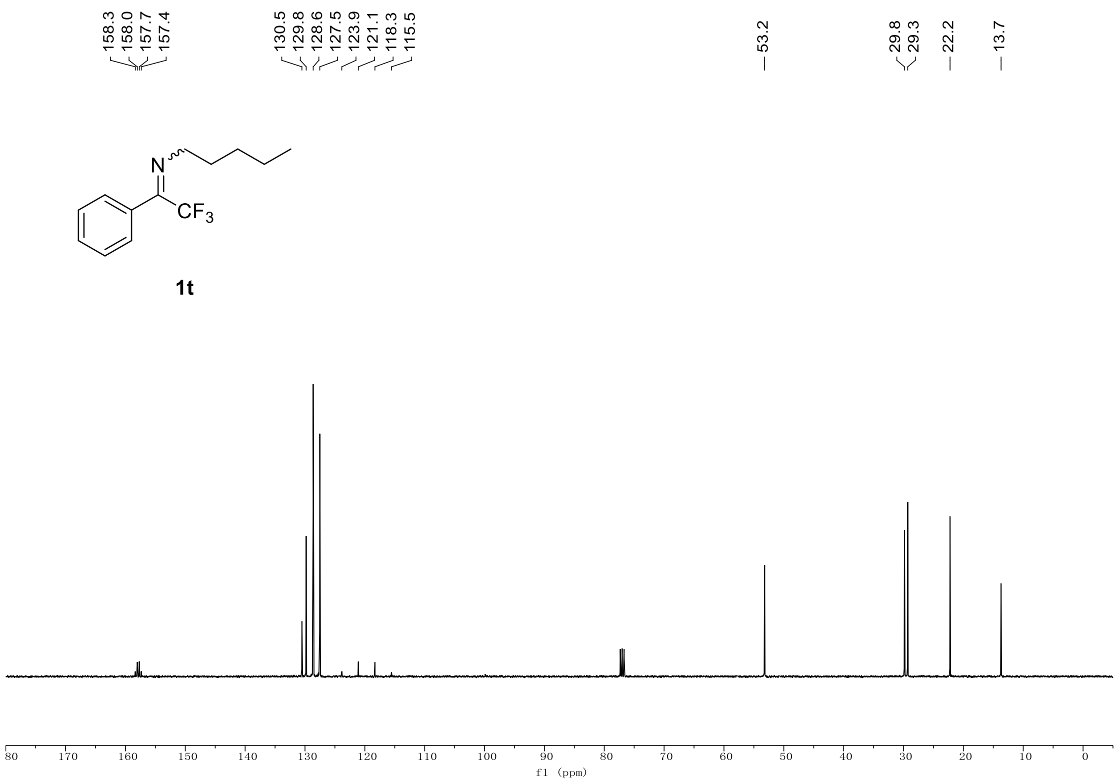

Figure S60. ^{13}C **NMR (101 MHz, CDCl$_3$) spectrum of compound 1t, related to Scheme 2**

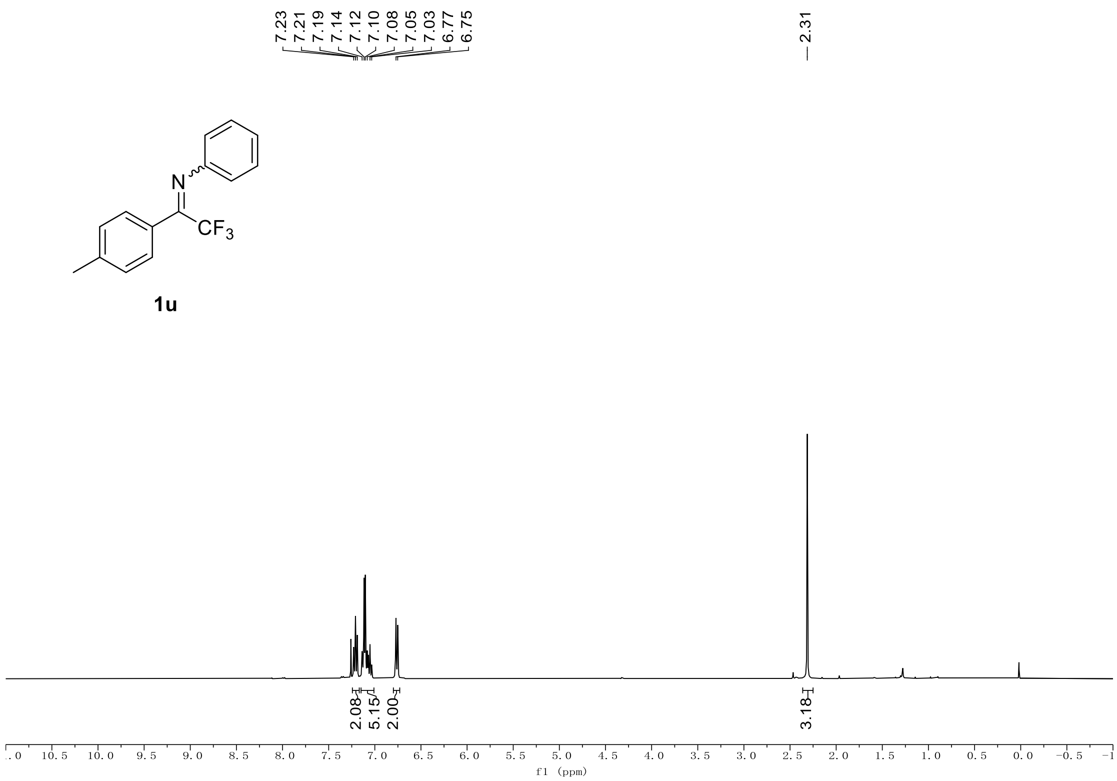

Figure S61. ^{1}H NMR (400 MHz, CDCl$_3$) spectrum of compound 1u, related to Scheme 2

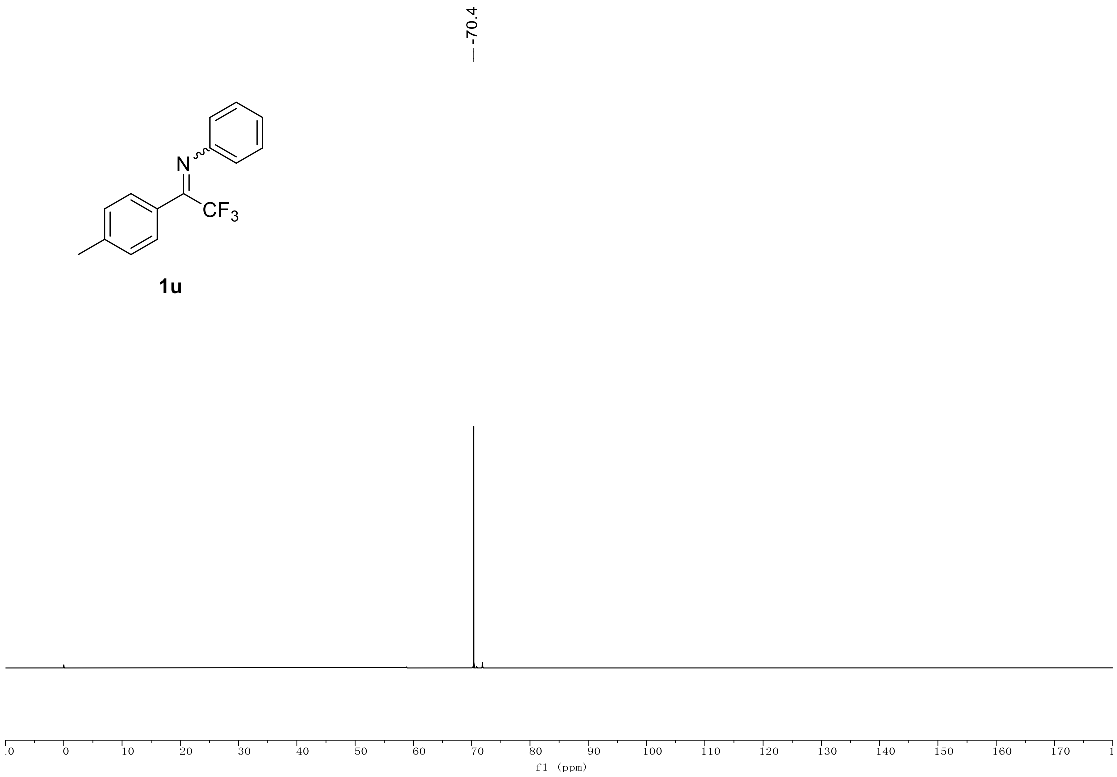

Figure S62. ^{19}F NMR (376 MHz, CDCl$_3$) spectrum of compound 1u, related to Scheme 2

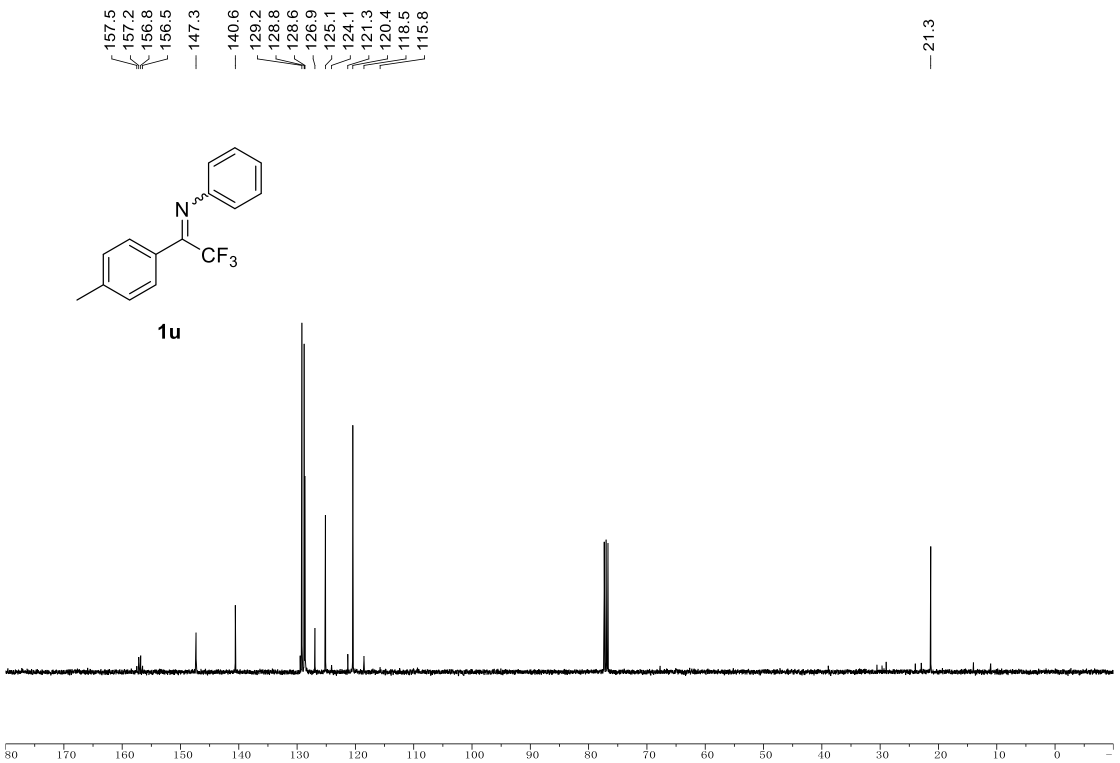

Figure S63. ^{13}C NMR (101 MHz, $CDCl_3$) spectrum of compound 1u, related to Scheme 2

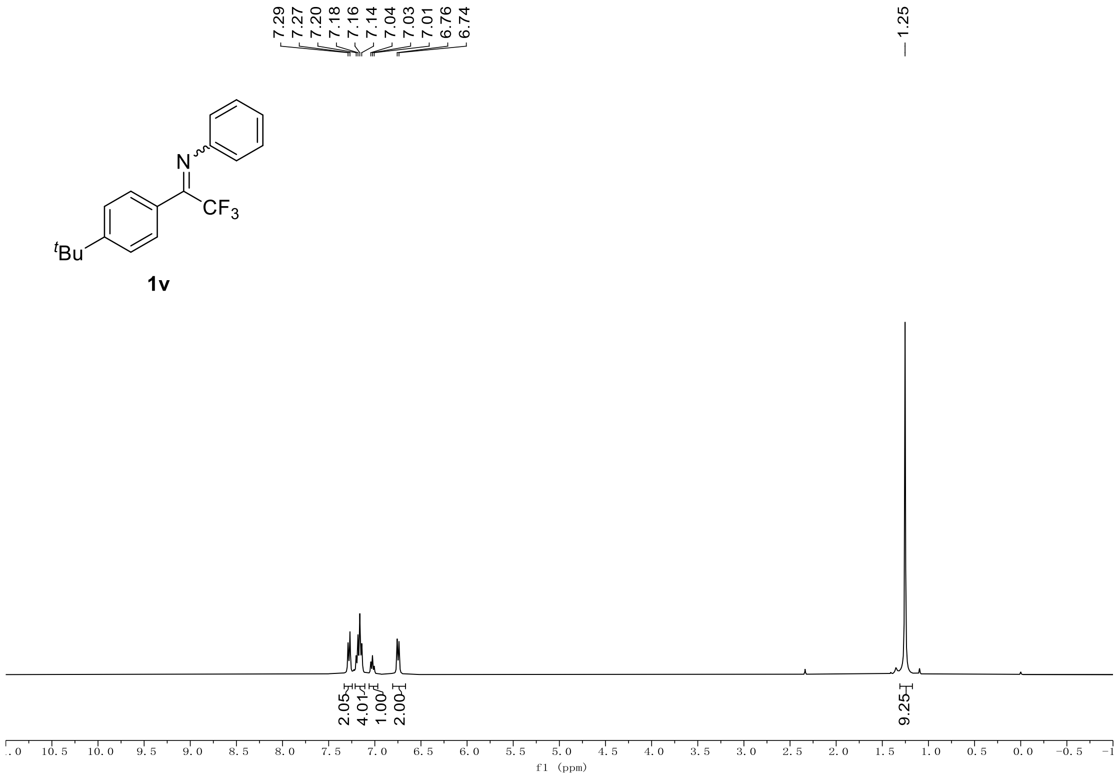

Figure S64. ^{1}H NMR (400 MHz, $CDCl_3$) spectrum of compound 1v, related to Scheme 2

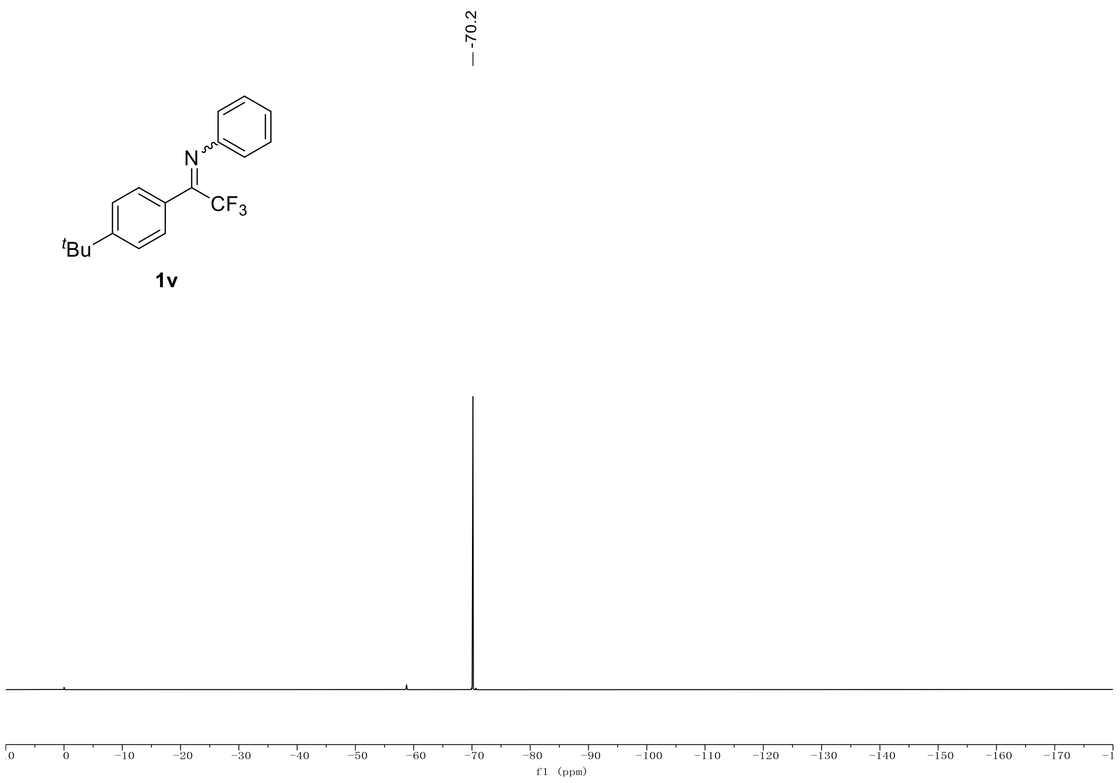

Figure S65. ^{19}F NMR (376 MHz, $CDCl_3$) spectrum of compound 1v, related to Scheme 2

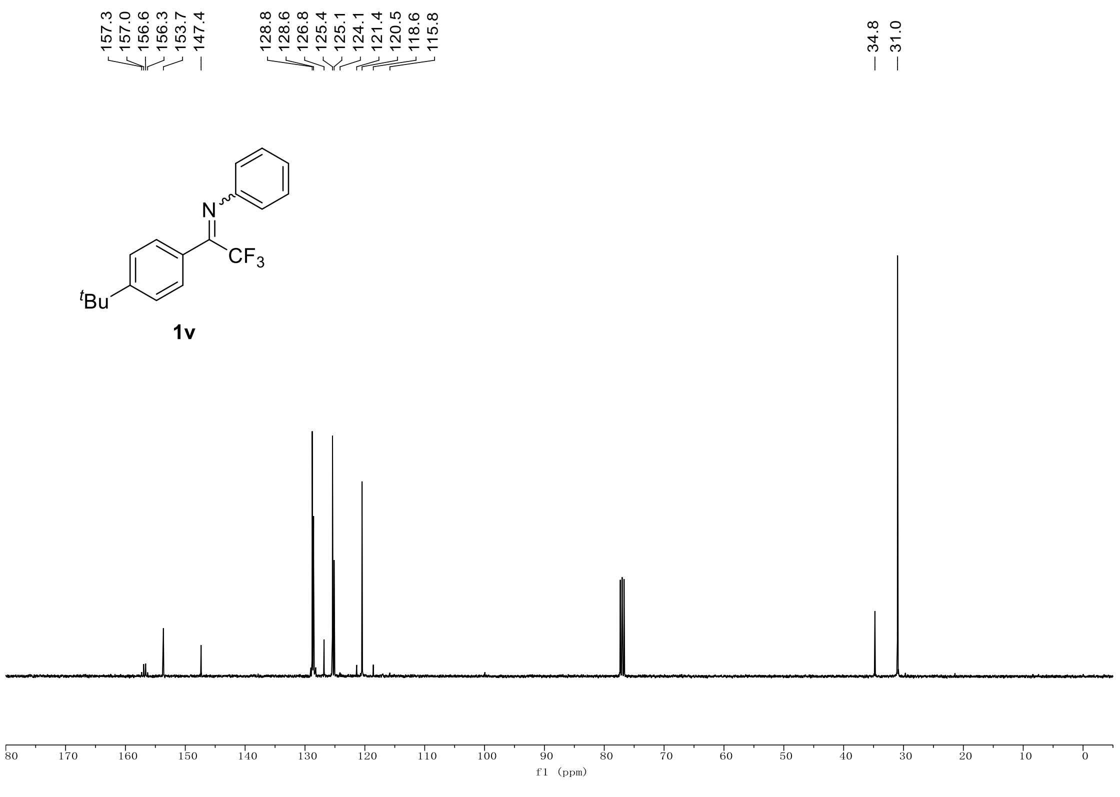

Figure S66. ^{13}C NMR (101 MHz, $CDCl_3$) spectrum of compound 1v, related to Scheme 2

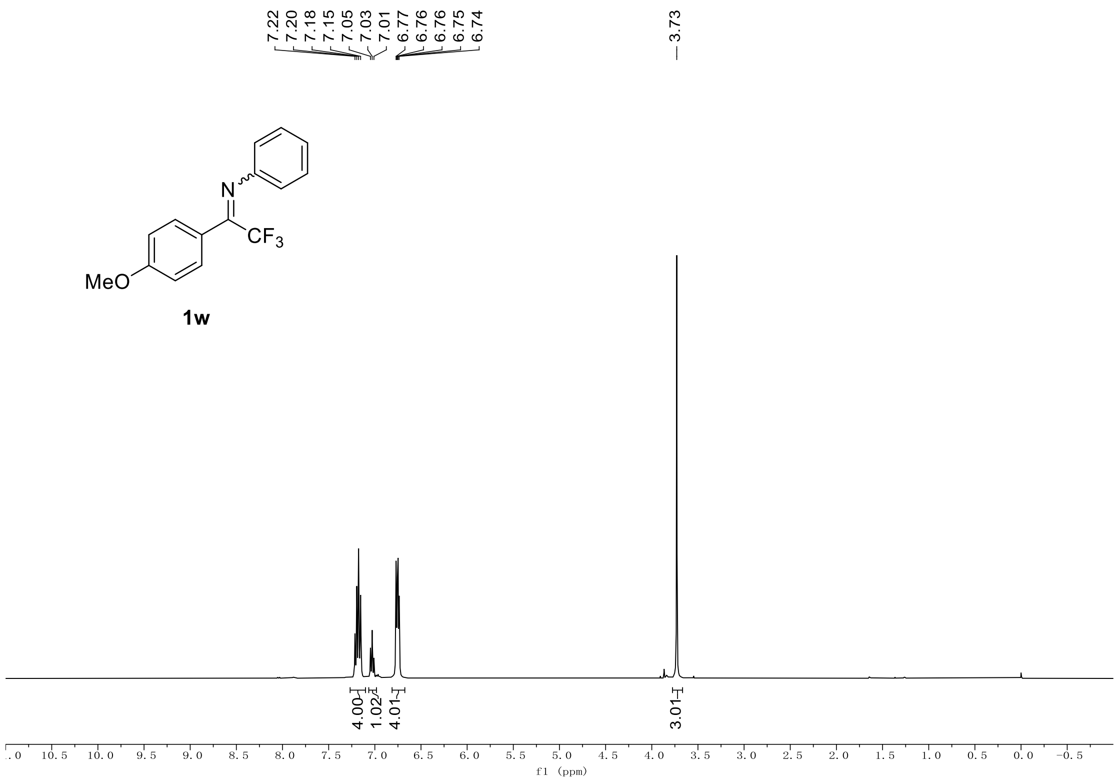

Figure S67. ^{1}H NMR (400 MHz, $CDCl_3$) spectrum of compound 1w, related to Scheme 2

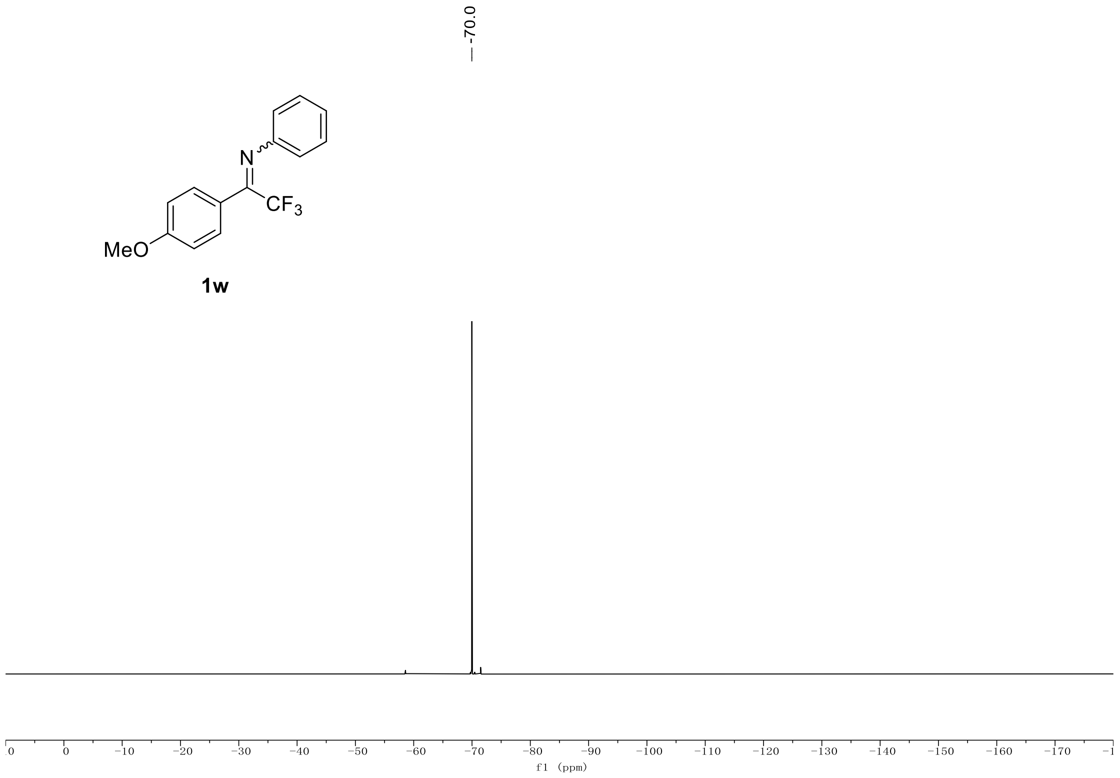

Figure S68. ^{19}F NMR (376 MHz, $CDCl_3$) spectrum of compound 1w, related to Scheme 2

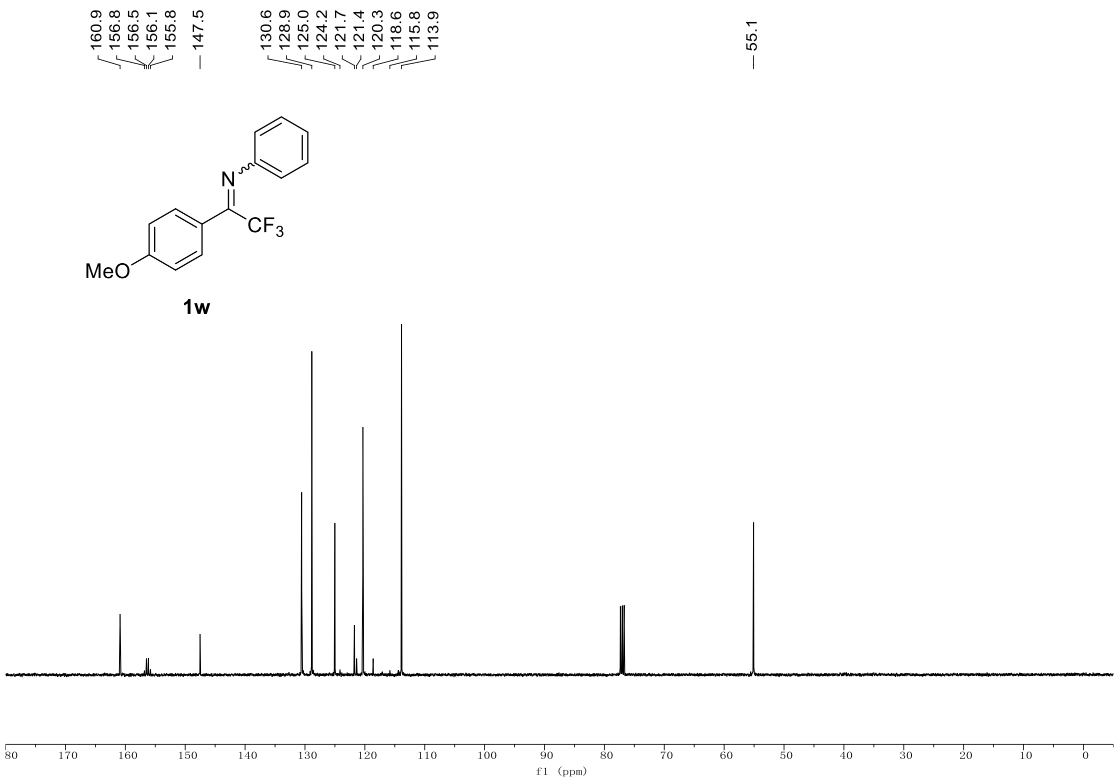

Figure S69. ^{13}C NMR (101 MHz, $CDCl_3$) spectrum of compound 1w, related to Scheme 2

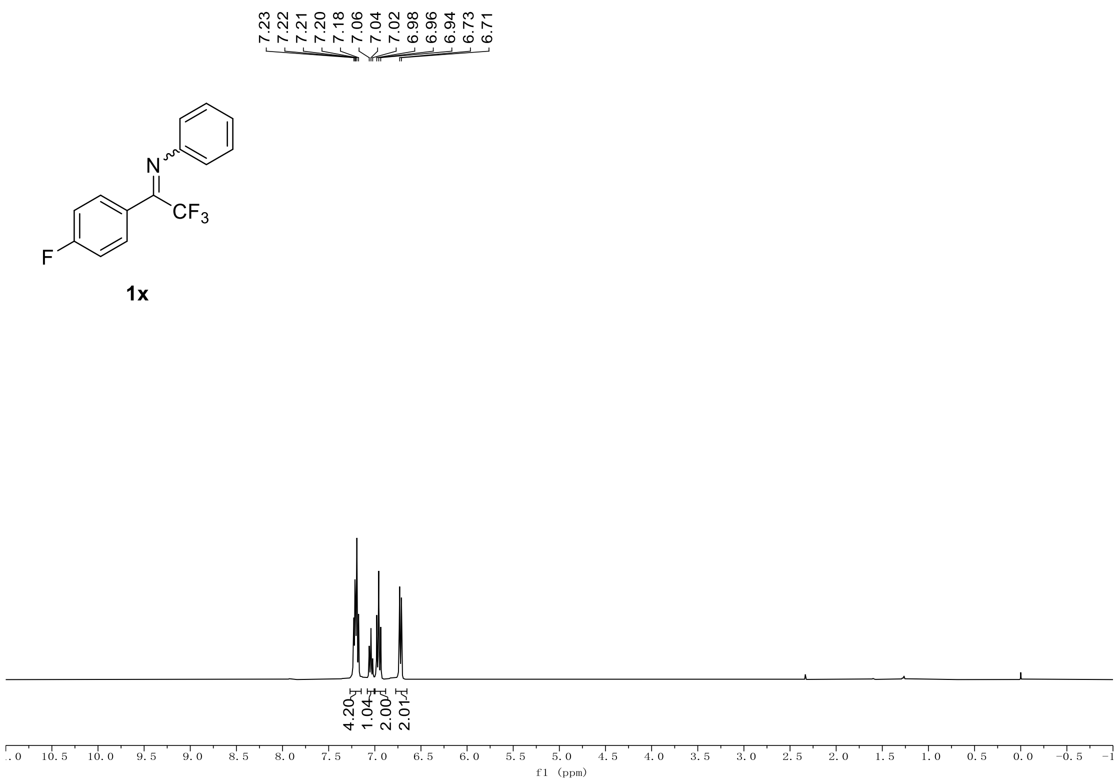

Figure S70. ^{1}H NMR (400 MHz, $CDCl_3$) spectrum of compound 1x, related to Scheme 2

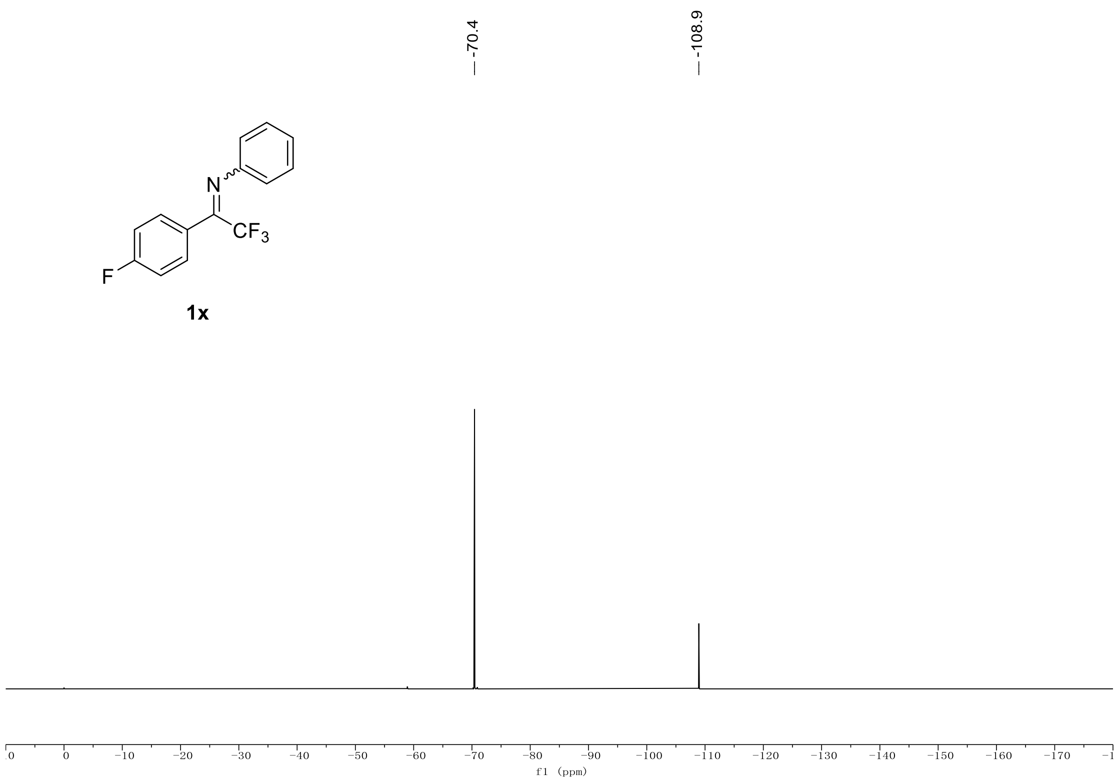

Figure S71. ^{19}F NMR (376 MHz, CDCl$_3$) spectrum of compound 1x, related to Scheme 2

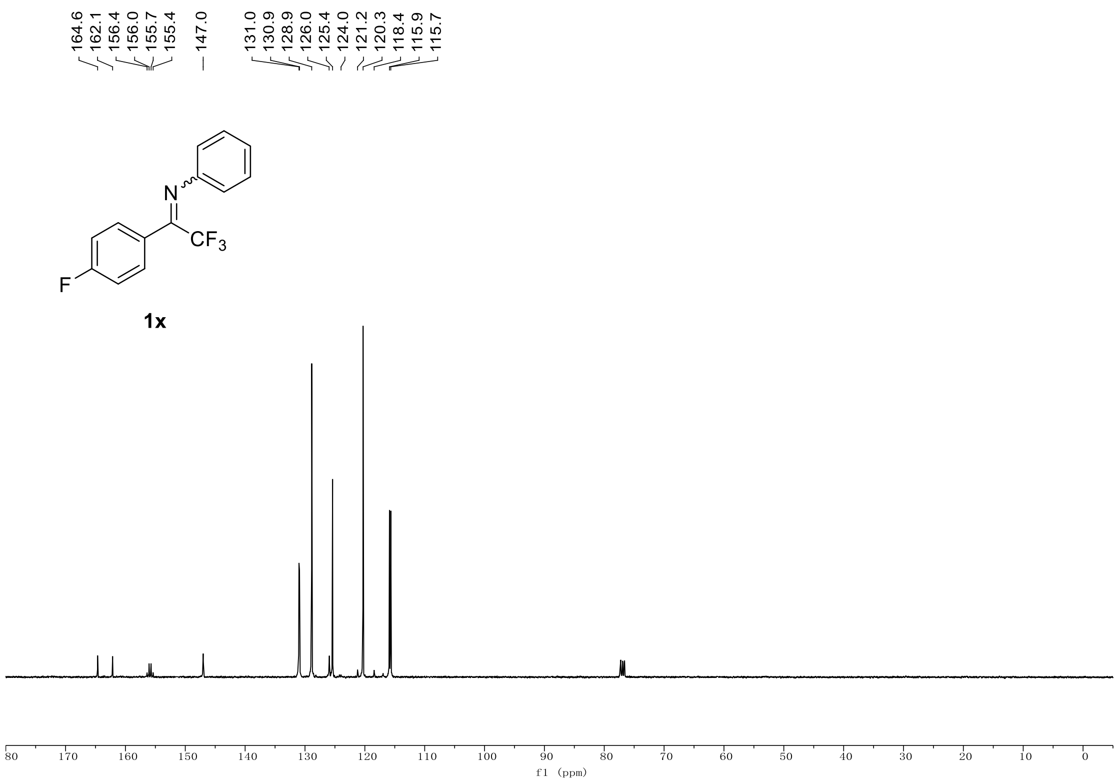

Figure S72. ^{13}C NMR (101 MHz, CDCl$_3$) spectrum of compound 1x, related to Scheme 2

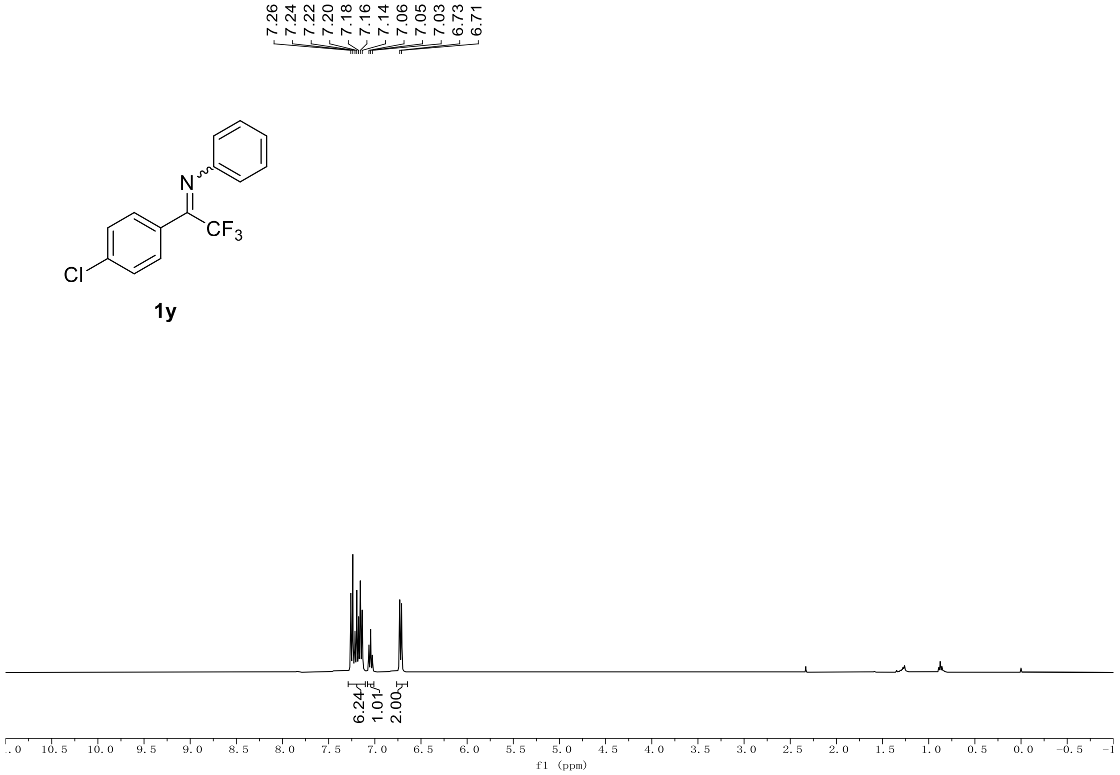

Figure S73. ^{1}H NMR (400 MHz, $CDCl_3$) spectrum of compound 1y, related to Scheme 2

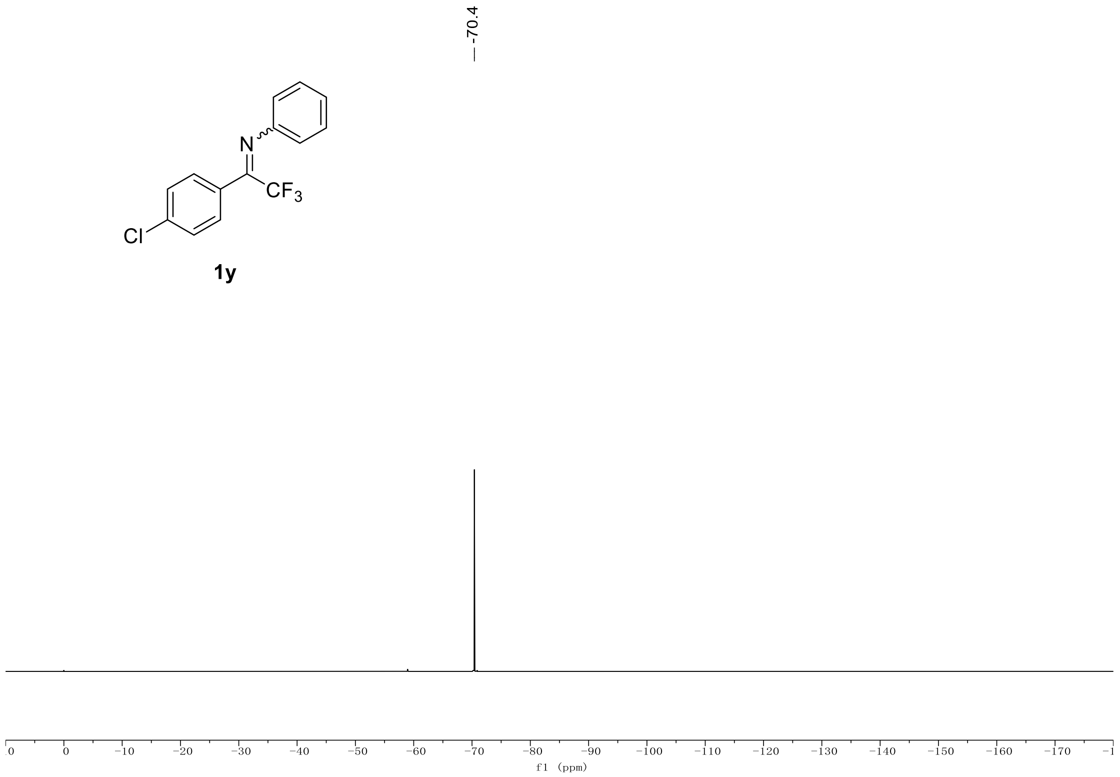

Figure S74. ^{19}F NMR (376 MHz, $CDCl_3$) spectrum of compound 1y, related to Scheme 2

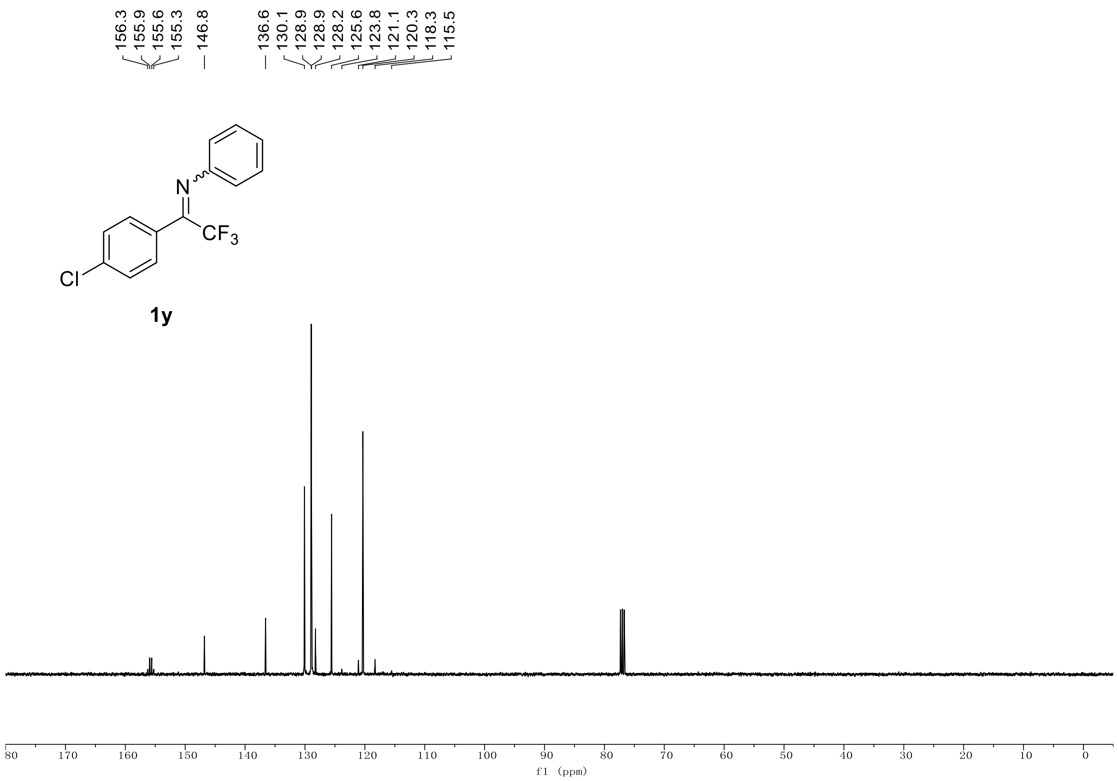

Figure S75. ^{13}C **NMR (101 MHz,** $CDCl_3$**) spectrum of compound 1y, related to Scheme 2**

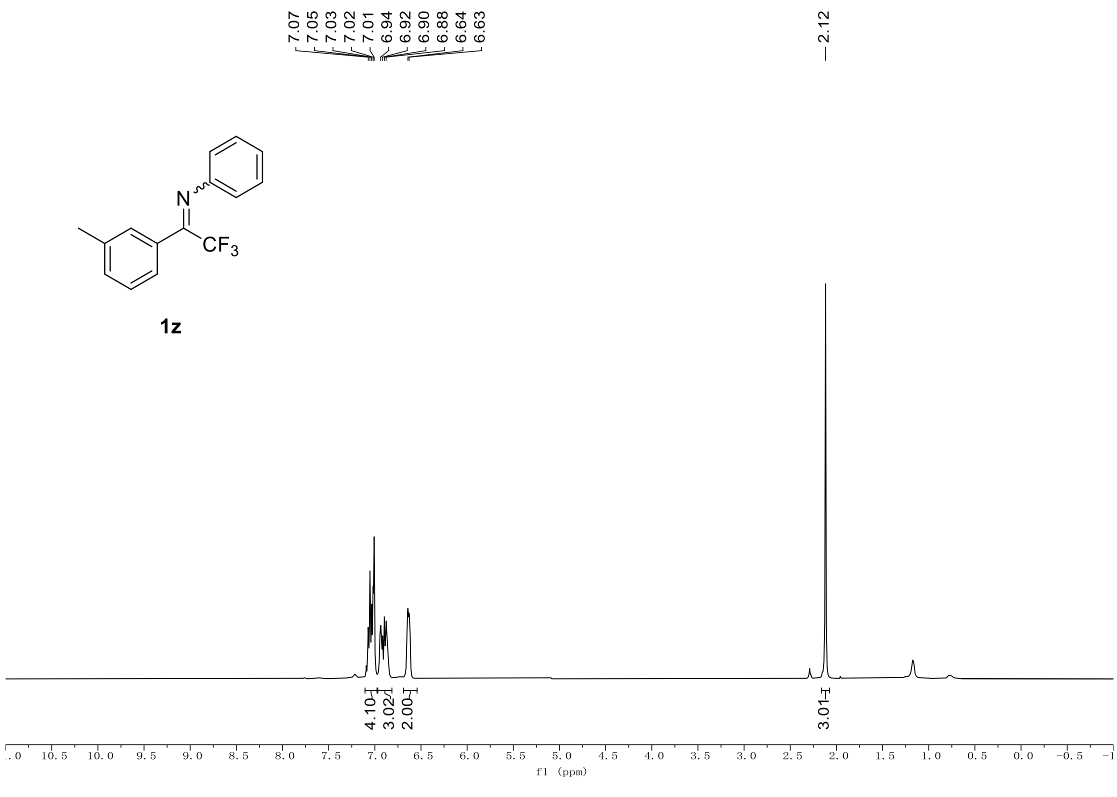

Figure S76. ^{1}H **NMR (400 MHz,** $CDCl_3$**) spectrum of compound 1z, related to Scheme 2**

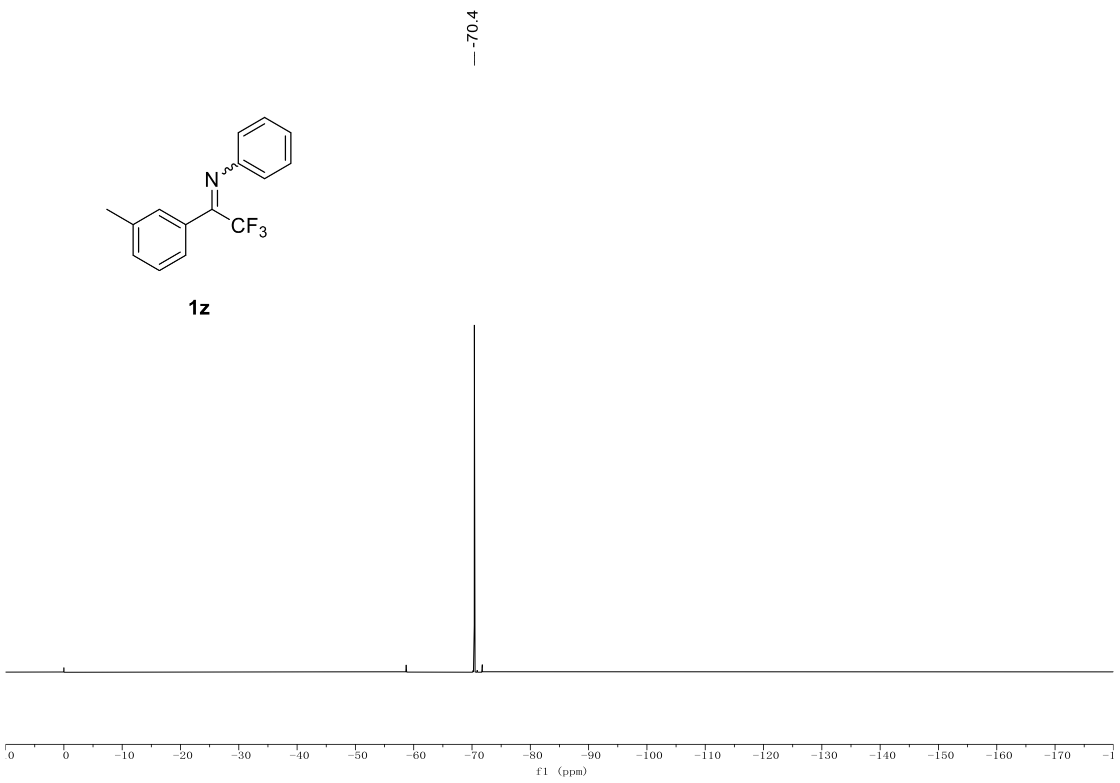

Figure S77. ^{19}**F NMR (376 MHz, CDCl$_3$) spectrum of compound 1z, related to Scheme 2**

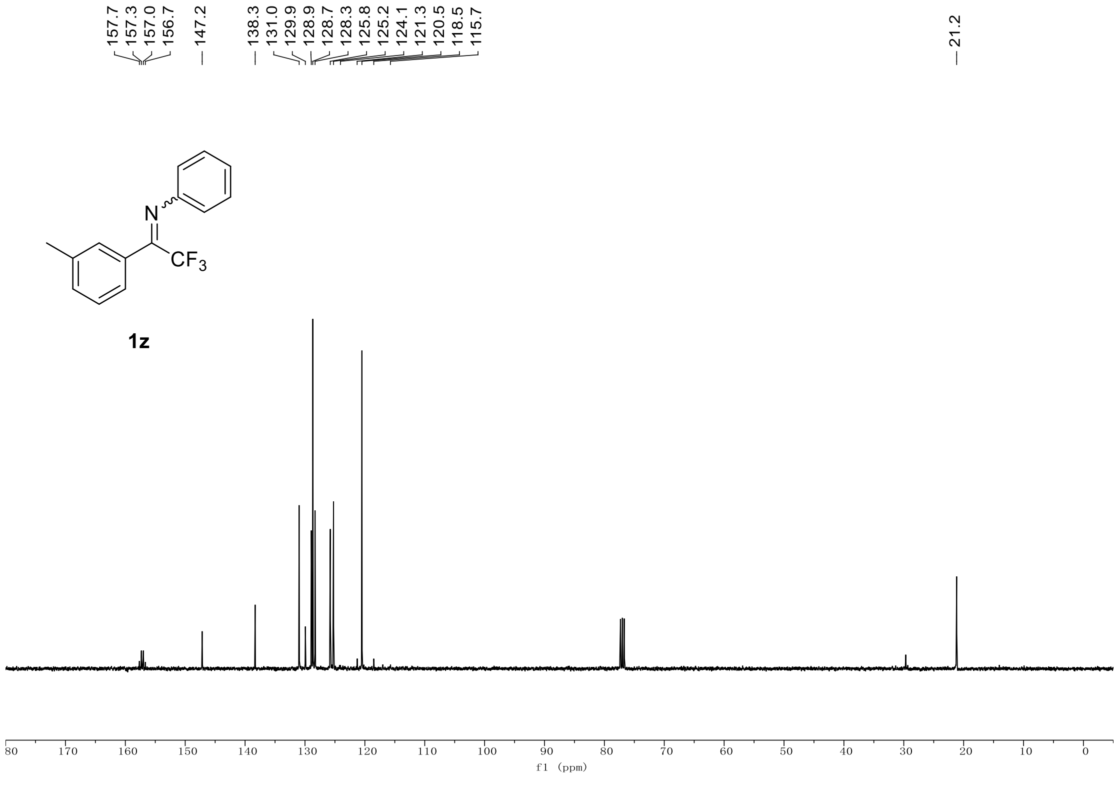

Figure S78. ^{13}**C NMR (101 MHz, CDCl$_3$) spectrum of compound 1z, related to Scheme 2**

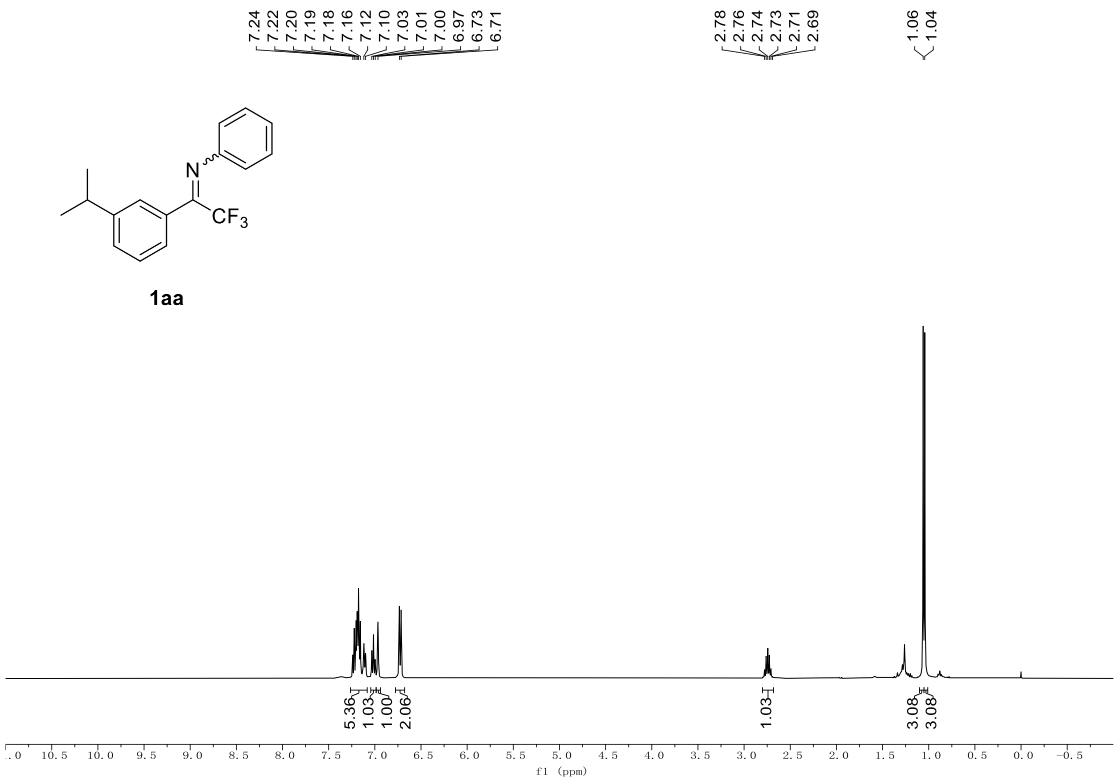

Figure S79. ^{1}**H NMR (400 MHz, CDCl$_3$) spectrum of compound 1aa, related to Scheme 2**

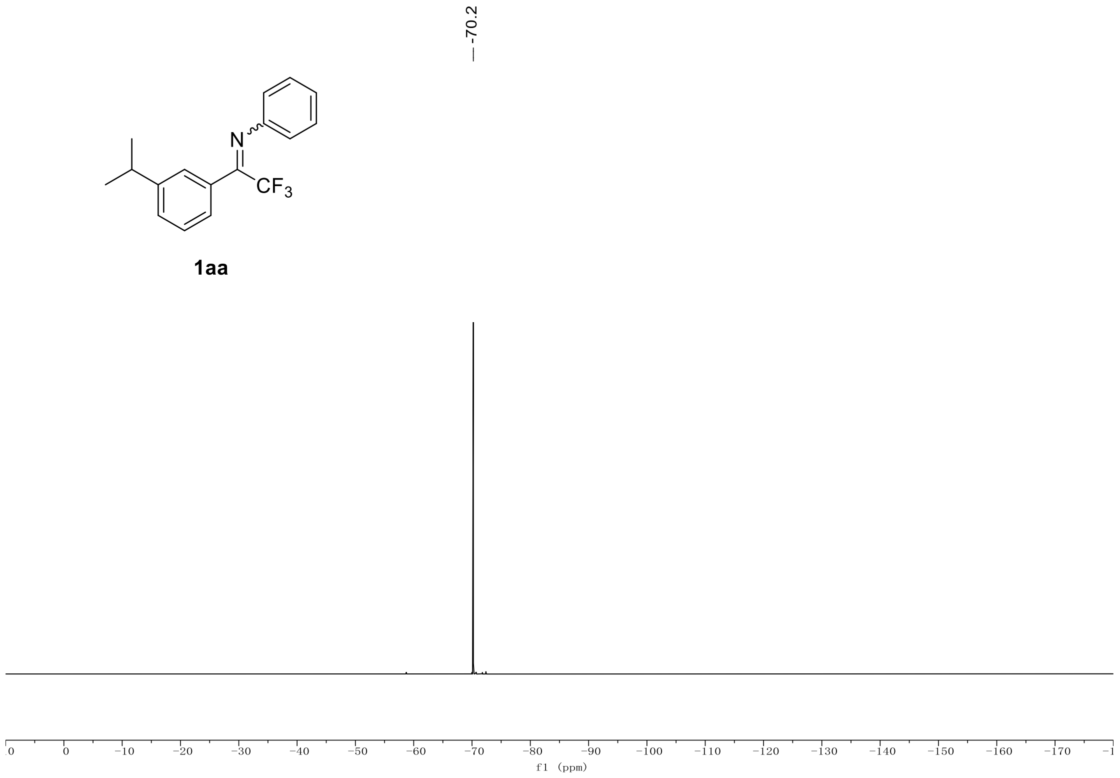

Figure S80. ^{19}**F NMR (376 MHz, CDCl$_3$) spectrum of compound 1aa, related to Scheme 2**

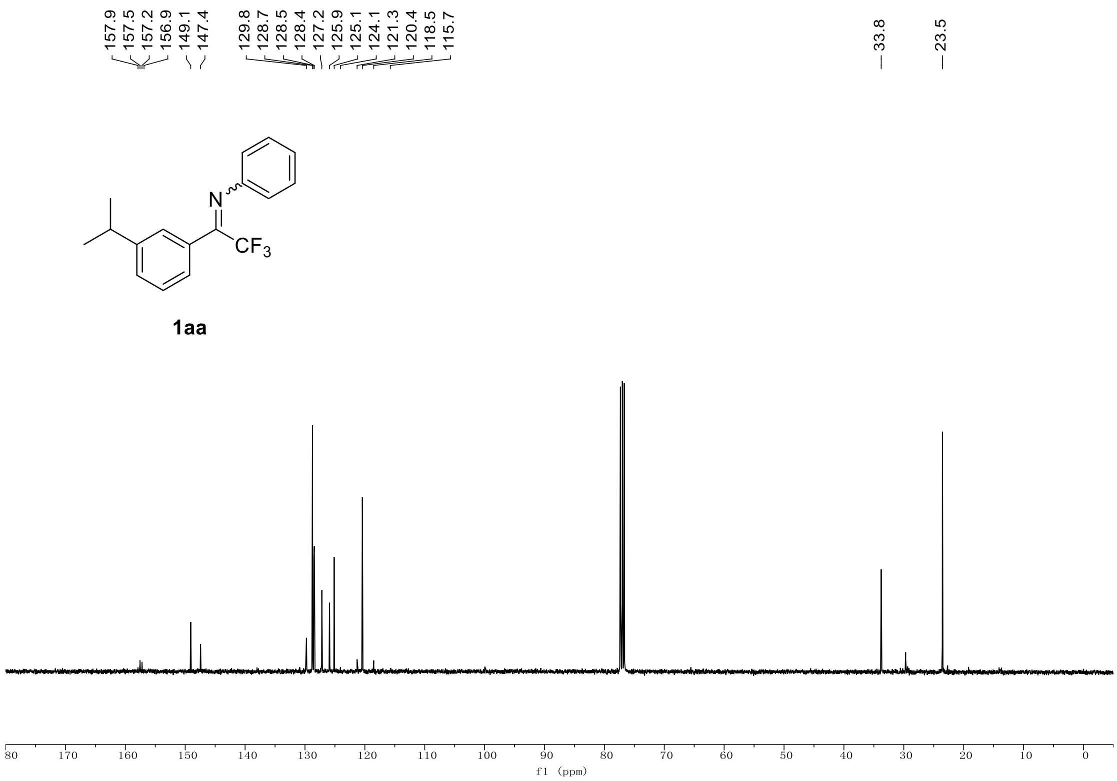

Figure S81. ^{13}C NMR (101 MHz, $CDCl_3$) spectrum of compound 1aa, related to Scheme 2

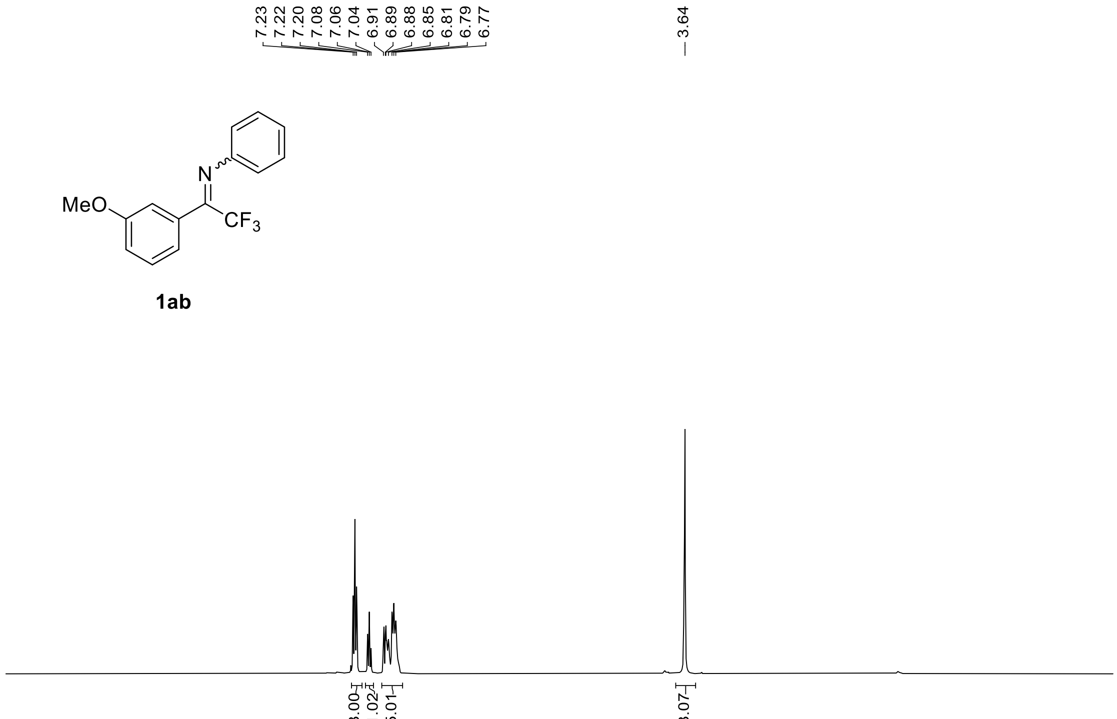

Figure S82. ^{1}H NMR (400 MHz, $CDCl_3$) spectrum of compound 1ab, related to Scheme 2

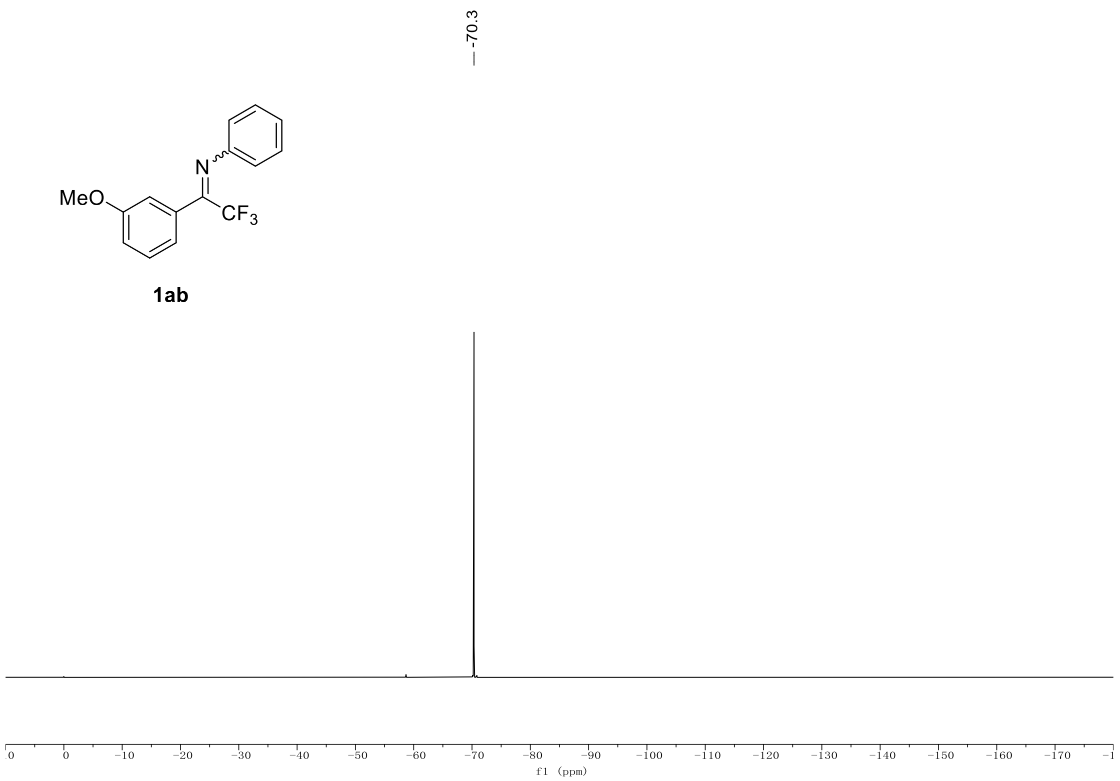

Figure S83. **^{19}F NMR (376 MHz, $CDCl_3$) spectrum of compound 1ab, related to Scheme 2**

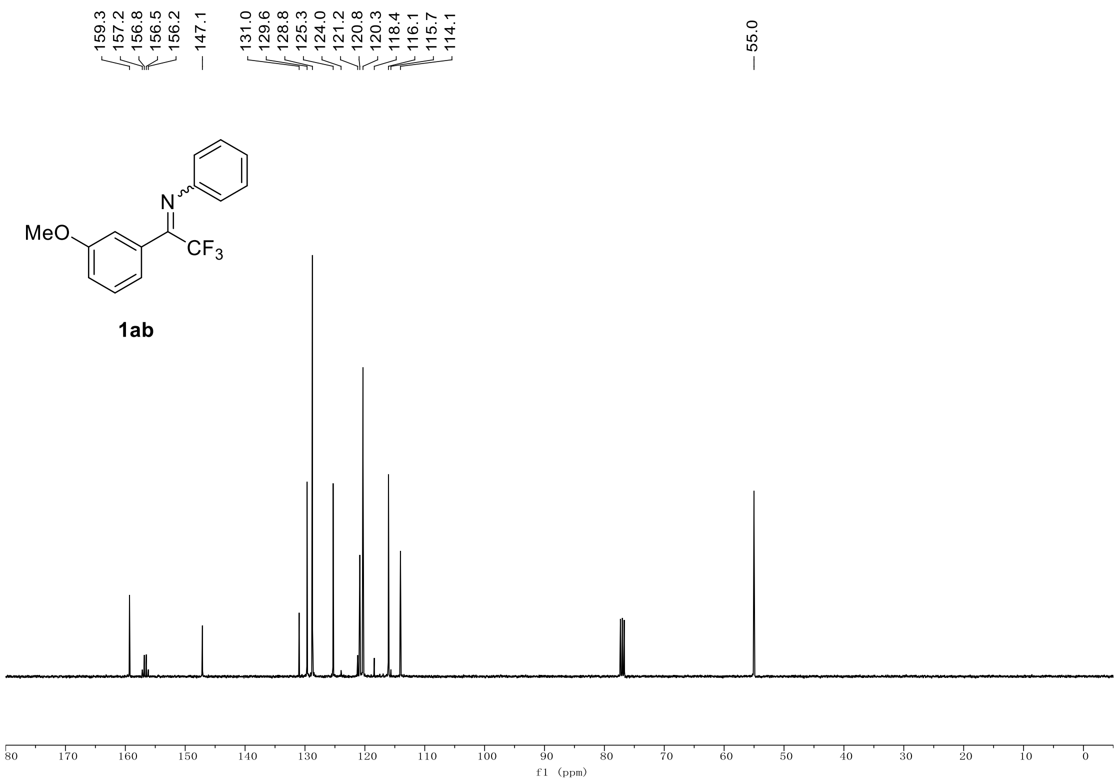

Figure S84. **^{13}C NMR (101 MHz, $CDCl_3$) spectrum of compound 1ab, related to Scheme 2**

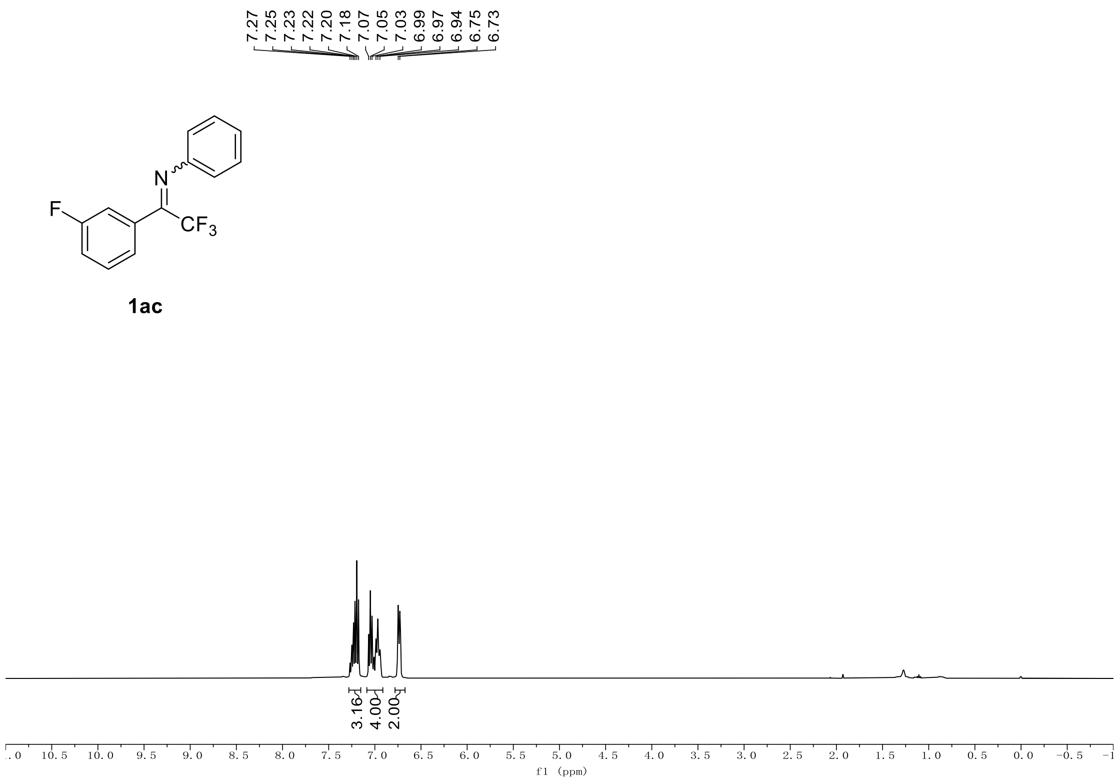

Figure S85. ^{1}H NMR (400 MHz, $CDCl_3$) spectrum of compound 1ac, related to Scheme 2

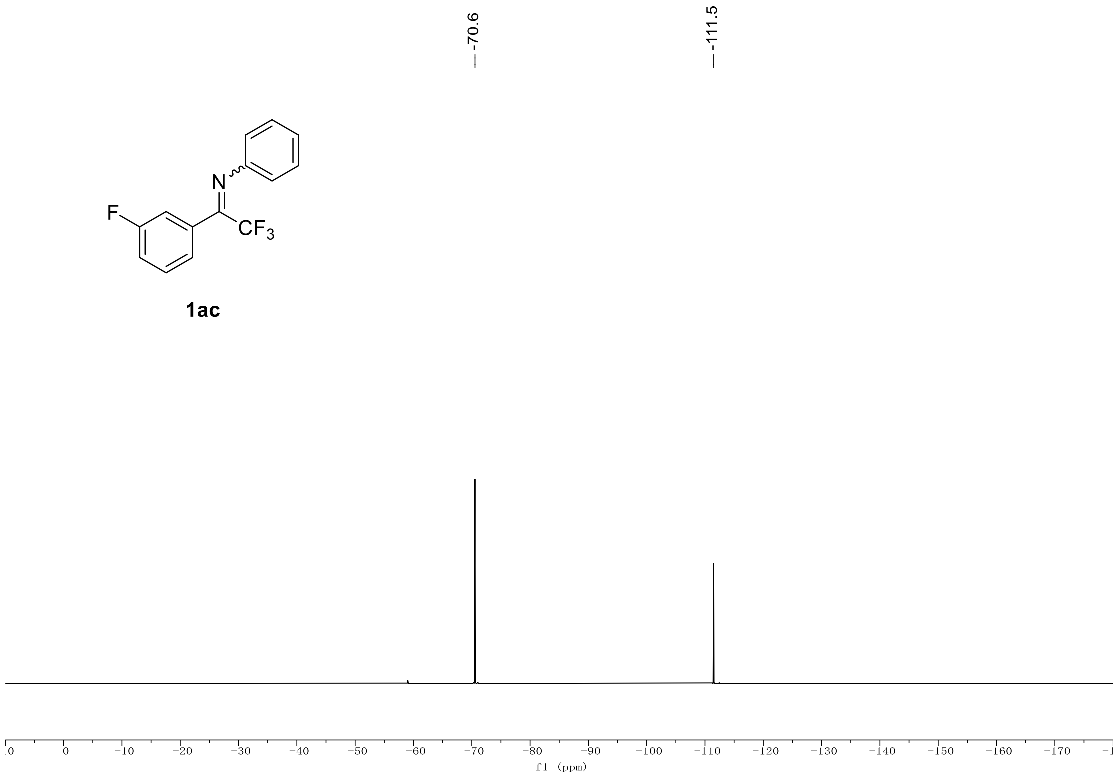

Figure S86. ^{19}F NMR (376 MHz, $CDCl_3$) spectrum of compound 1ac, related to Scheme 2

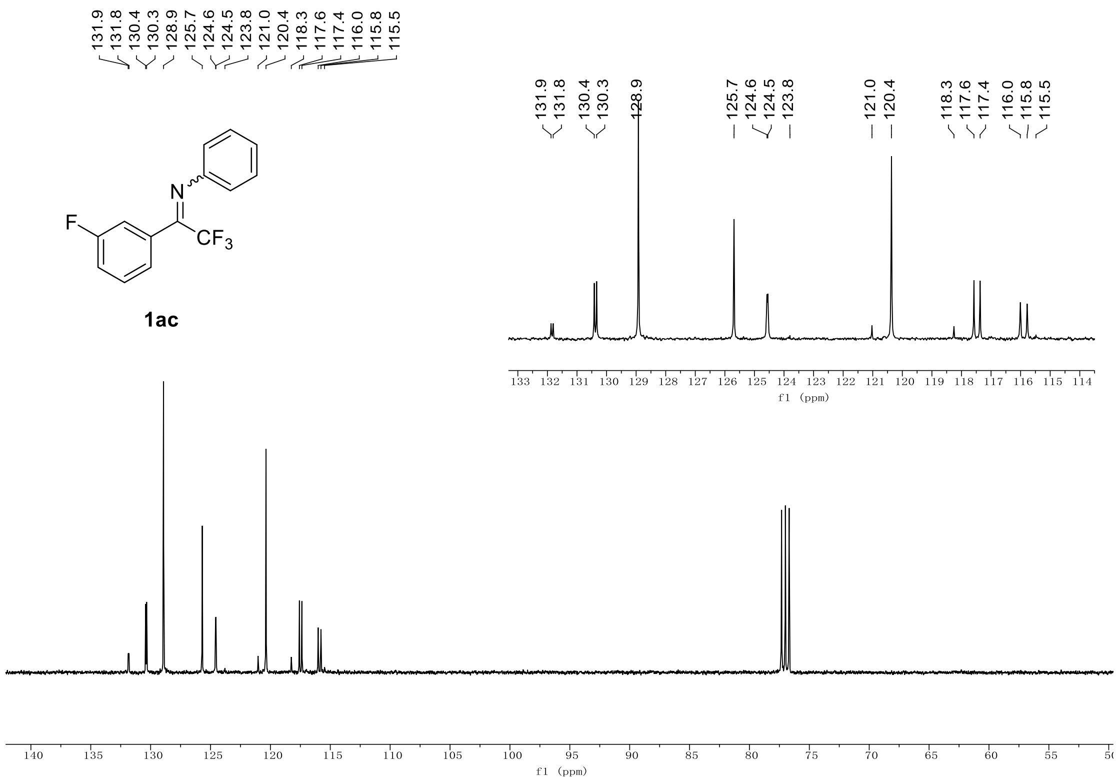

Figure S87. ^{13}C NMR (101 MHz, $CDCl_3$) spectrum of compound 1ac, related to Scheme 2

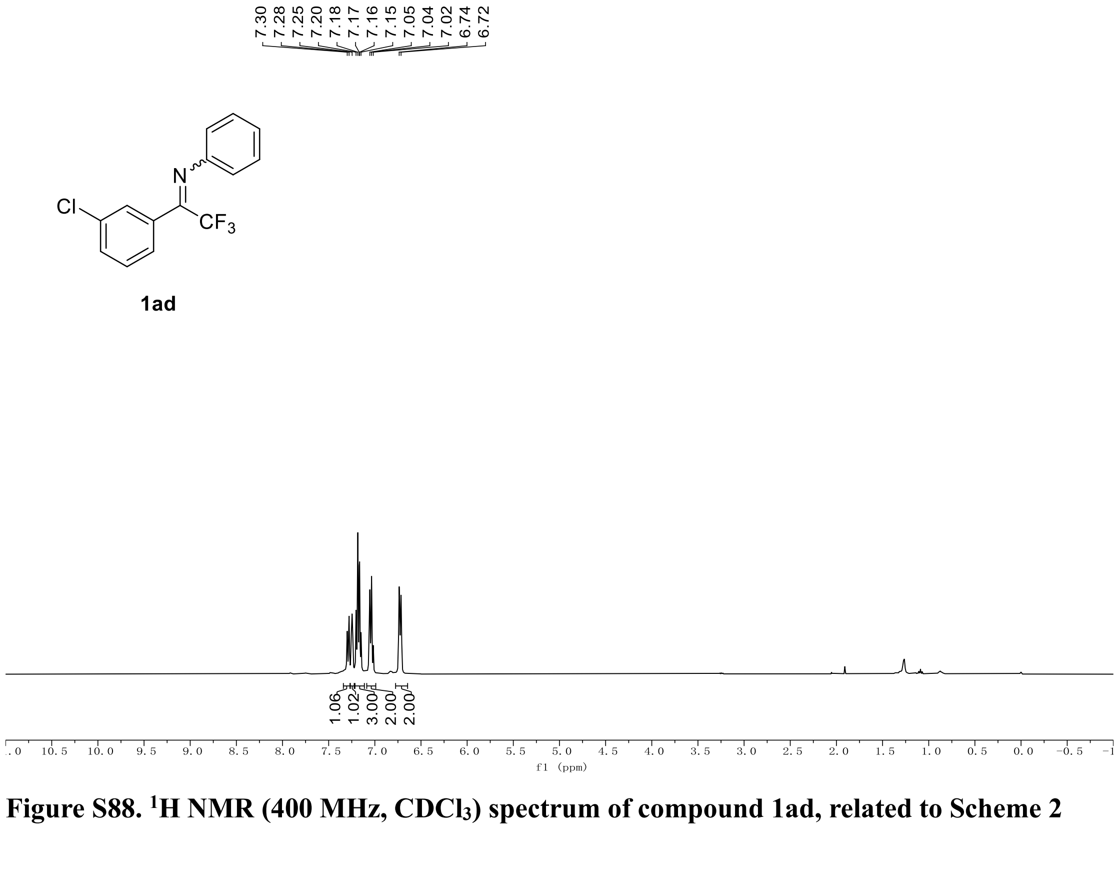

Figure S88. ^{1}H NMR (400 MHz, $CDCl_3$) spectrum of compound 1ad, related to Scheme 2

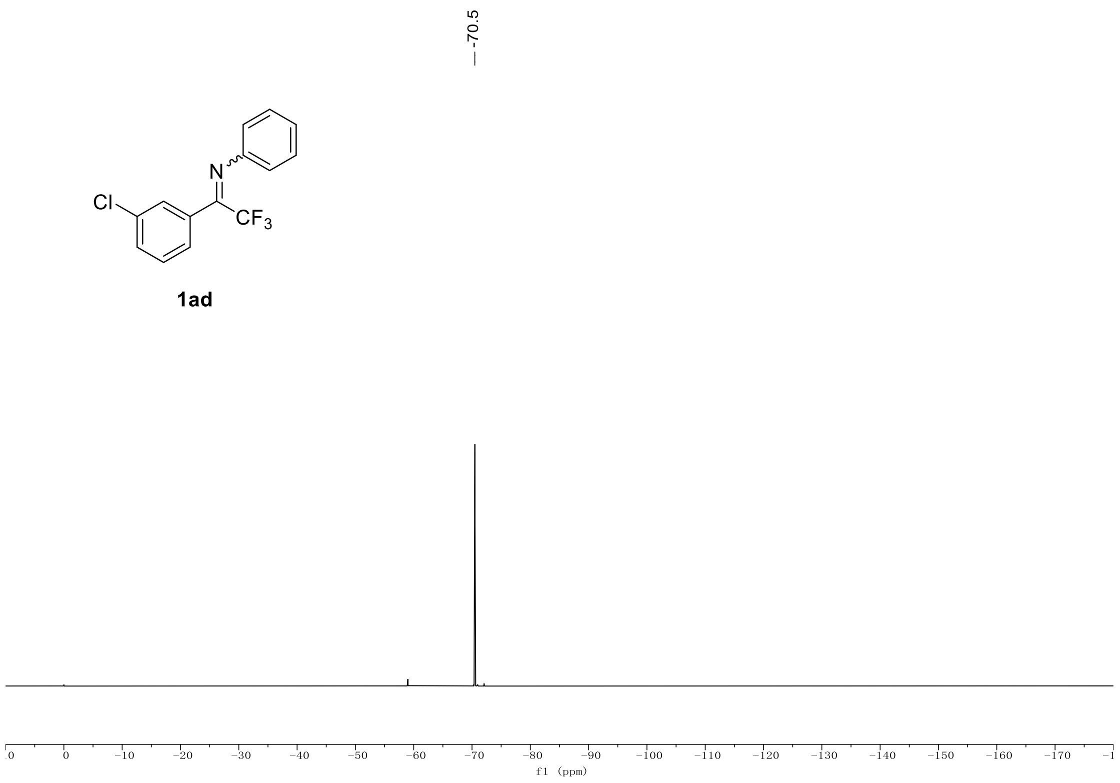

Figure S89. [19]**F NMR (376 MHz, CDCl₃) spectrum of compound 1ad, related to Scheme 2**

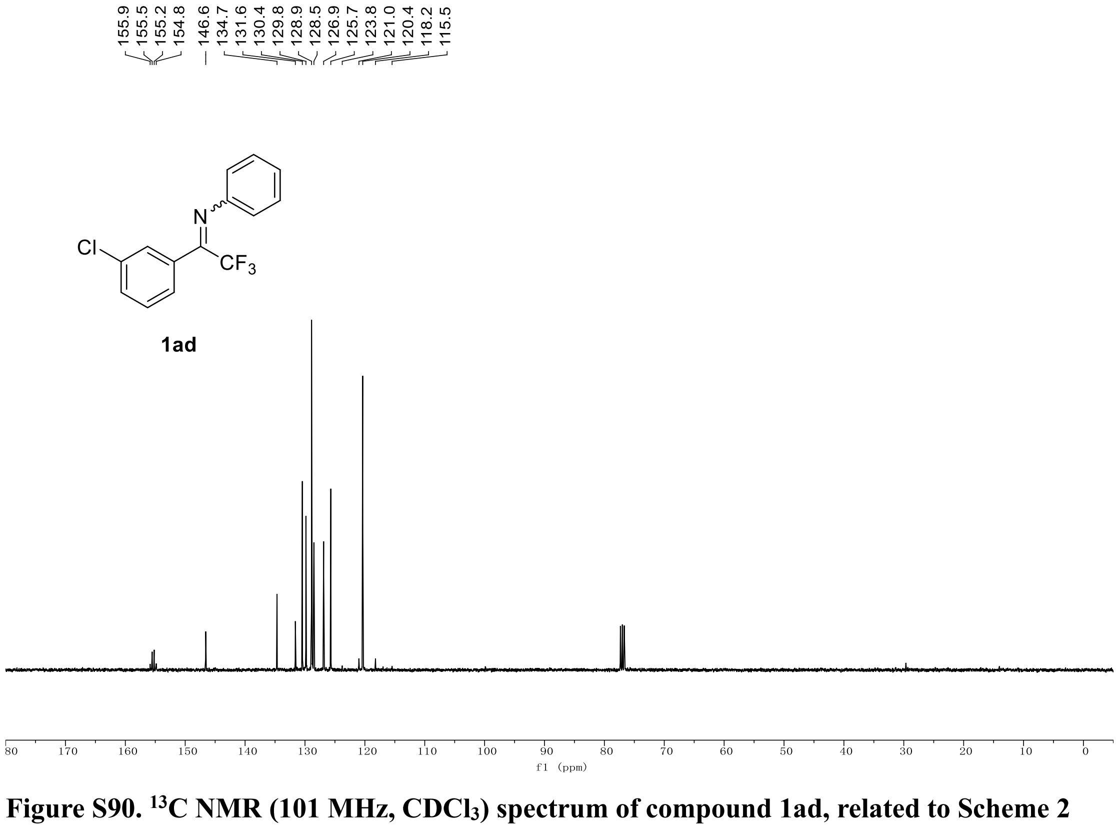

Figure S90. [13]**C NMR (101 MHz, CDCl₃) spectrum of compound 1ad, related to Scheme 2**

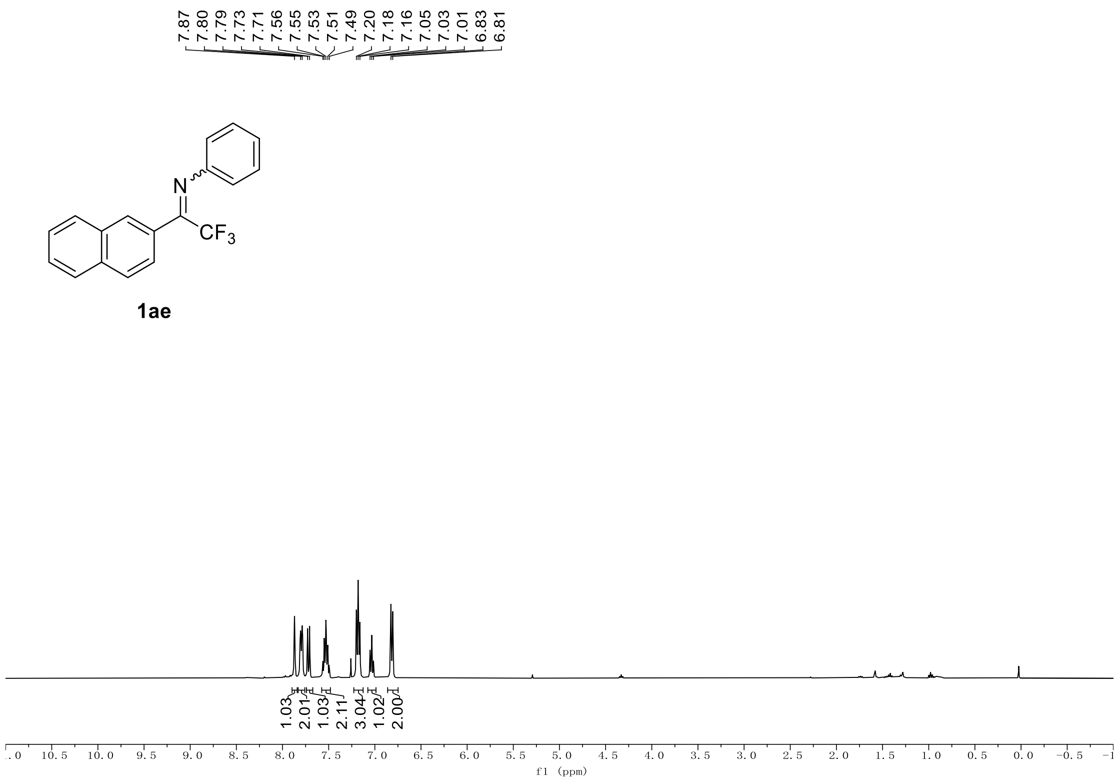

Figure S91. ^{1}H NMR (400 MHz, $CDCl_3$) spectrum of compound 1ae, related to Scheme 2

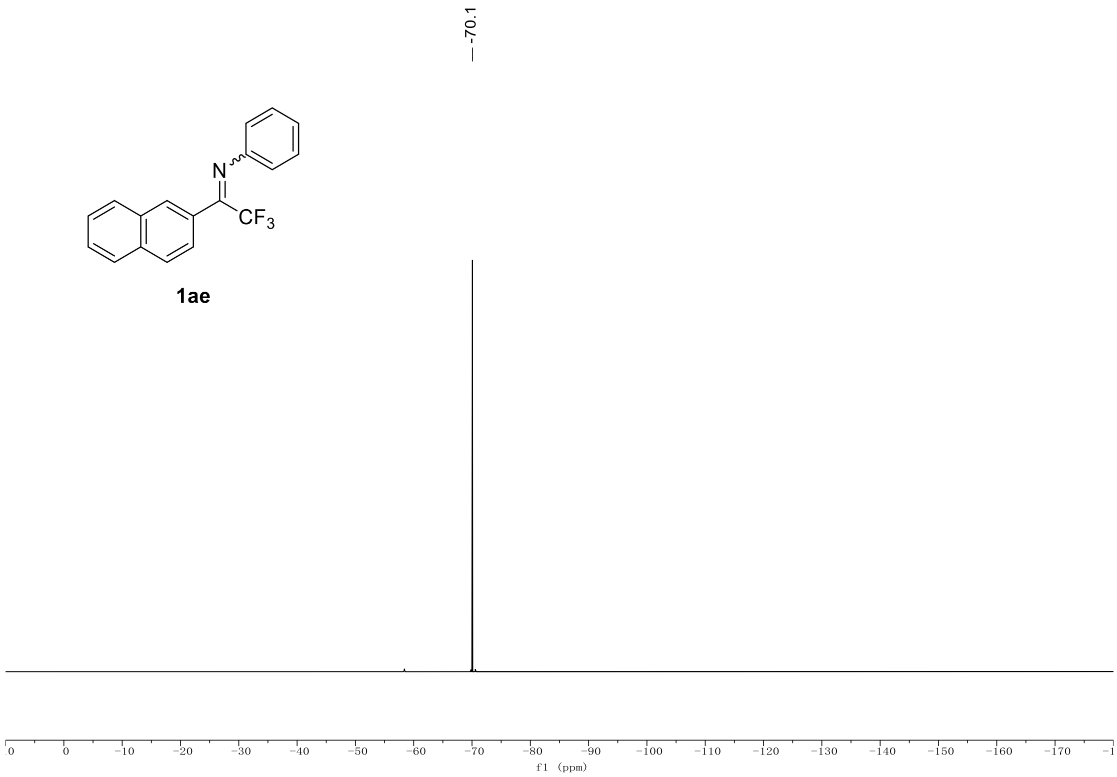

Figure S92. ^{19}F NMR (376 MHz, $CDCl_3$) spectrum of compound 1ae, related to Scheme 2

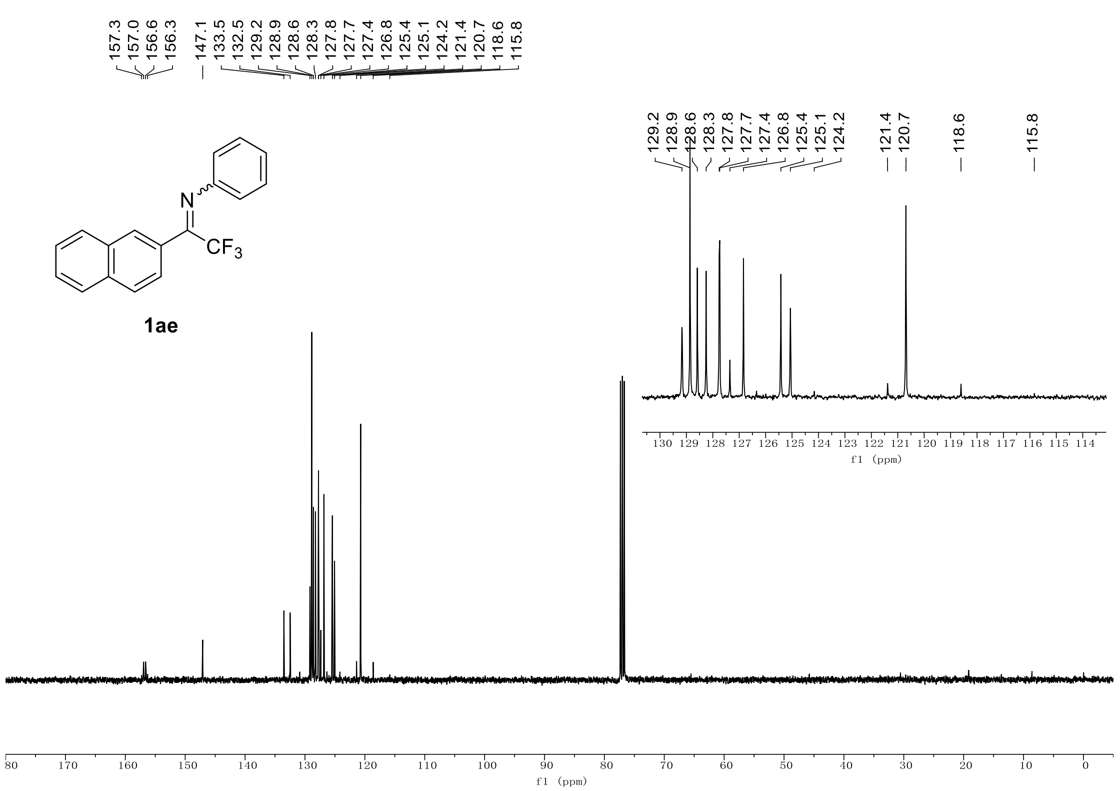

Figure S93. ^{13}C NMR (101 MHz, $CDCl_3$) spectrum of compound 1ae, related to Scheme 2

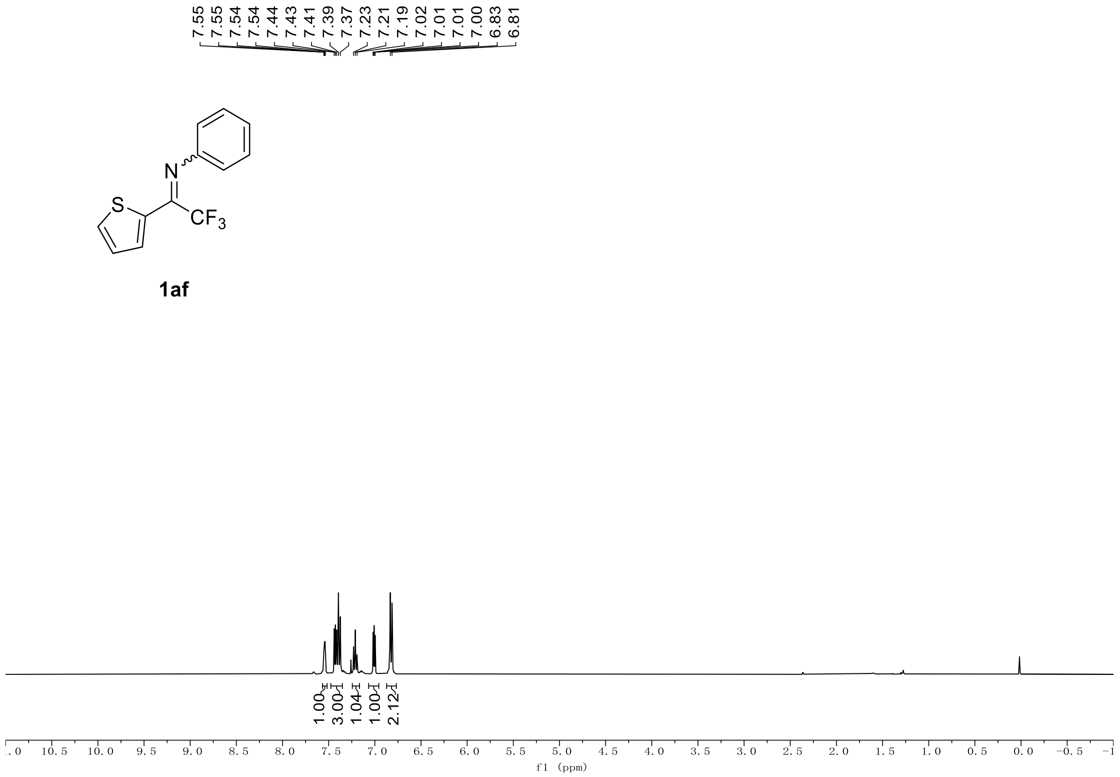

Figure S94. ^{1}H NMR (400 MHz, $CDCl_3$) spectrum of compound 1af, related to Scheme 2

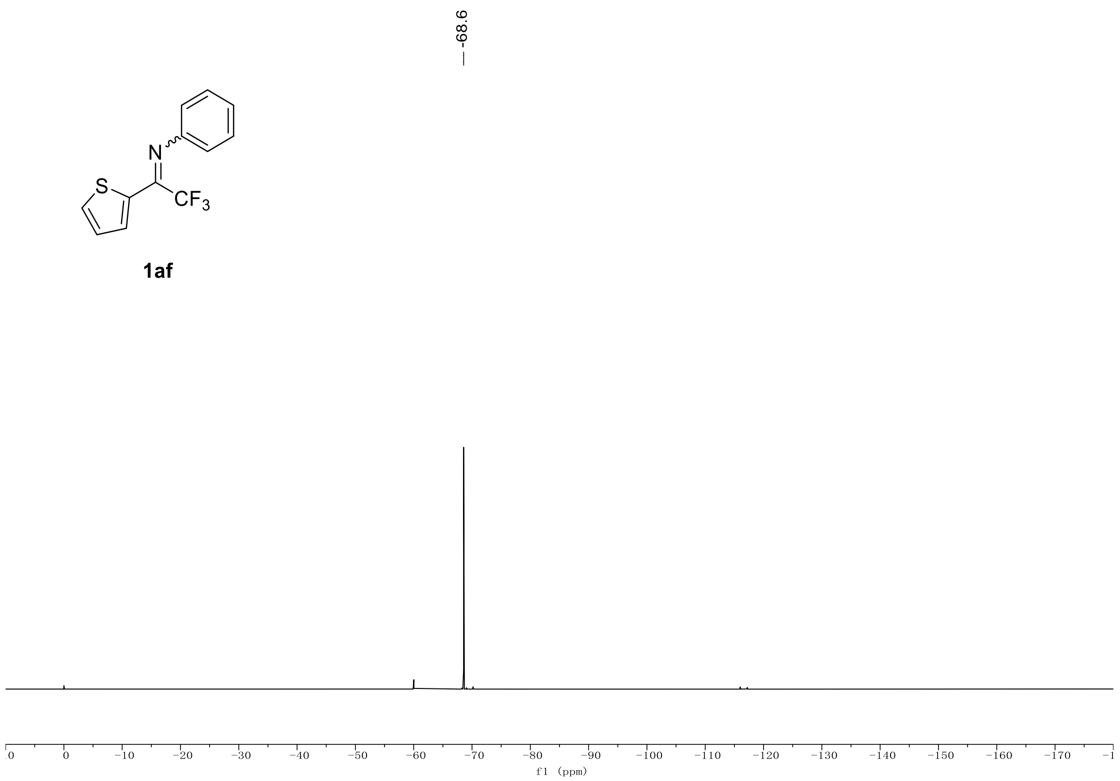

Figure S95. ^{19}F NMR (376 MHz, $CDCl_3$) spectrum of compound 1af, related to Scheme 2

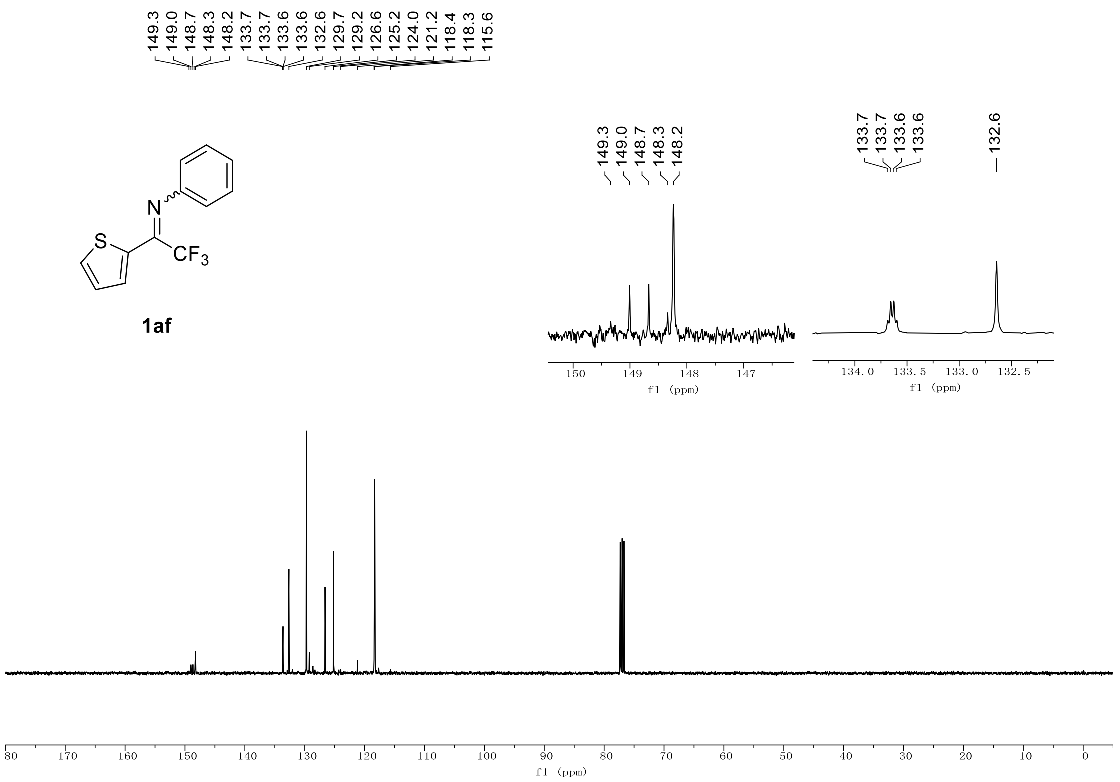

Figure S96. ^{13}C NMR (101 MHz, $CDCl_3$) spectrum of compound 1af, related to Scheme 2

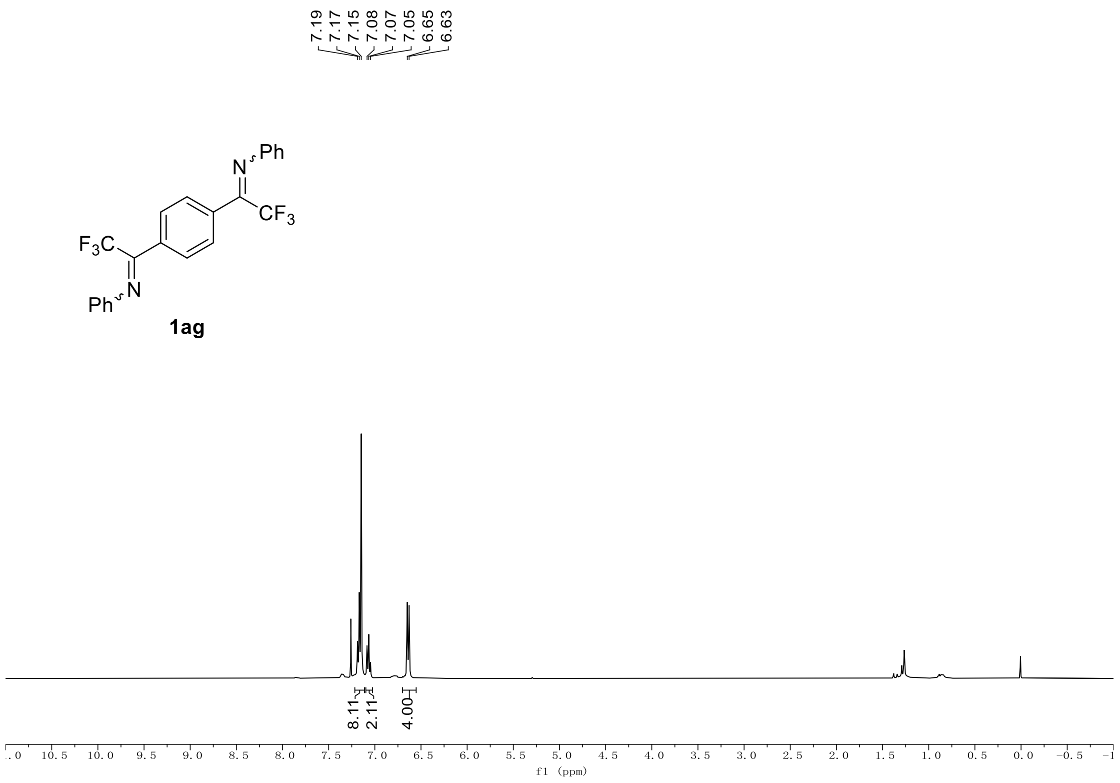

Figure S97. ^{1}H NMR (400 MHz, $CDCl_3$) spectrum of compound 1ag, related to Scheme 2

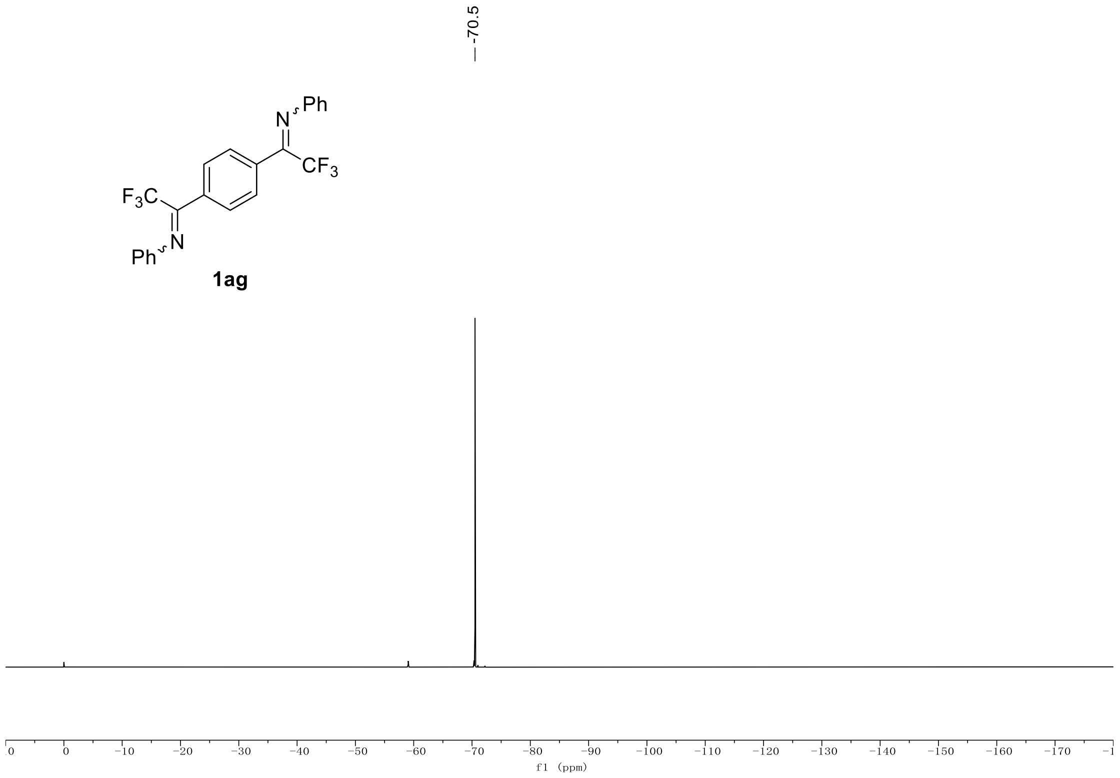

Figure S98. ^{19}F NMR (376 MHz, $CDCl_3$) spectrum of compound 1ag, related to Scheme 2

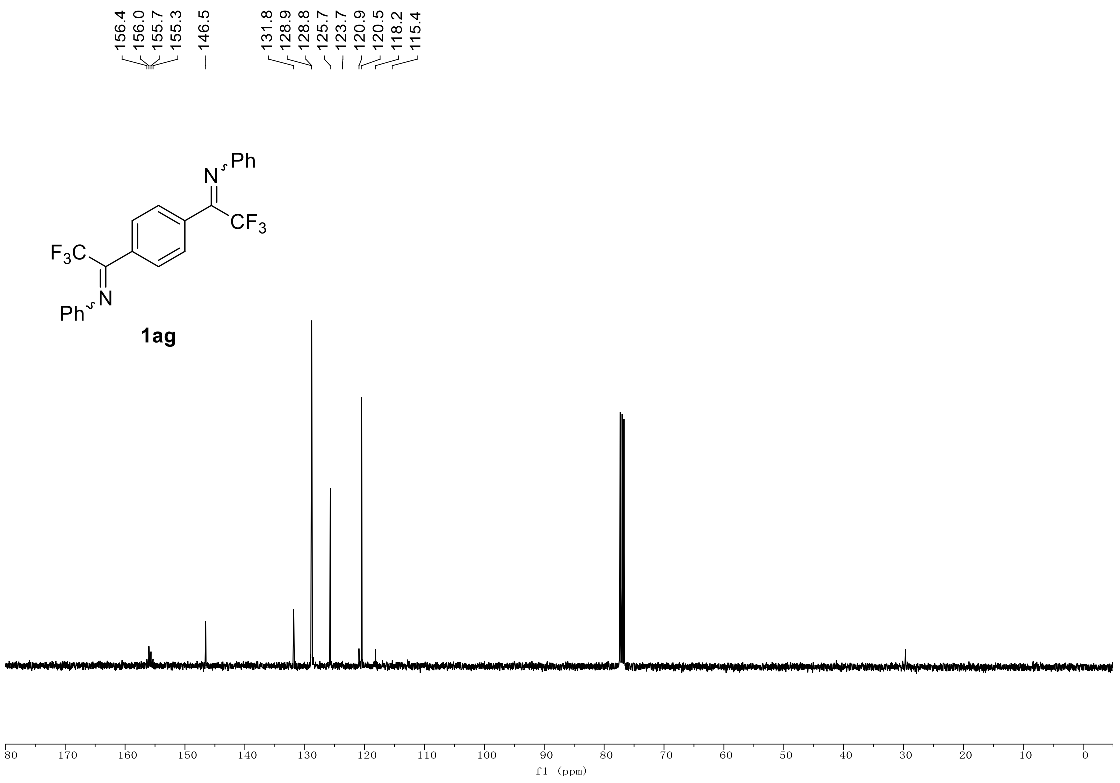

Figure S99. ^{13}C NMR (101 MHz, $CDCl_3$) spectrum of compound 1ag, related to Scheme 2

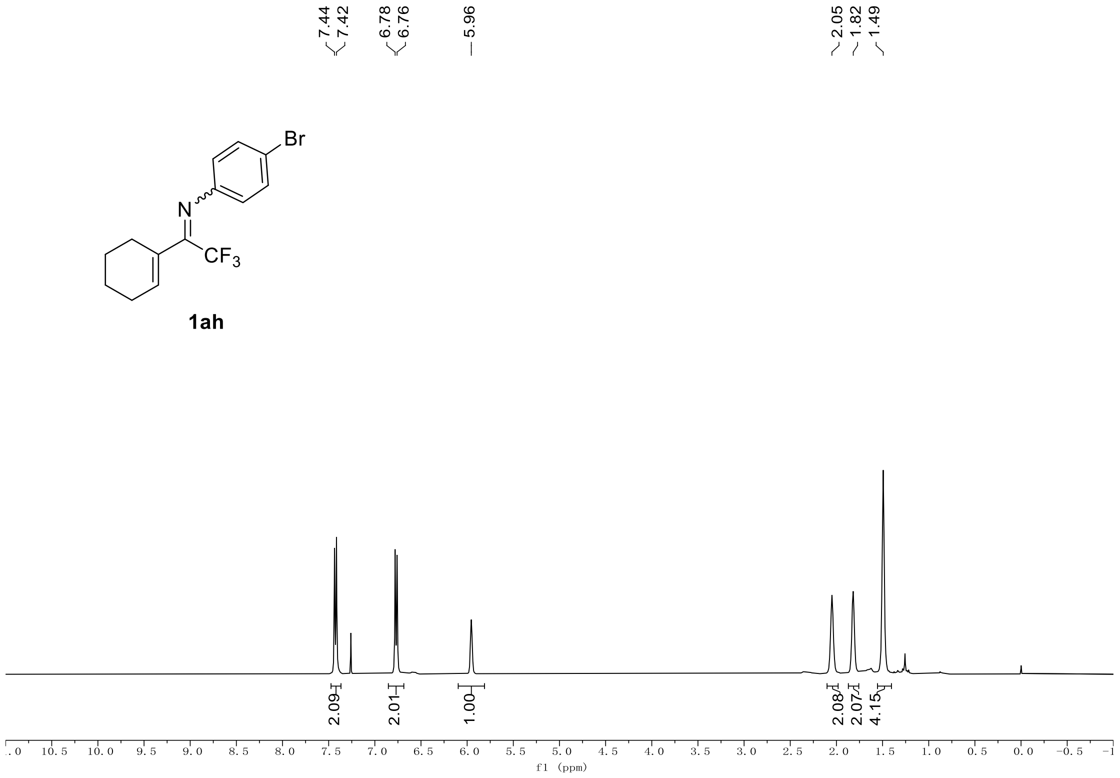

Figure S100. ^{1}H NMR (400 MHz, $CDCl_3$) spectrum of compound 1ah, related to Scheme 2

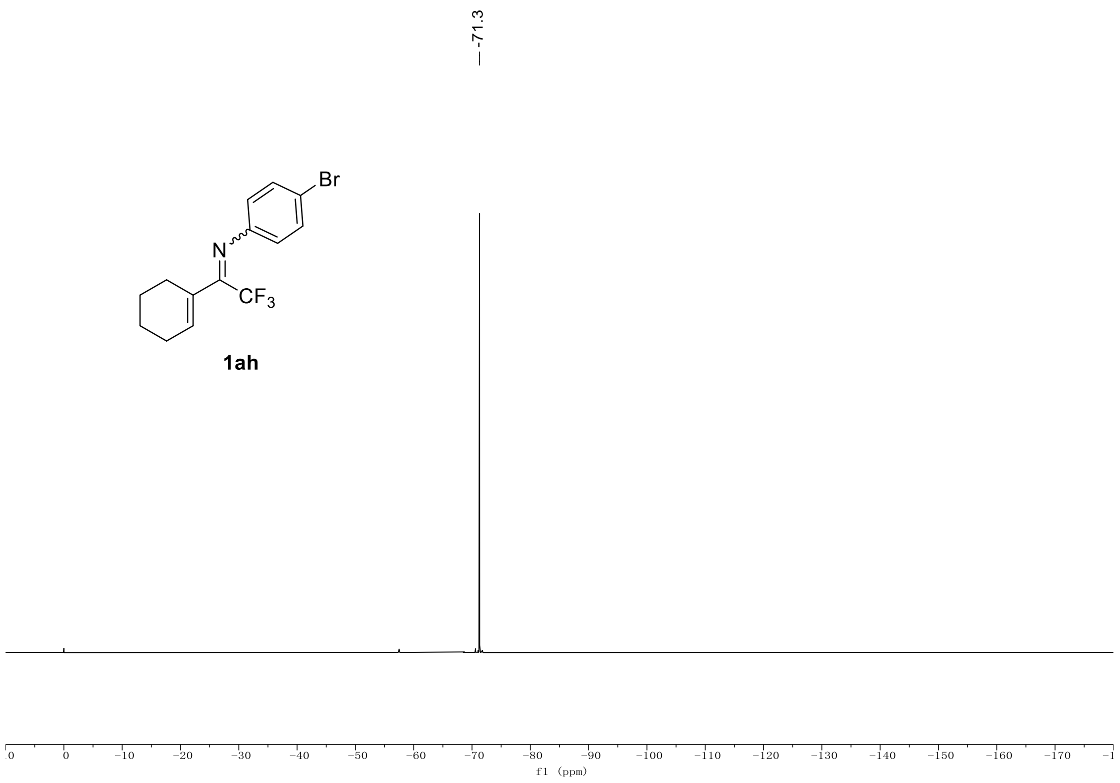

Figure S101. ^{19}F NMR (376 MHz, $CDCl_3$) spectrum of compound 1ah, related to Scheme 2

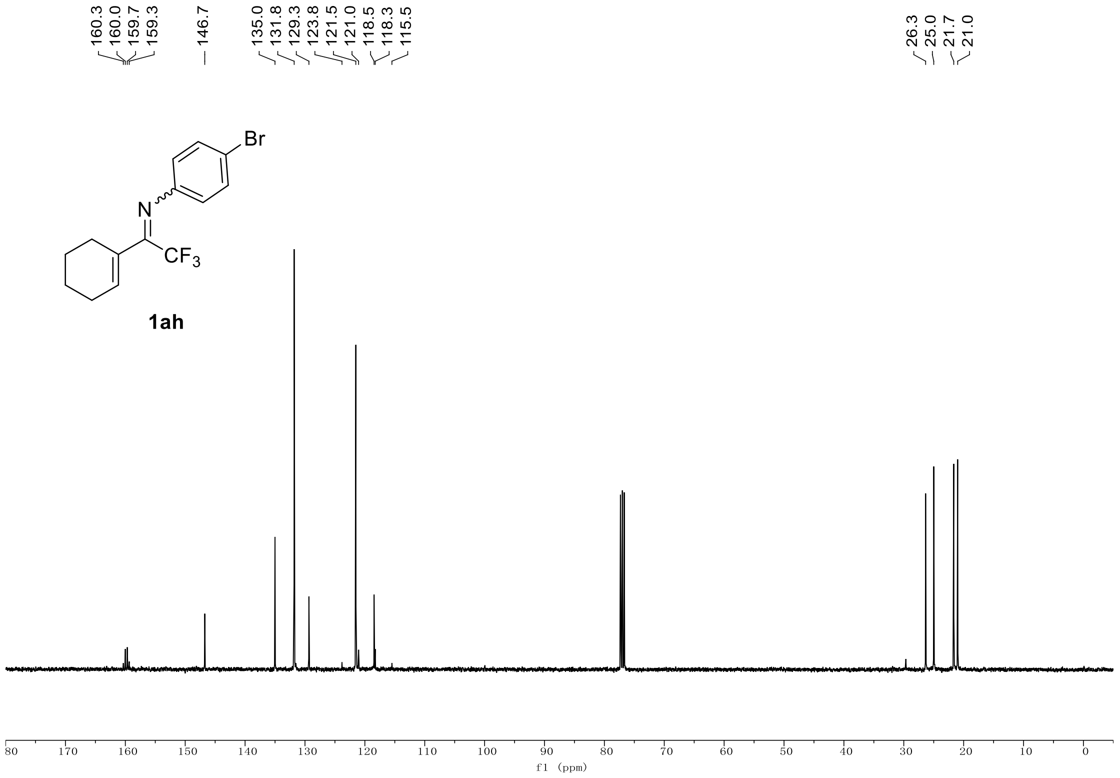

Figure S102. ^{13}C NMR (101 MHz, $CDCl_3$) spectrum of compound 1ah, related to Scheme 2

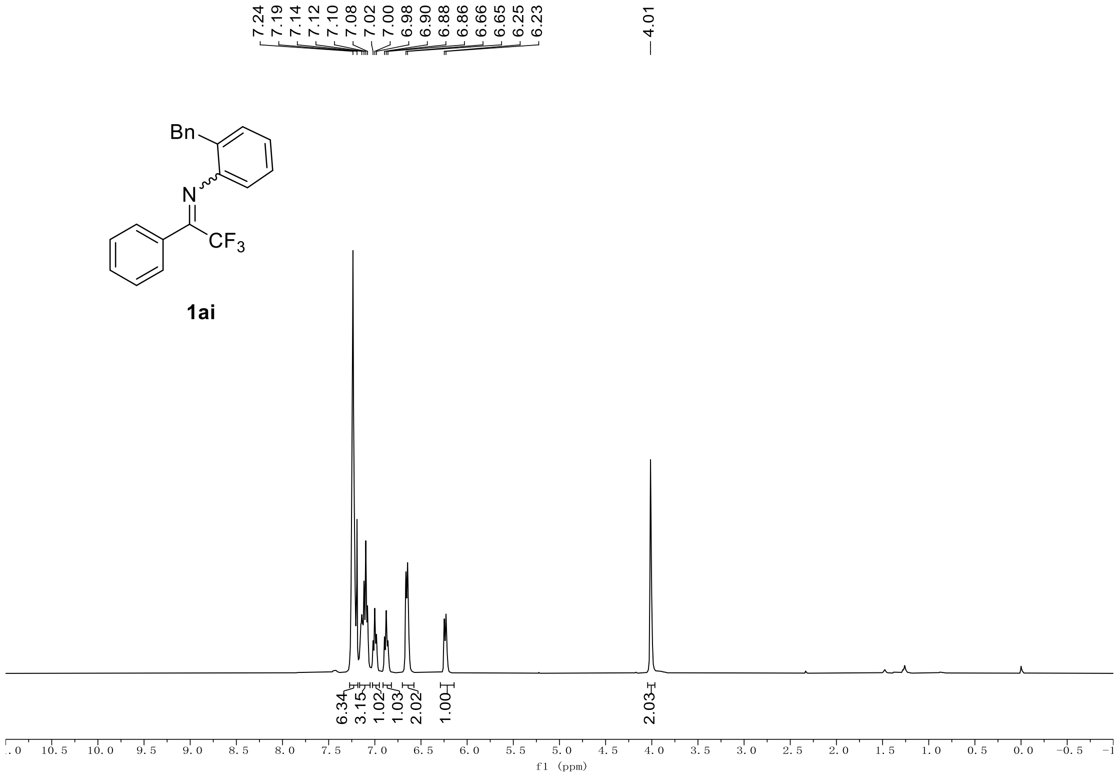

Figure S103. ^{1}H NMR (400 MHz, $CDCl_3$) spectrum of compound 1ai, related to Figure 2

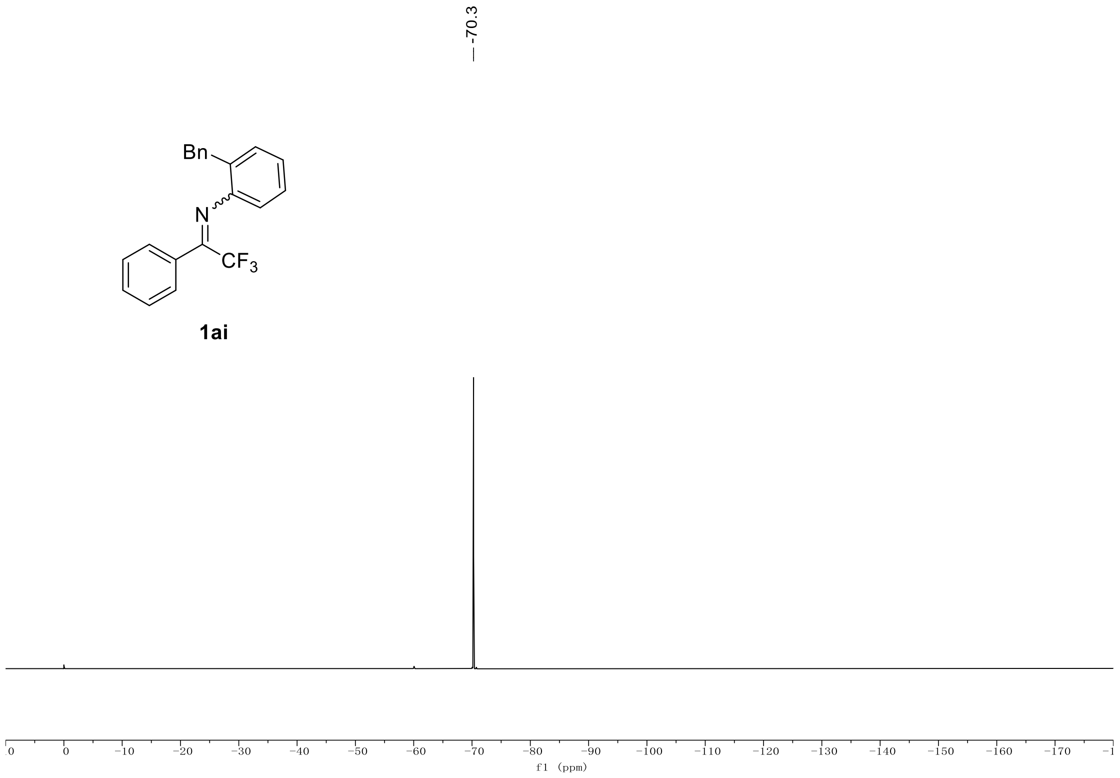

Figure S104. ^{19}F NMR (376 MHz, $CDCl_3$) spectrum of compound 1ai, related to Figure 2

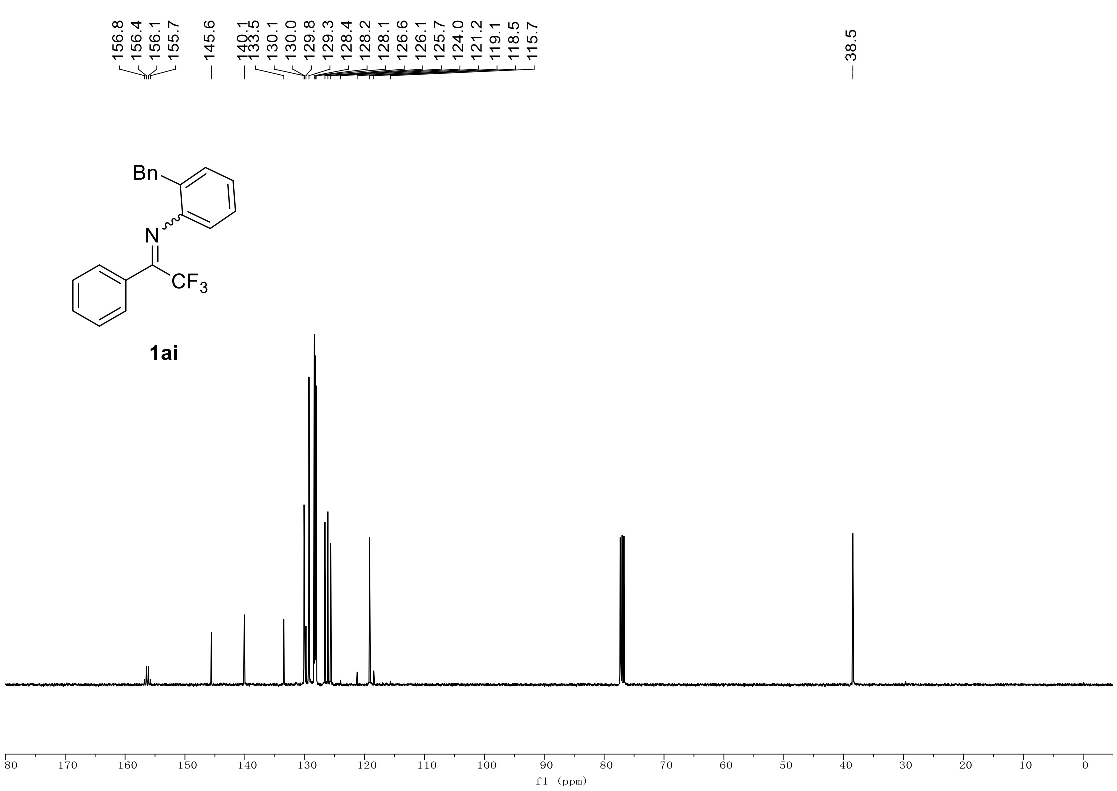

Figure S105. ^{13}C NMR (101 MHz, $CDCl_3$) spectrum of compound 1ai, related to Figure 2

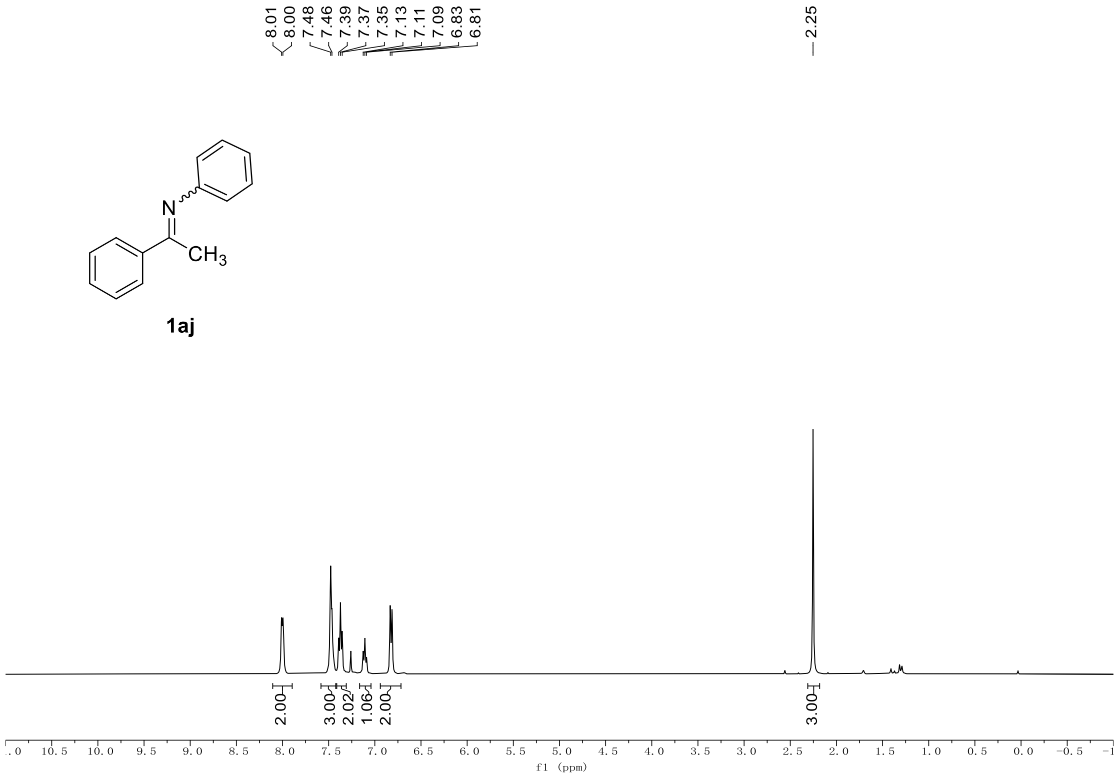

Figure S106. ^{1}H NMR (400 MHz, $CDCl_3$) spectrum of compound 1aj, related to Scheme 5

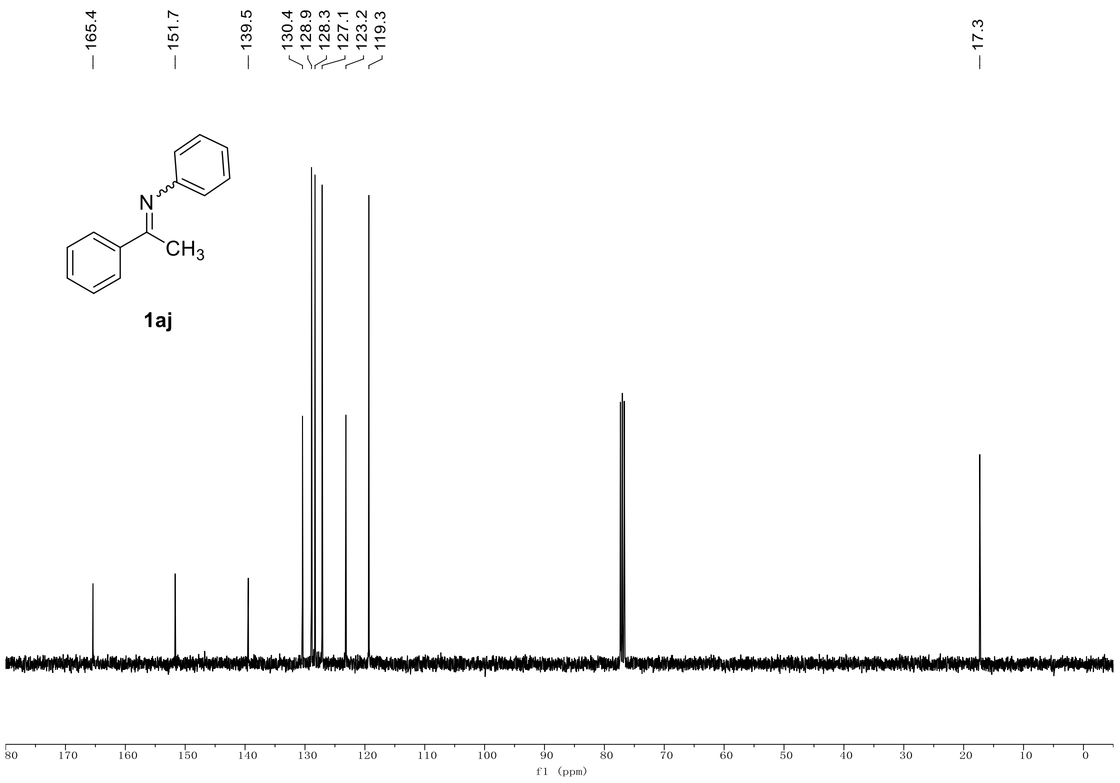

Figure S107. ^{13}C NMR (101 MHz, $CDCl_3$) spectrum of compound 1aj, related to Scheme 5

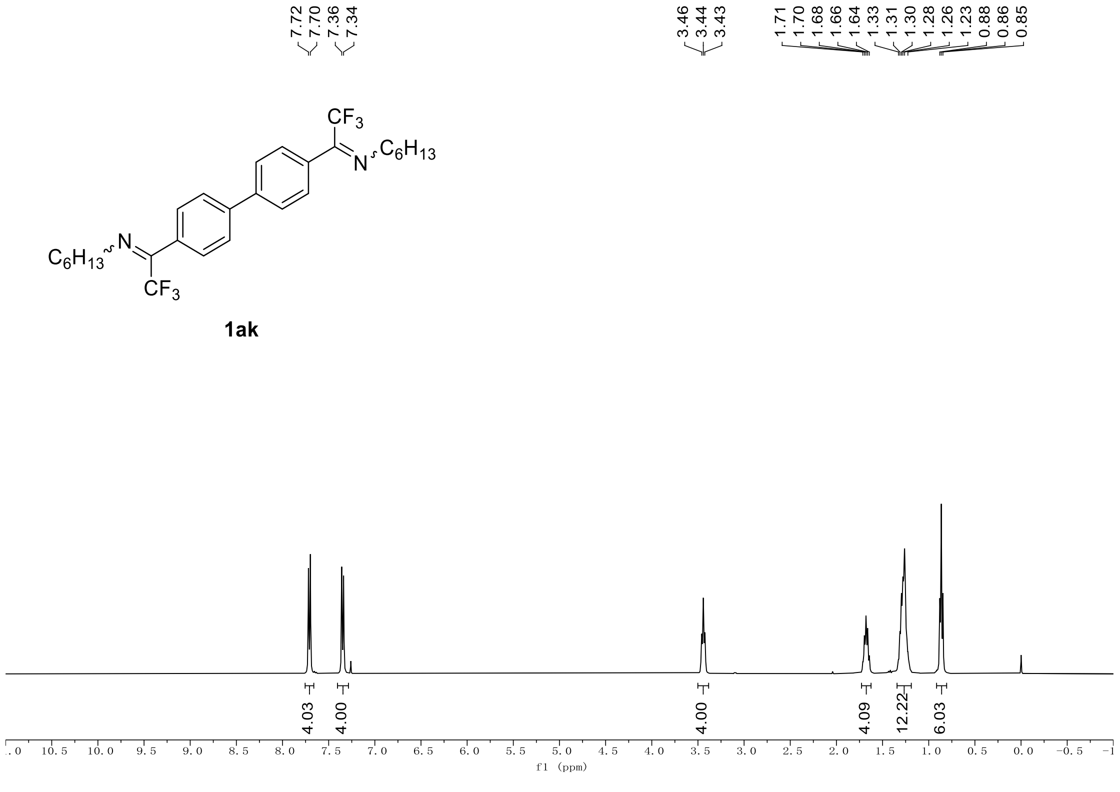

Figure S108. ^{1}H NMR (400 MHz, $CDCl_3$) spectrum of compound 1ak, related to Figure 3

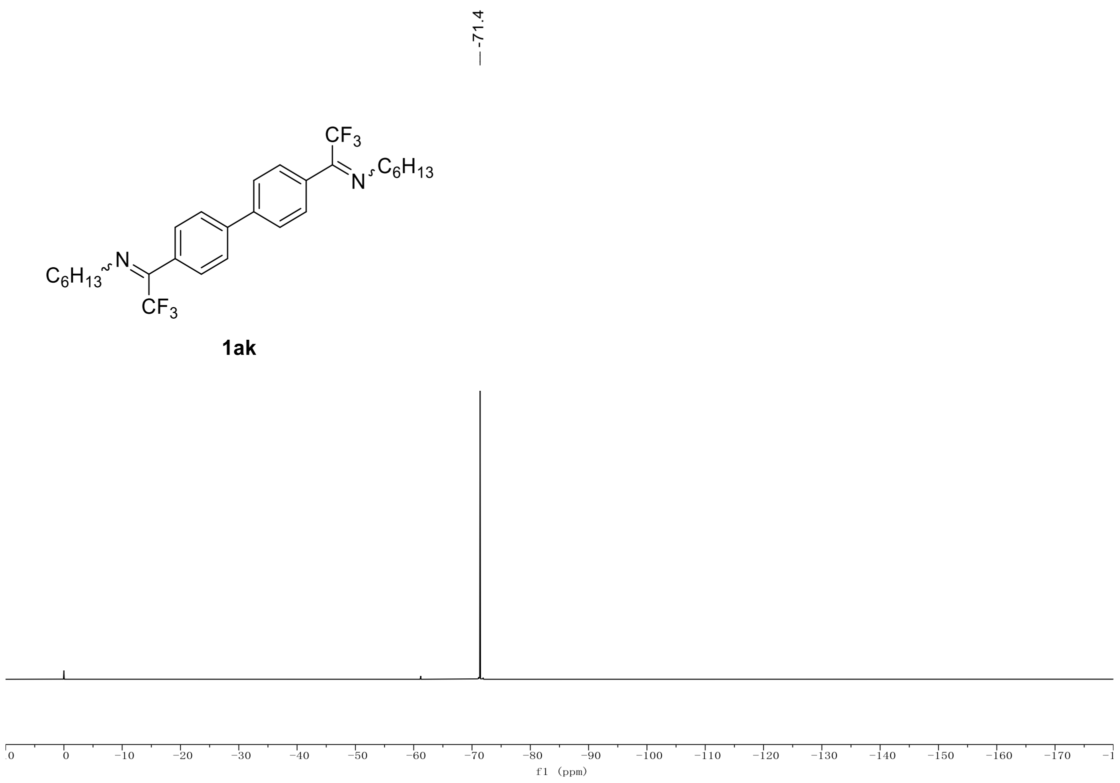

Figure S109. ^{19}F NMR (376 MHz, $CDCl_3$) spectrum of compound 1ak, related to Figure 3

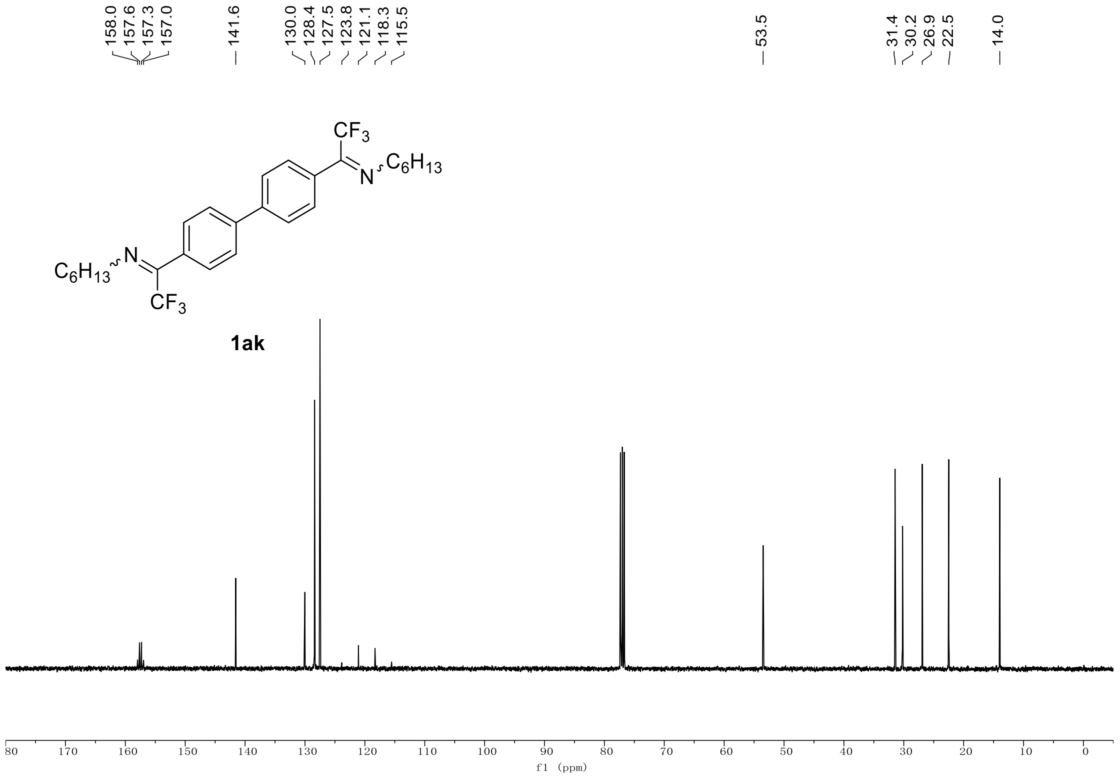

Figure S110. ^{13}C NMR (101 MHz, $CDCl_3$) spectrum of compound 1ak, related to Figure 3

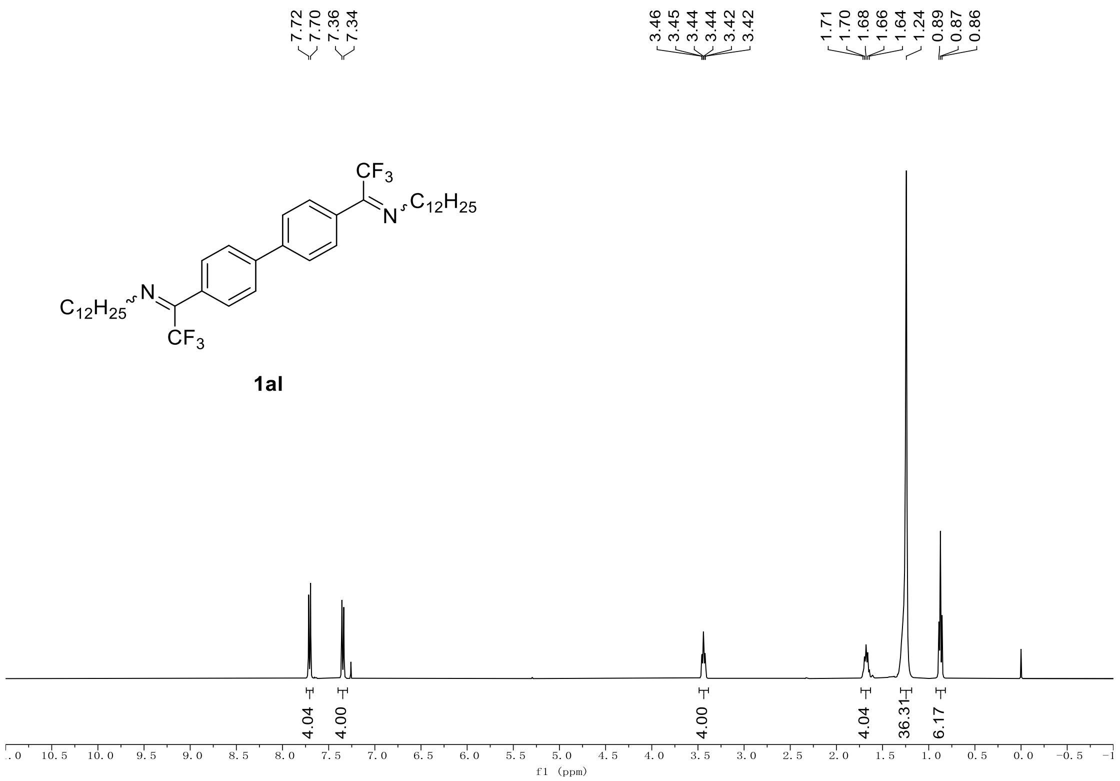

Figure S111. ^{1}H NMR (400 MHz, $CDCl_3$) spectrum of compound 1al, related to Figure 3

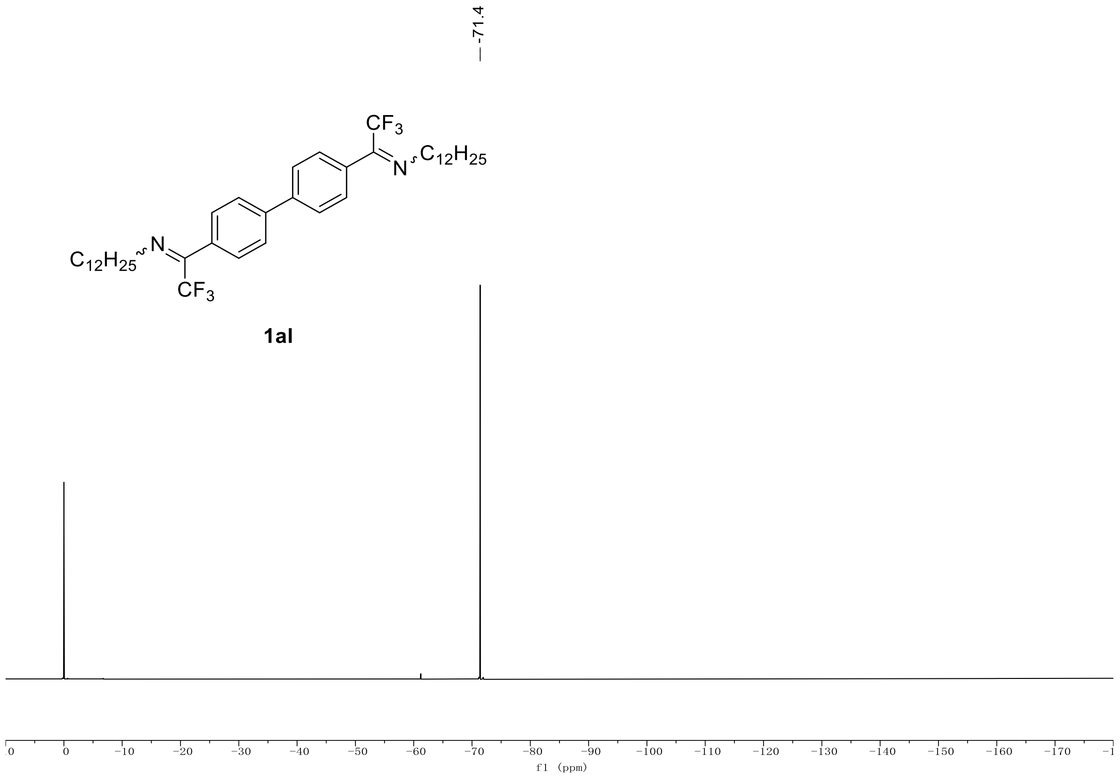

Figure S112. ^{19}F NMR (376 MHz, $CDCl_3$) spectrum of compound 1al, related to Figure 3

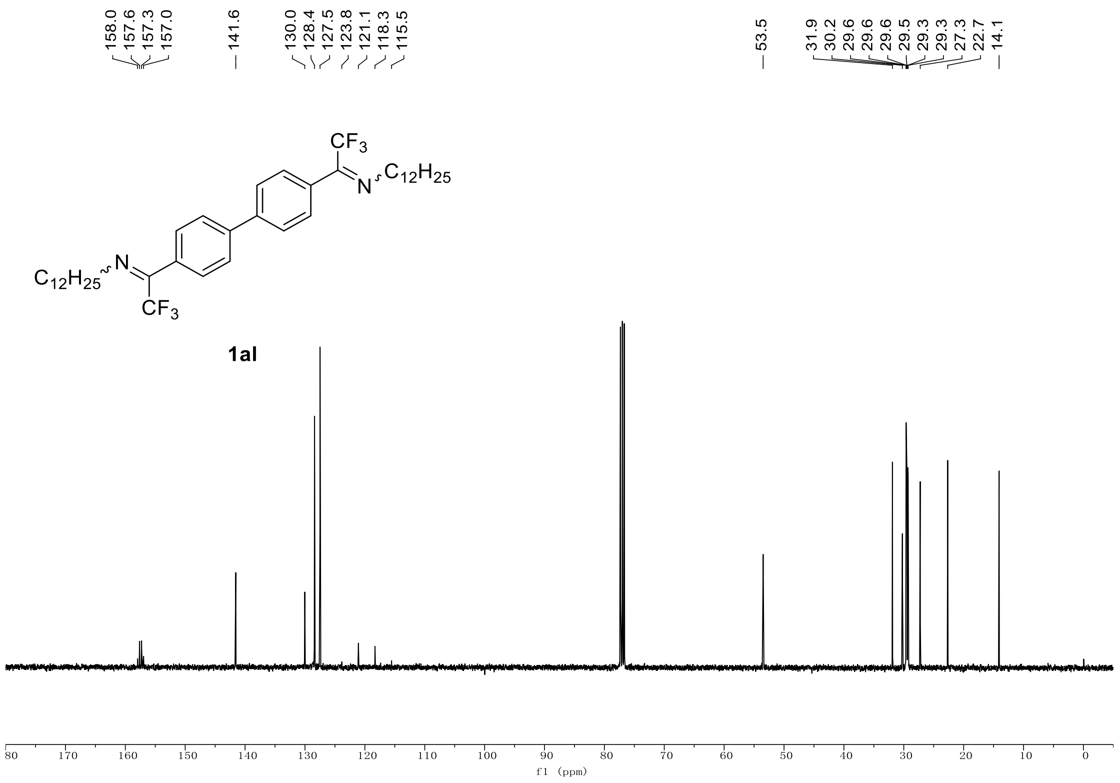

Figure S113. ^{13}C NMR (101 MHz, $CDCl_3$) spectrum of compound 1al, related to Figure 3

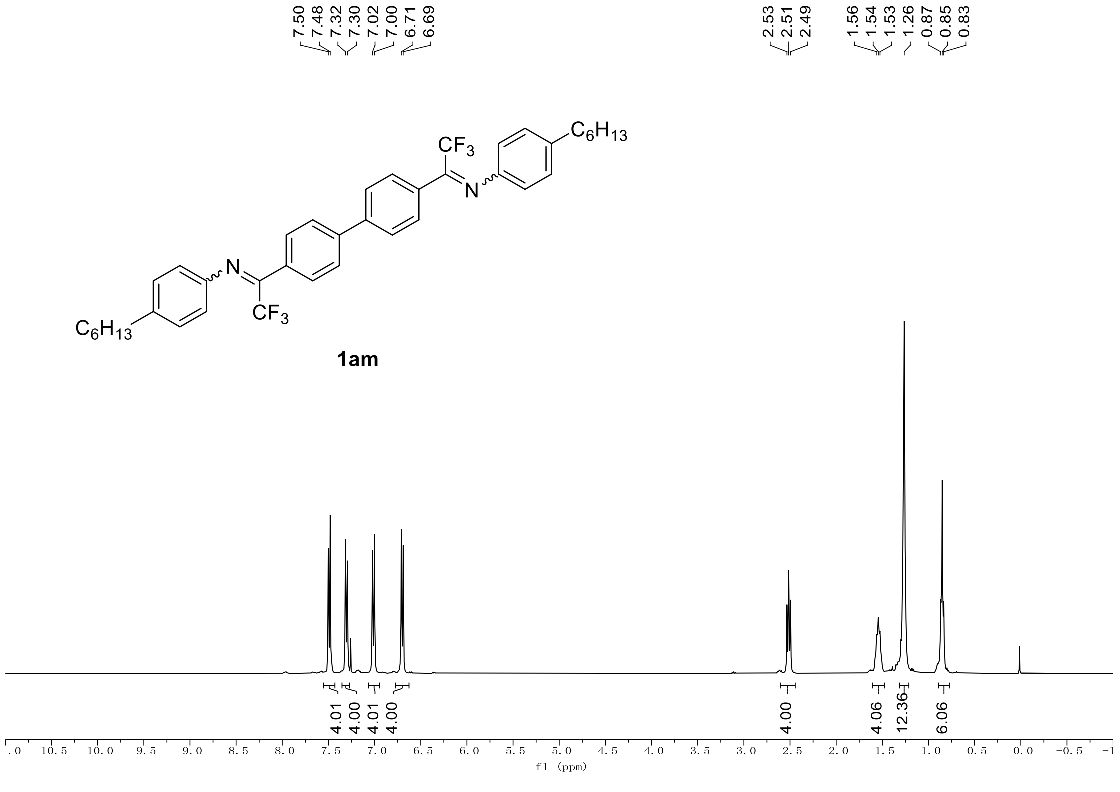

Figure S114. ^{1}H NMR (400 MHz, $CDCl_3$) spectrum of compound 1am, related to Figure 3

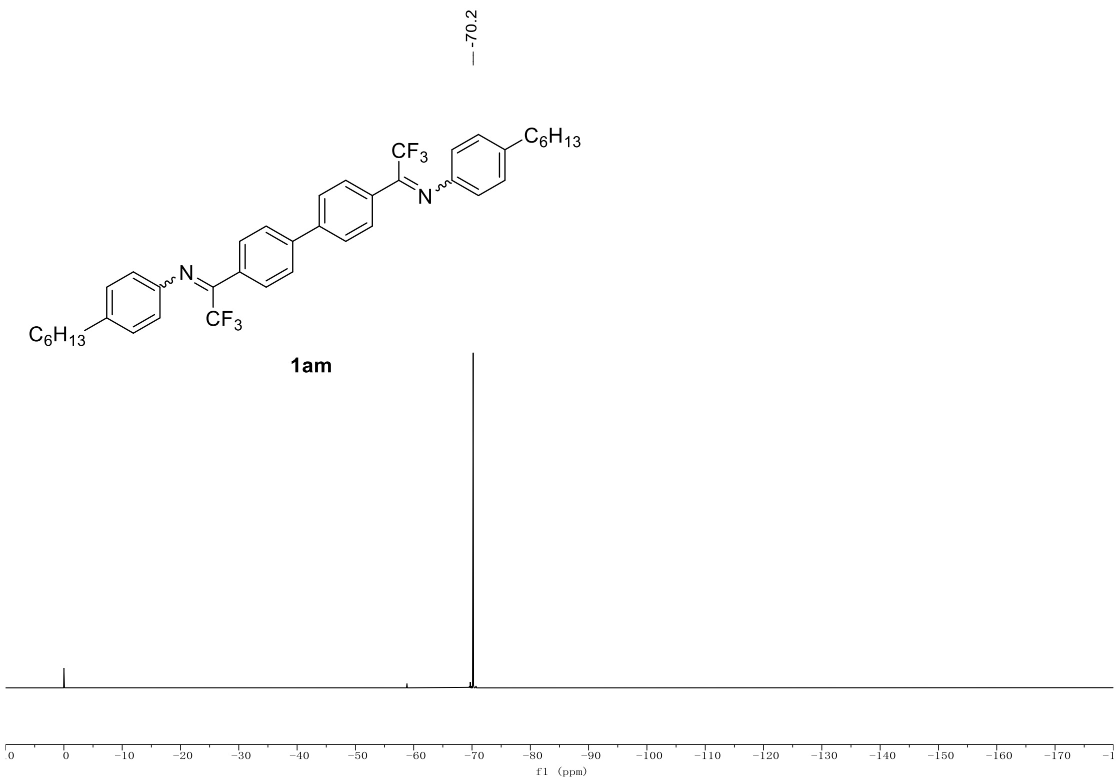

Figure S115. ^{19}F NMR (376 MHz, $CDCl_3$) spectrum of compound 1am, related to Figure 3

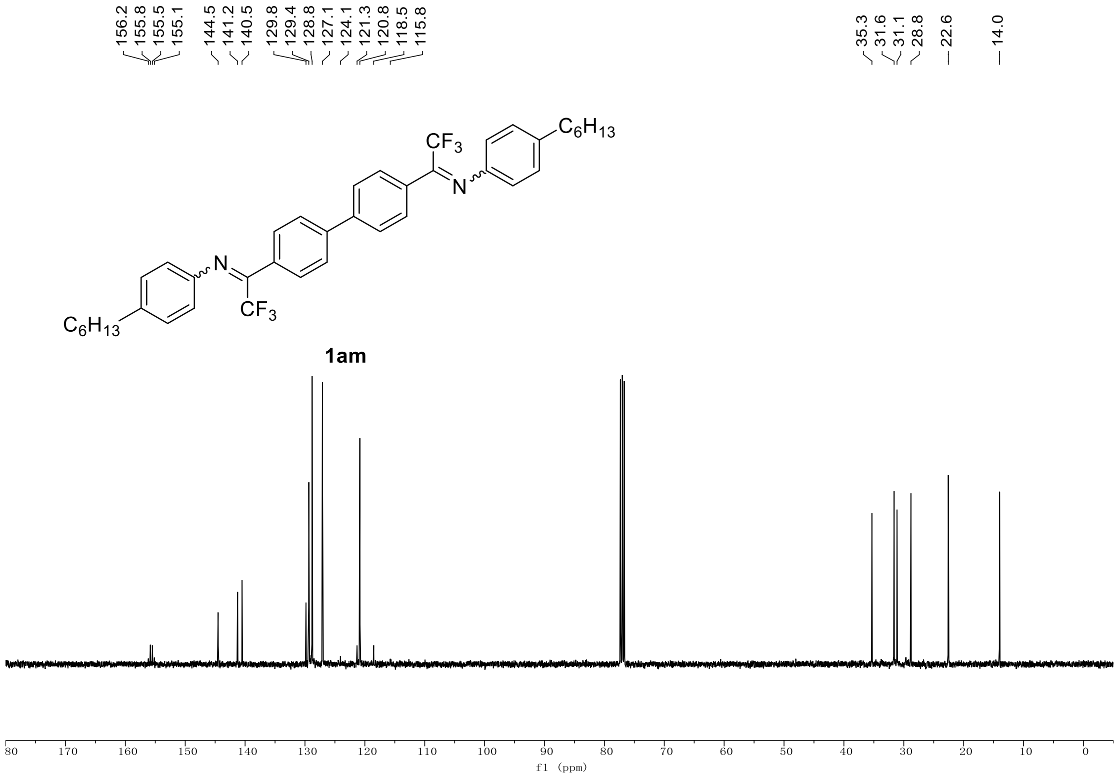

Figure S116. ^{13}C NMR (101 MHz, $CDCl_3$) spectrum of compound 1am, related to Figure 3

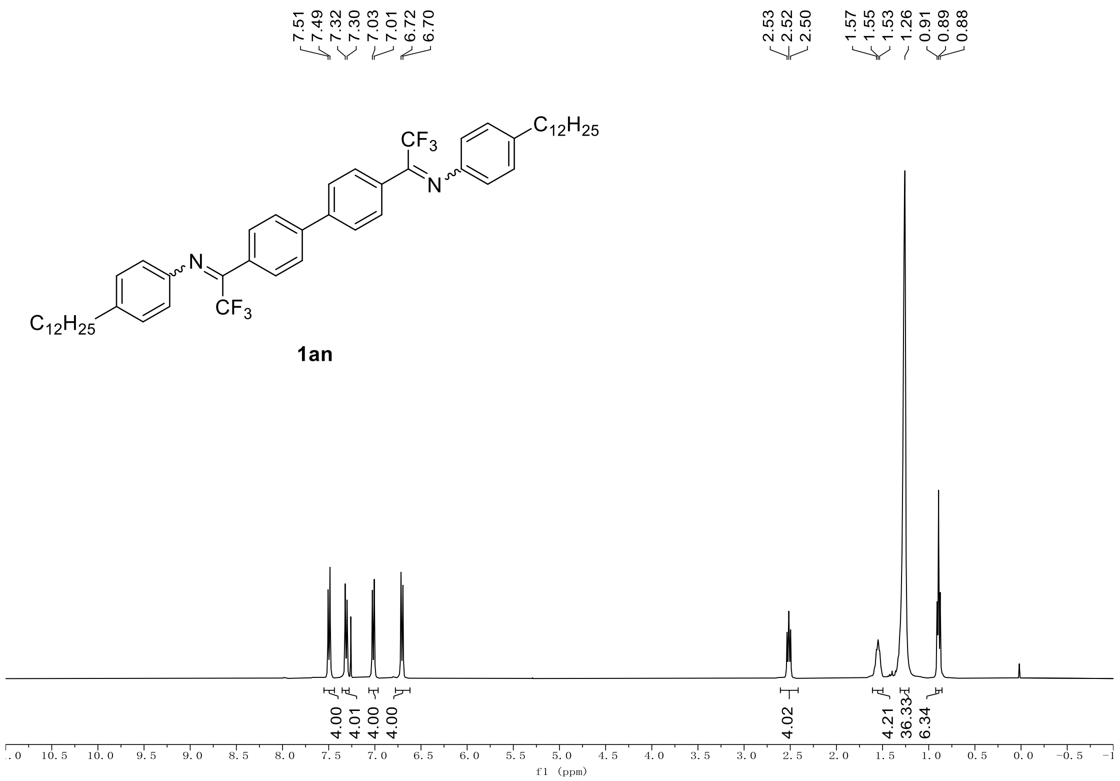

Figure S117. ^{1}H NMR (400 MHz, $CDCl_3$) spectrum of compound 1an, related to Figure 3

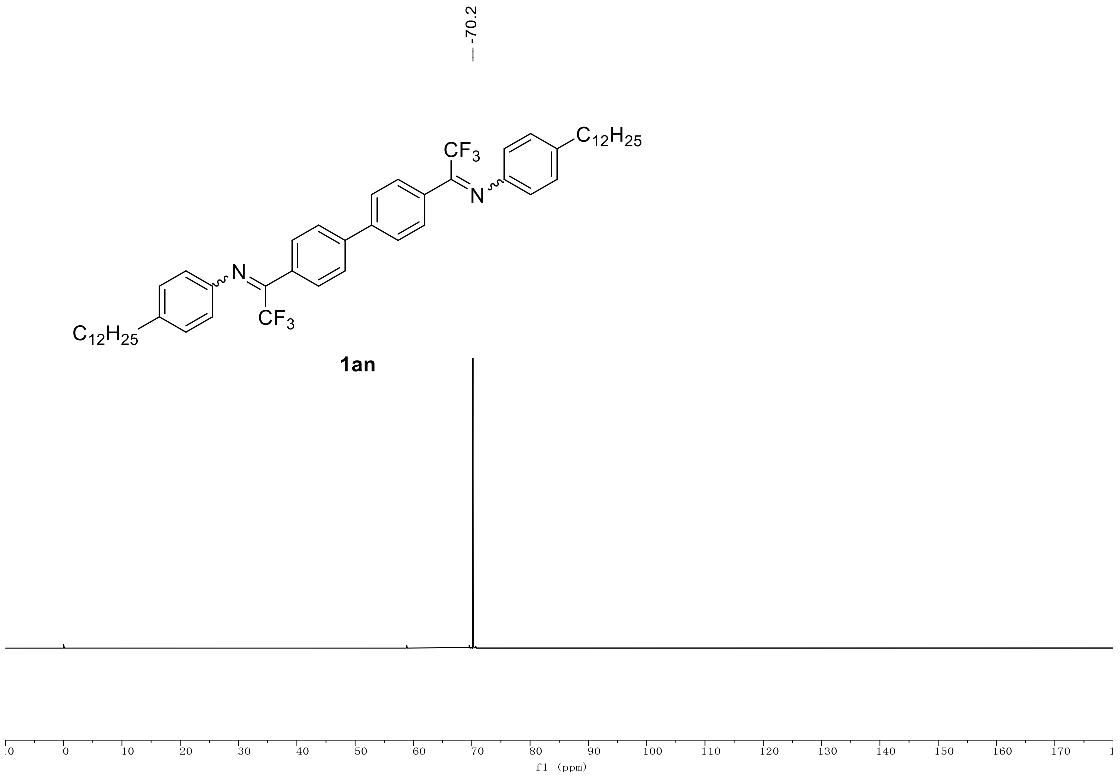

Figure S118. ^{19}F NMR (376 MHz, $CDCl_3$) spectrum of compound 1an, related to Figure 3

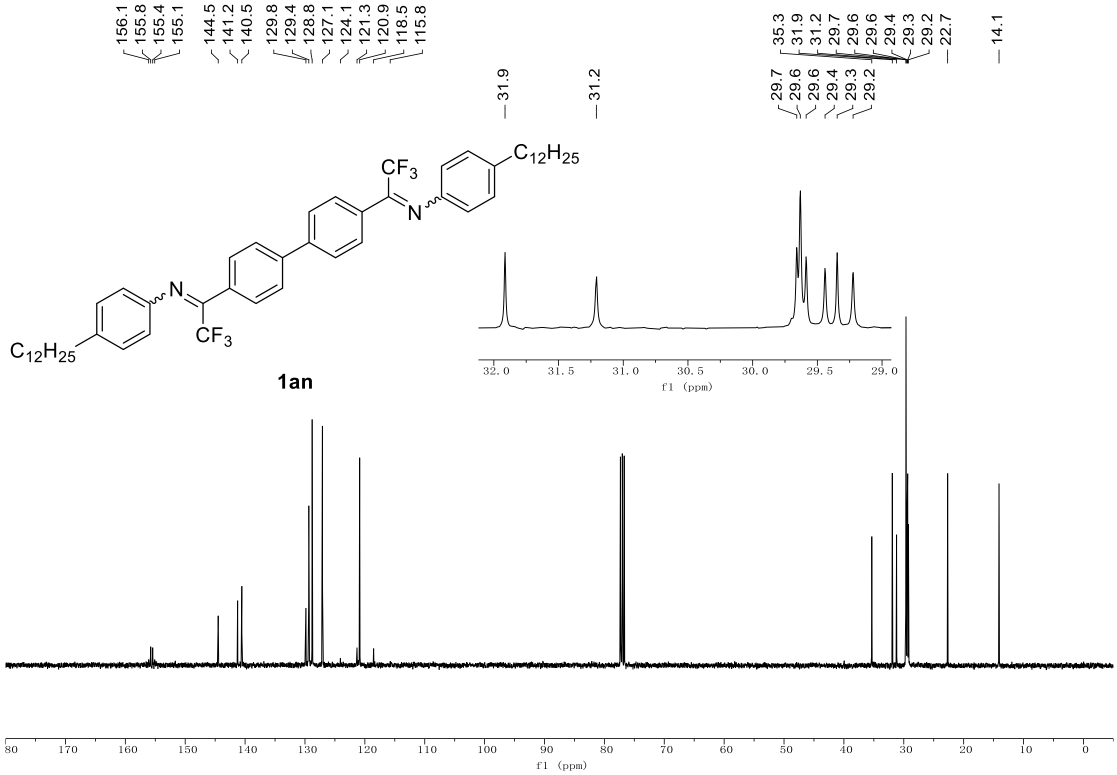

Figure S119. ^{13}C NMR (101 MHz, $CDCl_3$) spectrum of compound 1an, related to Figure 3

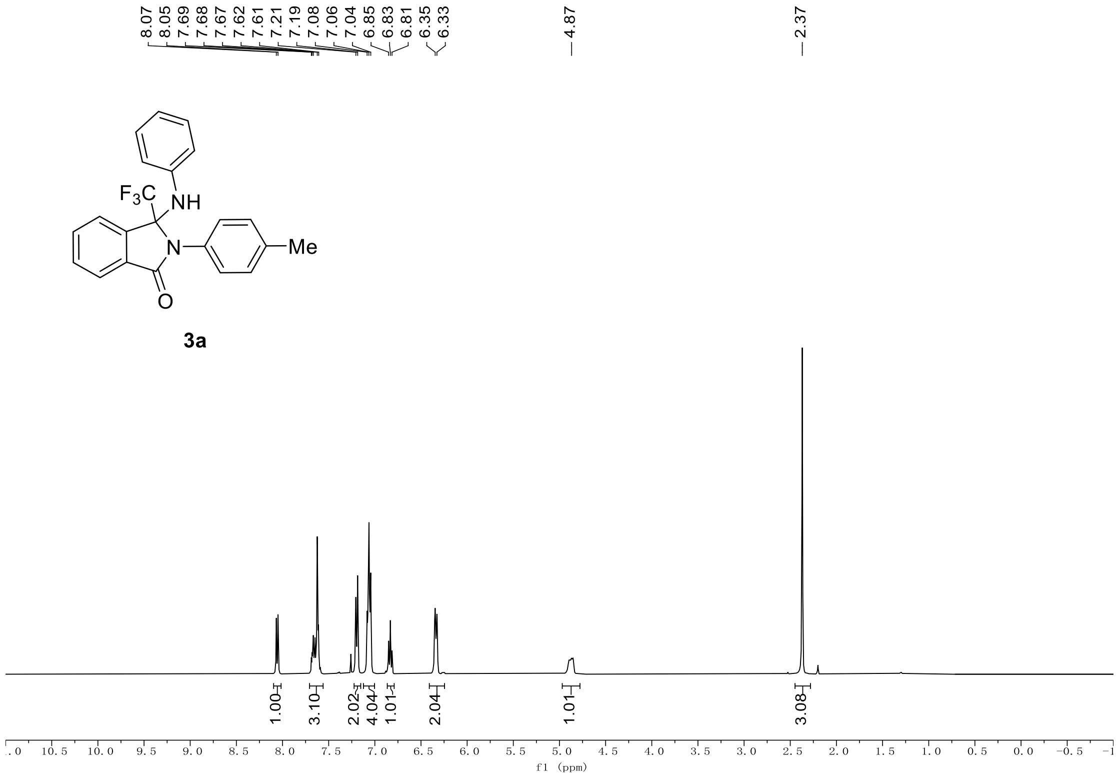

Figure S120. ^{1}H NMR (400 MHz, $CDCl_3$) spectrum of compound 3a, related to Table 1

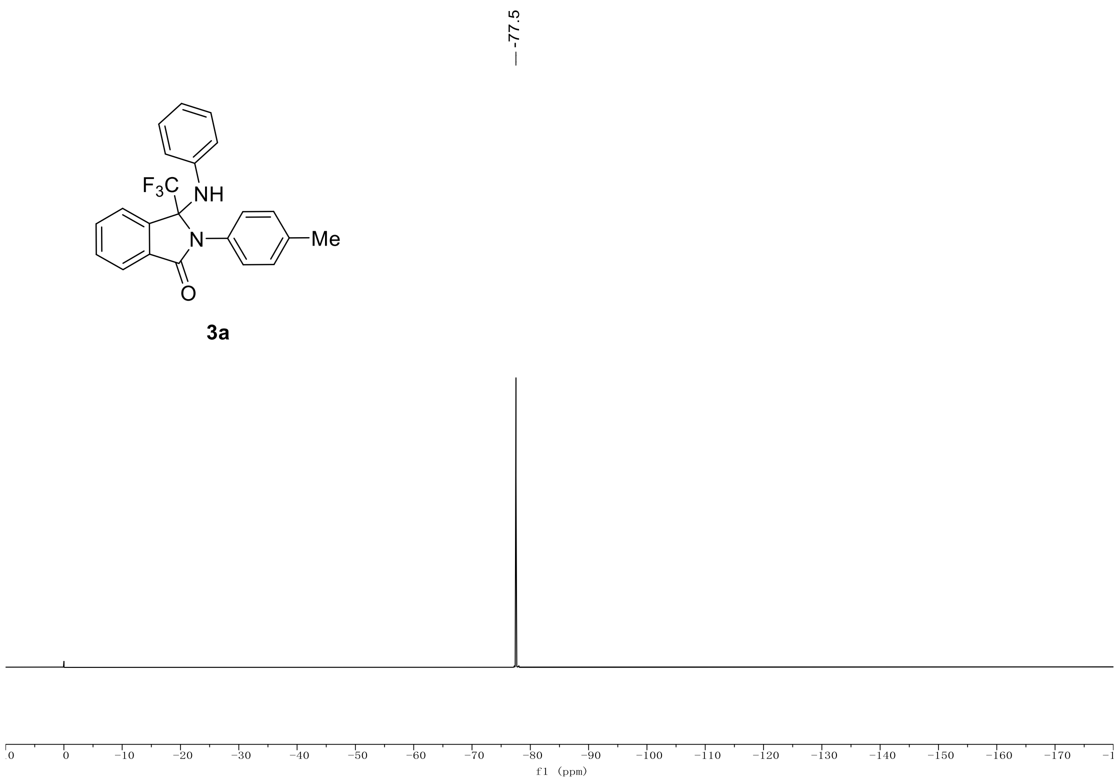

Figure S121. ^{19}**F NMR (376 MHz, CDCl$_3$) spectrum of compound 3a, related to Table 1**

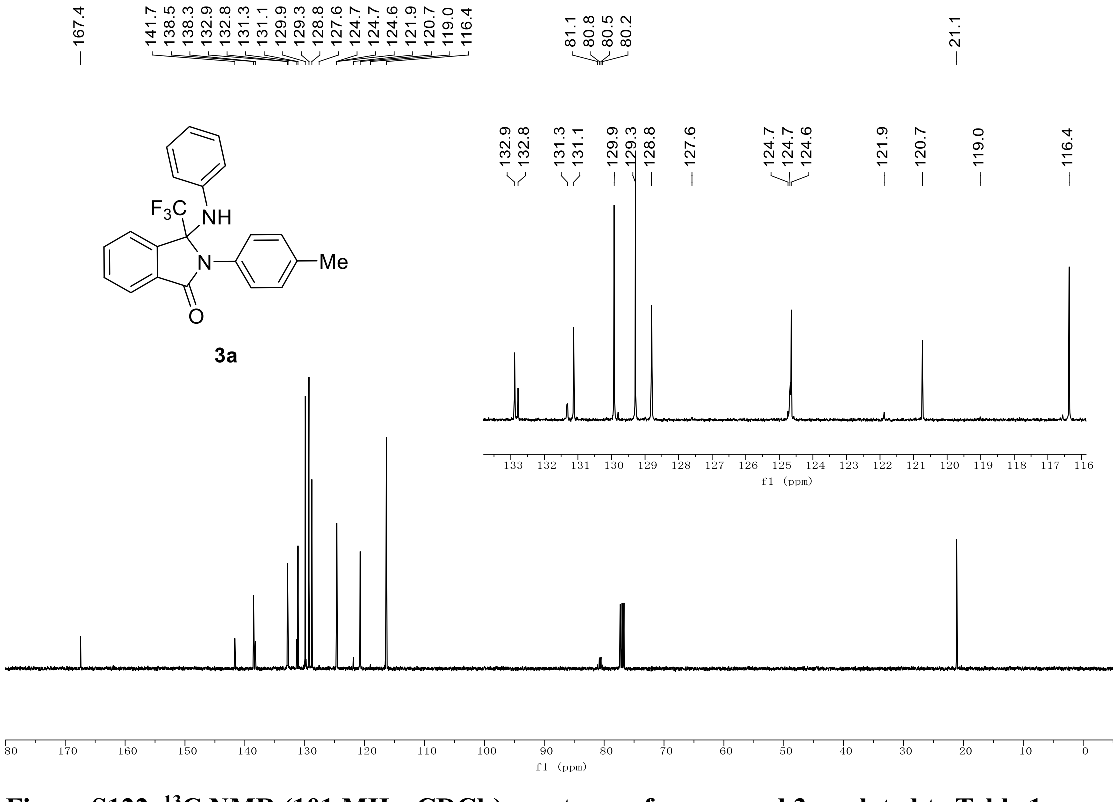

Figure S122. ^{13}**C NMR (101 MHz, CDCl$_3$) spectrum of compound 3a, related to Table 1**

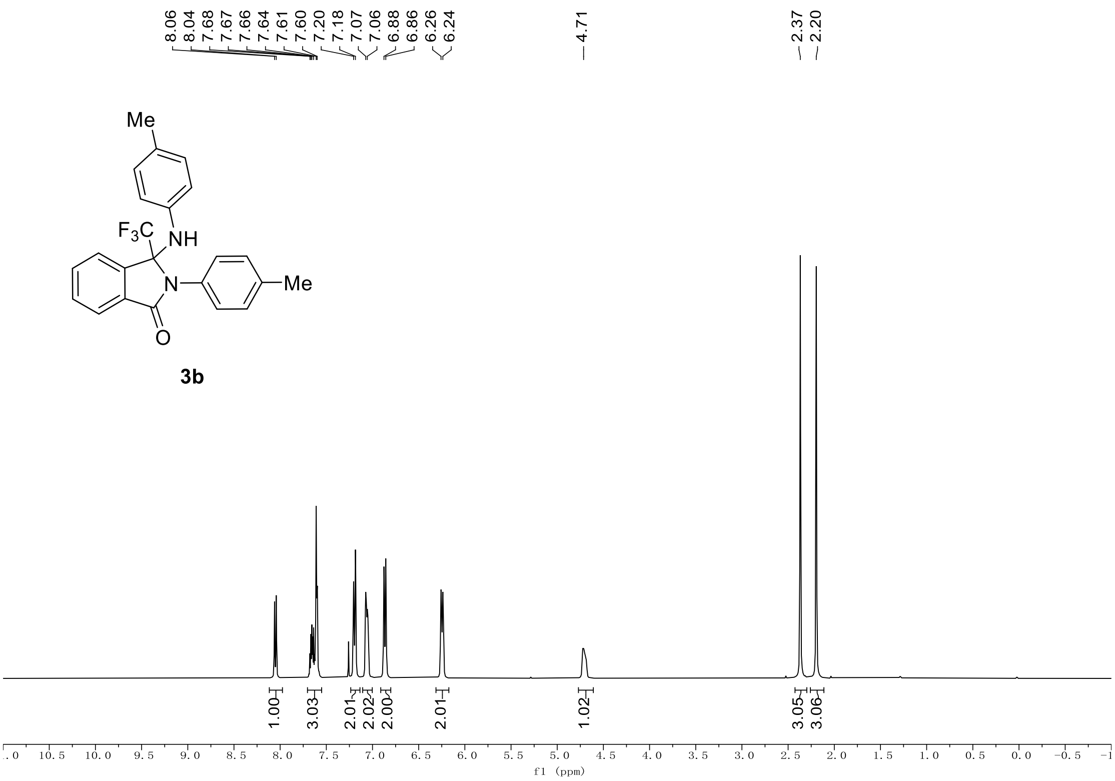

3b

Figure S123. ^{1}H NMR (400 MHz, CDCl$_3$) spectrum of compound 3b, related to Scheme 2

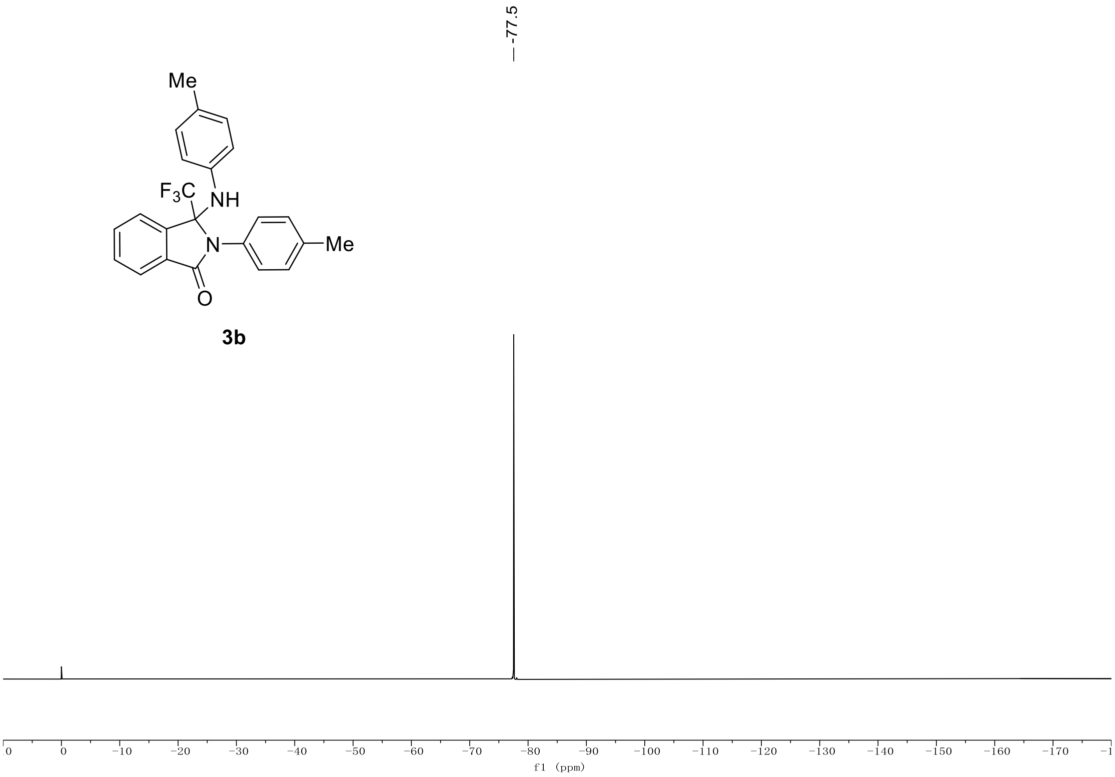

3b

Figure S124. ^{19}F NMR (376 MHz, CDCl$_3$) spectrum of compound 3b, related to Scheme 2

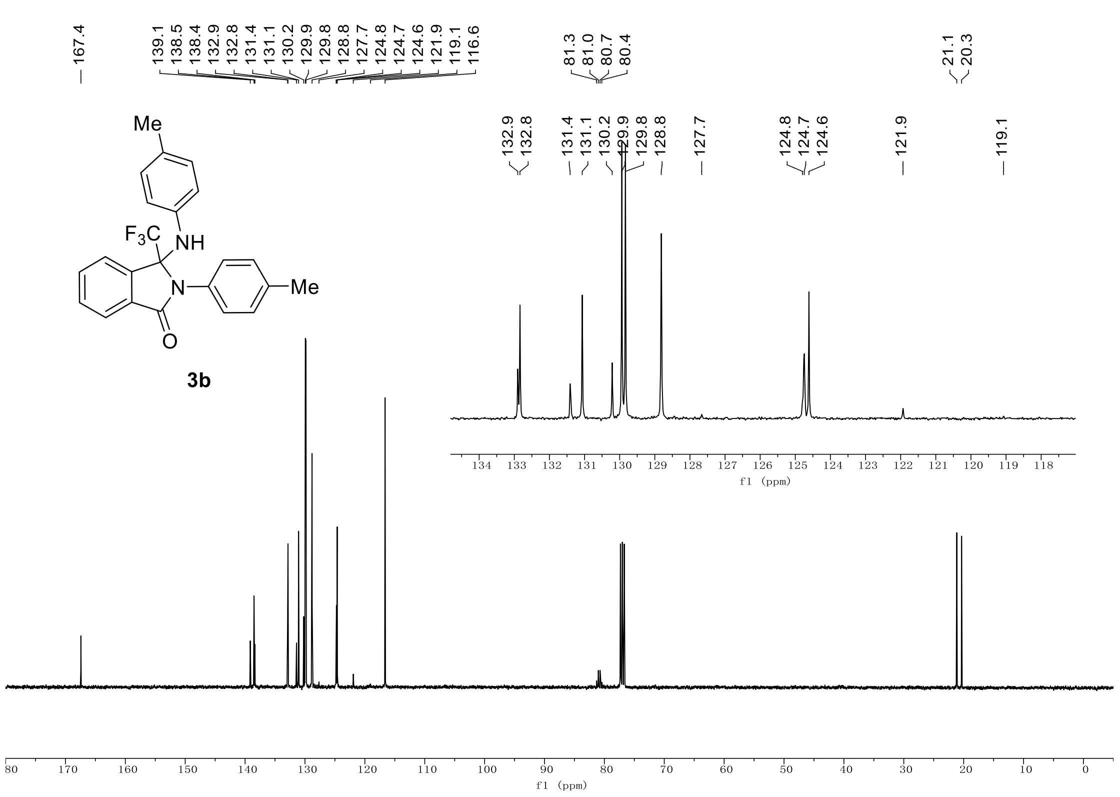

Figure S125. ^{13}C NMR (101 MHz, $CDCl_3$) spectrum of compound 3b, related to Scheme 2

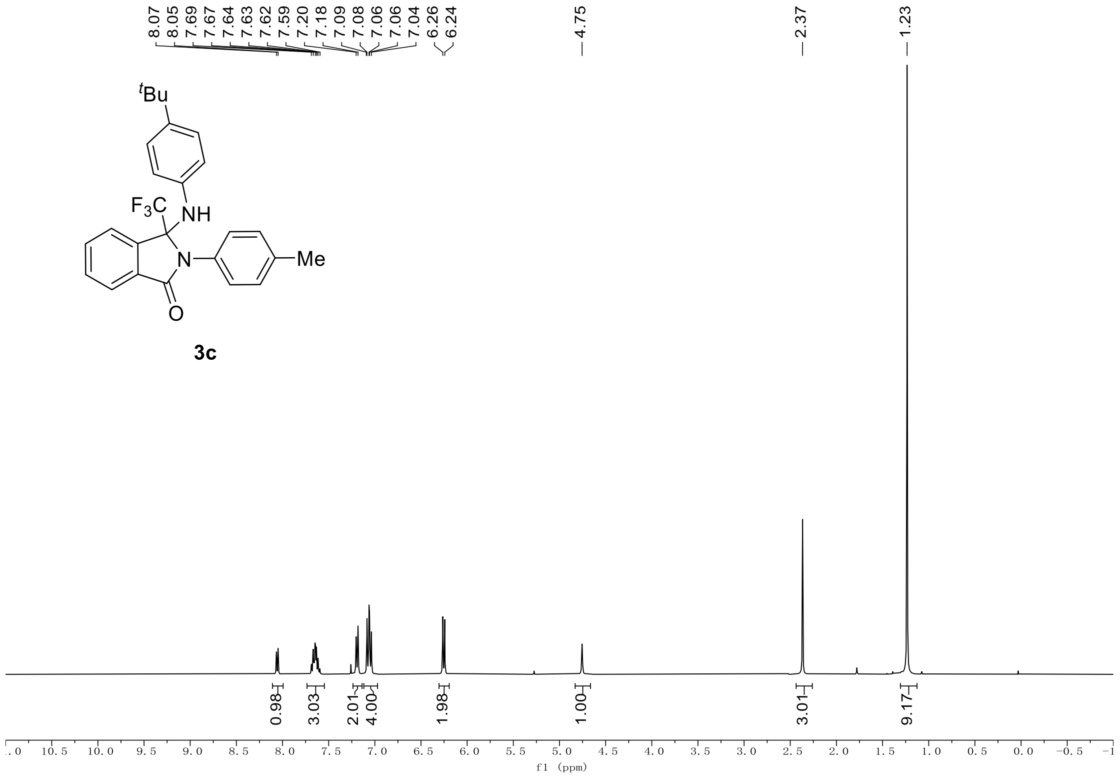

Figure S126. ^{1}H NMR (400 MHz, $CDCl_3$) spectrum of compound 3c, related to Scheme 2

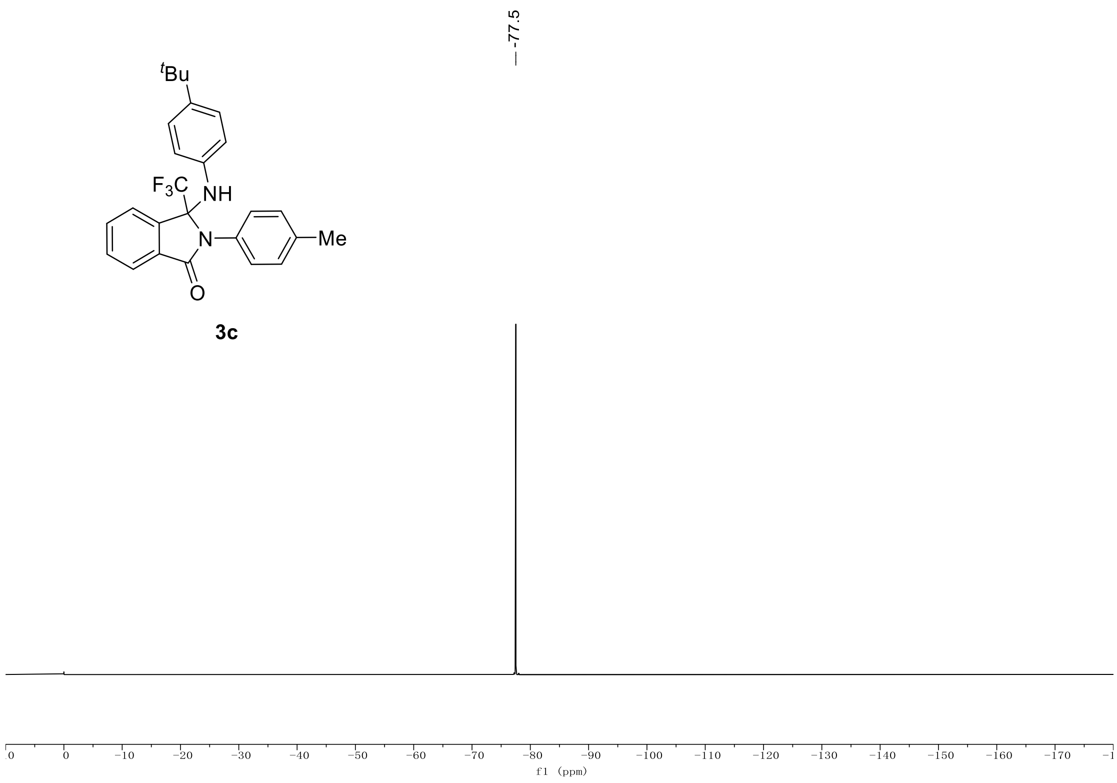

Figure S127. ^{19}F NMR (376 MHz, CDCl$_3$) spectrum of compound 3c, related to Scheme 2

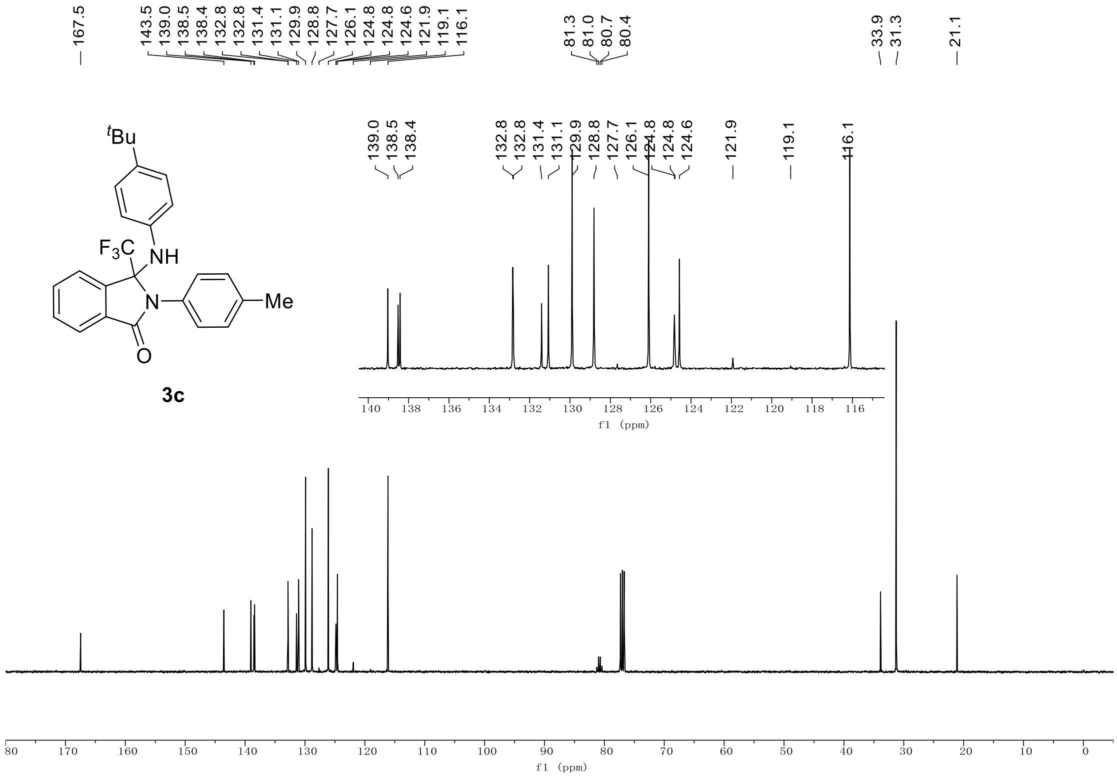

Figure S128. ^{13}C NMR (101 MHz, CDCl$_3$) spectrum of compound 3c, related to Scheme 2

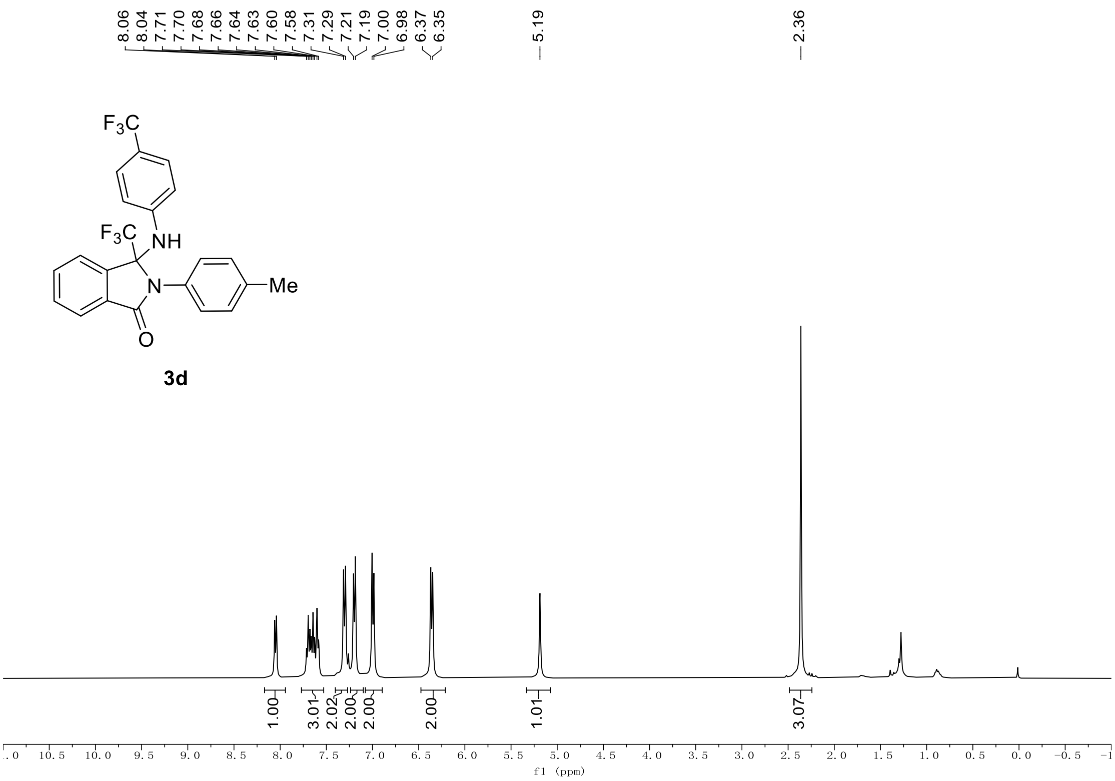

Figure S129. ^{1}H NMR (400 MHz, $CDCl_3$) spectrum of compound 3d, related to Scheme 2

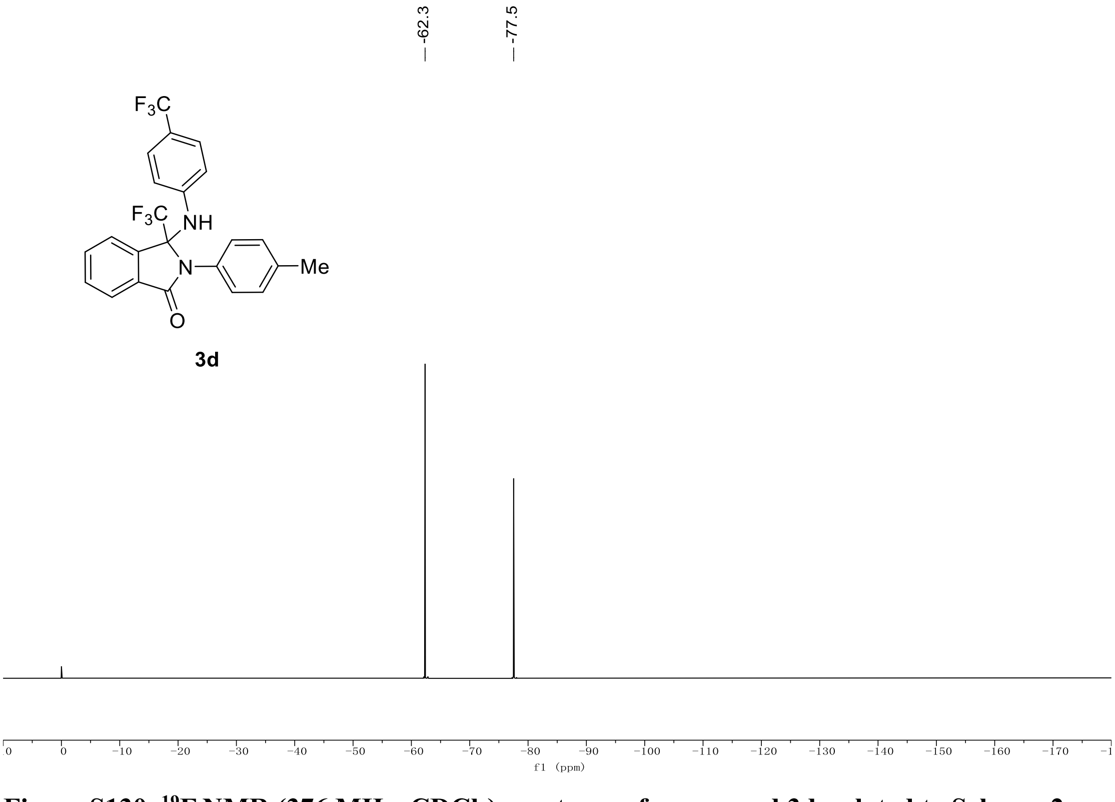

Figure S130. ^{19}F NMR (376 MHz, $CDCl_3$) spectrum of compound 3d, related to Scheme 2

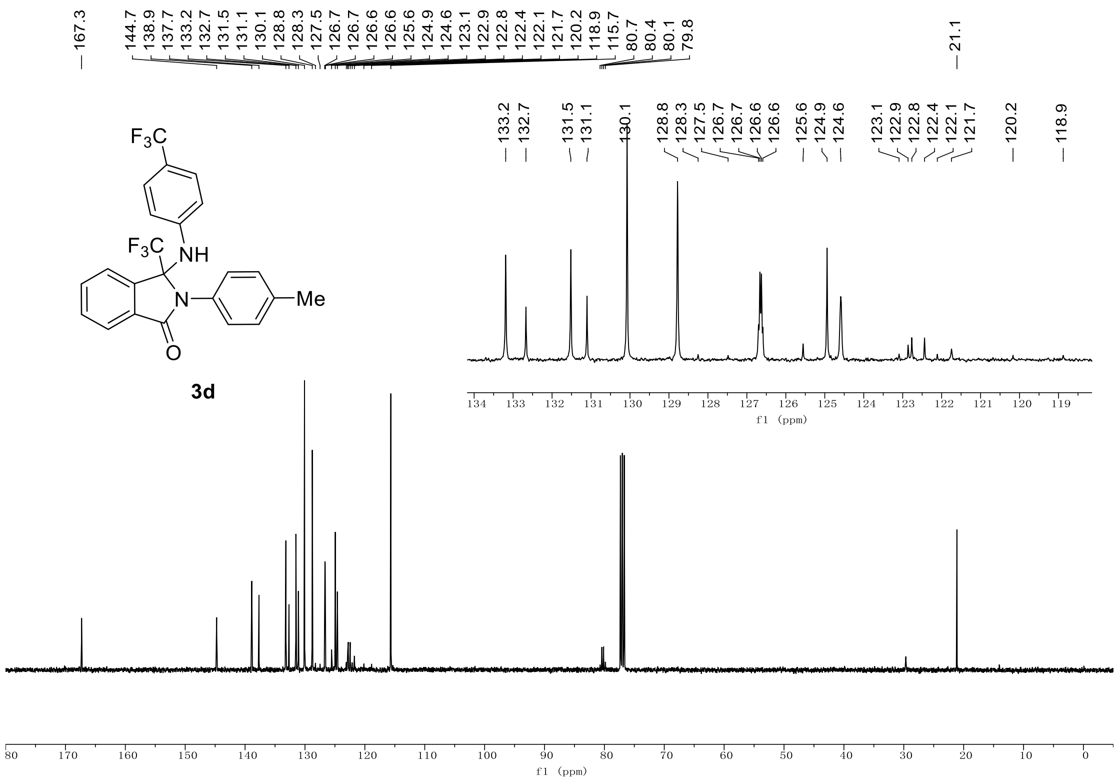

Figure S131. ^{13}C NMR (101 MHz, $CDCl_3$) spectrum of compound 3d, related to Scheme 2

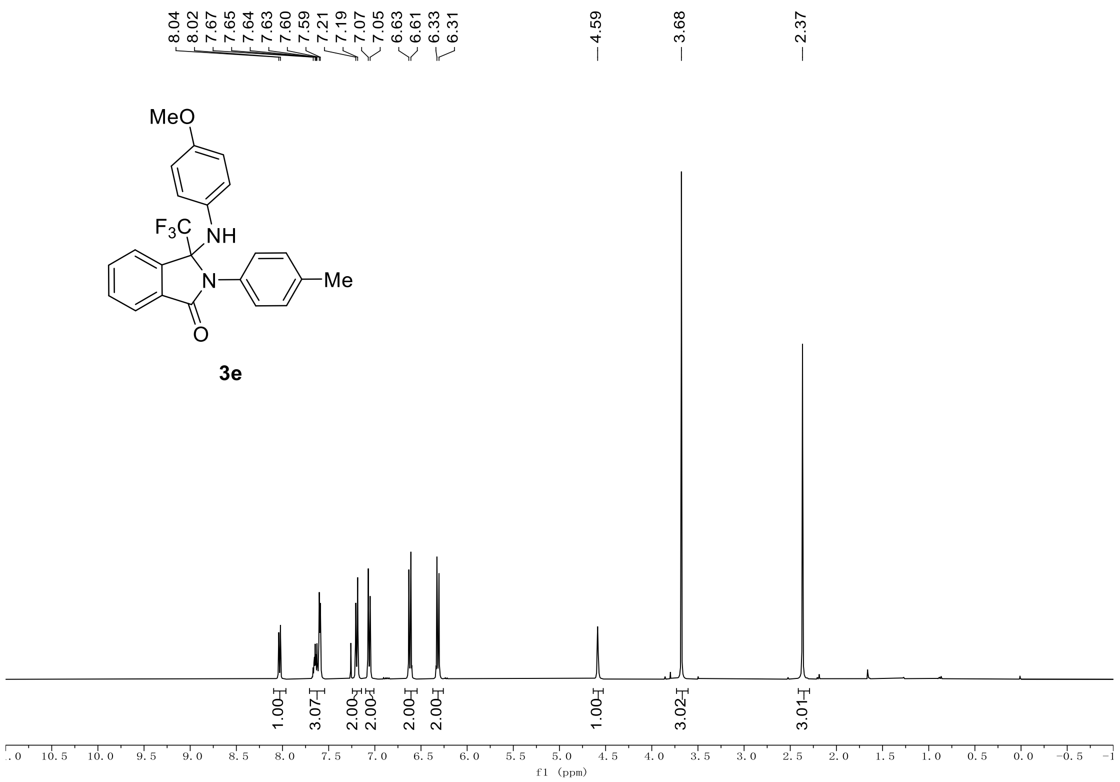

Figure S132. ^{1}H NMR (400 MHz, $CDCl_3$) spectrum of compound 3e, related to Scheme 2

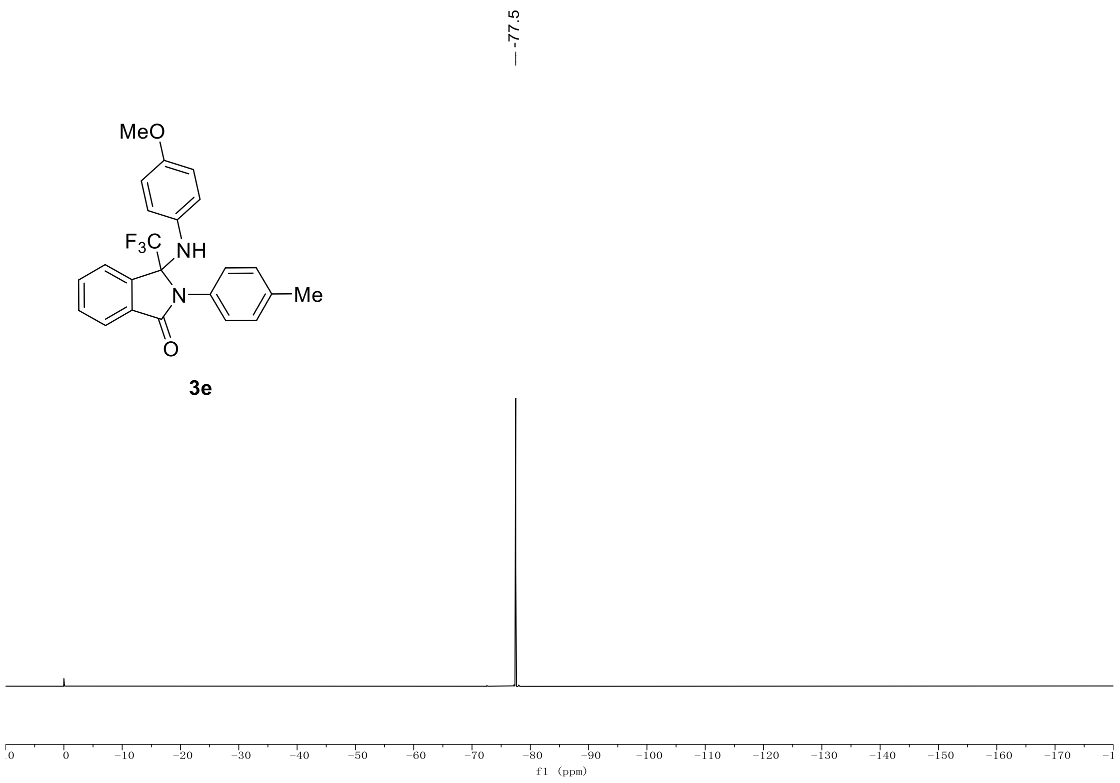

Figure S133. ^{19}F NMR (376 MHz, $CDCl_3$) spectrum of compound 3e, related to Scheme 2

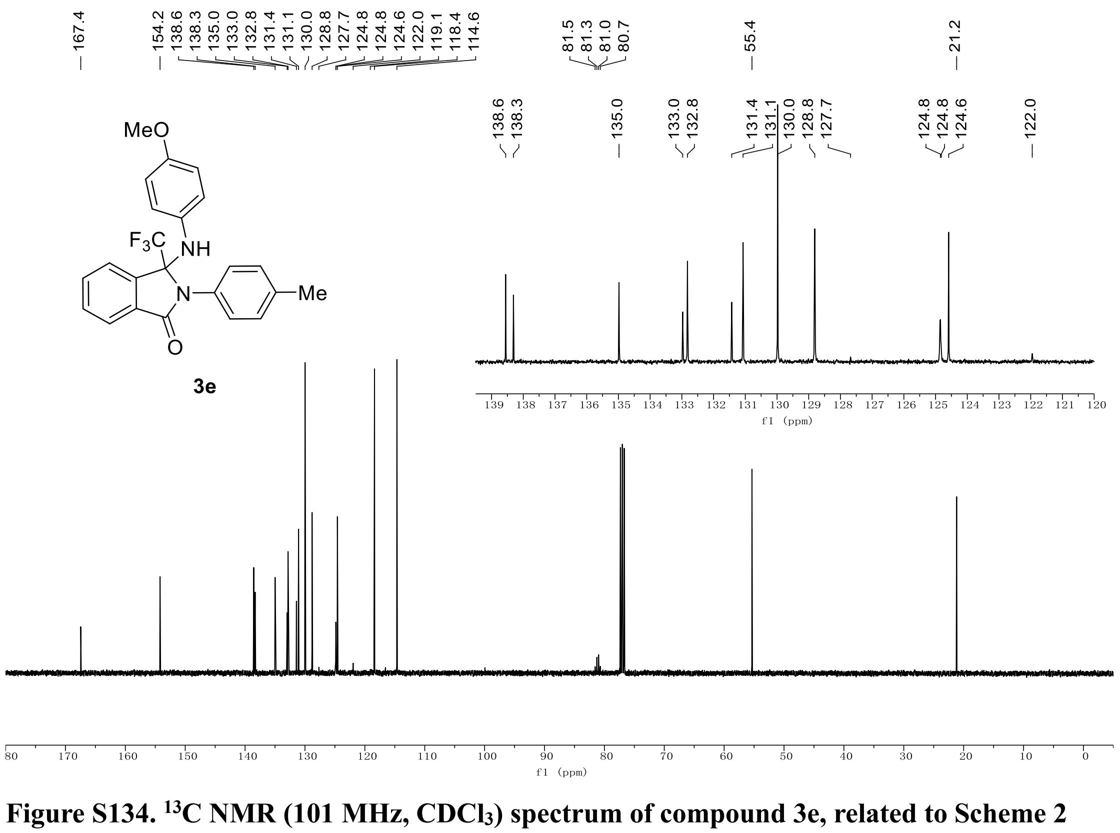

Figure S134. ^{13}C NMR (101 MHz, $CDCl_3$) spectrum of compound 3e, related to Scheme 2

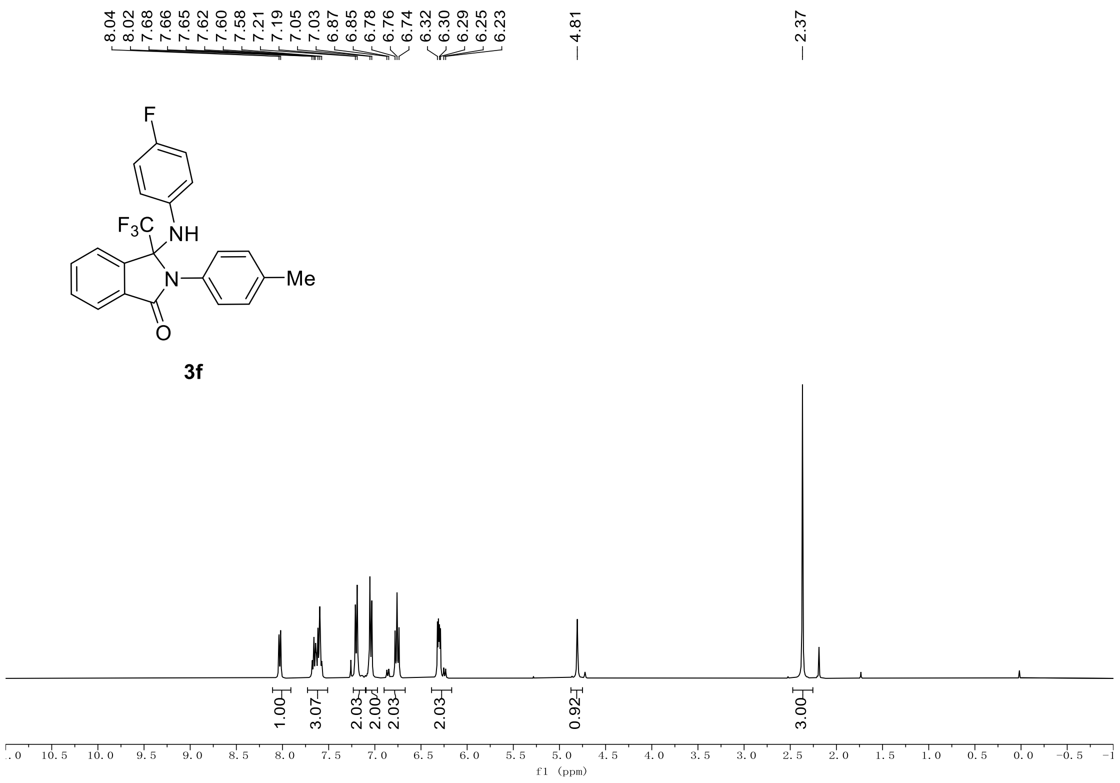

Figure S135. ^1H NMR (400 MHz, $CDCl_3$) spectrum of compound 3f, related to Scheme 2

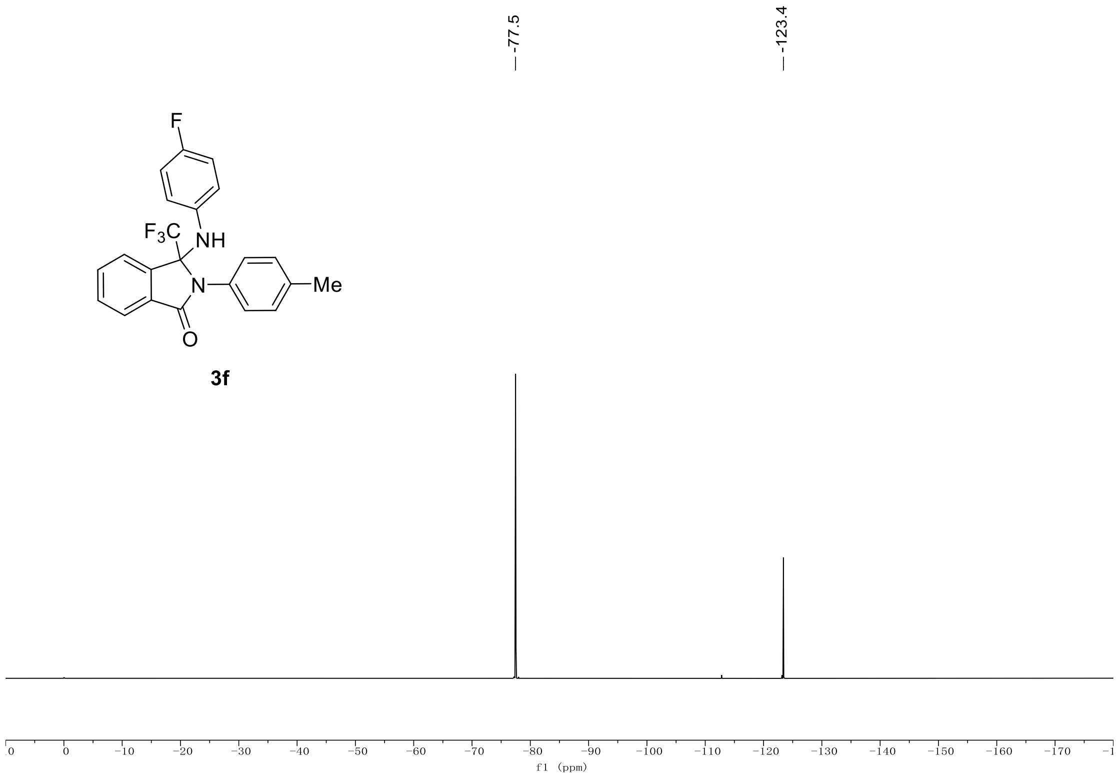

Figure S136. ^{19}F NMR (376 MHz, $CDCl_3$) spectrum of compound 3f, related to Scheme 2

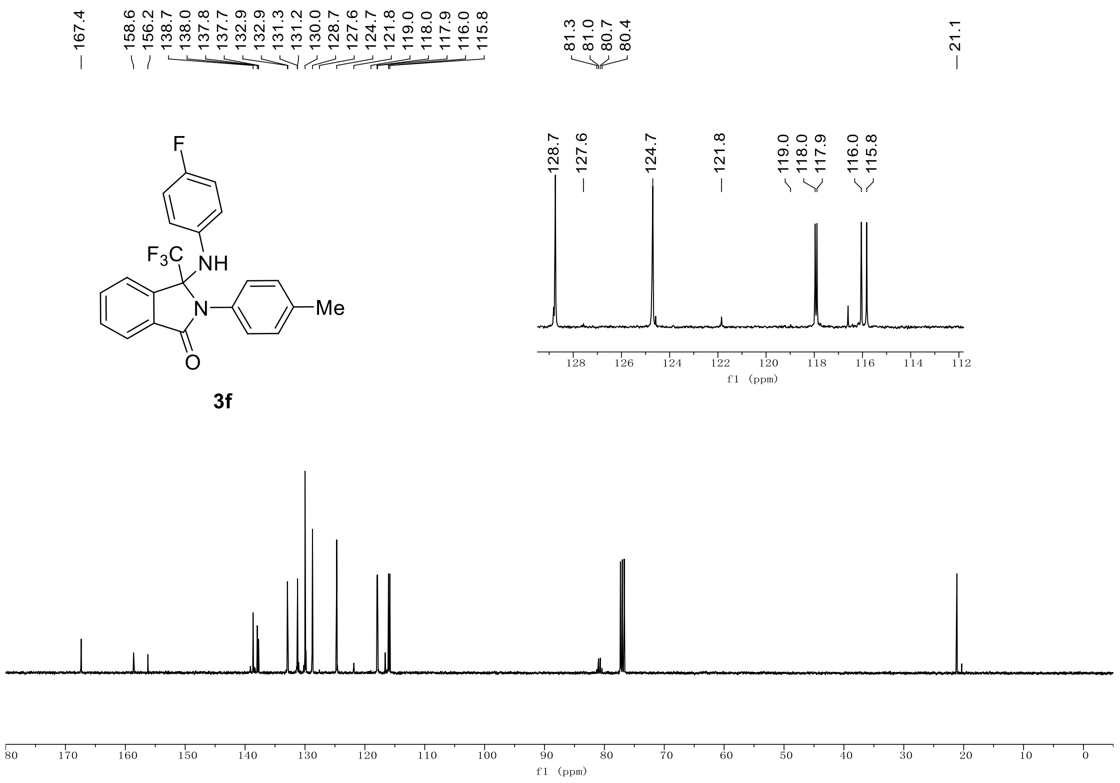

Figure S137. ^{13}C NMR (101 MHz, $CDCl_3$) spectrum of compound 3f, related to Scheme 2

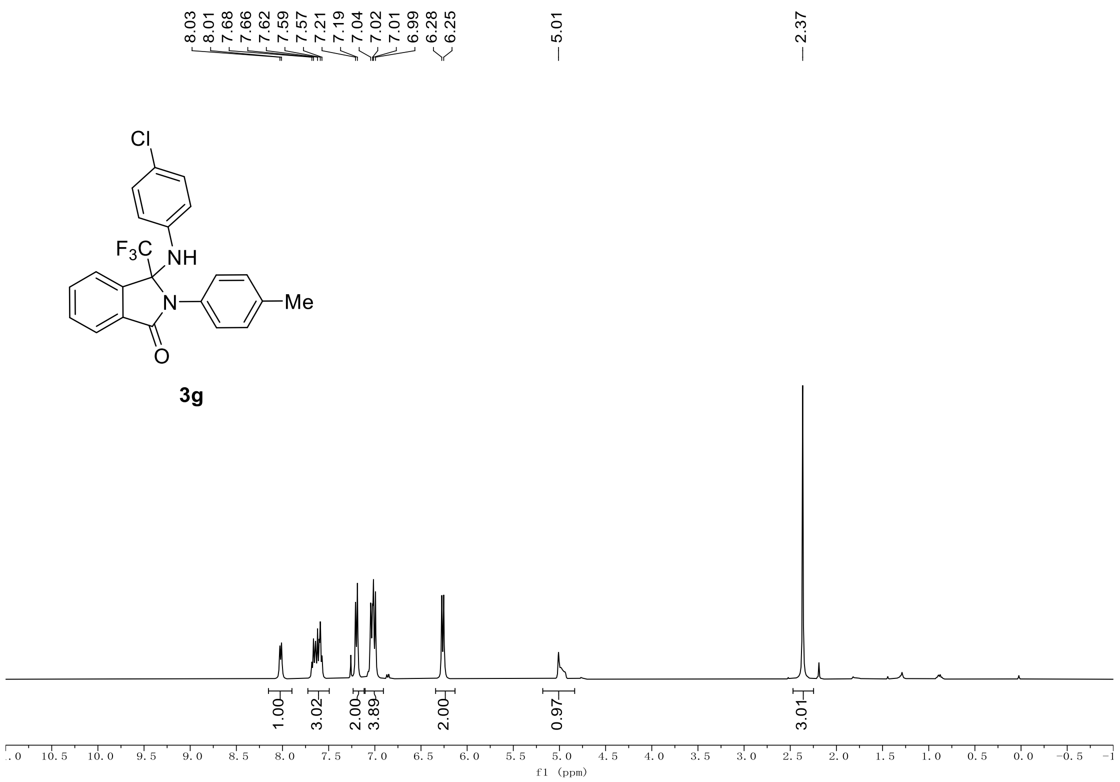

Figure S138. ^{1}H NMR (400 MHz, $CDCl_3$) spectrum of compound 3g, related to Scheme 2

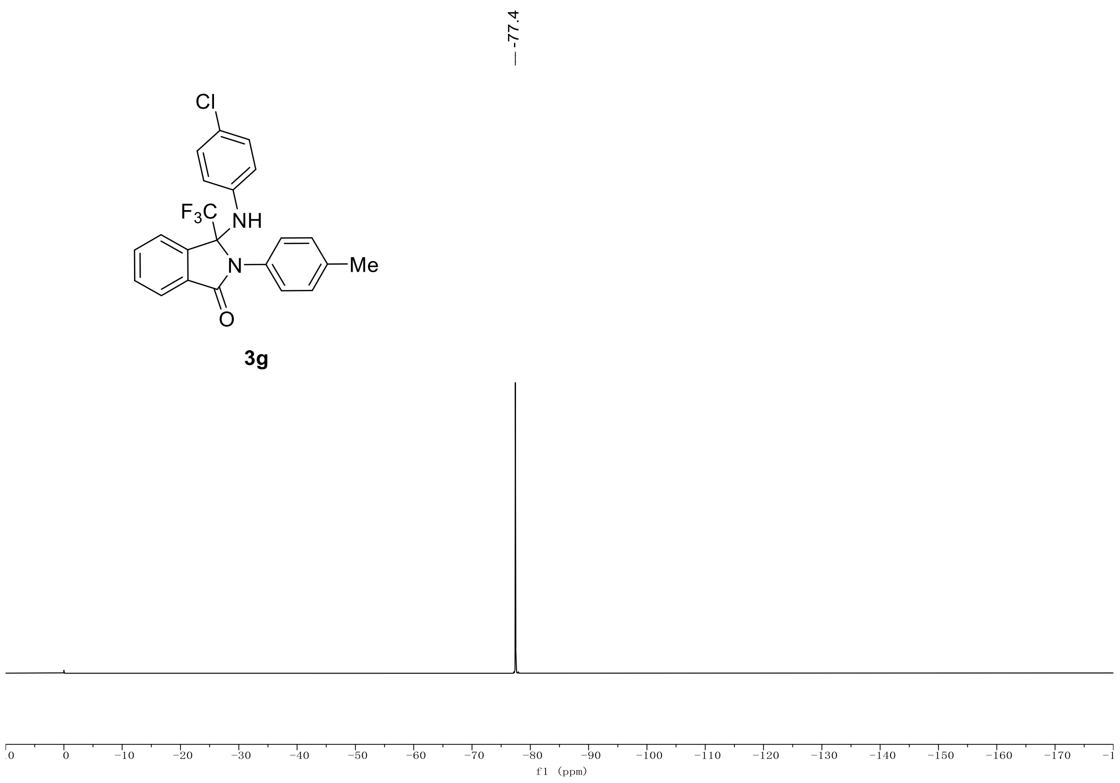

Figure S139. ^{19}F NMR (376 MHz, CDCl$_3$) spectrum of compound 3g, related to Scheme 2

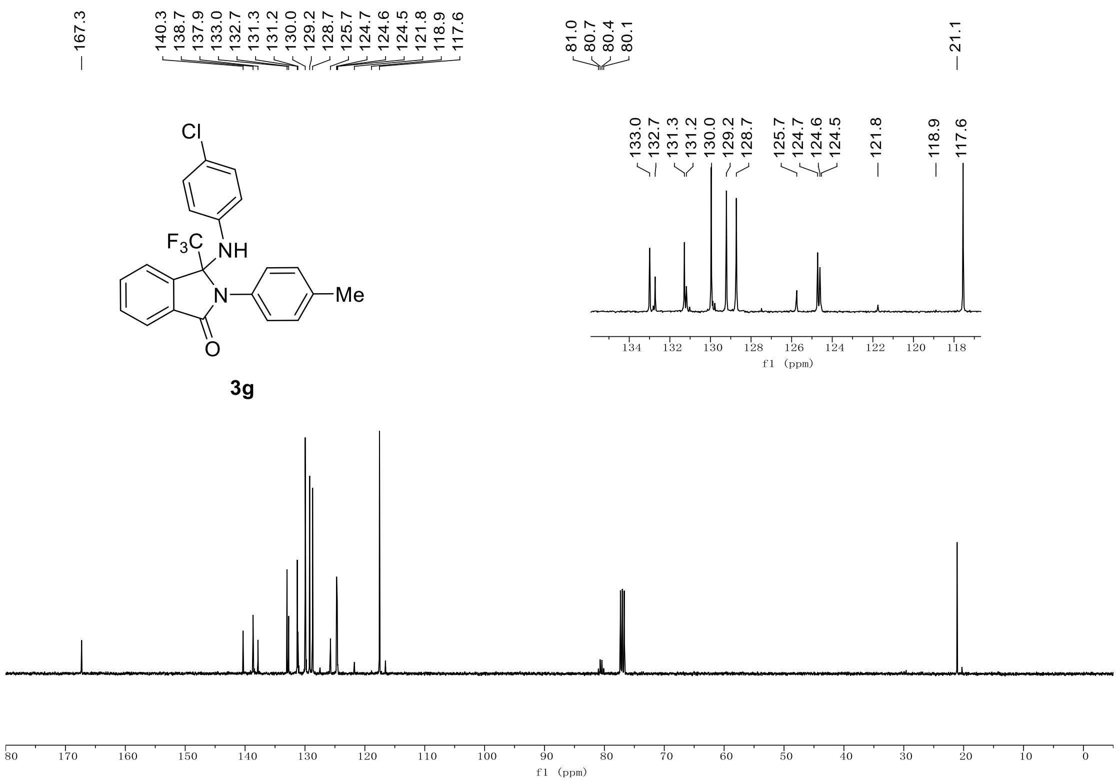

Figure S140. ^{13}C NMR (101 MHz, CDCl$_3$) spectrum of compound 3g, related to Scheme 2

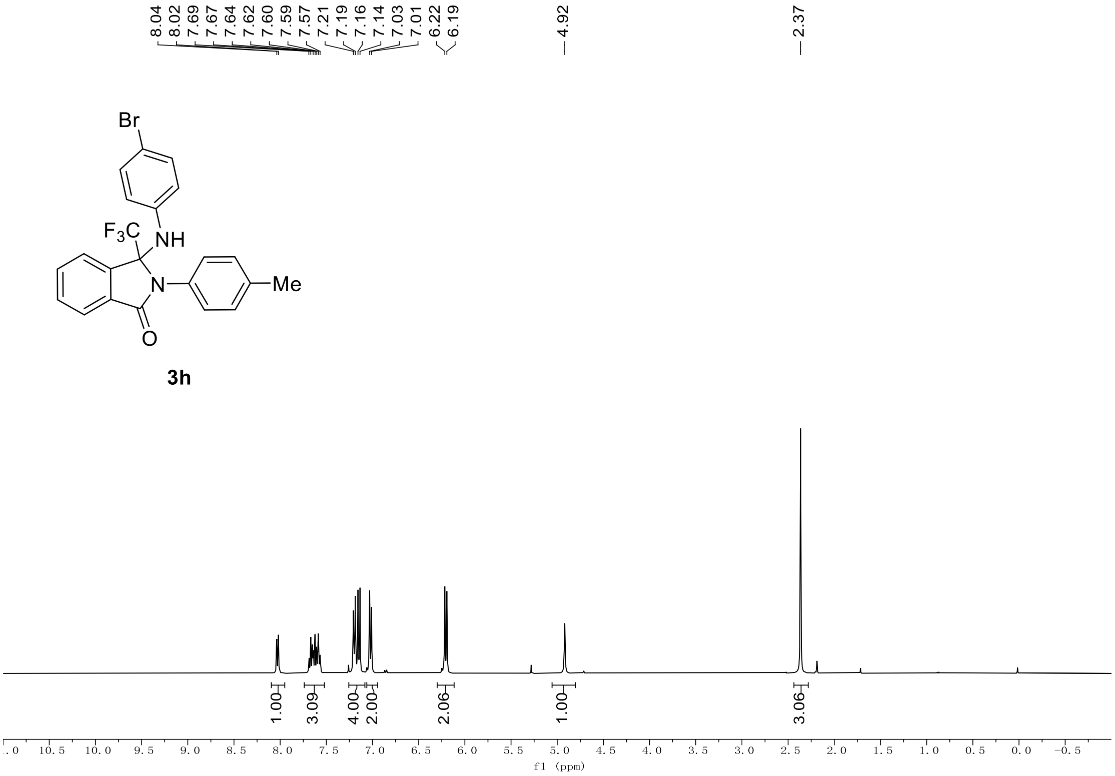

Figure S141. ^{1}H NMR (400 MHz, $CDCl_3$) spectrum of compound 3h, related to Scheme 2

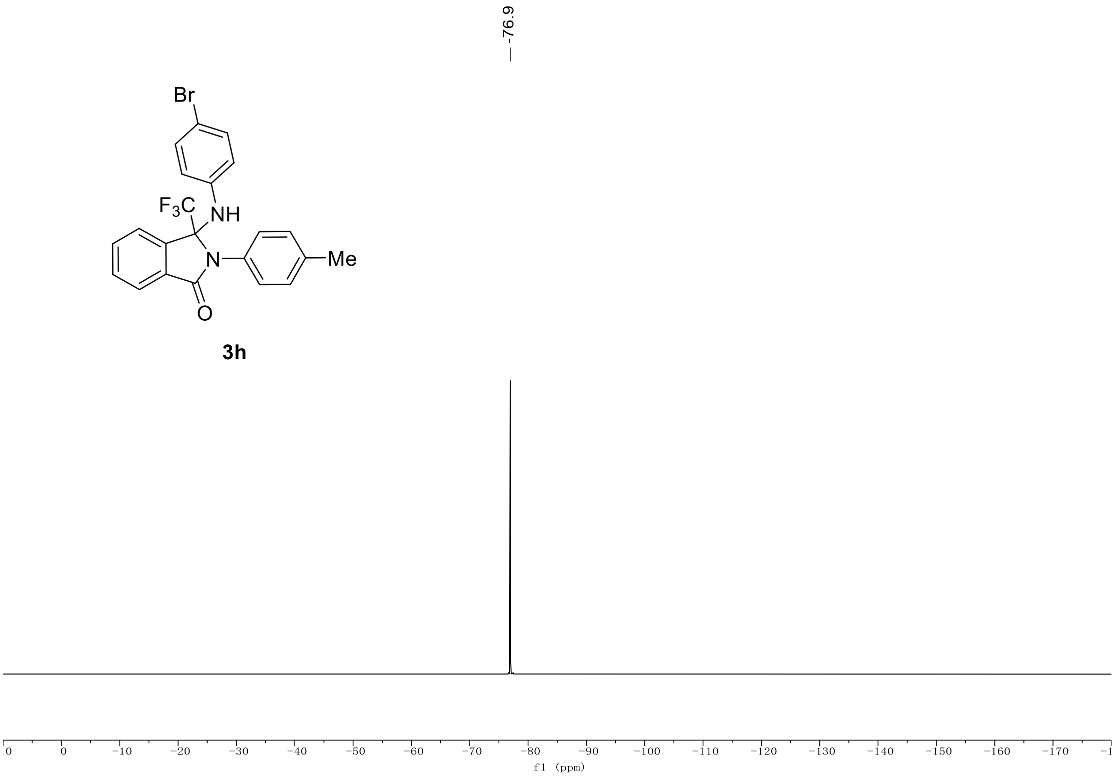

Figure S142. ^{19}F NMR (376 MHz, $CDCl_3$) spectrum of compound 3h, related to Scheme 2

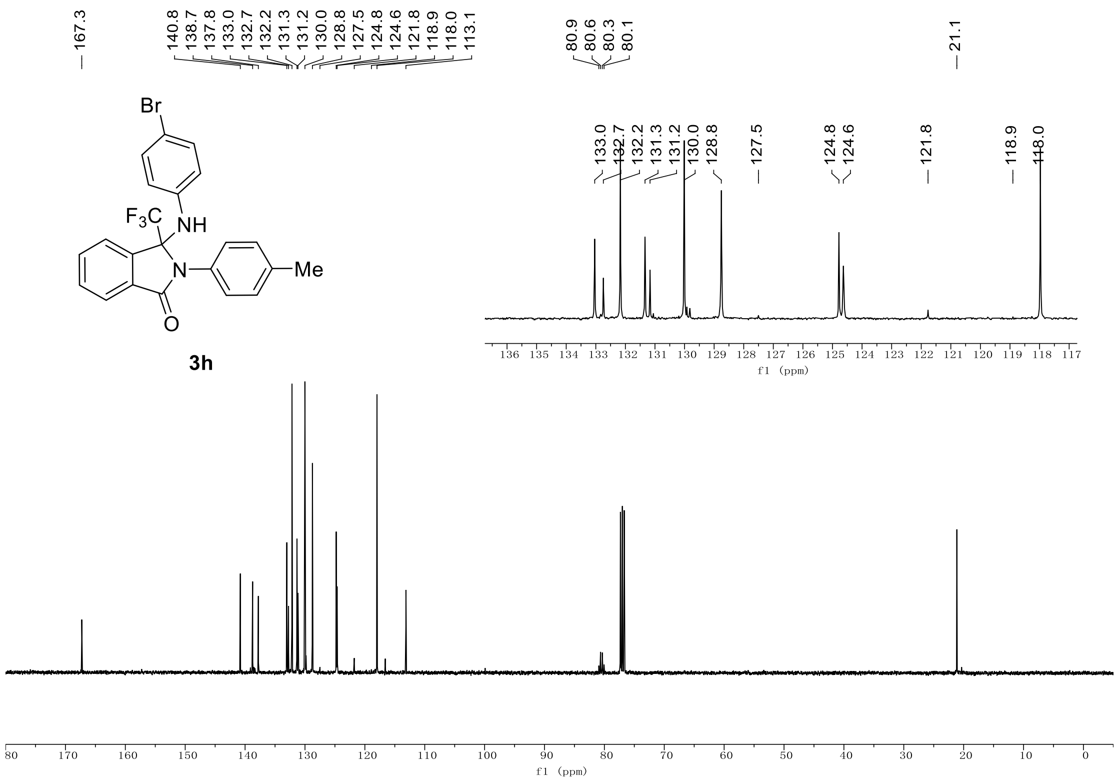

Figure S143. ^{13}C NMR (101 MHz, $CDCl_3$) spectrum of compound 3h, related to Scheme 2

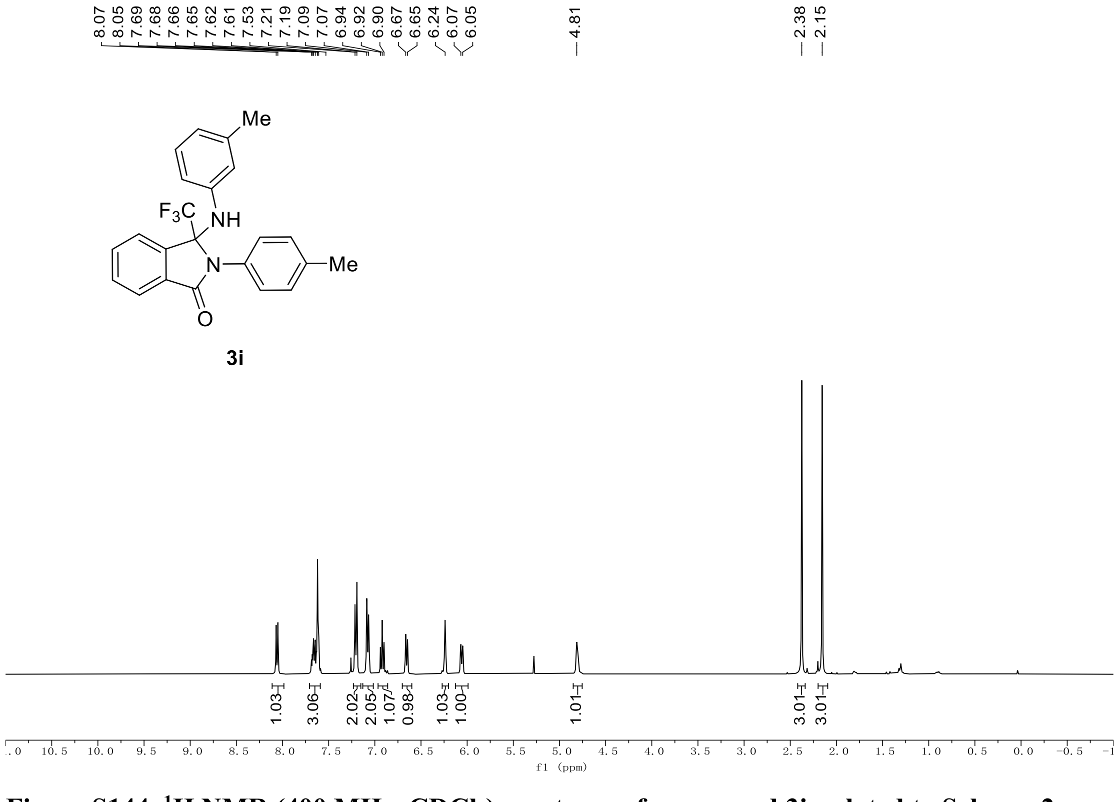

Figure S144. ^{1}H NMR (400 MHz, $CDCl_3$) spectrum of compound 3i, related to Scheme 2

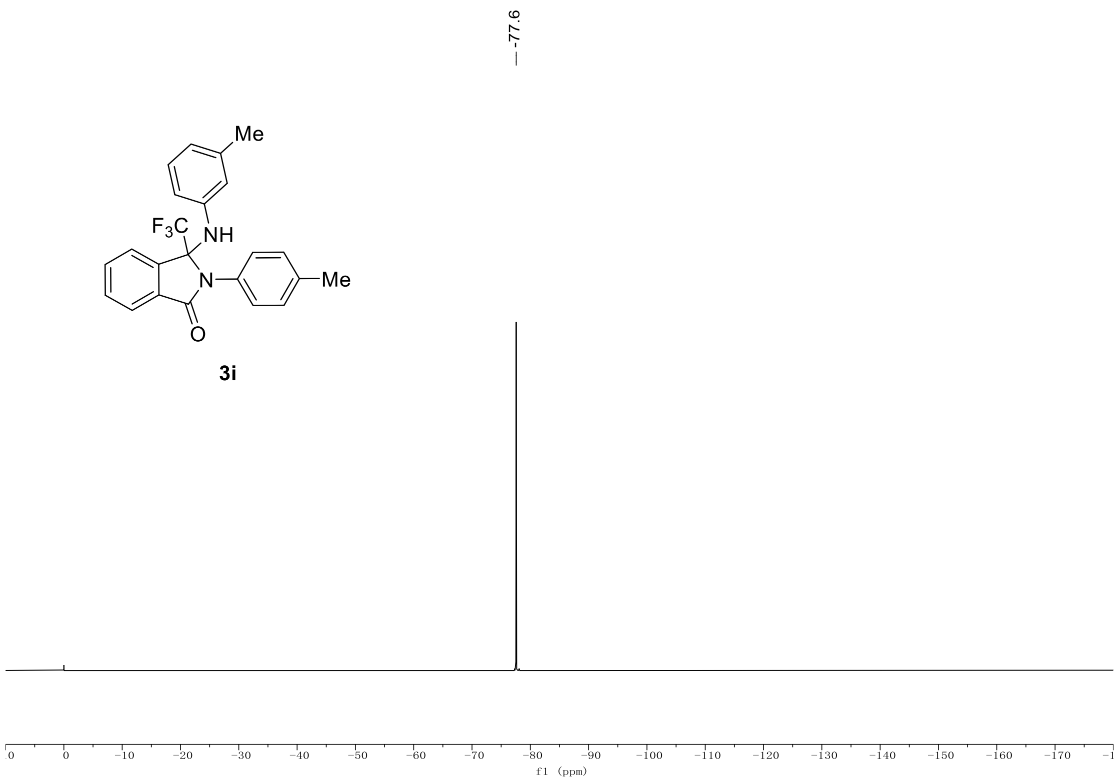

Figure S145. ^{19}F NMR (376 MHz, CDCl$_3$) spectrum of compound 3i, related to Scheme 2

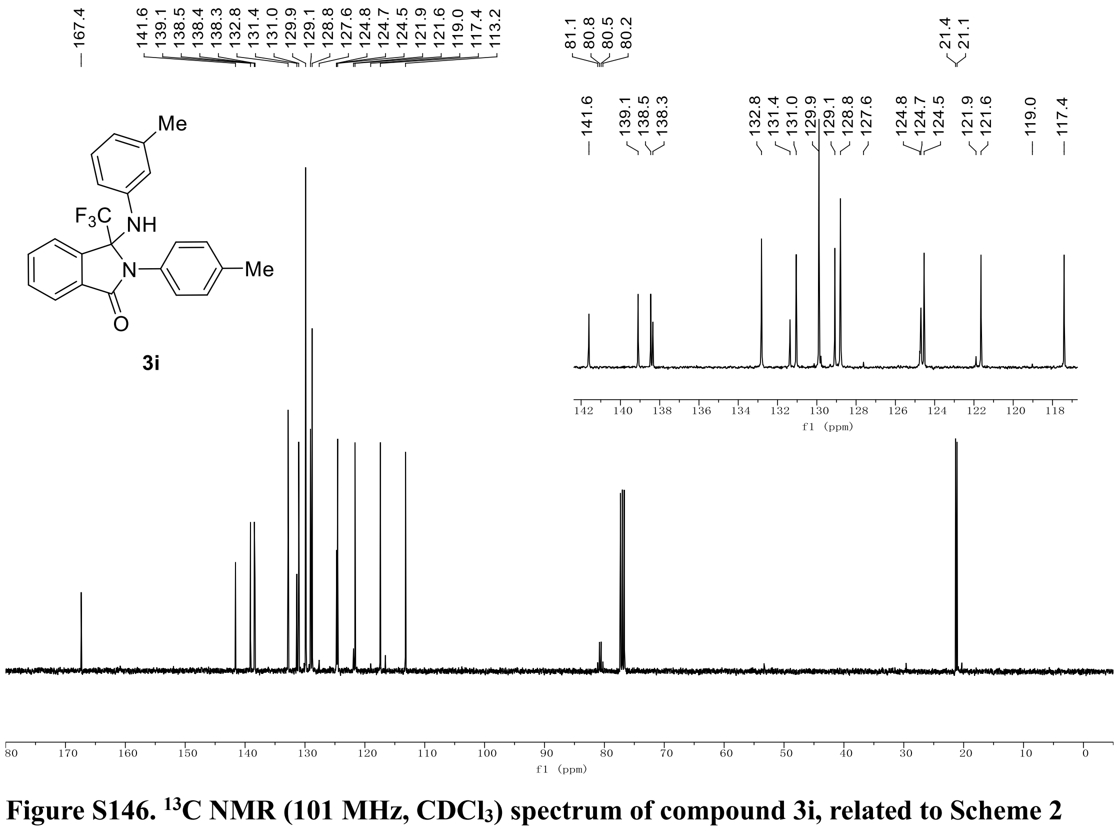

Figure S146. ^{13}C NMR (101 MHz, CDCl$_3$) spectrum of compound 3i, related to Scheme 2

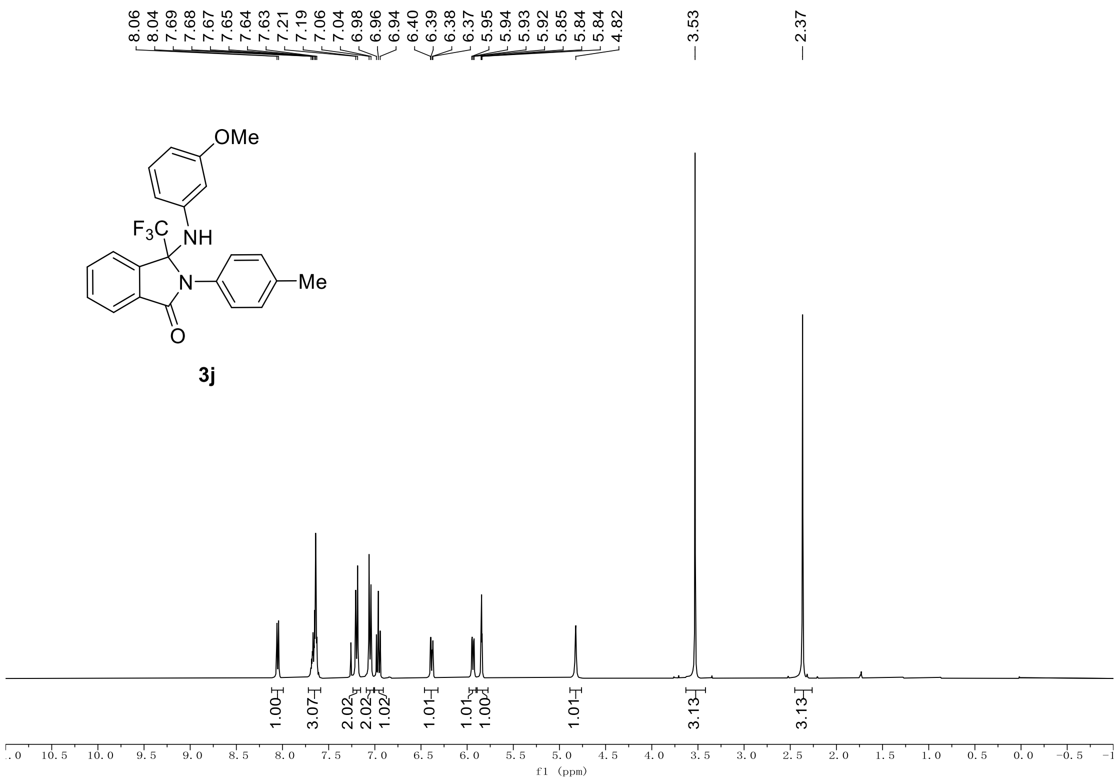

Figure S147. ^{1}H NMR (400 MHz, $CDCl_3$) spectrum of compound 3j, related to Scheme 2

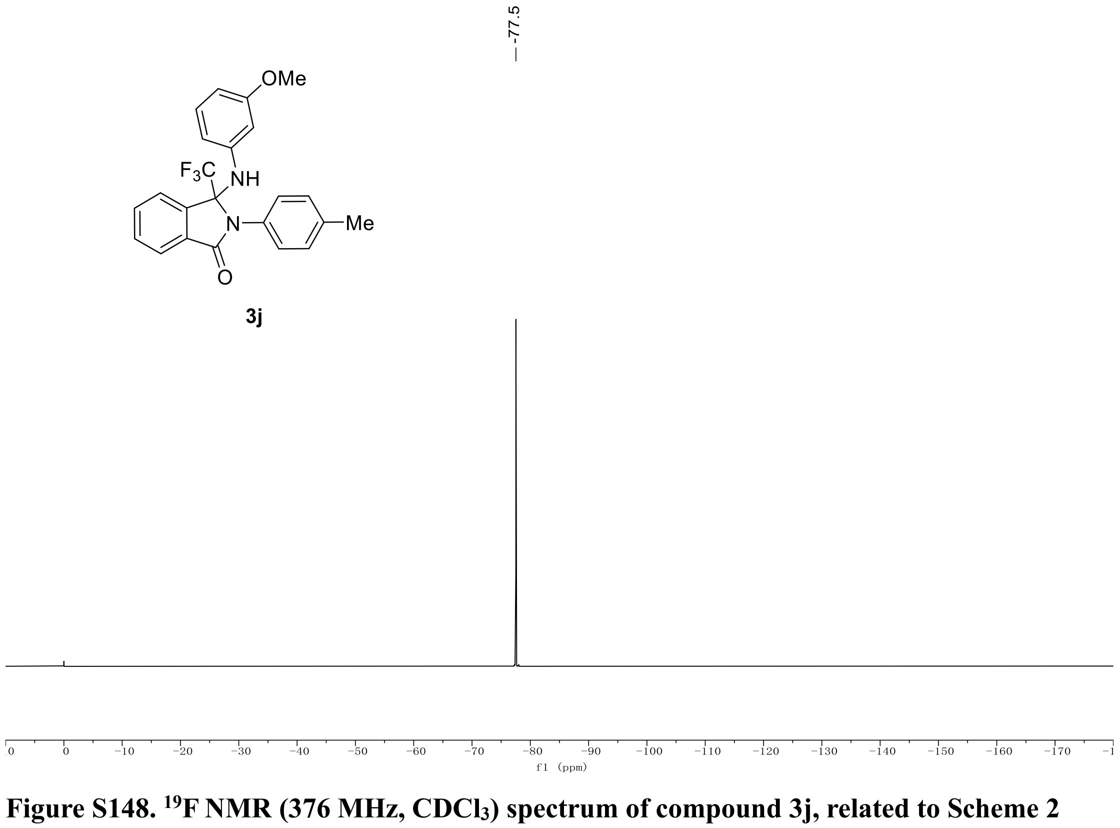

Figure S148. ^{19}F NMR (376 MHz, $CDCl_3$) spectrum of compound 3j, related to Scheme 2

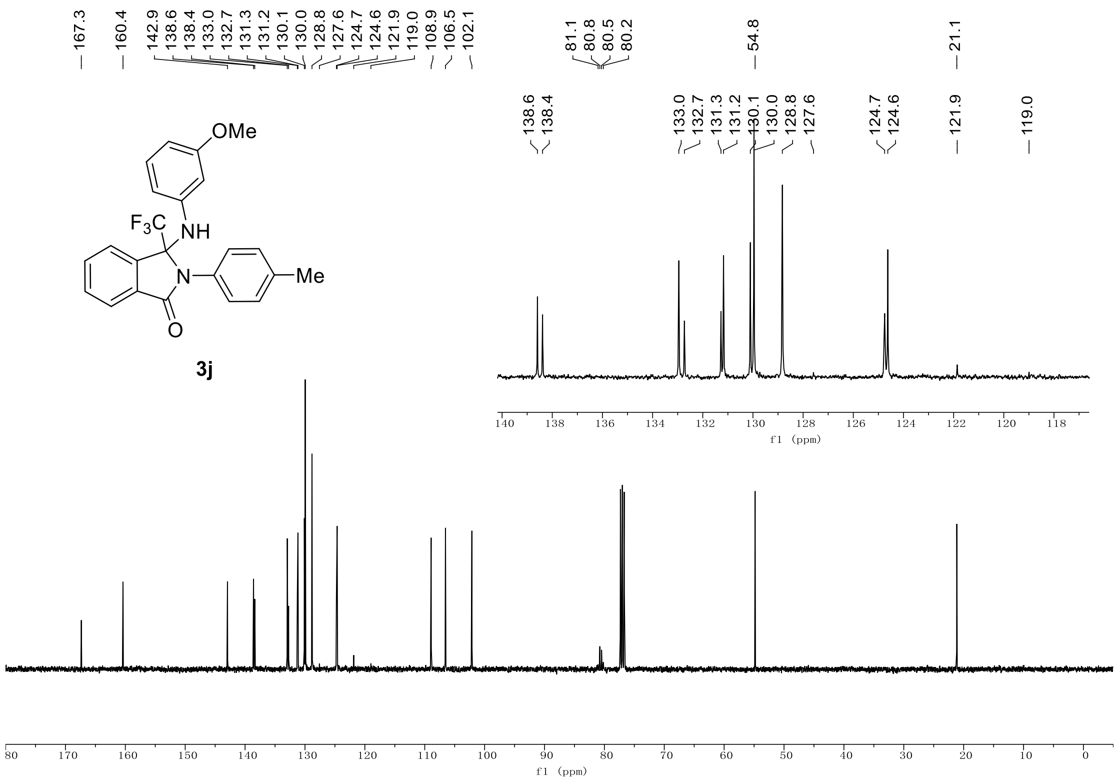

Figure S149. ^{13}C NMR (101 MHz, $CDCl_3$) spectrum of compound 3j, related to Scheme 2

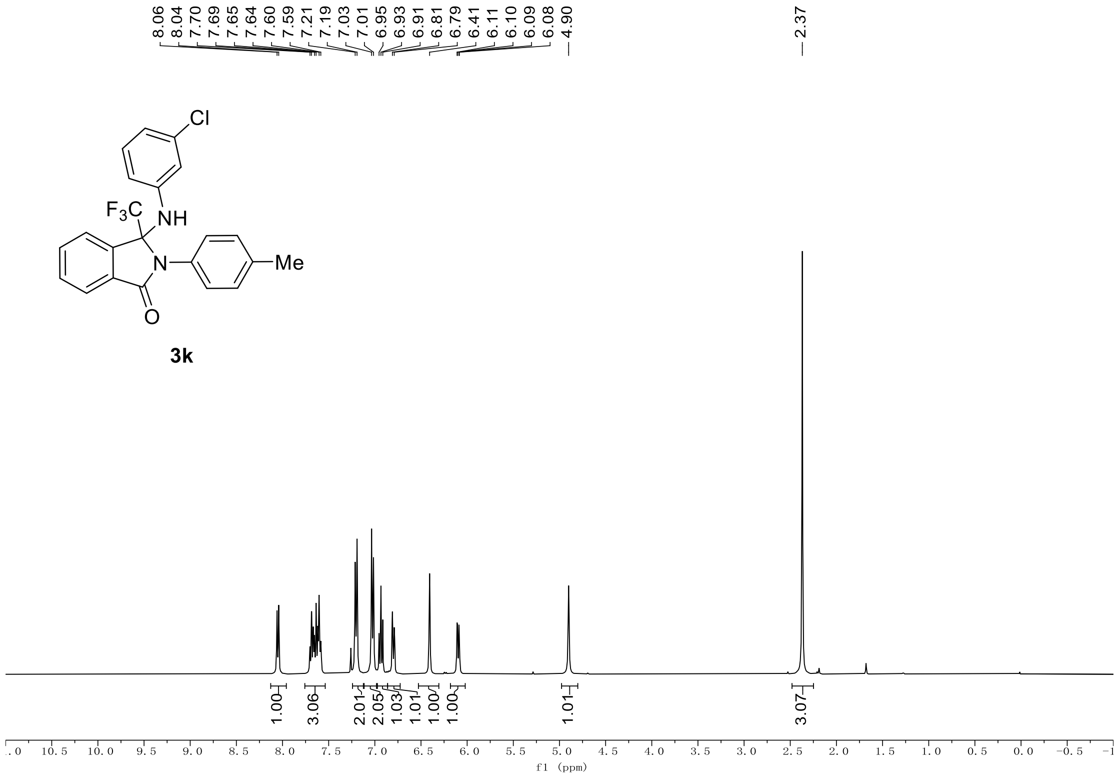

Figure S150. ^{1}H NMR (400 MHz, $CDCl_3$) spectrum of compound 3k, related to Scheme 2

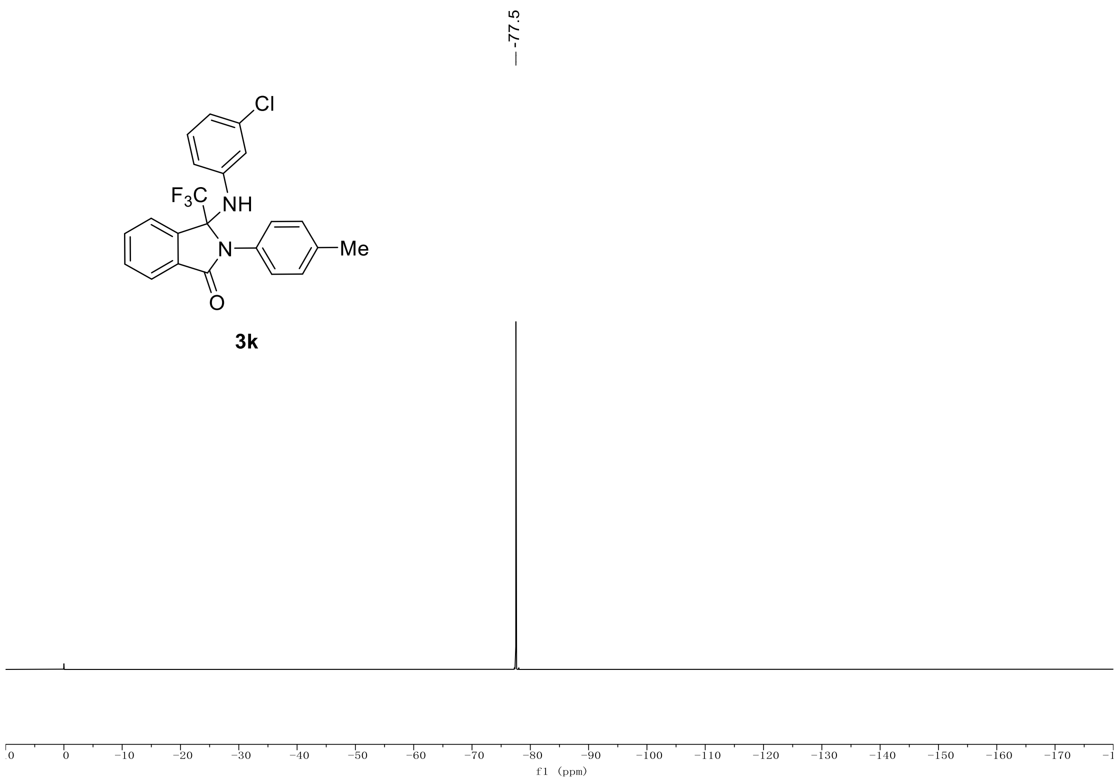

Figure S151. ^{19}F NMR (376 MHz, $CDCl_3$) spectrum of compound 3k, related to Scheme 2

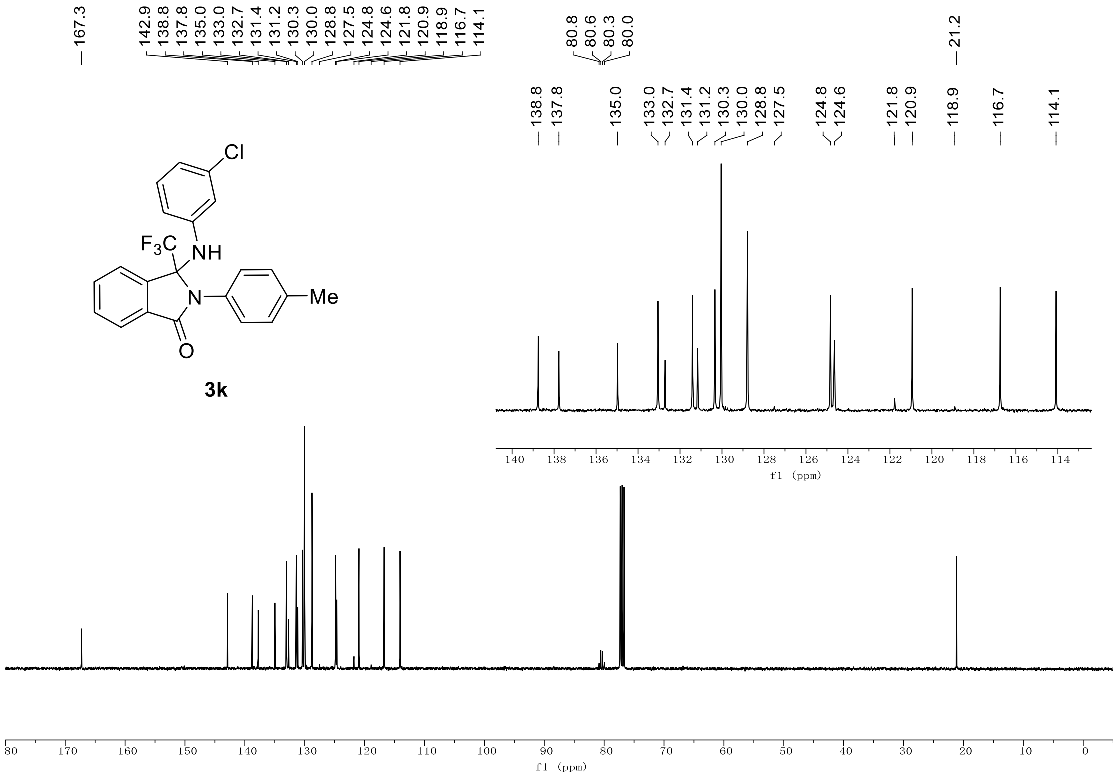

Figure S152. ^{13}C NMR (101 MHz, $CDCl_3$) spectrum of compound 3k, related to Scheme 2

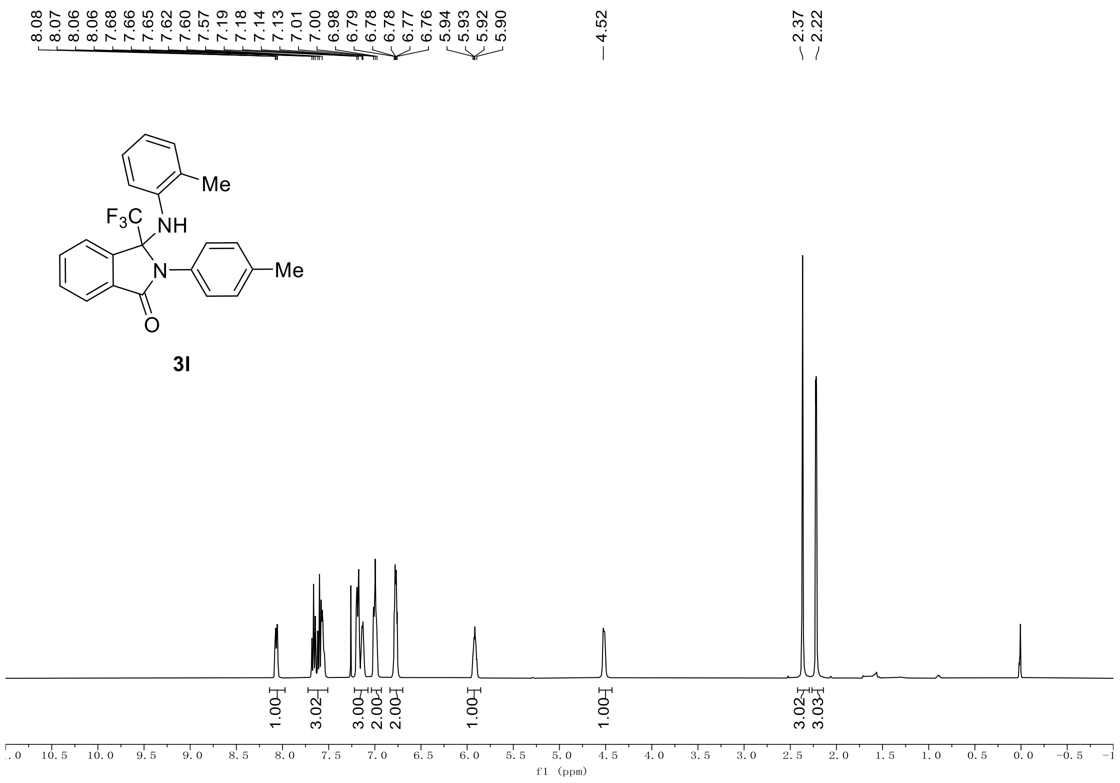

Figure S153. ^{1}H NMR (400 MHz, $CDCl_3$) spectrum of compound 3l, related to Scheme 2

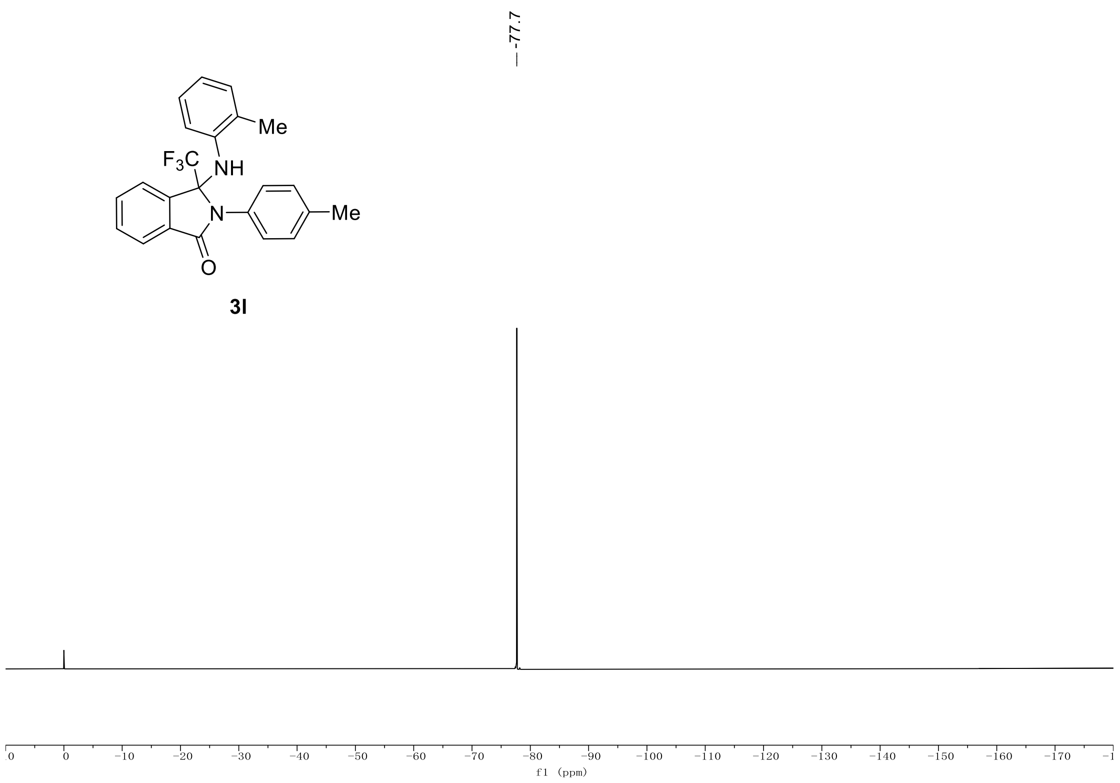

Figure S154. ^{19}F NMR (376 MHz, $CDCl_3$) spectrum of compound 3l, related to Scheme 2

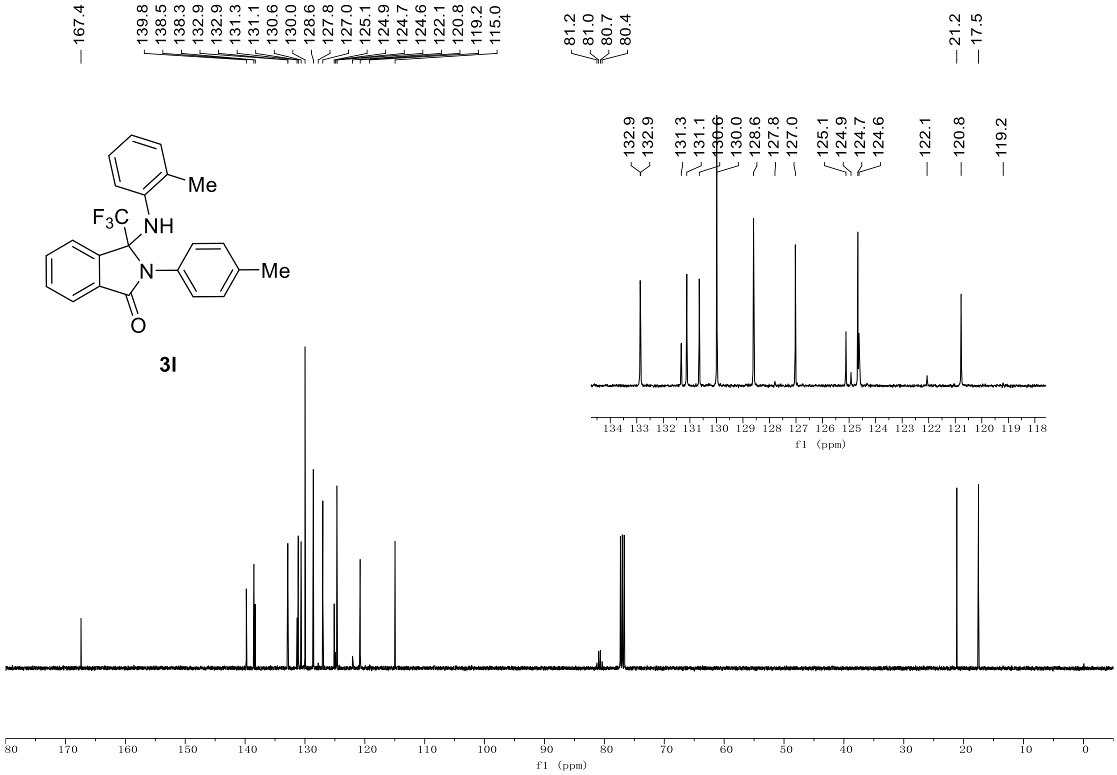

Figure S155. ^{13}C NMR (101 MHz, $CDCl_3$) spectrum of compound 3l, related to Scheme 2

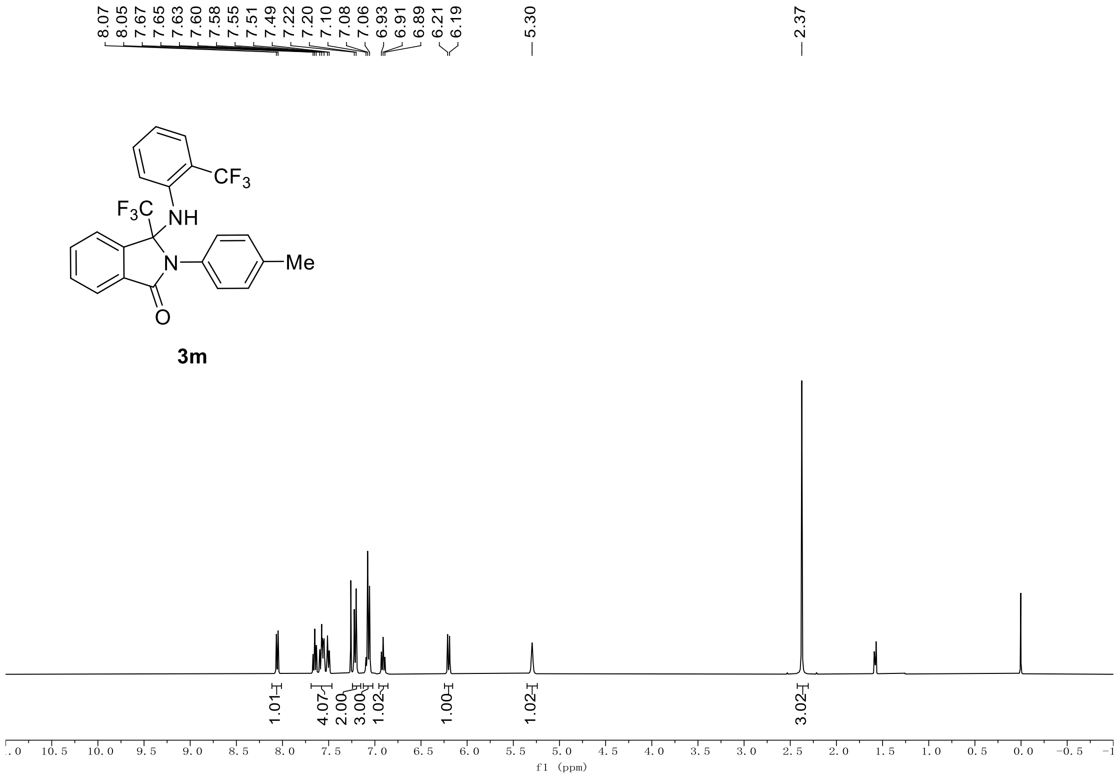

Figure S156. ^{1}H NMR (400 MHz, $CDCl_3$) spectrum of compound 3m, related to Scheme 2

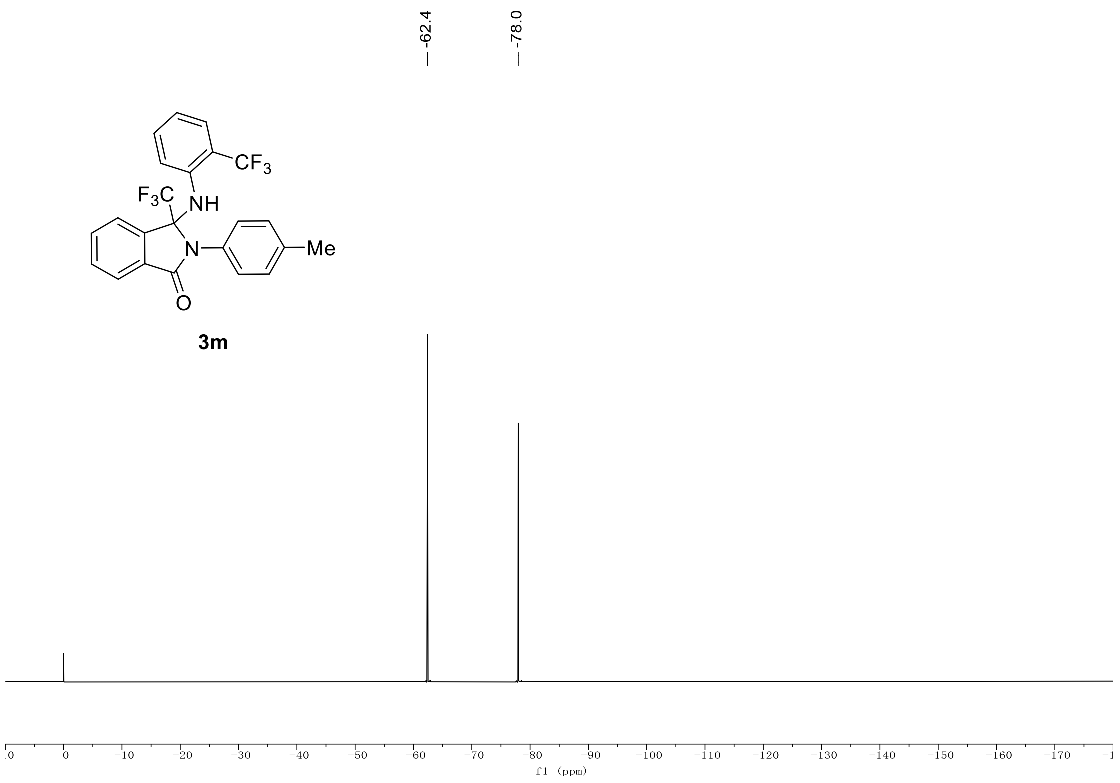

Figure S157. ^{19}F NMR (376 MHz, $CDCl_3$) spectrum of compound 3m, related to Scheme 2

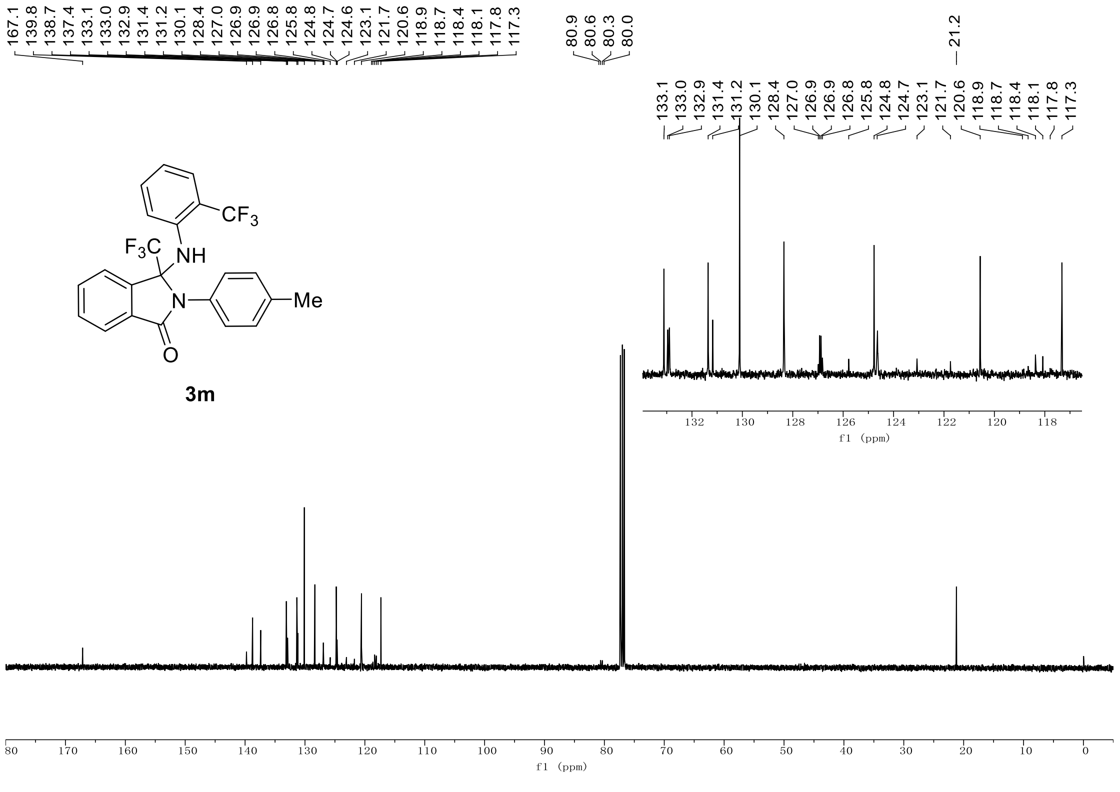

Figure S158. ^{13}C NMR (101 MHz, $CDCl_3$) spectrum of compound 3m, related to Scheme 2

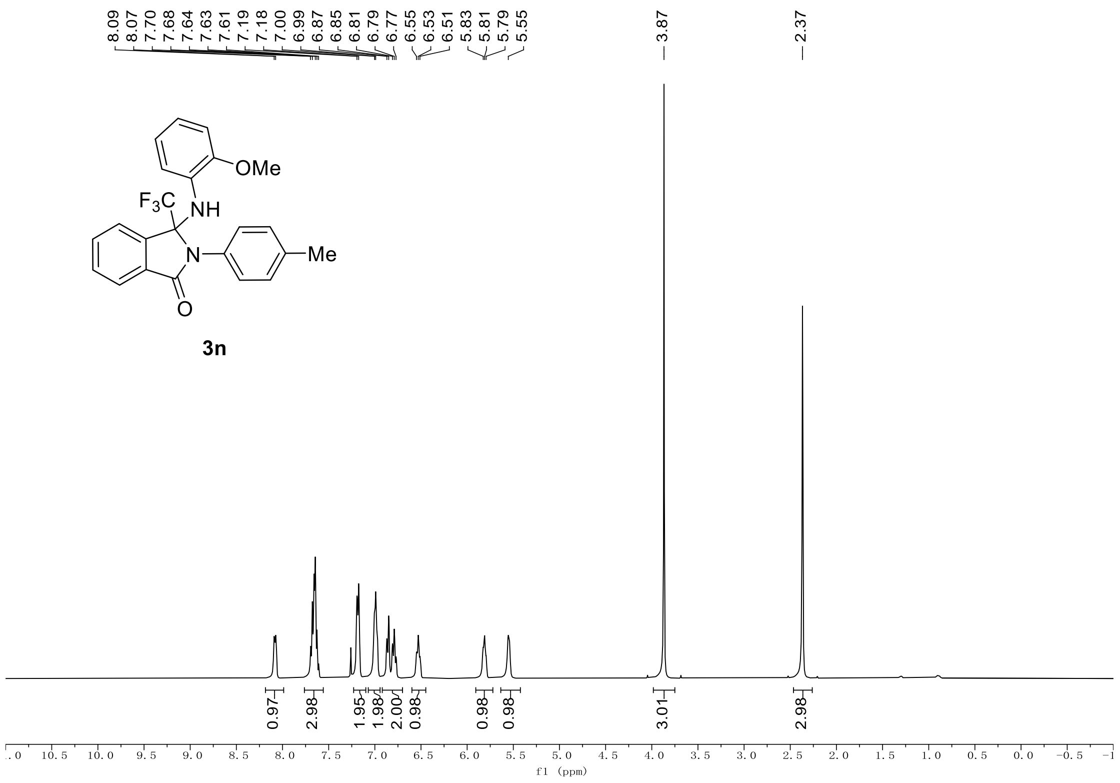

Figure S159. ^{1}H NMR (400 MHz, $CDCl_3$) spectrum of compound 3n, related to Scheme 2

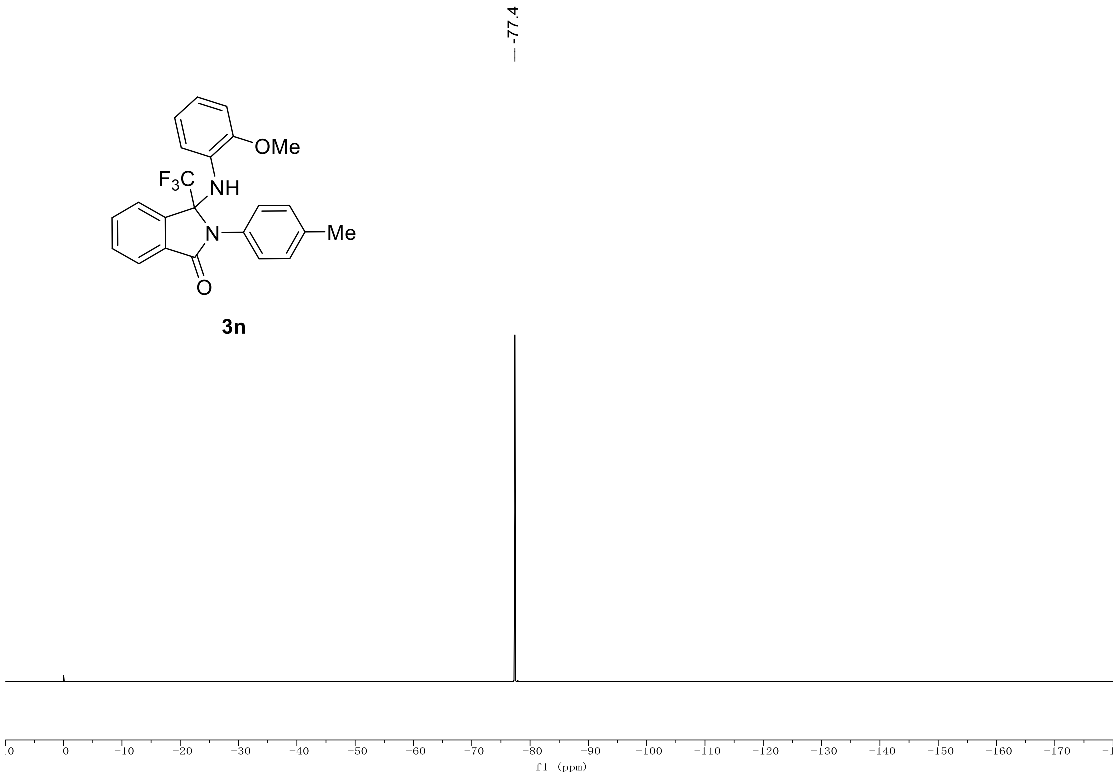

Figure S160. ^{19}F NMR (376 MHz, $CDCl_3$) spectrum of compound 3n, related to Scheme 2

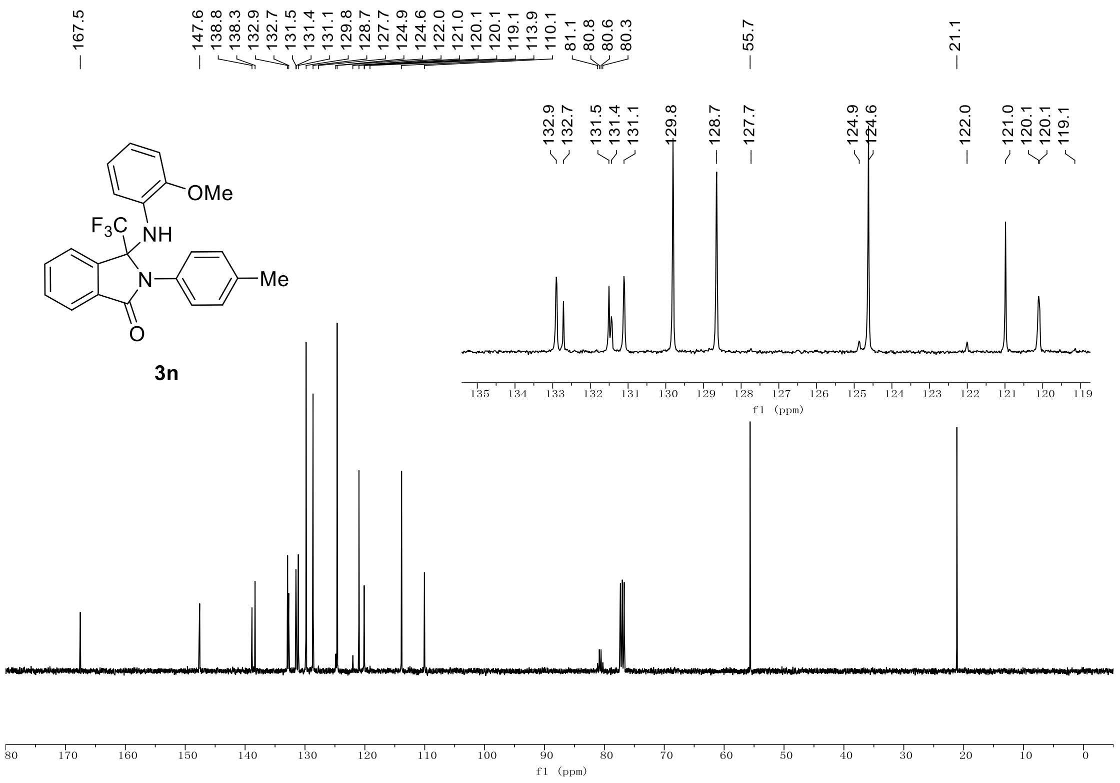

Figure S161. ^{13}C NMR (101 MHz, CDCl$_3$) spectrum of compound 3n, related to Scheme 2

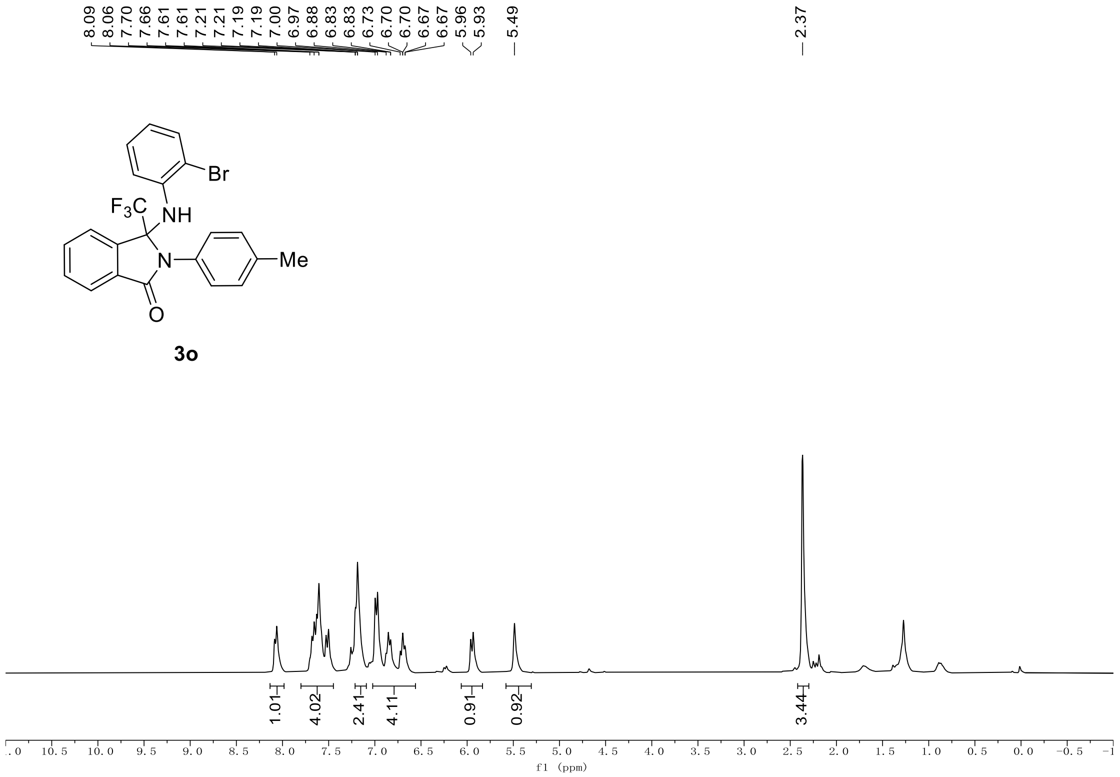

Figure S162. ^{1}H NMR (400 MHz, CDCl$_3$) spectrum of compound 3o, related to Scheme 2

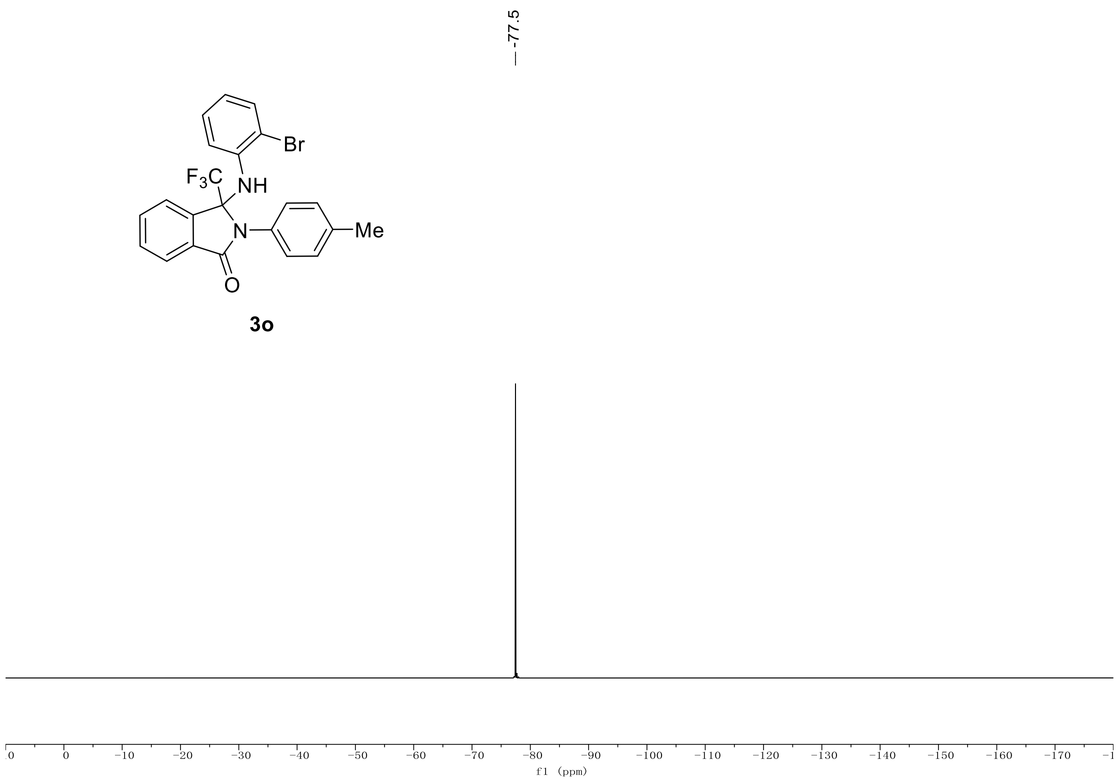

Figure S163. ^{19}F NMR (376 MHz, $CDCl_3$) spectrum of compound 3o, related to Scheme 2

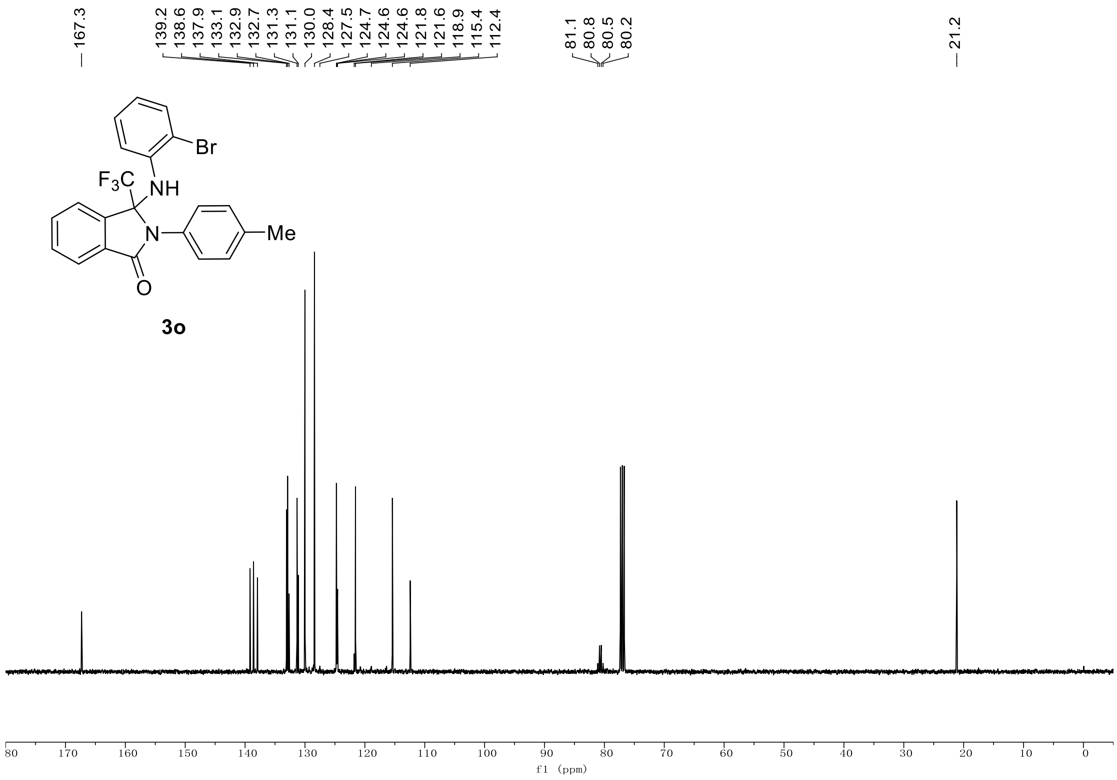

Figure S164. ^{13}C NMR (101 MHz, $CDCl_3$) spectrum of compound 3o, related to Scheme 2

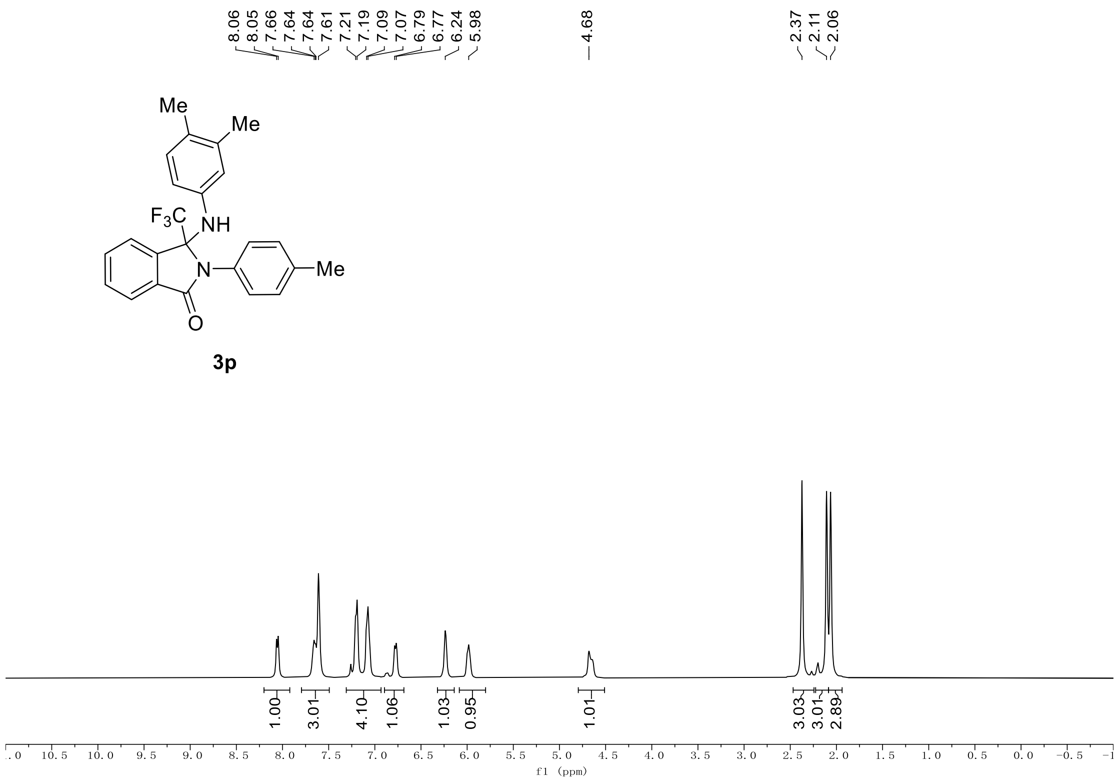

Figure S165. ^{1}H NMR (400 MHz, $CDCl_3$) spectrum of compound 3p, related to Scheme 2

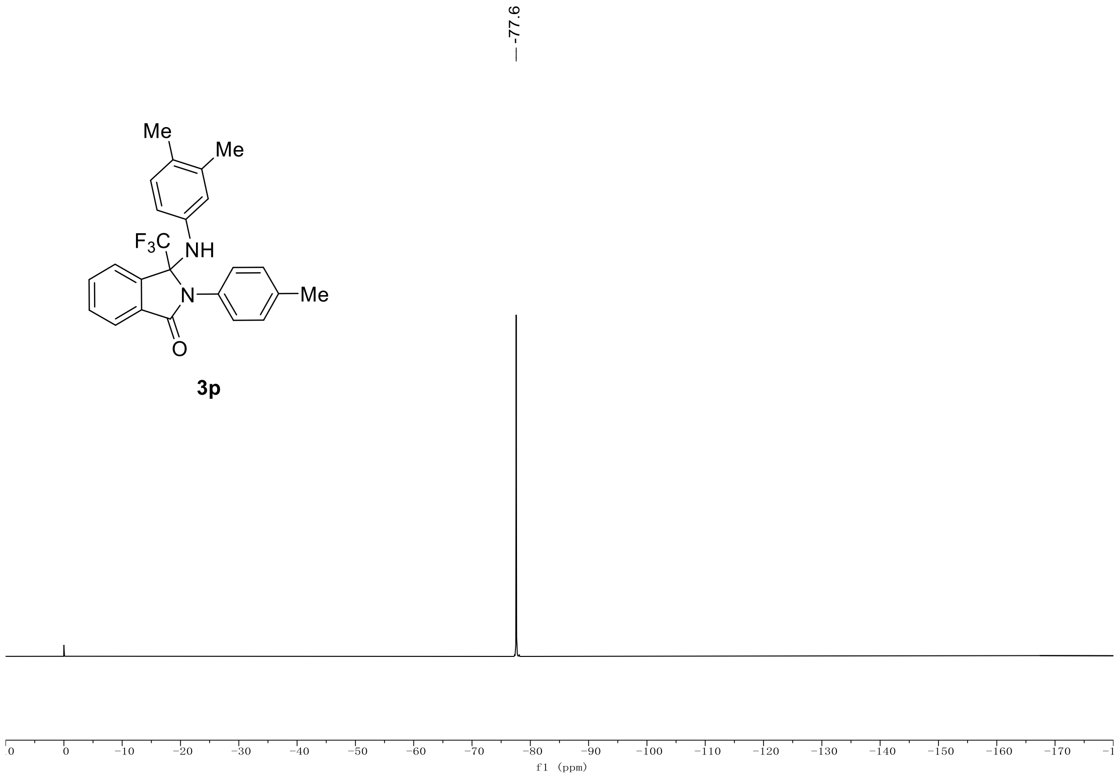

Figure S166. ^{19}F NMR (376 MHz, $CDCl_3$) spectrum of compound 3p, related to Scheme 2

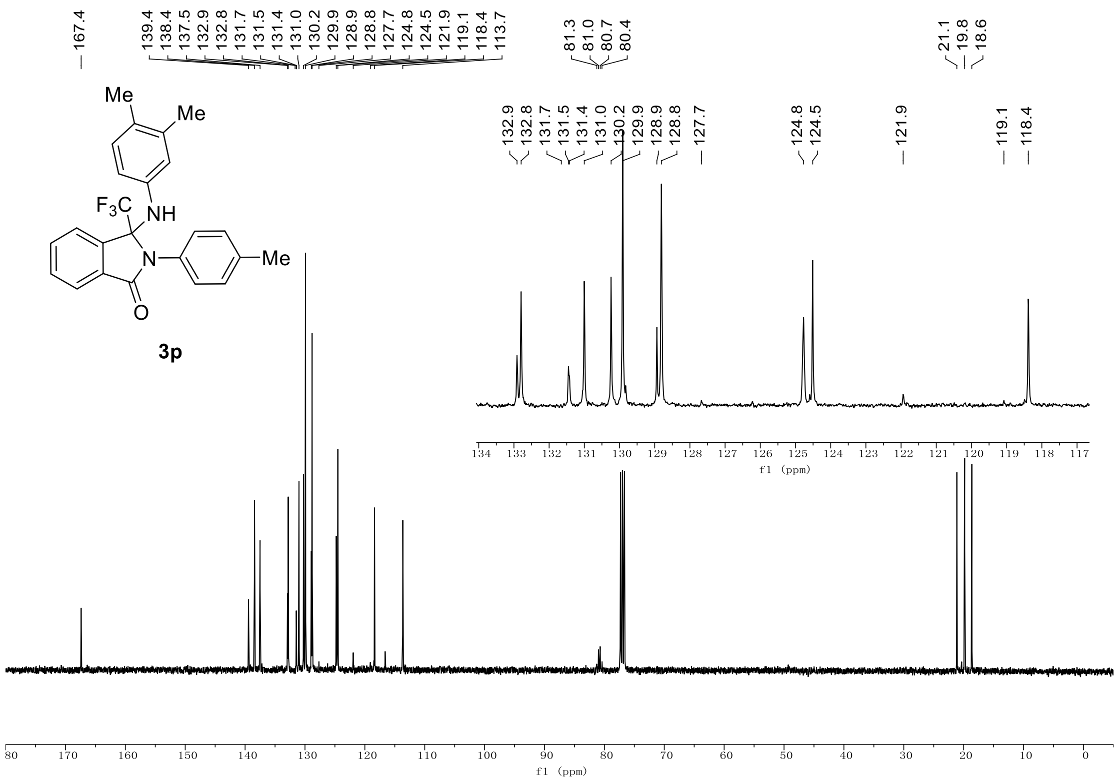

Figure S167. ^{13}C NMR (101 MHz, CDCl3) spectrum of compound 3p, related to Scheme 2

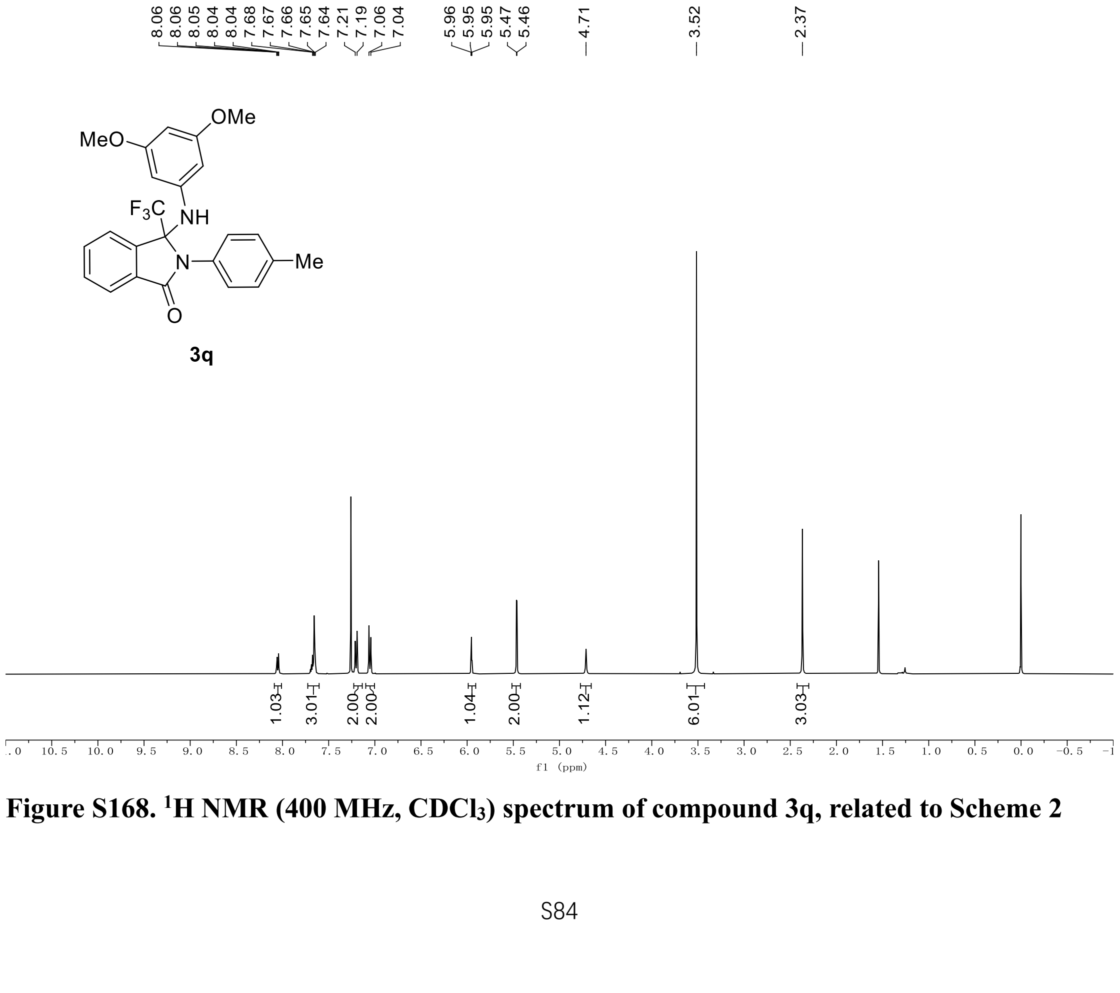

Figure S168. ^{1}H NMR (400 MHz, CDCl3) spectrum of compound 3q, related to Scheme 2

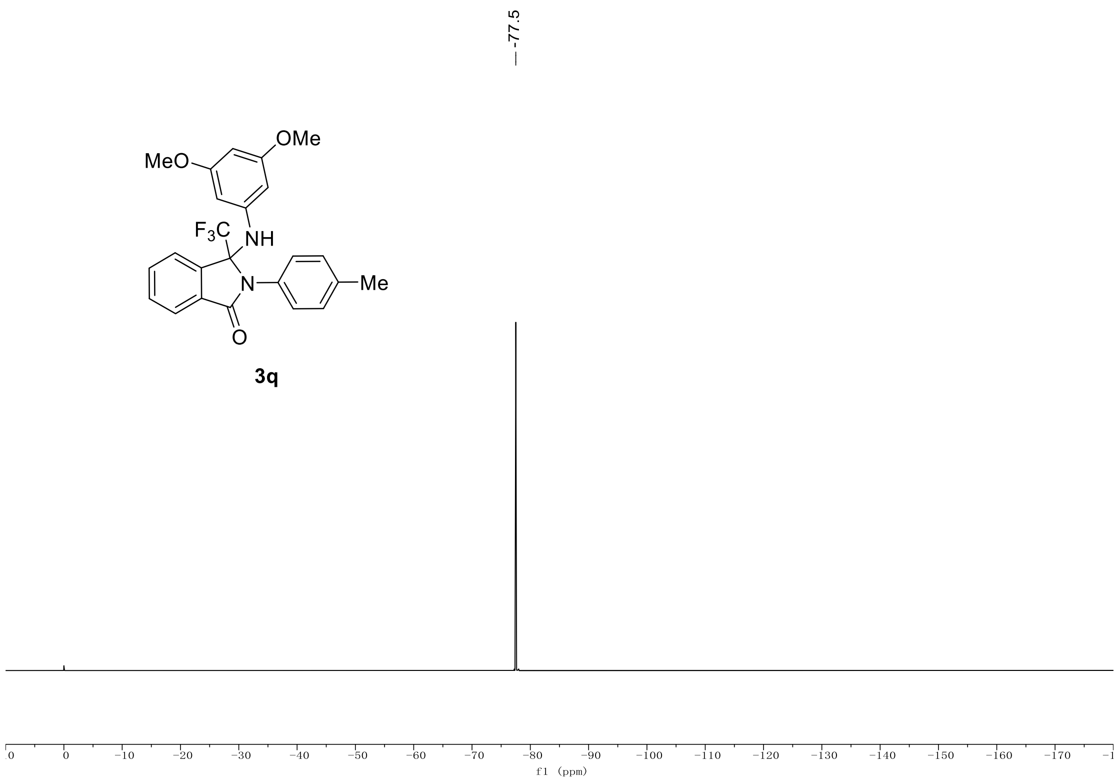

Figure S169. ^{19}F NMR (376 MHz, $CDCl_3$) spectrum of compound 3q, related to Scheme 2

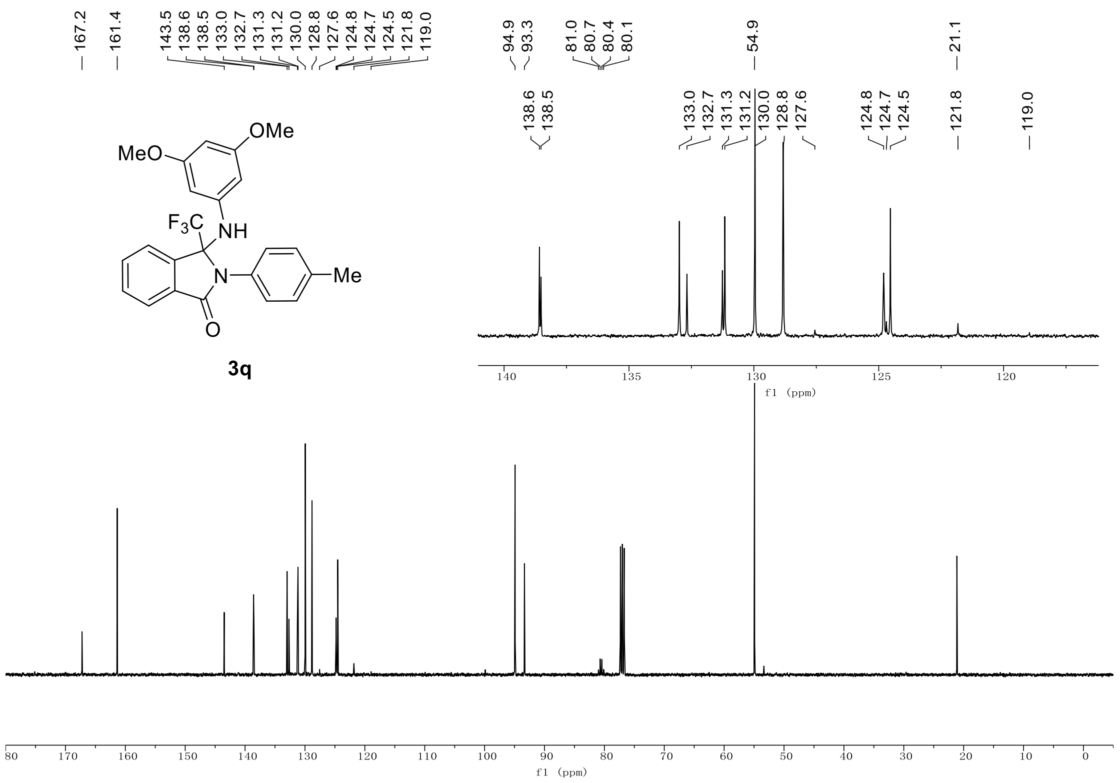

Figure S170. ^{13}C NMR (101 MHz, $CDCl_3$) spectrum of compound 3q, related to Scheme 2

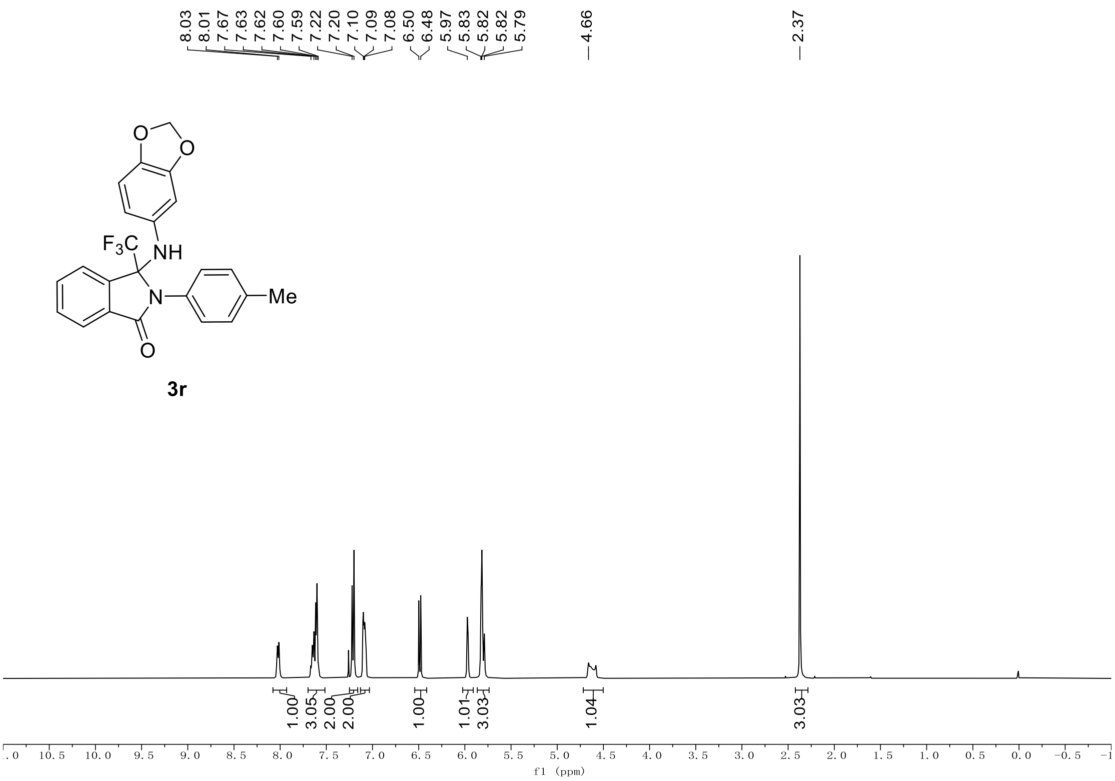

Figure S171. ^{1}**H NMR (400 MHz,** $CDCl_3$**) spectrum of compound 3r, related to Scheme 2**

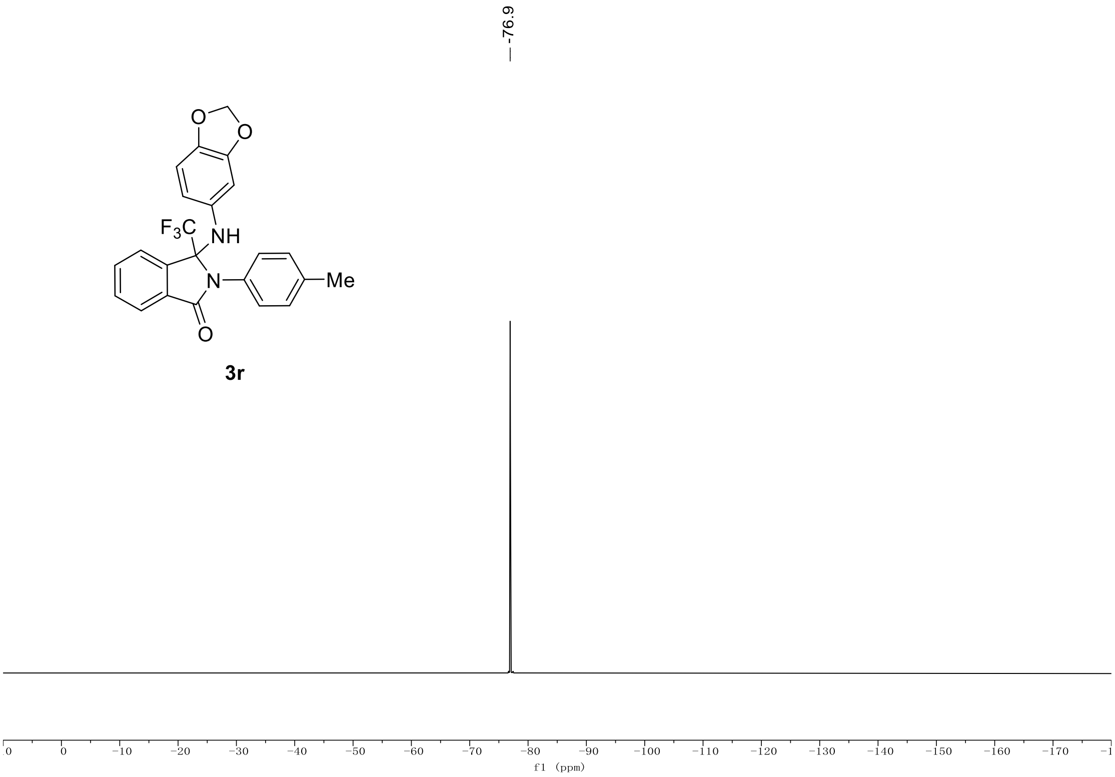

Figure S172. ^{19}**F NMR (376 MHz,** $CDCl_3$**) spectrum of compound 3r, related to Scheme 2**

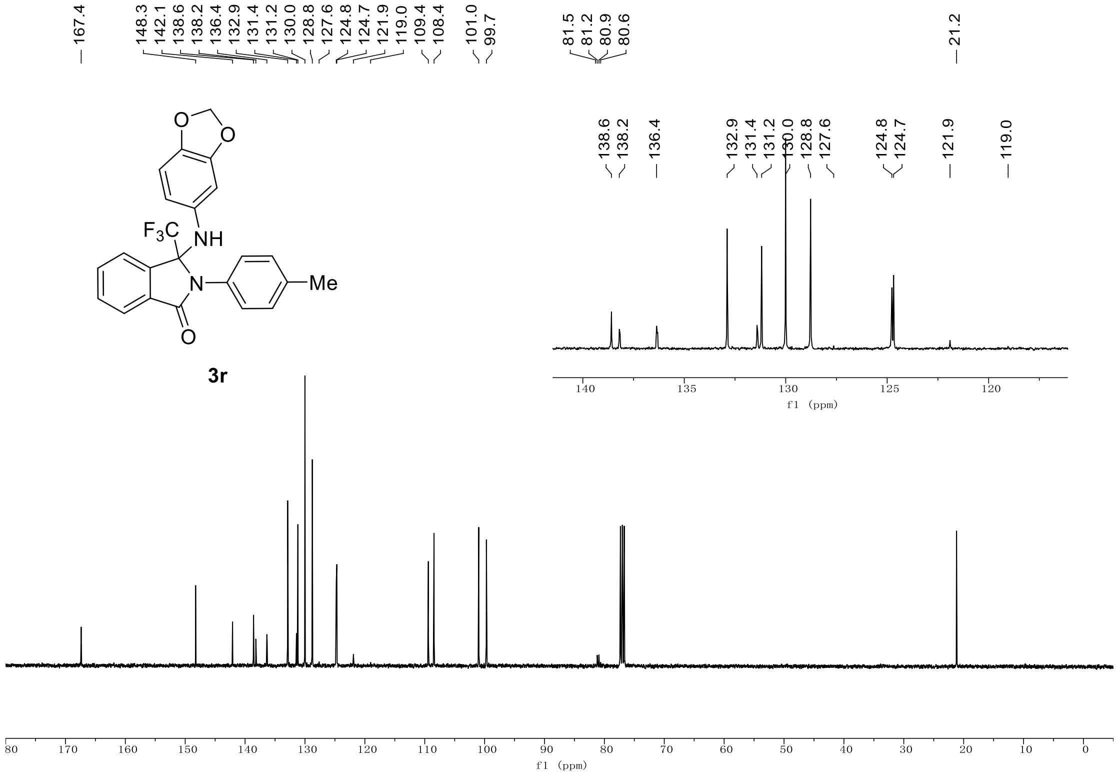

Figure S173. ^{13}C NMR (101 MHz, $CDCl_3$) spectrum of compound 3r, related to Scheme 2

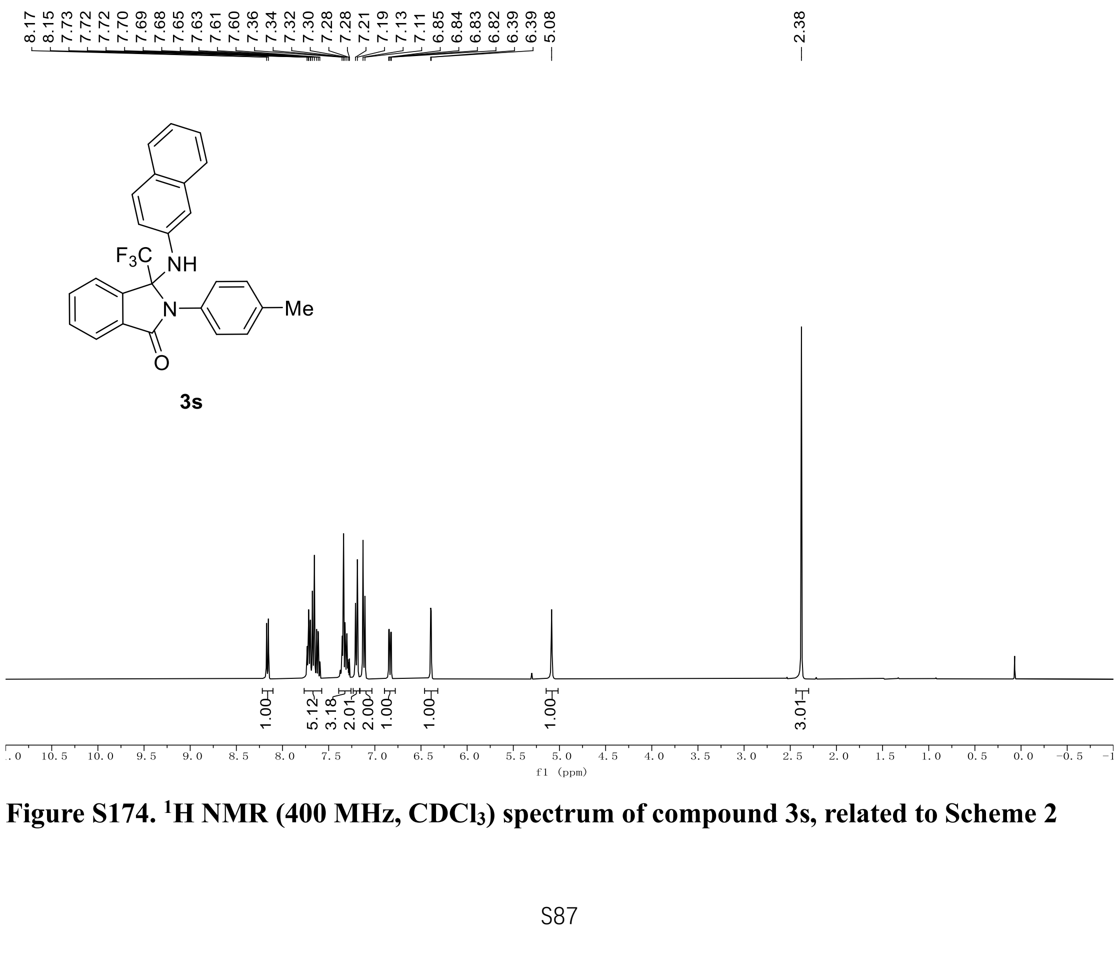

Figure S174. ^{1}H NMR (400 MHz, $CDCl_3$) spectrum of compound 3s, related to Scheme 2

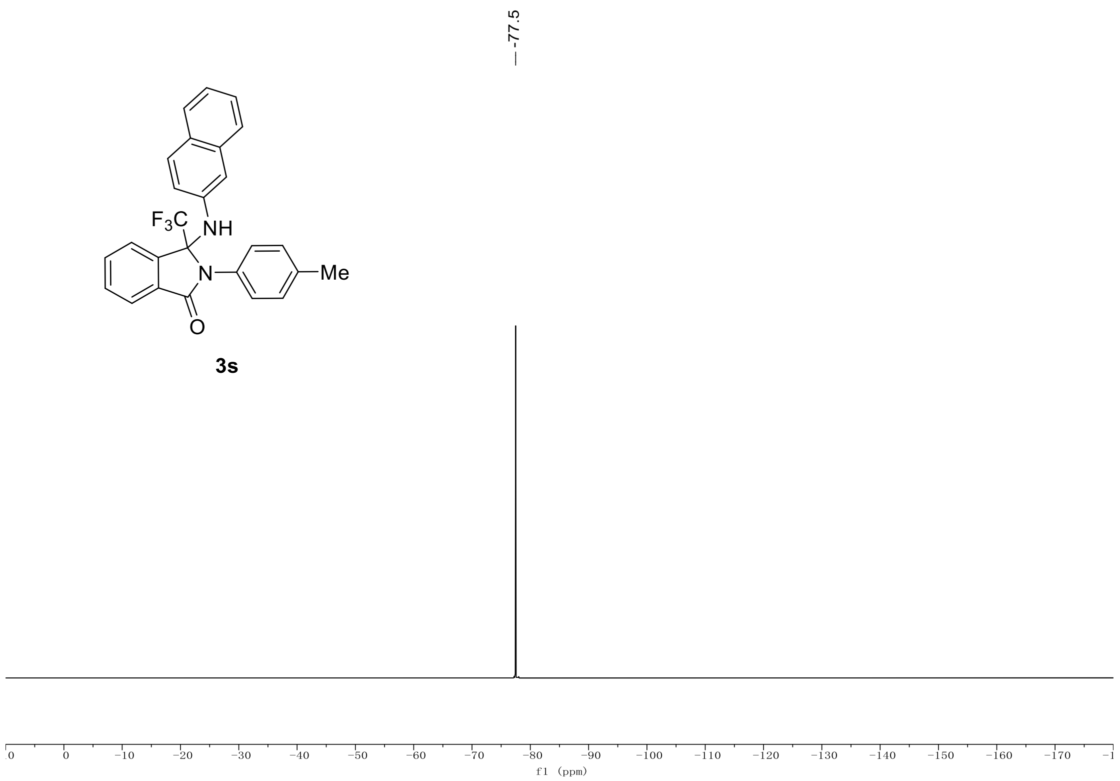

Figure S175. ^{19}F NMR (376 MHz, CDCl$_3$) spectrum of compound 3s, related to Scheme 2

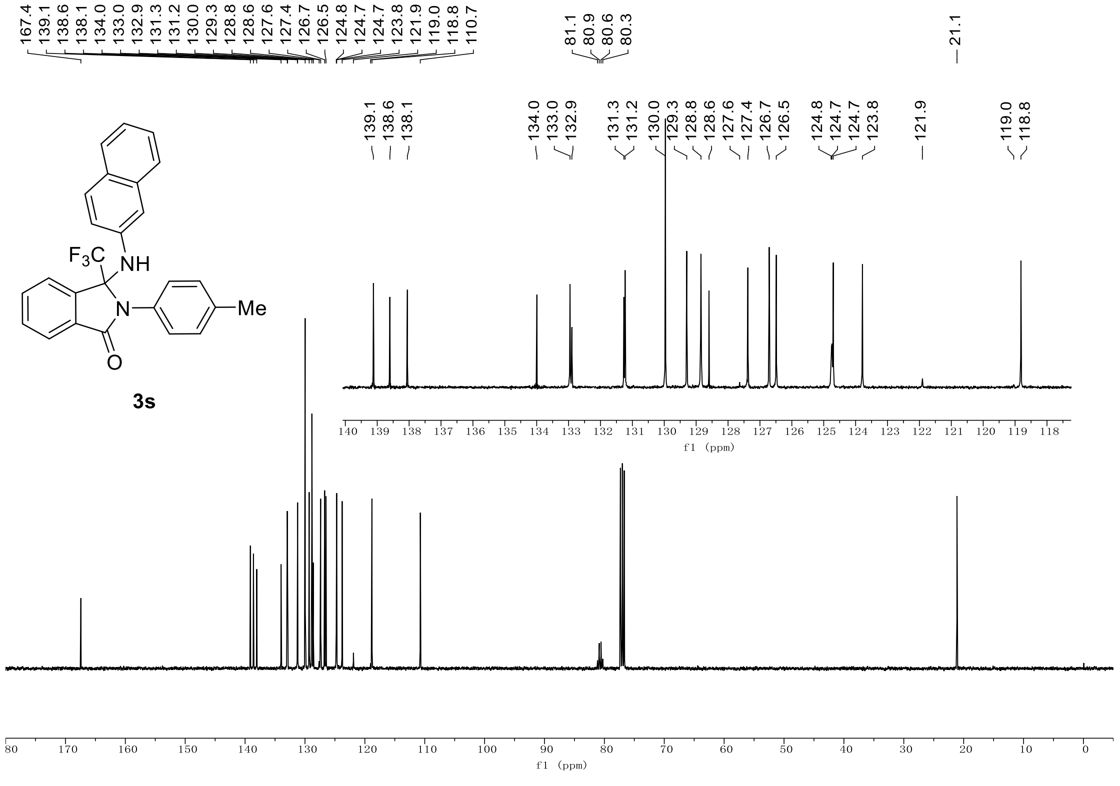

Figure S176. ^{13}C NMR (101 MHz, CDCl$_3$) spectrum of compound 3s, related to Scheme 2

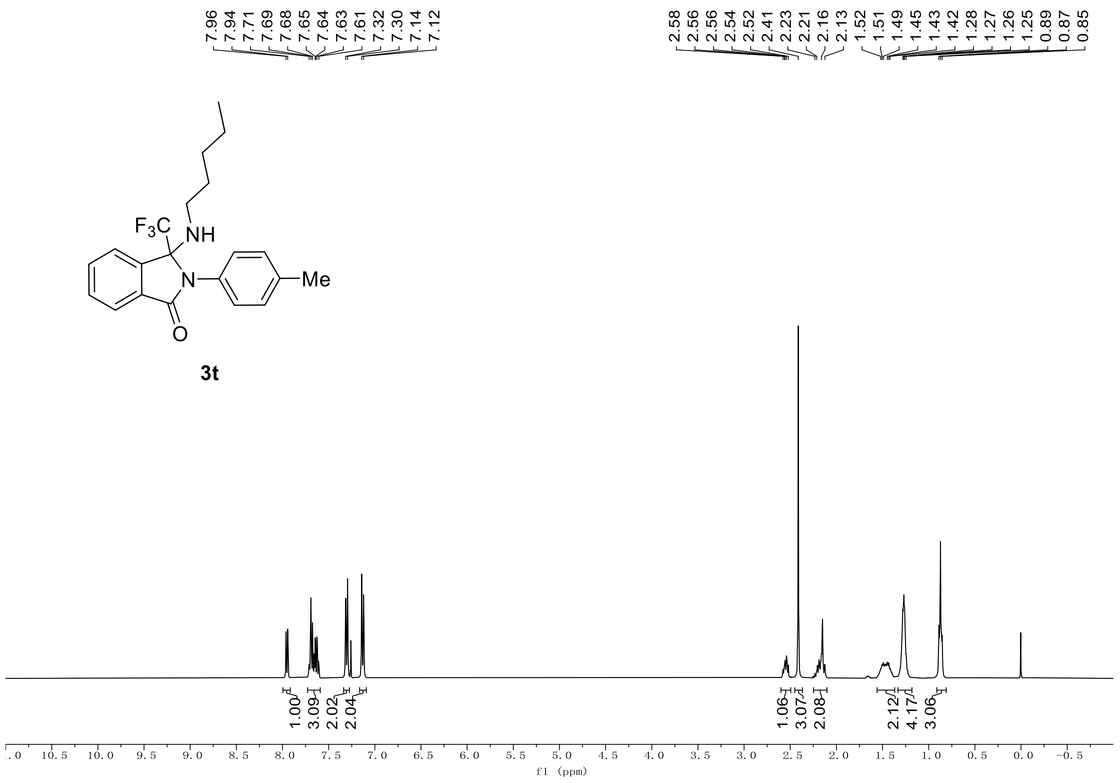

Figure S177. ^{1}H NMR (400 MHz, $CDCl_3$) spectrum of compound 3t, related to Scheme 2

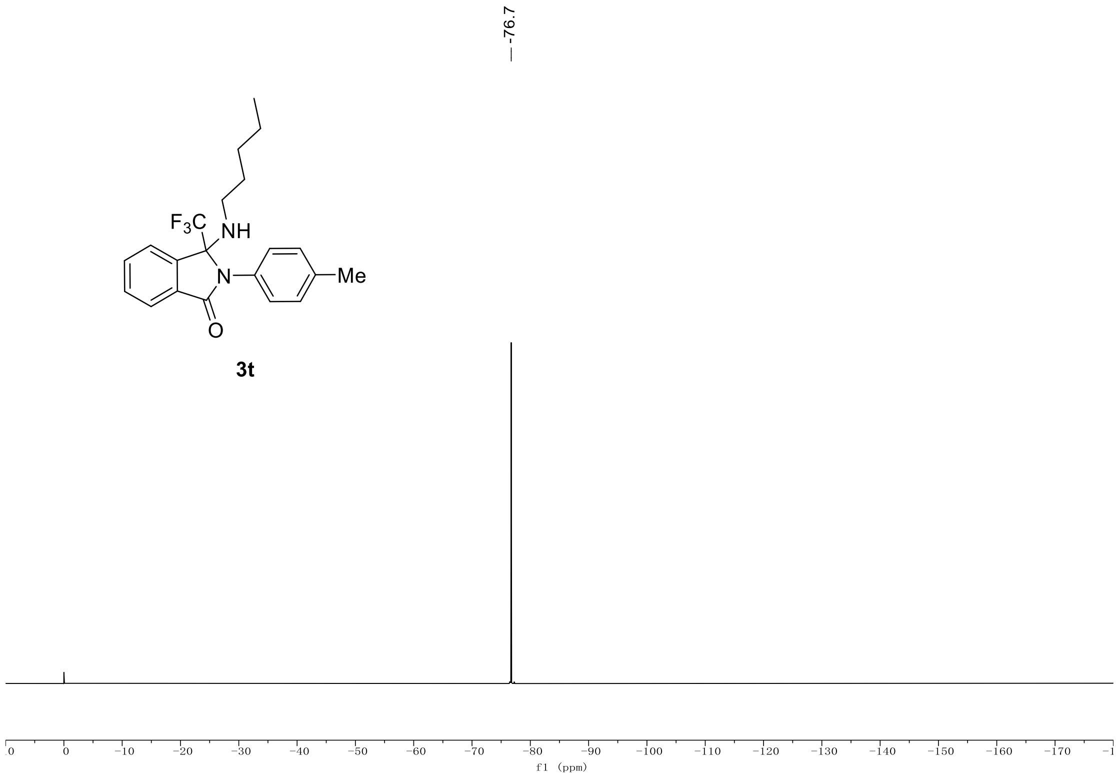

Figure S178. ^{19}F NMR (376 MHz, $CDCl_3$) spectrum of compound 3t, related to Scheme 2

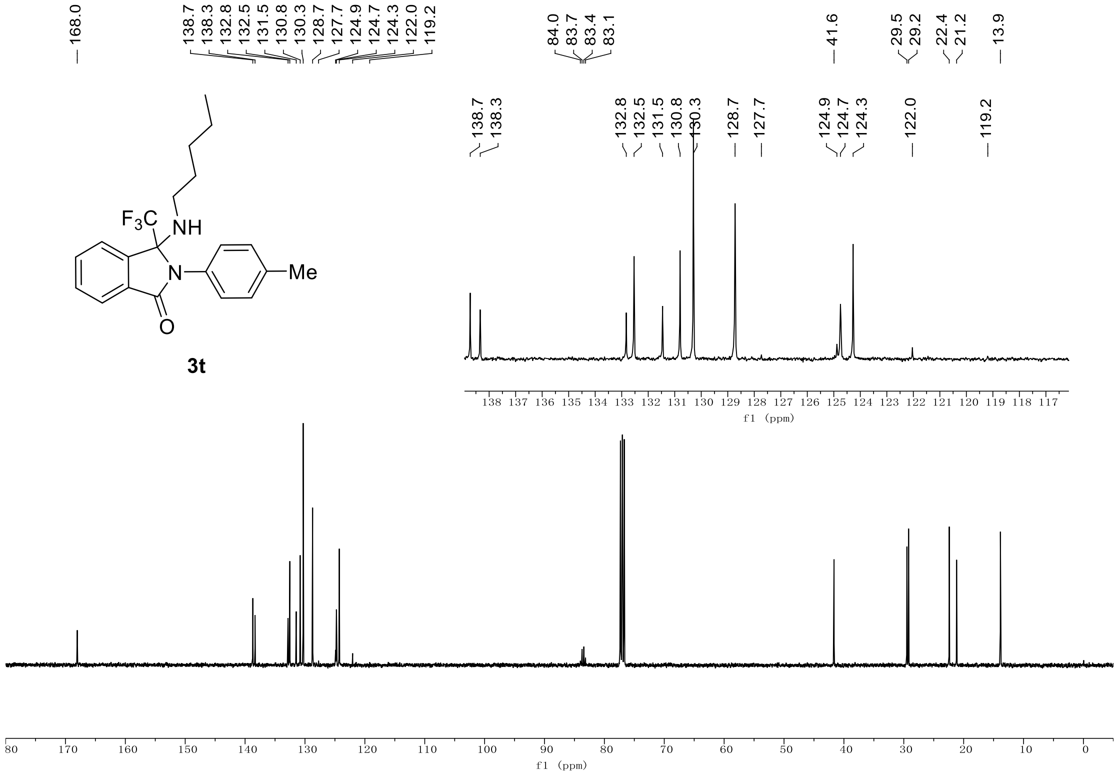

Figure S179. ^{13}C NMR (101 MHz, $CDCl_3$) spectrum of compound 3t, related to Scheme 2

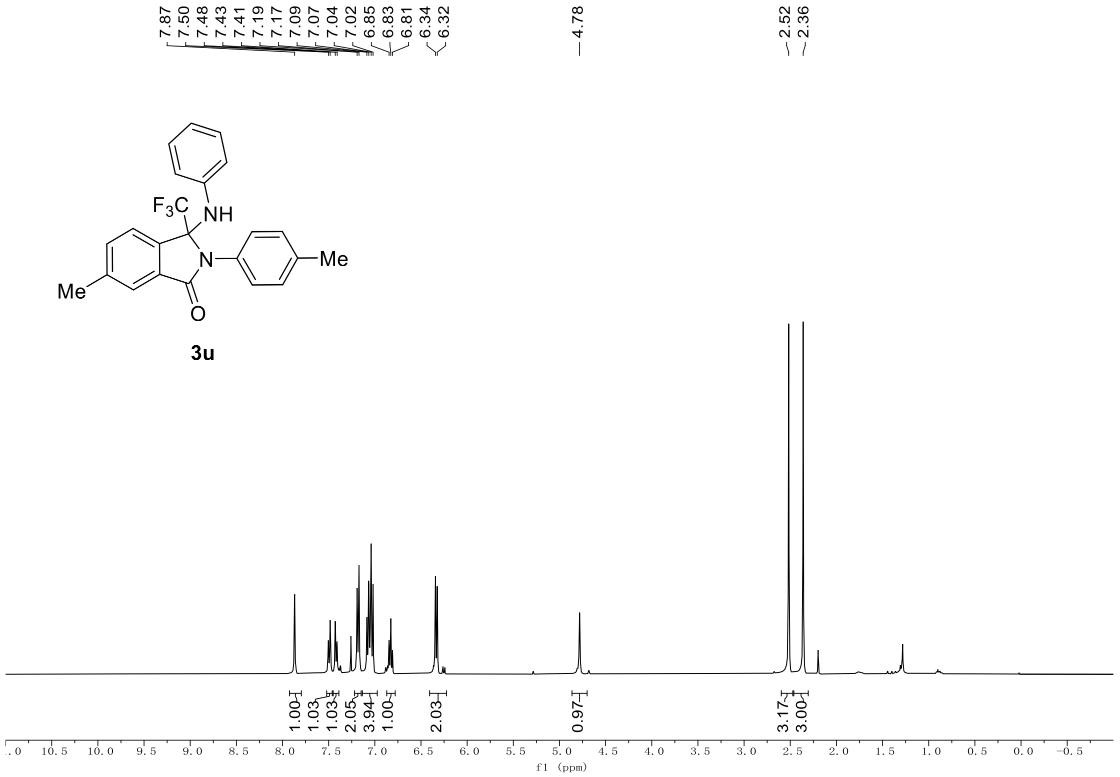

Figure S180. ^{1}H NMR (400 MHz, $CDCl_3$) spectrum of compound 3u, related to Scheme 2

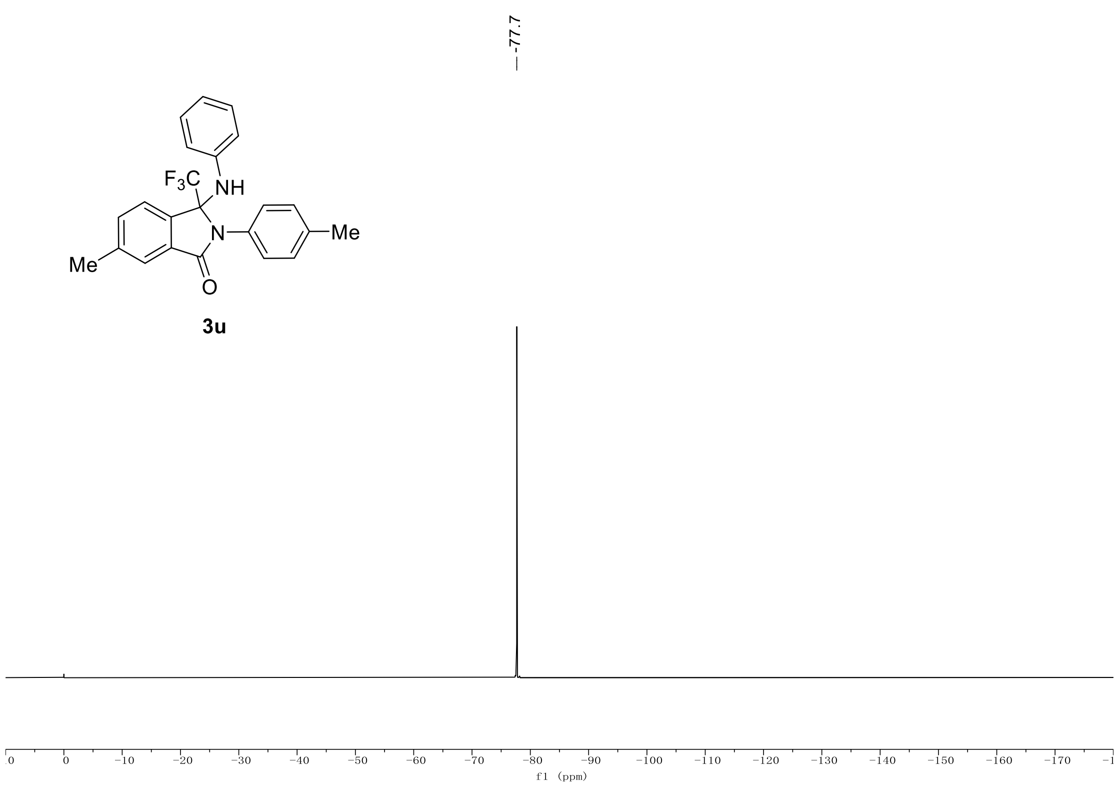

Figure S181. ^{19}F NMR (376 MHz, $CDCl_3$) spectrum of compound 3u, related to Scheme 2

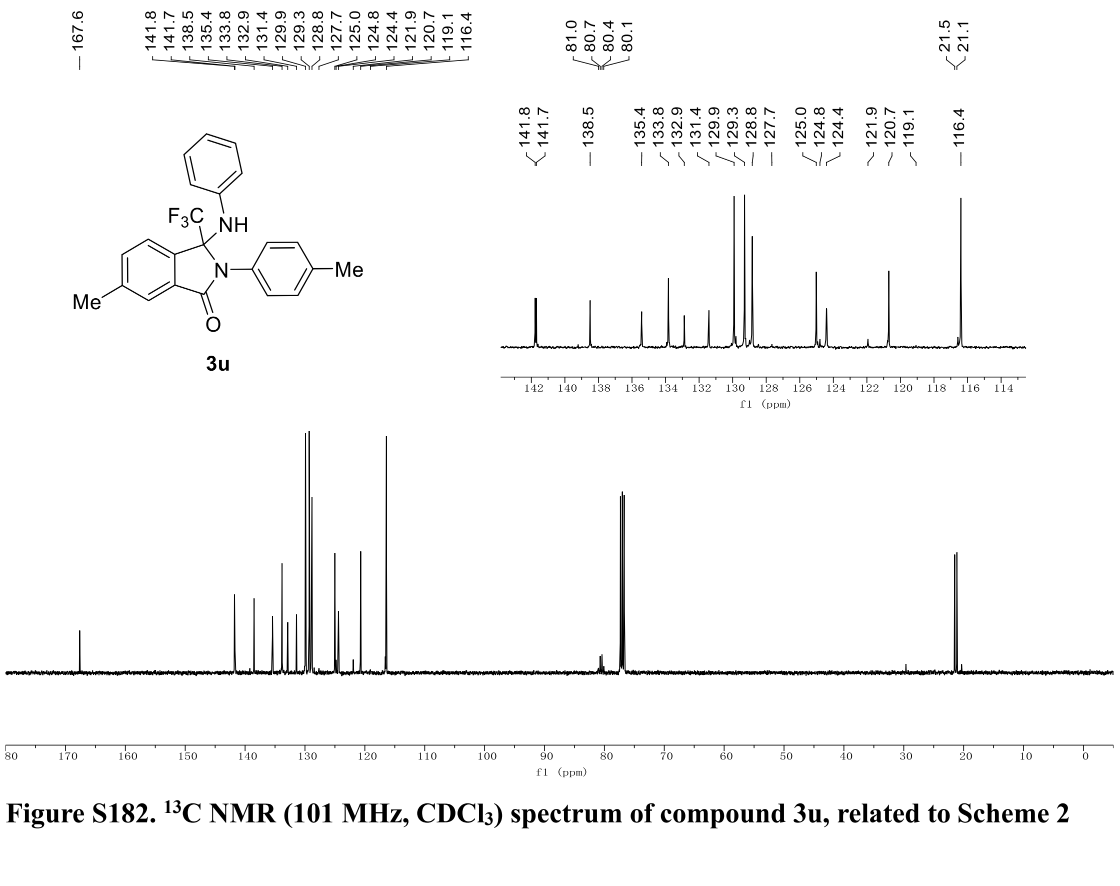

Figure S182. ^{13}C NMR (101 MHz, $CDCl_3$) spectrum of compound 3u, related to Scheme 2

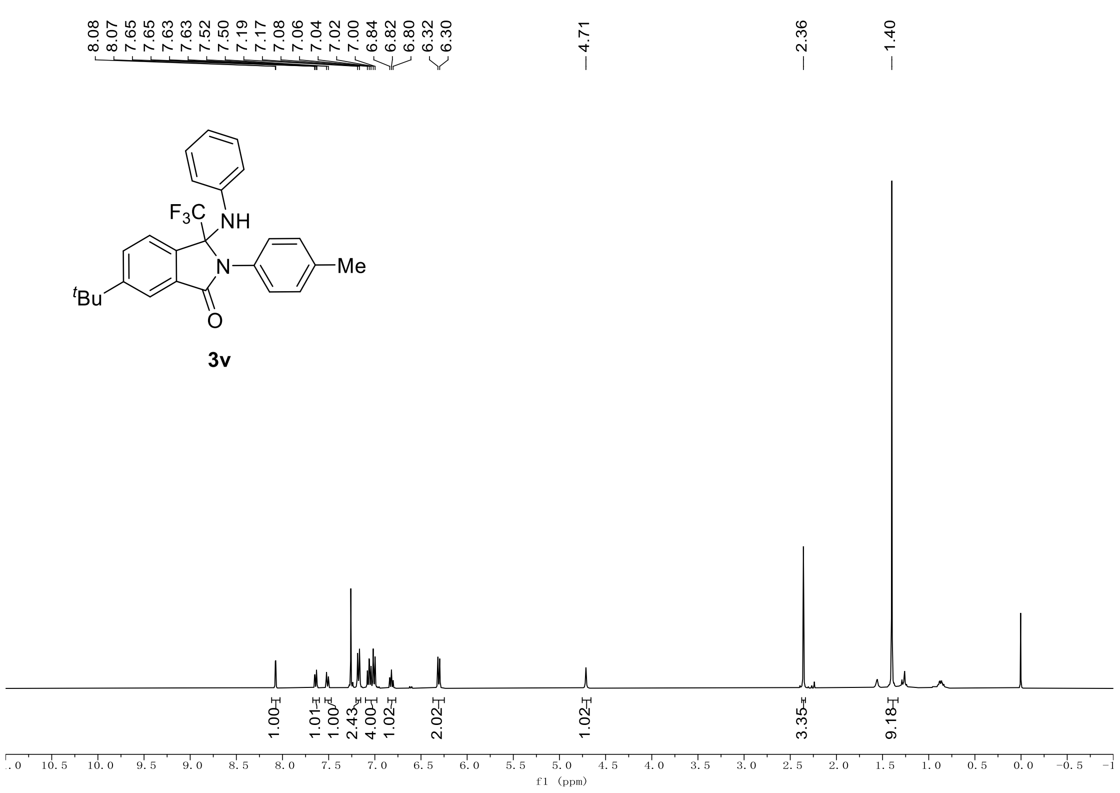

Figure S183. ^{1}H NMR (400 MHz, $CDCl_3$) spectrum of compound 3v, related to Scheme 2

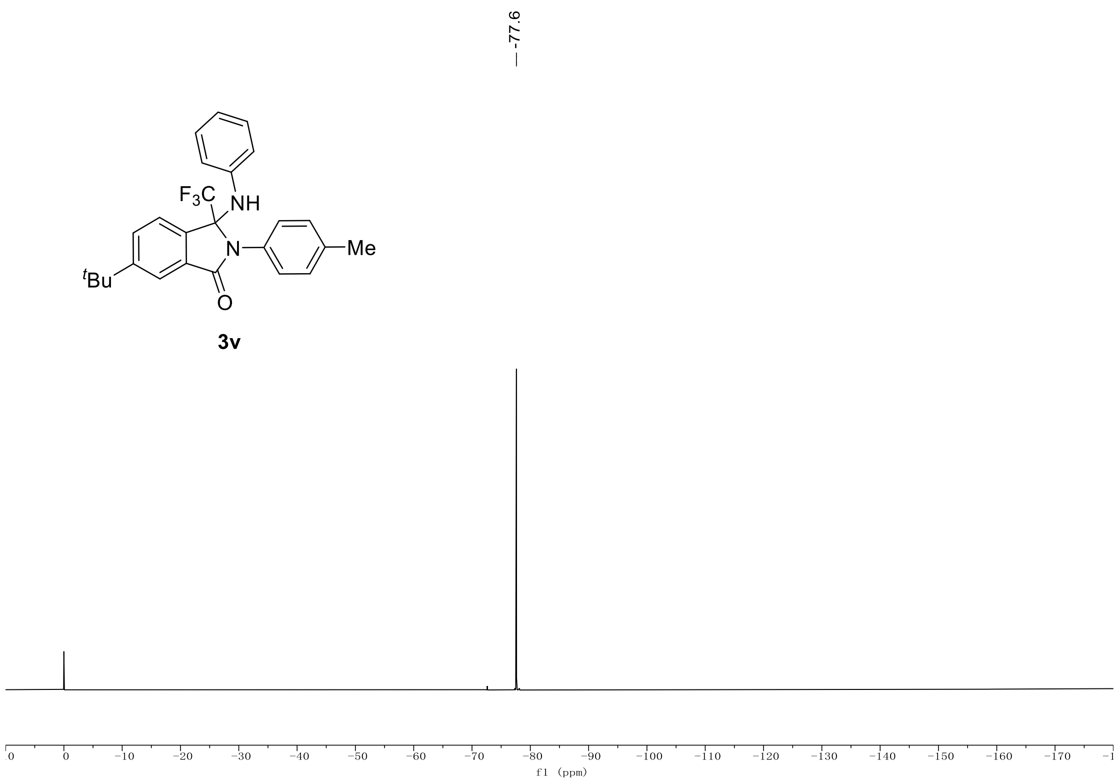

Figure S184. ^{19}F NMR (376 MHz, $CDCl_3$) spectrum of compound 3v, related to Scheme 2

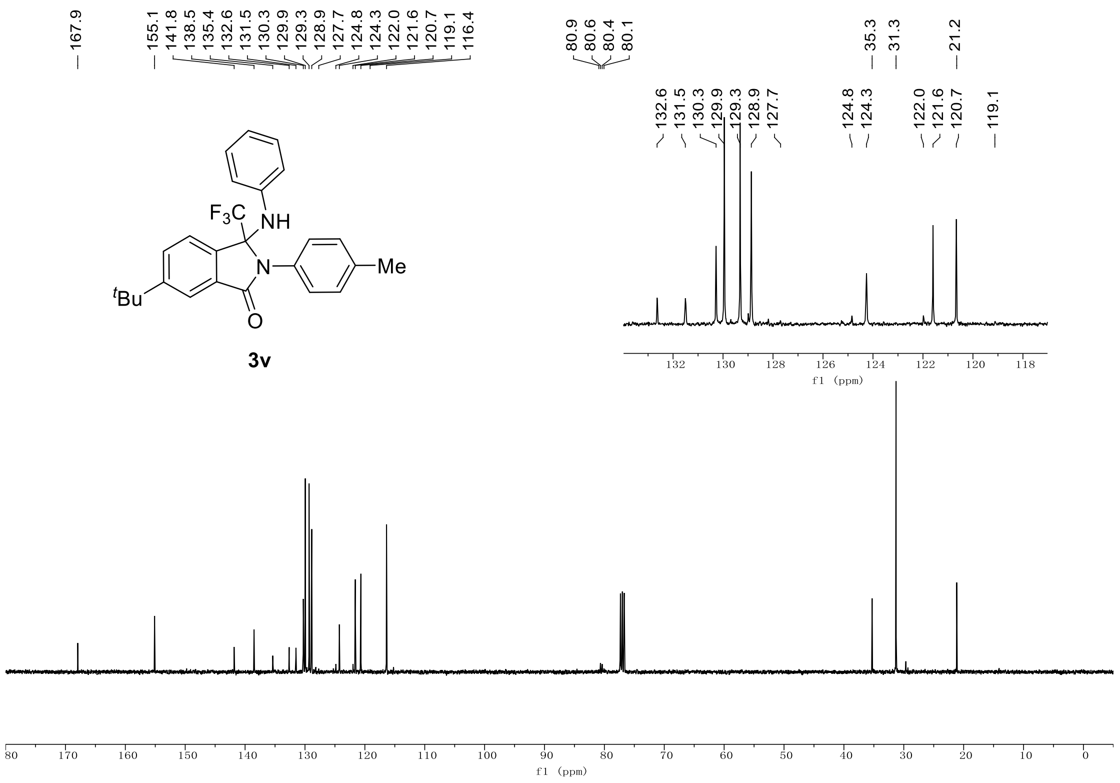

Figure S185. ^{13}C NMR (101 MHz, $CDCl_3$) spectrum of compound 3v, related to Scheme 2

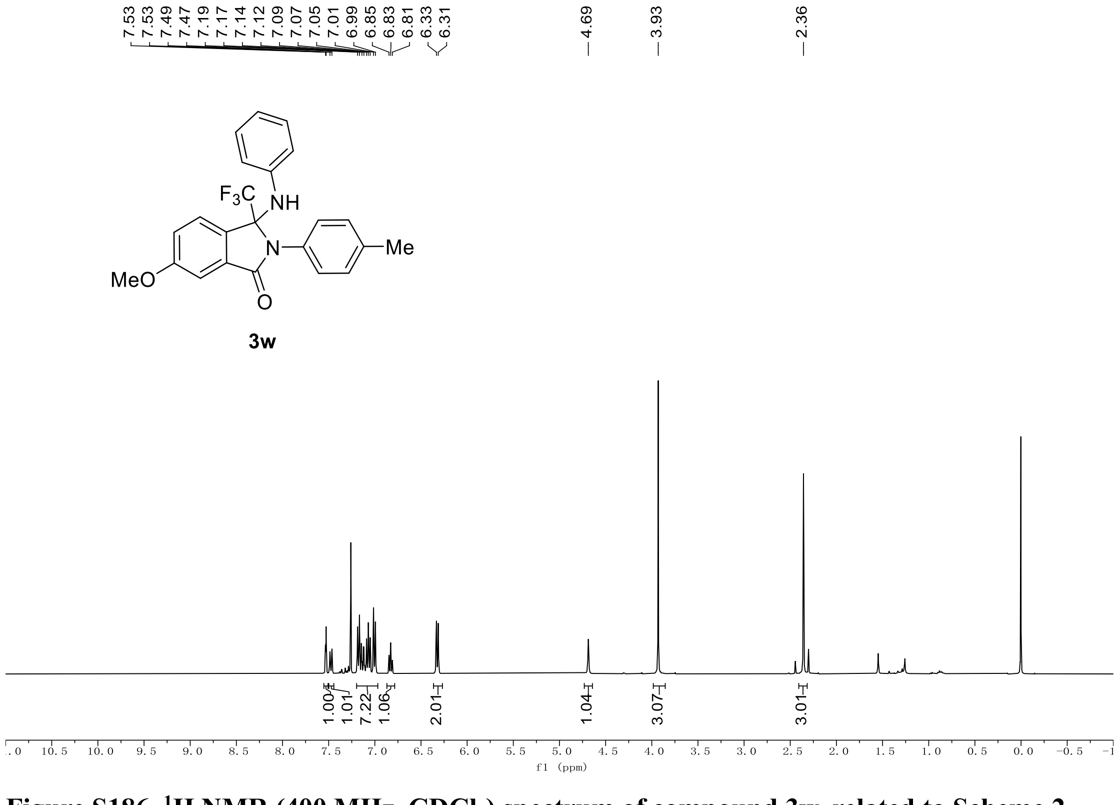

Figure S186. ^{1}H NMR (400 MHz, $CDCl_3$) spectrum of compound 3w, related to Scheme 2

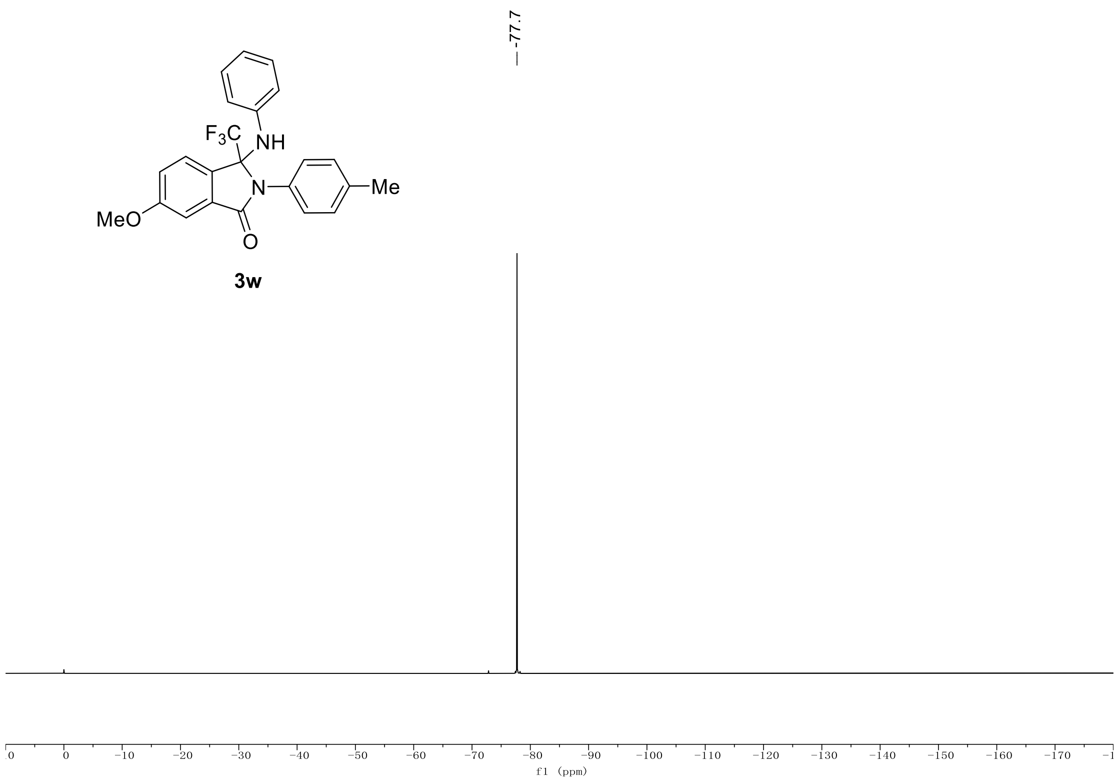

Figure S187. ^{19}F NMR (376 MHz, $CDCl_3$) spectrum of compound 3w, related to Scheme 2

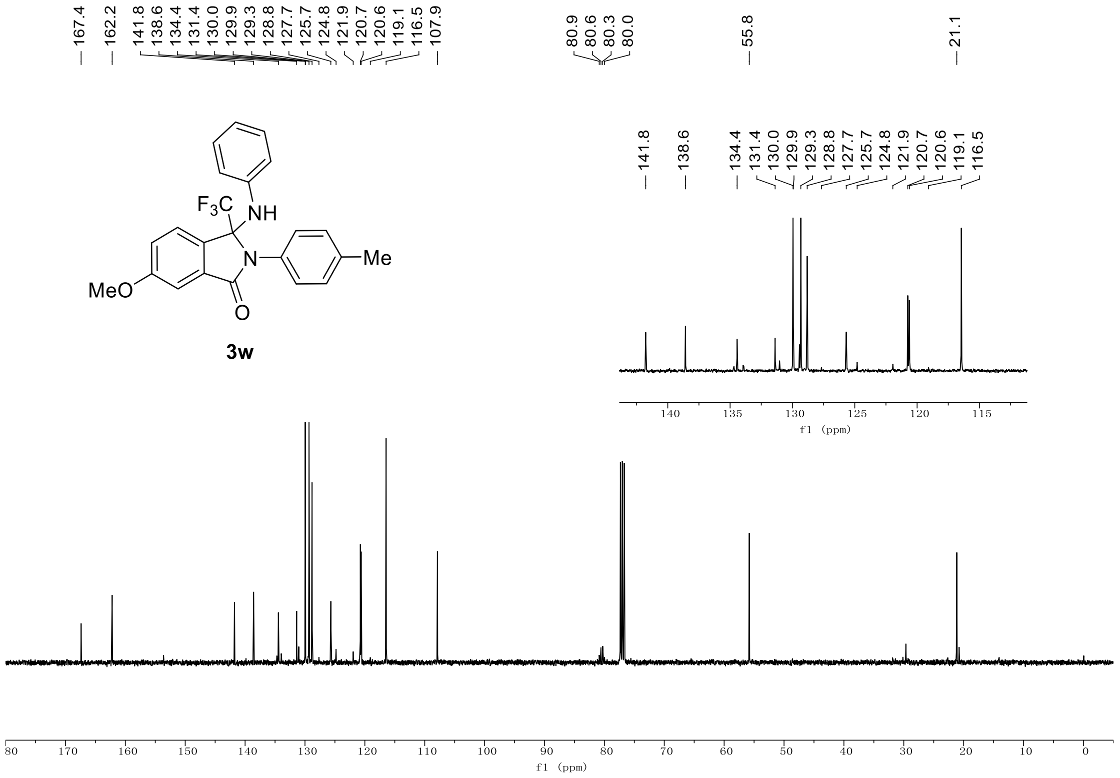

Figure S188. ^{13}C NMR (101 MHz, $CDCl_3$) spectrum of compound 3w, related to Scheme 2

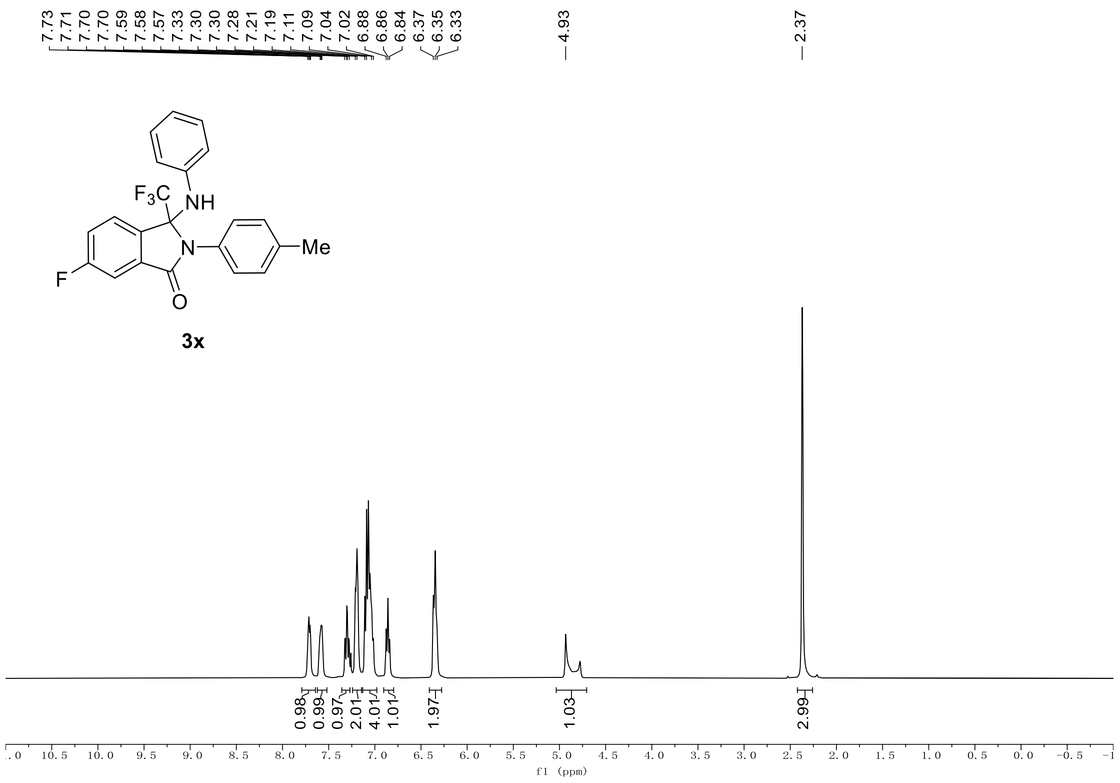

Figure S189. ^{1}**H NMR (400 MHz, CDCl$_3$) spectrum of compound 3x, related to Scheme 2**

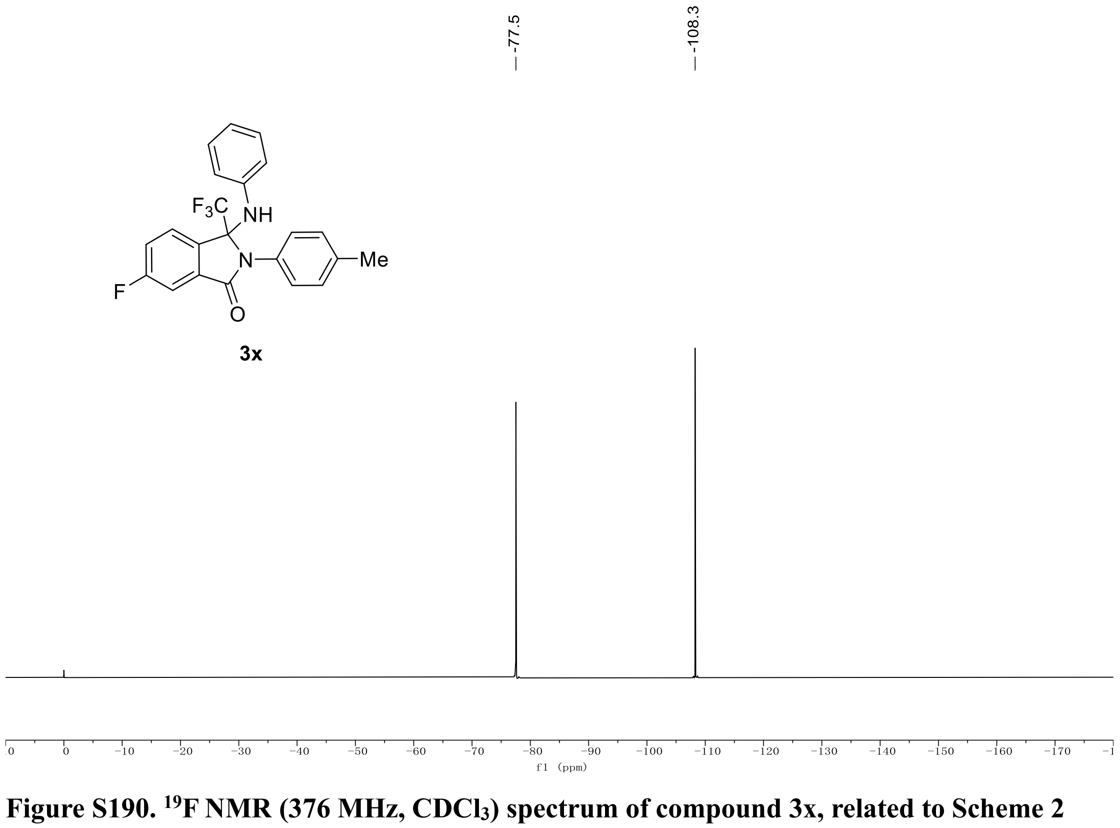

Figure S190. ^{19}**F NMR (376 MHz, CDCl$_3$) spectrum of compound 3x, related to Scheme 2**

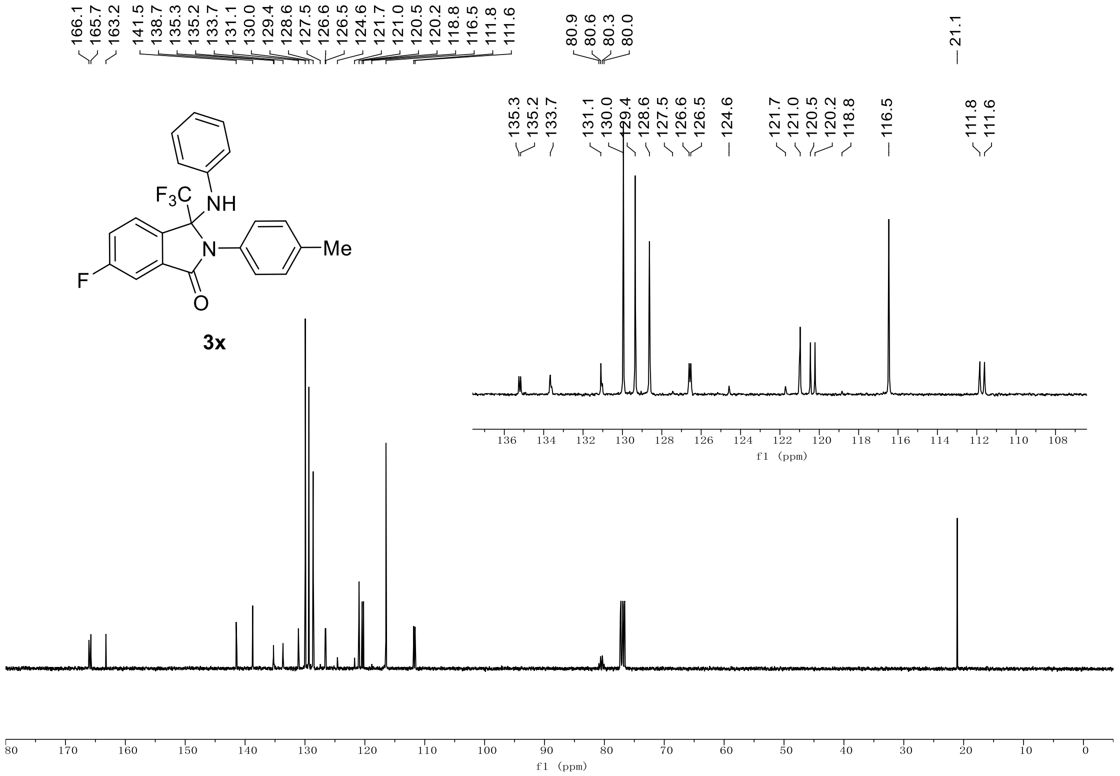

Figure S191. ^{13}C **NMR (101 MHz,** $CDCl_3$**) spectrum of compound 3x, related to Scheme 2**

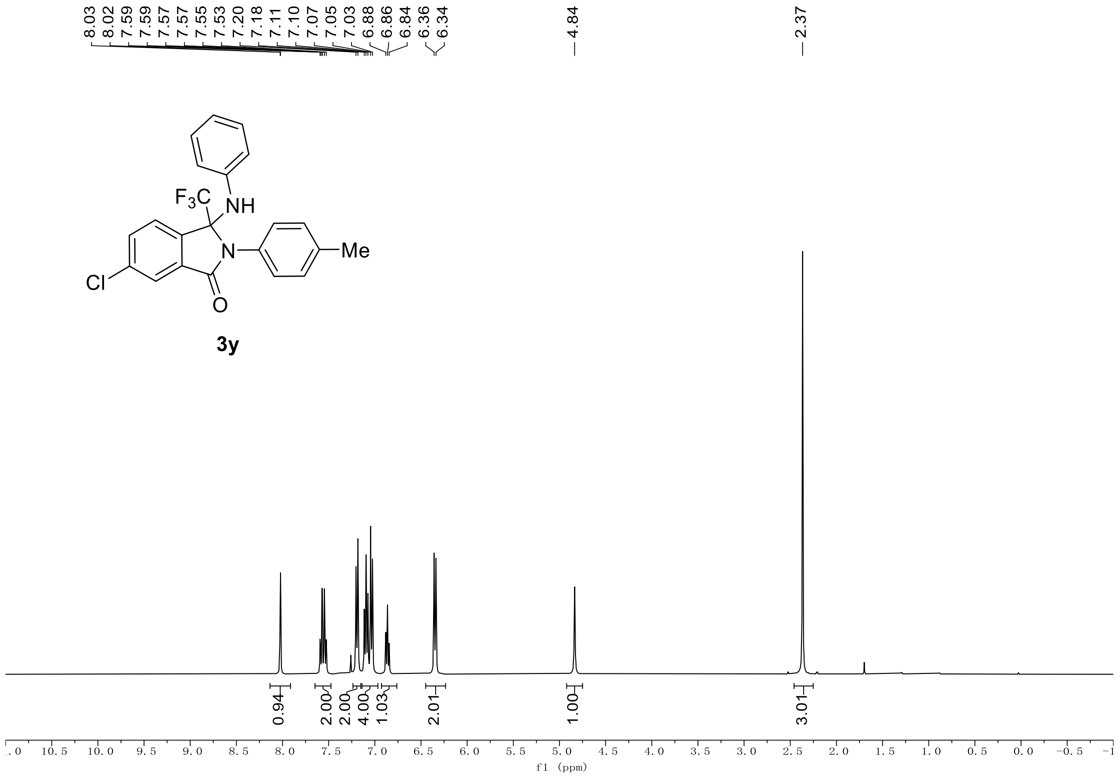

Figure S192. ^{1}H **NMR (400 MHz,** $CDCl_3$**) spectrum of compound 3y, related to Scheme 2**

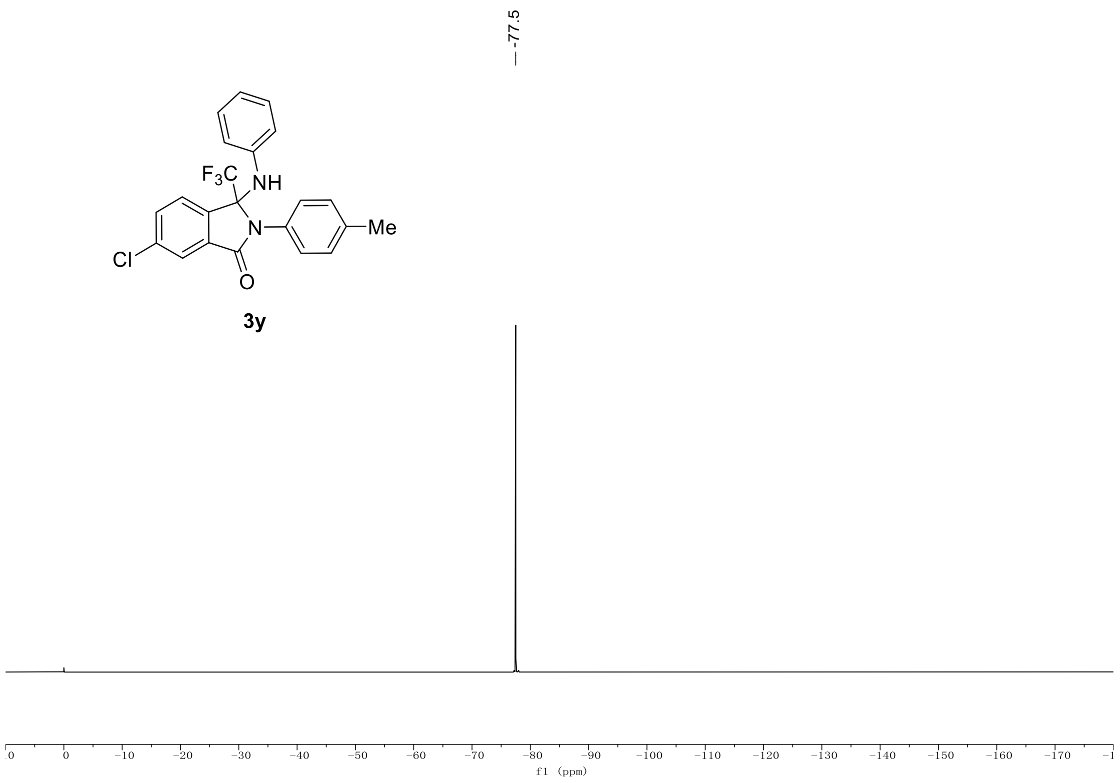

Figure S193. ^{19}F NMR (376 MHz, CDCl$_3$) spectrum of compound 3y, related to Scheme 2

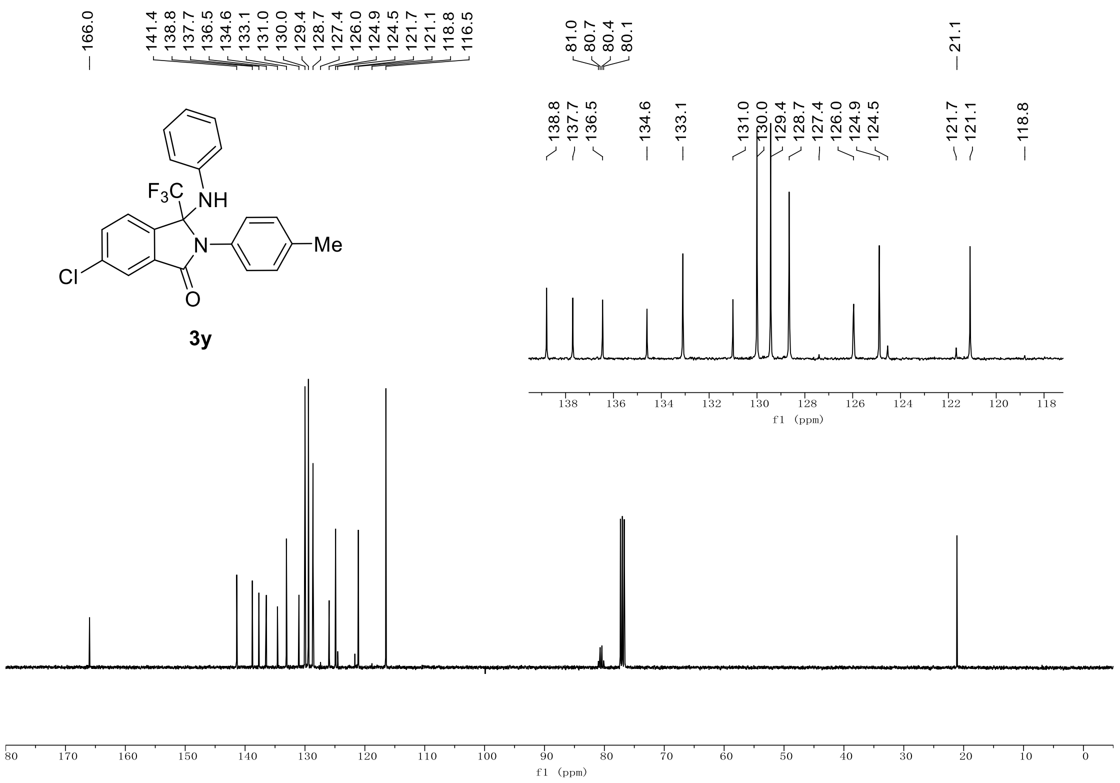

Figure S194. ^{13}C NMR (101 MHz, CDCl$_3$) spectrum of compound 3y, related to Scheme 2

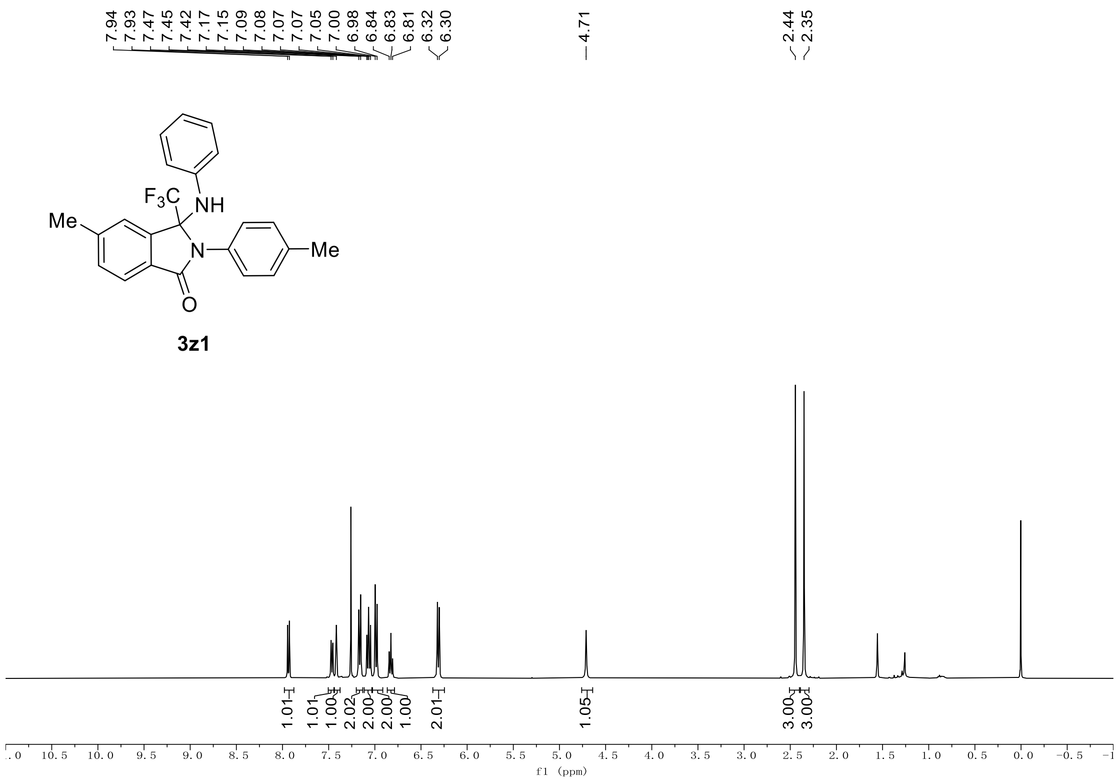

Figure S195. **^{1}H NMR (400 MHz, $CDCl_3$) spectrum of compound 3z1, related to Scheme 2**

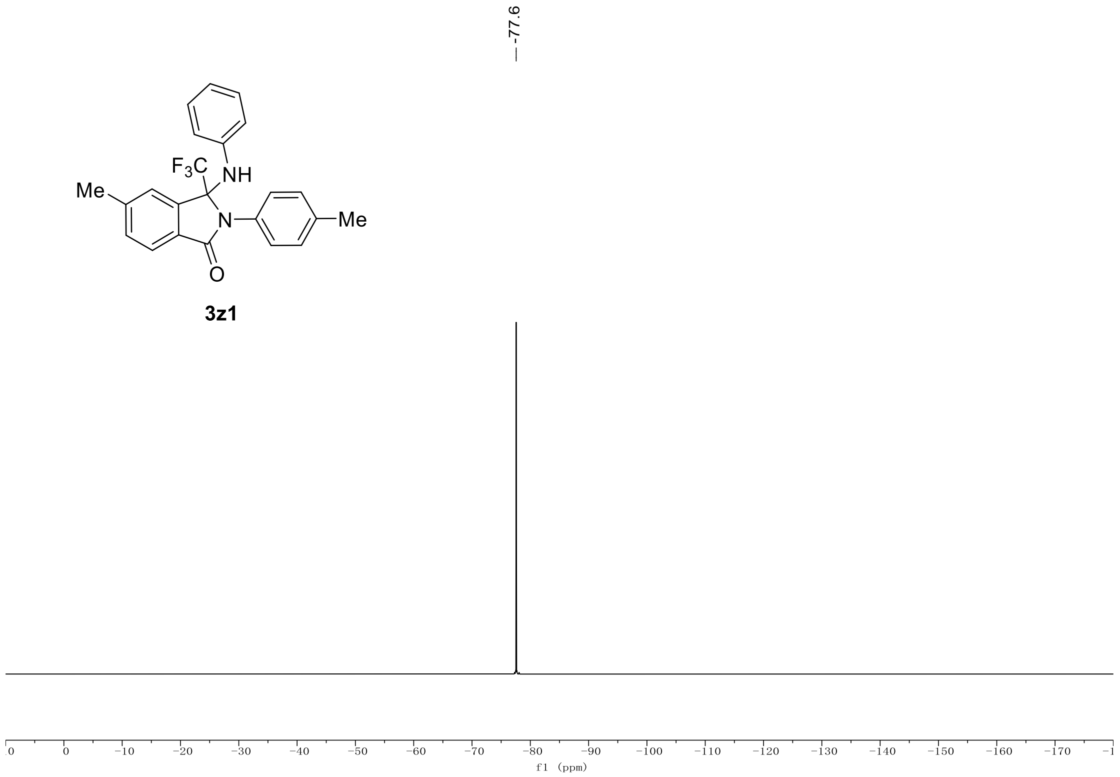

Figure S196. **^{19}F NMR (376 MHz, $CDCl_3$) spectrum of compound 3z1, related to Scheme 2**

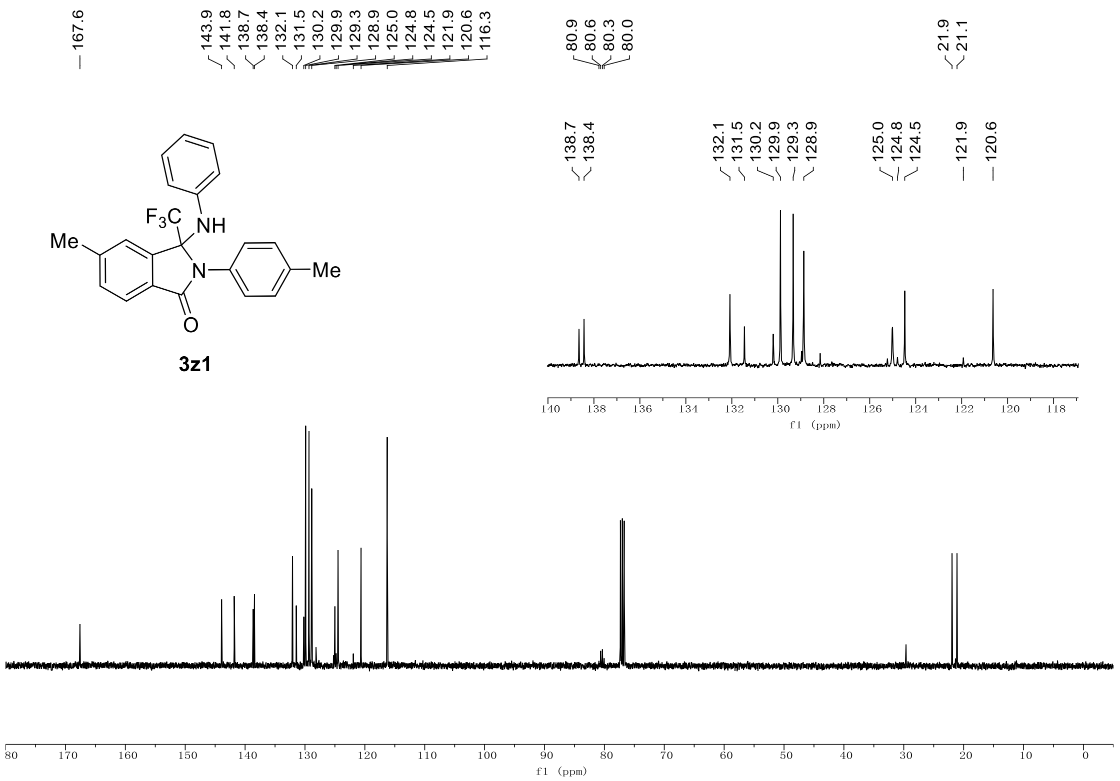

Figure S197. ^{13}C NMR (101 MHz, $CDCl_3$) spectrum of compound 3z1, related to Scheme 2

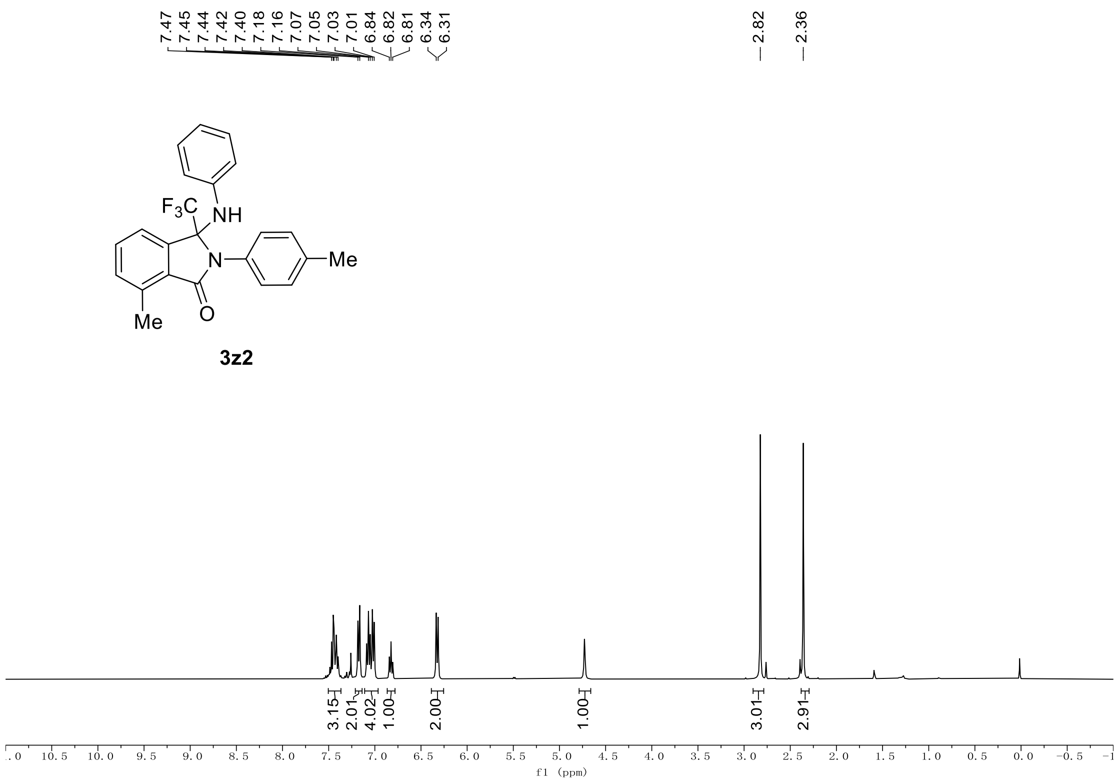

Figure S198. ^{1}H NMR (300 MHz, $CDCl_3$) spectrum of compound 3z2, related to Scheme 2

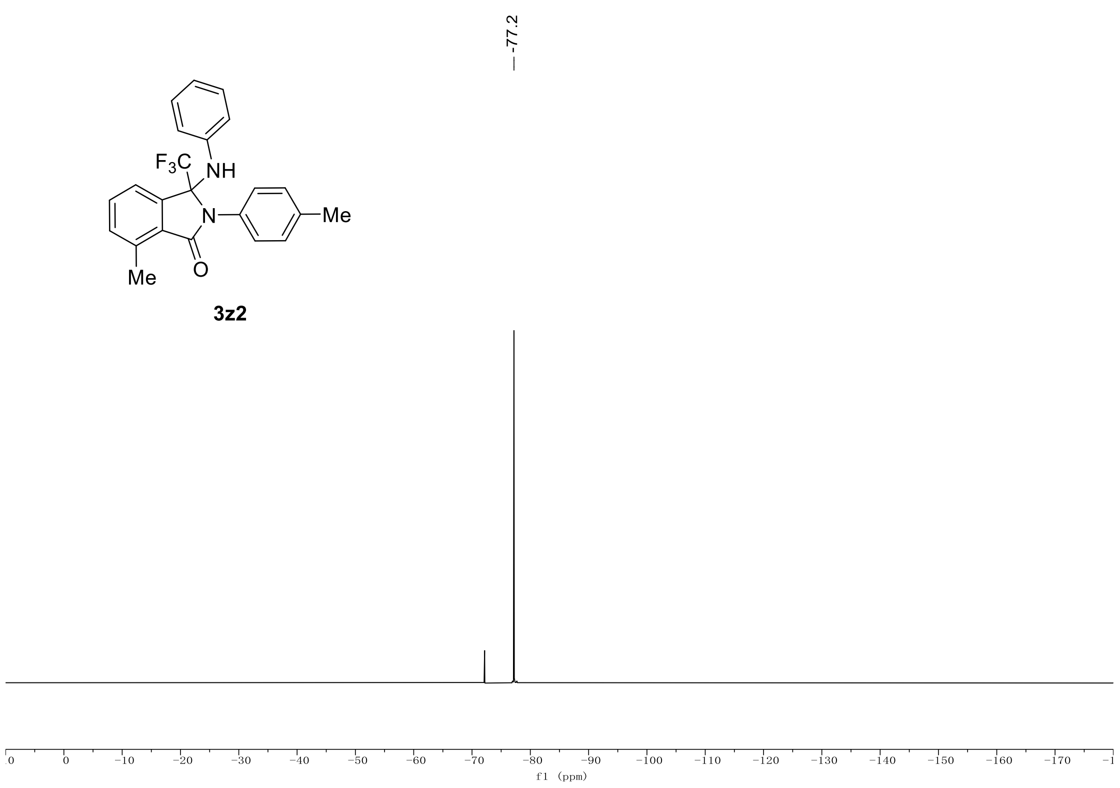

Figure S199. **^{19}F NMR (376 MHz, CDCl$_3$) spectrum of compound 3z2, related to Scheme 2**

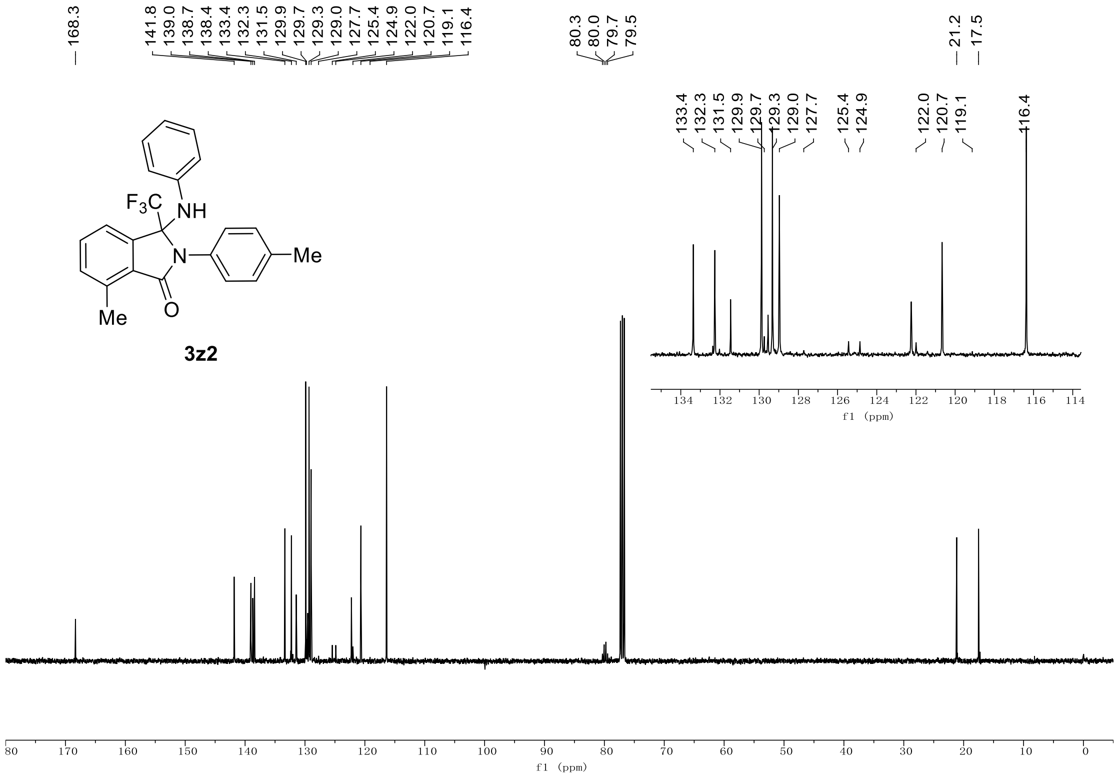

Figure S200. **^{13}C NMR (101 MHz, CDCl$_3$) spectrum of compound 3z2, related to Scheme 2**

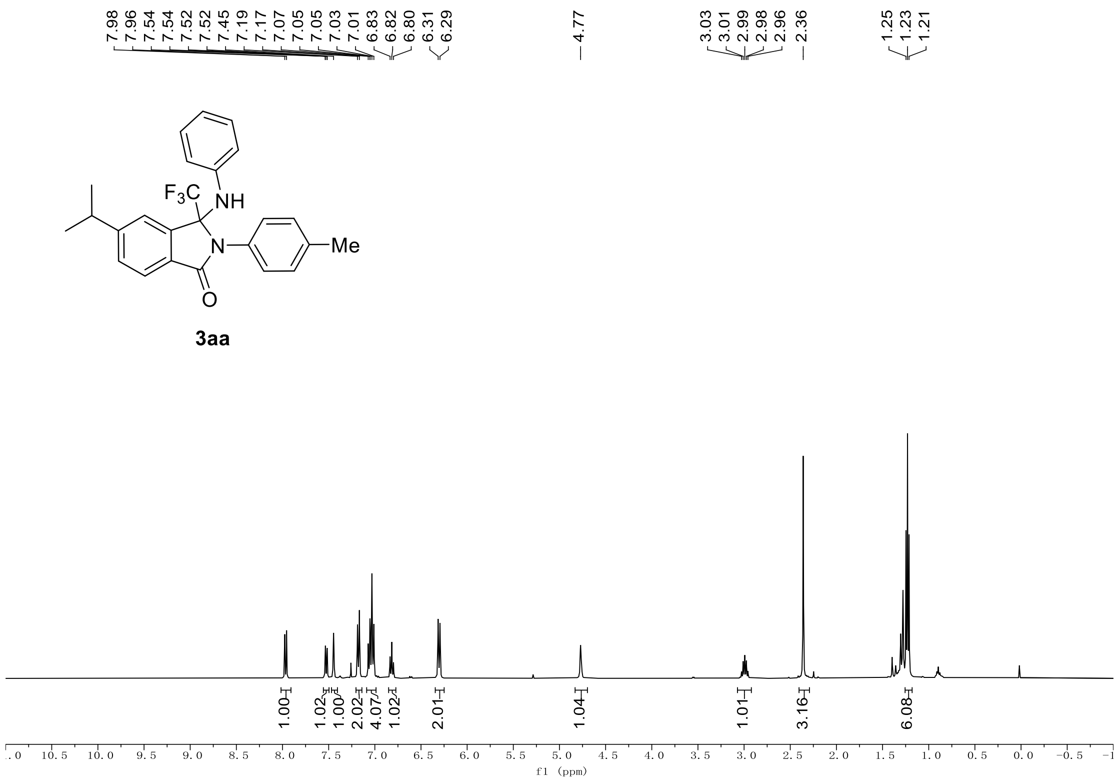

Figure S201. ^{1}H NMR (400 MHz, $CDCl_3$) spectrum of compound 3aa, related to Scheme 2

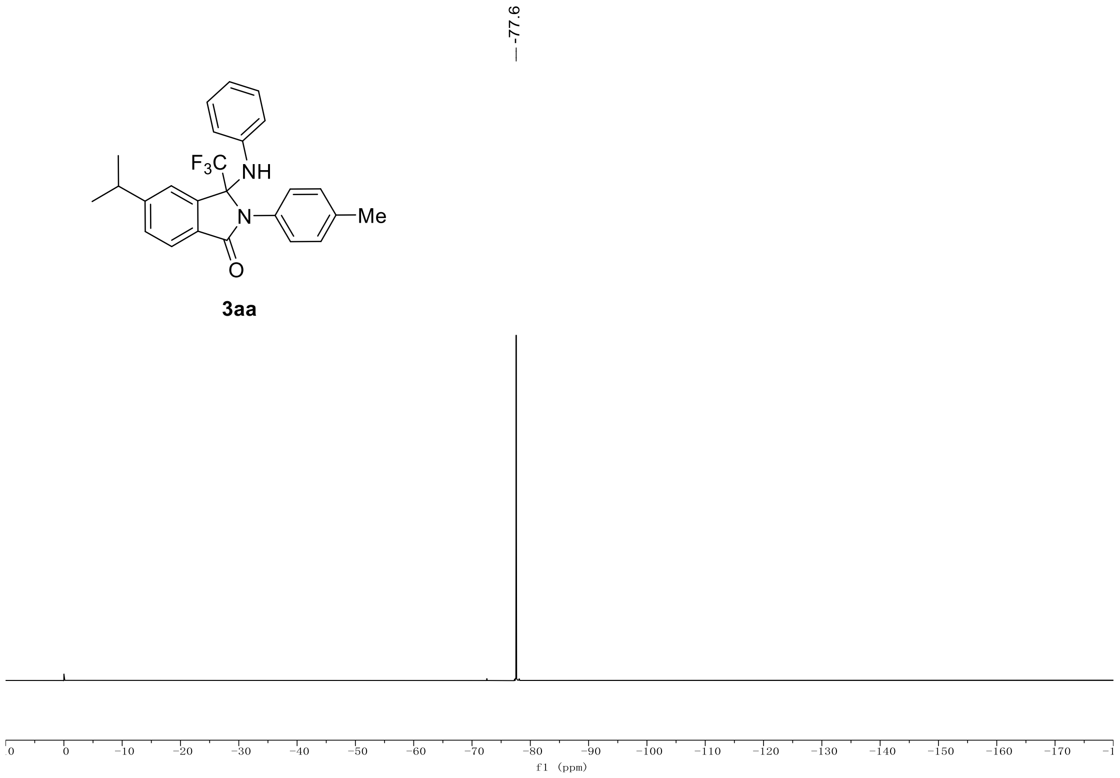

Figure S202. ^{19}F NMR (376 MHz, $CDCl_3$) spectrum of compound 3aa, related to Scheme 2

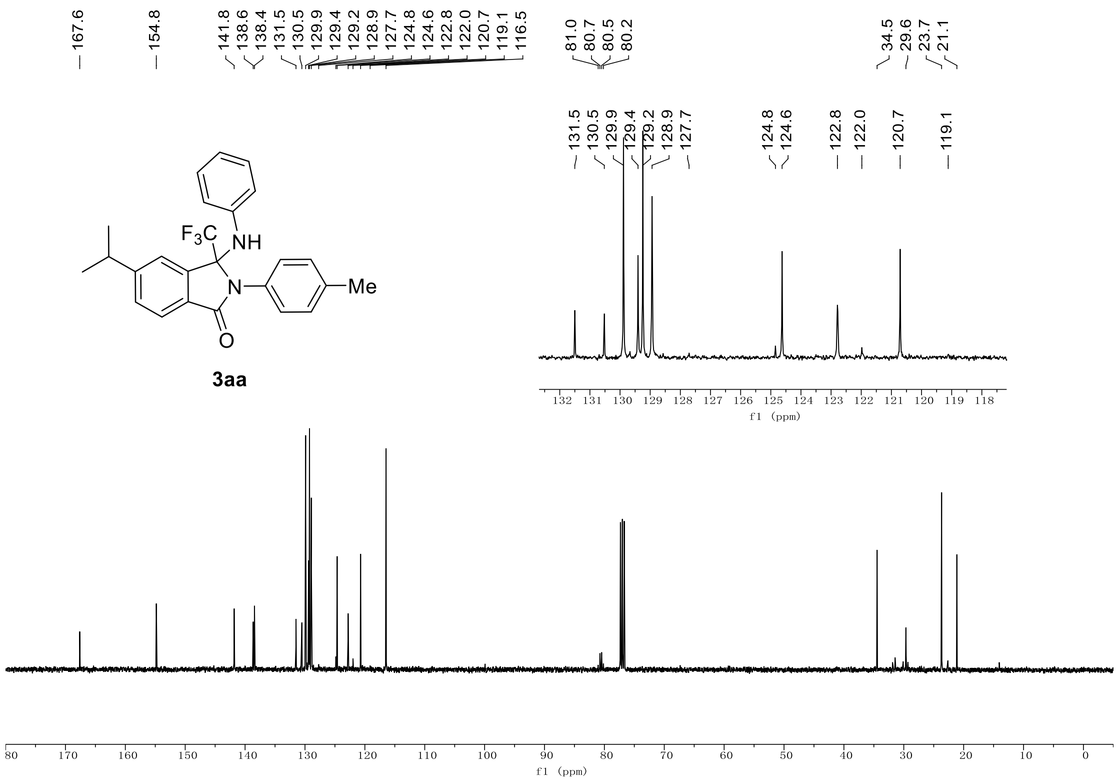

Figure S203. ^{13}C NMR (101 MHz, $CDCl_3$) spectrum of compound 3aa, related to Scheme 2

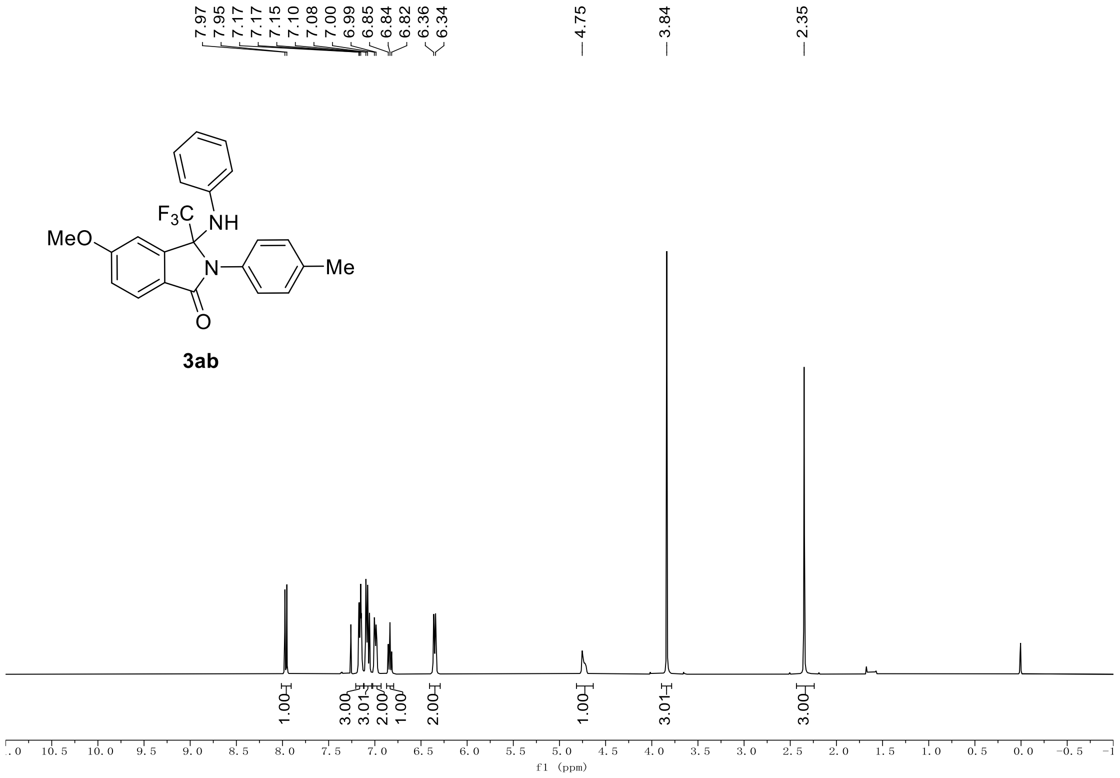

Figure S204. ^{1}H NMR (400 MHz, $CDCl_3$) spectrum of compound 3ab, related to Scheme 2

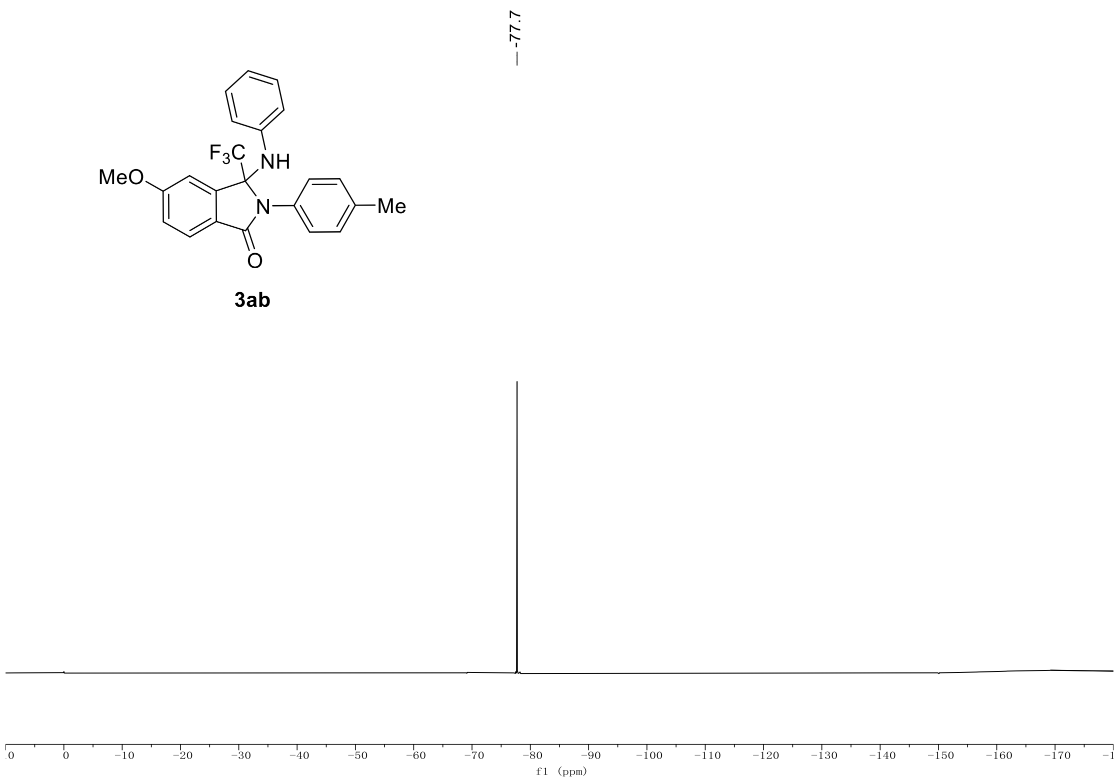

Figure S205. ^{19}F NMR (376 MHz, CDCl₃) spectrum of compound 3ab, related to Scheme 2

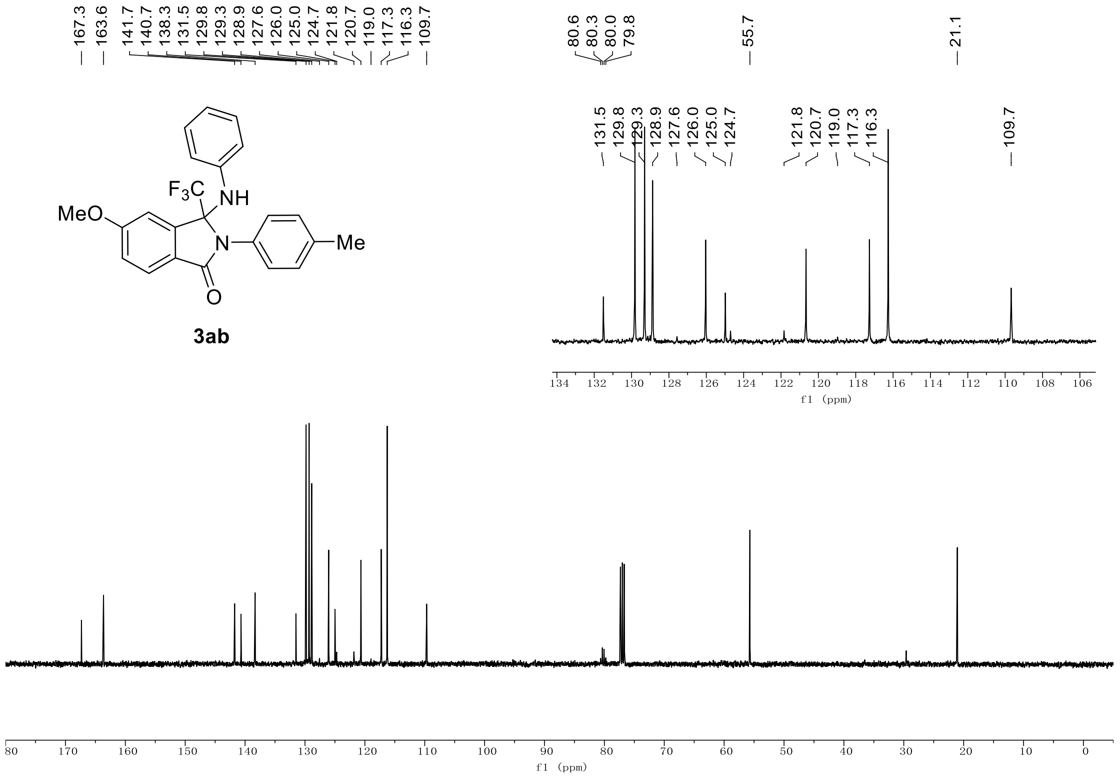

Figure S206. ^{13}C NMR (101 MHz, CDCl₃) spectrum of compound 3ab, related to Scheme 2

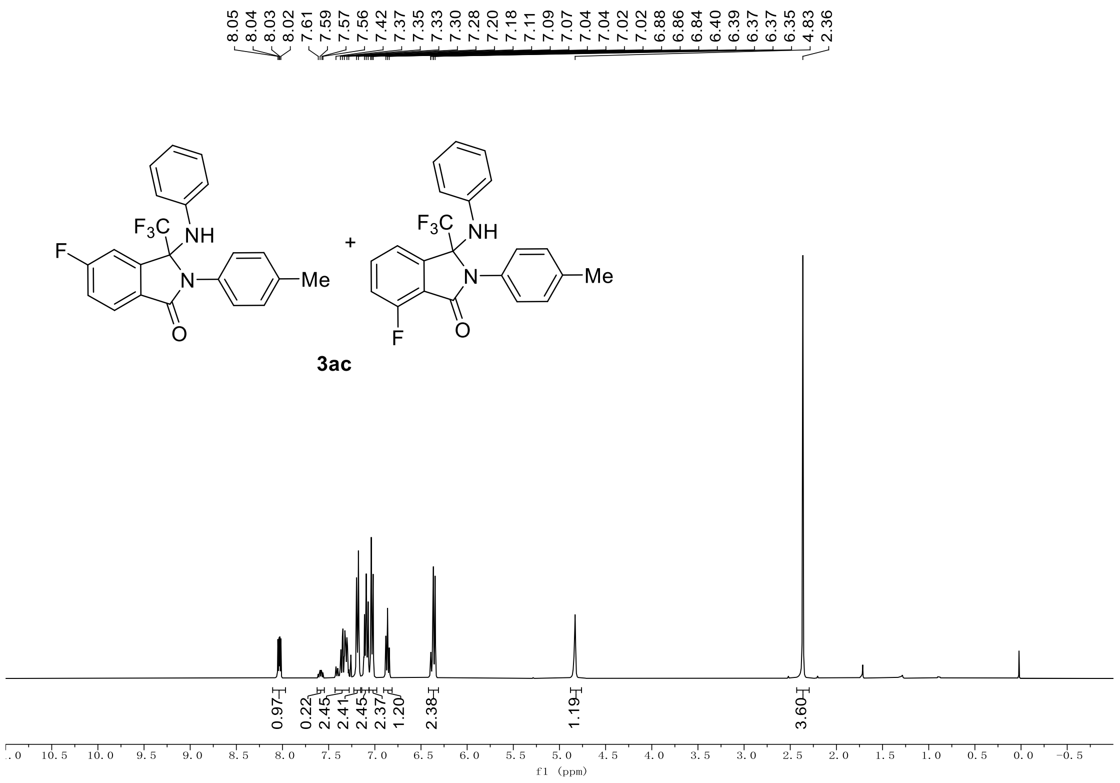

Figure S207. ^{1}**H NMR (400 MHz,** $CDCl_3$**) spectrum of compound 3ac, related to Scheme 2**

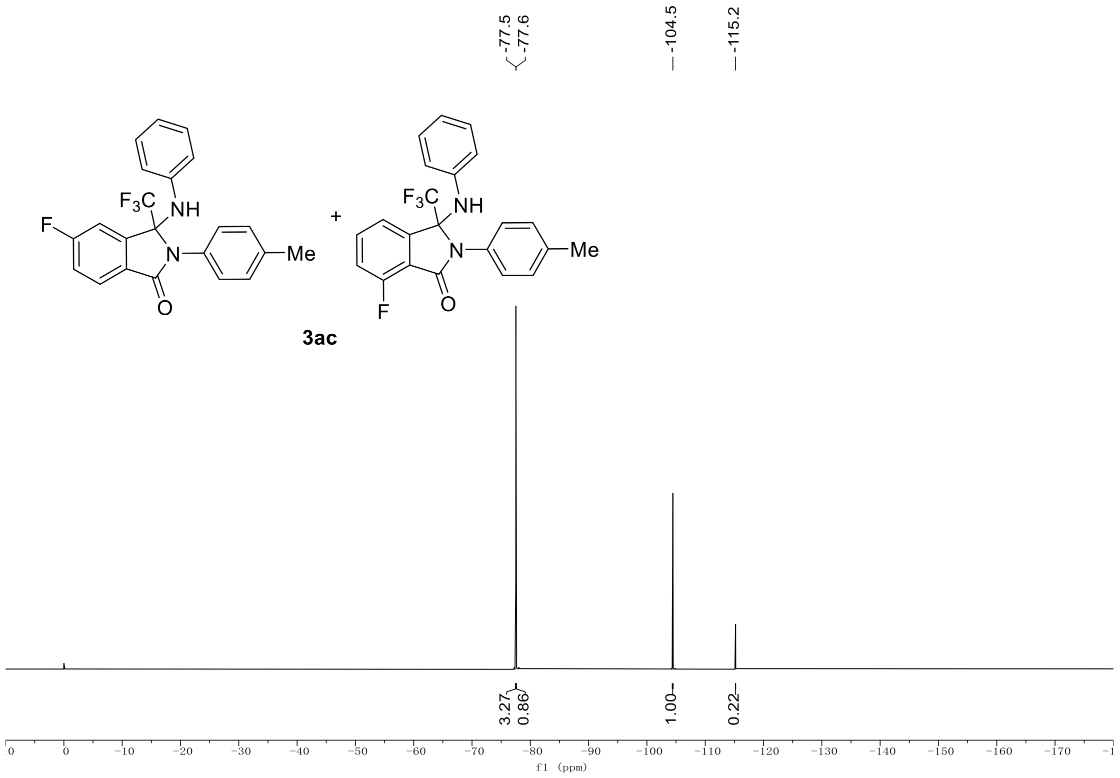

Figure S208. ^{19}**F NMR (376 MHz,** $CDCl_3$**) spectrum of compound 3ac, related to Scheme 2**

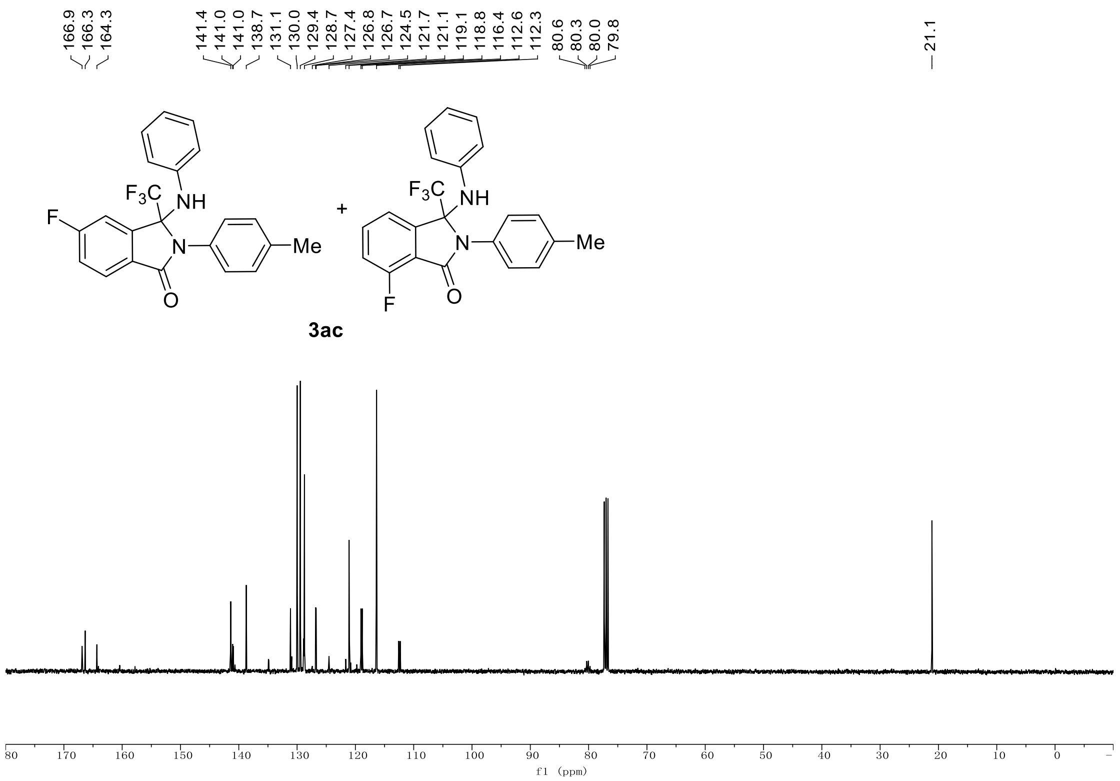

Figure S209. ^{13}**C NMR (101 MHz, CDCl$_3$) spectrum of compound 3ac, related to Scheme 2**

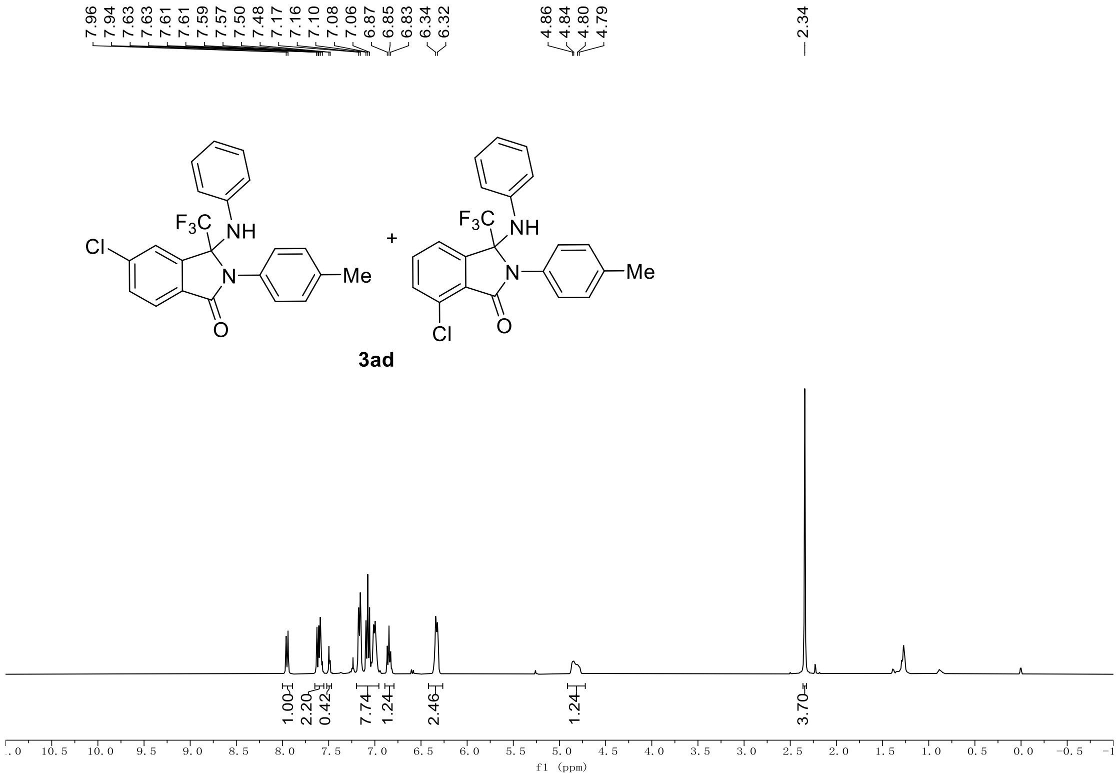

Figure S210. ^{1}**H NMR (400 MHz, CDCl$_3$) spectrum of compound 3ad, related to Scheme 2**

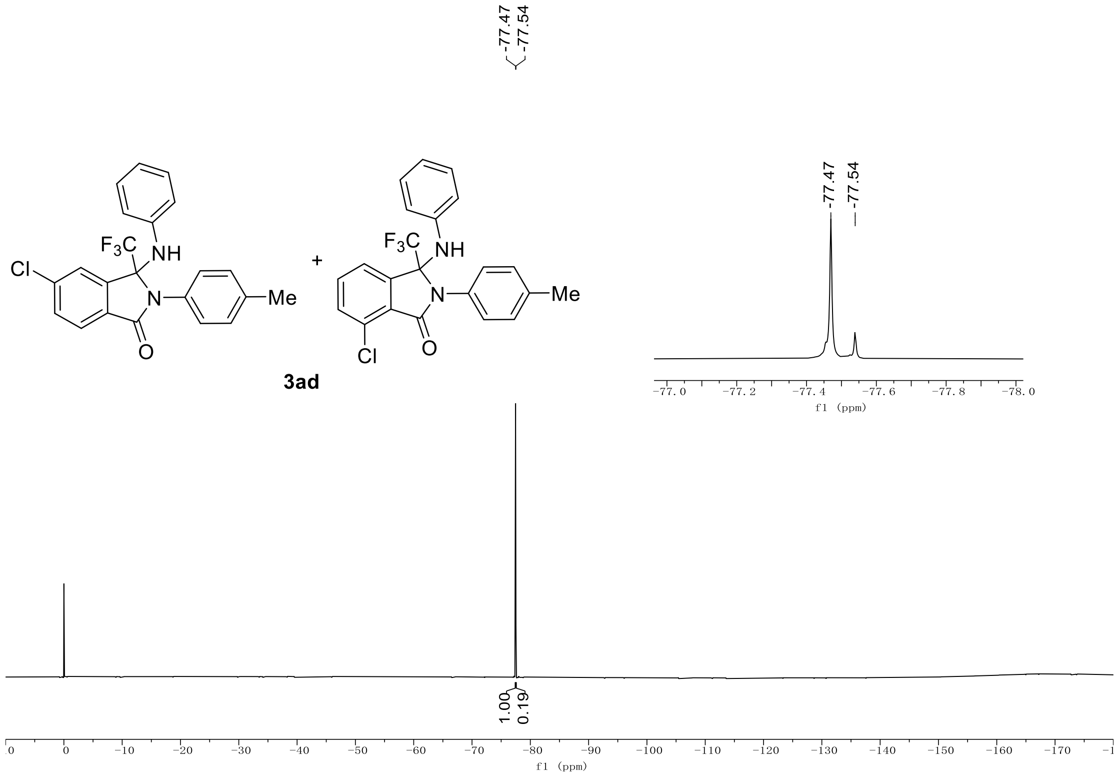

Figure S211. ^{19}F NMR (376 MHz, CDCl$_3$) spectrum of compound 3ad, related to Scheme 2

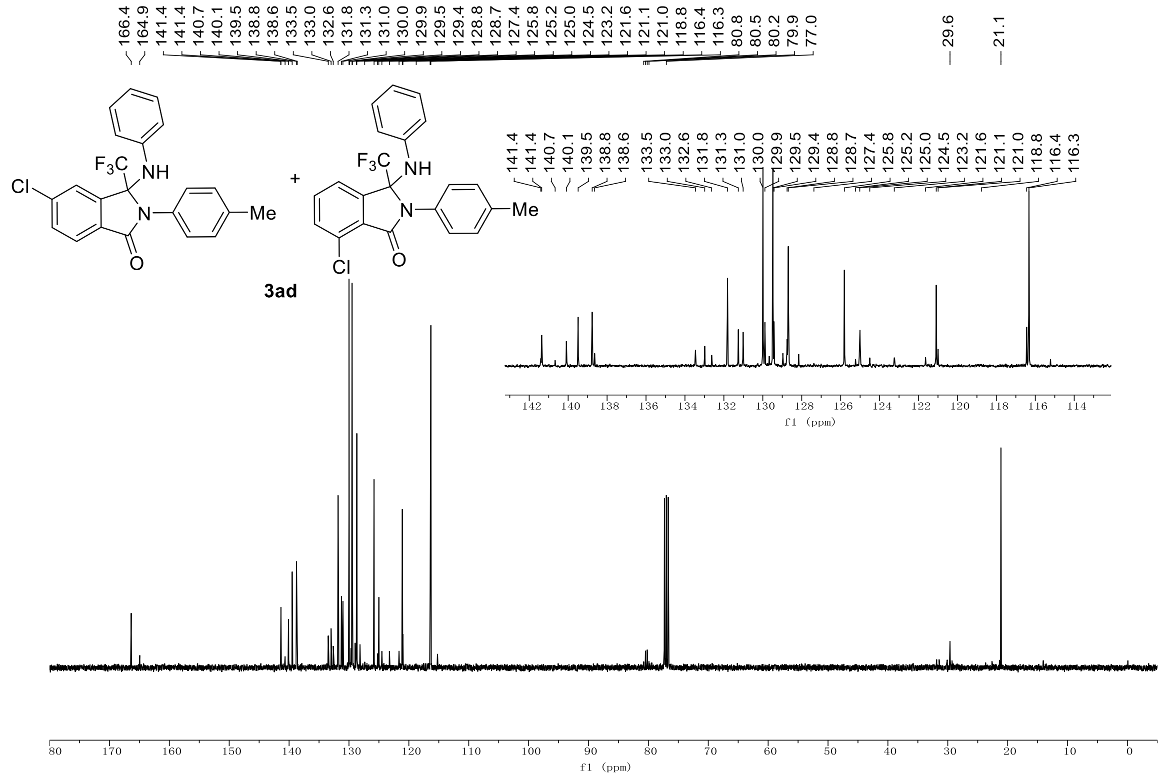

Figure S212. ^{13}C NMR (101 MHz, CDCl$_3$) spectrum of compound 3ad, related to Scheme 2

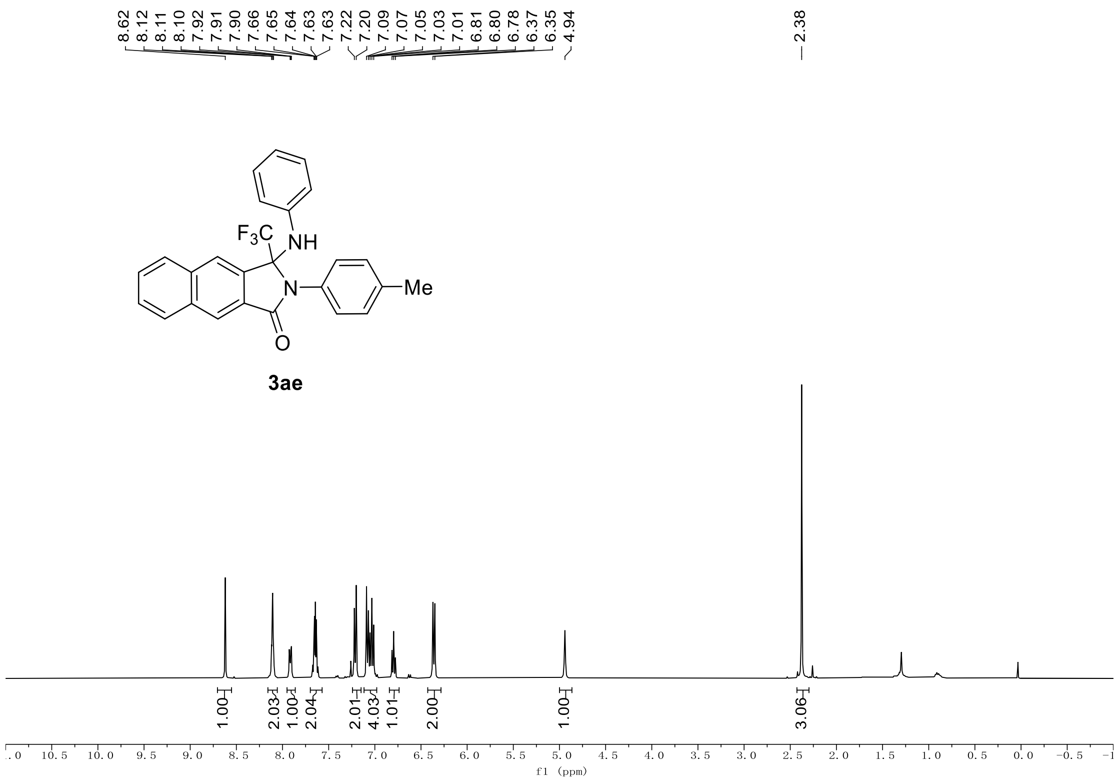

Figure S213. ¹H NMR (400 MHz, CDCl₃) spectrum of compound 3ae, related to Scheme 2

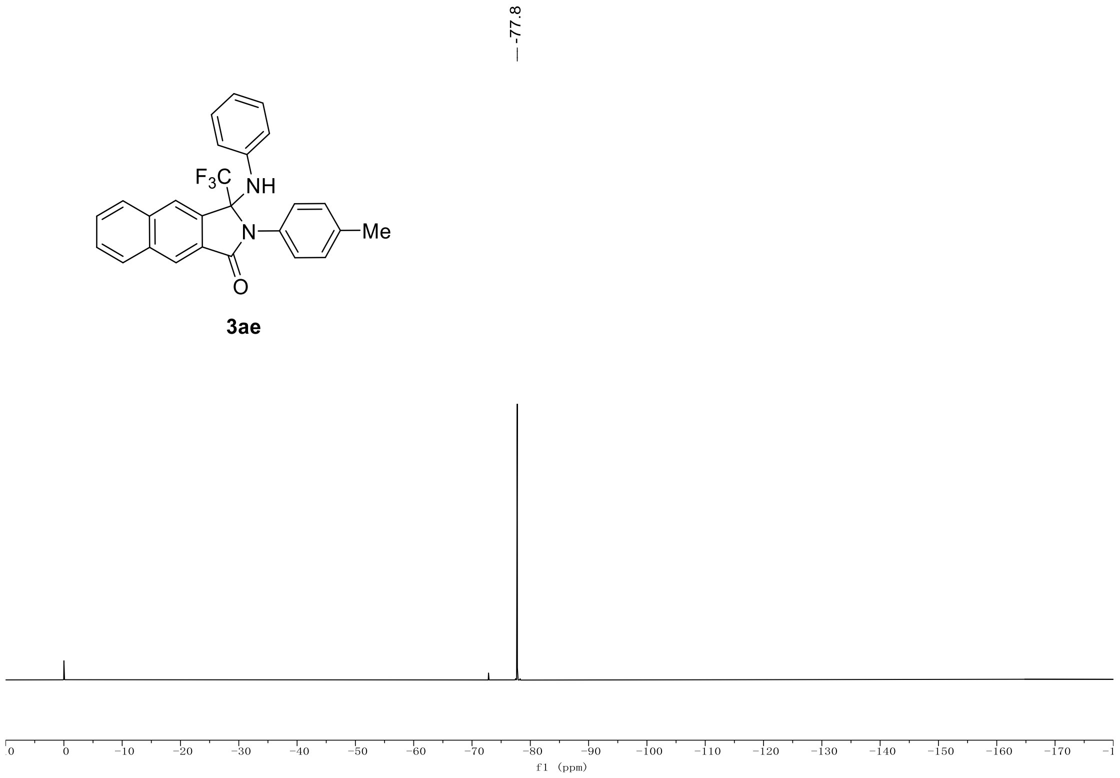

Figure S214. ¹⁹F NMR (376 MHz, CDCl₃) spectrum of compound 3ae, related to Scheme 2

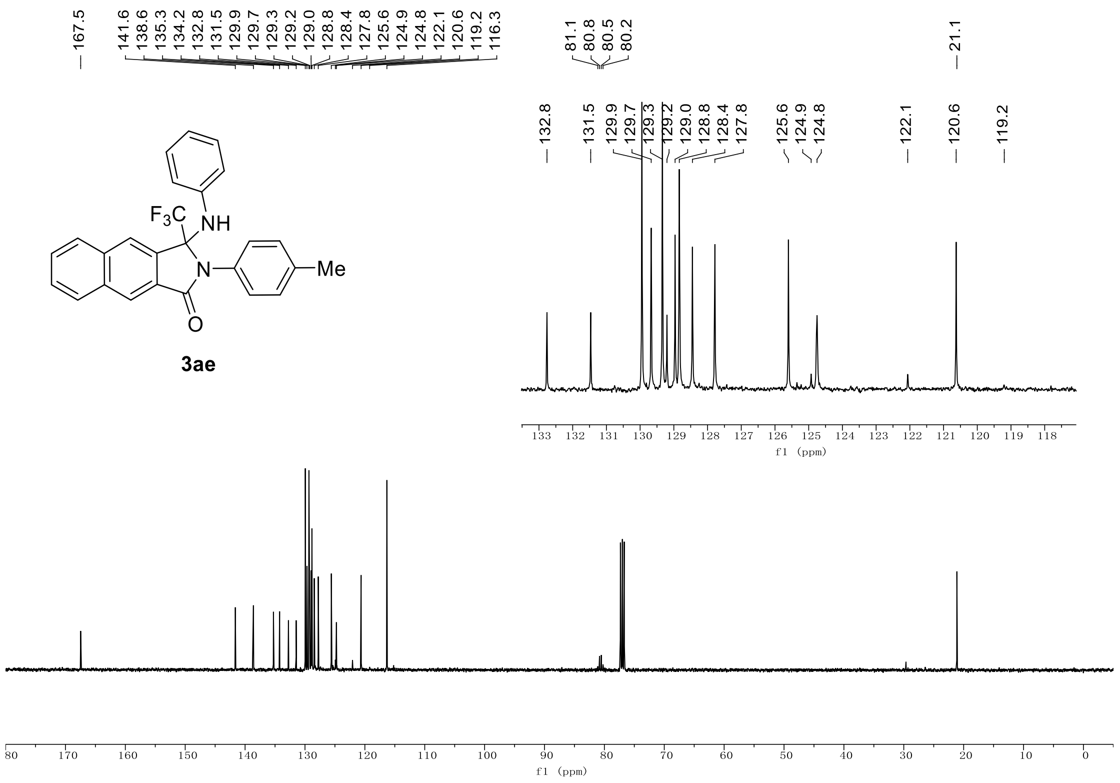

Figure S215. ^{13}C NMR (101 MHz, $CDCl_3$) spectrum of compound 3ae, related to Scheme 2

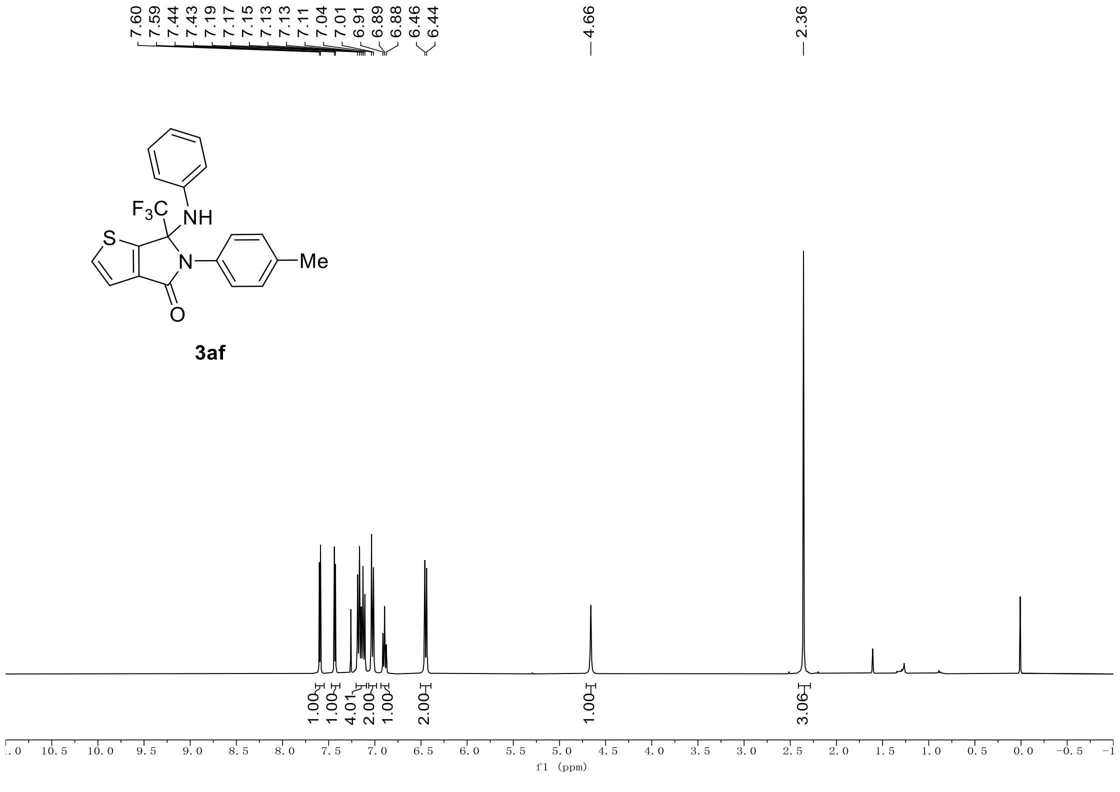

Figure S216. ^{1}H NMR (400 MHz, $CDCl_3$) spectrum of compound 3af, related to Scheme 2

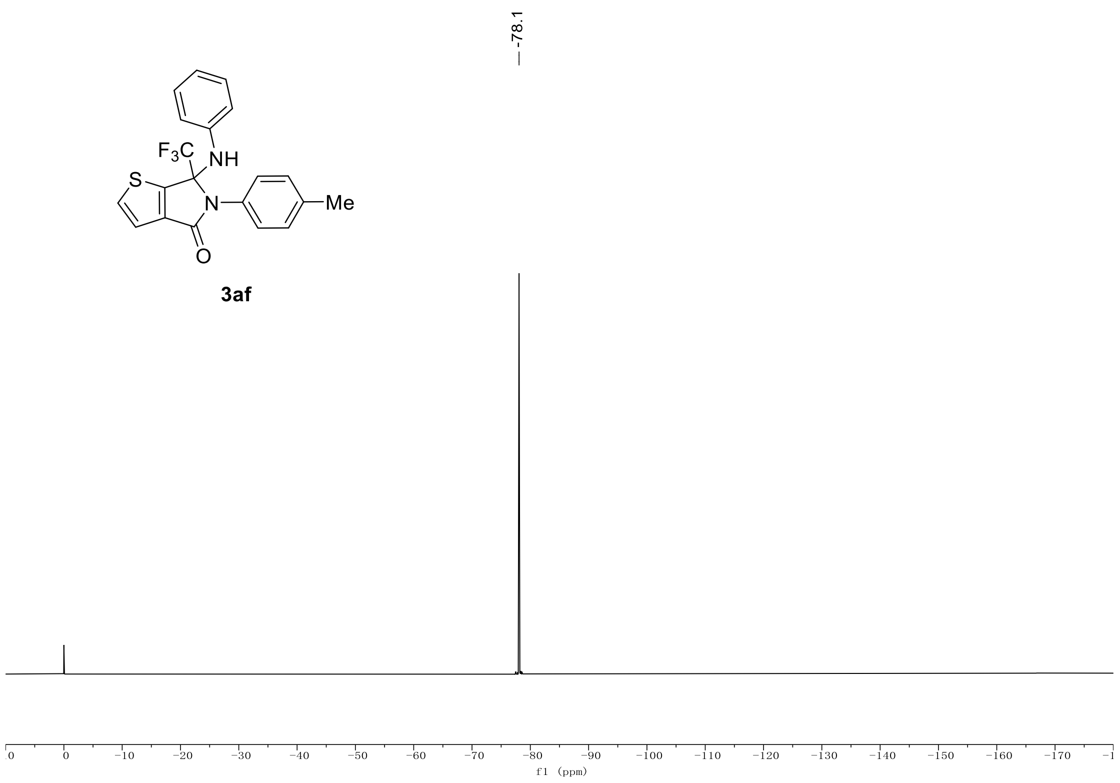

Figure S217. ^{19}**F NMR (376 MHz, CDCl$_3$) spectrum of compound 3af, related to Scheme 2**

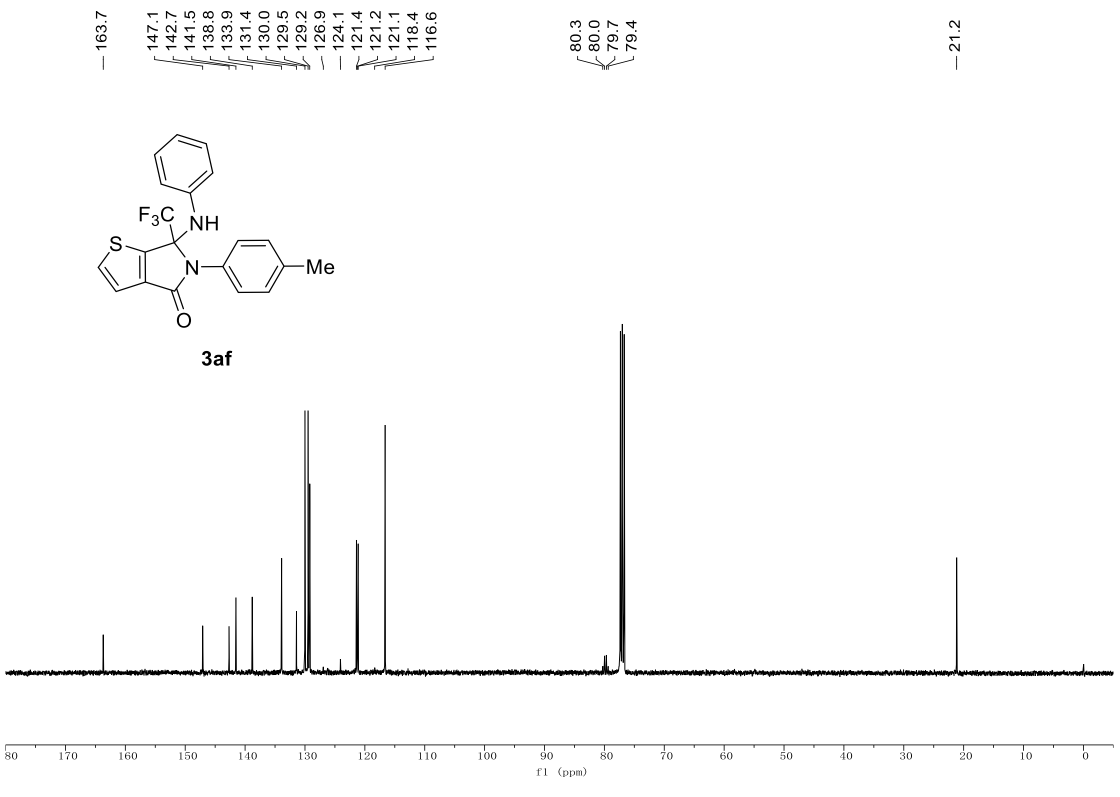

Figure S218. ^{13}**C NMR (101 MHz, CDCl$_3$) spectrum of compound 3af, related to Scheme 2**

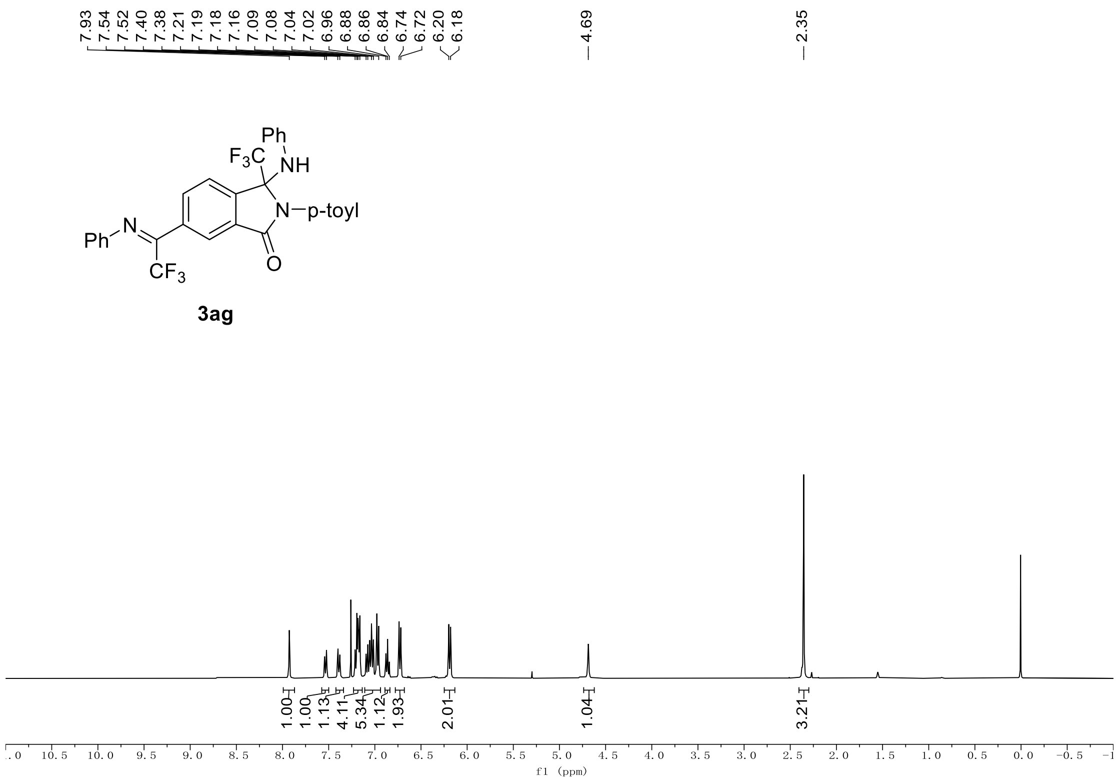

Figure S219. ^{1}H NMR (400 MHz, $CDCl_3$) spectrum of compound 3ag, related to Scheme 2

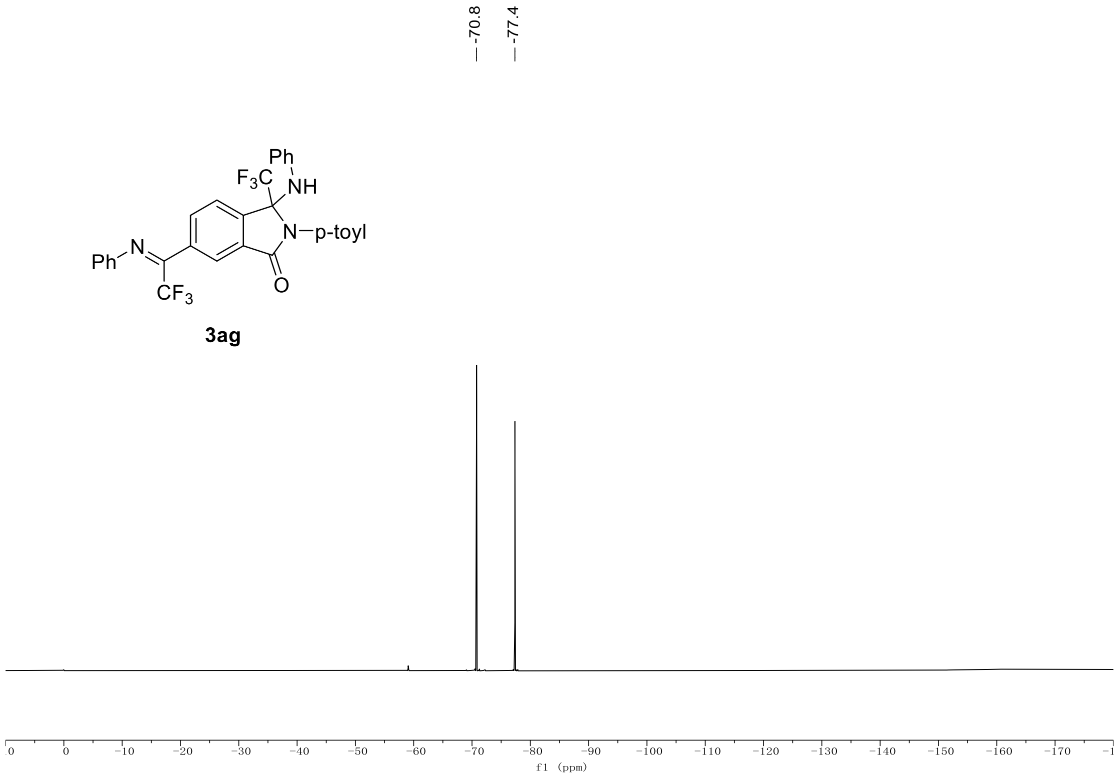

Figure S220. ^{19}F NMR (376 MHz, $CDCl_3$) spectrum of compound 3ag, related to Scheme 2

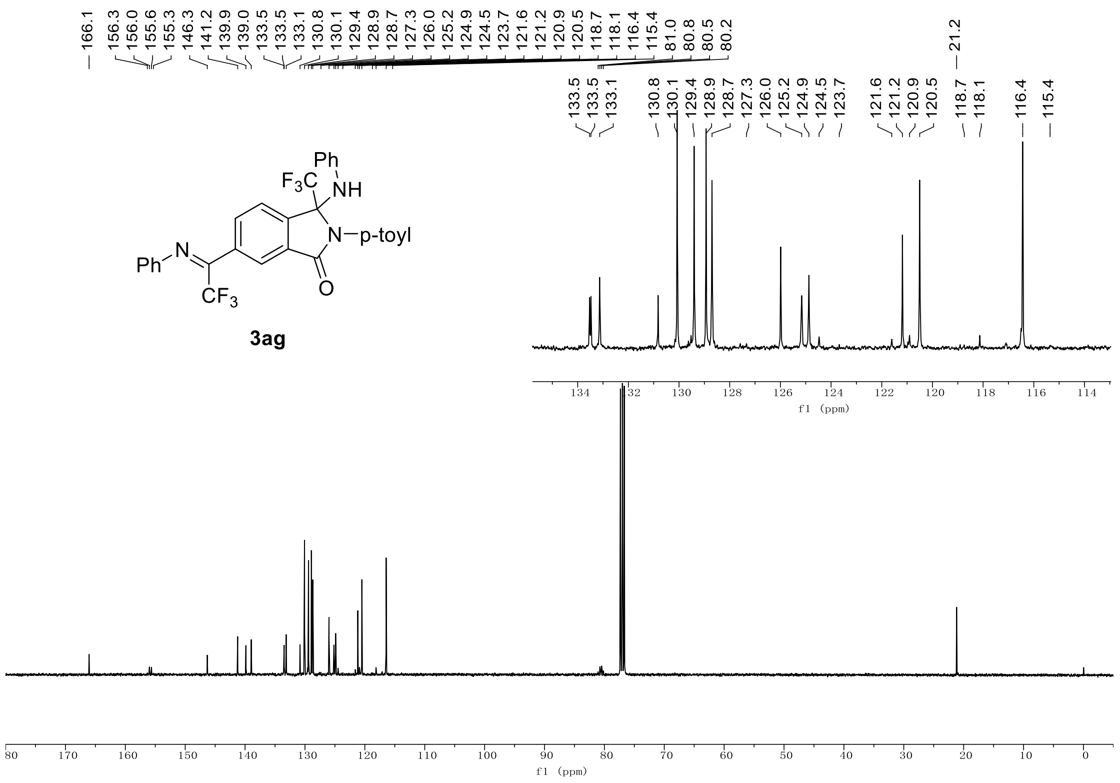

Figure S221. ^{13}C NMR (101 MHz, $CDCl_3$) spectrum of compound 3ag, related to Scheme 2

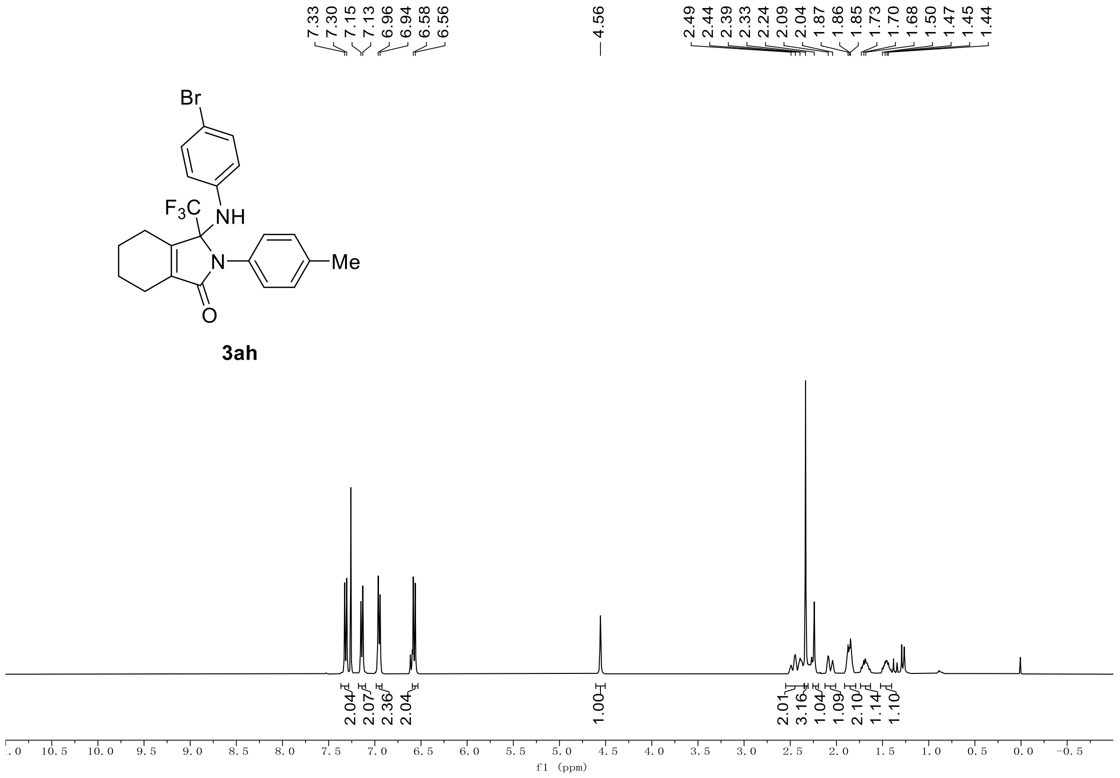

Figure S222. ^{1}H NMR (400 MHz, $CDCl_3$) spectrum of compound 3ah, related to Scheme 2

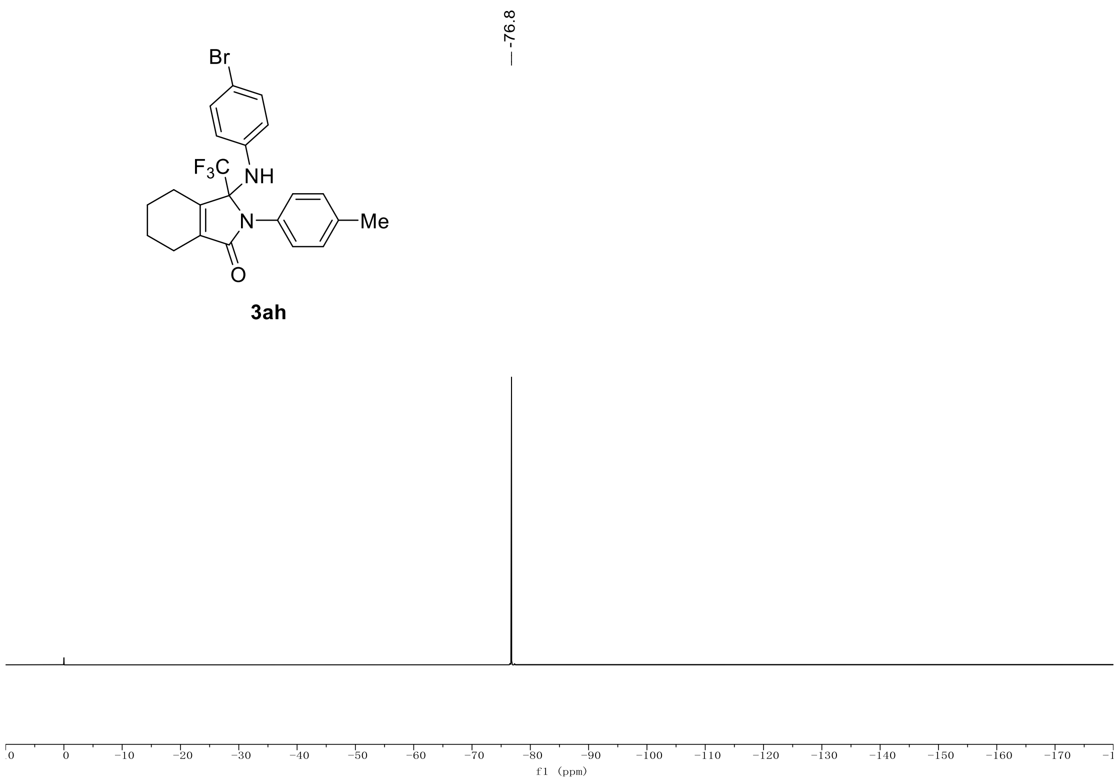

Figure S223. ^{19}F NMR (376 MHz, CDCl$_3$) spectrum of compound 3ah, related to Scheme 2

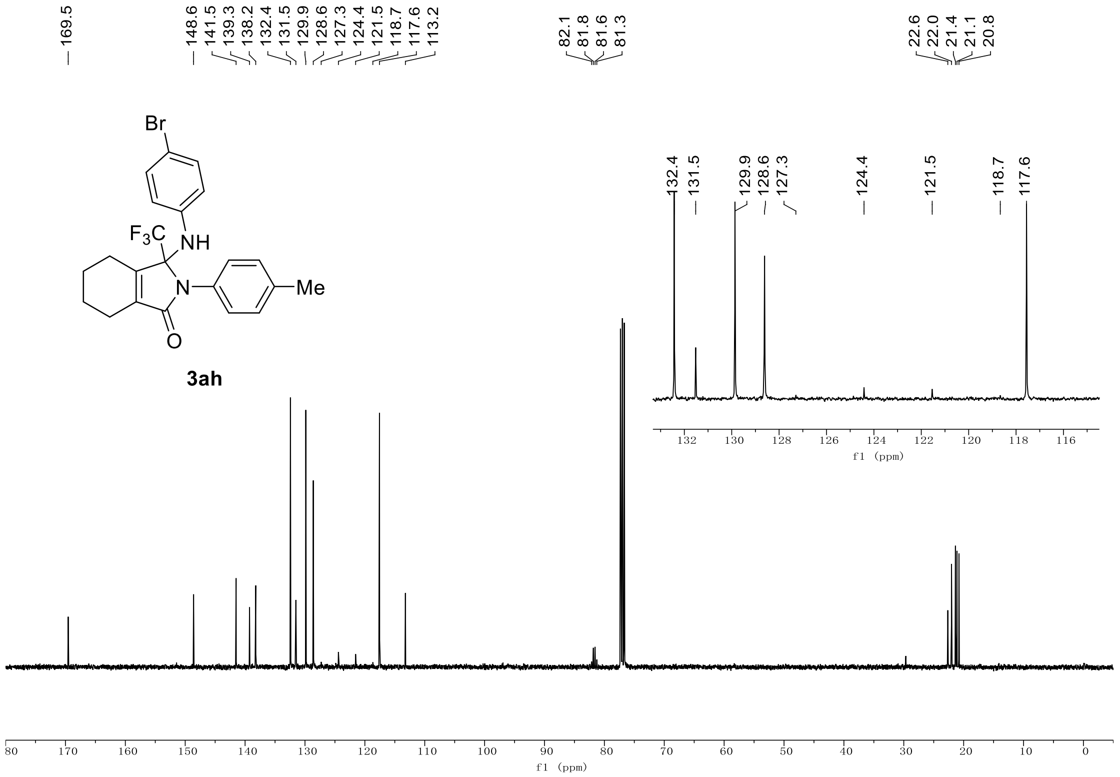

Figure S224. ^{13}C NMR (101 MHz, CDCl$_3$) spectrum of compound 3ah, related to Scheme 2

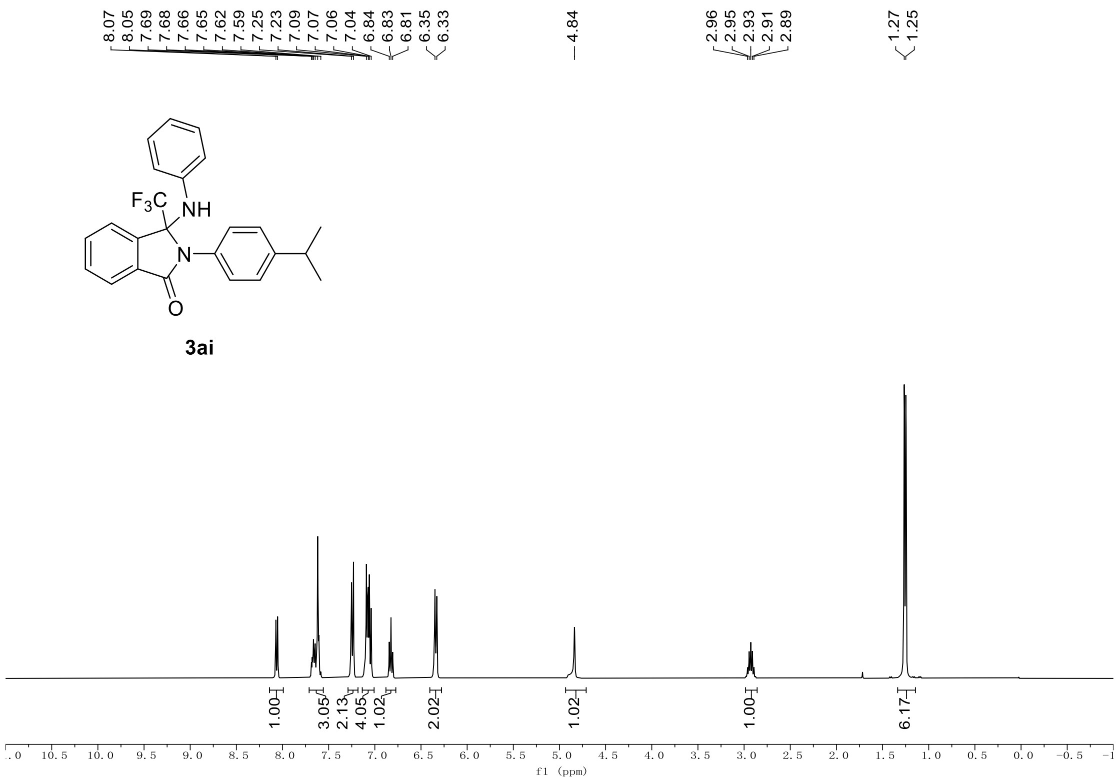

Figure S225. **^{1}H NMR (400 MHz, $CDCl_3$) spectrum of compound 3ai, related to Scheme 3**

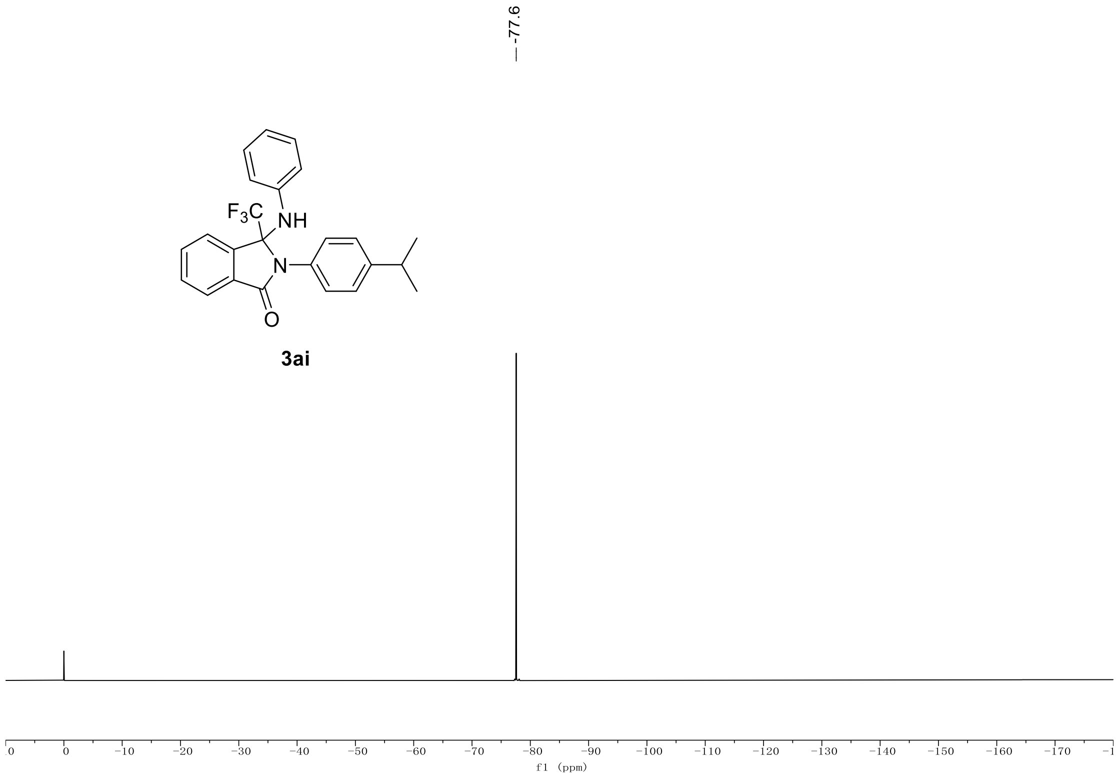

Figure S226. **^{19}F NMR (376 MHz, $CDCl_3$) spectrum of compound 3ai, related to Scheme 3**

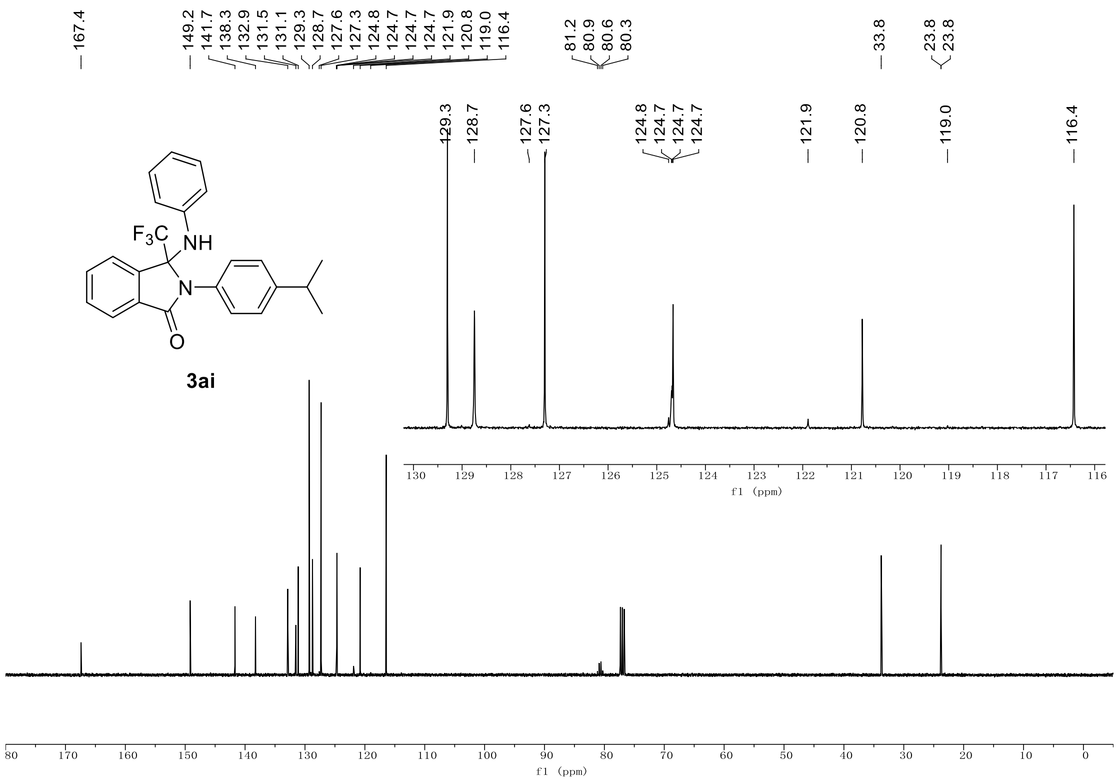

Figure S227. ^{13}C NMR (101 MHz, $CDCl_3$) spectrum of compound 3ai, related to Scheme 3

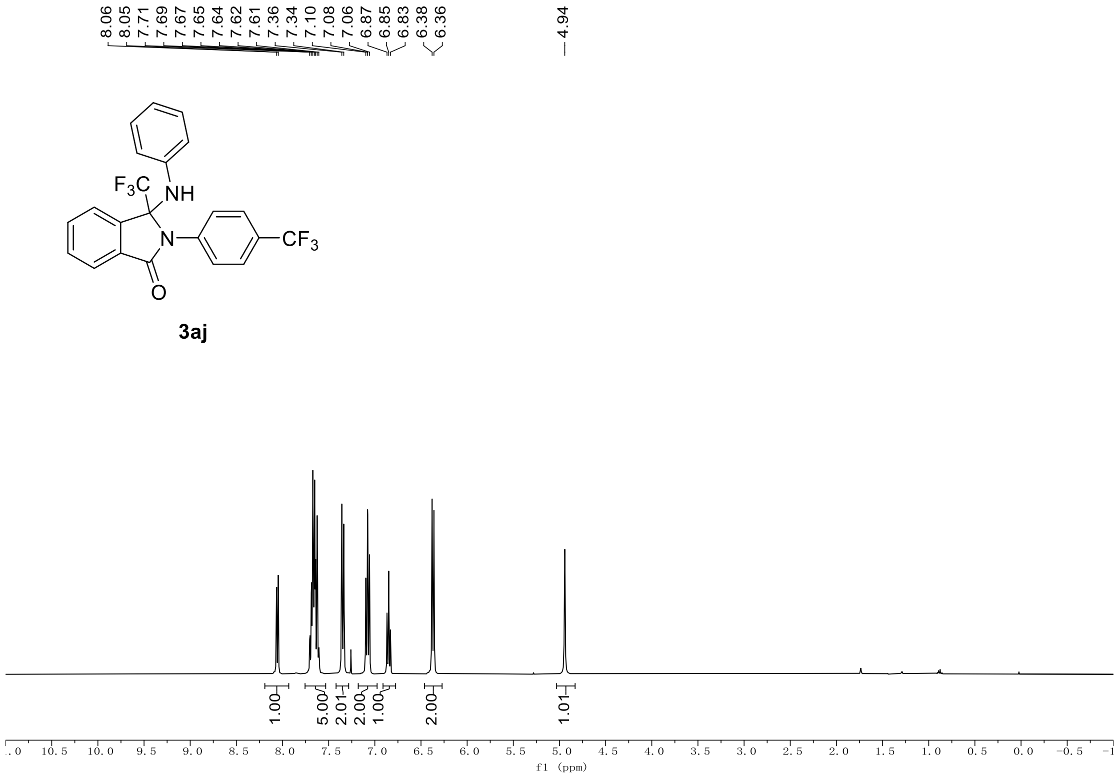

Figure S228. ^{1}H NMR (400 MHz, $CDCl_3$) spectrum of compound 3aj, related to Scheme 3

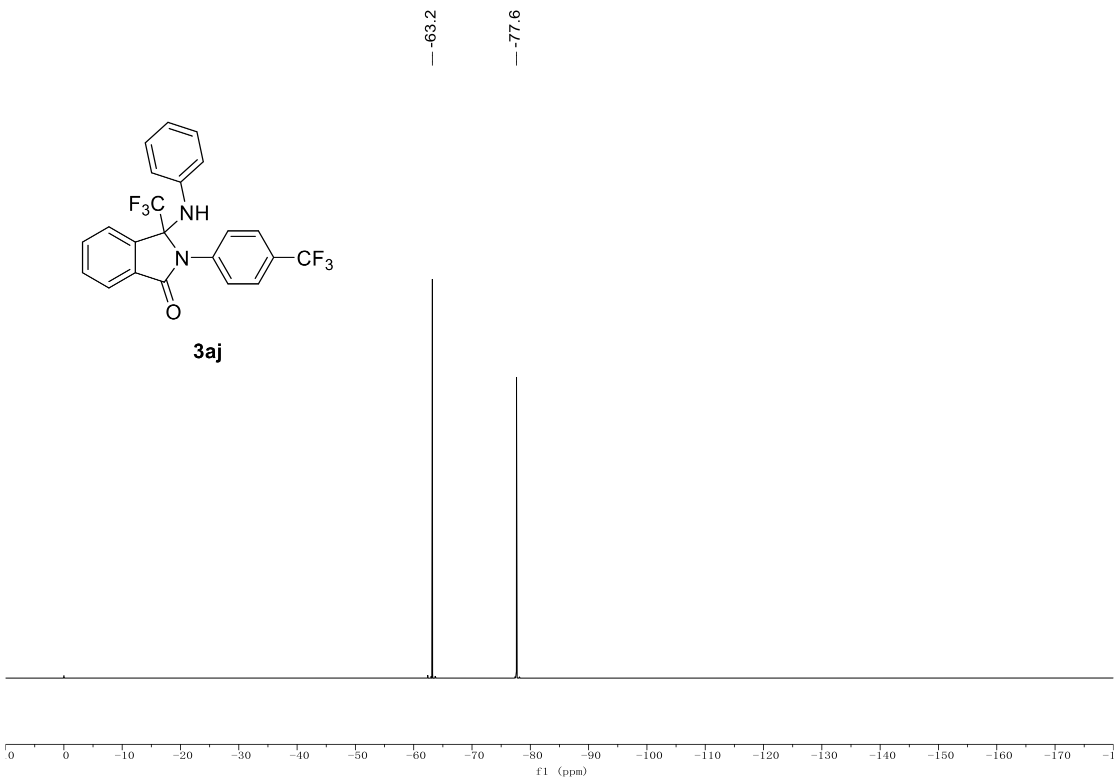

Figure S229. ^{19}F NMR (376 MHz, CDCl₃) spectrum of compound 3aj, related to Scheme 3

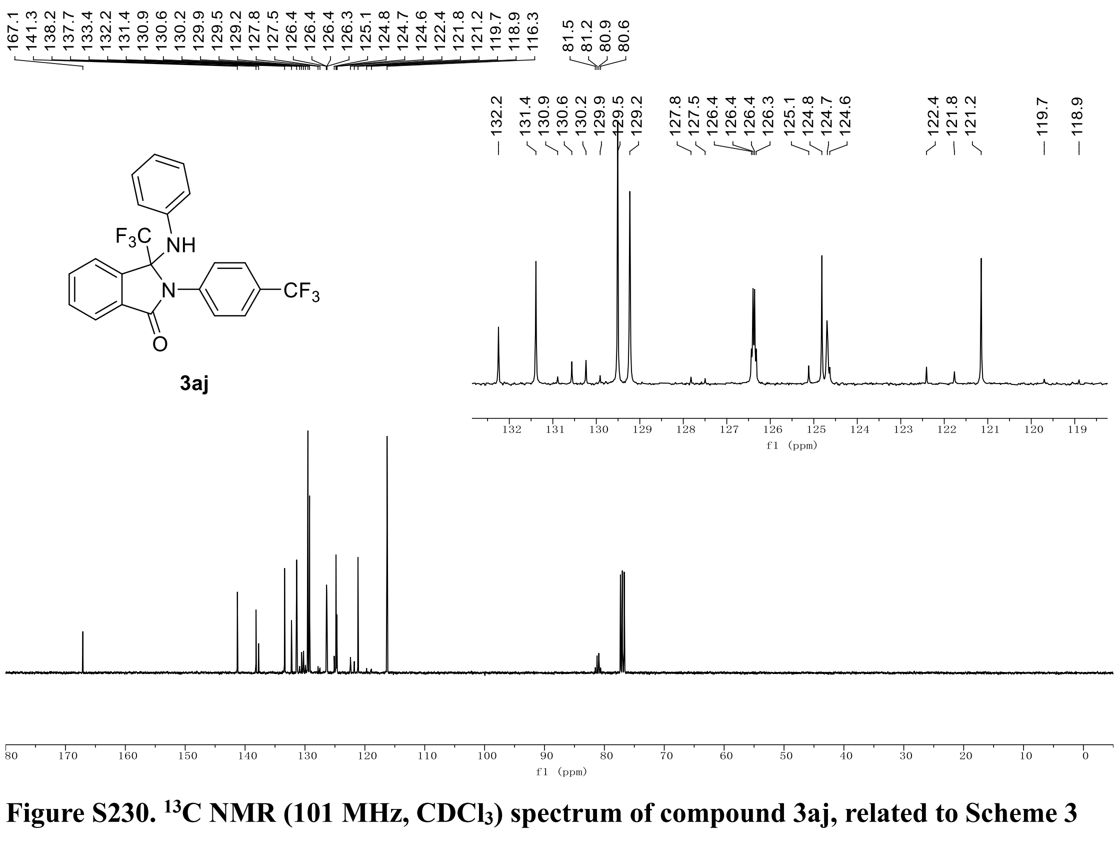

Figure S230. ^{13}C NMR (101 MHz, CDCl₃) spectrum of compound 3aj, related to Scheme 3

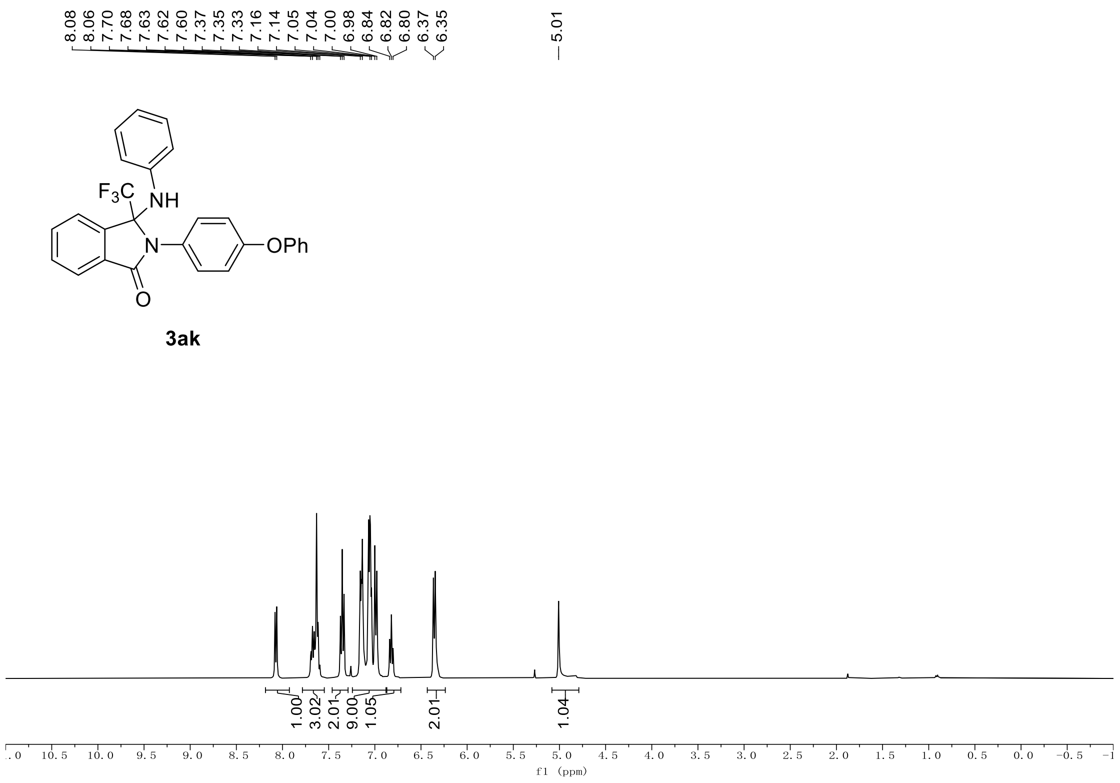

Figure S231. ^{1}H NMR (400 MHz, $CDCl_3$) spectrum of compound 3ak, related to Scheme 3

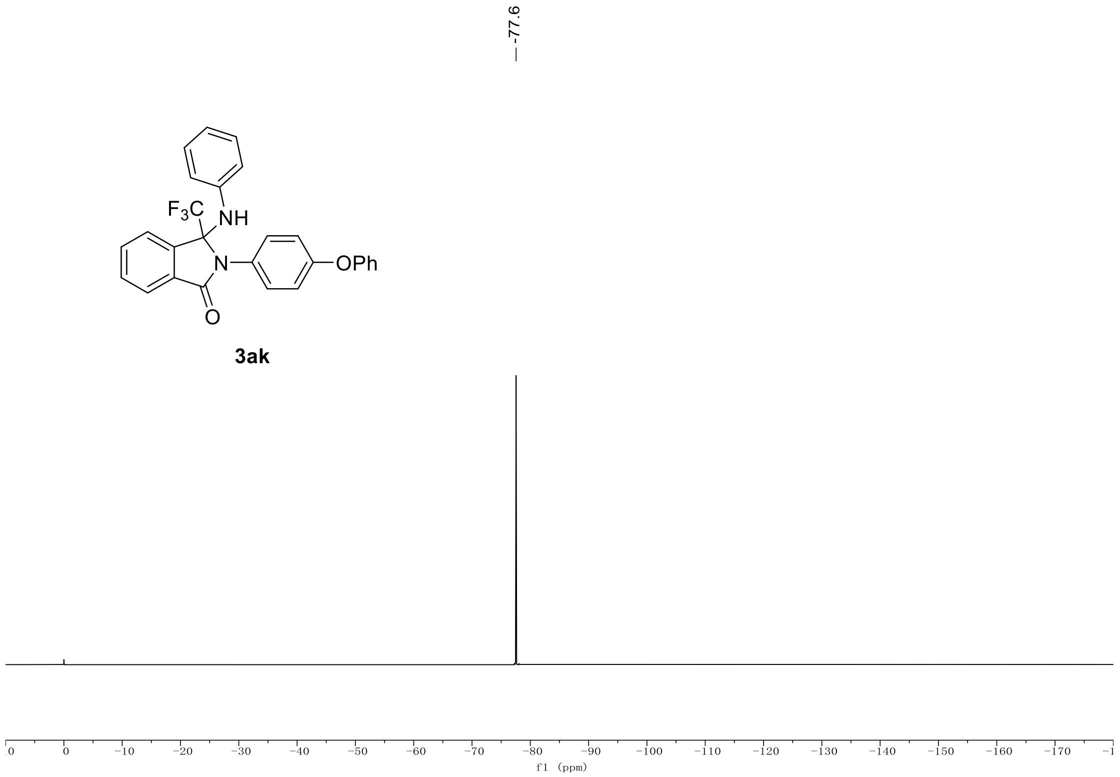

Figure S232. ^{19}F NMR (376 MHz, $CDCl_3$) spectrum of compound 3ak, related to Scheme 3

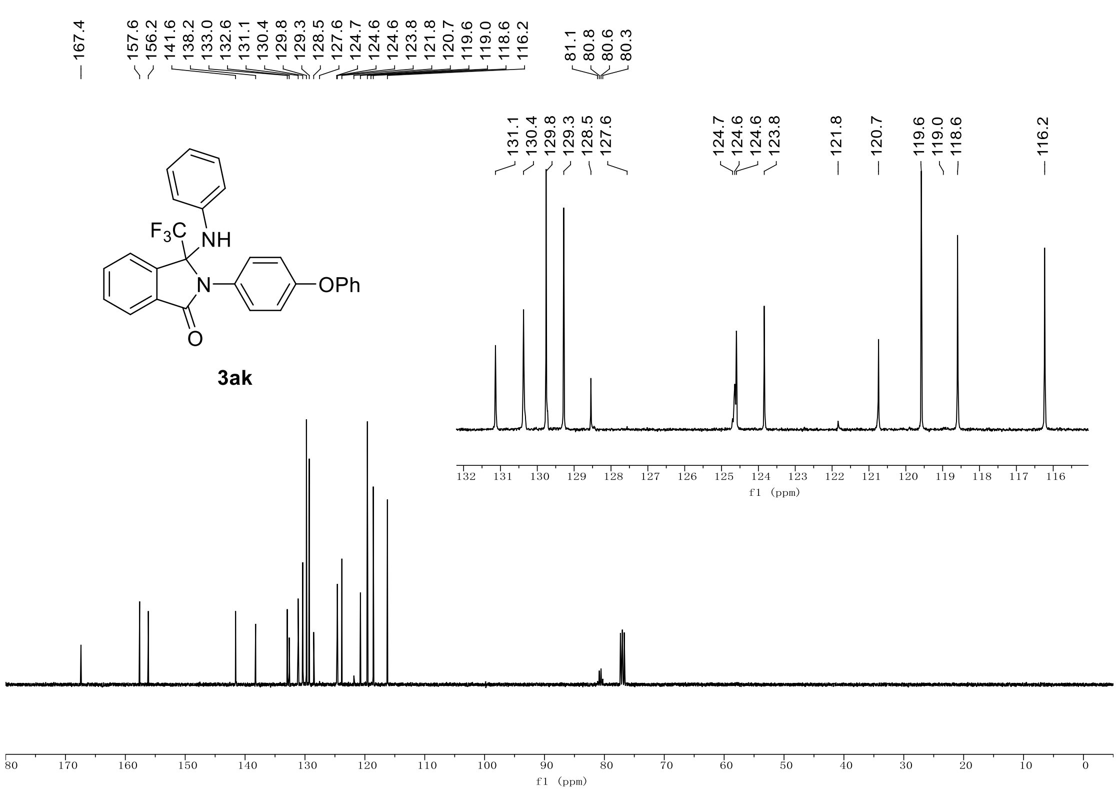

Figure S233. ^{13}C NMR (101 MHz, $CDCl_3$) spectrum of compound 3ak, related to Scheme 3

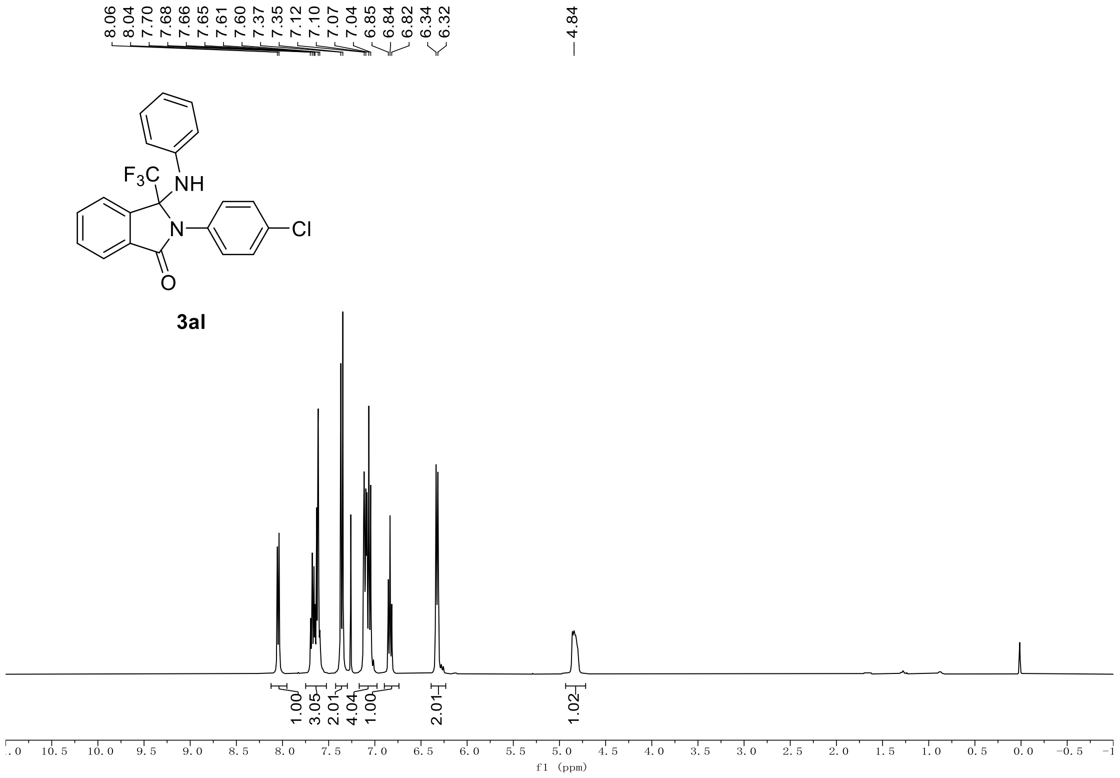

Figure S234. ^{1}H NMR (400 MHz, $CDCl_3$) spectrum of compound 3al, related to Scheme 3

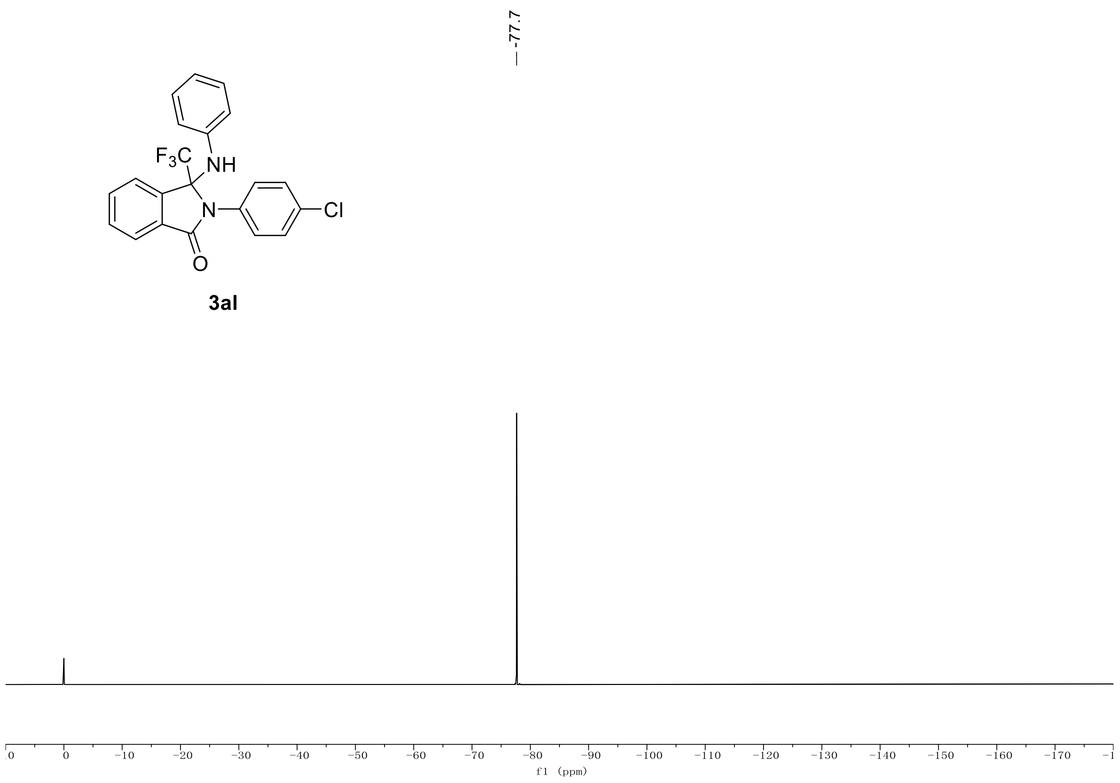

Figure S235. ^{19}F NMR (376 MHz, $CDCl_3$) spectrum of compound 3al, related to Scheme 3

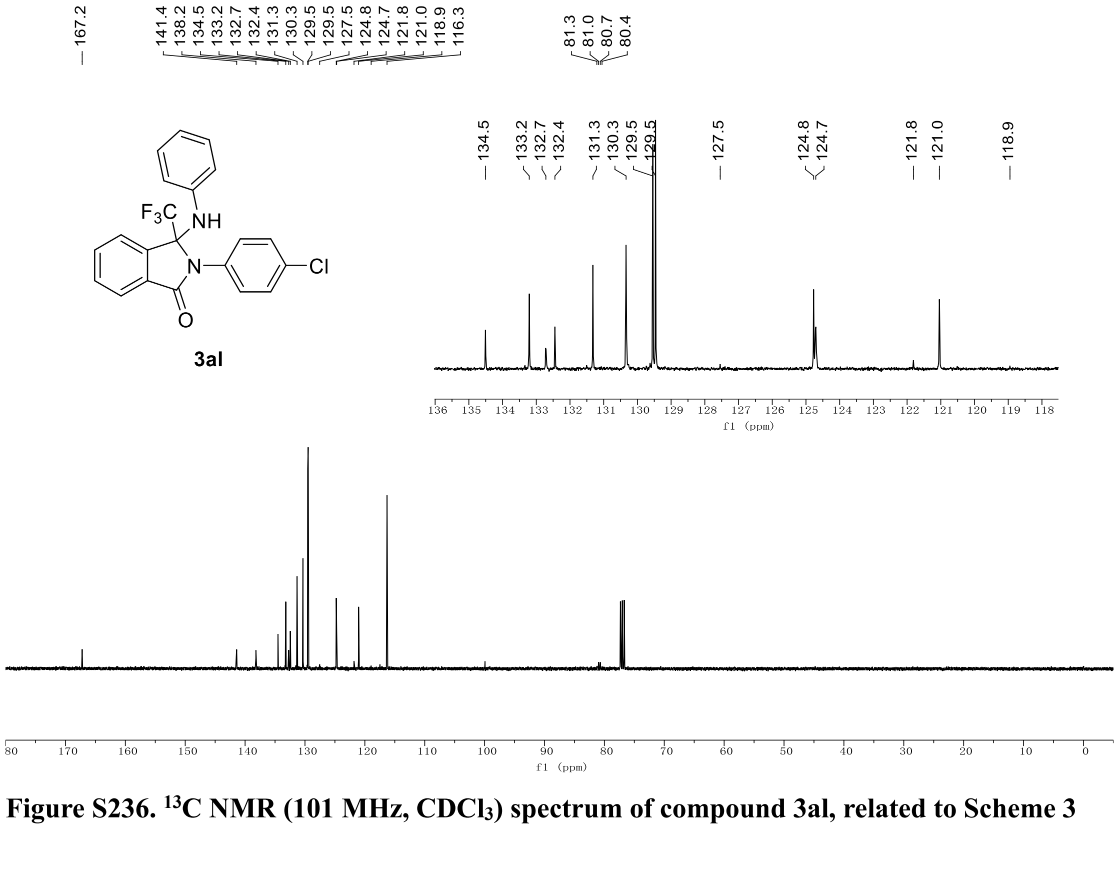

Figure S236. ^{13}C NMR (101 MHz, $CDCl_3$) spectrum of compound 3al, related to Scheme 3

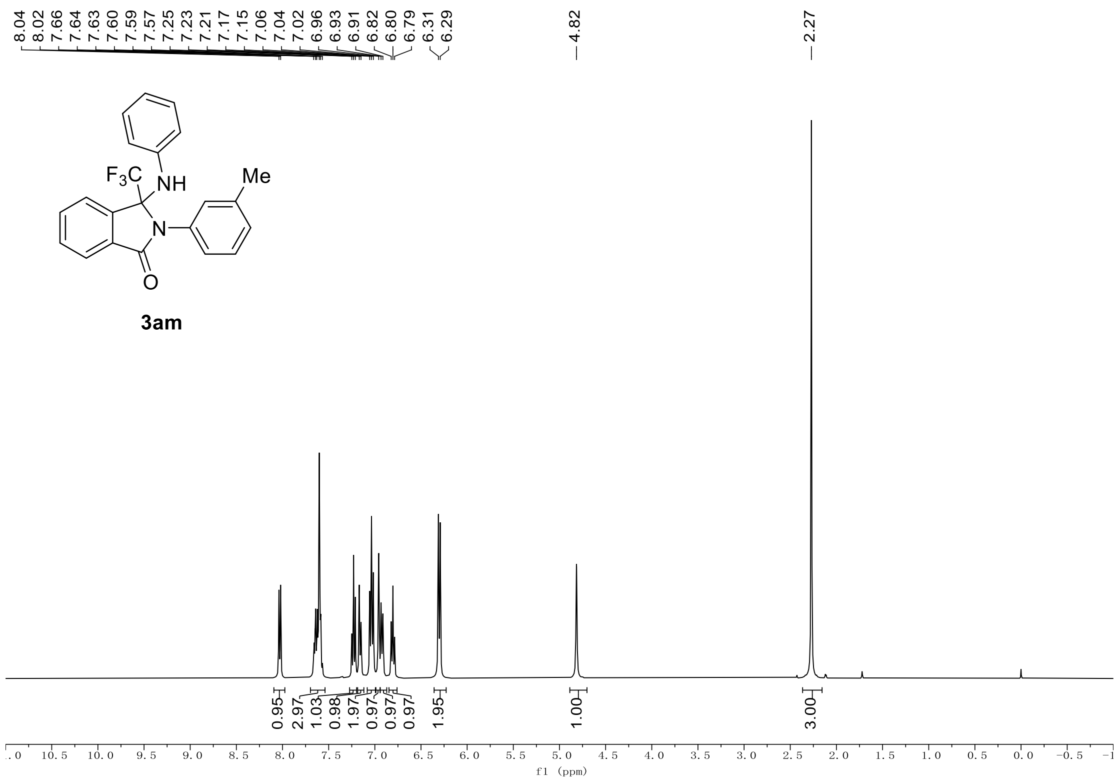

Figure S237. ^{1}**H NMR (400 MHz, CDCl$_3$) spectrum of compound 3am, related to Scheme 3**

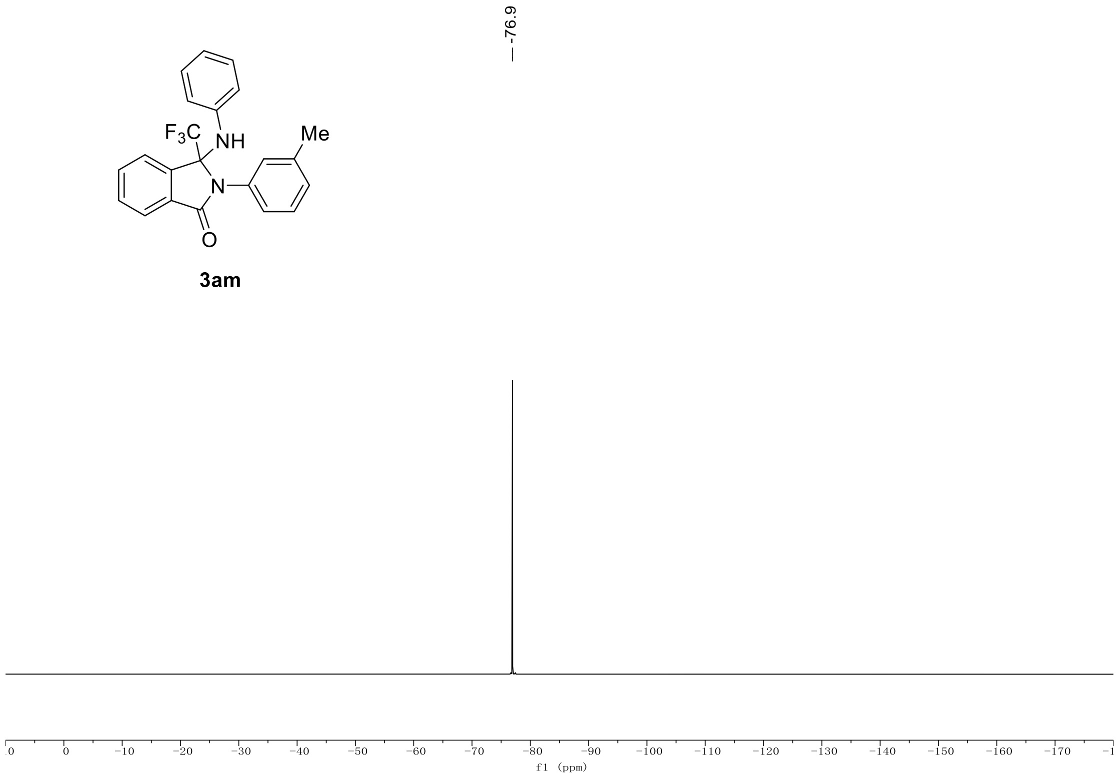

Figure S238. ^{19}**F NMR (376 MHz, CDCl$_3$) spectrum of compound 3am, related to Scheme 3**

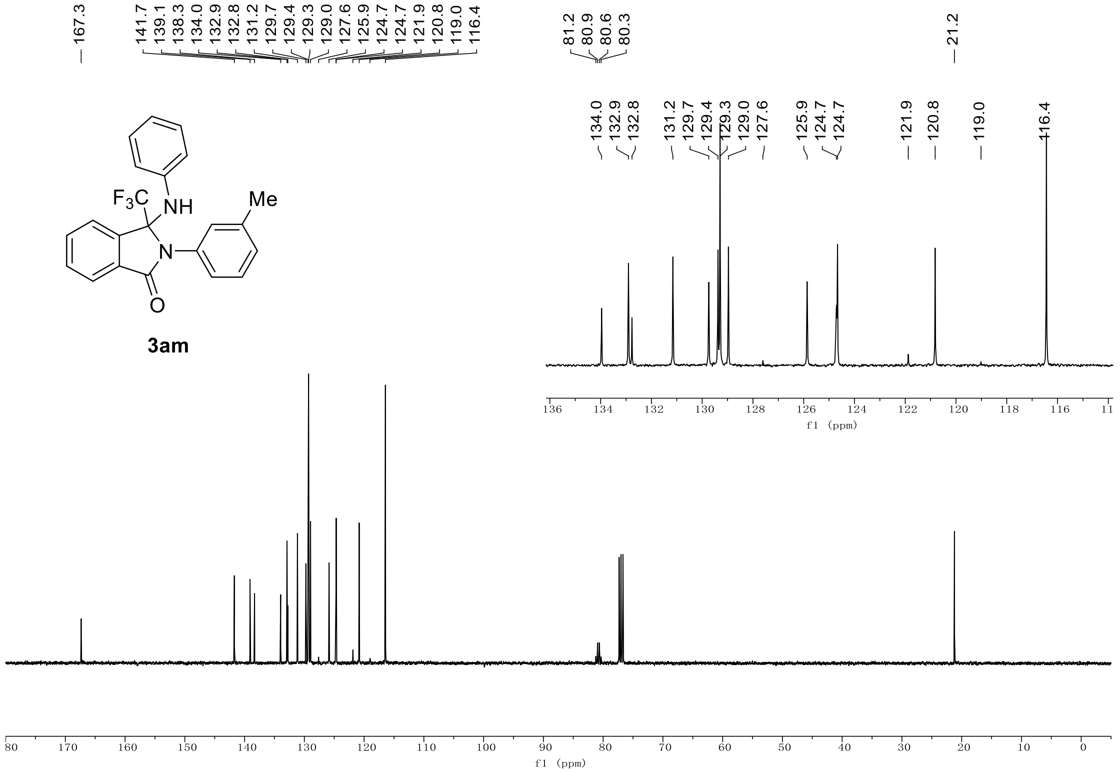

Figure S239. ^{13}C NMR (101 MHz, $CDCl_3$) spectrum of compound 3am, related to Scheme 3

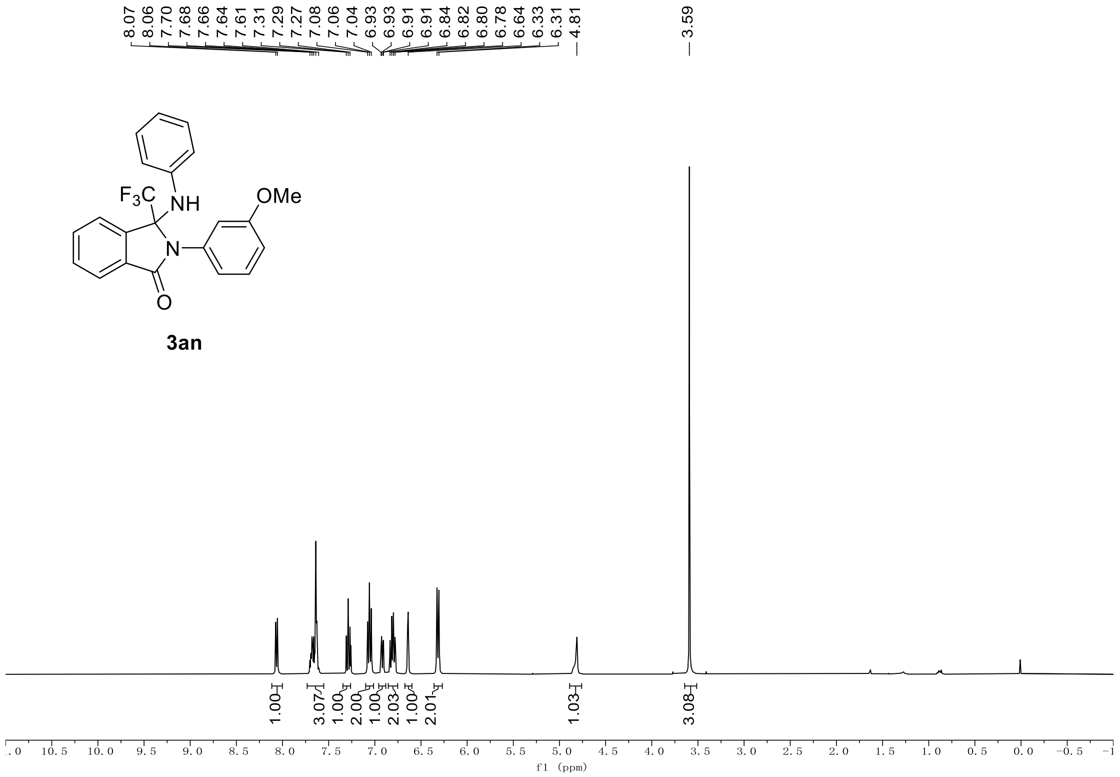

Figure S240. ^{1}H NMR (400 MHz, $CDCl_3$) spectrum of compound 3an, related to Scheme 3

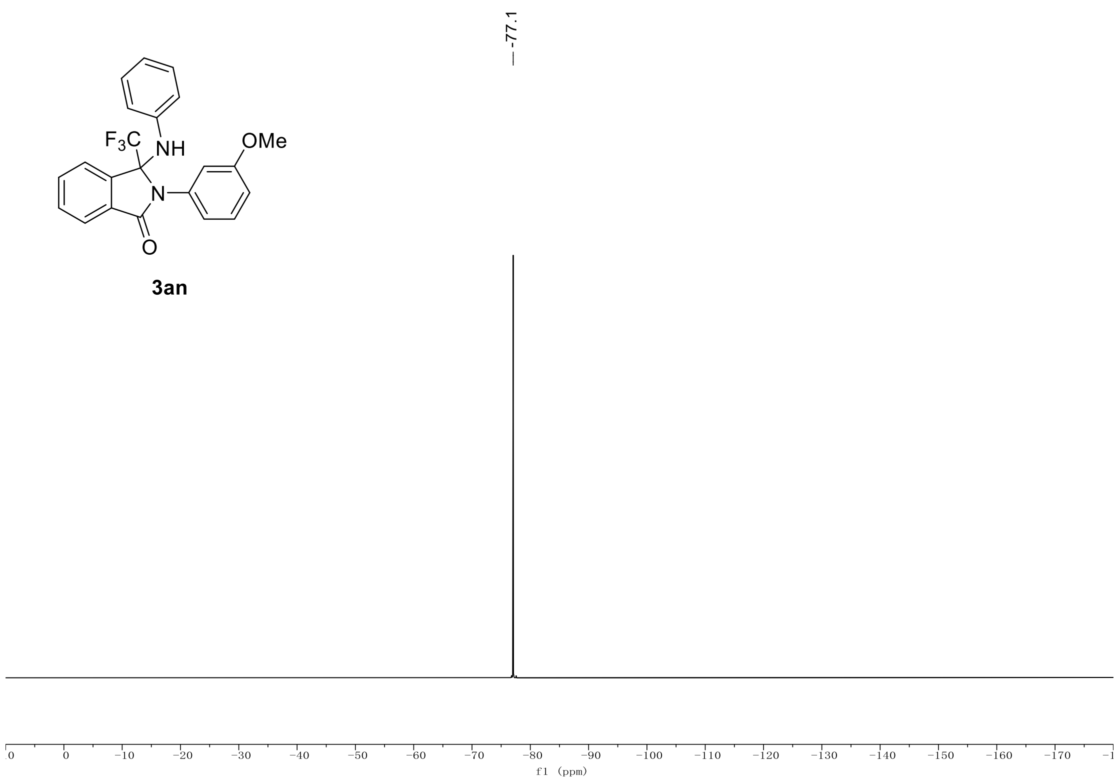

Figure S241. ^{19}F NMR (376 MHz, CDCl$_3$) spectrum of compound 3an, related to Scheme 3

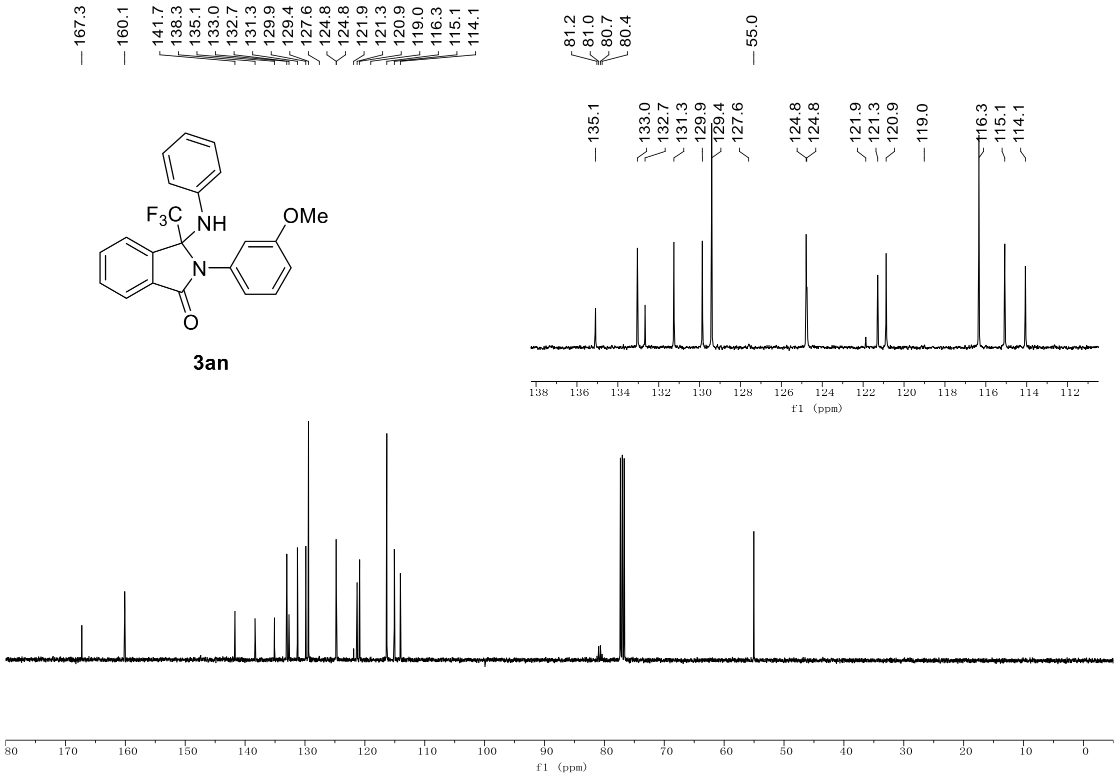

Figure S242. ^{13}C NMR (101 MHz, CDCl$_3$) spectrum of compound 3an, related to Scheme 3

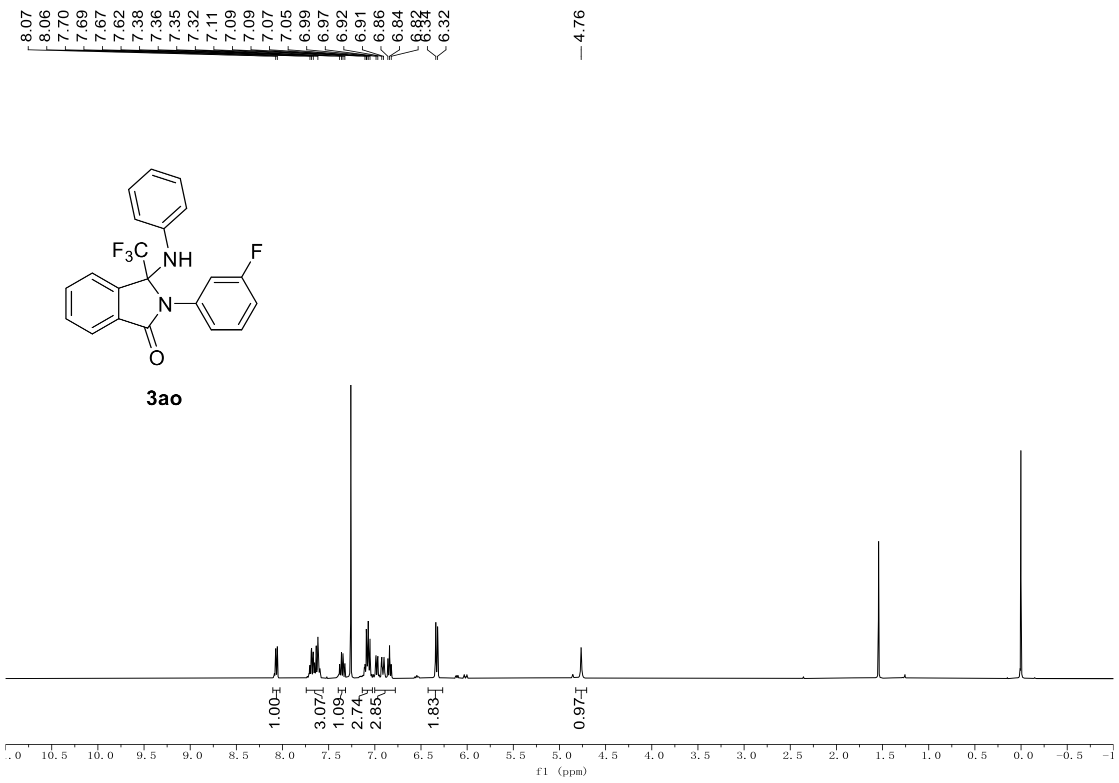

Figure S243. ^{1}H NMR (400 MHz, CDCl$_3$) spectrum of compound 3ao, related to Scheme 3

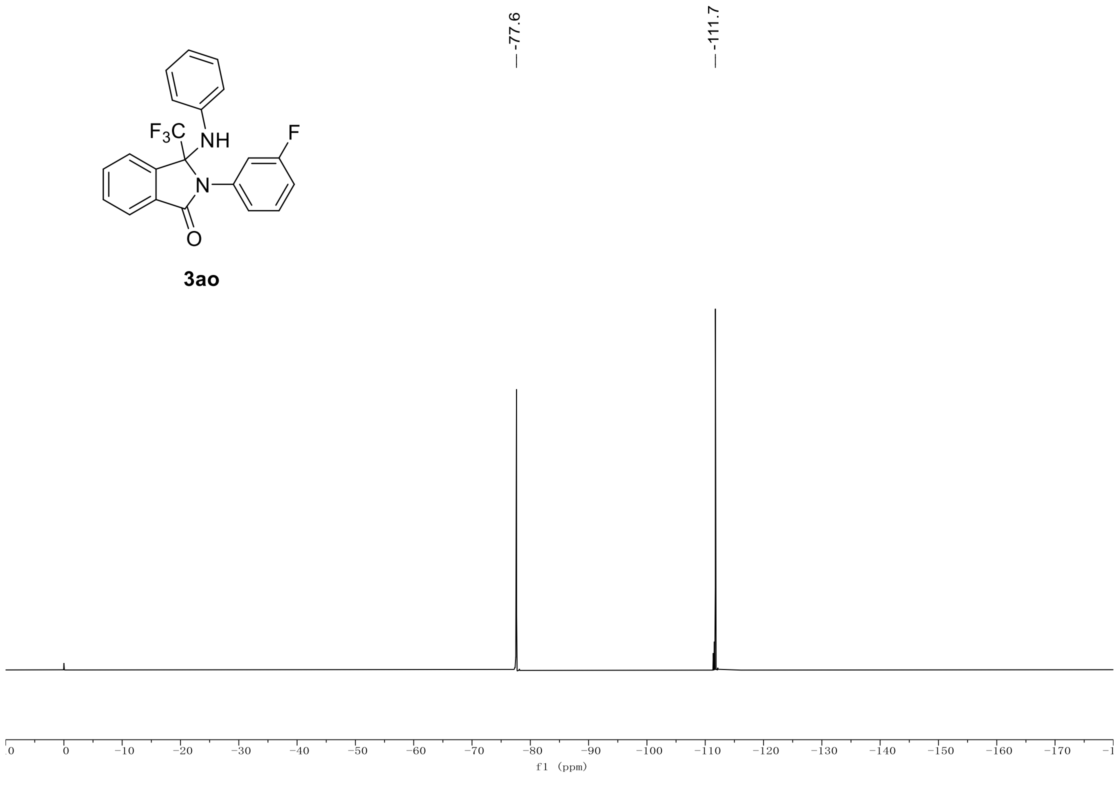

Figure S244. ^{19}F NMR (376 MHz, CDCl$_3$) spectrum of compound 3ao, related to Scheme 3

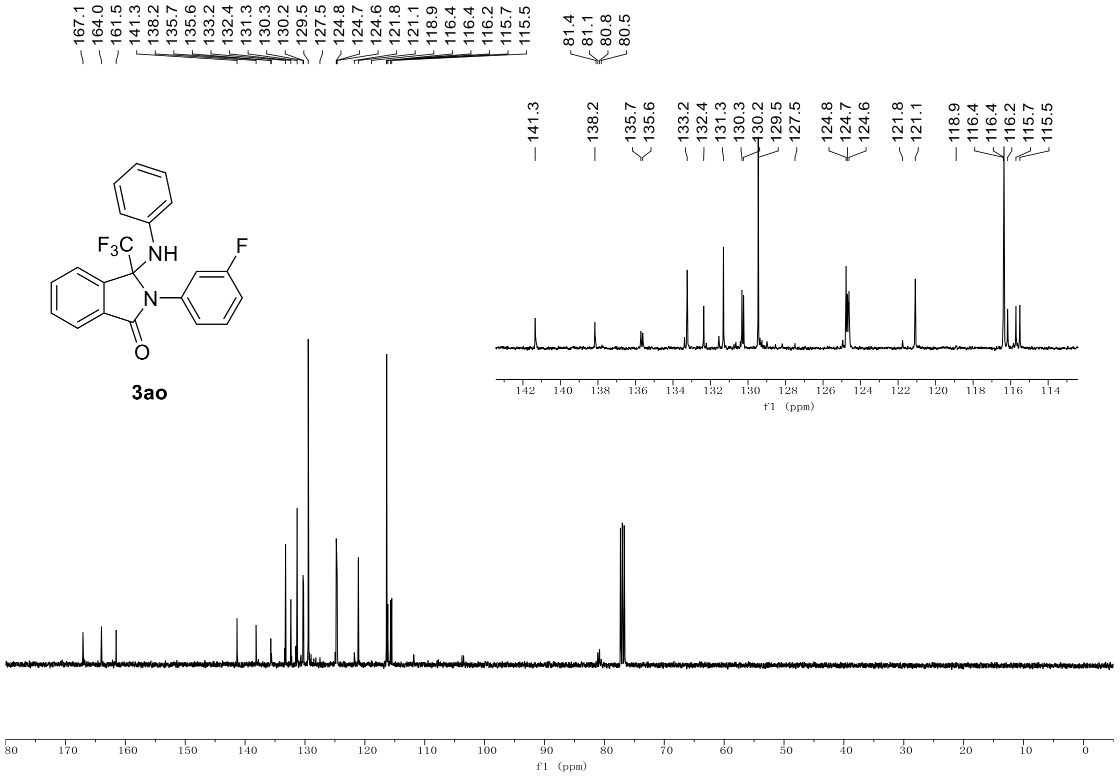

Figure S245. ^{13}C NMR (101 MHz, $CDCl_3$) spectrum of compound 3ao, related to Scheme 3

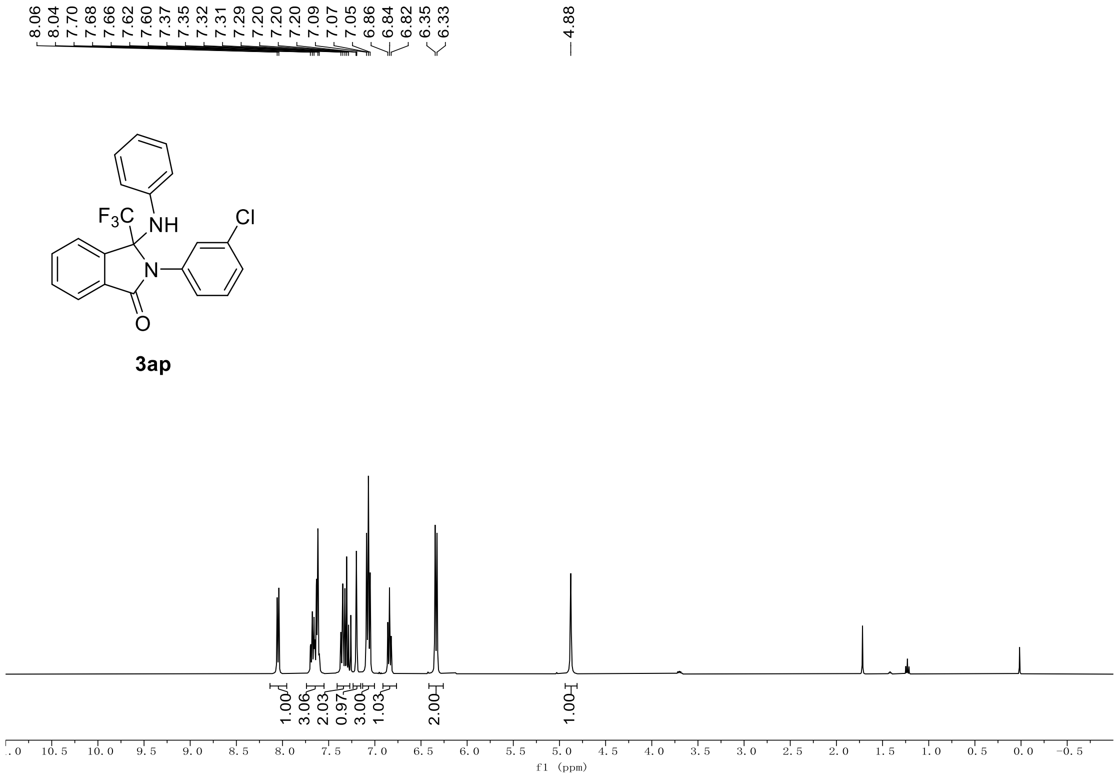

Figure S246. ^{1}H NMR (400 MHz, $CDCl_3$) spectrum of compound 3ap, related to Scheme 3

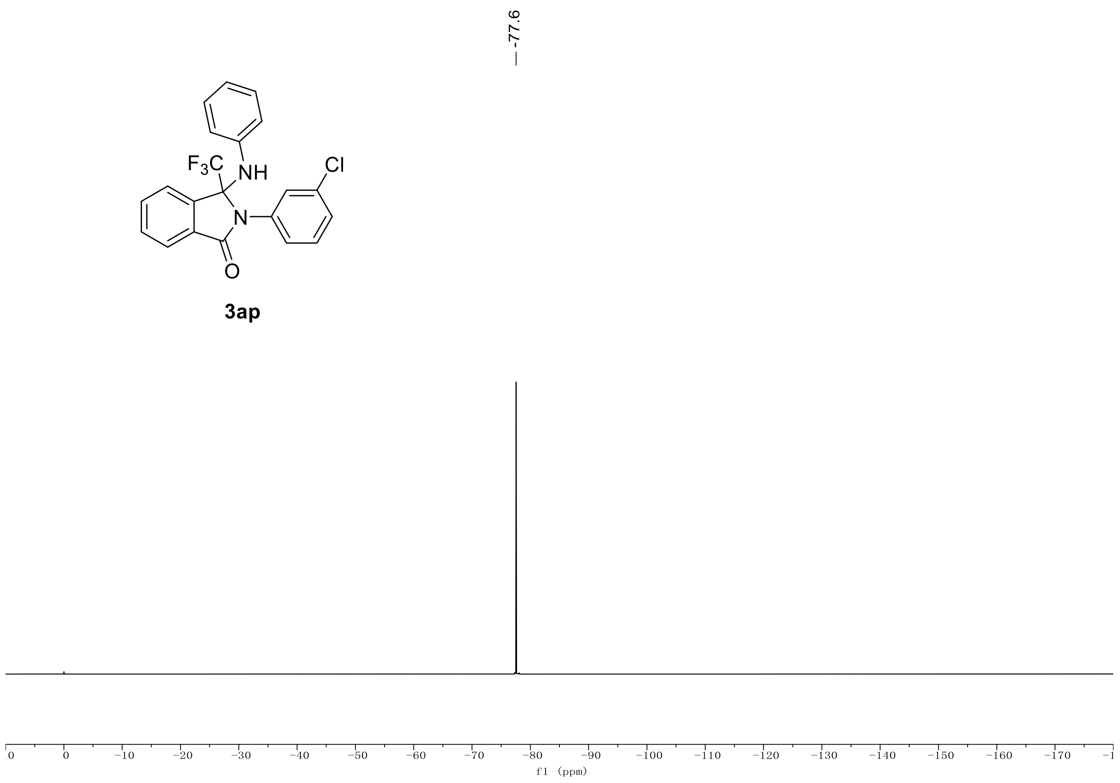

Figure S247. ^{19}F NMR (376 MHz, CDCl$_3$) spectrum of compound 3ap, related to Scheme 3

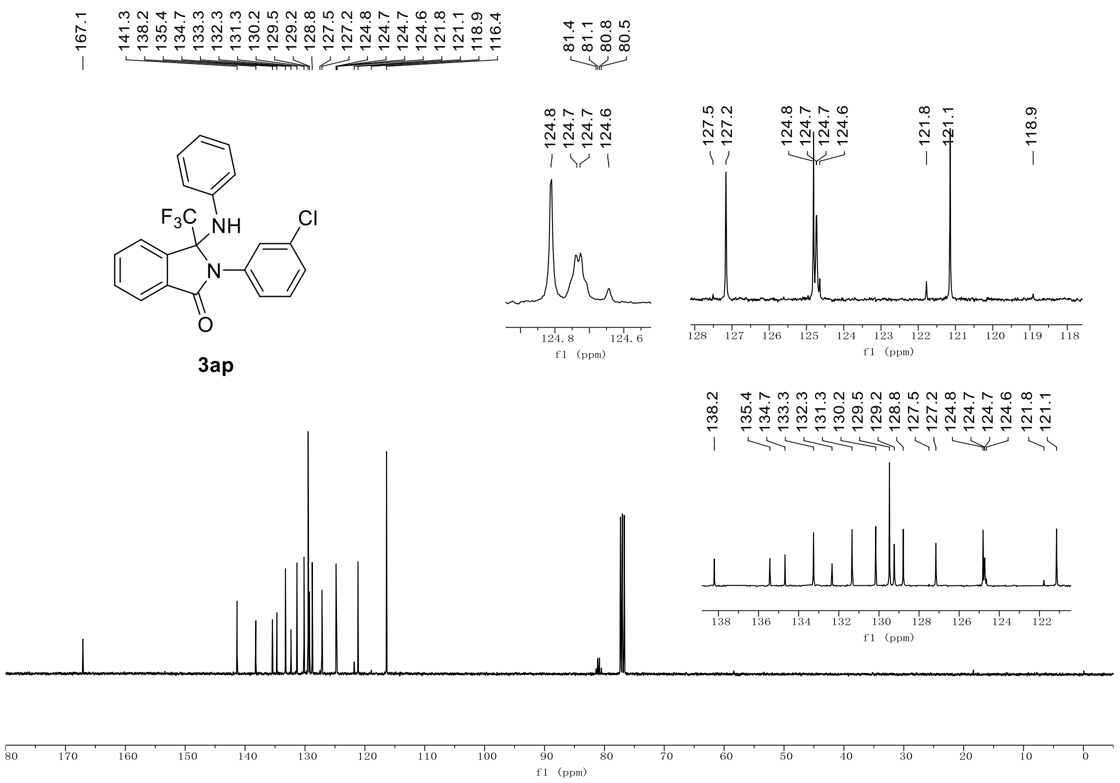

Figure S248. ^{13}C NMR (101 MHz, CDCl$_3$) spectrum of compound 3ap, related to Scheme 3

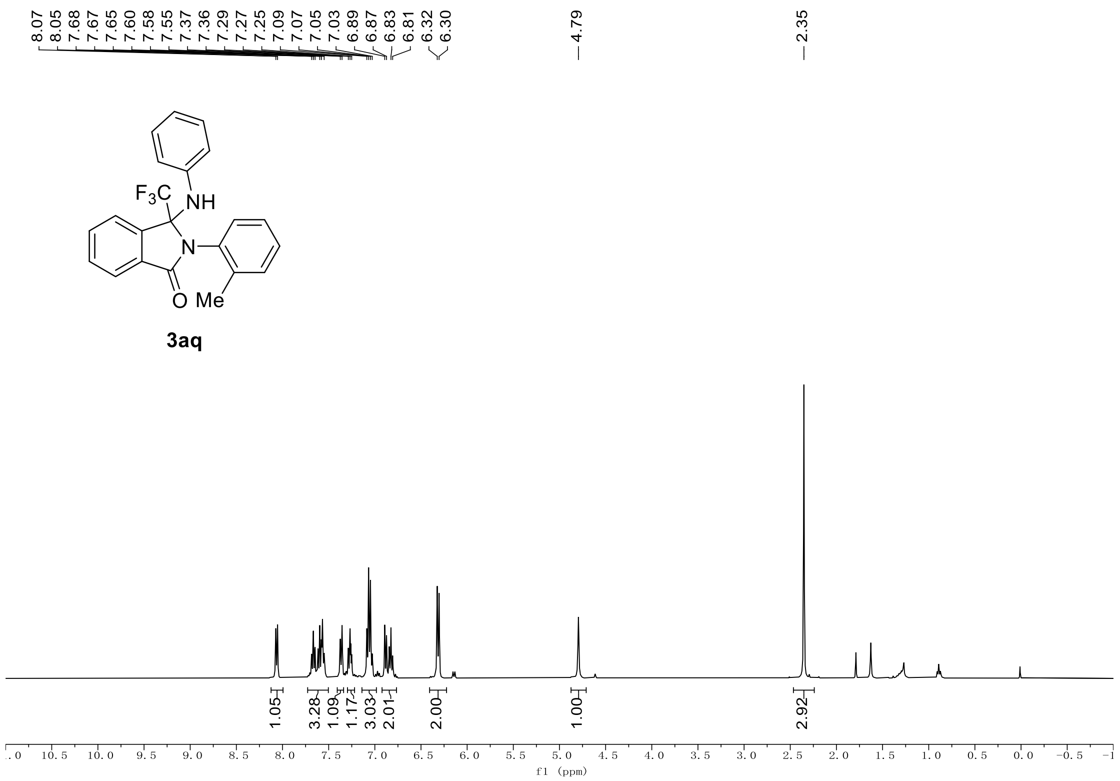

Figure S249. ^{1}H NMR (400 MHz, $CDCl_3$) spectrum of compound 3aq, related to Scheme 3

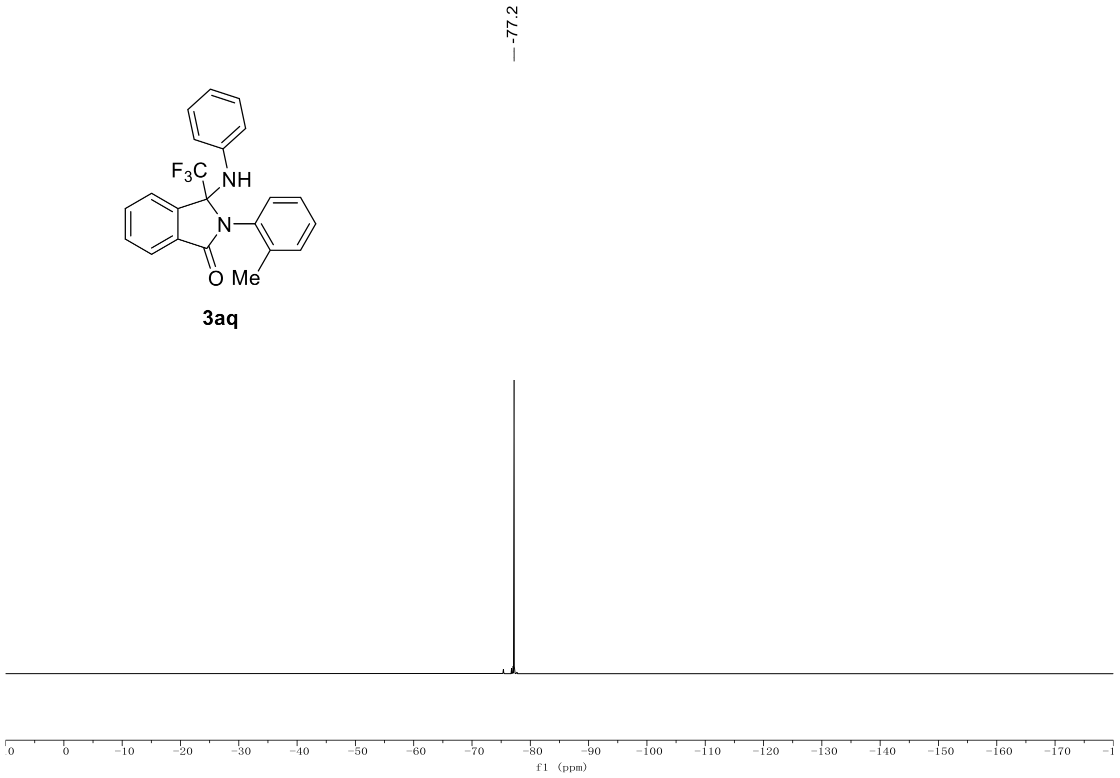

Figure S250. ^{19}F NMR (376 MHz, $CDCl_3$) spectrum of compound 3aq, related to Scheme 3

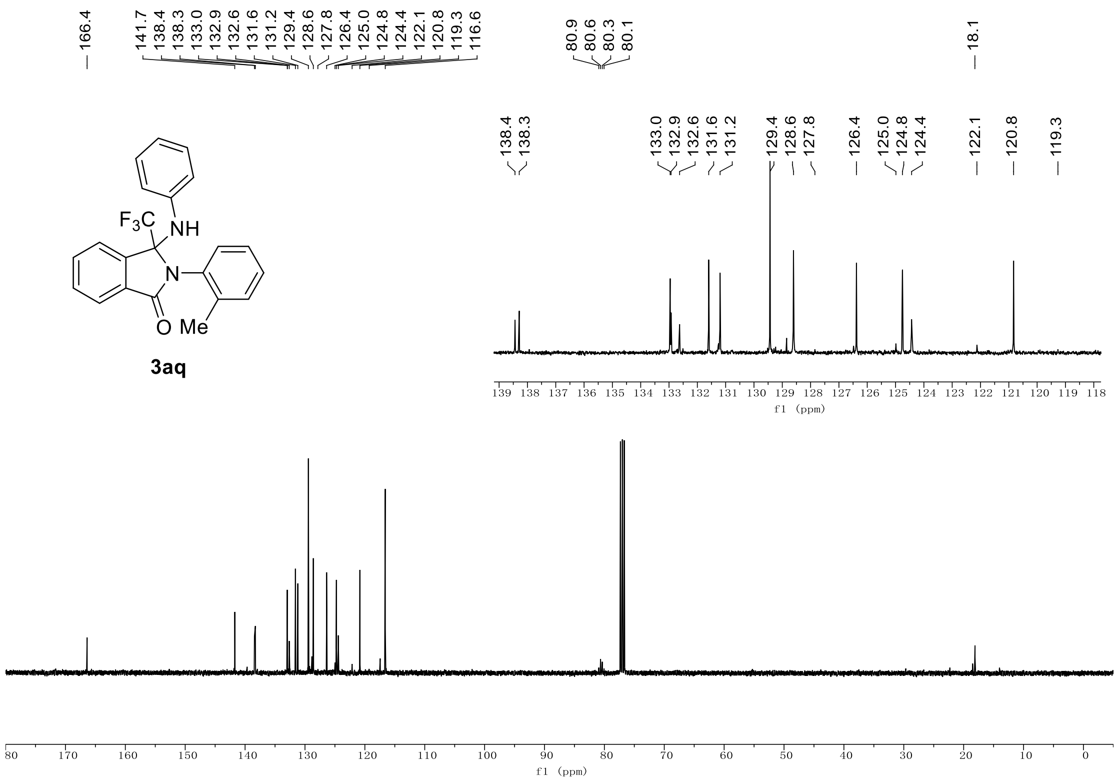

Figure S251. ^{13}C **NMR (101 MHz,** $CDCl_3$**) spectrum of compound 3aq, related to Scheme 3**

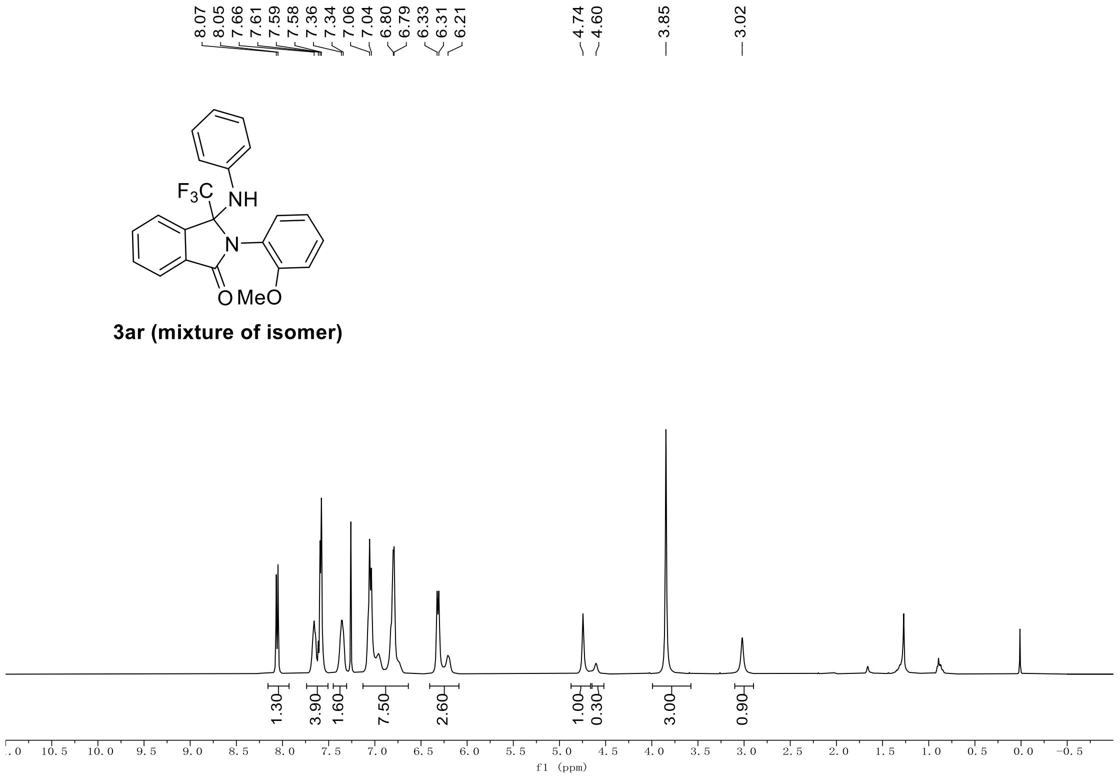

Figure S252. ^{1}H **NMR (400 MHz,** $CDCl_3$**) spectrum of compound 3ar, related to Scheme 3**

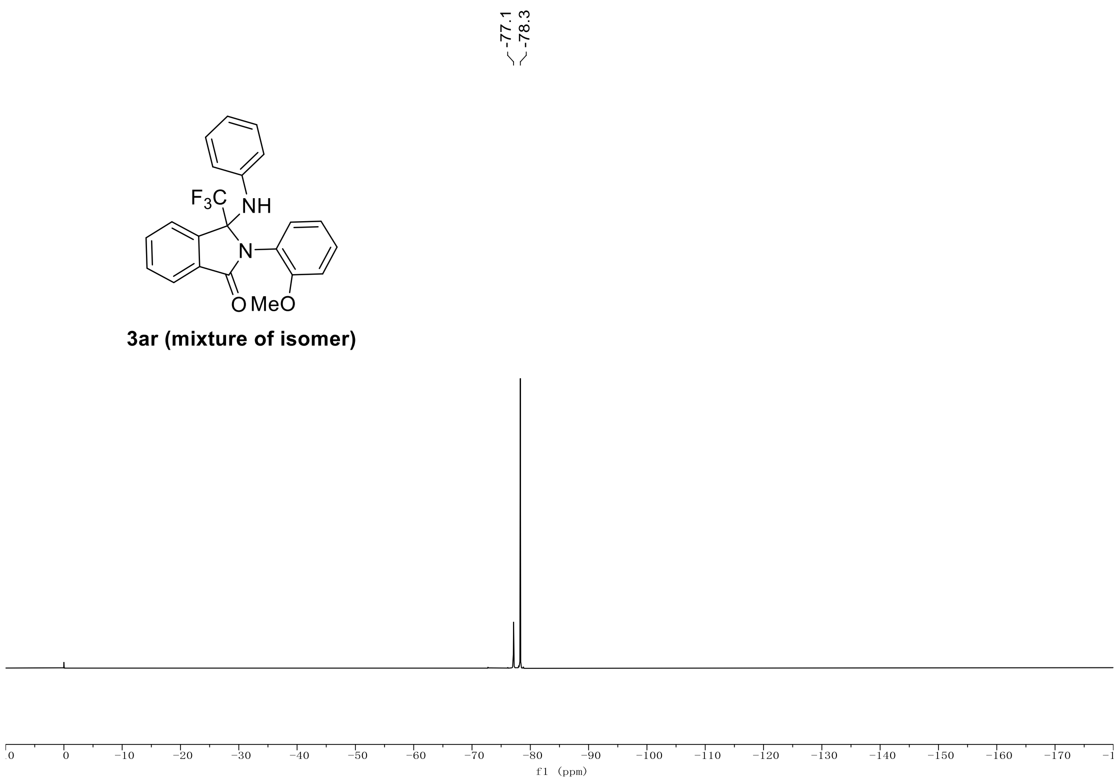

Figure S253. ^{19}F NMR (376 MHz, $CDCl_3$) spectrum of compound 3ar, related to Scheme 3

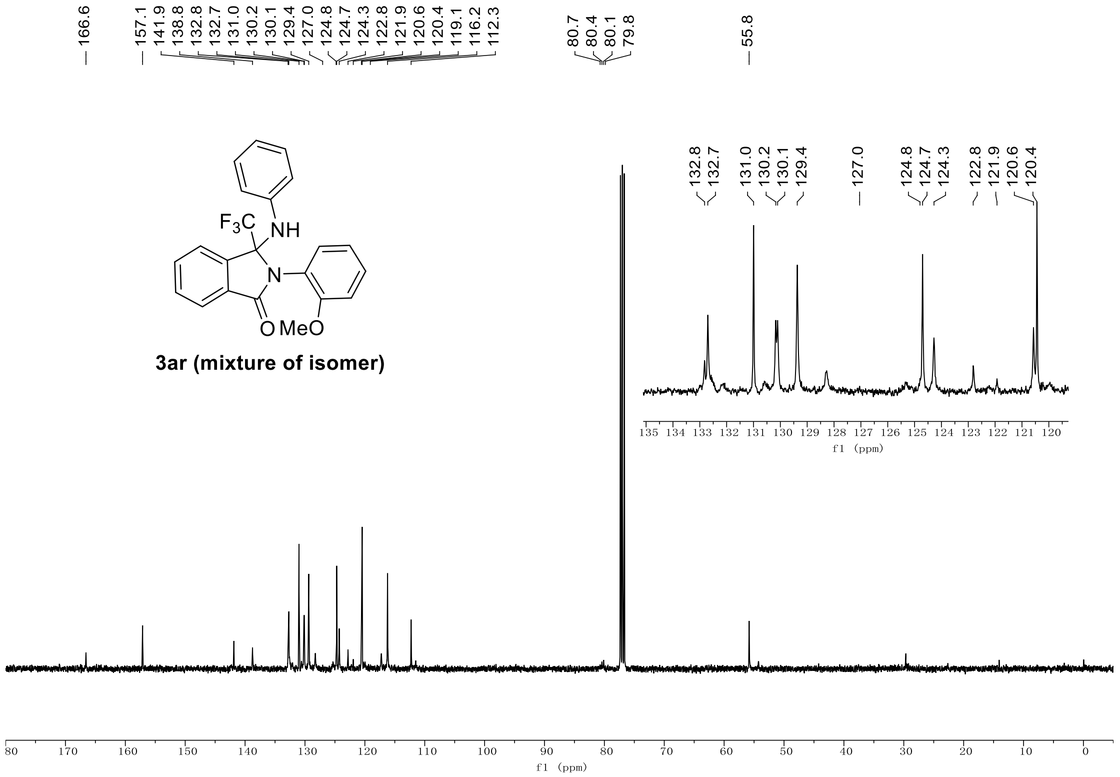

Figure S254. ^{13}C NMR (101 MHz, $CDCl_3$) spectrum of compound 3ar, related to Scheme 3

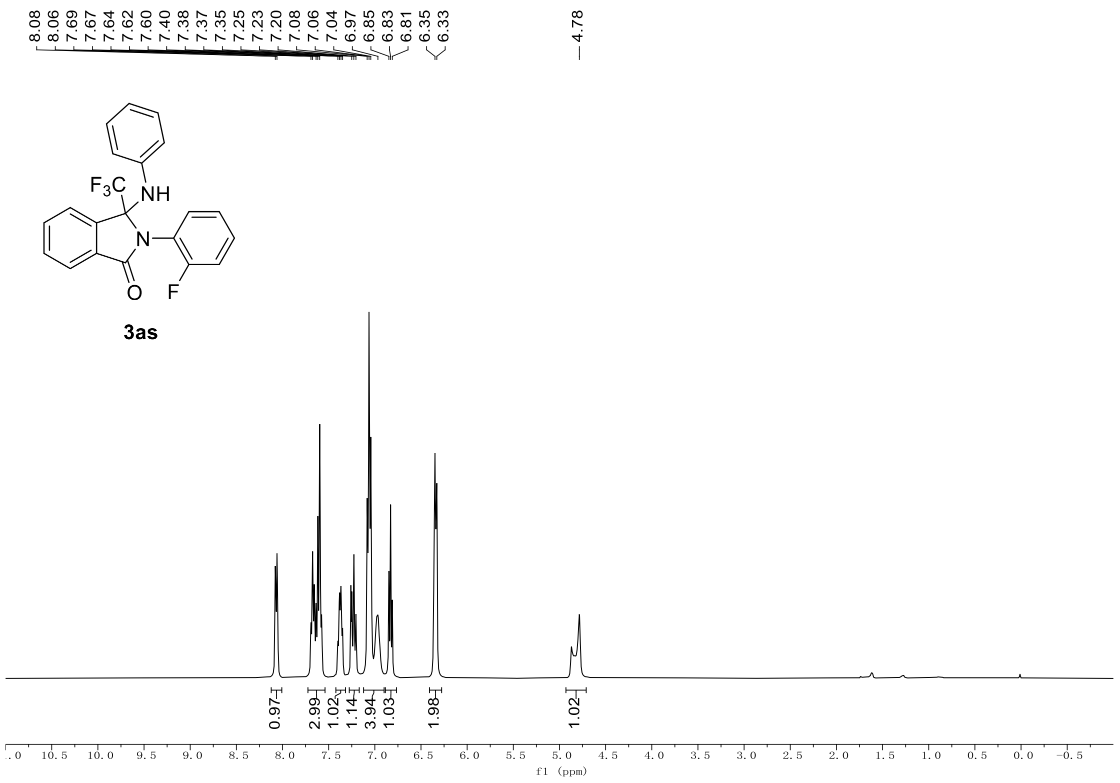

Figure S255. ^{1}H NMR (400 MHz, $CDCl_3$) spectrum of compound 3as, related to Scheme 3

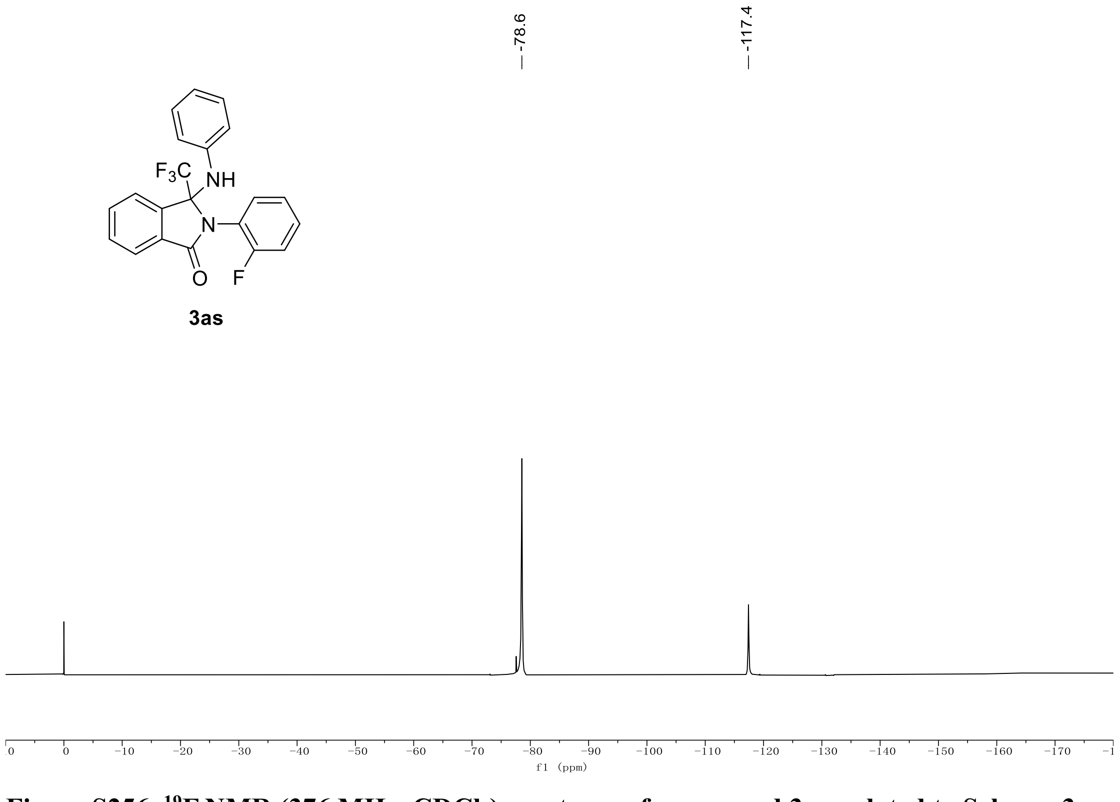

Figure S256. ^{19}F NMR (376 MHz, $CDCl_3$) spectrum of compound 3as, related to Scheme 3

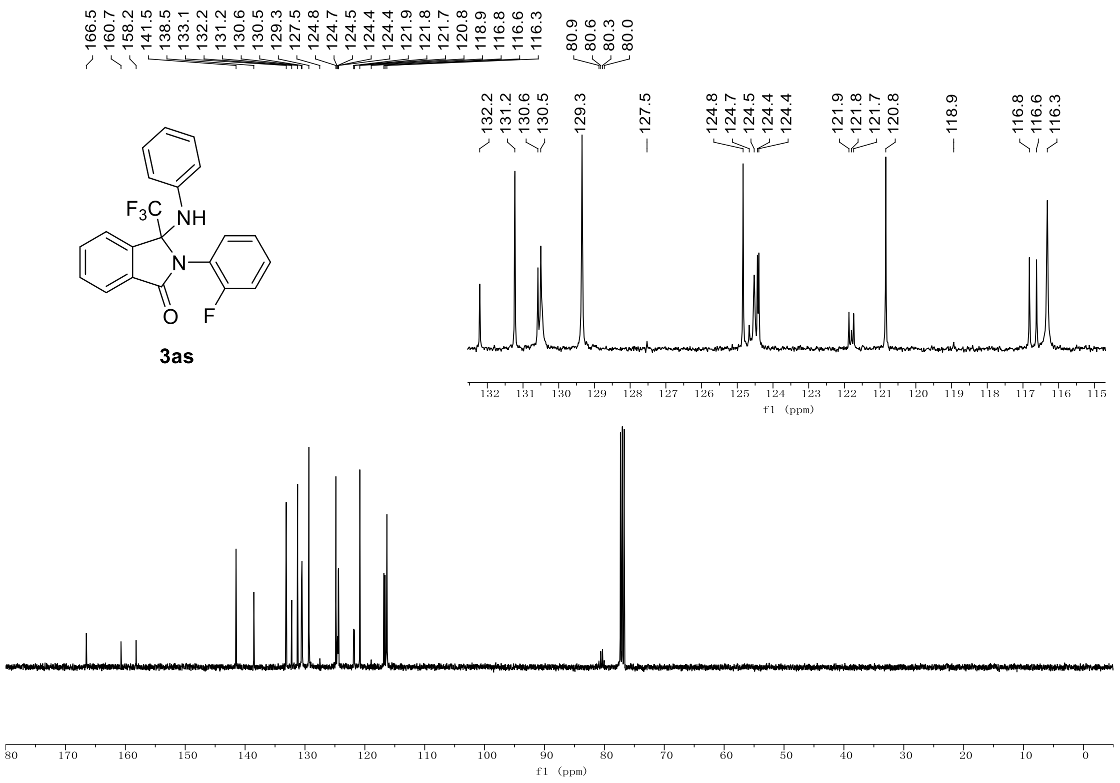

Figure S257. ^{13}**C NMR (101 MHz, CDCl$_3$) spectrum of compound 3as, related to Scheme 3**

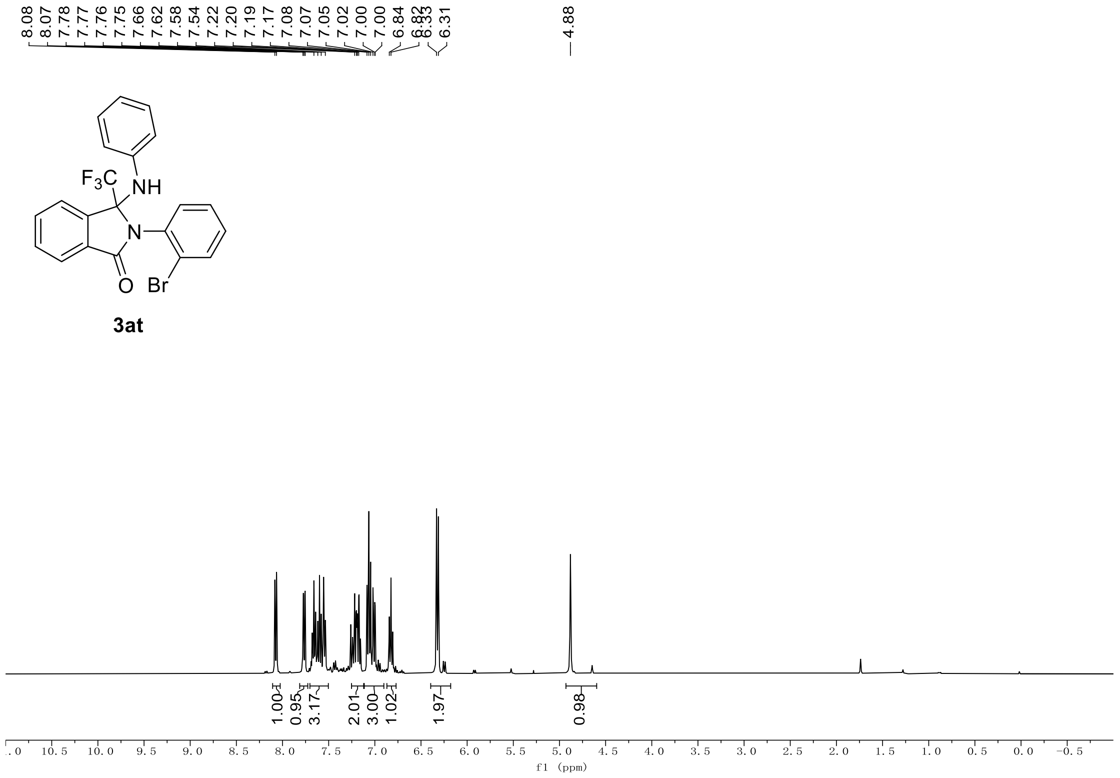

Figure S258. ^{1}**H NMR (400 MHz, CDCl$_3$) spectrum of compound 3at, related to Scheme 3**

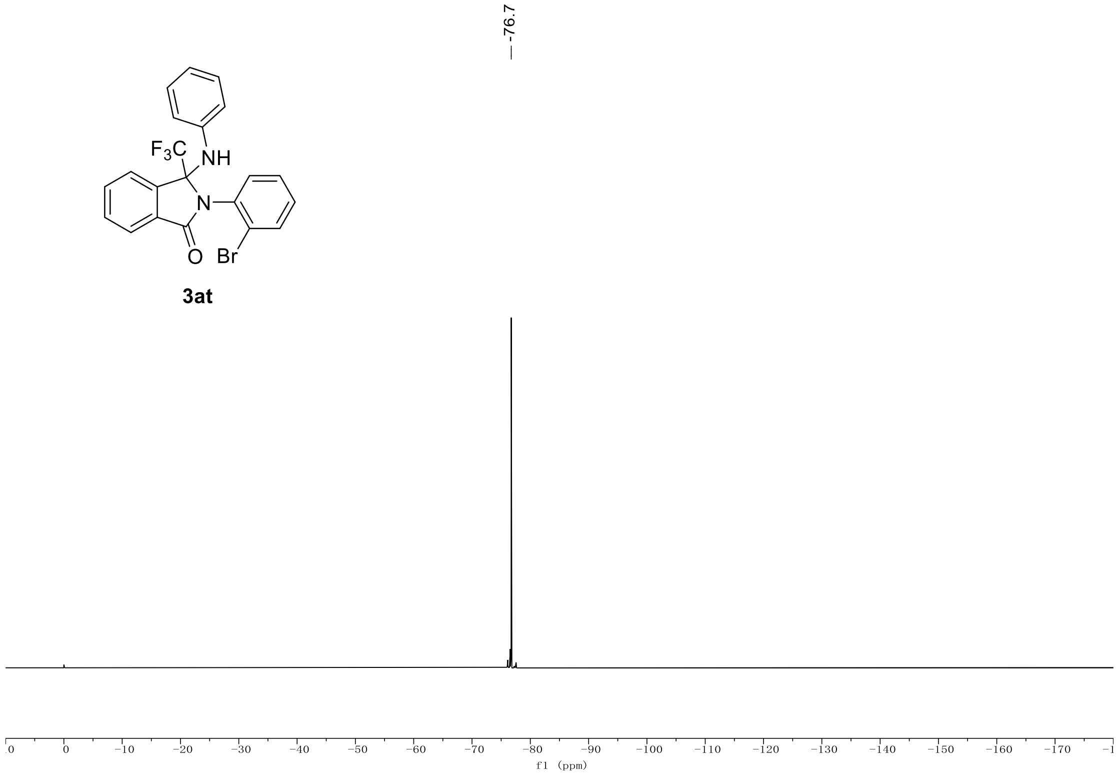

Figure S259. ^{19}F NMR (376 MHz, $CDCl_3$) spectrum of compound 3at, related to Scheme 3

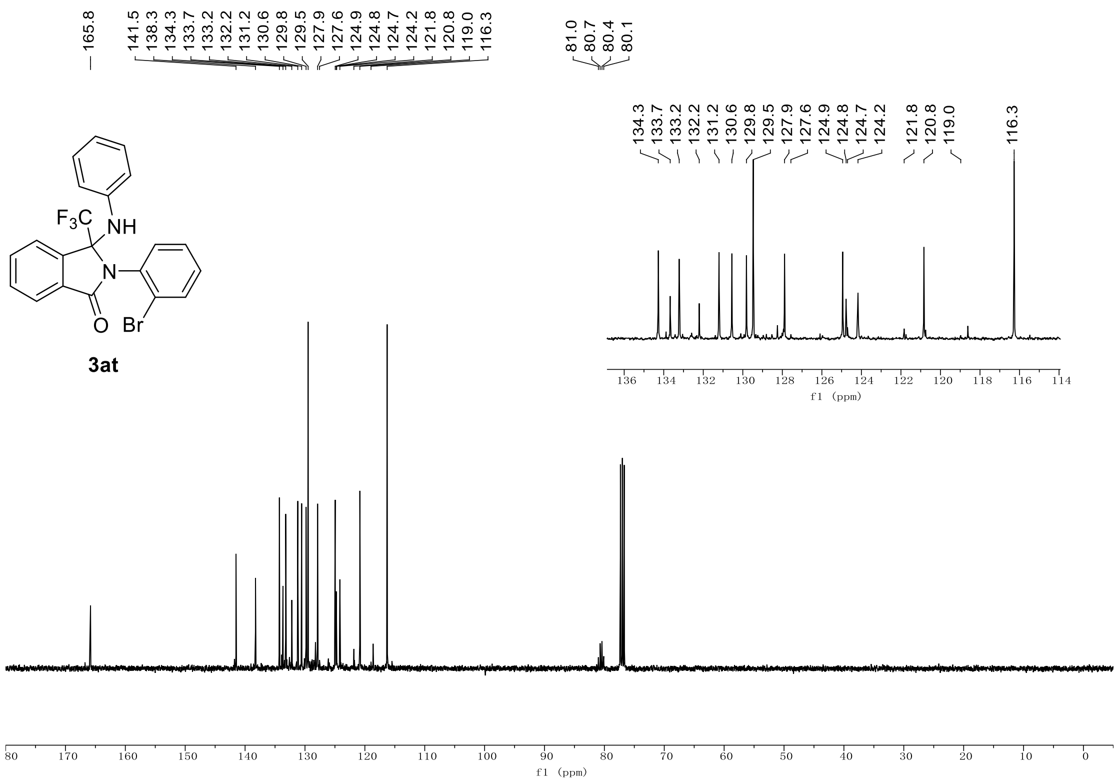

Figure S260. ^{13}C NMR (101 MHz, $CDCl_3$) spectrum of compound 3at, related to Scheme 3

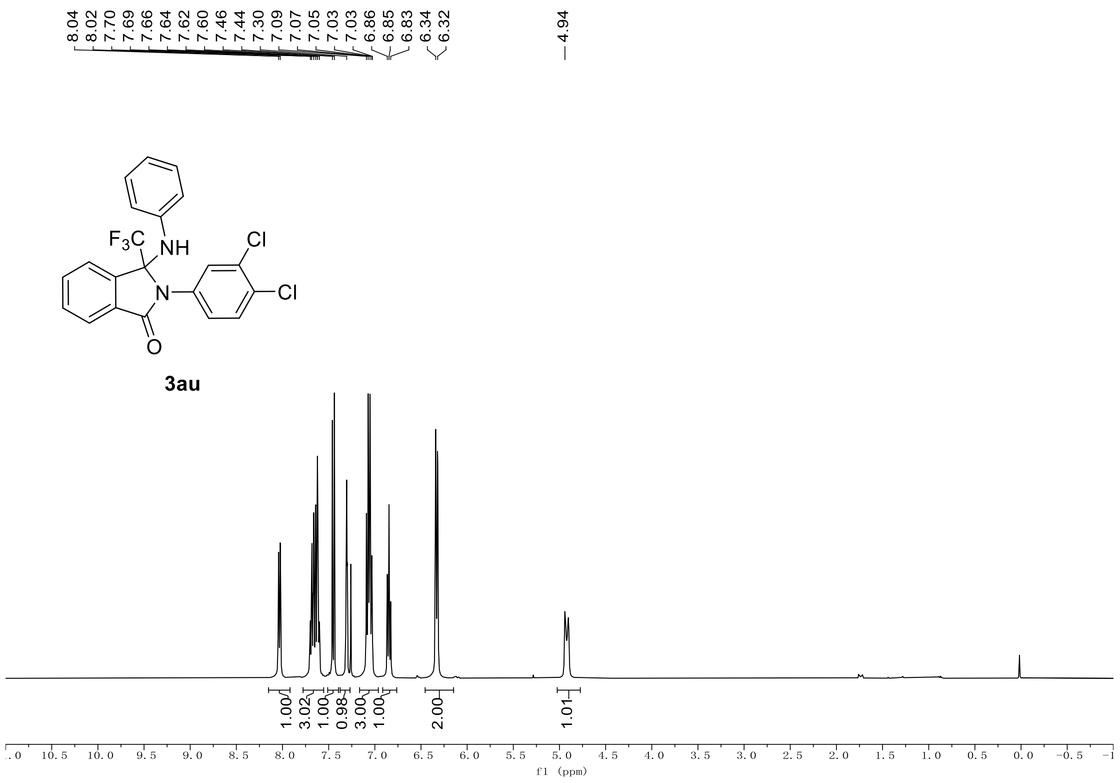

Figure S261. ^{1}H NMR (400 MHz, $CDCl_3$) spectrum of compound 3au, related to Scheme 3

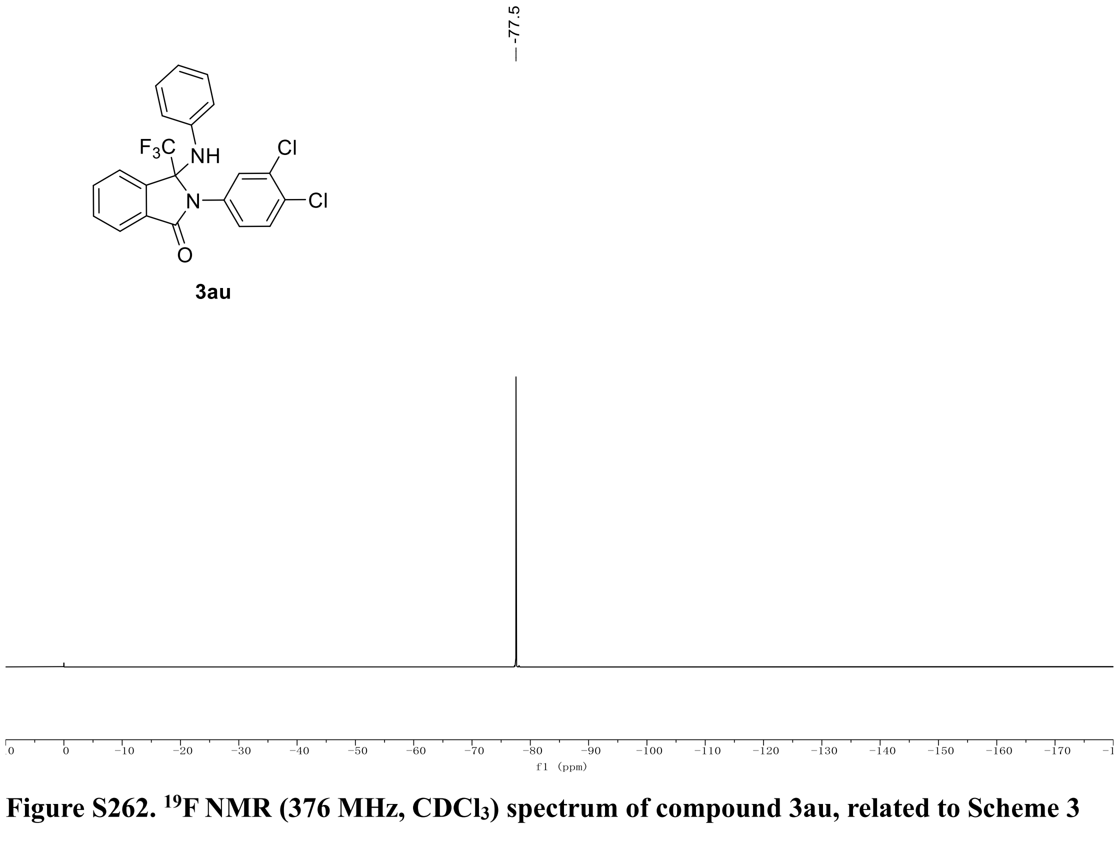

Figure S262. ^{19}F NMR (376 MHz, $CDCl_3$) spectrum of compound 3au, related to Scheme 3

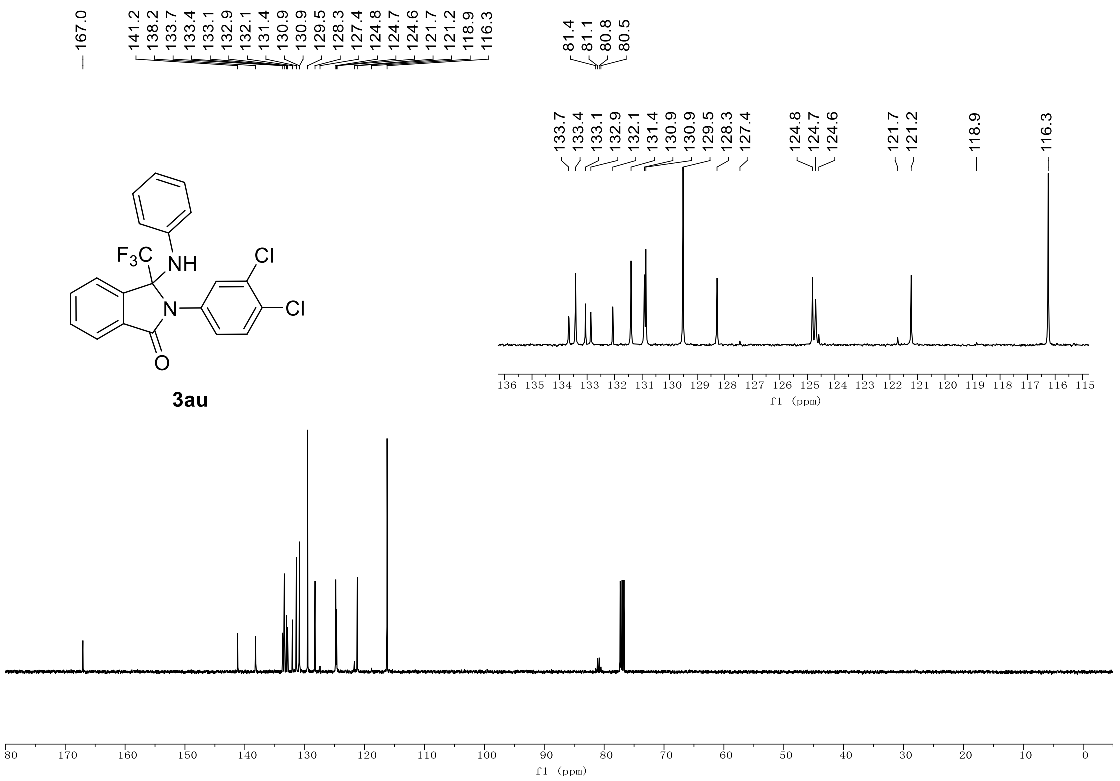

3au

Figure S263. ^{13}C NMR (101 MHz, $CDCl_3$) spectrum of compound 3au, related to Scheme 3

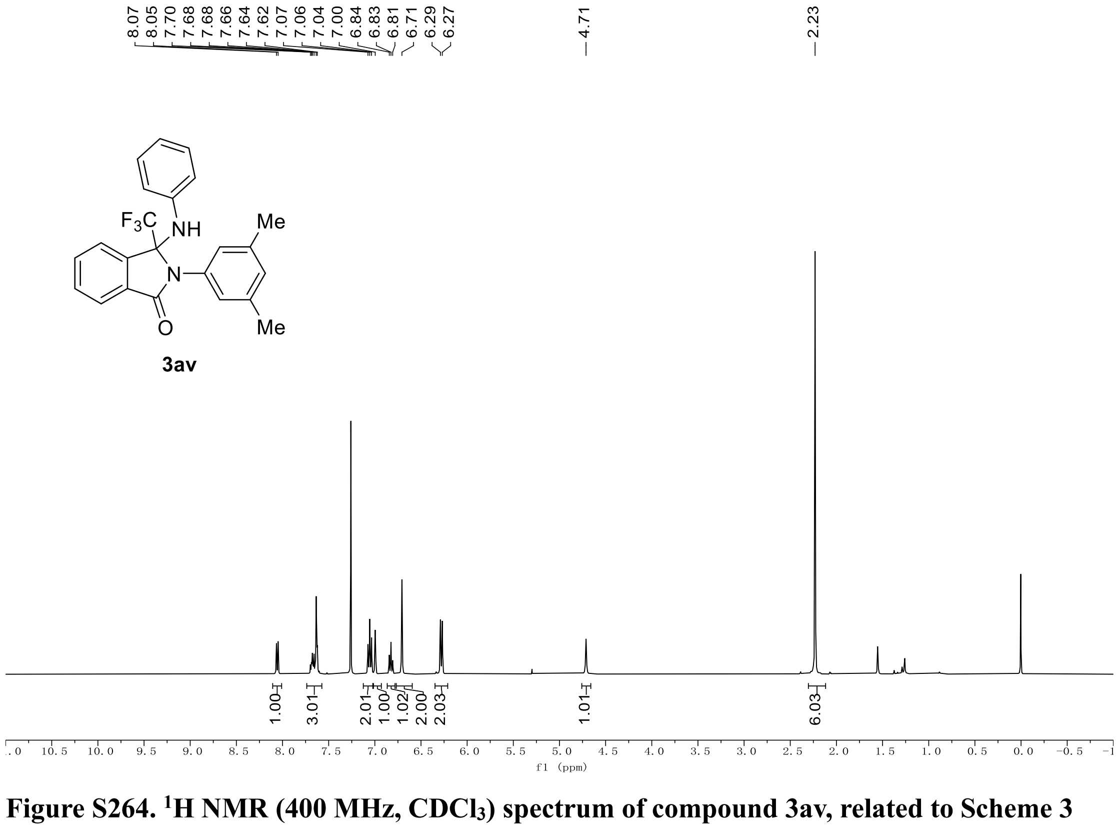

3av

Figure S264. ^{1}H NMR (400 MHz, $CDCl_3$) spectrum of compound 3av, related to Scheme 3

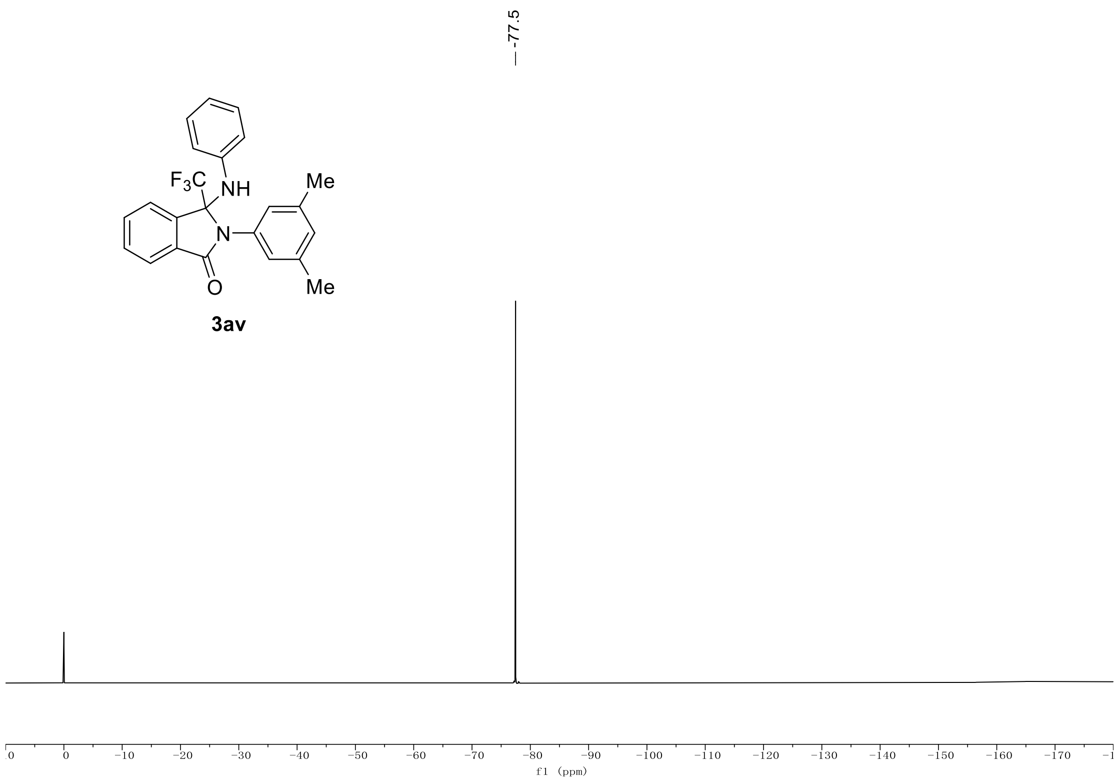

Figure S265. ^{19}F NMR (376 MHz, $CDCl_3$) spectrum of compound 3av, related to Scheme 3

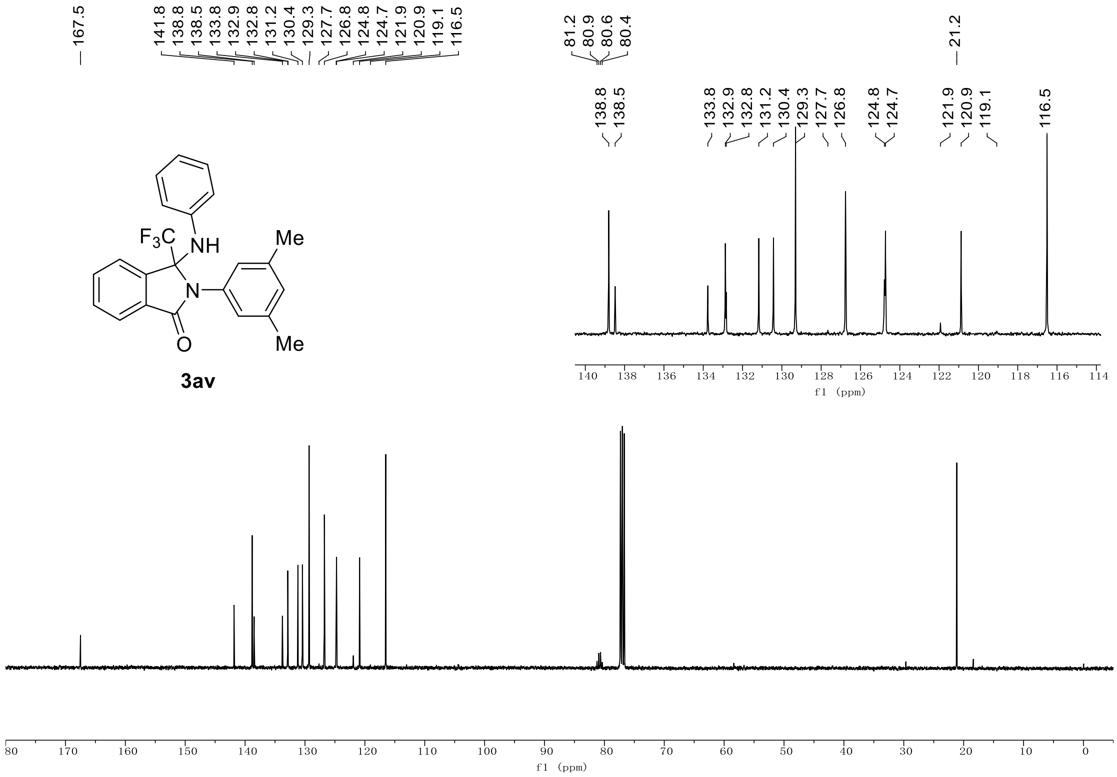

Figure S266. ^{13}C NMR (101 MHz, $CDCl_3$) spectrum of compound 3av, related to Scheme 3

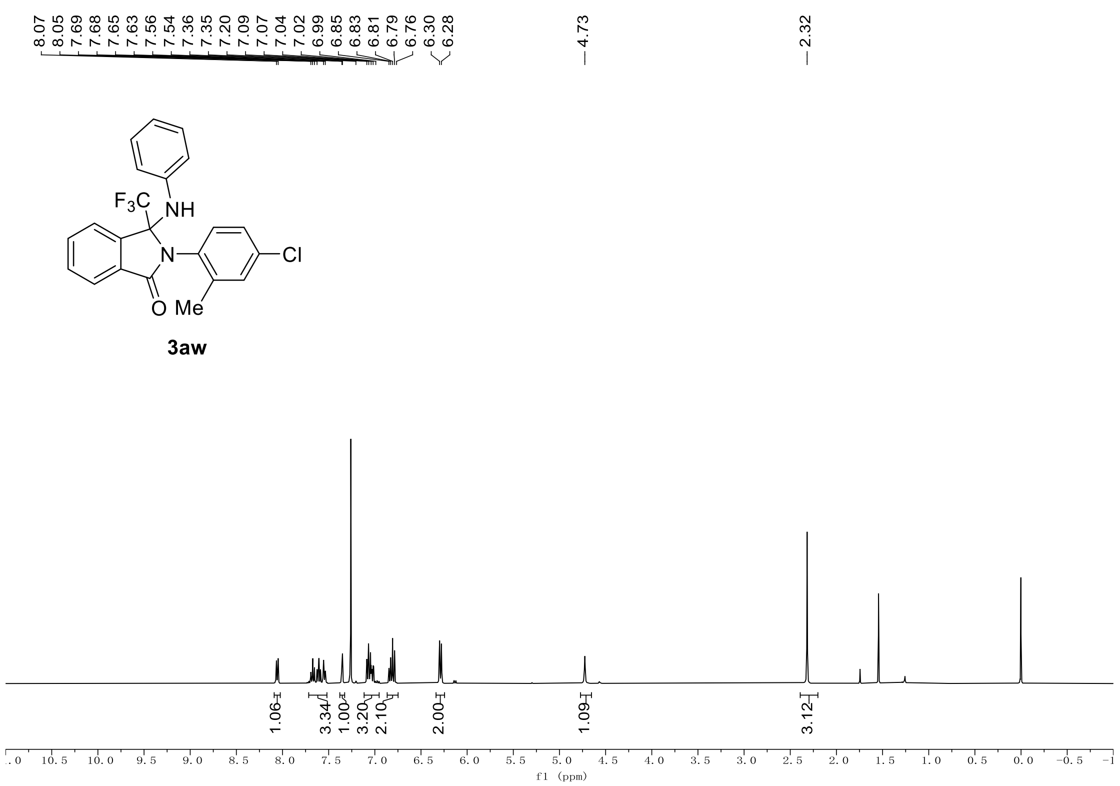

Figure S267. ^{1}H NMR (400 MHz, $CDCl_3$) spectrum of compound 3aw, related to Scheme 3

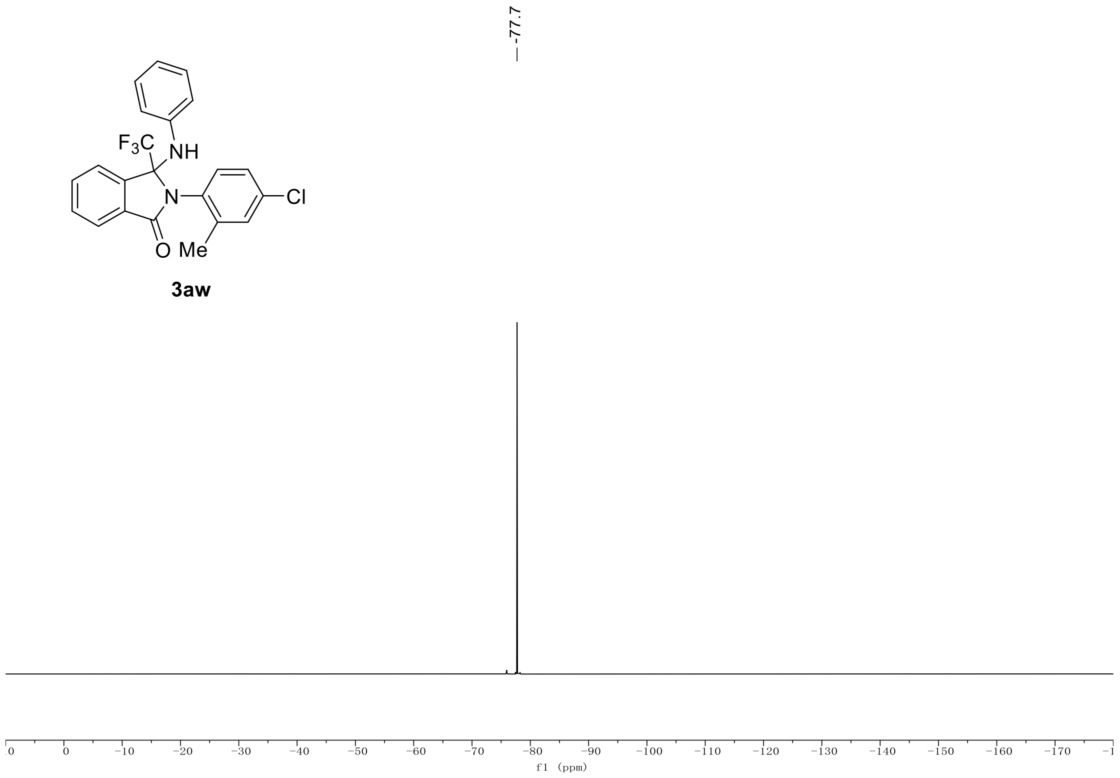

Figure S268. ^{19}F NMR (376 MHz, $CDCl_3$) spectrum of compound 3aw, related to Scheme 3

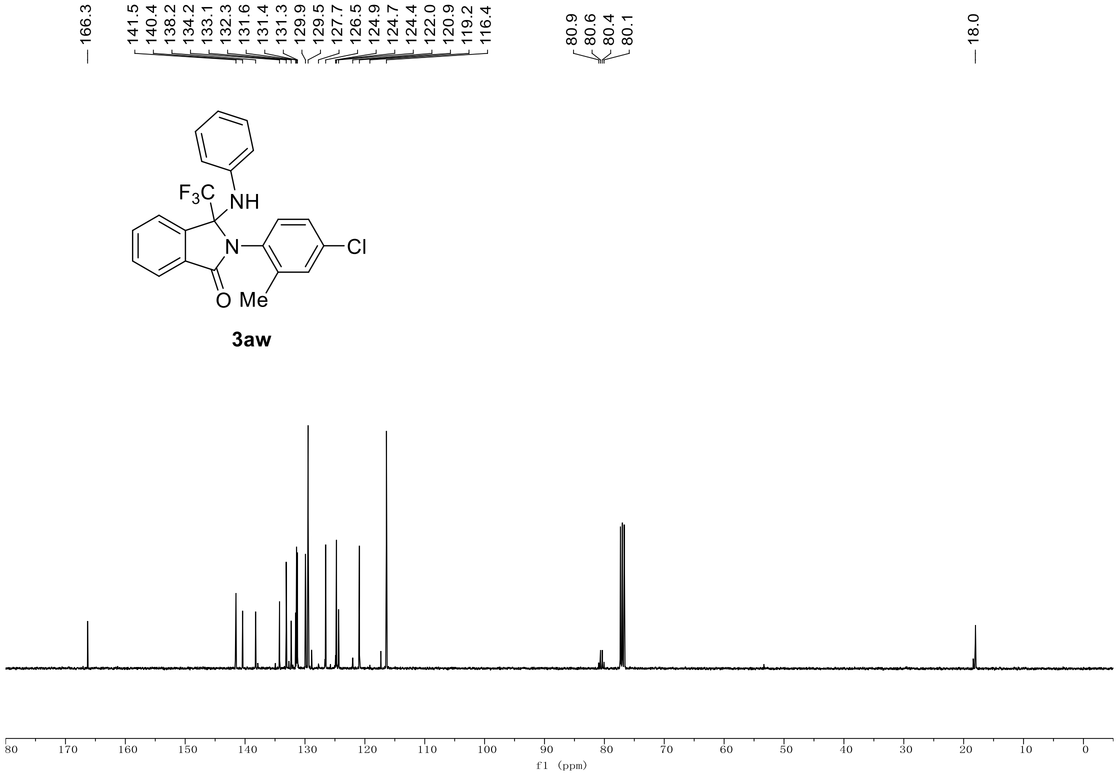

Figure S269. ^{13}C NMR (101 MHz, $CDCl_3$) spectrum of compound 3aw, related to Scheme 3

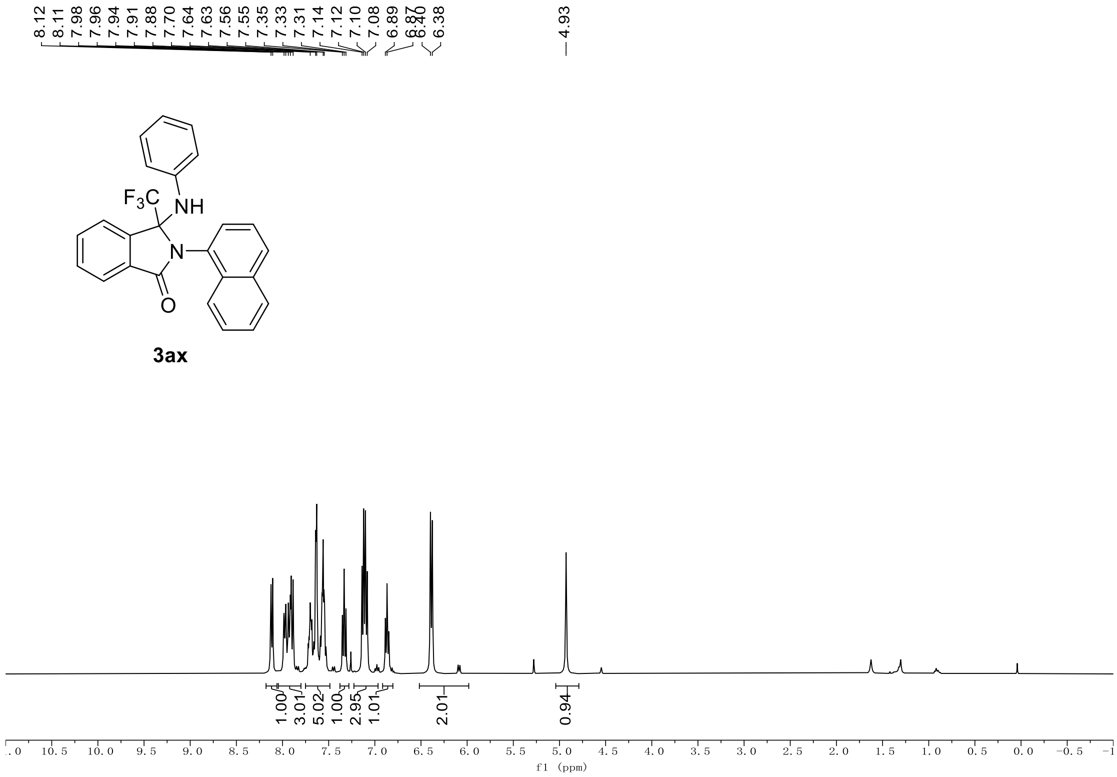

Figure S270. ^{1}H NMR (400 MHz, $CDCl_3$) spectrum of compound 3ax, related to Scheme 3

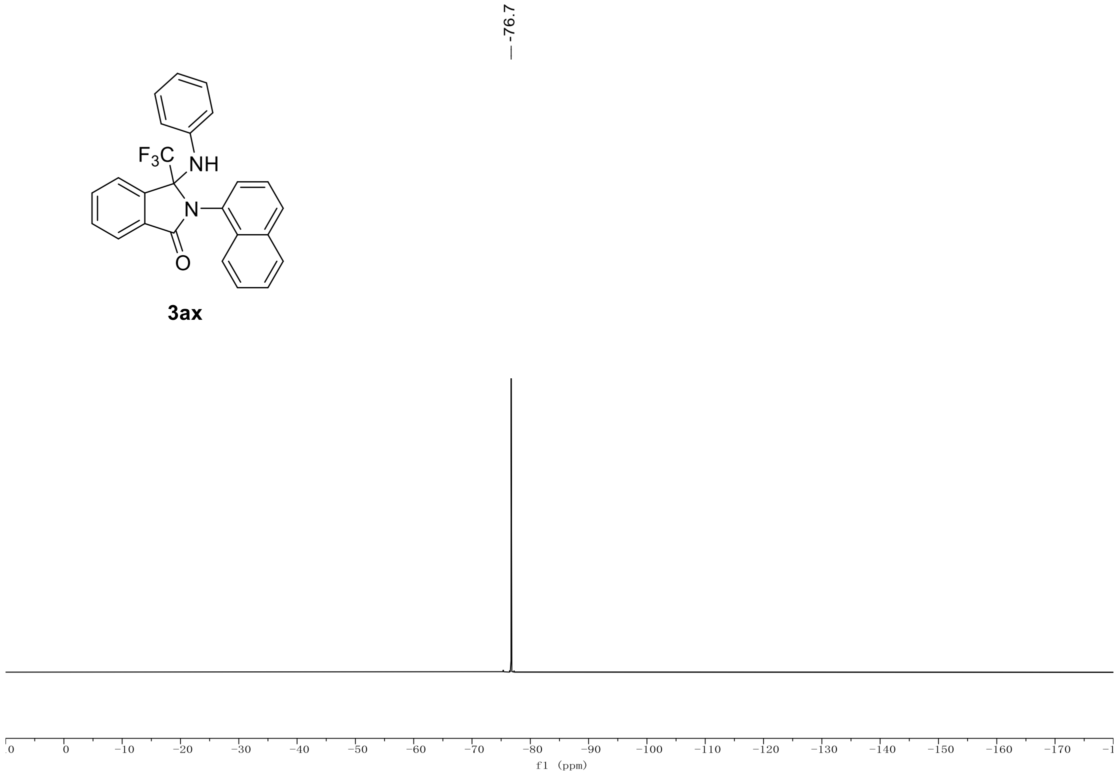

Figure S271. ^{19}**F NMR (376 MHz, CDCl$_3$) spectrum of compound 3ax, related to Scheme 3**

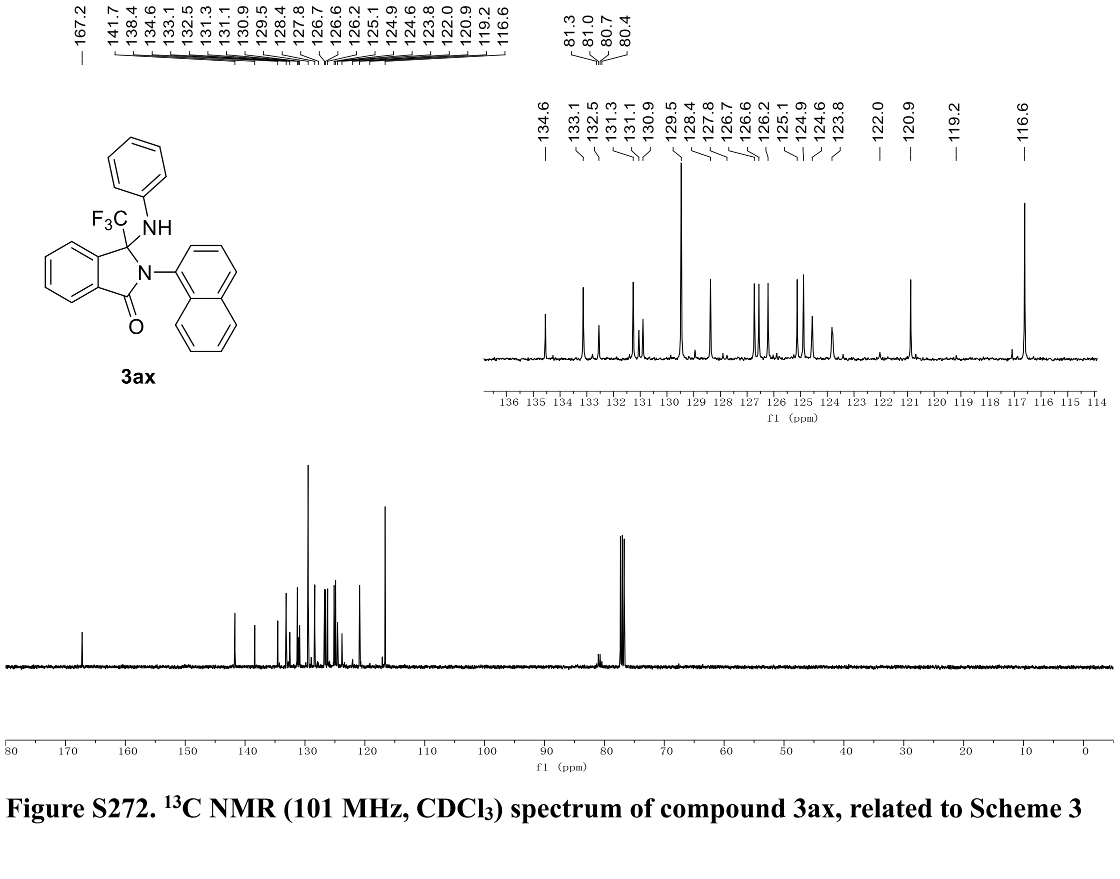

Figure S272. ^{13}**C NMR (101 MHz, CDCl$_3$) spectrum of compound 3ax, related to Scheme 3**

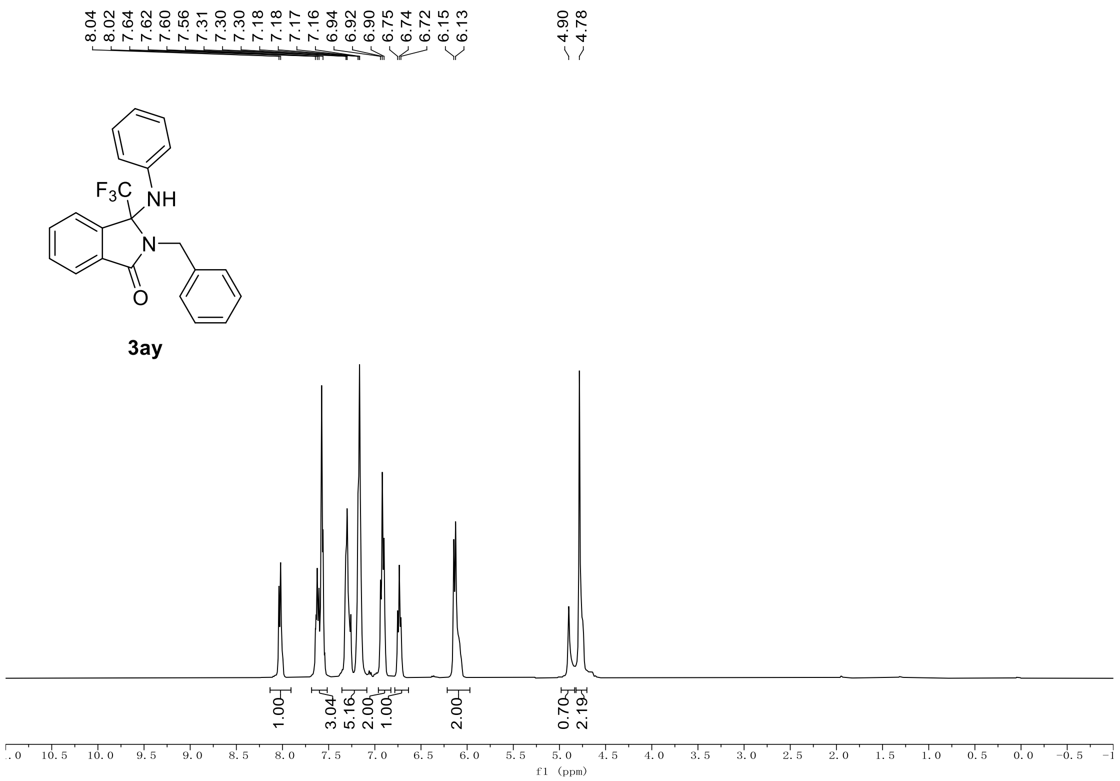

Figure S273. ^{1}H NMR (400 MHz, $CDCl_3$) spectrum of compound 3ay, related to Scheme 3

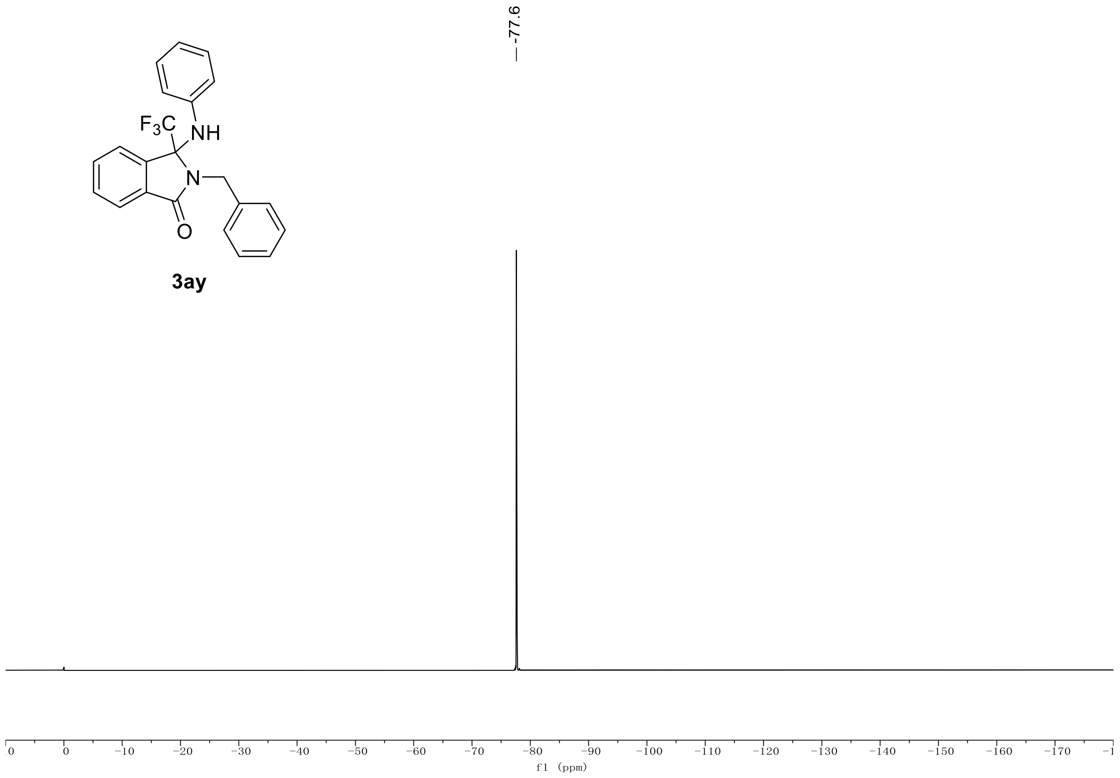

Figure S274. ^{19}F NMR (376 MHz, $CDCl_3$) spectrum of compound 3ay, related to Scheme 3

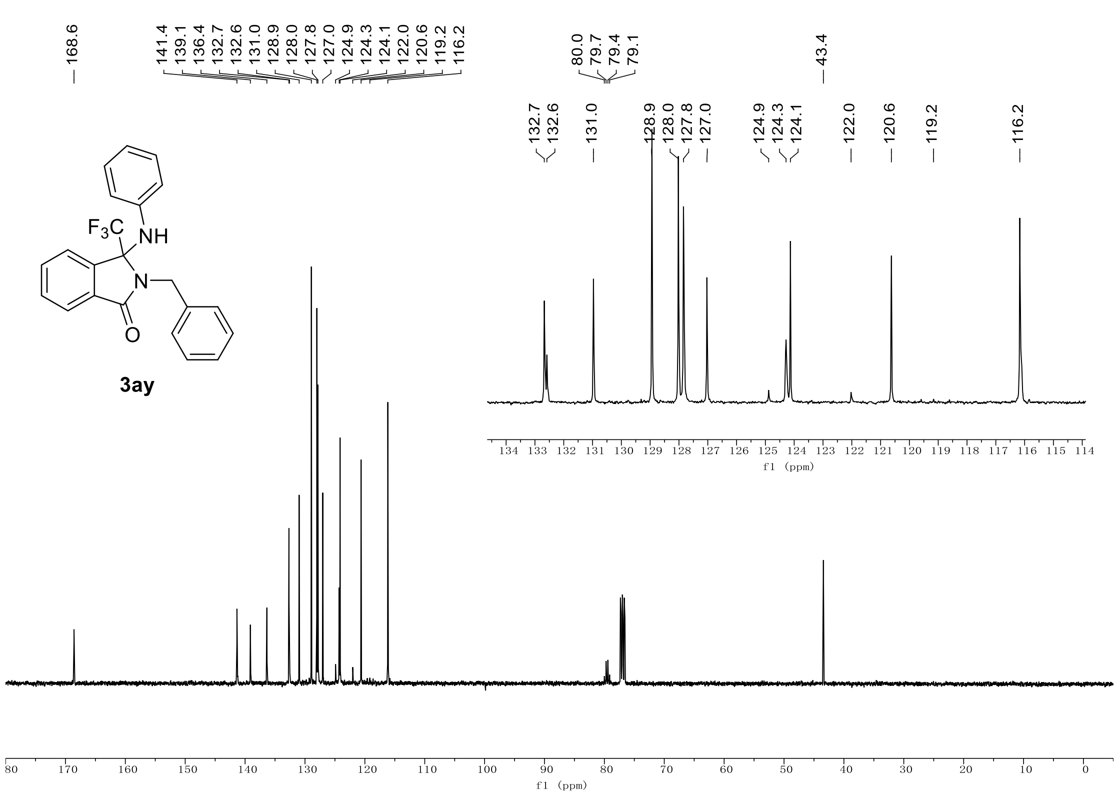

Figure S275. ^{13}C NMR (101 MHz, $CDCl_3$) spectrum of compound 3ay, related to Scheme 3

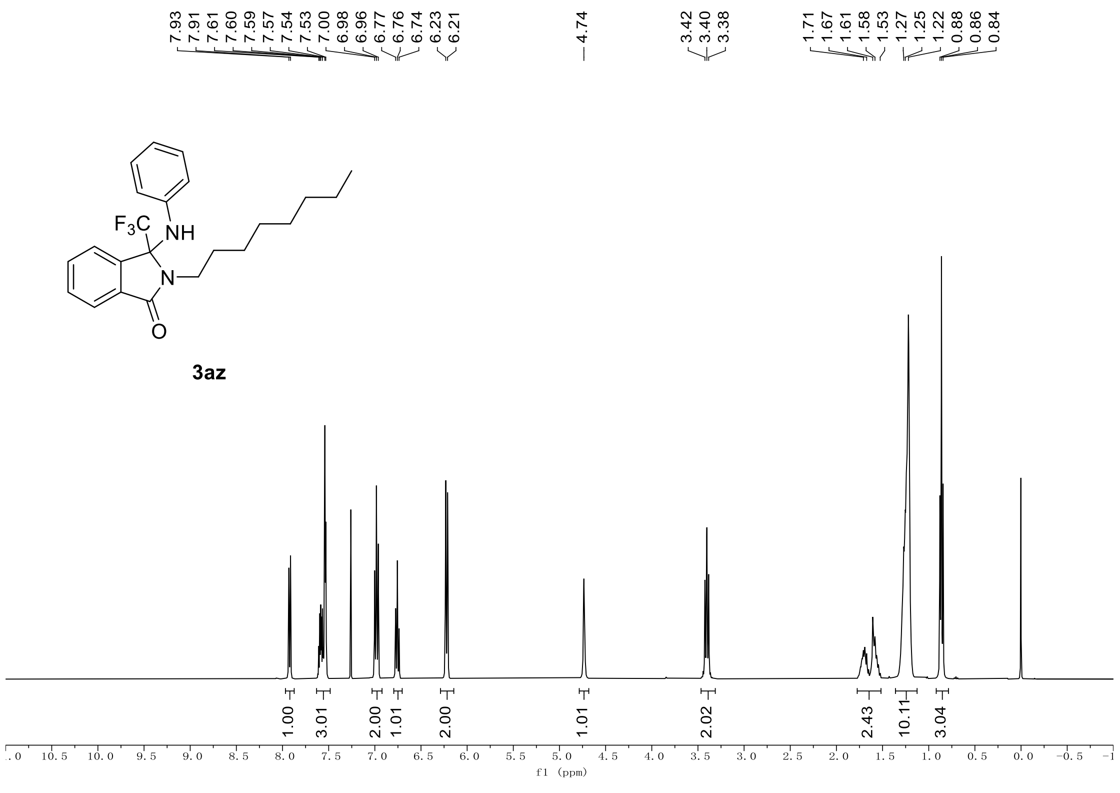

Figure S276. ^{1}H NMR (400 MHz, $CDCl_3$) spectrum of compound 3az, related to Scheme 3

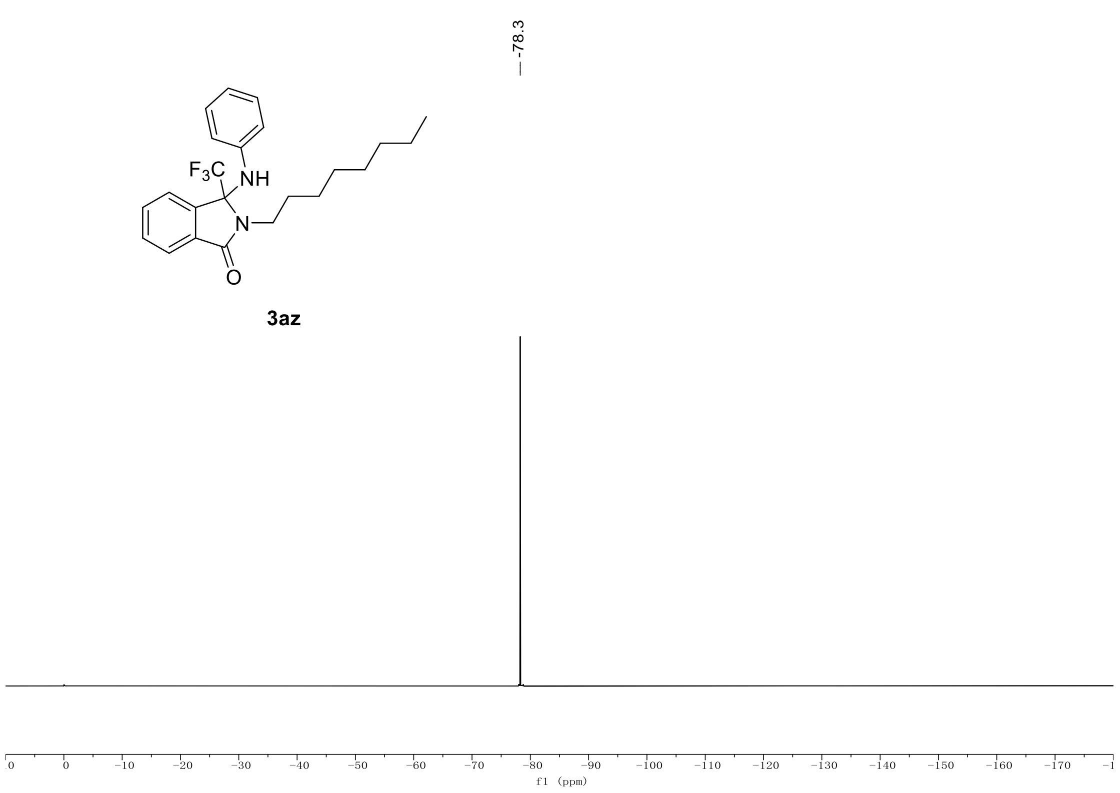

Figure S277. ^{19}F **NMR (376 MHz, CDCl$_3$) spectrum of compound 3az, related to Scheme 3**

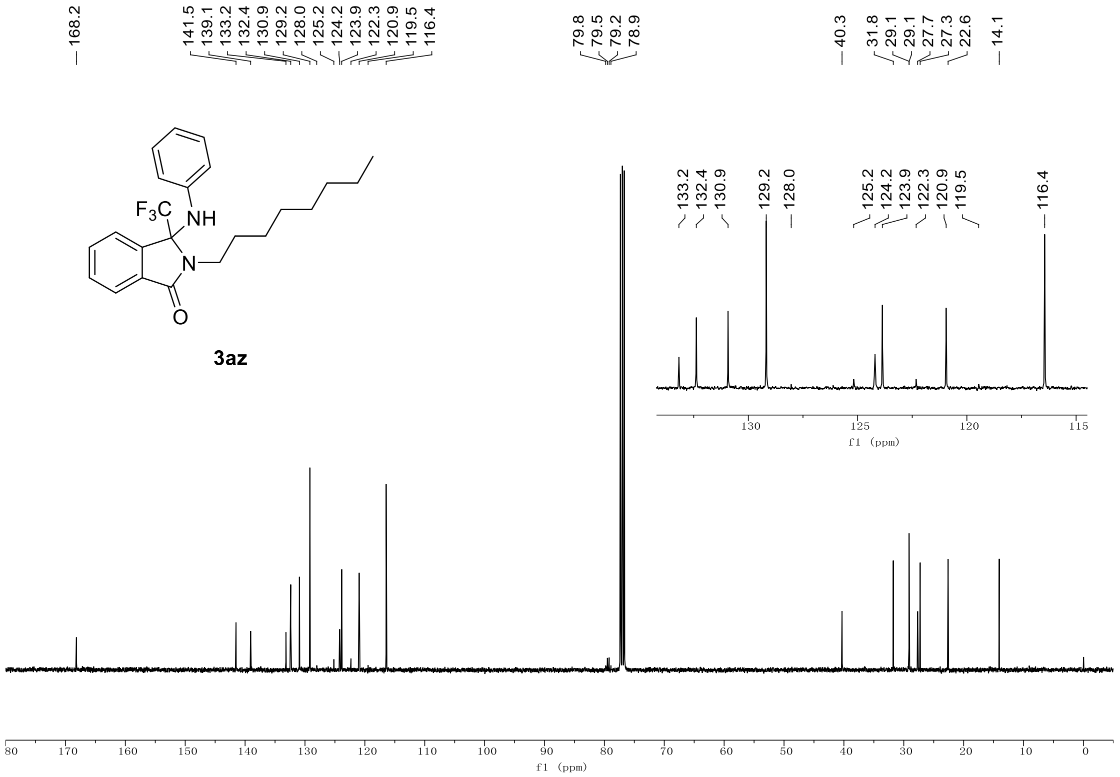

Figure S278. ^{13}C **NMR (101 MHz, CDCl$_3$) spectrum of compound 3az, related to Scheme 3**

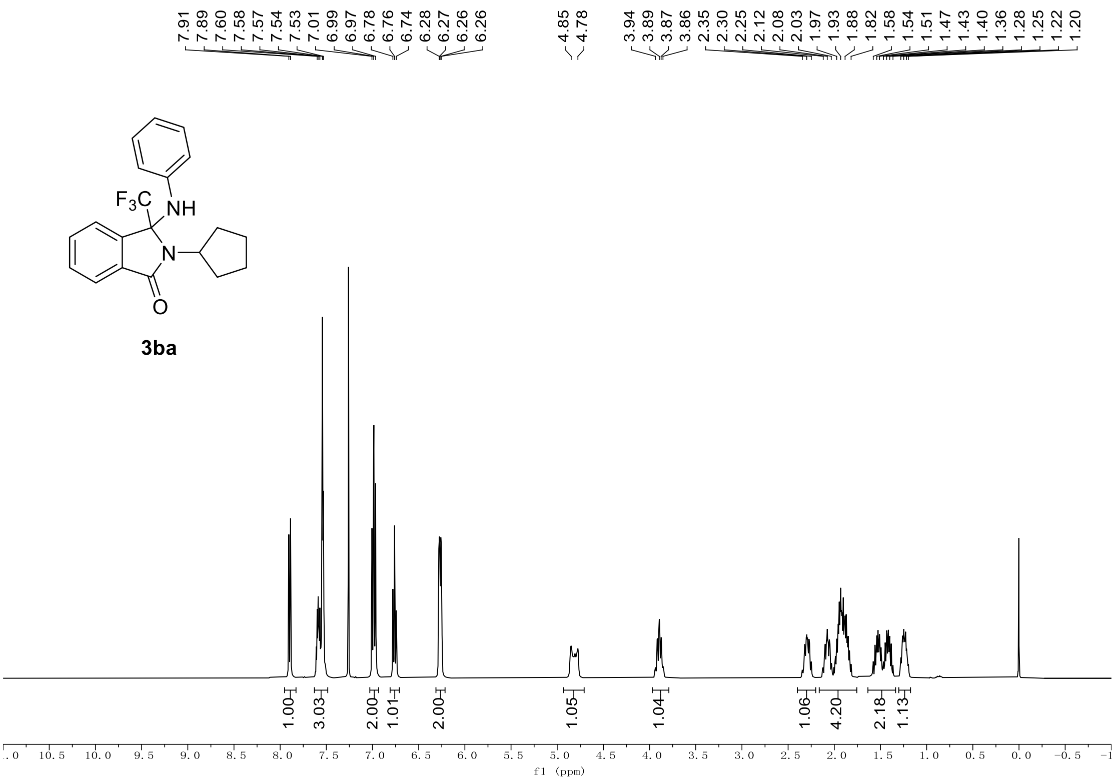

Figure S279. ^{1}H **NMR (400 MHz,** $CDCl_3$**) spectrum of compound 3ba, related to Scheme 3**

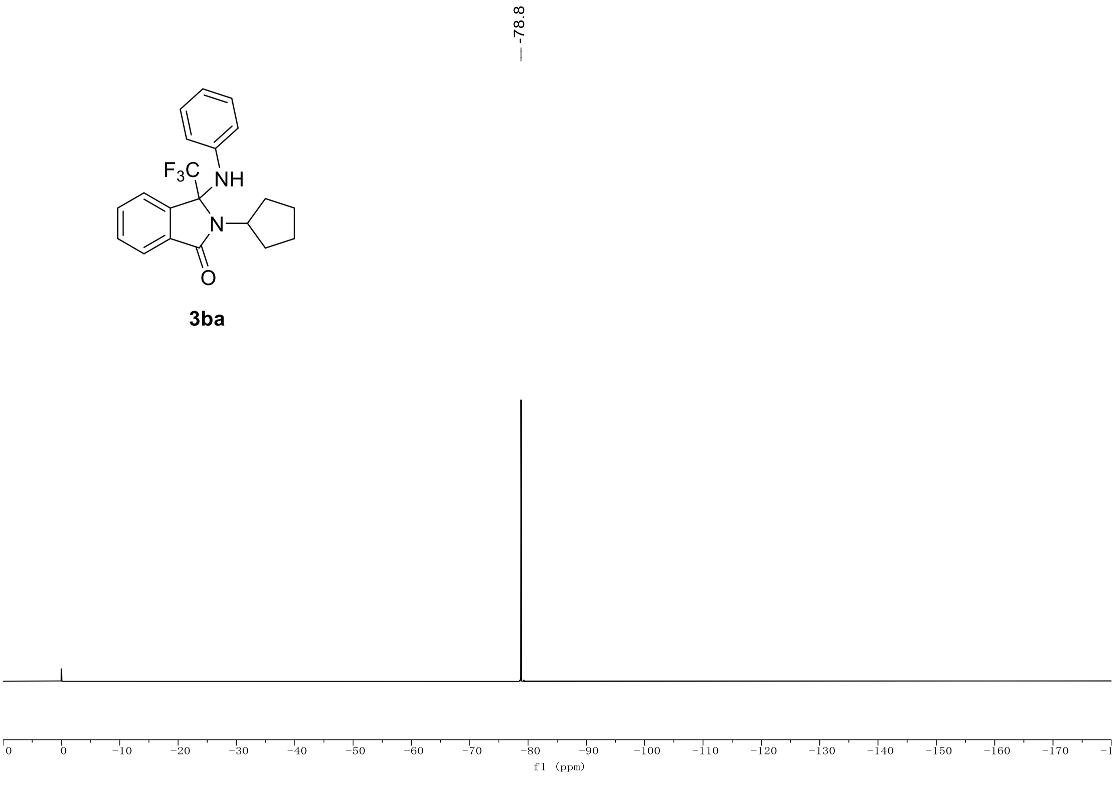

Figure S280. ^{19}F **NMR (376 MHz,** $CDCl_3$**) spectrum of compound 3ba, related to Scheme 3**

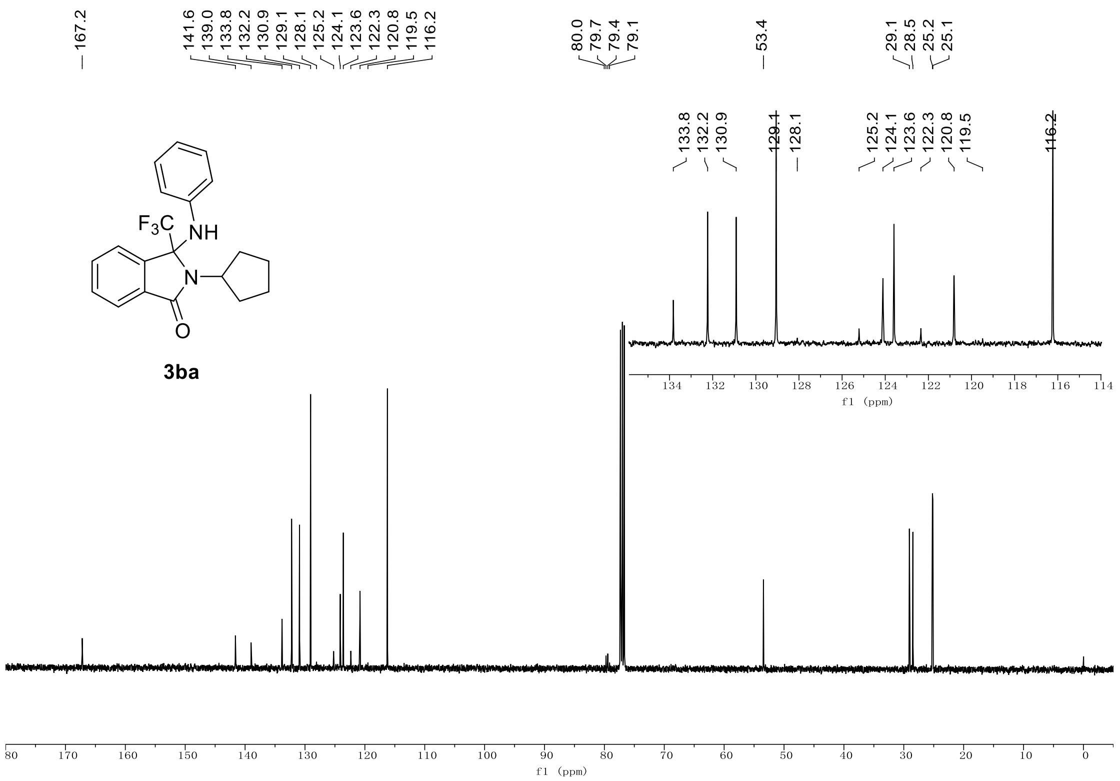

Figure S281. ^{13}**C NMR (101 MHz, CDCl**$_3$**) spectrum of compound 3ba, related to Scheme 3**

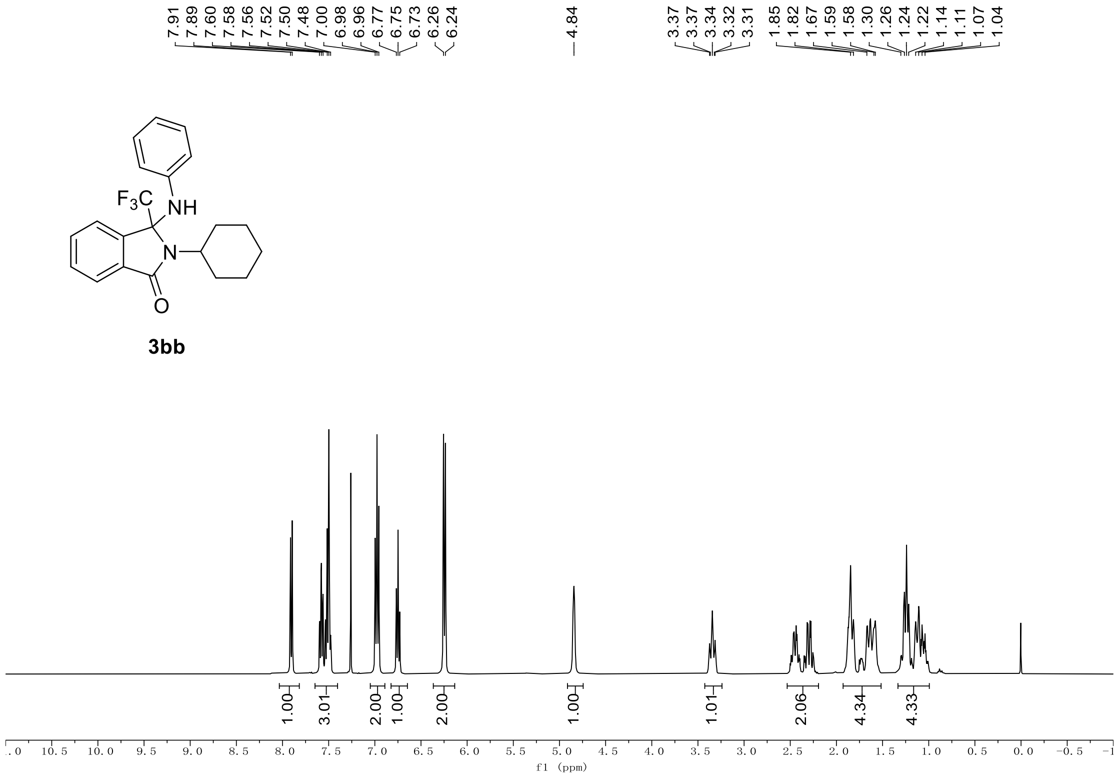

Figure S282. ^{1}**H NMR (400 MHz, CDCl**$_3$**) spectrum of compound 3bb, related to Scheme 3**

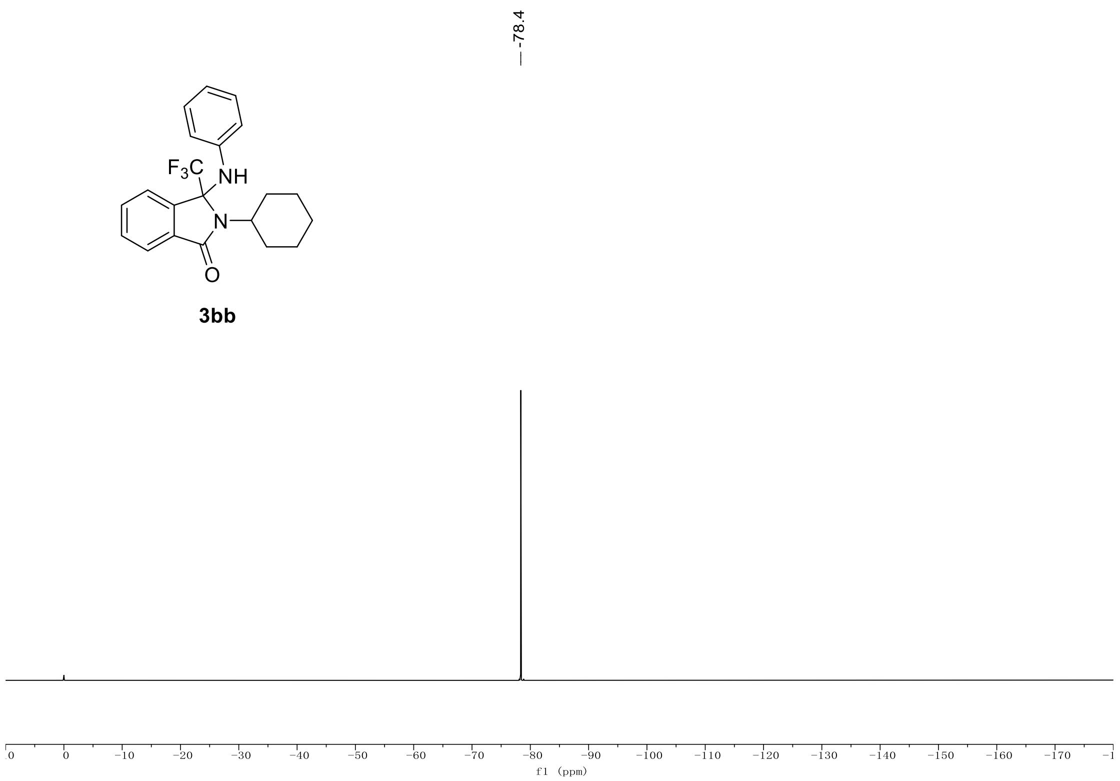

Figure S283. ^{19}F NMR (376 MHz, $CDCl_3$) spectrum of compound 3bb, related to Scheme 3

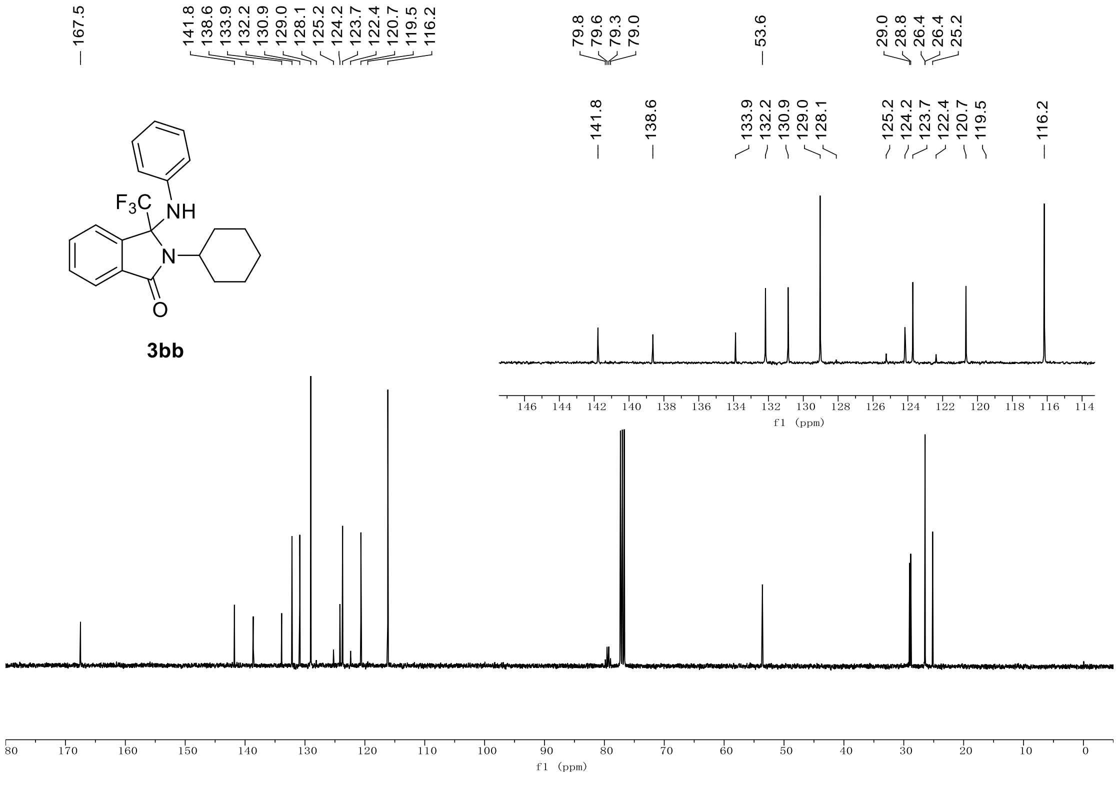

Figure S284. ^{13}C NMR (101 MHz, $CDCl_3$) spectrum of compound 3bb, related to Scheme 3

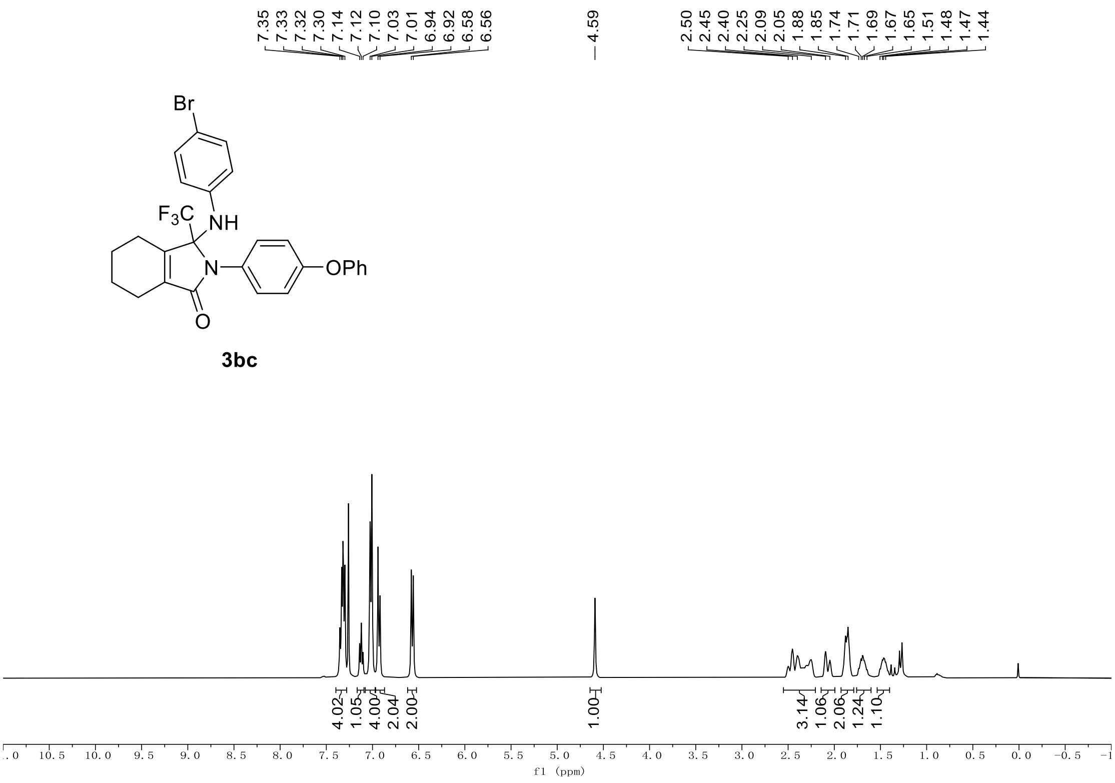

Figure S285. ^{1}H NMR (400 MHz, $CDCl_3$) spectrum of compound 3bc, related to Scheme 3

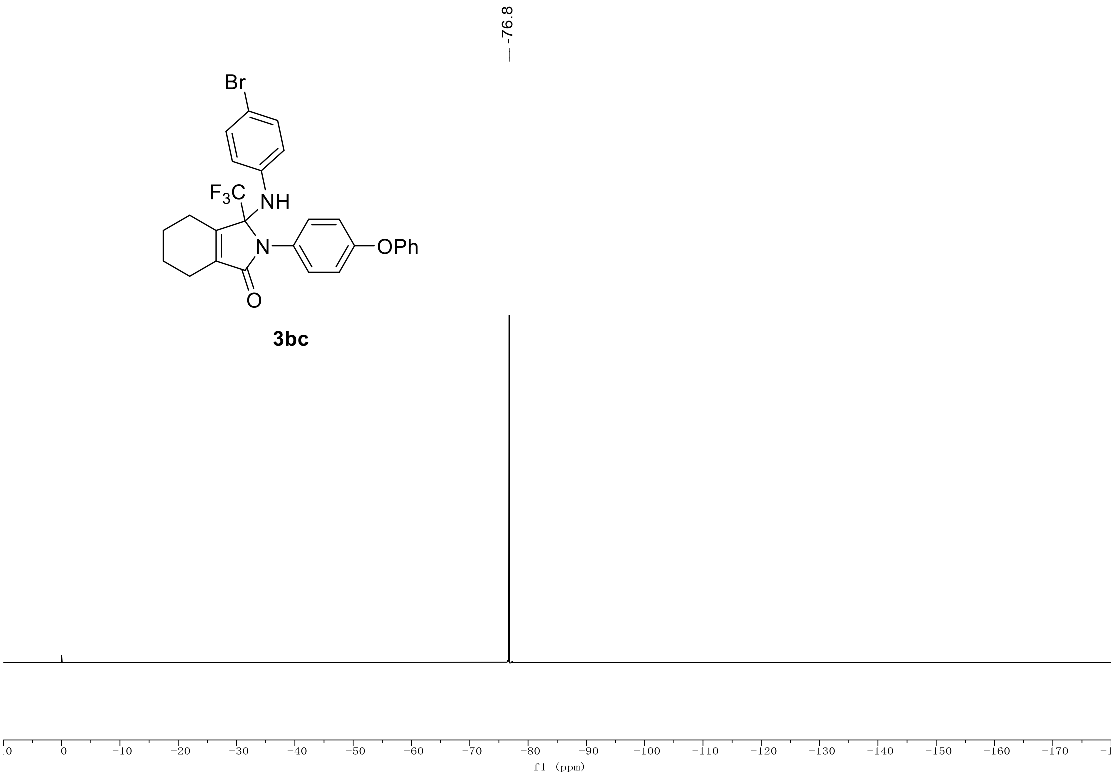

Figure S286. ^{19}F NMR (376 MHz, $CDCl_3$) spectrum of compound 3bc, related to Scheme 3

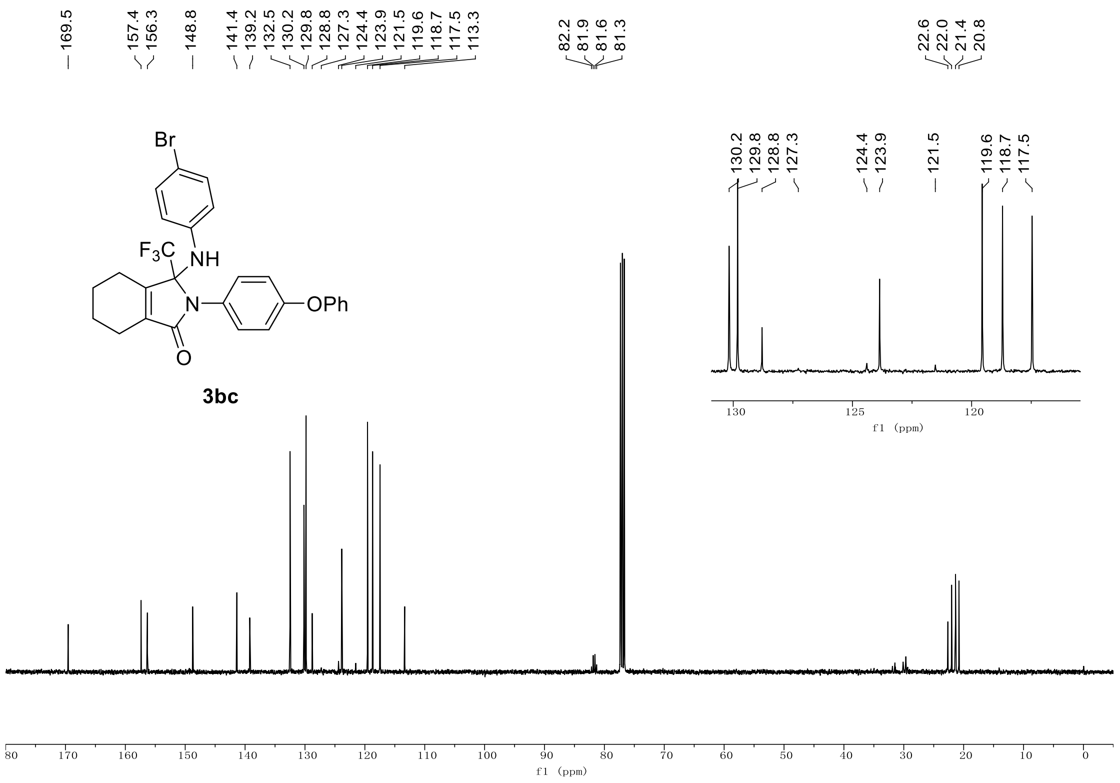

Figure S287. ^{13}C NMR (101 MHz, CDCl$_3$) spectrum of compound 3bc, related to Scheme 3

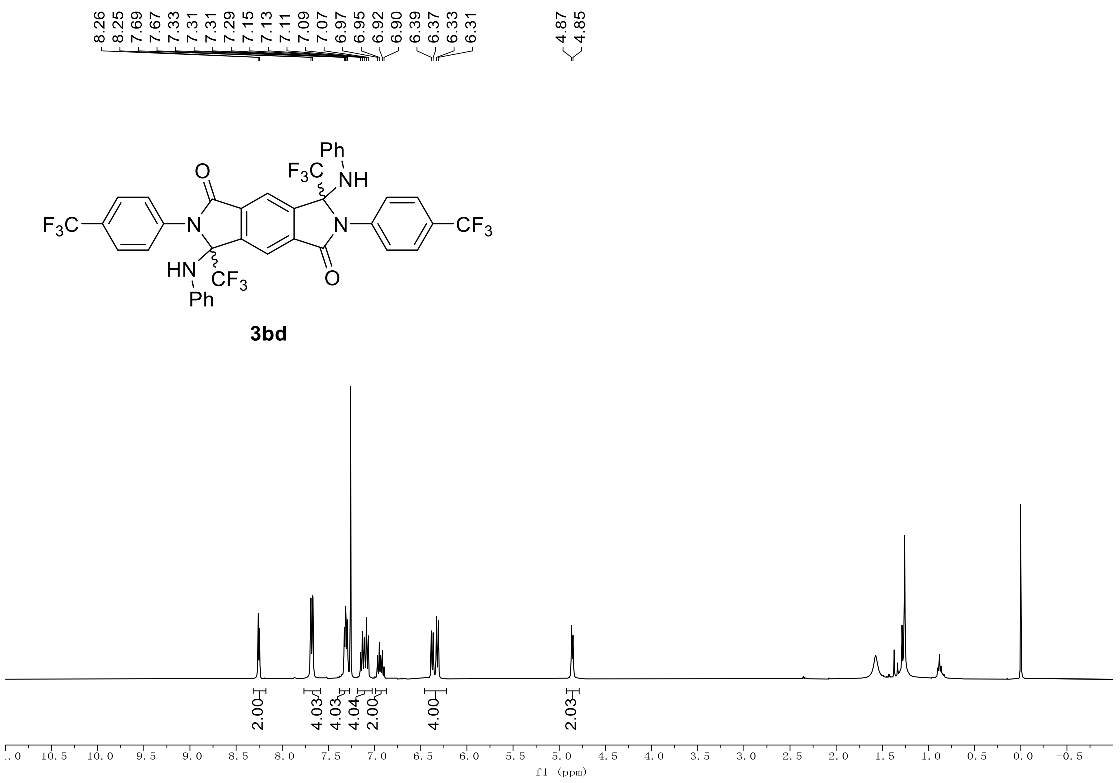

Figure S288. ^{1}H NMR (400 MHz, CDCl$_3$) spectrum of compound 3bd, related to Scheme 3

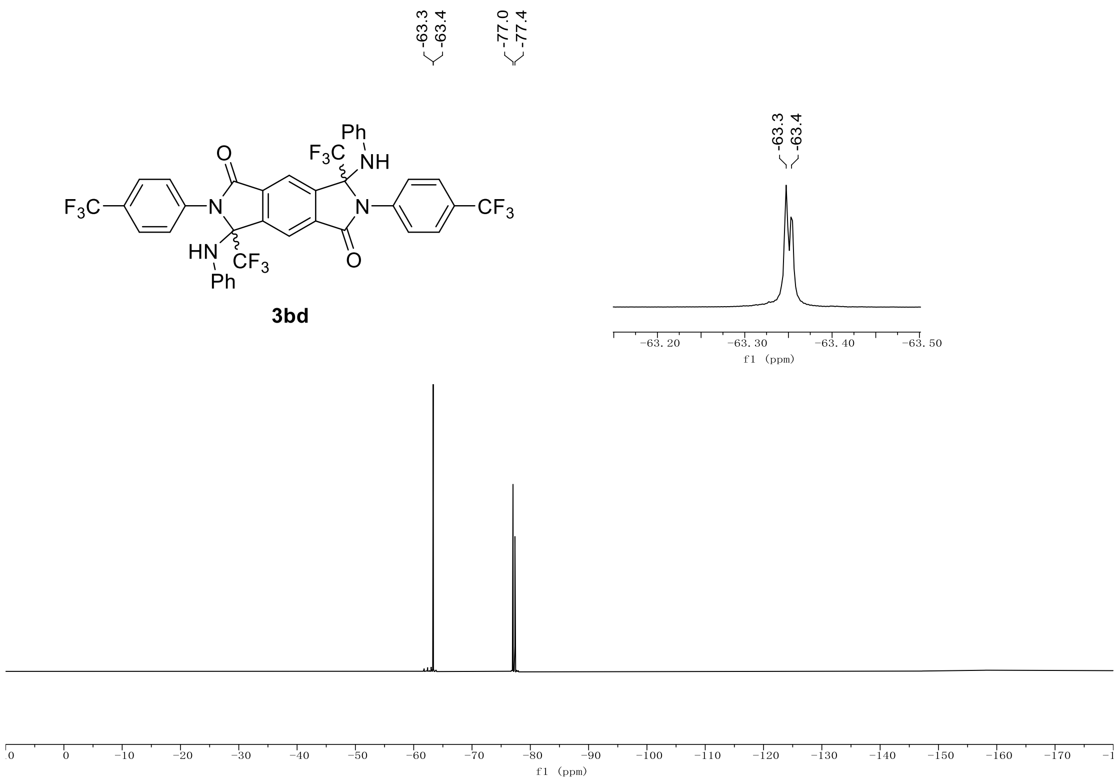

Figure S289. ^{19}F **NMR (376 MHz, CDCl$_3$) spectrum of compound 3bd, related to Scheme 3**

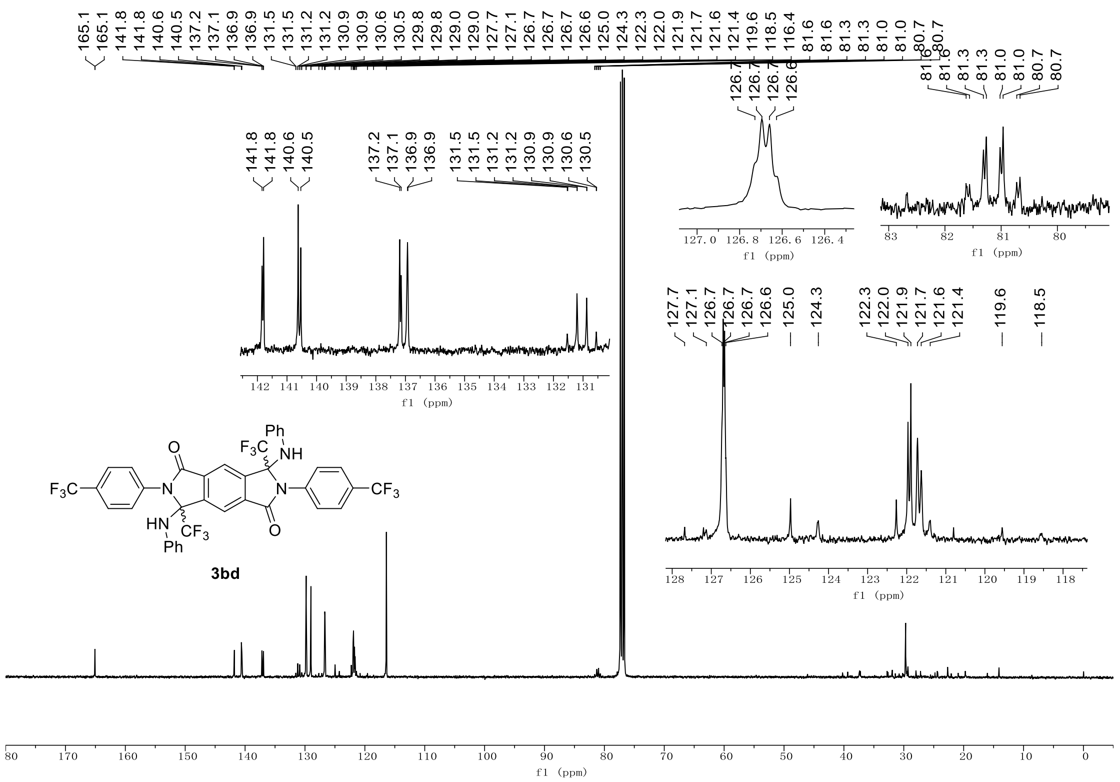

Figure S290. ^{13}C **NMR (101 MHz, CDCl$_3$) spectrum of compound 3bd, related to Scheme 3**

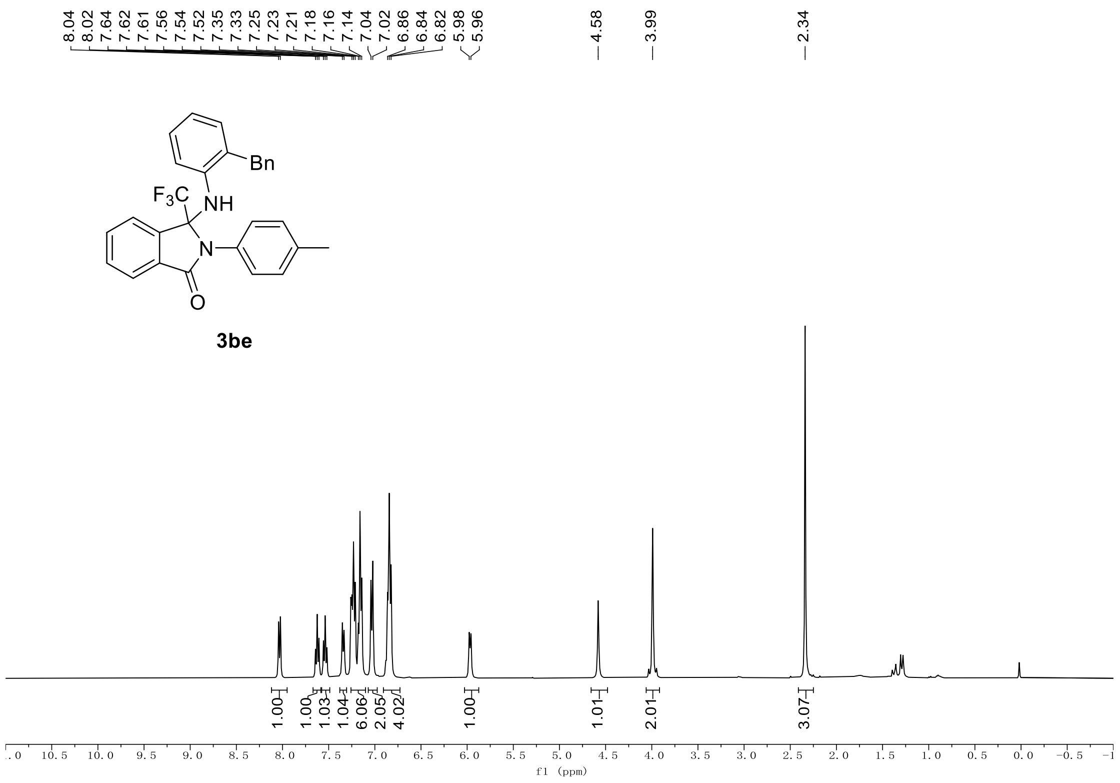

Figure S291. ^{1}H NMR (400 MHz, $CDCl_3$) spectrum of compound 3be, related to Scheme 3

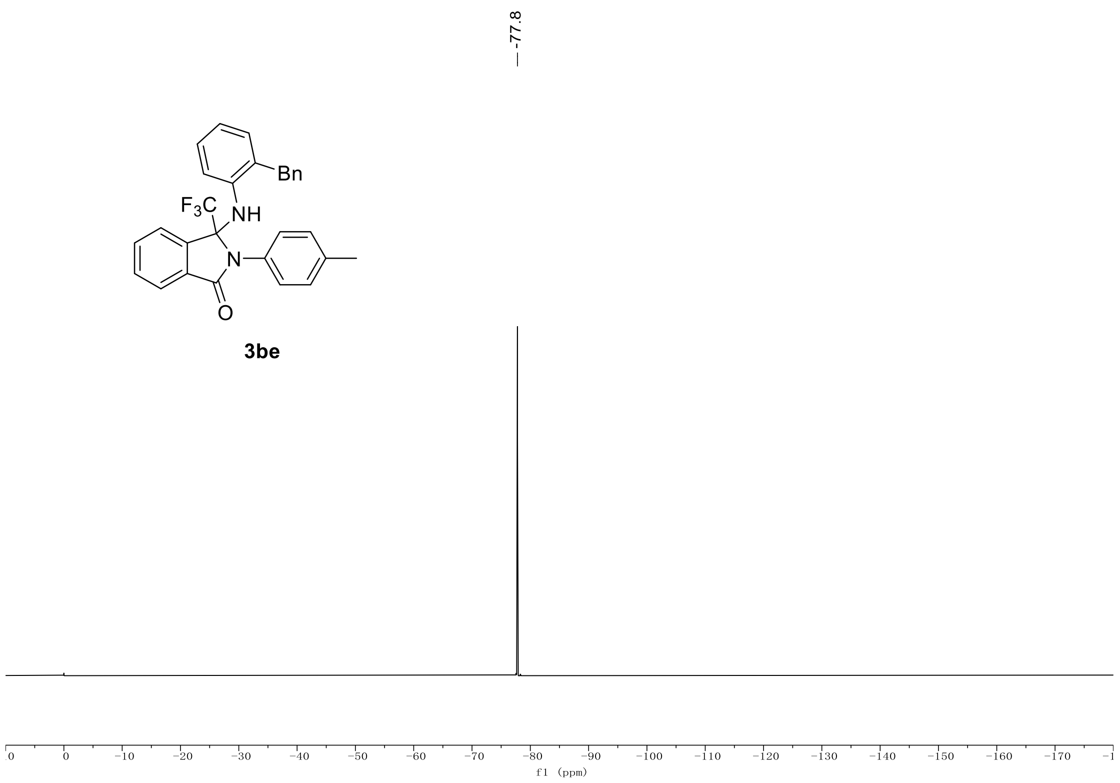

Figure S292. ^{19}F NMR (376 MHz, $CDCl_3$) spectrum of compound 3be, related to Scheme 3

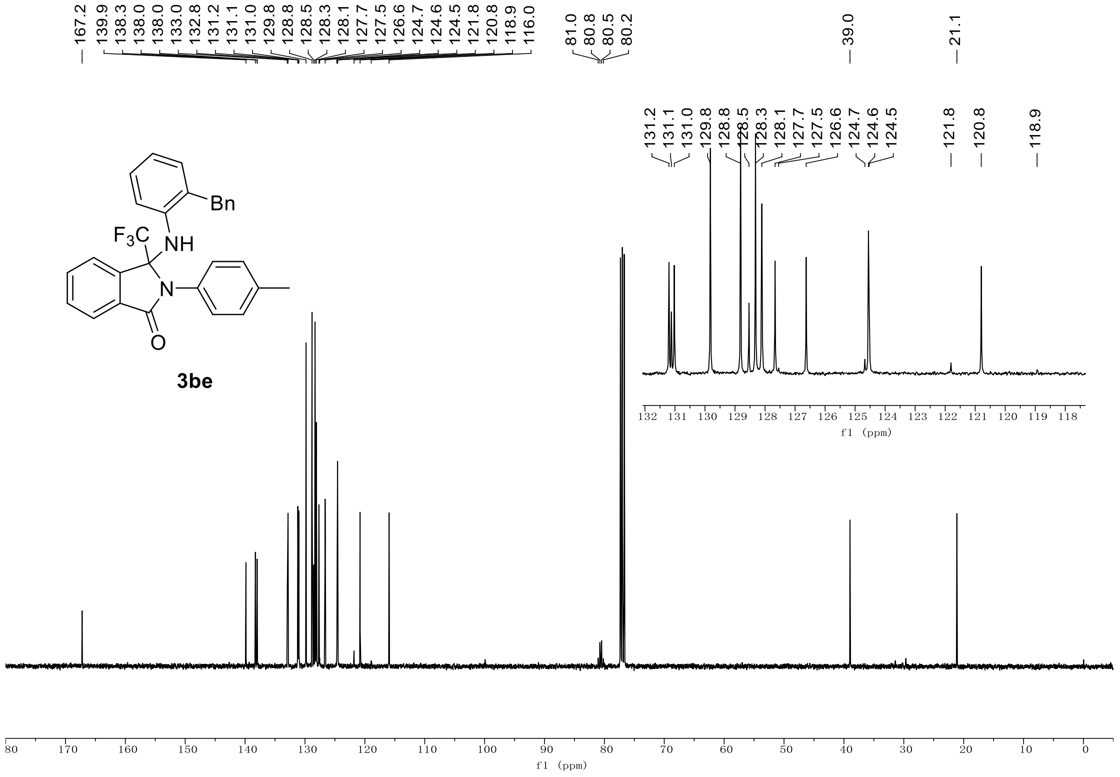

Figure S293. ^{13}C NMR (101 MHz, $CDCl_3$) spectrum of compound 3be, related to Scheme 3

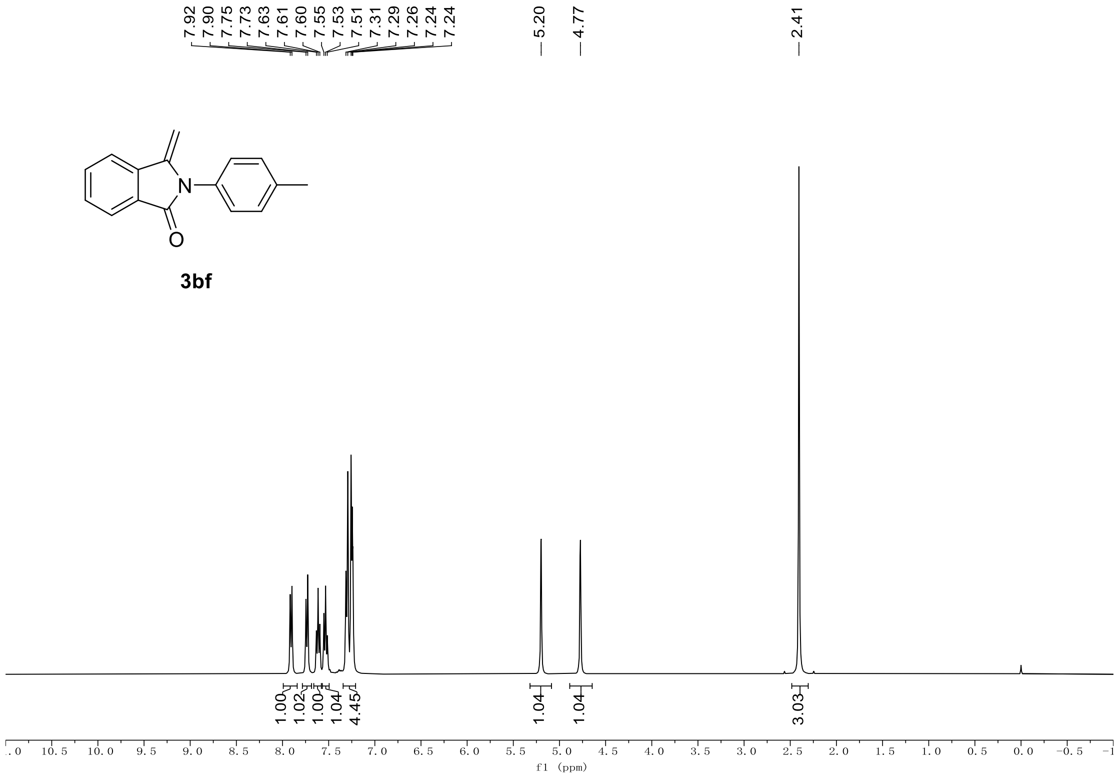

Figure S294. ^{1}H NMR (400 MHz, $CDCl_3$) spectrum of compound 3bf, related to Scheme 5

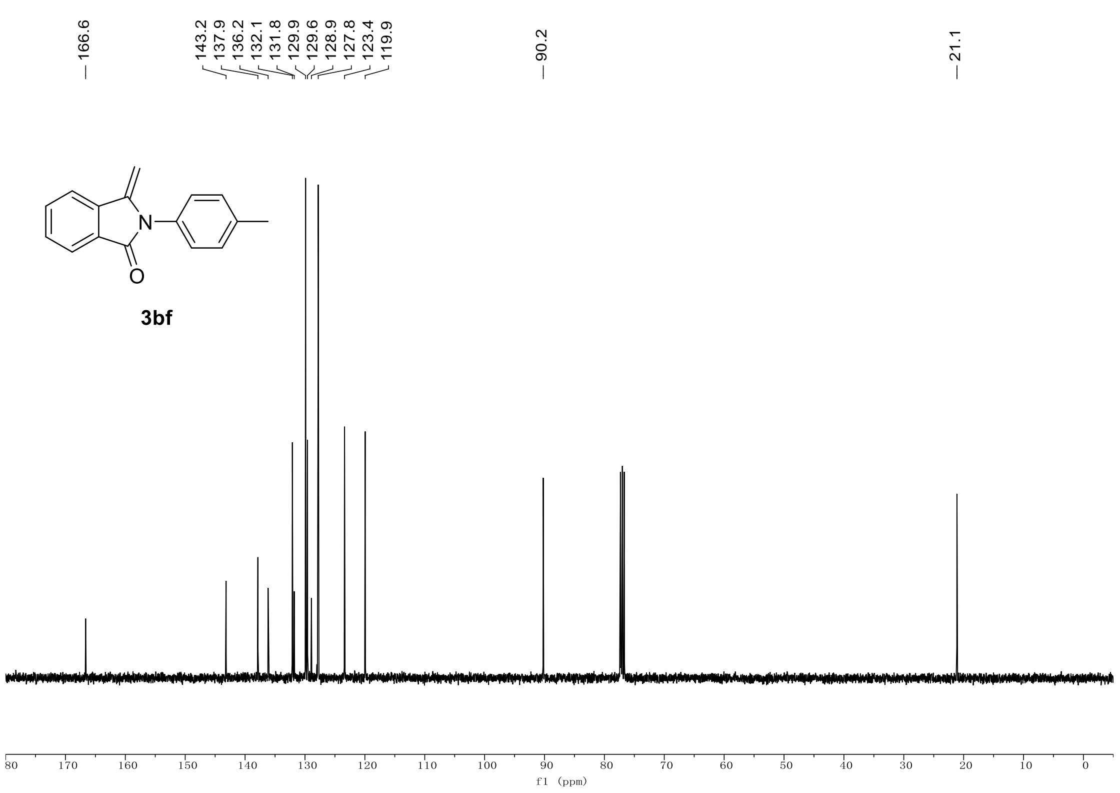

Figure S295. ^{13}C NMR (101 MHz, $CDCl_3$) spectrum of compound 3bf, related to Scheme 5

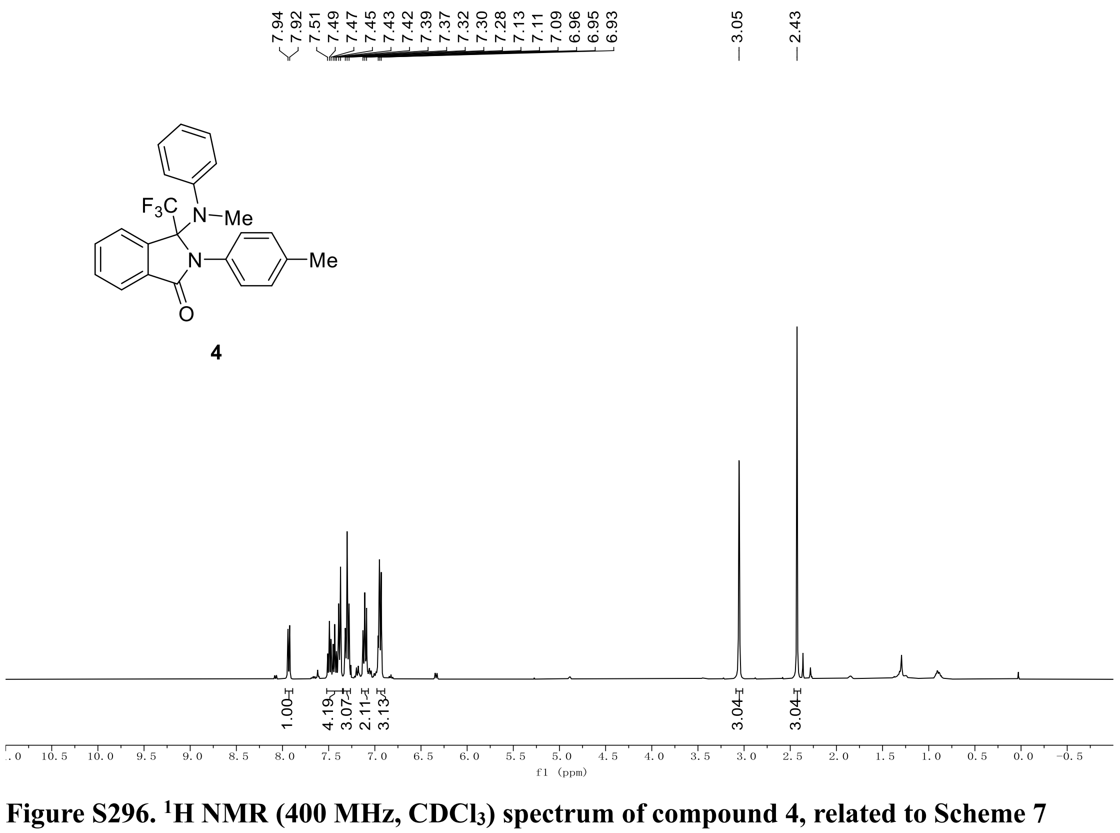

Figure S296. ^{1}H NMR (400 MHz, $CDCl_3$) spectrum of compound 4, related to Scheme 7

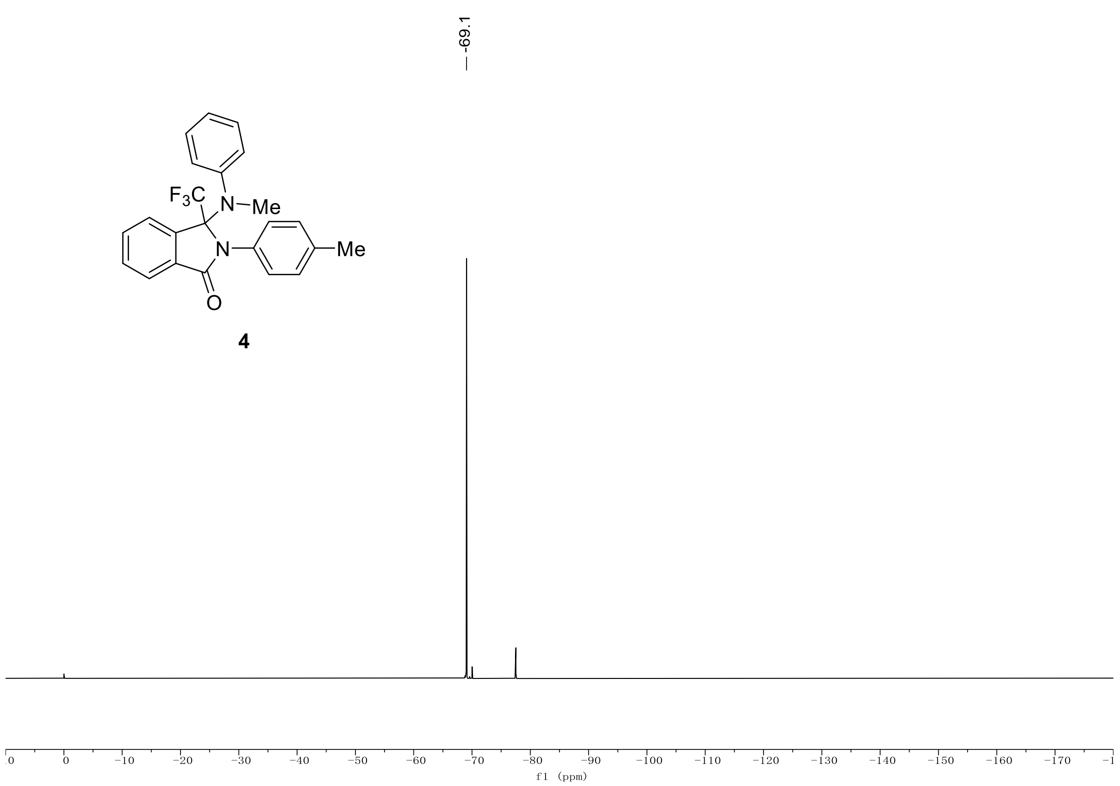

Figure S297. ^{19}F NMR (376 MHz, CDCl$_3$) spectrum of compound 4, related to Scheme 7

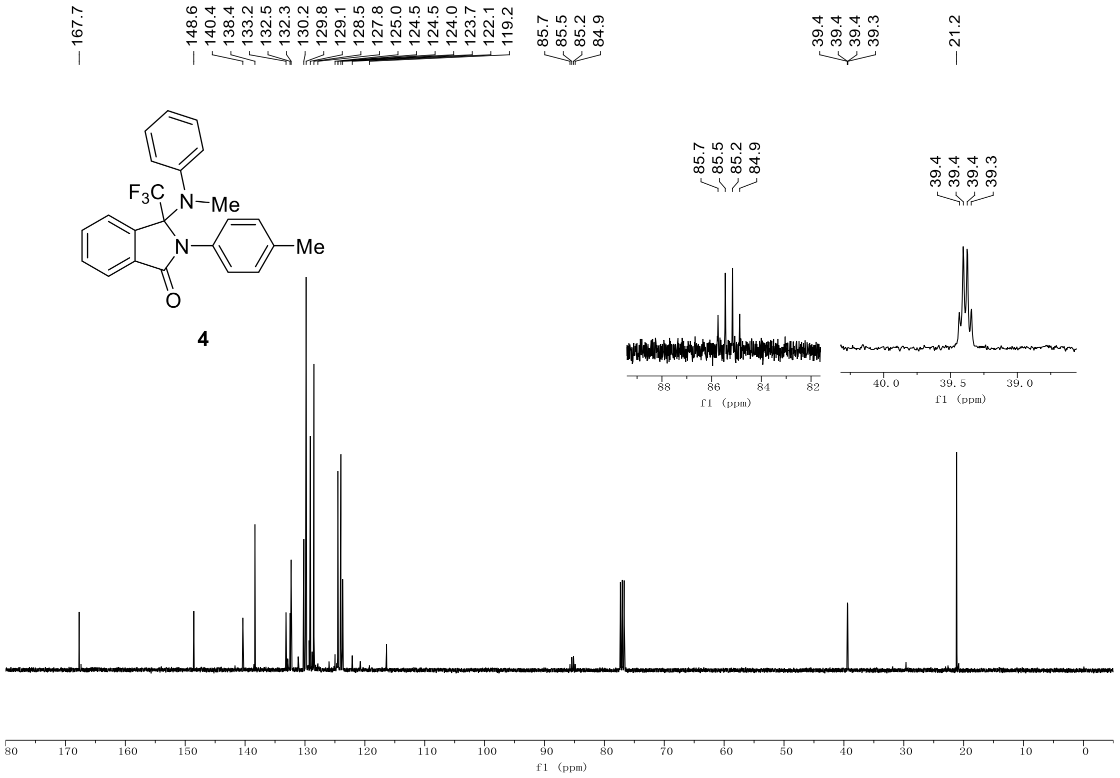

Figure S298. ^{13}C NMR (101 MHz, CDCl$_3$) spectrum of compound 4, related to Scheme 7

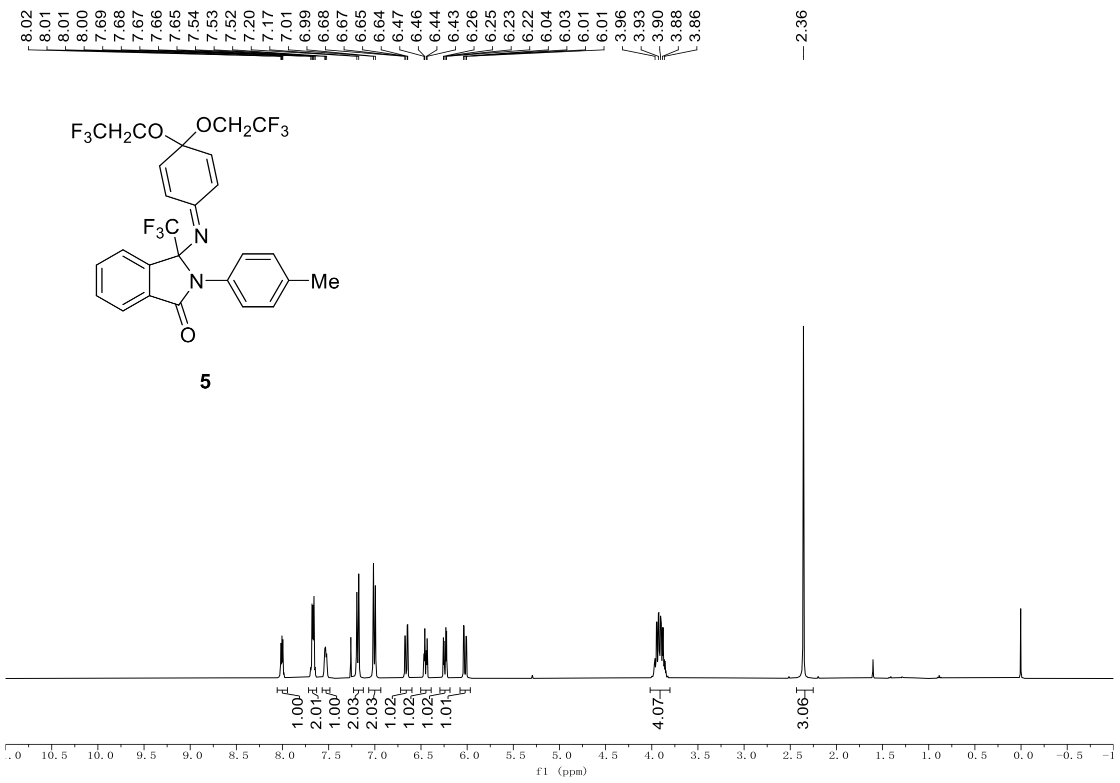

Figure S299. **^{1}H NMR (400 MHz, $CDCl_3$) spectrum of compound 5, related to Scheme 7**

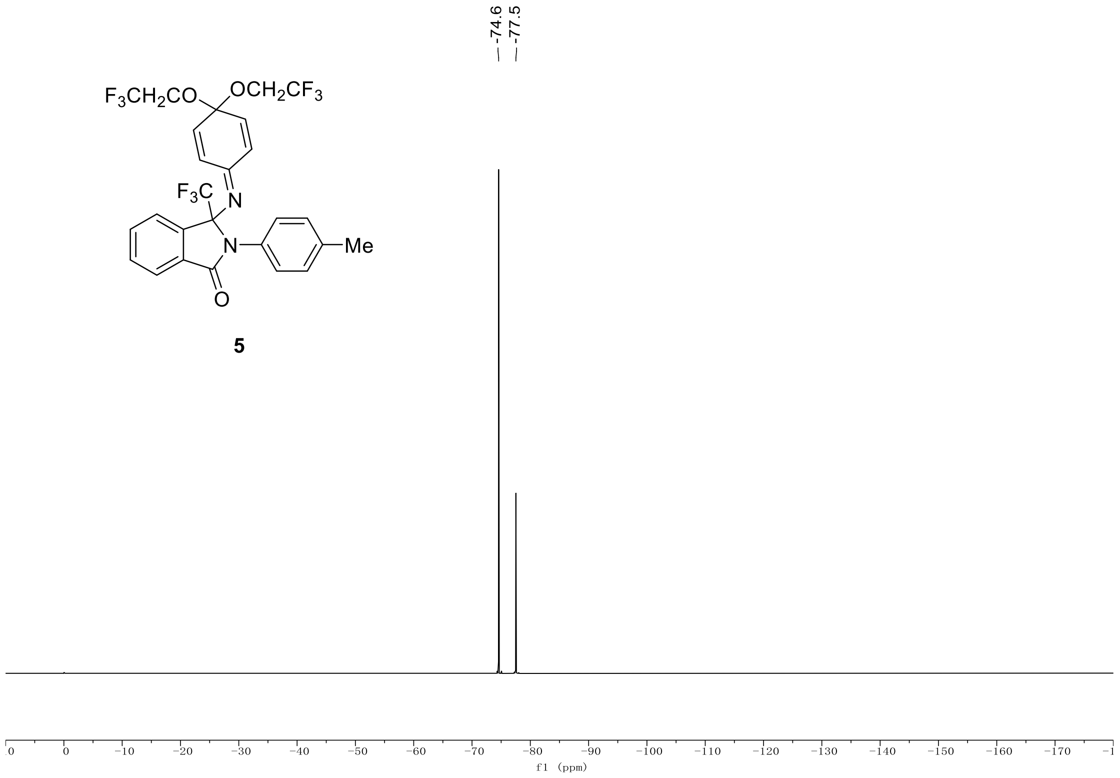

Figure S300. **^{19}F NMR (376 MHz, $CDCl_3$) spectrum of compound 5, related to Scheme 7**

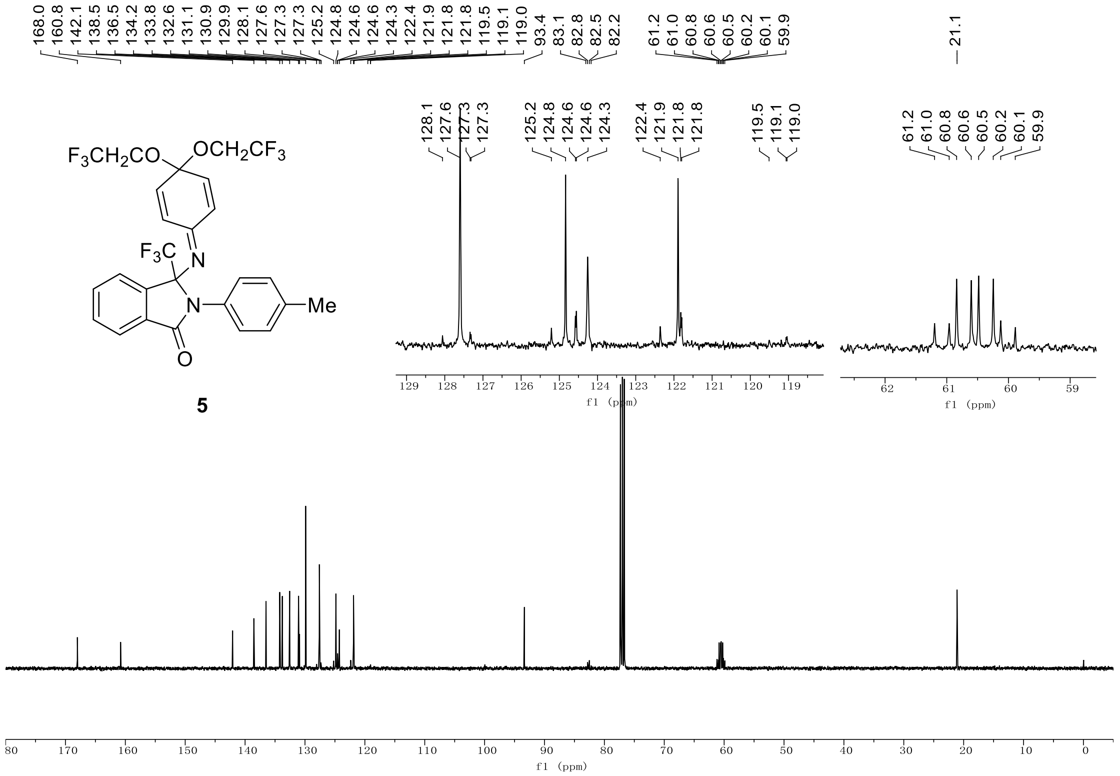

Figure S301. ^{13}C NMR (101 MHz, $CDCl_3$) spectrum of compound 5, related to Scheme 7

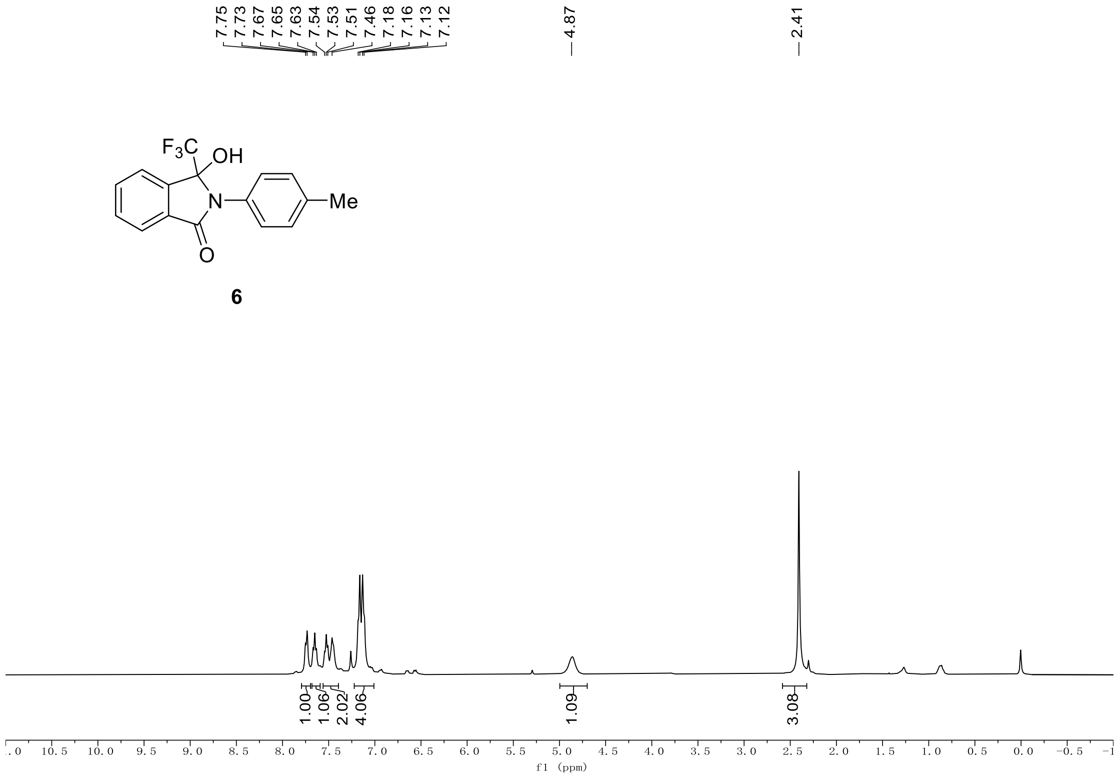

Figure S302. ^{1}H NMR (400 MHz, $CDCl_3$) spectrum of compound 6, related to Scheme 7

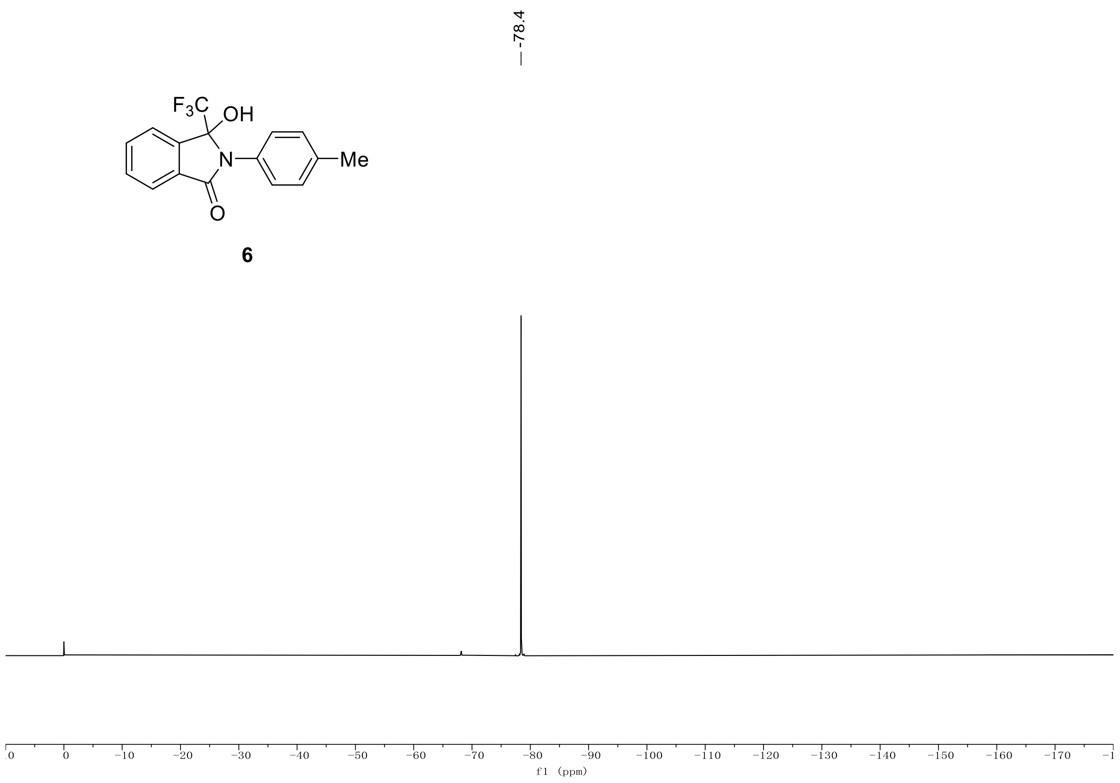

Figure S303. ^{19}F NMR (376 MHz, CDCl3) spectrum of compound 6, related to Scheme 7

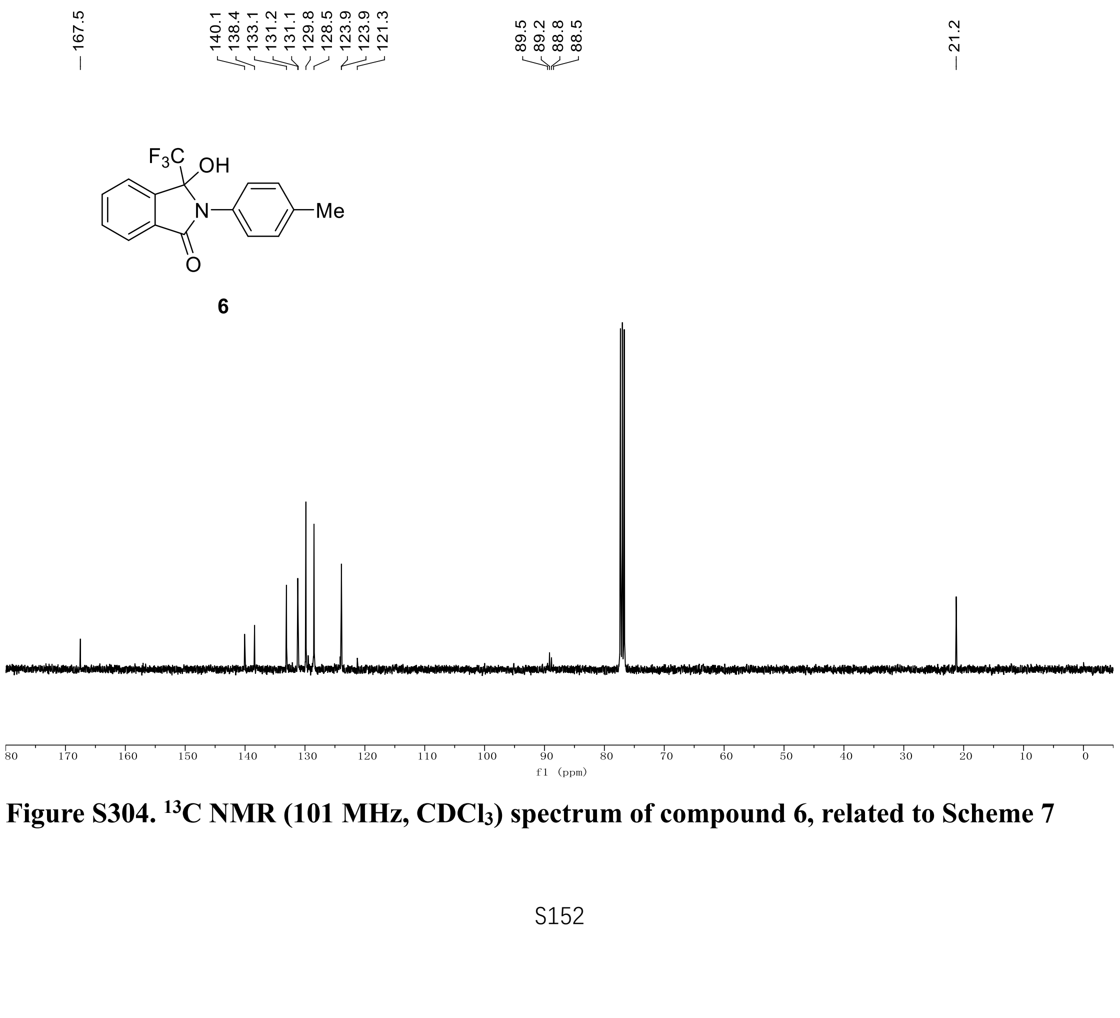

Figure S304. ^{13}C NMR (101 MHz, CDCl3) spectrum of compound 6, related to Scheme 7

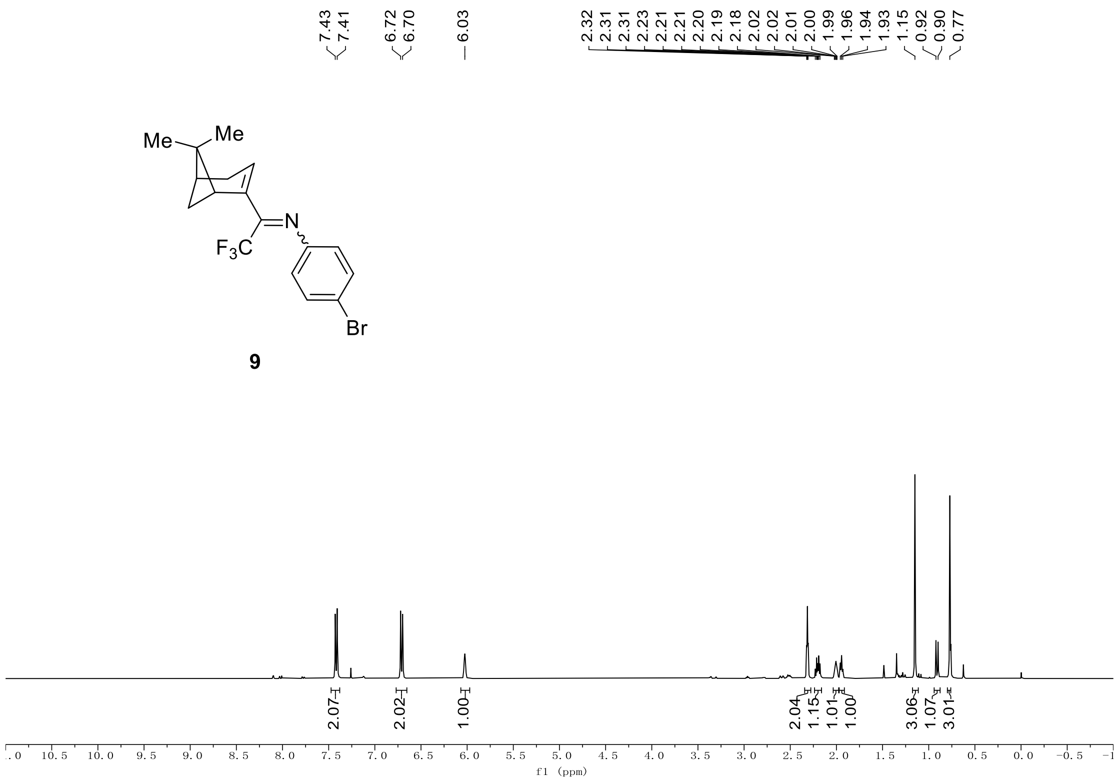

Figure S305. ^{1}H NMR (400 MHz, $CDCl_3$) spectrum of compound 9, related to Scheme 8

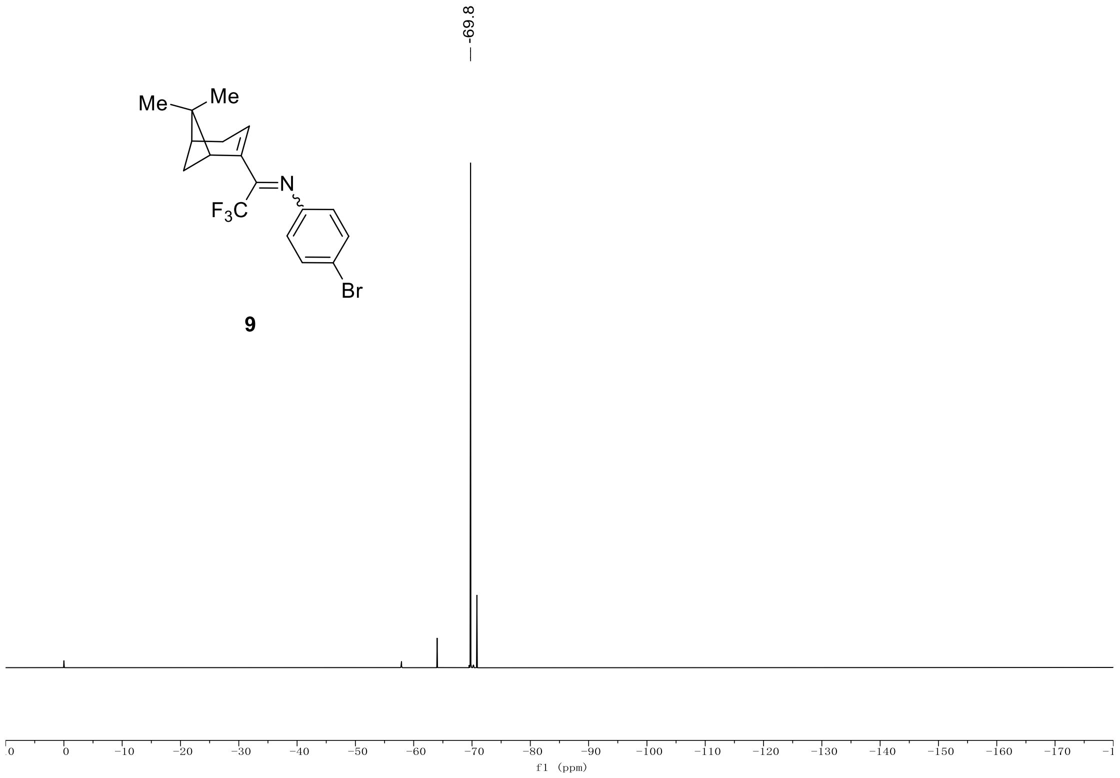

Figure S306. ^{19}F NMR (376 MHz, $CDCl_3$) spectrum of compound 9, related to Scheme 8

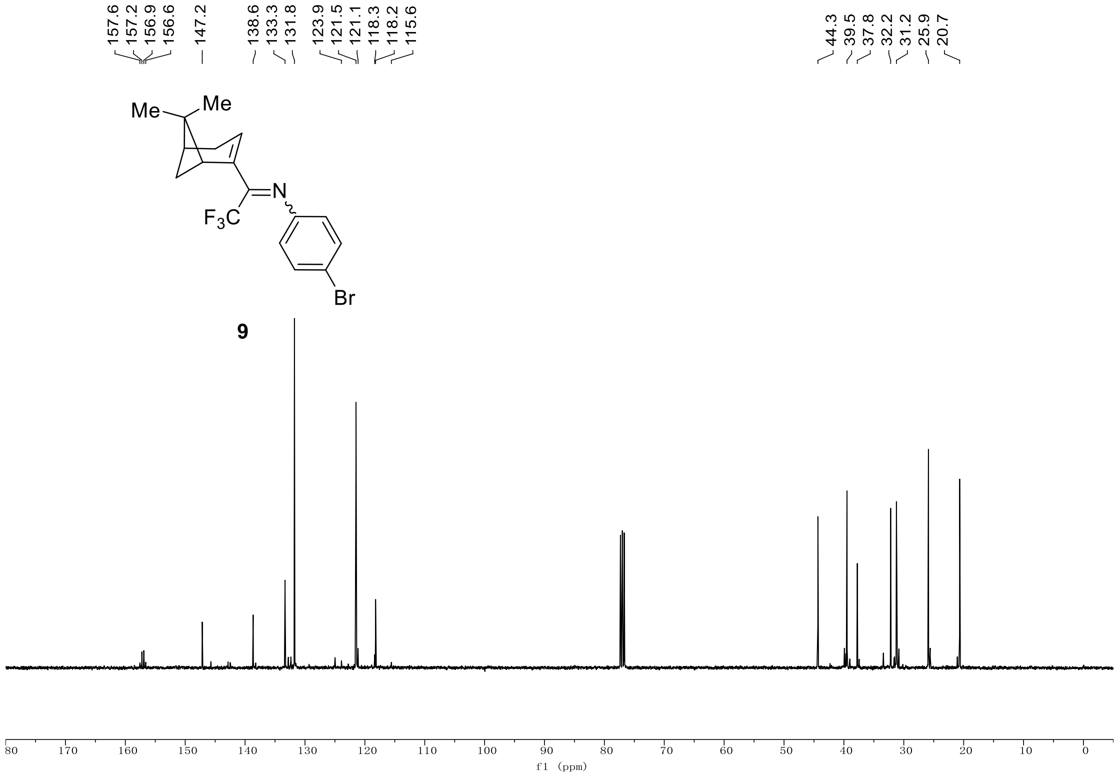

Figure S307. ^{13}C NMR (101 MHz, $CDCl_3$) spectrum of compound 9, related to Scheme 8

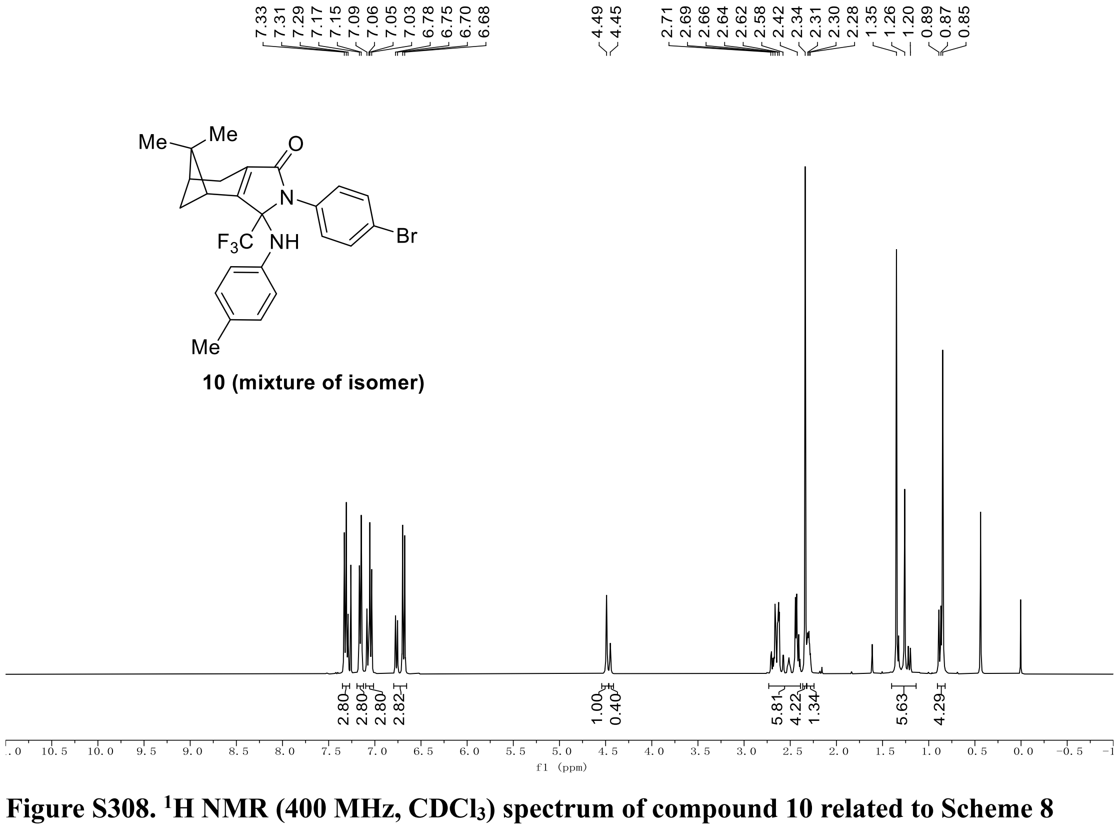

Figure S308. ^{1}H NMR (400 MHz, $CDCl_3$) spectrum of compound 10 related to Scheme 8

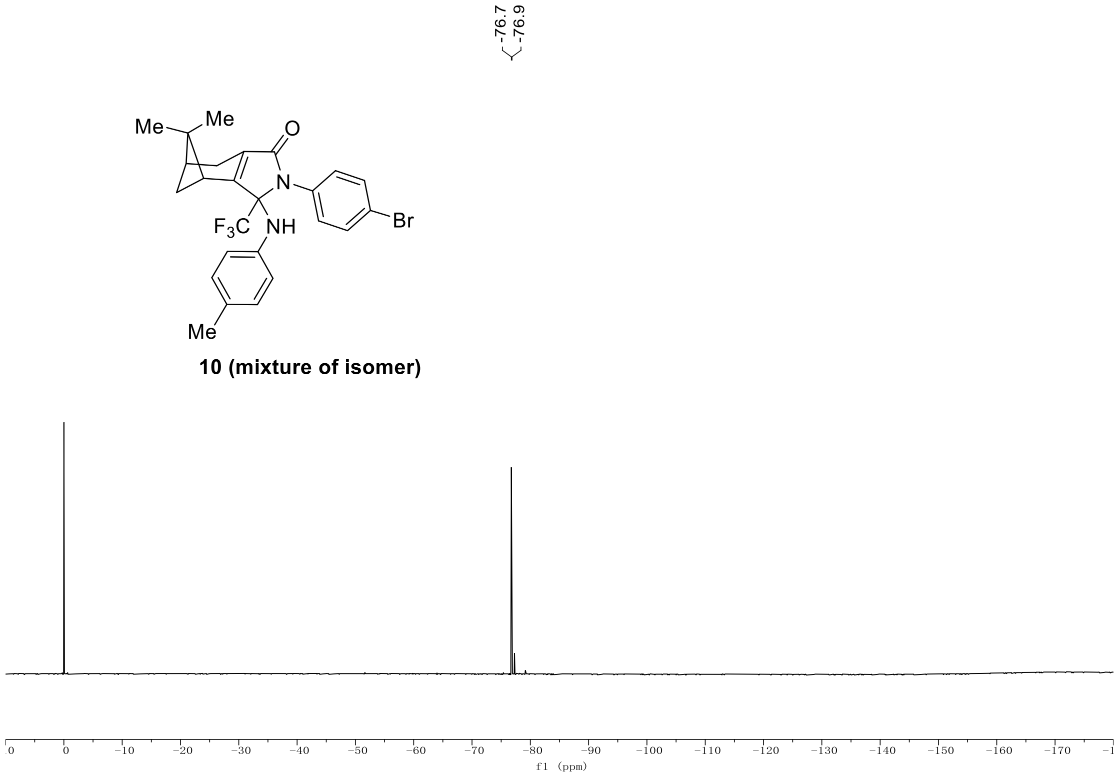

Figure S309. ^{19}F NMR (376 MHz, $CDCl_3$) spectrum of compound 10, related to Scheme 8

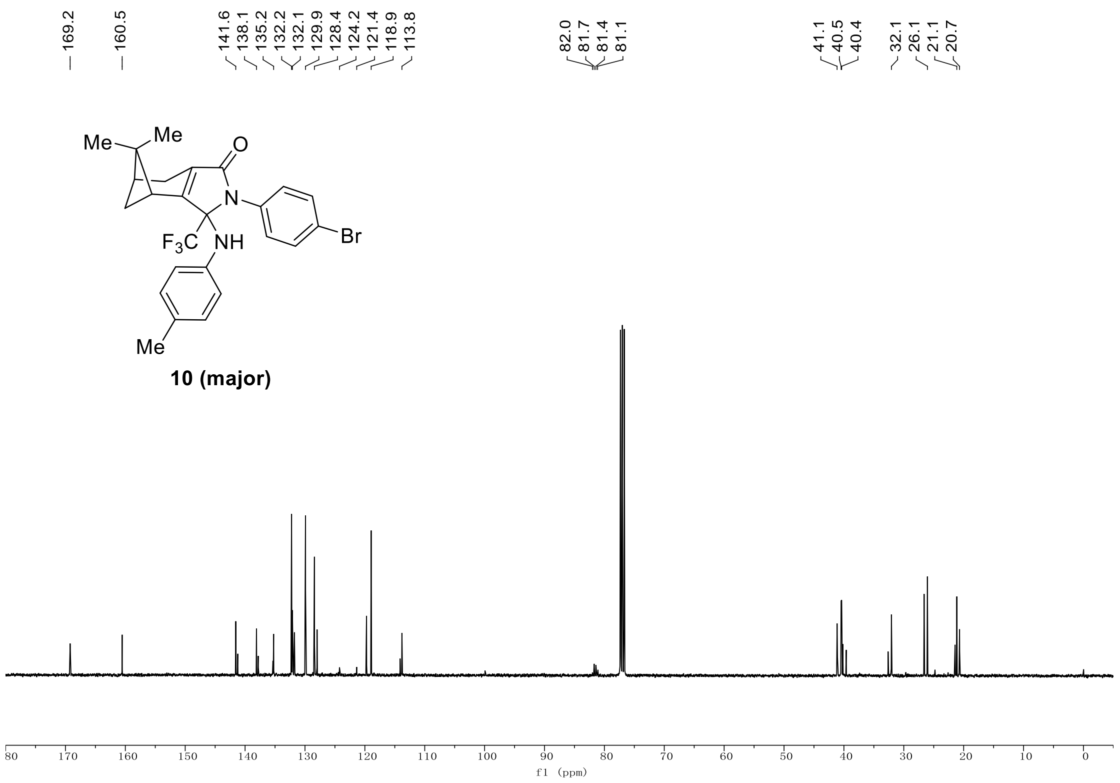

Figure S310. ^{13}C NMR (101 MHz, $CDCl_3$) spectrum of compound 10 (major), related to Scheme 8

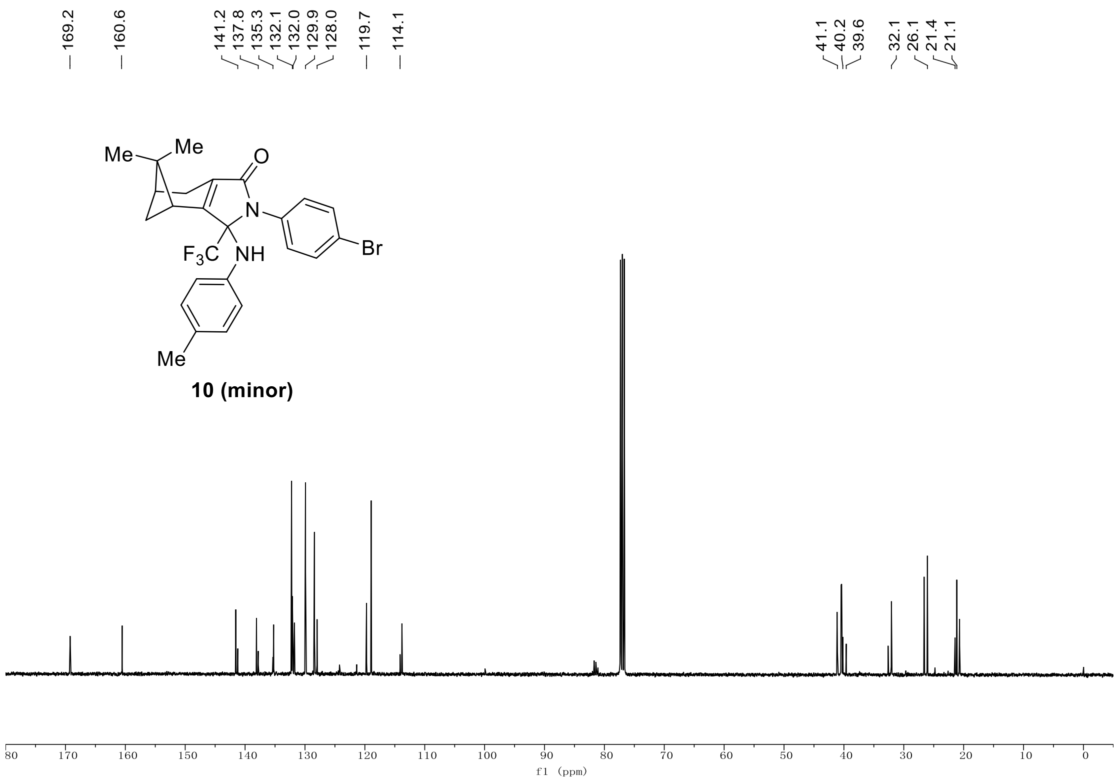

Figure S311. ^{13}**C NMR (101 MHz, CDCl$_3$) spectrum of compound 10 (minor), related to Scheme 8**

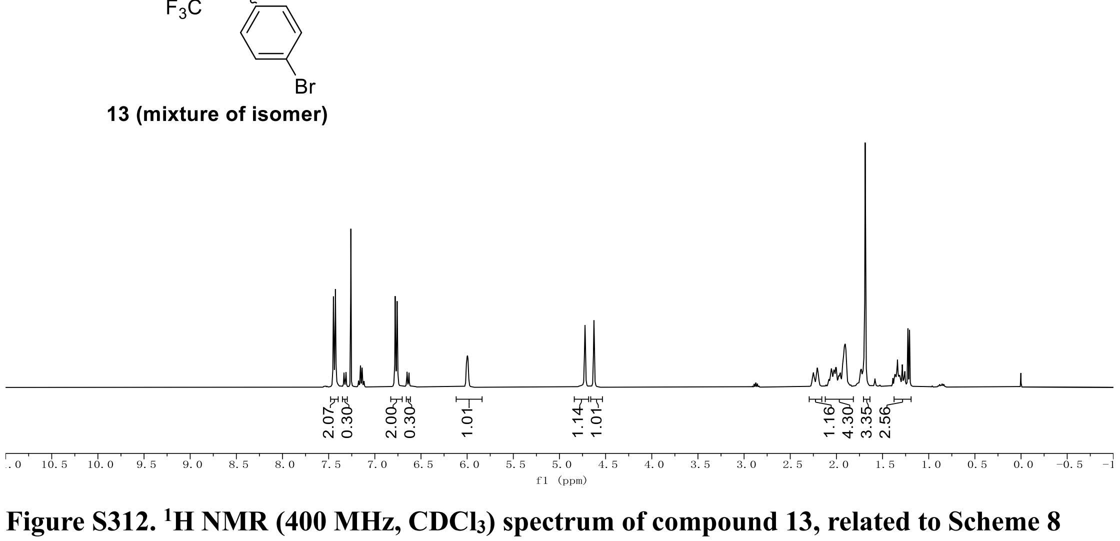

Figure S312. ^{1}**H NMR (400 MHz, CDCl$_3$) spectrum of compound 13, related to Scheme 8**

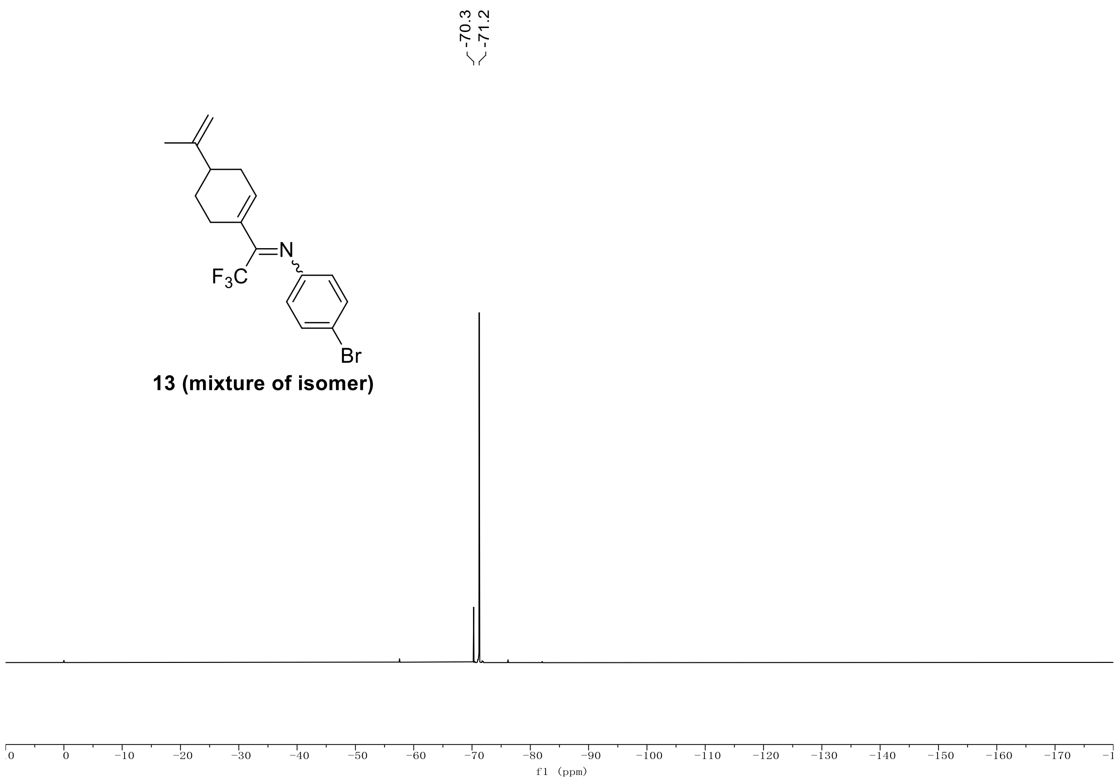

Figure S313. ^{19}**F NMR (376 MHz,** $CDCl_3$**) spectrum of compound 13, related to Scheme 8**

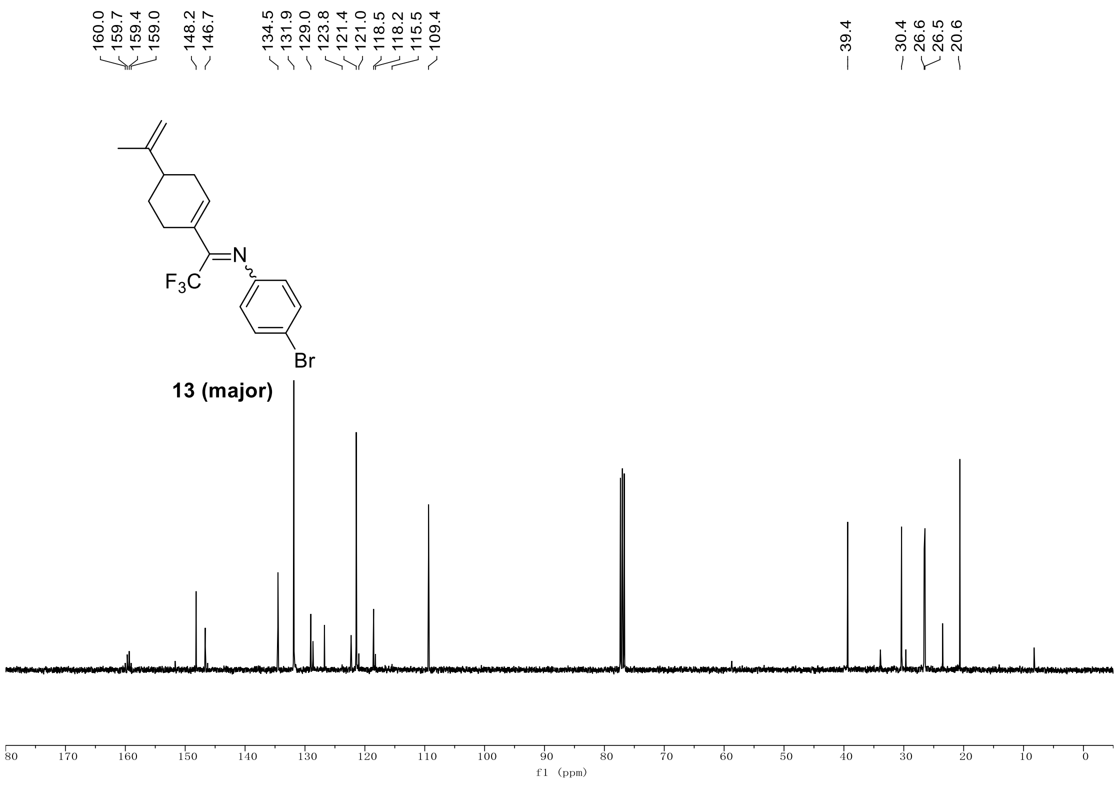

Figure S314. ^{13}**C NMR (101 MHz,** $CDCl_3$**) spectrum of compound 13 (major), related to Scheme 8**

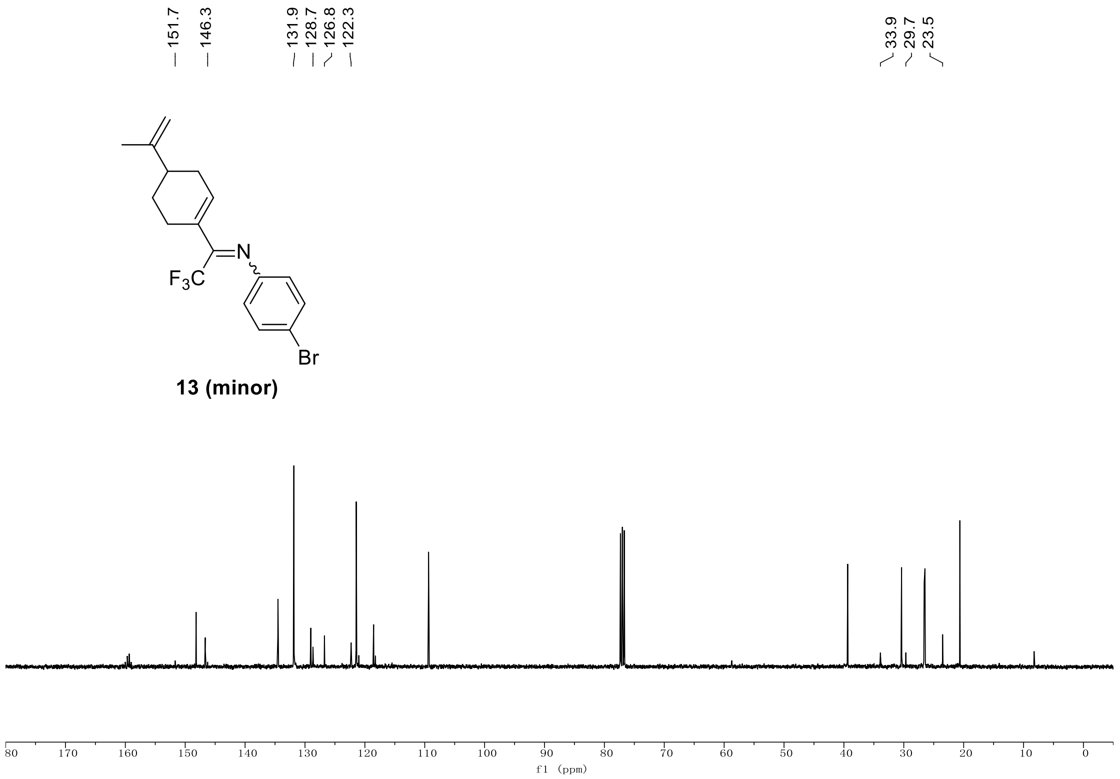

Figure S315. ^{13}C NMR (101 MHz, $CDCl_3$) spectrum of compound 13 (minor), related to Scheme 8

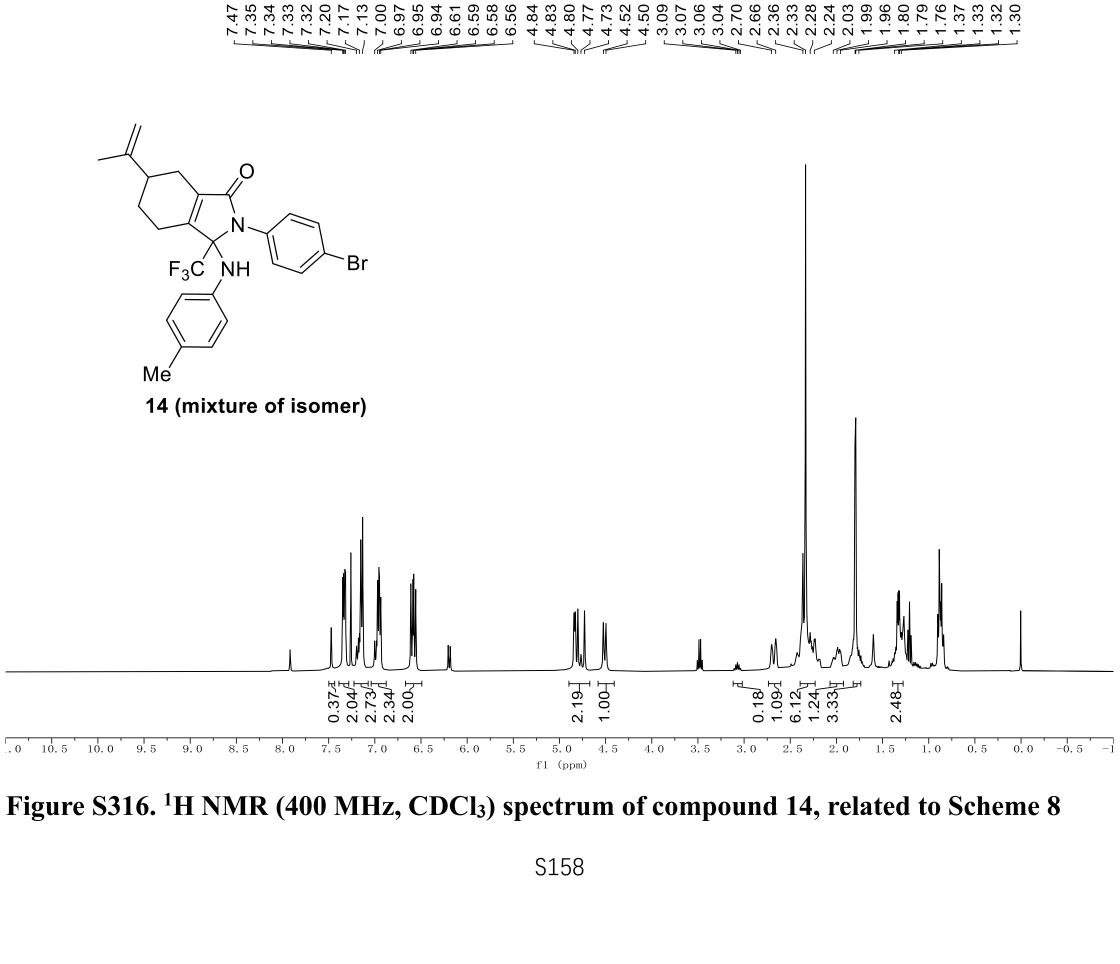

Figure S316. ^{1}H NMR (400 MHz, $CDCl_3$) spectrum of compound 14, related to Scheme 8

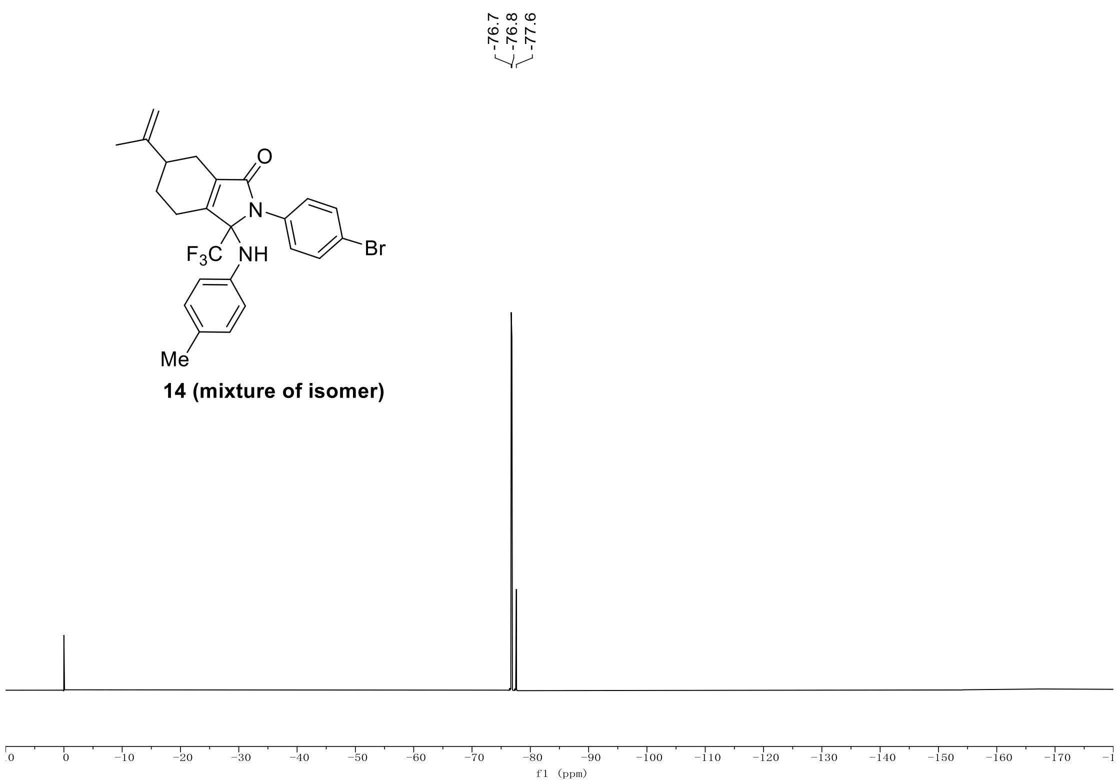

Figure S317. ^{19}F NMR (376 MHz, CDCl$_3$) spectrum of compound 14, related to Scheme 8

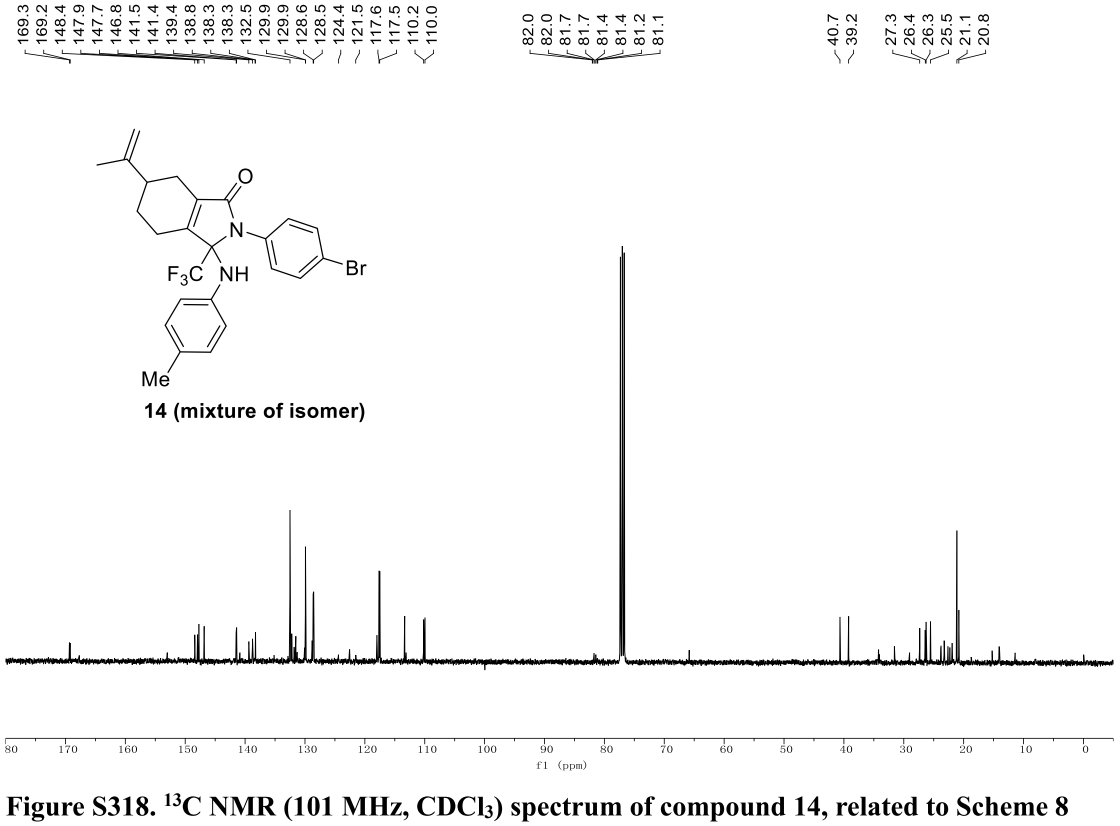

Figure S318. ^{13}C NMR (101 MHz, CDCl$_3$) spectrum of compound 14, related to Scheme 8

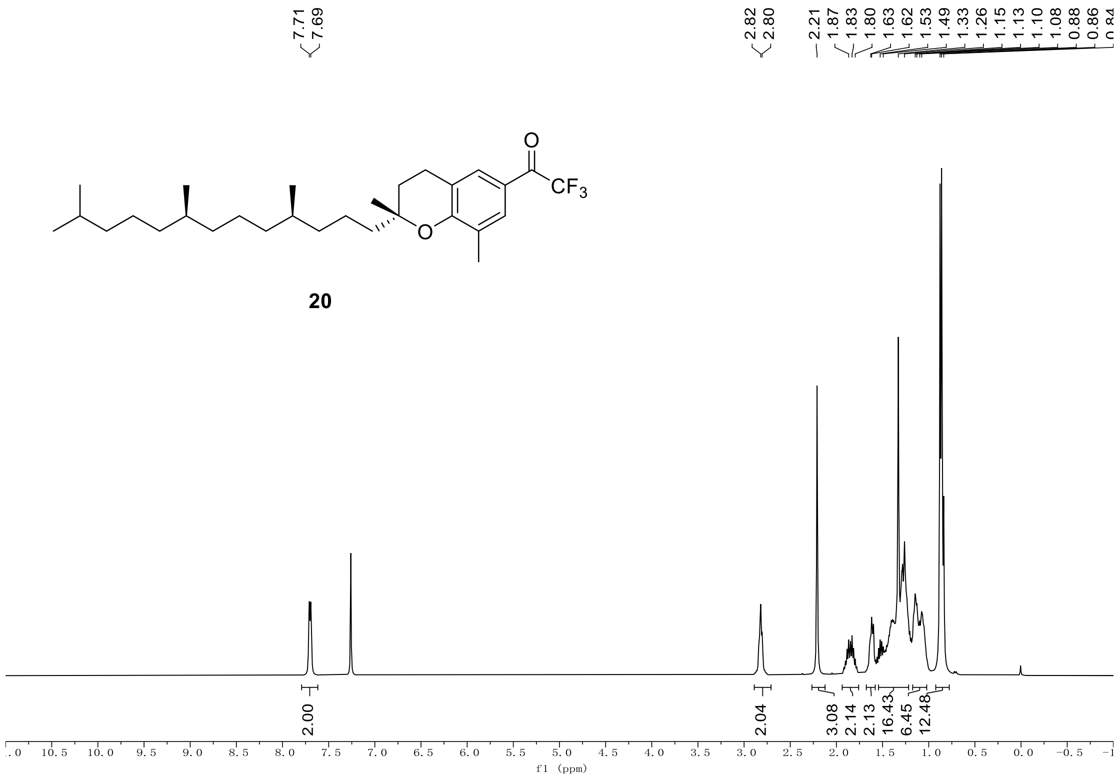

Figure S319. ^1H NMR (400 MHz, $CDCl_3$) spectrum of compound 20, related to Scheme 8

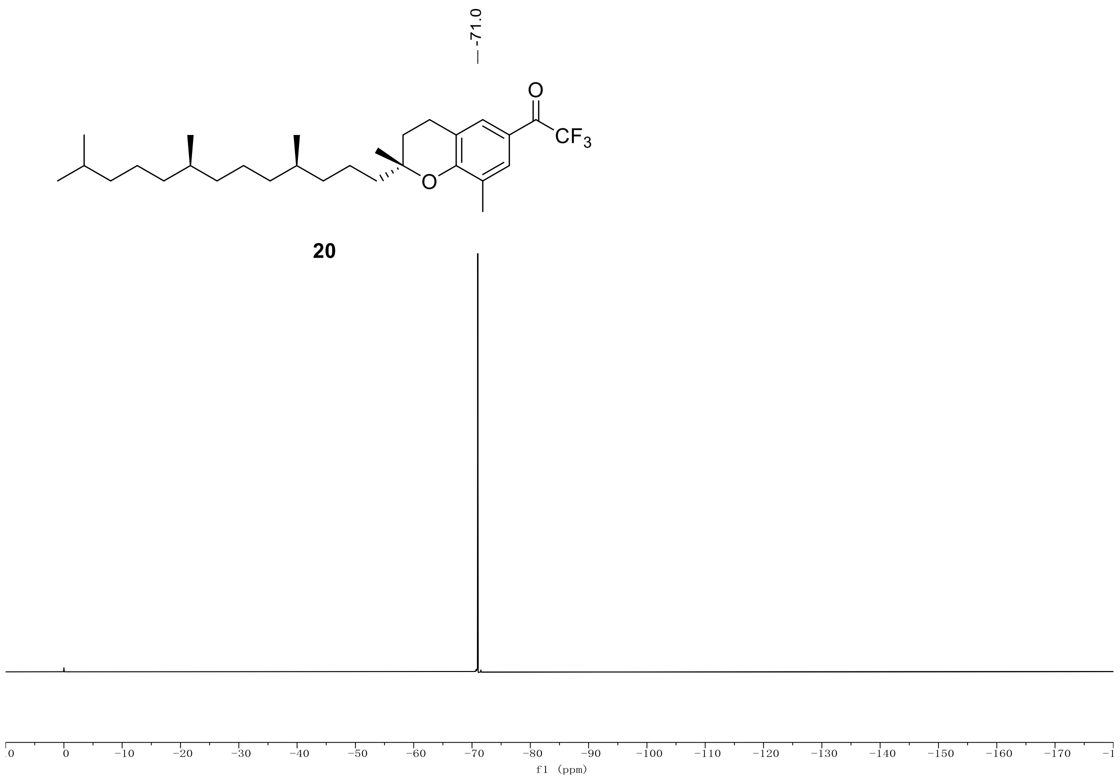

Figure S320. ^{19}F NMR (376 MHz, $CDCl_3$) spectrum of compound 20, related to Scheme 8

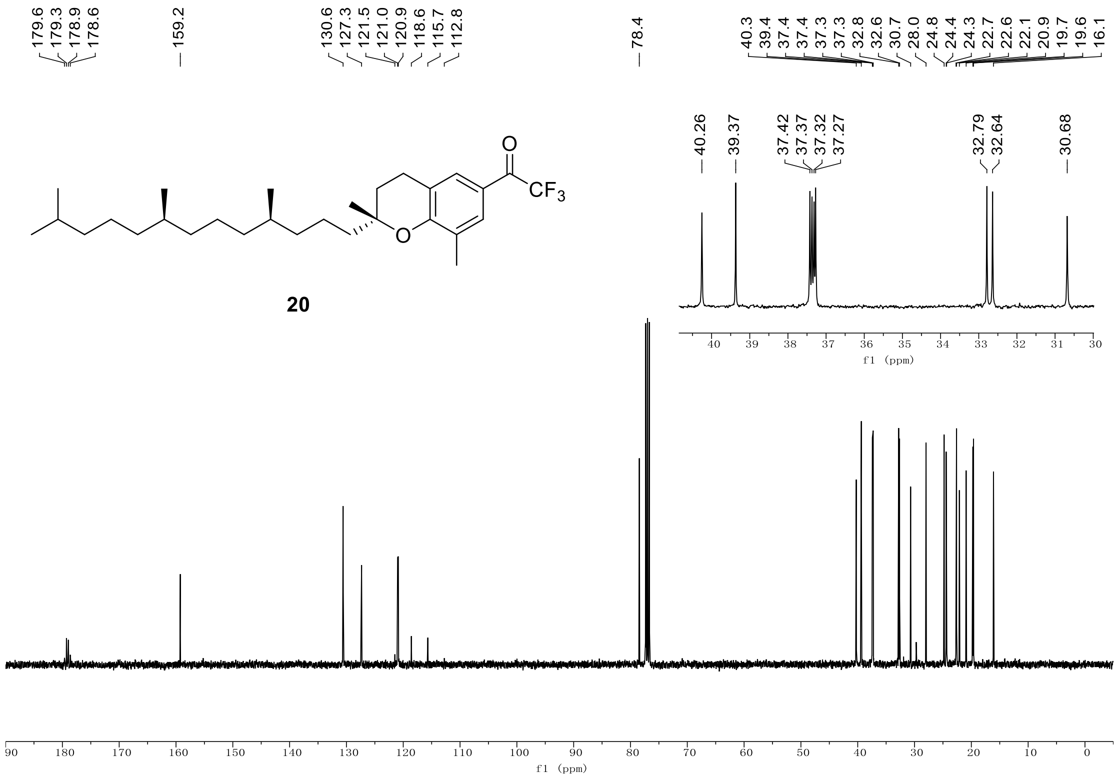

Figure S321. ^{13}C NMR (101 MHz, $CDCl_3$) spectrum of compound 20, related to Scheme 8

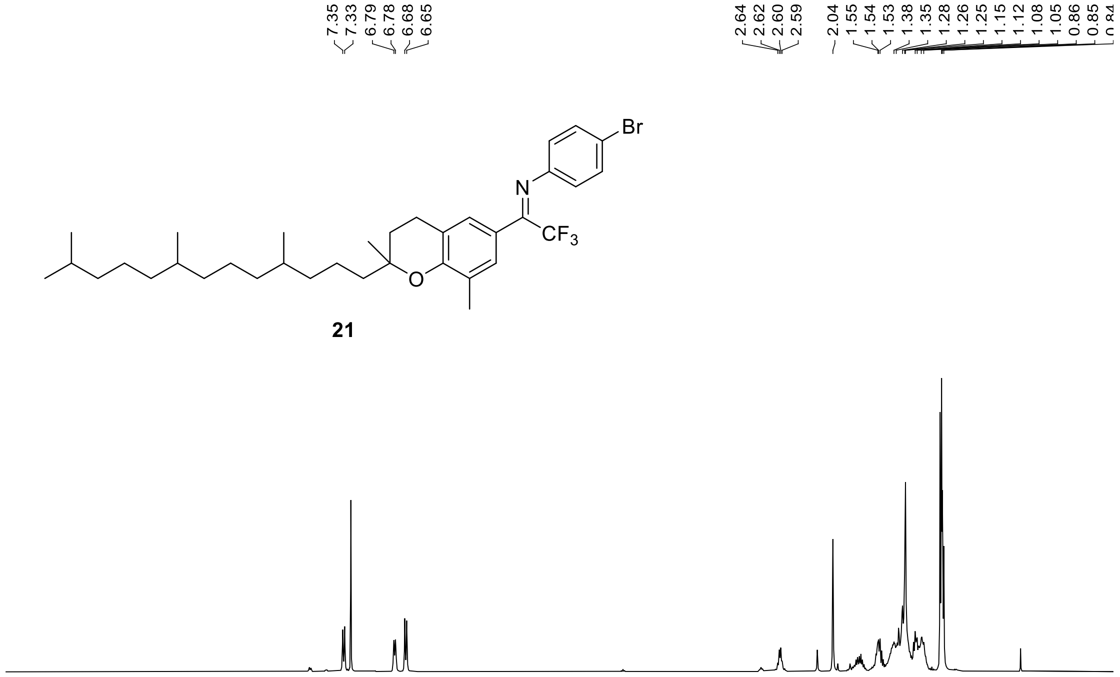

Figure S322. ^{1}H NMR (400 MHz, $CDCl_3$) spectrum of compound 21, related to Scheme 8

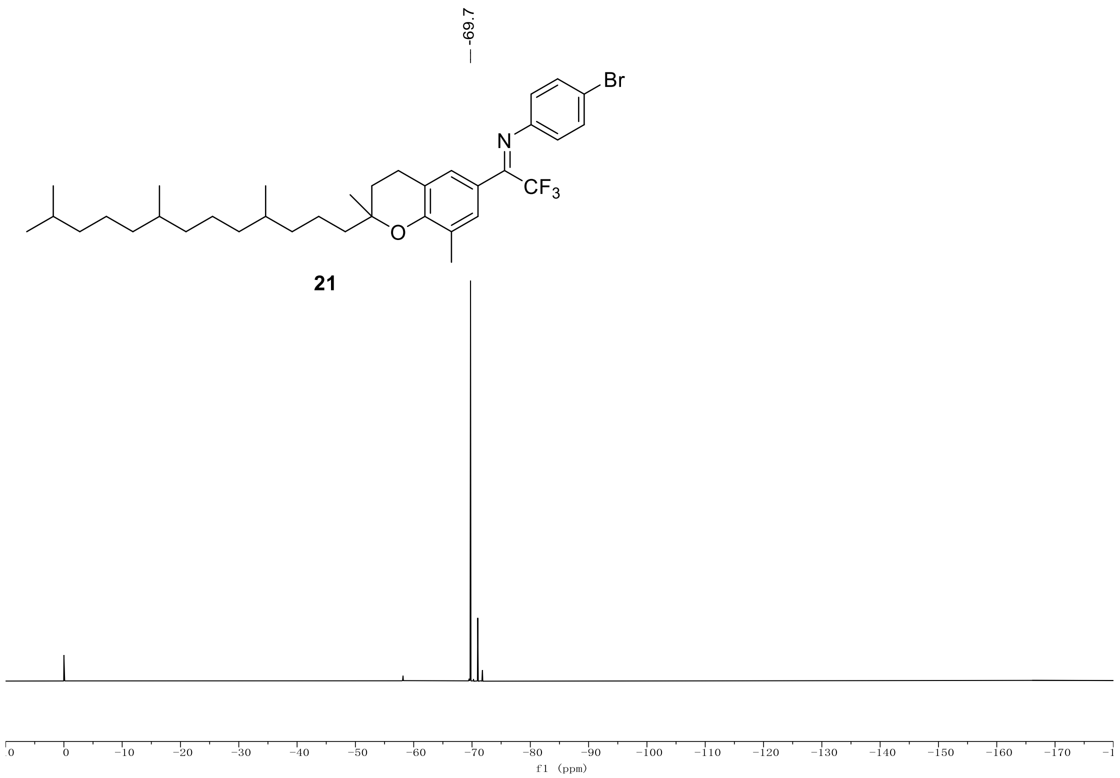

Figure S323. ^{19}F NMR (376 MHz, CDCl$_3$) spectrum of compound 21, related to Scheme 8

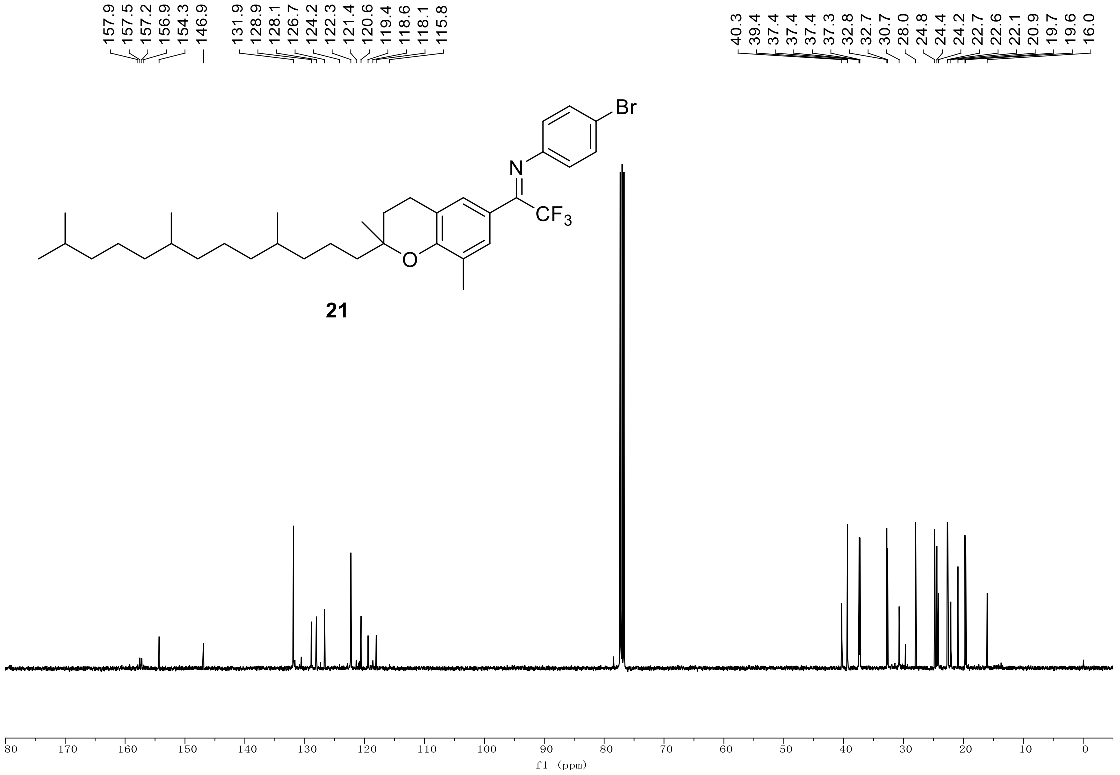

Figure S324. ^{13}C NMR (101 MHz, CDCl$_3$) spectrum of compound 21, related to Scheme 8

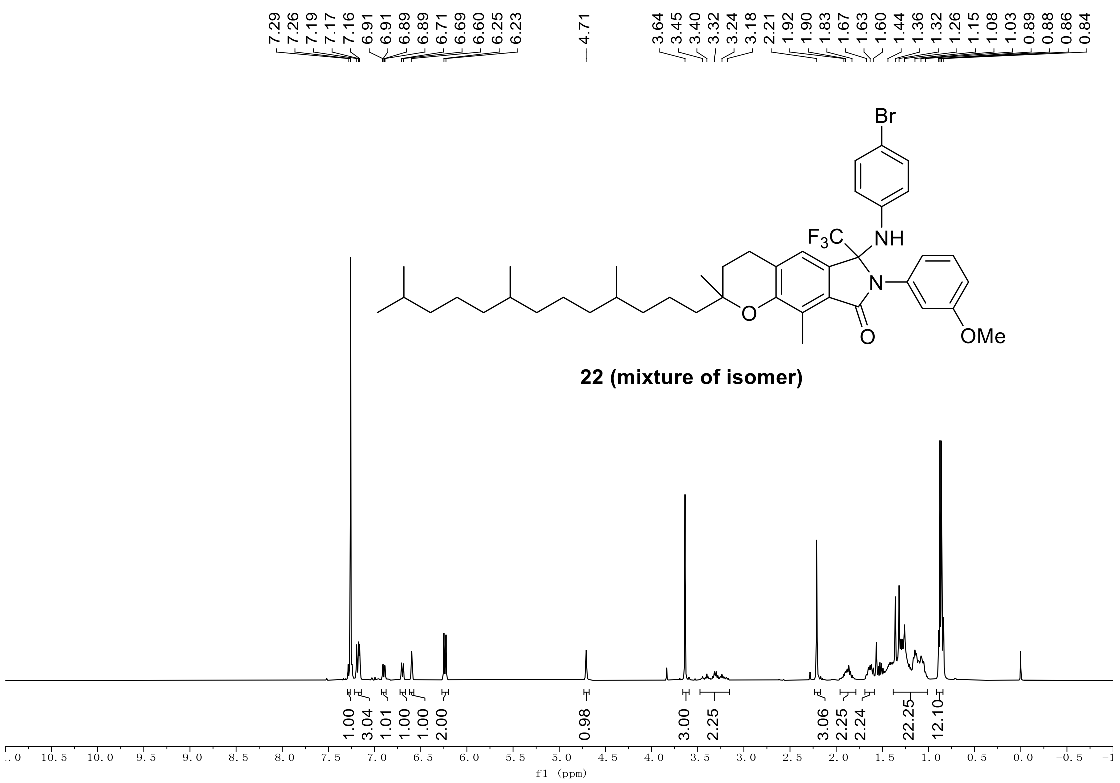

Figure S325. ^{1}H NMR (400 MHz, $CDCl_3$) spectrum of compound 22, related to Scheme 8

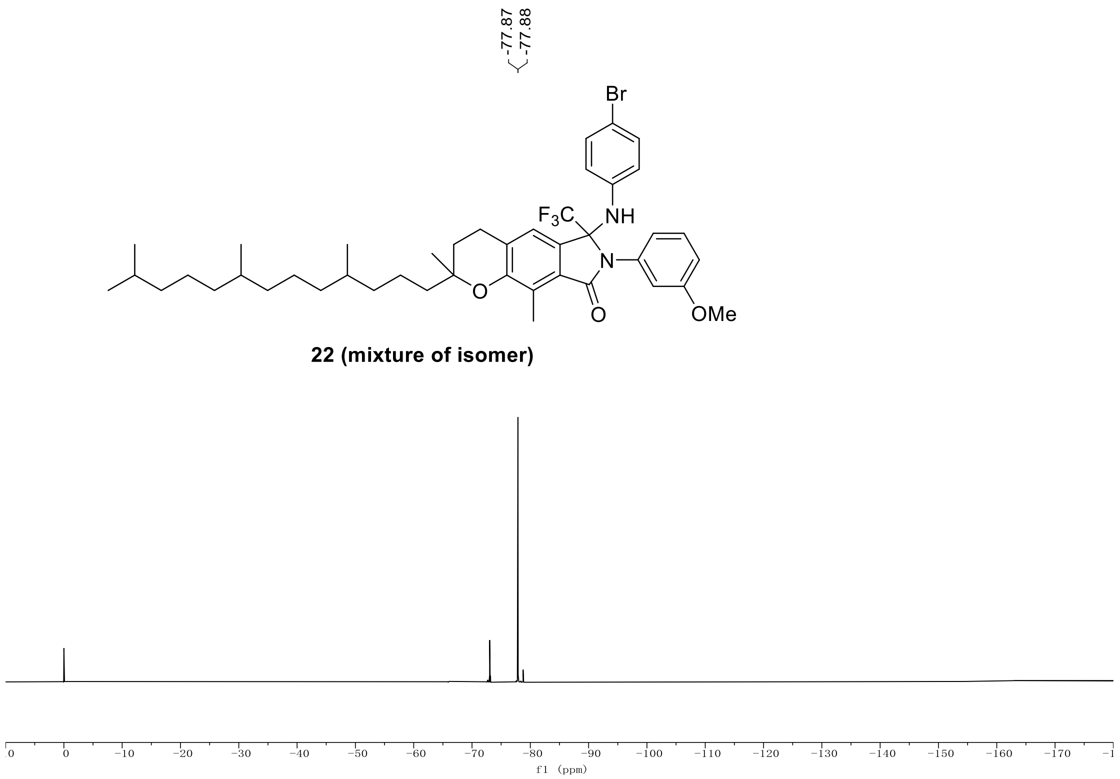

Figure S326. ^{19}F NMR (376 MHz, $CDCl_3$) spectrum of compound 22, related to Scheme 8

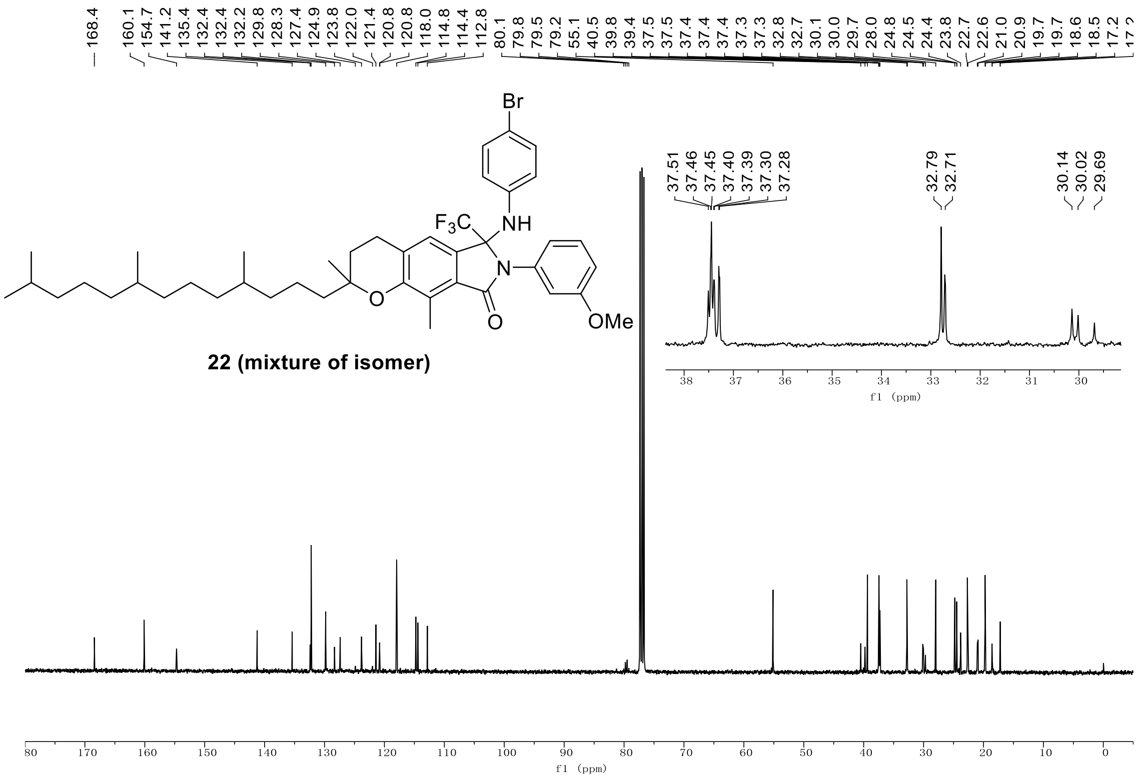

Figure S327. ^{13}C NMR (101 MHz, $CDCl_3$) spectrum of compound 22, related to Scheme 8

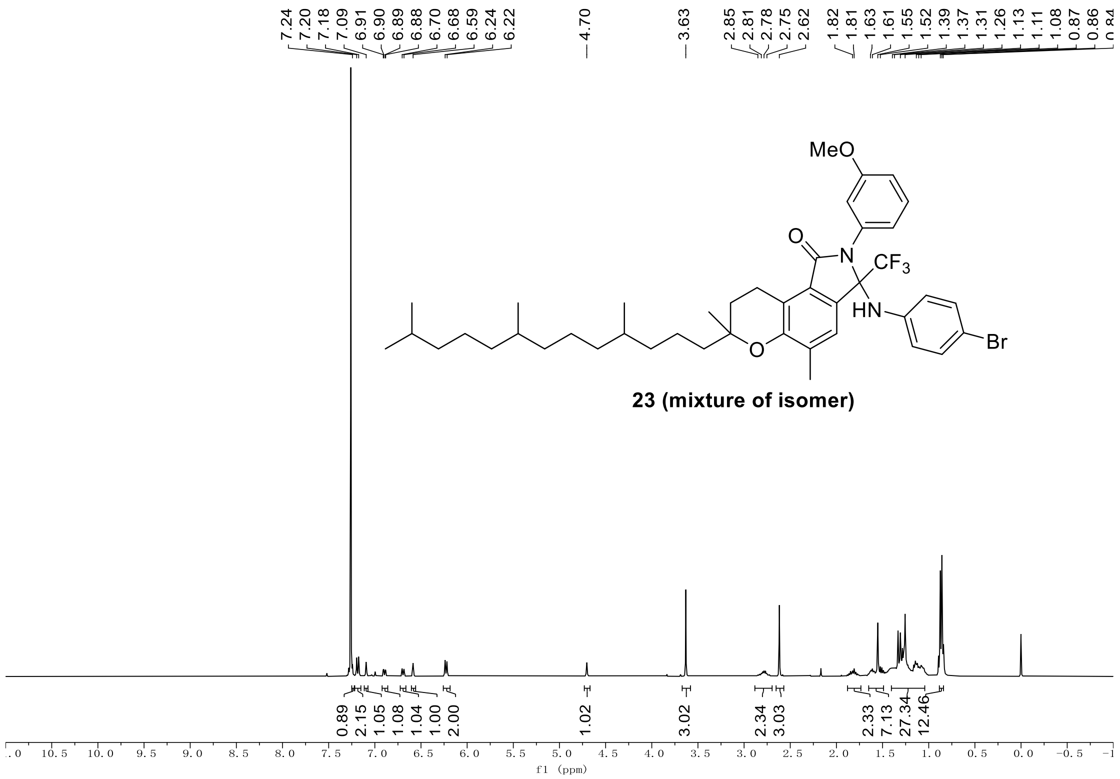

Figure S328. ^{1}H NMR (400 MHz, $CDCl_3$) spectrum of compound 23, related to Scheme 8

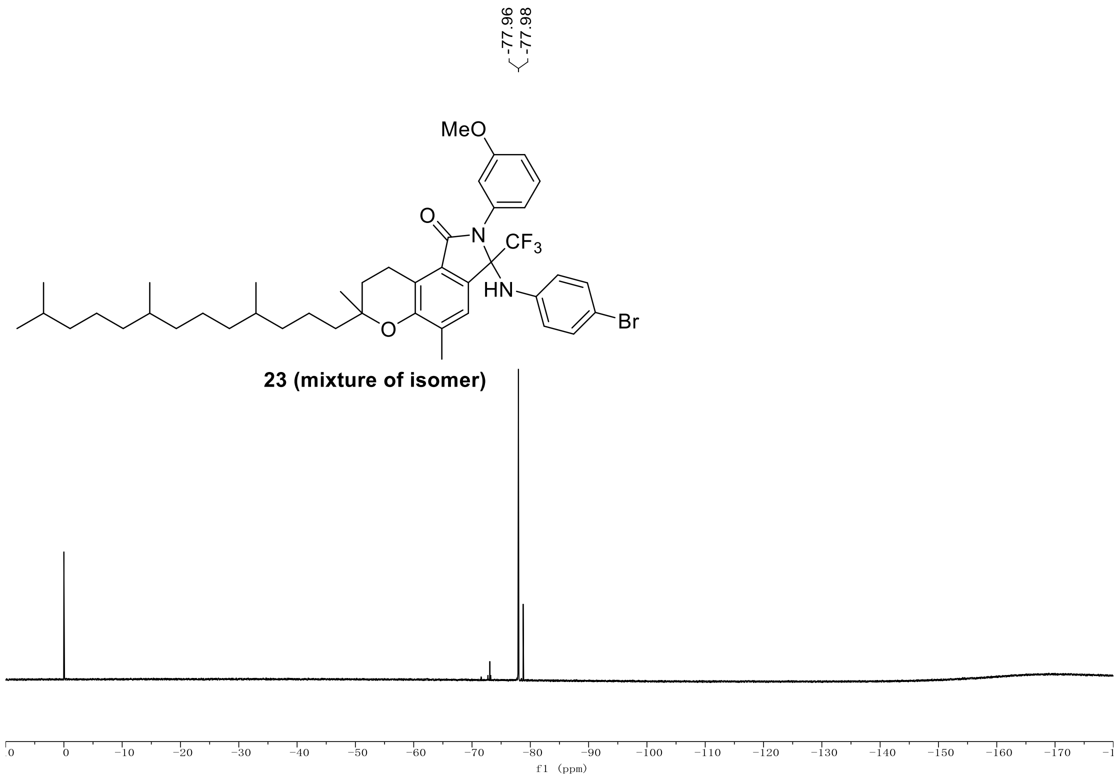

Figure S329. ^{19}F NMR (376 MHz, $CDCl_3$) spectrum of compound 23, related to Scheme 8

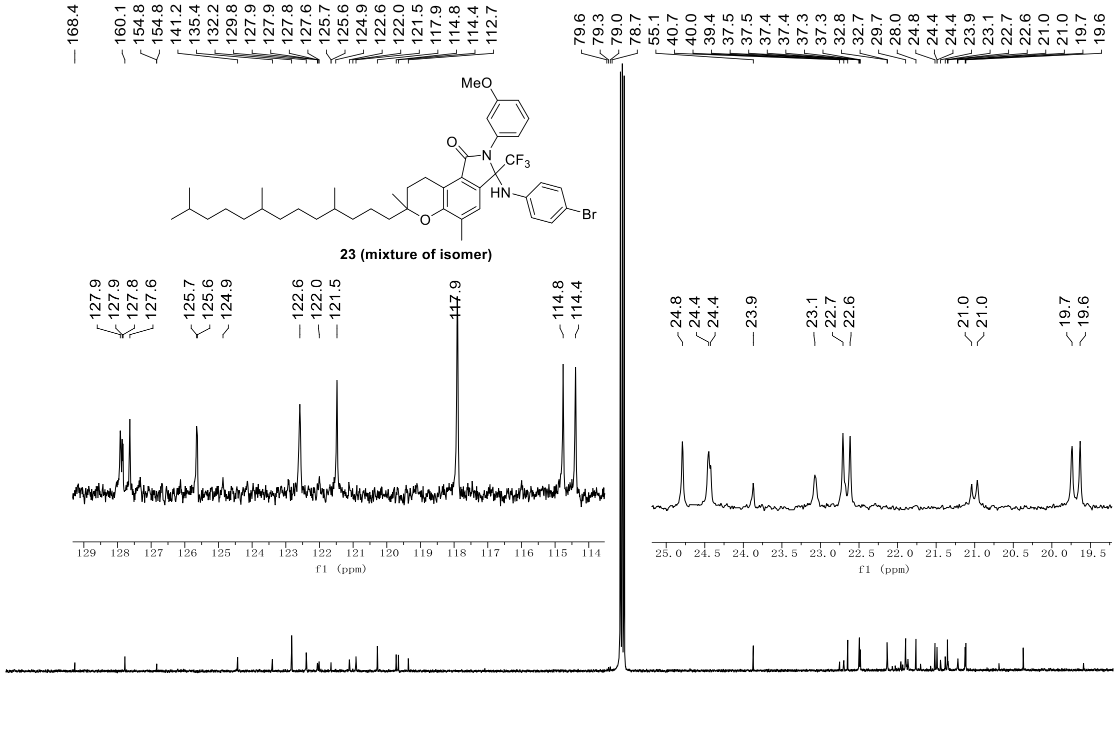

Figure S330. ^{13}C NMR (101 MHz, $CDCl_3$) spectrum of compound 23, related to Scheme 8

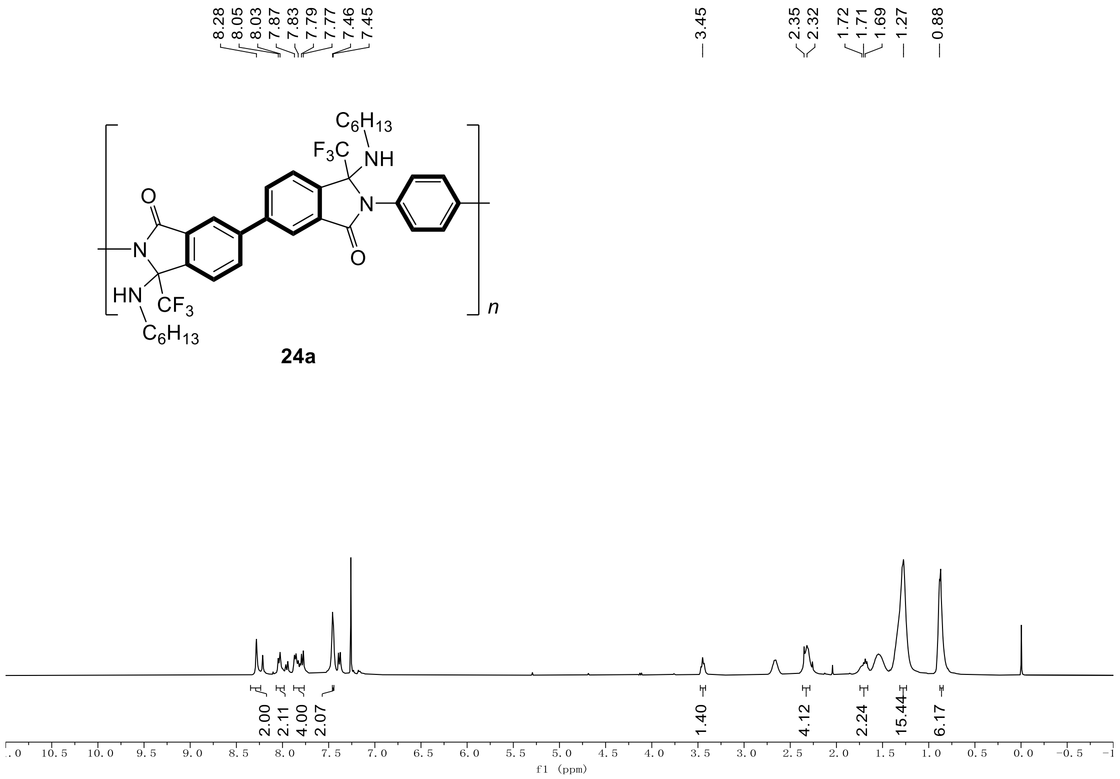

Figure S331. ^{1}H NMR (400 MHz, $CDCl_3$) spectrum of compound 24a, related to Figure 3

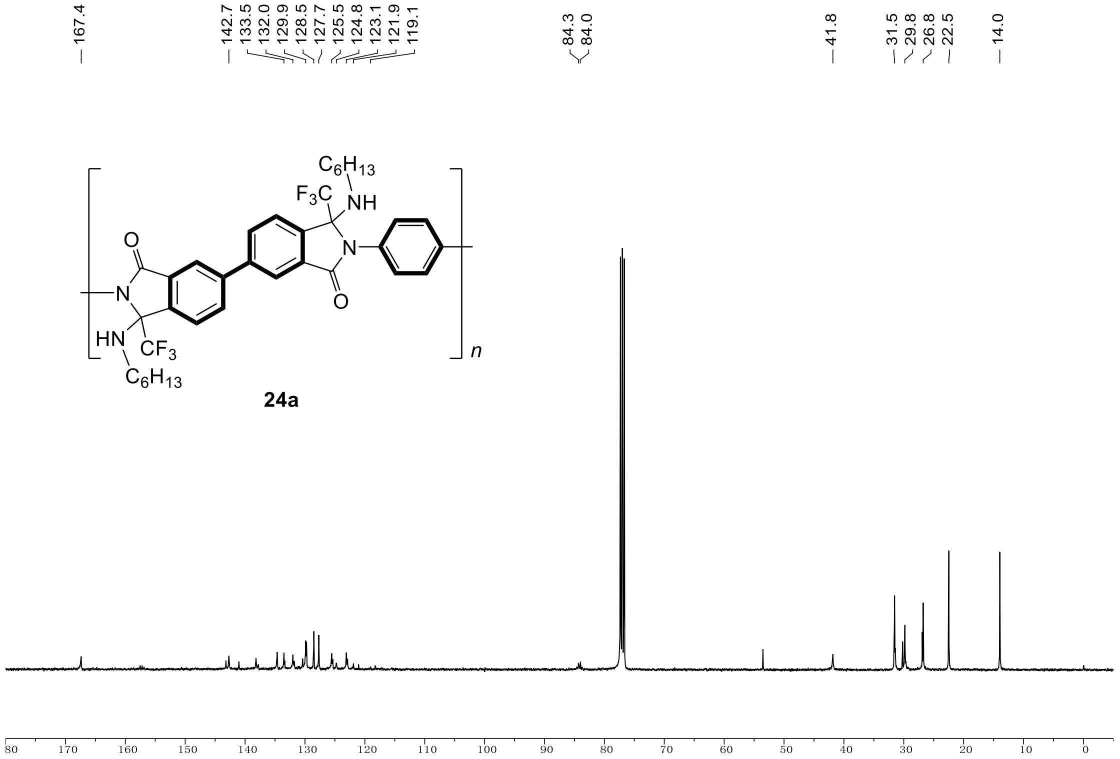

Figure S332. ^{13}C NMR (101 MHz, $CDCl_3$) spectrum of compound 24a, related to Figure 3

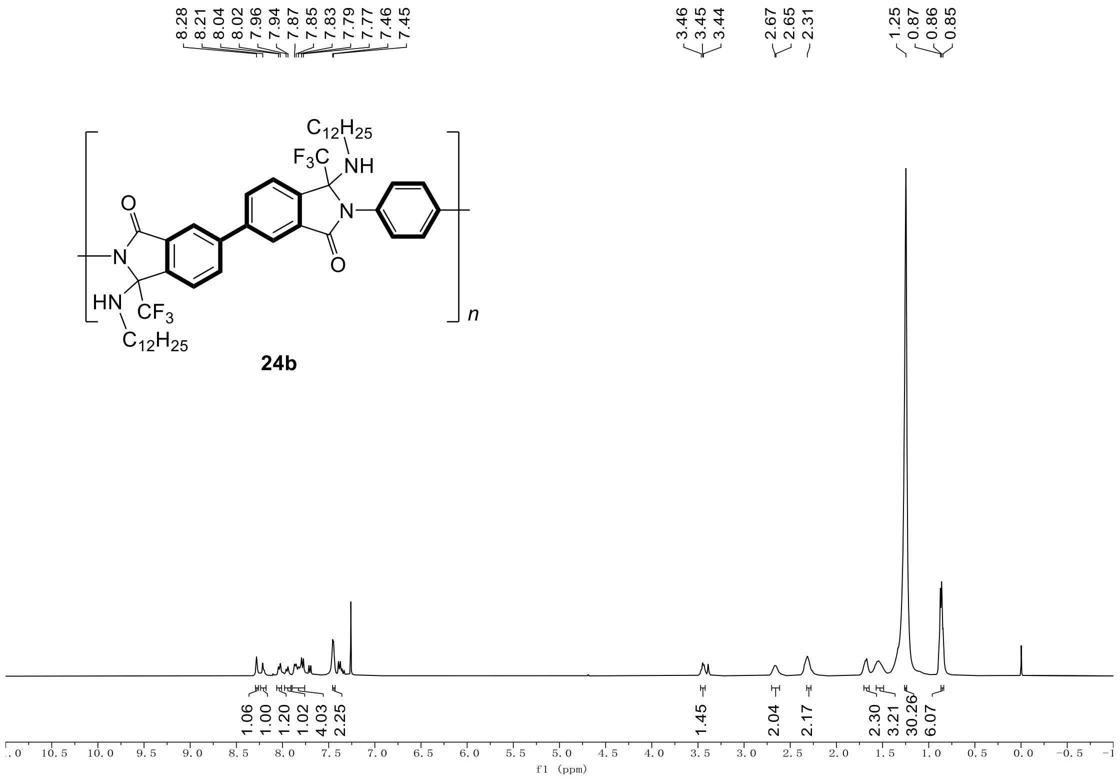

Figure S333. ^{1}H NMR (400 MHz, $CDCl_3$) spectrum of compound 24b, related to Figure 3

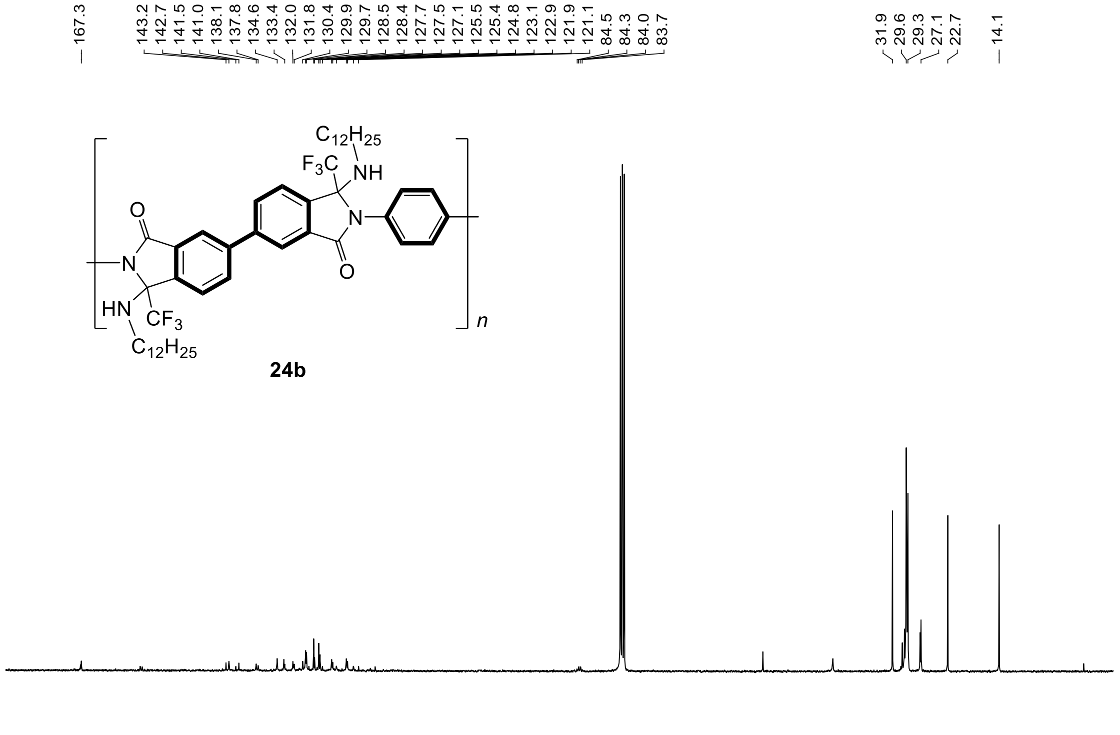

Figure S334. ^{13}C NMR (101 MHz, $CDCl_3$) spectrum of compound 24b, related to Figure 3

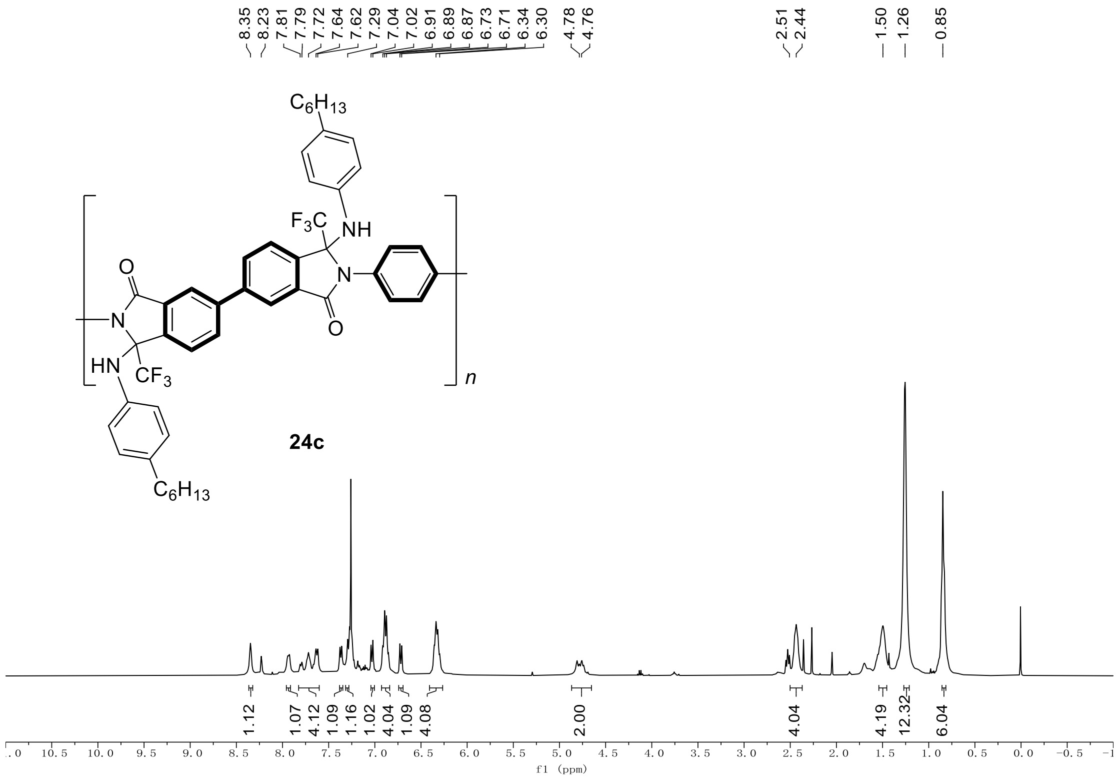

Figure S335. ^{1}H NMR (400 MHz, $CDCl_3$) spectrum of compound 24c, related to Figure 3

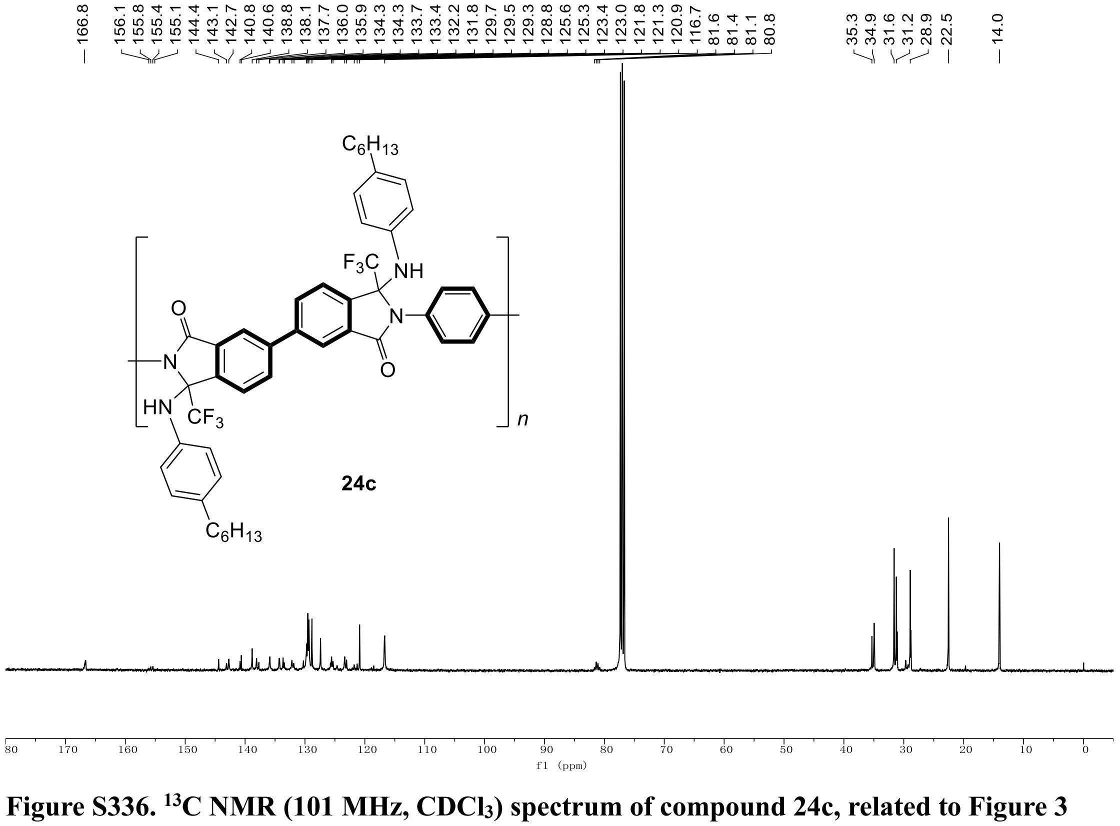

Figure S336. ^{13}C NMR (101 MHz, $CDCl_3$) spectrum of compound 24c, related to Figure 3

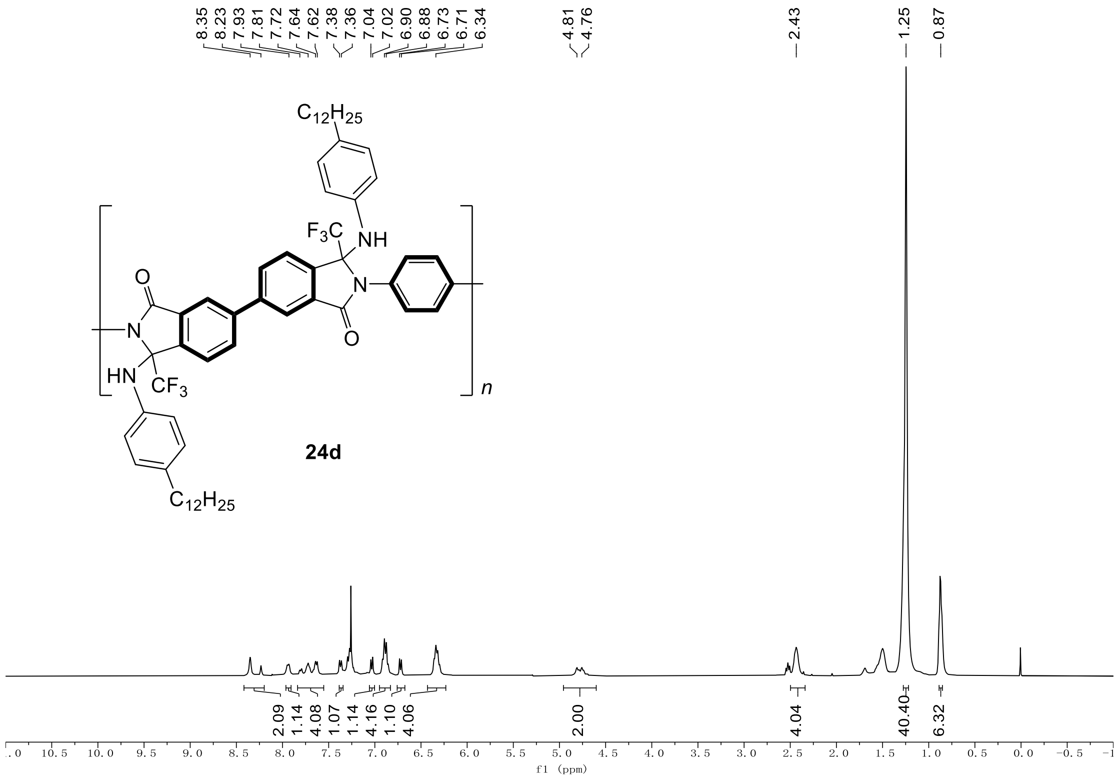

Figure S337. ^{1}H NMR (400 MHz, $CDCl_3$) spectrum of compound 24d, related to Figure 3

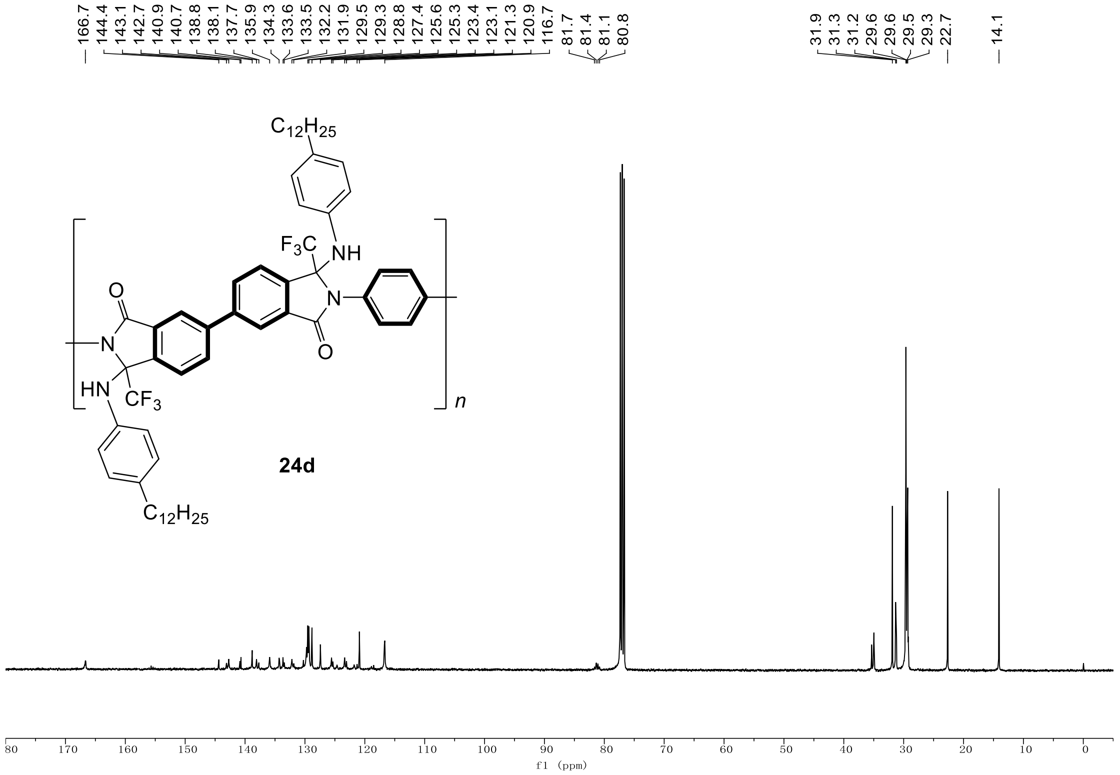

Figure S338. ^{13}C NMR (101 MHz, $CDCl_3$) spectrum of compound 24d, related to Figure 3

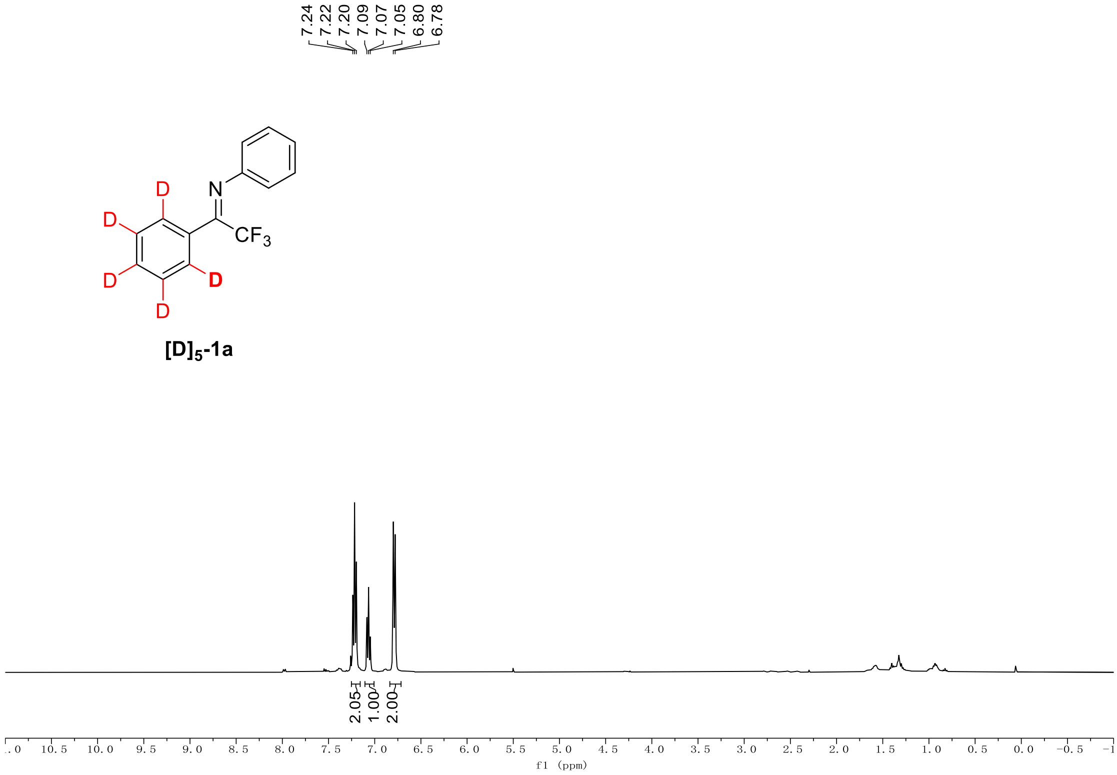

Figure S339. H[1] NMR spectra copy of [D]$_5$-1a, related to Scheme 4

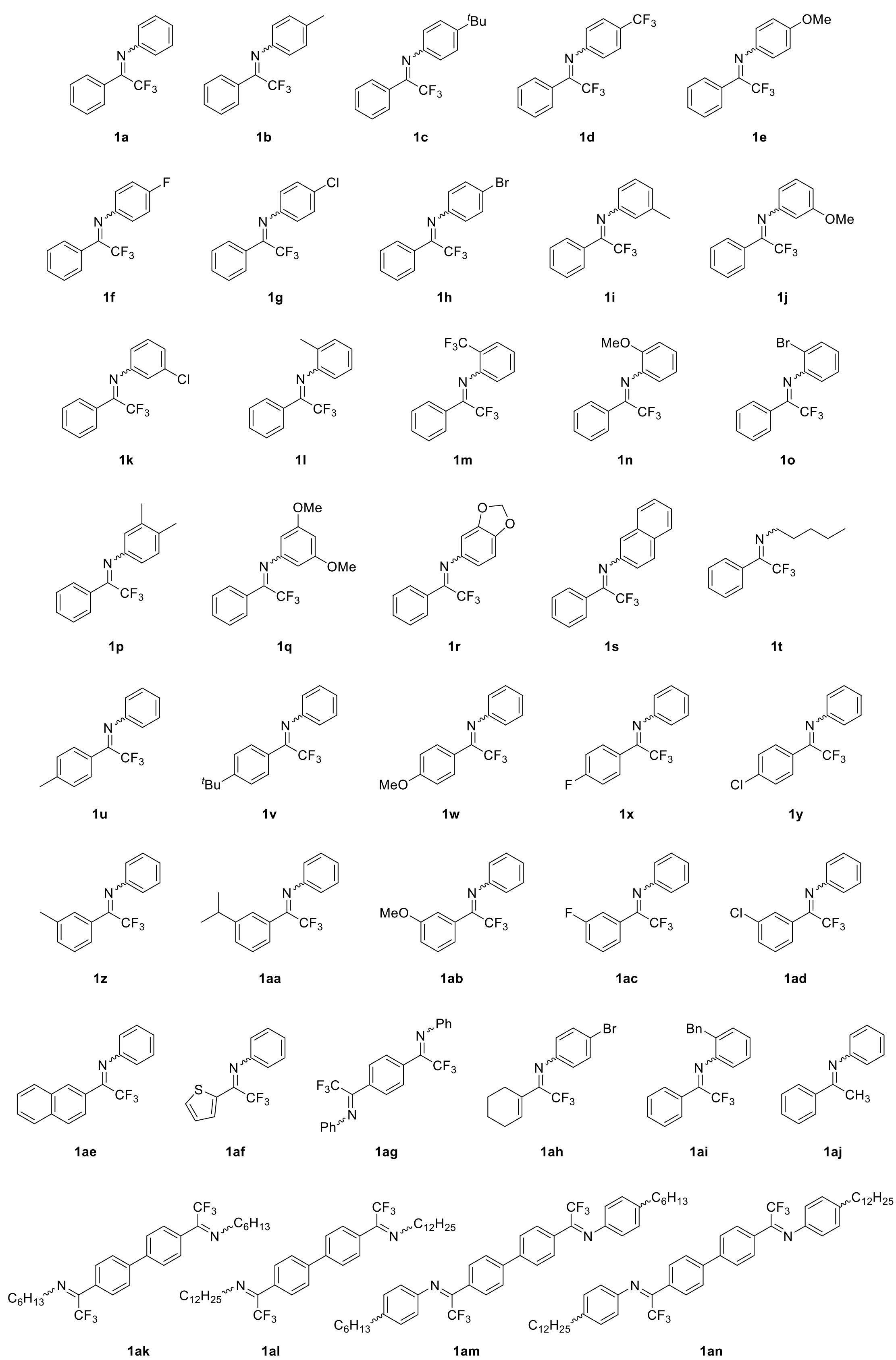

Figure S340. List of the derivatives 1, related to Table 1 and Scheme 2

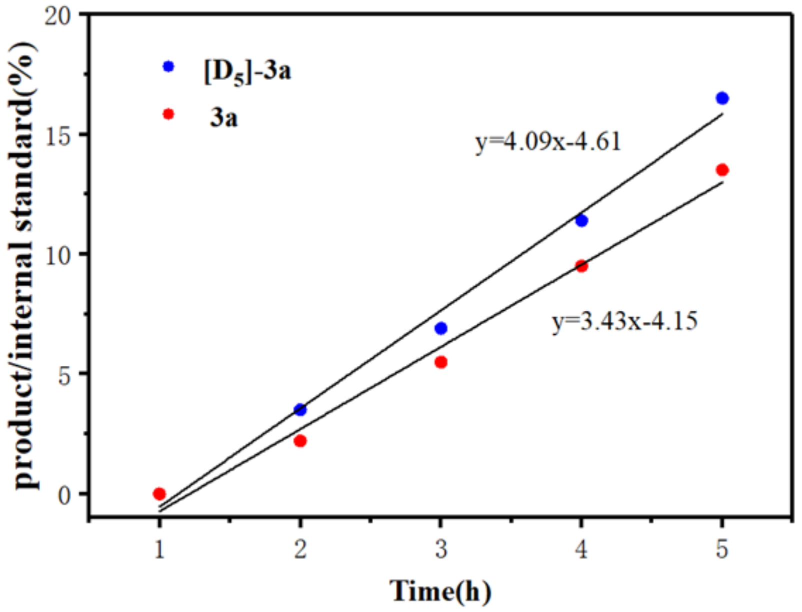

Figure S341. Parallel reactions Kinetic Isotope Effect (KIE) measurements, related to Scheme 4

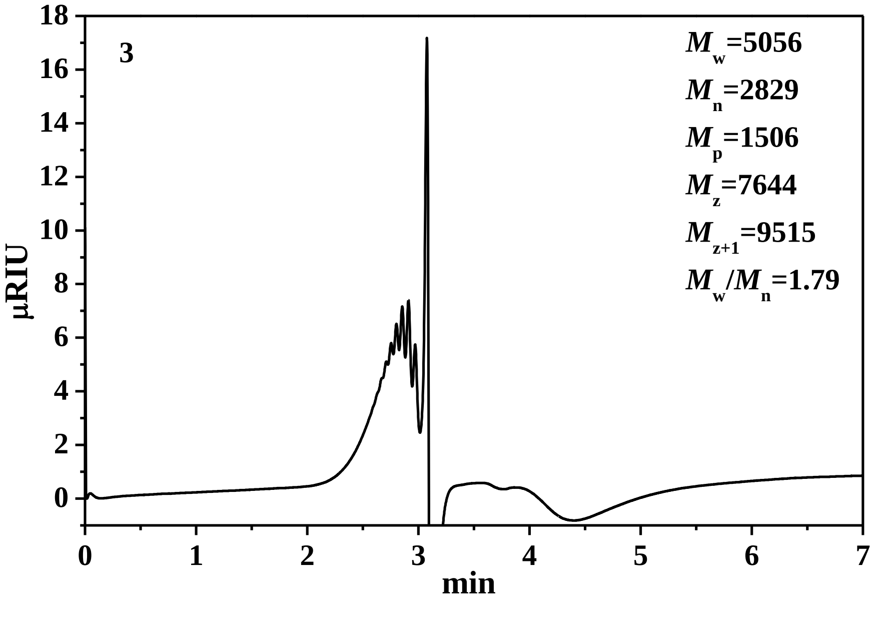

Figure S342. Molecular weight (Mn) and molecular weight distribution (Mw/Mn) values of 24a, related to Figure 3

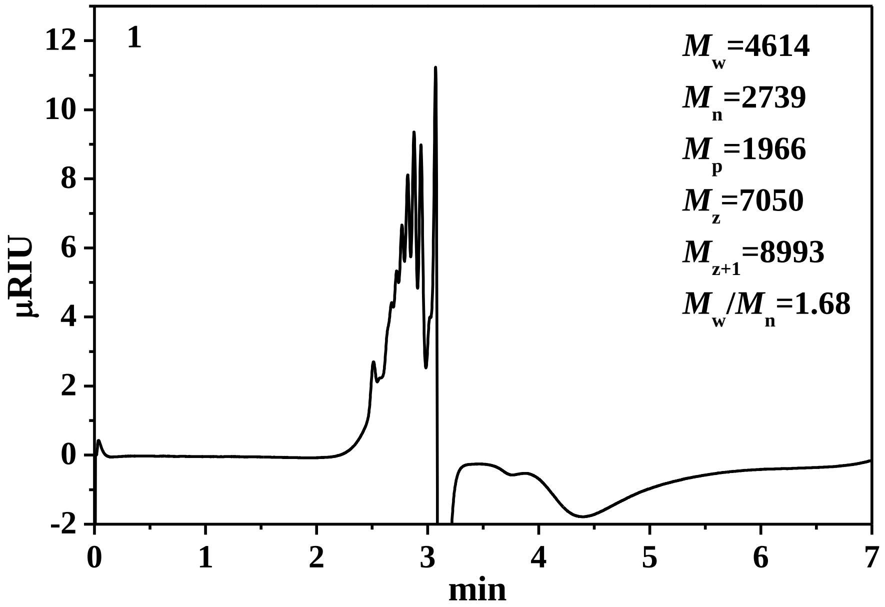

Figure S343. Molecular weight (Mn) and molecular weight distribution (Mw/Mn) values of 24b, related to Figure 3

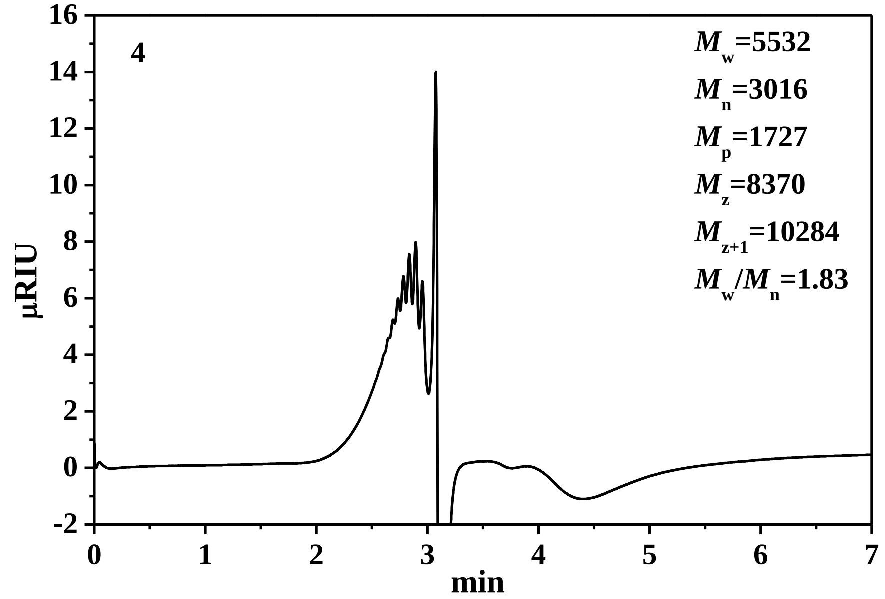

Figure S344. Molecular weight (Mn) and molecular weight distribution (Mw/Mn) values of 24c, related to Figure 3

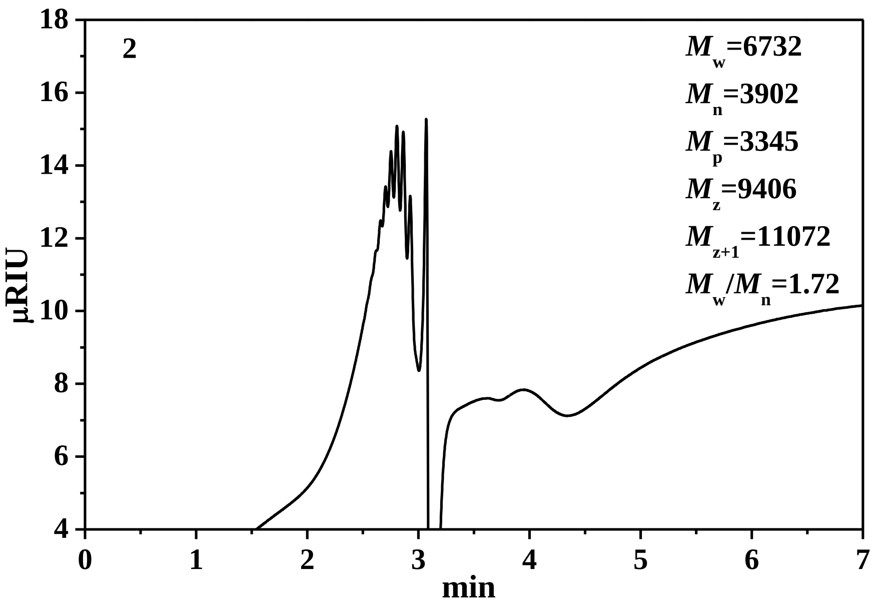

Figure S345. Molecular weight (Mn) and molecular weight distribution (Mw/Mn) values of 24d, related to Figure 3

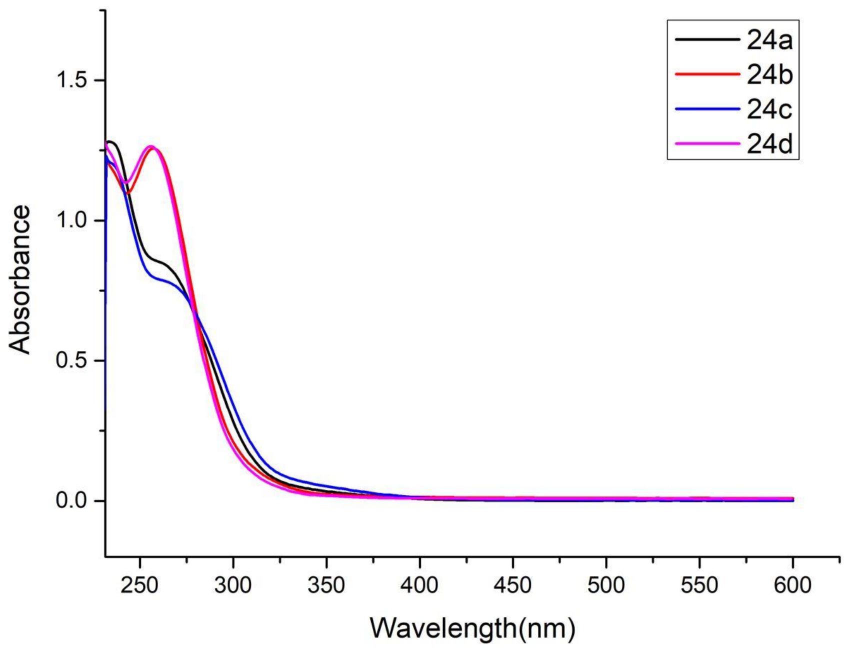

Figure S346. Absorption spectra (UV) of 24a-d ([C] = 1×10^{-5} M, related to Figure 3

Supplementary Schemes

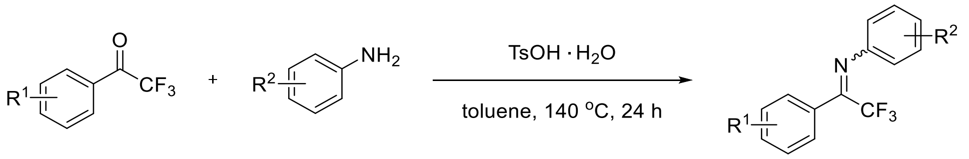

1

Scheme S1. General procedure for the synthesis of 1a-1u, 1w-1y, 1af, 1ai and 1aj, related to Table 1 and Scheme 2

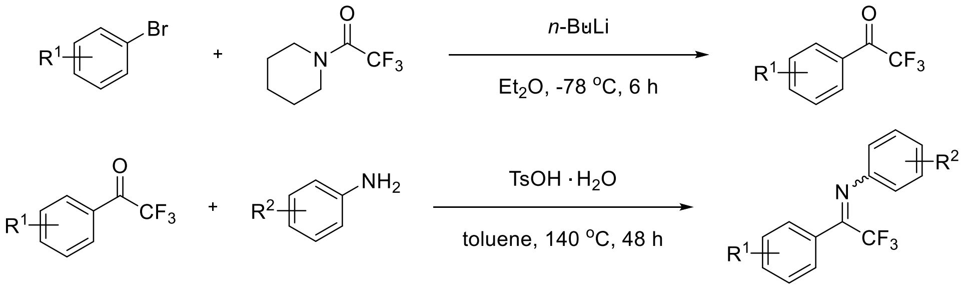

1

Scheme S2. General procedure for the synthesis of 1aa and 1ae, related to Scheme 2

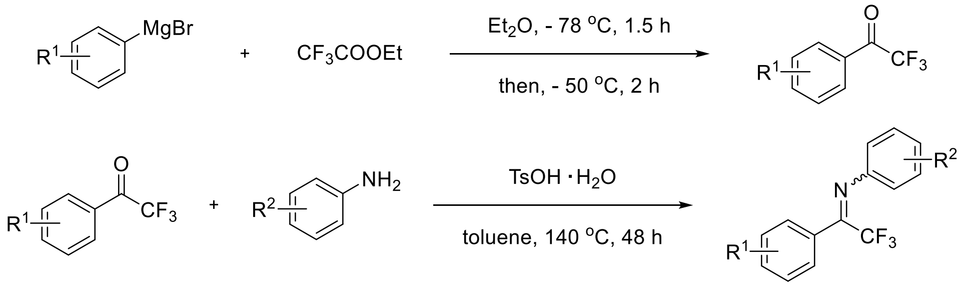

1

Scheme S3. General procedure for the synthesis of 1v, 1z, 1ab-1ad, related to Scheme 2

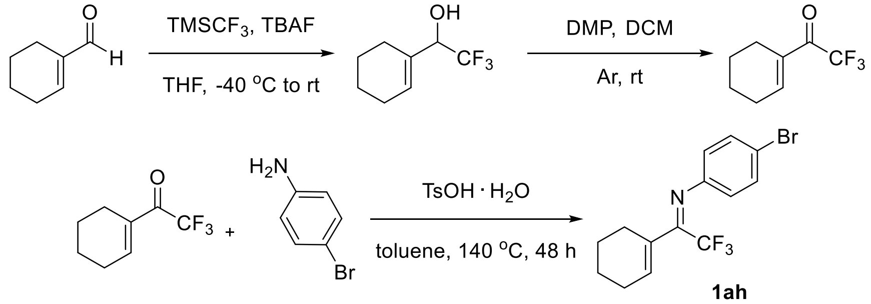

1ah

Scheme S4. Procedure for the synthesis of 1ah, related to Scheme 2

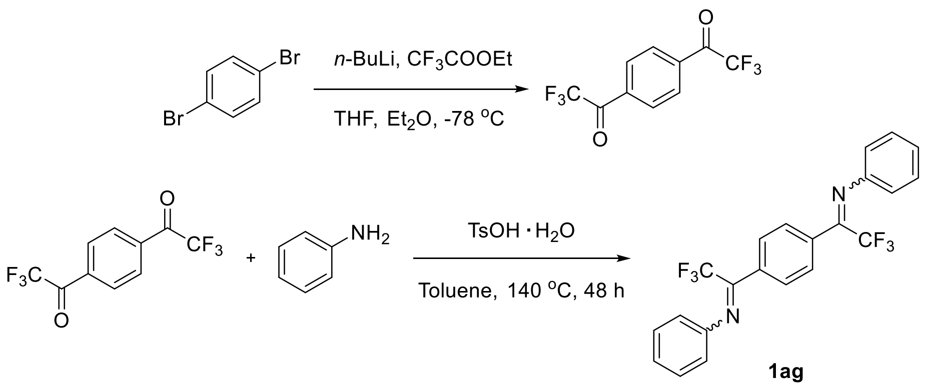

Scheme S5. Procedure for the synthesis of 1ag, related to Scheme 2

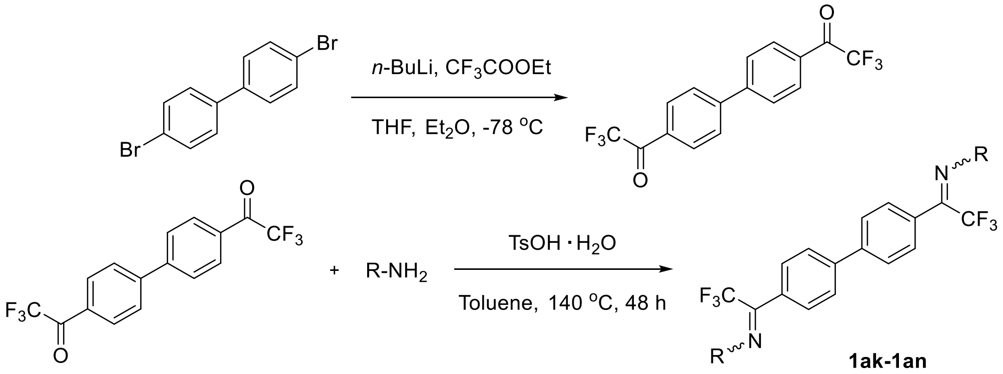

Scheme S6. General procedure for the synthesis of 1ak-1an, related to Scheme 2

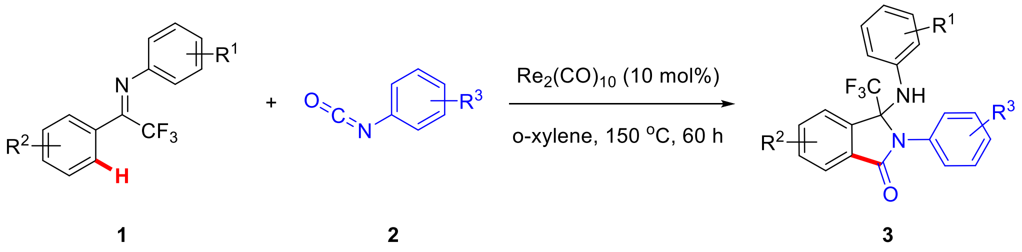

Scheme S7. General procedure for the synthesis of 3a-3be, related to Scheme 2, Scheme 3 and Figure 2

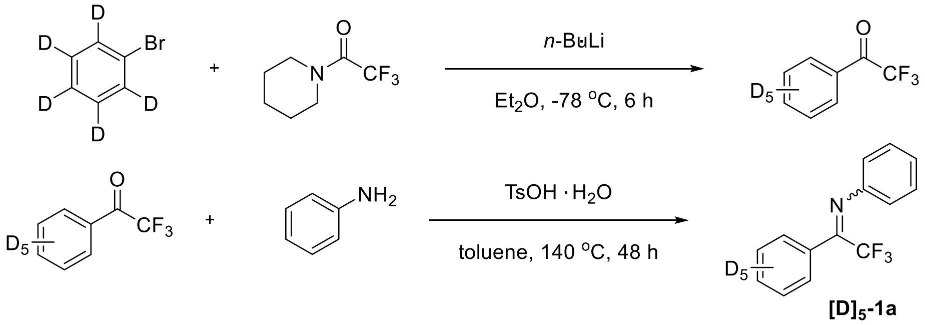

Scheme S8. Procedure for the synthesis of $[D]_5$-1a, related to Scheme 4

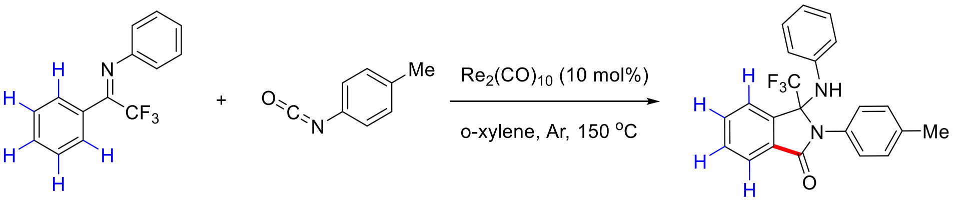

1a 2a 3a

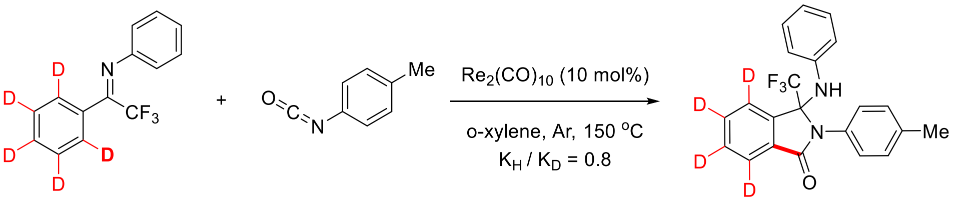

[D]$_5$-1a 2a [D]$_4$-3a

Scheme S9. Procedure for the synthesis of [D]$_5$-1a, related to Scheme 4

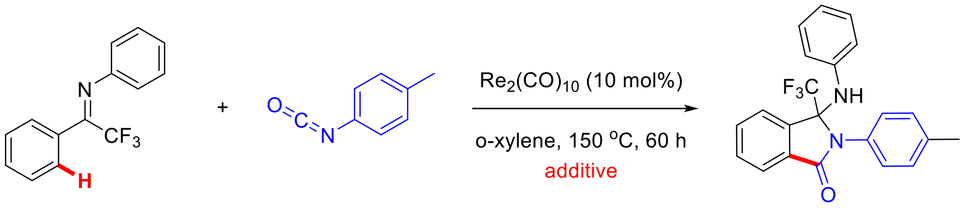

1a 2a 3a

Scheme S10. Control experiments under different additives, related to Scheme 5

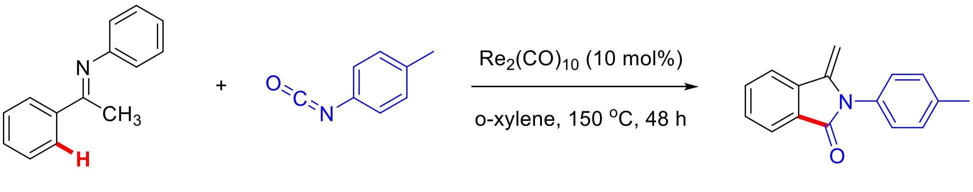

1aj 2a 3bf

Scheme S11. Control experiments use 1aj instead of 1a, related to Scheme 5

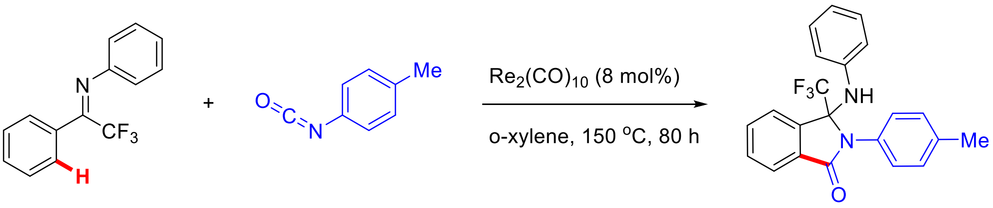

1a 2a 3a

Scheme S12. Procedure for the gram synthesis of 3a, related to Scheme 7

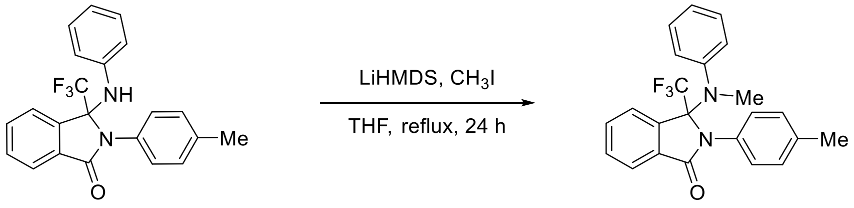

3a **4**

Scheme S13. Procedure for the synthesis of 4, related to Scheme 7

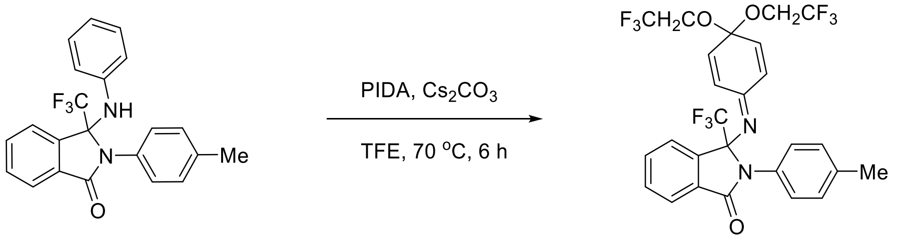

3a **5**

Scheme S14. Procedure for the synthesis of 5, related to Scheme 7

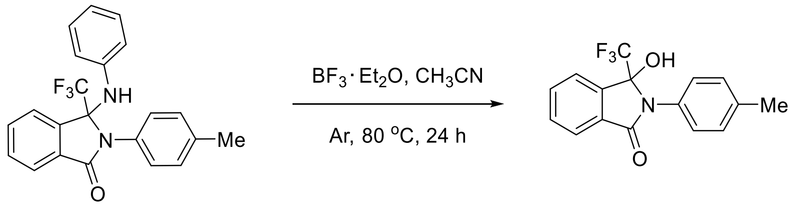

3a **6**

Scheme S15. Procedure for the synthesis of 6, related to Scheme 7

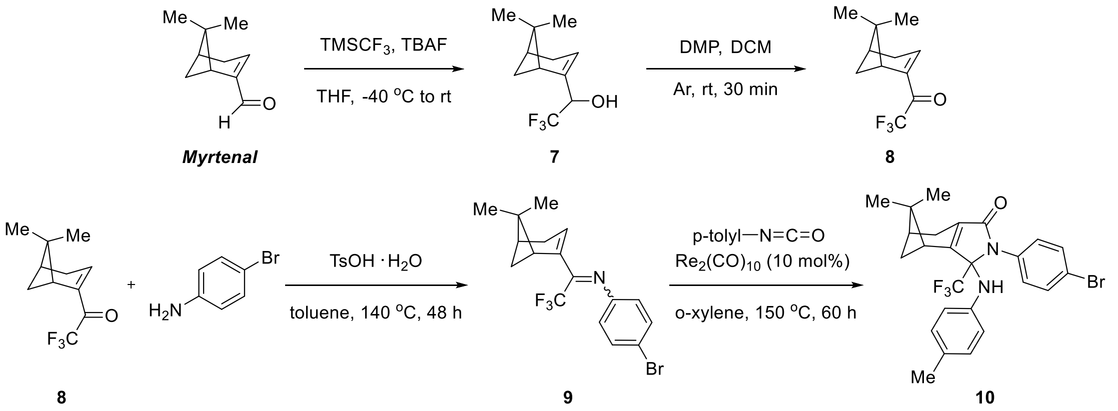

Scheme S16. Procedure for the synthesis of 10, related to Scheme 8

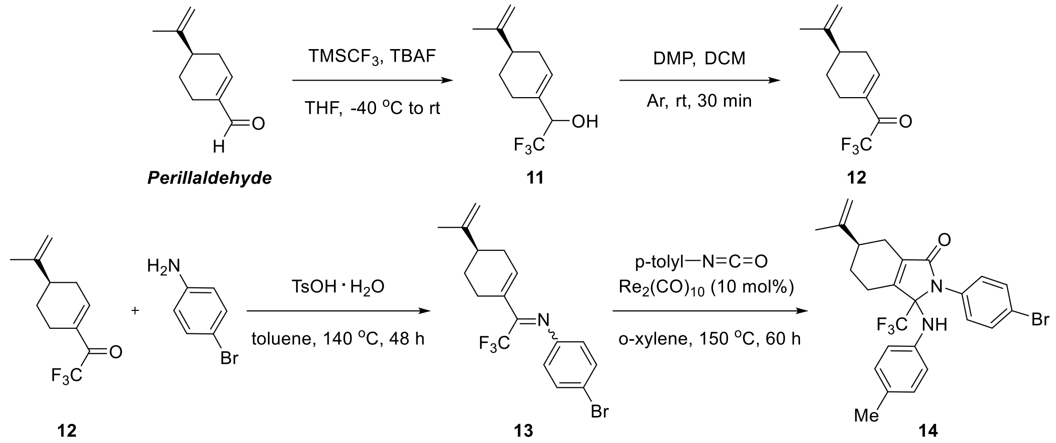

Scheme S17. Procedure for the synthesis of 14, related to Scheme 8

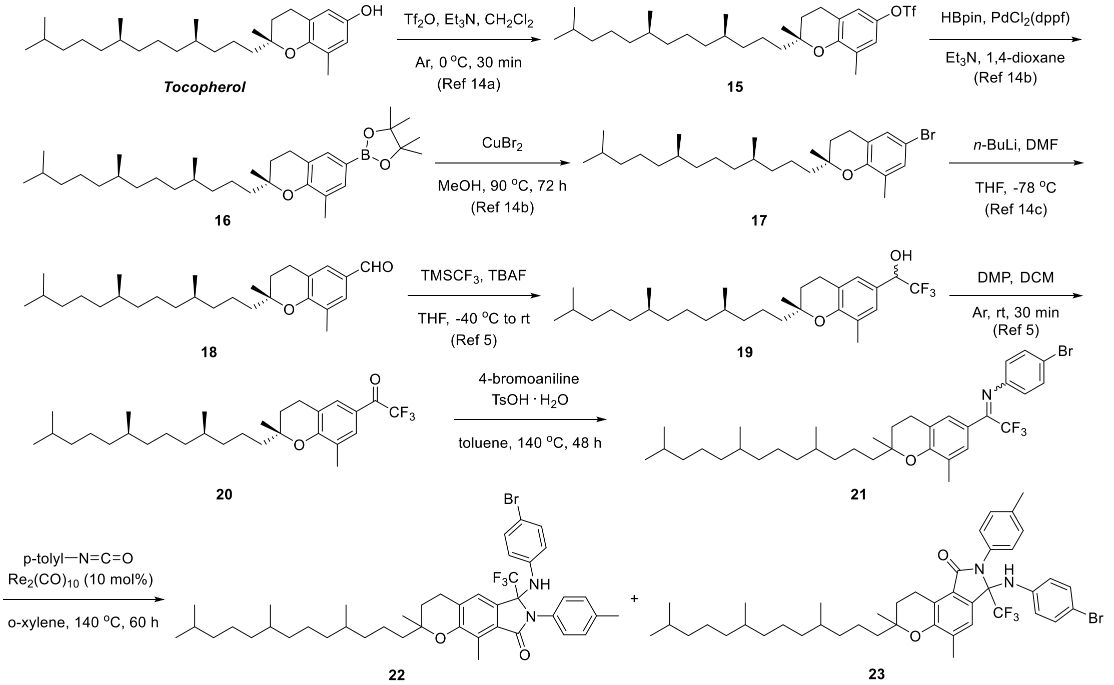

Scheme S18. Procedure for the synthesis of 22 and 23, related to Scheme 8

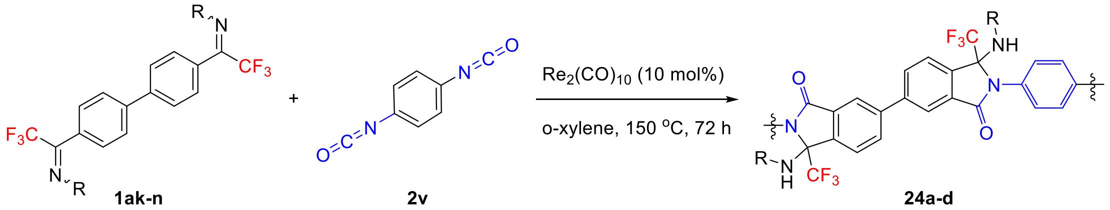

Scheme S19. Procedure for the synthesis of 24a-d, related to Figure 3

Supplementary Table

Table S1. ^{19}F NMR yield in different time, related to Scheme 4

Time（h）	1	2	3	4	5
**3a**	0	2.2	5.5	9.5	13.5
**[D]$_5$-3a**	0	3.5	6.9	11.4	16.5

Transparent Methods

General Methods for Experiments

Anhydrous o-xylene was purchased from Innochem Ltd (Extra Dry, with molecular sieves, Water≤50 ppm, in resealable bottle), anhydrous PhCl was purchased from Energy Chemical Ltd (Extra Dry, with molecular sieves, Water≤50 ppm, Energyseal), and these were degassed before using. Anhydrous THF was purchased from J&K Scientific Ltd (Super Dry, with molecular sieves, Water≤50 ppm, J&K seal). Diethyl ether was distilled over sodium prior to use. $Re_2(CO)_{10}$ was purchased from Strem chemicals, Inc. All the isocyanates were commercially available and were used as received unless otherwise stated

All reactions were carried out using oven-dried glassware and magnetic stirring under argon gas unless otherwise stated. Reaction temperatures are reported as the temperature of the bath surrounding the vessel. Analytical thin layer chromatography was performed on silica gel aluminum plates with F-254 indicator and visualized by UV light (254 nm). Column chromatography was performed using 200-300 mesh silica gel. NMR spectra were recorded on AVANCE III HD 400 MHz. Chemical shifts (δ) are quoted in ppm relative to TMS (^{1}H) and $CFCl_3$ (^{19}F). Coupling constants (J) are quoted in Hz. The following abbreviations were used to show the multiplicities: s: singlet, d: doublet, t: triplet, q: quadruplet, dd: doublet of doublet, m: multiplet. The residual solvent signals were used as references ($CDCl_3$: δ_H = 7.26 ppm, δ_C = 77.00 ppm or relative to external $CFCl_3$, δ_F = 0 ppm). High-resolution mass spectrometry (HRMS) was carried out on a Waters Xevo G2-XS QTof. IR spectra were recorded on a VERTEX 70, the wave numbers of recorded IR-signals are quoted in cm^{-1}. Ultraviolet-visible-near infrared spectrophotometer (UV-vis) were recorded on a UH4150. Molecular weight (Mn) and molecular weight distribution (Mw/Mn) values were determined by Advanced Polymer Chromatographyy (ACQUITY APC). The APC system used THF as the eluent at a flow rate of 1.0 mL/min at 40 °C using linear PMMA standards.

General procedure for the synthesis of derivatives

General procedure A (1a-1u, 1w-1y, 1af, 1ai, 1aj) (Scheme S1) (Elliott et al., 2019; Dai and Cahard, 2014)

To a solution of 2,2,2-trifluoroacetephenone (10 mmol, 1 equiv) in toluene (90 mL) was added aniline (13.9 mmol, 1.39 equiv) followed by *p*-toluenesulfonic acid monohydrate

(95.1 mg, 0.5 mmol, 5 mol%). The resulting reaction mixture was heated at 140 °C for 24 h with removal of water *via* Dean-Stark trap. After cooling to room temperature, the reaction mixture was concentrated under vacuum. The residue was purified by flash column chromatography on silica gel to give the corresponding ketimine **1a-1u, 1w-1y, 1af, 1ai** and **1aj**.

General procedure B (1aa, 1ae) (Scheme S2) (Strømgaard et al., 2002)

To a solution of bromobenzene (10 mmol, 1 equiv) in dry Et_2O (30 mL) at -78 °C was slowly added *n*-BuLi (2.5 M in hexane, 1.1 equiv). After that, the reaction mixture was warmed to 0 °C and stirred at that temperature for 2 h. Then the reaction mixture was cooled down to -60 °C and a solution of *N*-trifluoroacetylpiperidine (2.2 mL, 15 mmol, 1.5 equiv) in dry Et_2O (10 mL) was added in portions. The resulting reaction mixture was stirred at -60 °C for 3 h and then warmed to room temperature. The reaction mixture was then quenched by the addition of the saturated aqueous NH_4Cl (50 mL) and the organic phase was subsequently washed with saturated aqueous NH_4Cl (5 × 50 mL) and H_2O (3 × 50 mL). The combined organic layers was dried over Na_2SO_4, filtered and the volatiles were removed under vacuum. The crude was used directly for the synthesis of **1aa** or **1ae** without further purification according to procedure A (2 equiv of aniline and 10 mol% of *p*-toluenesulfonic acid monohydrate were used).

General procedure C (1v, 1z, 1ab-1ad) (Scheme S3) (Fujita et al., 2017)

To a solution of ethyl trifluoroacetate (1.2 equiv) in dry Et_2O (1.2 M) at -78 °C was slowly added Grignard reagent (1 equiv), and the resulting reaction mixture was stirred at that temperature for 1.5 h. Then, the reaction mixture was warmed to -50 °C and stirred for another 2 h. The reaction mixture was then quenched by the addition of the saturated aqueous NH_4Cl (40 mL). The organic layer were washed with brine (3 × 20 mL), dried over Na_2SO_4 and filtered. The volatiles were removed under vacuum, and the crude was used directly for the synthesis of **1v, 1z, 1ab-1ad** without further purification according to procedure A.

Procedure D (1ah) (Scheme S4) (Trost and Debien, 2015)

To a solution of cyclohex-1-ene-1-carbaldehyde (1.54 g, 14 mmol, 1 equiv) in THF (42 mL) was slowly added TBAF (1 M in THF, 9.8 mL, 0.7 equiv) and $TMSCF_3$ (4.6 mL, 30.8 mmol, 2.2 equiv) at -40 °C under Ar. After addition completed, the reaction mixture was slowly warmed to room temperature and stirred at that temperature for 20 h. The yellow reaction mixture was quenched by the addition of HCl (2 M, 7 mL) and then separated. The aqueous layer was extracted with Et_2O (3 × 40 mL) and the combined organic layers was dried over Na_2SO_4, filtered, and concentrated under

vacuum. The crude was used directly for the next step without further purification.

To a solution of DMP (15.6 g, 36.7 mmol, 1.2 equiv) in DCM (20 mL) was added the solution of 1-(cyclohex-1-en-1-yl)-2,2,2-trifluoroethan-1-ol in DCM (20 mL) at 0 °C. The resulting reaction mixture was stirred at room temperature for 12 h. Then aqueous NaOH (0.5 M, 10 mL) was added to quench the reaction and the mixture was extracted with Et_2O (3 × 40 mL). The combined organic layer was dried over Na_2SO_4, filtered and concentrated under vacuum to give the crude 1-(cyclohex-1-en-1-yl)-2,2,2-trifluoroethan-1-one which was used directly for the synthesis of **1ah** without further purification according to procedure A.

Procedure E (1ag) (Scheme S5) (Chen et al., 1983)

To a solution of 1,4-dibromobenzene (17 mmol, 1 equiv) in THF and Et_2O (76 mL, 1:1) was slowly added *n*-BuLi (2.5 M in hexane, 1 equiv) at -78 °C. Then the reaction mixture was warmed to -75 °C and stirred at that temperature for 10 min. After that, the reaction mixture was cooled to -78 °C, and ethyl trifluoroacetate (2.02 mL, 17 mmol, 1 equiv) was slowly added. Then the reaction mixture was warmed to -70 °C and stirred at that temperature for 30 min. After that the reaction mixture was cooled to -78 °C again, and *n*-BuLi (2.5 M in hexane, 1 equiv) was added. The obtained reaction mixture was allowed to warm to -73 °C and stirred at that temperature for 10 min. Then the mixture was cooled to -78 °C again, and ethyl trifluoroacetate (2.02 mL, 17 mmol, 1 equiv) was slowly added. After that, the obtained solution was warmed to -66 °C and stirred at that temperature for 10 min. Precooled mixture of HCl (2 M, 10 mL) and EtOH (5 mL) was added to the reaction mixture to quench the reaction. The obtained organic layer was washed with aqueous HCl (2 M, 3 × 50 mL), dried over Na_2SO_4, filtered. After concentrated under vacuum, the crude was used directly for the next step without further purification according to procedure A (2 mmol of 1,1'-(1,4-phenylene)bis(2,2,2-trifluoroethan-1-one), 4 equiv of aniline and 20 mol% of *p*-toluenesulfonic acid monohydrate were used).

General procedure F (1ak-1an) (Scheme S6) (Chen et al., 1983)

The 1,1'-([1,1'-biphenyl]-4,4'-diyl)bis(2,2,2-trifluoroethan-1-one) was synthesized according to procedure E on a 20 mmol scale. The residue was purified by flash column chromatography on silica gel to give the corresponding product, yield = 69% (4.8 g).

To a solution of 1,1'-([1,1'-biphenyl]-4,4'-diyl)bis(2,2,2-trifluoroethan-1-one) (1.04 g, 3 mmol, 1 equiv) in toluene (90 mL) was added aniline (12 mmol, 4 equiv) followed by *p*-toluenesulfonic acid monohydrate (114.1 mg, 0.6 mmol, 0.2 equiv). The reaction mixture was heated at 140 °C for 48 h with removal of water *via* Dean-Stark trap. After

cooling to room temperature, the reaction mixture was concentrated under vacuum. The residue was purified by flash column chromatography on silica gel to give **1ak-1an**.

Purification and characterization of derivatives 1

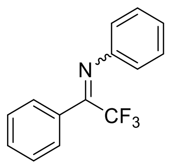

1a

2,2,2-trifluoro-*N*,1-diphenylethan-1-imine 1a. (Elliott et al., 2019; Abid et al., 2007). Following general procedure A, on a 10 mmol scale. The product was purified by flash column chromatography on silica gel (height 16 cm, width 4.5 cm, eluent: petroleum ether + 3% Et_3N) as a yellow oil, yield = 74% (1.85 g). R_f (petroleum ether): 0.5. **^{1}H NMR** (400 MHz, $CDCl_3$) δ 7.35 – 7.09 (m, 7H), 7.01 (t, J = 7.6 Hz, 1H), 6.73 (d, J = 7.2 Hz, 2H). **^{19}F NMR** (376 MHz, $CDCl_3$) δ -70.5 (s). **^{13}C NMR** (101 MHz, $CDCl_3$) δ 157.0 (q, J = 34.3 Hz), 147.1, 130.1, 130.0, 128.7, 128.6, 128.4, 125.3, 120.5, 119.9 (q, J = 279.8 Hz). **IR** (KBr, cm^{-1}) ν: 3404, 3069, 2928, 1664, 1591, 1487, 1330, 1194, 1135, 971, 773, 697. **HRMS** (ESI) calcd for $C_{14}H_{11}F_3N^+$ *m/z* 250.0838 $[M+H]^+$, Found 250.0842.

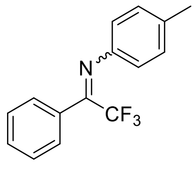

1b

2,2,2-trifluoro-1-phenyl-*N*-(p-tolyl)ethan-1-imine 1b. Following general procedure A, on a 10 mmol scale. The product was purified by flash column chromatography on silica gel (height 18 cm, width 3.5 cm, eluent: petroleum ether + 3% Et_3N) as a yellow oil, yield = 77% (2.03 g). R_f (petroleum ether): 0.6. **^{1}H NMR** (400 MHz, $CDCl_3$) δ 7.36 – 7.15 (m, 5H), 6.96 (d, J = 8.0 Hz, 2H), 6.64 (d, J = 7.2 Hz, 2H), 2.21 (s, 3H). **^{19}F NMR** (376 MHz, $CDCl_3$) δ -70.4 (s). **^{13}C NMR** (101 MHz, $CDCl_3$) δ 156.4 (q, J = 34.3 Hz), 144.4, 135.3, 130.4, 130.1, 129.4, 128.6, 128.5, 120.9, 120.0 (q, J = 279.8 Hz), 20.8. **IR** (KBr, cm^{-1}) ν: 3452, 3034, 2926, 1659, 1502, 1449, 1331, 1234, 1194, 1133, 972, 826, 697. **HRMS** (ESI) calcd for $C_{15}H_{13}F_3N^+$ *m/z* 264.0995 $[M+H]^+$, Found 264.0998.

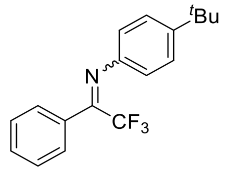

1c

***N*-(4-(tert-butyl)phenyl)-2,2,2-trifluoro-1-phenylethan-1-imine 1c.** Following general procedure A , on a 10 mmol scale. The product was purified by flash column chromatography on silica gel (height 18 cm, width 3.5 cm, eluent: petroleum ether + 3% Et_3N) as an orange solid, yield = 62% (1.89 g). R_f (petroleum ether): 0.5. **^{1}H NMR** (400 MHz, $CDCl_3$) δ 7.41 – 7.29 (m, 3H), 7.27 – 7.17 (m, 4H), 6.71 (d, J = 8.5 Hz, 2H), 1.26 (s, 9H). **^{19}F NMR** (376 MHz, $CDCl_3$) δ -70.4 (s). **^{13}C NMR** (101 MHz, $CDCl_3$) δ 156.1 (q, J = 33.3 Hz), 148.7, 144.2, 130.4, 130.1, 128.6, 128.5, 125.6, 120.8, 120.0 (q, J = 280.8 Hz), 34.4, 31.2. **IR** (KBr, cm^{-1}) ν: 3432, 3070, 2961, 1665, 1501, 1325, 1197, 1128, 970, 836, 702. **HRMS** (ESI) calcd for $C_{18}H_{19}F_3N^+$ *m/z* 306.1464 $[M+H]^+$, Found 306.1467.

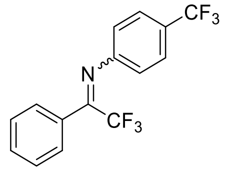

1d

2,2,2-trifluoro-1-phenyl-*N*-(4-(trifluoromethyl)phenyl)ethan-1-imine 1d. Following general procedure A, on a 10 mmol scale. The product was purified by flash column chromatography on silica gel (height 20 cm, width 3.5 cm, eluent: petroleum ether + 3% Et_3N) as a yellow solid, yield = 46% (1.46 g). R_f (petroleum ether): 0.6. **^{1}H NMR** (400 MHz, $CDCl_3$) δ 7.48 (d, J = 8.4 Hz, 2H), 7.43 – 7.28 (m, 3H), 7.22 (d, J = 7.6 Hz, 2H), 6.84 (d, J = 8.0 Hz, 2H). **^{19}F NMR** (376 MHz, $CDCl_3$) δ -62.8 (s), -70.6 (s). **^{13}C NMR** (101 MHz, $CDCl_3$) δ 158.5 (q, J = 34.3 Hz), 150.3, 130.7, 129.3, 128.7, 128.5, 127.2 (q, J = 33.3 Hz), 126.1 (q, J = 3.0 Hz), 124.0 (q, J = 272.7 Hz), 120.3, 119.6 (q, J = 280.8 Hz). **IR** (KBr, cm^{-1}) ν: 3395, 3069, 1669, 1612, 1326, 1196, 1133, 971, 846, 699. **HRMS** (ESI) calcd for $C_{15}H_{10}F_6N^+$ *m/z* 318.0712 $[M+H]^+$, Found 318.0721.

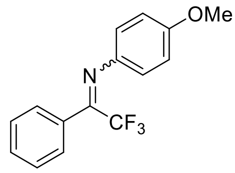

1e

2,2,2-trifluoro-*N*-(4-methoxyphenyl)-1-phenylethan-1-imine 1e. (Henseler et al., 2011). Following general procedure A, on a 10 mmol scale. The product was purified by flash column chromatography on silica gel (height 16 cm, width 3.5 cm, eluent: petroleum ether + 3% Et_3N) as a yellow oil, yield = 85% (2.36 g). R_f (petroleum ether): 0.2. **^{1}H NMR** (400 MHz, $CDCl_3$) δ 7.43 – 7.26 (m, 3H), 7.22 (d, J = 7.2 Hz, 2H), 6.79 – 6.63 (m, 4H), 3.71 (s, 3H). **^{19}F NMR** (376 MHz, $CDCl_3$) δ -70.4 (s). **^{13}C NMR** (101 MHz, $CDCl_3$) δ 157.8, 155.4 (q, J = 34.3 Hz), 139.7, 130.7, 130.1, 128.7, 128.6, 123.3, 120.0 (q, J = 279.8 Hz), 114.0, 55.2. **IR** (KBr, cm^{-1}) ν: 3457, 3063, 2954, 1653, 1600, 1502, 1294, 1193, 1034, 971, 838, 774, 697. **HRMS** (ESI) calcd for $C_{15}H_{13}F_3NO^+$ *m/z* 280.0944 $[M+H]^+$, Found 280.0945.

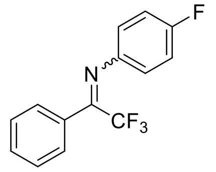

1f

2,2,2-trifluoro-*N*-(4-fluorophenyl)-1-phenylethan-1-imine 1f. Following general procedure A, on a 10 mmol scale. The product was purified by flash column chromatography on silica gel (height 14 cm, width 3.5 cm, eluent: petroleum ether + 3% Et_3N) as a yellow solid, yield = 85% (2.28 g). R_f (petroleum ether): 0.6. **^{1}H NMR** (400 MHz, $CDCl_3$) δ 7.42 – 7.29 (m, 3H), 7.22 (d, J = 7.6 Hz, 2H), 6.94 – 6.84 (m, 2H), 6.74 (dd, J = 8.4, 4.8 Hz, 2H). **^{19}F NMR** (376 MHz, $CDCl_3$) δ -70.6 (s), -117.5 (s). **^{13}C NMR** (101 MHz, $CDCl_3$) δ 160.5 (d, J = 246.4 Hz), 157.4 (q, J = 33.3 Hz), 143.0, 130.4, 130.0, 128.7, 128.5, 122.6 (d, J = 8.1 Hz), 119.7 (q, J = 279.8 Hz), 115.7 (d, J = 23.2 Hz). **IR** (KBr, cm^{-1}) ν: 3452, 3066, 2926, 1661, 1500, 1330, 1206, 1137, 972, 843, 782, 699. **HRMS** (ESI) calcd for $C_{14}H_{10}F_4N^+$ *m/z* 268.0744 $[M+H]^+$, Found 268.0749.

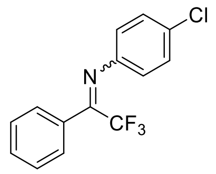

1g

***N*-(4-chlorophenyl)-2,2,2-trifluoro-1-phenylethan-1-imine 1g.** (Li et al., 2010). Following general procedure A, on a 14.5 mmol scale. The product was purified by flash column chromatography on silica gel (height 14 cm, width 3.5 cm, eluent: petroleum ether + 3% Et_3N) as a pale yellow solid, yield = 23% (0.94 g). R_f (petroleum ether): 0.8. **^{1}H NMR** (400 MHz, $CDCl_3$) δ 7.43 – 7.28 (m, 3H), 7.25 – 7.10 (m, 4H), 6.70 (d, J = 8.8 Hz, 2H). **^{19}F NMR** (376 MHz, $CDCl_3$) δ -70.6 (s). **^{13}C NMR** (101 MHz,

$CDCl_3$) δ 157.7 (q, J = 34.3 Hz), 145.5, 131.0, 130.5, 129.7, 129.0, 128.7, 128.5, 122.1, 119.7 (q, J = 280.8 Hz). **IR** (KBr, cm^{-1}) ν: 3432, 3070, 1663, 1485, 1330, 1195, 1135, 971, 834, 704. **HRMS** (ESI) calcd for $C_{14}H_{10}ClF_3N^+$ *m/z* 284.0448 $[M+H]^+$, Found 284.0459.

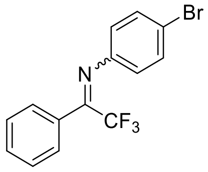

1h

***N*-(4-bromophenyl)-2,2,2-trifluoro-1-phenylethan-1-imine 1h.** Following general procedure A, on a 10 mmol scale. The product was purified by flash column chromatography on silica gel (height 14 cm, width 3.5 cm, eluent: petroleum ether + 3% Et_3N) as a pale yellow solid, yield = 59% (1.92 g). R*f* (petroleum ether): 0.6. **^{1}H NMR** (400 MHz, $CDCl_3$) δ 7.42 – 7.28 (m, 5H), 7.22 (d, J = 7.2 Hz, 2H), 6.64 (d, J = 8.8 Hz, 2H). **^{19}F NMR** (376 MHz, $CDCl_3$) δ -70.6 (s). **^{13}C NMR** (101 MHz, $CDCl_3$) δ 157.7 (q, J = 34.3 Hz), 146.0, 131.9, 130.5, 129.6, 128.7, 128.5, 122.3, 119.7 (q, J = 279.8 Hz), 118.8. **IR** (KBr, cm^{-1}) ν: 3441, 3077, 1661, 1480, 1332, 1199, 1132, 969, 823, 707. **HRMS** (ESI) calcd for $C_{14}H_{10}BrF_3N^+$ *m/z* 327.9943 $[M+H]^+$, Found 327.9953.

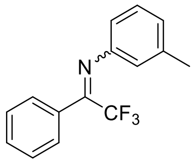

1i

2,2,2-trifluoro-1-phenyl-*N*-(m-tolyl)ethan-1-imine 1i. Following general procedure A, on a 10 mmol scale. The product was purified by flash column chromatography on silica gel (height 18 cm, width 3.5 cm, eluent: petroleum ether + 3% Et_3N) as a yellow oil, yield = 45% (1.19 g). R*f* (petroleum ether): 0.9. **^{1}H NMR** (400 MHz, $CDCl_3$) δ 7.41 – 7.28 (m, 3H), 7.25 (d, J = 7.6 Hz, 2H), 7.07 (t, J = 7.6 Hz, 1H), 6.88 (d, J = 7.6 Hz, 1H), 6.65 (s, 1H), 6.52 (d, J = 8.0 Hz, 1H), 2.25 (s, 3H). **^{19}F NMR** (376 MHz, $CDCl_3$) δ -70.5 (s). **^{13}C NMR** (101 MHz, $CDCl_3$) δ 156.7 (q, J = 34.3 Hz), 147.1, 138.7, 130.1, 128.6, 128.55, 128.54, 128.4, 126.1, 121.4, 119.9 (q, J = 279.8 Hz), 117.4, 21.2. **IR** (KBr, cm^{-1}) ν: 3446, 3060, 2925, 1663, 1595, 1485, 1328, 1199, 1134, 972, 873, 782. **HRMS** (ESI) calcd for $C_{15}H_{13}F_3N^+$ *m/z* 264.0995 $[M+H]^+$, Found 264.1001.

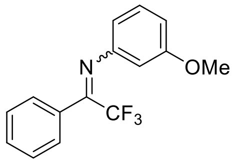

1j

2,2,2-trifluoro-*N*-(3-methoxyphenyl)-1-phenylethan-1-imine 1j. Following general procedure A, on a 10 mmol scale. The product was purified by flash column chromatography on silica gel (height 18 cm, width 3.5 cm, eluent: petroleum ether + 3% Et_3N) as a yellow oil, yield = 38% (1.07 g). R_f (petroleum ether): 0.4. **^{1}H NMR** (400 MHz, $CDCl_3$) δ 7.38 – 7.19 (m, 5H), 7.06 (t, J = 8.4 Hz, 1H), 6.58 (dd, J = 8.4, 2.4 Hz, 1H), 6.33 (t, J = 2.4 Hz, 1H), 6.29 (d, J = 8.0 Hz, 1H), 3.66 (s, 3H). **^{19}F NMR** (376 MHz, $CDCl_3$) δ -70.5 (s). **^{13}C NMR** (101 MHz, $CDCl_3$) δ 160.1, 157.1 (q, J = 33.3 Hz), 148.4, 130.2, 130.1, 129.6, 128.5, 128.5, 112.7, 119.9 (q, J = 279.8 Hz), 111.2, 106.2, 54.8. **IR** (KBr, cm^{-1}) ν: 3416, 3069, 2953, 1594, 1481, 1327, 1200, 1136, 1043, 973, 777, 698. **HRMS** (ESI) calcd for $C_{15}H_{13}F_3NO^+$ *m/z* 280.0944 $[M+H]^+$, Found 280.0945.

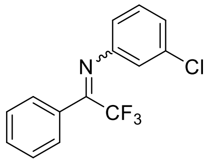

1k

***N*-(3-chlorophenyl)-2,2,2-trifluoro-1-phenylethan-1-imine 1k.** Following general procedure A, on a 10 mmol scale. The product was purified by flash column chromatography on silica gel (height 18 cm, width 3.5 cm, eluent: petroleum ether + 3% Et_3N) as a yellow oil, yield = 61% (1.74 g). R_f (petroleum ether): 0.5. **^{1}H NMR** (400 MHz, $CDCl_3$) δ 7.38 – 7.16 (m, 5H), 7.07 (t, J = 8.0 Hz, 1H), 6.99 (d, J = 8.4 Hz, 1H), 6.79 (t, J = 2.0 Hz, 1H), 6.57 (d, J = 8.0 Hz, 1H). **^{19}F NMR** (376 MHz, $CDCl_3$) δ -70.6 (s). **^{13}C NMR** (101 MHz, $CDCl_3$) δ 158.1 (q, J = 34.3 Hz), 148.3, 134.5, 130.6, 129.9, 129.4, 128.6, 128.5, 125.3, 120.6, 119.7 (q, J = 280.8 Hz), 118.4. **IR** (KBr, cm^{-1}) ν: 3403, 3069, 1666, 1583, 1464, 1330, 1195, 1137, 971, 877, 783, 701. **HRMS** (ESI) calcd for $C_{14}H_{10}ClF_3N^+$ *m/z* 284.0448 $[M+H]^+$, Found 284.0456.

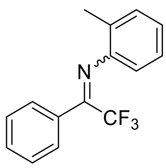

1l

2,2,2-trifluoro-1-phenyl-*N*-(o-tolyl)ethan-1-imine 1l. (Kiselyov, 1999). Following general procedure A, on a 10 mmol scale. The product was purified by flash column chromatography on silica gel (height 18 cm, width 3.5 cm, eluent: petroleum ether + 3%

Et_3N) as a yellow oil, yield = 63% (1.65 g). R*f* (petroleum ether): 0.9. **^{1}H NMR** (400 MHz, $CDCl_3$) δ 7.32 – 7.14 (m, 5H), 7.10 (d, *J* = 6.8 Hz, 1H), 6.96 – 6.85 (m, 2H), 6.37 (d, *J* = 7.2 Hz, 1H), 2.18 (s, 3H). **^{19}F NMR** (376 MHz, $CDCl_3$) δ -70.0 (s). **^{13}C NMR** (101 MHz, $CDCl_3$) δ 156.5 (q, *J* = 33.3 Hz), 146.1, 130.4, 130.3, 130.1, 128.4, 128.4, 128.2, 126.1, 125.1, 119.8 (q, *J* = 280.8 Hz), 118.5, 17.7. **IR** (KBr, cm^{-1}) ν: 3401, 3067, 2925, 1664, 1486, 1330, 1195, 1136, 970, 771, 697. **HRMS** (ESI) calcd for $C_{15}H_{13}F_3N^+$ *m/z* 264.0995 $[M+H]^+$, Found 264.0999.

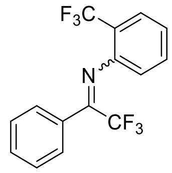

1m

2,2,2-trifluoro-1-phenyl-*N*-(2-(trifluoromethyl)phenyl)ethan-1-imine 1m. (Patterson et al., 1992). Following general procedure A, on a 10 mmol scale. The product was purified by flash column chromatography on silica gel (height 18 cm, width 3.5 cm, eluent: petroleum ether + 3% Et_3N) as a yellow oil, yield = 36% (1.15 g). R*f* (petroleum ether): 0.6. **^{1}H NMR** (400 MHz, $CDCl_3$) δ 7.62 (d, *J* = 8.0 Hz, 1H), 7.37 – 7.06 (m, 7H), 6.43 (d, *J* = 6.8 Hz, 1H). **^{19}F NMR** (376 MHz, $CDCl_3$) δ -70.6 (s), -61.7 (s). **^{13}C NMR** (101 MHz, $CDCl_3$) δ 158.4 (q, *J* = 35.4 Hz), 145.4, 132.4, 130.7, 129.4, 128.6, 128.4, 126.4 (q, *J* = 5.1 Hz), 124.9, 123.8 (q, *J* = 274.7 Hz), 121.4 (q, *J* = 30.3 Hz), 119.6, 119.6 (q, *J* = 280.8 Hz). **IR** (KBr, cm^{-1}) ν: 3323, 3073, 1673, 1594, 1451, 1324, 1198, 1125, 1046, 968, 768, 699. **HRMS** (ESI) calcd for $C_{15}H_{10}F_6N^+$ *m/z* 318.0712 $[M+H]^+$, Found 318.0714.

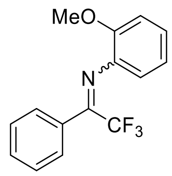

1n

2,2,2-trifluoro-*N*-(2-methoxyphenyl)-1-phenylethan-1-imine 1n. Following general procedure A, on a 10 mmol scale. The product was purified by flash column chromatography on silica gel (height 18 cm, width 3.5 cm, eluent: petroleum ether + 3% Et_3N) as a yellow solid, yield = 65% (1.81 g). R*f* (petroleum ether): 0.3. **^{1}H NMR** (400 MHz, $CDCl_3$) δ 7.32 – 7.17 (m, 5H), 6.98 (t, *J* = 7.6 Hz, 1H), 6.79 – 6.70 (m, 2H), 6.63 (d, *J* = 7.6 Hz, 1H), 3.62 (s, 3H). **^{19}F NMR** (376 MHz, $CDCl_3$) δ -70.1 (s). **^{13}C NMR** (101 MHz, $CDCl_3$) δ 158.4 (q, *J* = 34.3 Hz), 148.6, 136.8, 130.8, 130.1, 128.2, 127.8, 126.1, 120.6, 120.5, 117.1 (q, *J* = 280.8 Hz), 111.4, 55.2. **IR** (KBr, cm^{-1}) ν: 3306, 3067,

2952, 1666, 1590, 1491, 1453, 1332, 1191, 1131, 1033, 971, 750, 698. **HRMS** (ESI) calcd for $C_{15}H_{13}F_3NO^+$ *m/z* 280.0944 $[M+H]^+$, Found 280.0945.

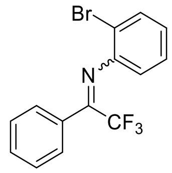

1o

***N*-(2-bromophenyl)-2,2,2-trifluoro-1-phenylethan-1-imine 1o.** (Wang et al., 2013). Following general procedure A, on a 10 mmol scale. The product was purified by flash column chromatography on silica gel (height 18 cm, width 3.5 cm, eluent: petroleum ether + 3% Et_3N) as a yellow oil, yield = 44% (1.46 g). R_f (petroleum ether): 0.5. **^{1}H NMR** (400 MHz, $CDCl_3$) δ 7.47 (d, J = 8.0 Hz, 1H), 7.33 – 7.19 (m, 5H), 7.01 (t, J = 7.6 Hz, 1H), 6.83 (t, J = 8.0 Hz, 1H), 6.47 (d, J = 8.0 Hz, 1H). **^{19}F NMR** (376 MHz, $CDCl_3$) δ -70.2 (s). **^{13}C NMR** (101 MHz, $CDCl_3$) δ 158.7 (q, J = 34.3 Hz), 146.3, 132.6, 130.5, 129.5, 128.4, 127.9, 127.7, 125.9, 119.7, 119.6 (q, J = 279.8 Hz), 114.0. **IR** (KBr, cm^{-1}) ν: 3392, 3066, 1672, 1464, 1332, 1193, 1140, 1035, 970, 703. **HRMS** (ESI) calcd for $C_{14}H_{10}BrF_3N^+$ *m/z* 327.9943 $[M+H]^+$, Found 327.9946.

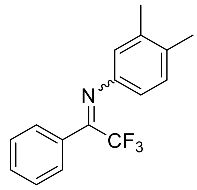

1p

***N*-(3,4-dimethylphenyl)-2,2,2-trifluoro-1-phenylethan-1-imine 1p.** Following general procedure A, on a 10 mmol scale. The product was purified by flash column chromatography on silica gel (height 15 cm, width 8 cm, eluent: petroleum ether + 3% Et_3N) as a yellow oil, yield = 32% (0.89 g). R_f (petroleum ether): 0.9. **^{1}H NMR** (400 MHz, $CDCl_3$) δ 7.38 – 7.17 (m, 5H), 6.89 (d, J = 8.0 Hz, 1H), 6.61 (s, 1H), 6.42 (dd, J = 8.0, 1.6 Hz, 1H), 2.14 (s, 3H), 2.11 (s, 3H). **^{19}F NMR** (376 MHz, $CDCl_3$) δ -70.4 (s). **^{13}C NMR** (101 MHz, $CDCl_3$) δ 156.0 (q, J = 33.3 Hz), 144.6, 137.8, 137.0, 133.9, 130.5, 130.0, 129.8, 129.0, 128.55, 128.45, 128.2, 125.2, 122.6, 120.0 (q, J = 280.8 Hz), 118.1, 21.3, 19.5, 19.1. **IR** (KBr, cm^{-1}) ν: 3460, 3027, 2932, 1660, 1495, 1328, 1198, 1133, 973, 820, 697. **HRMS** (ESI) calcd for $C_{16}H_{15}F_3N^+$ *m/z* 278.1151 $[M+H]^+$, Found 278.1159.

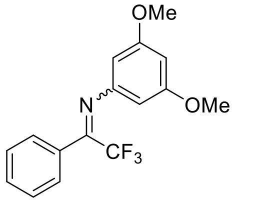

1q

***N*-(3,5-dimethoxyphenyl)-2,2,2-trifluoro-1-phenylethan-1-imine 1q.** Following general procedure A, on a 10 mmol scale. The product was purified by flash column chromatography on silica gel (height 18 cm, width 3.5 cm, eluent: petroleum ether + 3% Et_3N) as a yellow oil, yield = 29% (0.90 g). R_f (petroleum ether): 0.2. **^{1}H NMR** (400 MHz, $CDCl_3$) δ 7.39 – 7.21 (m, 5H), 6.15 (t, *J* = 2.0 Hz, 1H), 5.91 (d, *J* = 2.4 Hz, 2H), 3.62 (s, 6H). **^{19}F NMR** (376 MHz, $CDCl_3$) δ -70.5 (s). **^{13}C NMR** (101 MHz, $CDCl_3$) δ 160.9, 157.0 (q, *J* = 33.3 Hz), 148.8, 130.1, 129.9, 128.3, 128.2, 119.8 (q, *J* = 280.8 Hz), 98.6, 97.4, 54.8. **IR** (KBr, cm^{-1}) ν: 3463, 3007, 2953, 2843, 1666, 1597, 1462, 1329, 1204, 1134, 1062, 984, 836, 705, 645. **HRMS** (ESI) calcd for $C_{16}H_{15}F_3NO_2^+$ *m/z* 310.1049 $[M+H]^+$, Found 310.1055.

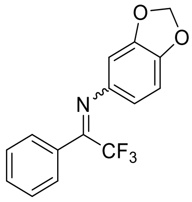

1r

***N*-(benzo[*d*][1,3]dioxol-5-yl)-2,2,2-trifluoro-1-phenylethan-1-imine 1r.** Following general procedure A, on a 10 mmol scale. The product was purified by flash column chromatography on silica gel (height 18 cm, width 3.5 cm, eluent: petroleum ether + 3% Et_3N) as an pale yellow solid, yield = 38% (1.11 g). R_f (petroleum ether): 0.2. **^{1}H NMR** (400 MHz, $CDCl_3$) δ 7.42 – 7.28 (m, 3H), 7.24 (d, *J* = 7.2 Hz, 2H), 6.61 (d, *J* = 8.8 Hz, 1H), 6.35 – 6.21 (m, 2H), 5.86 (s, 2H). **^{19}F NMR** (376 MHz, $CDCl_3$) δ -70.4 (s). **^{13}C NMR** (101 MHz, $CDCl_3$) δ 156.0 (q, *J* = 33.3 Hz), 147.8, 145.7, 141.1, 130.4, 130.2, 128.7, 128.5, 119.9 (q, *J* = 280.8 Hz), 115.3, 108.1, 103.0, 101.3. **IR** (KBr, cm^{-1}) ν: 3435, 3071, 2905, 1652, 1479, 1320, 1203, 1130, 1033, 972, 925, 850, 812, 702, 625. **HRMS** (ESI) calcd for $C_{15}H_{11}F_3NO_2^+$ *m/z* 294.0736 $[M+H]^+$, Found 294.0741.

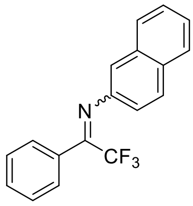

1s

2,2,2-trifluoro-*N*-(naphthalen-2-yl)-1-phenylethan-1-imine 1s. (Dai and Cahard,

2014). Following general procedure A, on a 10 mmol scale. The product was purified by flash column chromatography on silica gel (height 18 cm, width 4.5 cm, eluent: petroleum ether + 3% Et_3N) as a yellow oil, yield = 43% (1.30 g). R*f* (petroleum ether): 0.5. **^{1}H NMR** (400 MHz, $CDCl_3$) δ 7.79 – 7.53 (m, 3H), 7.42 – 7.08 (m, 8H), 6.86 (d, J = 8.8 Hz, 1H). **^{19}F NMR** (376 MHz, $CDCl_3$) δ -70.3 (s). **^{13}C NMR** (101 MHz, $CDCl_3$) δ 157.1 (q, J = 34.3 Hz), 144.6, 133.5, 131.2, 130.3, 130.0, 128.67, 128.65, 128.57, 127.8, 127.7, 126.5, 125.6, 120.4, 119.9 (q, J = 280.8 Hz), 118.2. **IR** (KBr, cm^{-1}) ν: 3402, 3061, 1664, 1328, 1195, 1135, 972, 755, 700. **HRMS** (ESI) calcd for $C_{18}H_{13}F_3N^+$ *m/z* 300.0995 $[M+H]^+$, Found 300.1002.

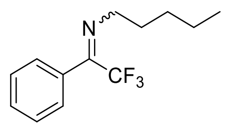

1t

2,2,2-trifluoro-*N*-pentyl-1-phenylethan-1-imine 1t. Following general procedure A, on a 10 mmol scale. The product was purified by flash column chromatography on silica gel (height 18 cm, width 3.5 cm, eluent: petroleum ether + 3% Et_3N) as a colorless oil, yield = 59% (1.44 g). R*f* (petroleum ether): 0.6. **^{1}H NMR** (400 MHz, $CDCl_3$) δ 7.51 – 7.37 (m, 3H), 7.26 – 7.15 (m, 2H), 3.46 – 3.31 (m, 2H), 1.72 – 1.60 (m, 2H), 1.34 – 1.18 (m, 4H), 0.86 (t, J = 6.8 Hz, 3H). **^{19}F NMR** (376 MHz, $CDCl_3$) δ -71.5 (s). **^{13}C NMR** (101 MHz, $CDCl_3$) δ 157.9 (q, J = 34.3 Hz), 130.5, 129.8, 128.6, 127.5, 119.7 (q, J = 279.8 Hz), 53.2, 29.8, 29.3, 22.2, 13.7. **IR** (KBr, cm^{-1}) ν: 3449, 3066, 2936, 2866, 1668, 1457, 1334, 1197, 1133, 1008, 956, 704. **HRMS** (ESI) calcd for $C_{13}H_{17}F_3N^+$ *m/z* 244.1308 $[M+H]^+$, Found 244.1317.

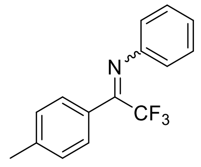

1u

2,2,2-trifluoro-*N*-phenyl-1-(p-tolyl)ethan-1-imine 1u. Following general procedure A, on a 10 mmol scale. The product was purified by flash column chromatography on silica gel (height 18 cm, width 3.5 cm, eluent: petroleum ether + 3% Et_3N) as an orange oil, yield = 43% (1.14 g). R*f* (petroleum ether): 0.7. **^{1}H NMR** (400 MHz, $CDCl_3$) δ 7.21 (t, J = 7.6 Hz, 2H), 7.15 – 7.01 (m, 5H), 6.76 (d, J = 7.6 Hz, 2H), 2.31 (s, 3H). **^{19}F NMR** (376 MHz, $CDCl_3$) δ -70.4 (s). **^{13}C NMR** (101 MHz, $CDCl_3$) δ 157.0 (q, J = 33.3 Hz), 147.3, 140.6, 129.2, 128.8, 128.6, 126.9, 125.1, 120.4, 119.9 (q, J = 280.8 Hz), 21.3. **IR** (KBr, cm^{-1}) ν: 3402, 3035, 2928, 1662, 1601, 1329, 1234, 1192, 1133, 971, 819, 766, 695. **HRMS** (ESI) calcd for $C_{15}H_{13}F_3N^+$ *m/z* 264.0995 $[M+H]^+$, Found 264.1002.

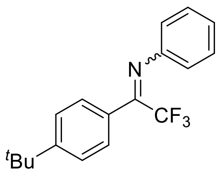

1v

1-(4-(tert-butyl)phenyl)-2,2,2-trifluoro-*N*-phenylethan-1-imine 1v. Following general procedure C, on a 15 mmol scale. The product was purified by flash column chromatography on silica gel (height 18 cm, width 3.5 cm, eluent: petroleum ether + 3% Et_3N) as a yellow oil, overall yield = 17% (0.76 g). R*f* (petroleum ether): 0.4. **^{1}H NMR** (400 MHz, $CDCl_3$) δ 7.28 (d, *J* = 8.4 Hz, 2H), 7.21 – 7.11 (m, 4H), 7.03 (t, *J* = 7.6 Hz, 1H), 6.75 (d, *J* = 7.6 Hz, 2H), 1.25 (s, 9H). **^{19}F NMR** (376 MHz, $CDCl_3$) δ -70.2 (s). **^{13}C NMR** (101 MHz, $CDCl_3$) δ 156.8 (q, *J* = 34.3 Hz), 153.7, 147.4, 128.8, 128.6, 126.8, 125.4, 125.1, 120.5, 120.0 (q, *J* = 280.8 Hz), 34.8, 31.0. **IR** (KBr, cm^{-1}) ν: 3432, 2966, 1716, 1605, 1194, 1139, 972, 838, 702. **HRMS** (ESI) calcd for $C_{18}H_{19}F_3N^+$ *m/z* 306.1464 $[M+H]^+$, Found 306.1471.

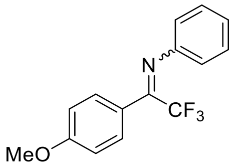

1w

2,2,2-trifluoro-1-(4-methoxyphenyl)-*N*-phenylethan-1-imine 1w. Following general procedure A, on a 3 mmol scale. The product was purified by flash column chromatography on silica gel (height 20 cm, width 3.5 cm, eluent: petroleum ether + 3% Et_3N) as a yellow oil, yield = 44% (0.37 g). R*f* (petroleum ether): 0.3. **^{1}H NMR** (400 MHz, $CDCl_3$) δ 7.27 – 7.10 (m, 4H), 7.03 (t, *J* = 7.6 Hz, 1H), 6.81 – 6.68 (m, 4H), 3.73 (s, 3H). **^{19}F NMR** (376 MHz, $CDCl_3$) δ - 70.0 (s). **^{13}C NMR** (101 MHz, $CDCl_3$) δ 160.9, 156.3 (q, *J* = 33.3 Hz), 147.5, 130.6, 128.9, 125.0, 121.7, 120.3, 120.0 (q, *J* = 280.8 Hz), 113.9, 55.1. **IR** (KBr, cm^{-1}) ν: 3397, 3017, 2941, 1707, 1602, 1514, 1460, 1325, 1269, 1167, 1027, 941, 843, 768. **HRMS** (ESI) calcd for $C_{15}H_{13}F_3NO^+$ *m/z* 280.0944 $[M+H]^+$, Found 280.0951.

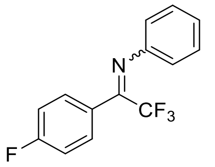

1x

2,2,2-trifluoro-1-(4-fluorophenyl)-*N*-phenylethan-1-imine 1x. Following general procedure A, on a 10 mmol scale. The product was purified by flash column chromatography on silica gel (height 18 cm, width 3.5 cm, eluent: petroleum ether + 3%

Et$_3$N) as a yellow oil, yield = 32% (0.85 g). R$_f$ (petroleum ether): 0.7. **^{1}H NMR** (400 MHz, CDCl$_3$) δ 7.27 – 7.15 (m, 4H), 7.04 (t, J = 7.6 Hz, 1H), 6.96 (t, J = 8.4 Hz, 2H), 6.72 (d, J = 7.6 Hz, 2H). **^{19}F NMR** (376 MHz, CDCl$_3$) δ - 70.4 (s), - 108.9 (s). **^{13}C NMR** (101 MHz, CDCl$_3$) δ 163.4 (d, J = 253.5 Hz), 155.9 (q, J = 34.3 Hz), 147.0, 131.0 (d, J = 8.1 Hz), 128.9, 126.0, 125.4, 120.3, 119.8 (q, J = 280.8 Hz), 115.8 (d, J = 22.2 Hz). **IR** (KBr, cm^{-1}) ν: 3452, 3072, 1664, 1600, 1504, 1330, 1236, 1195, 1135, 972, 838, 769, 696. **HRMS** (ESI) calcd for $C_{14}H_{10}F_4N^+$ *m/z* 268.0744 [M+H]$^+$, Found 268.0746.

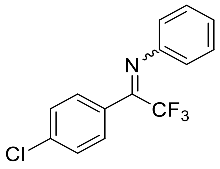

1y

1-(4-chlorophenyl)-2,2,2-trifluoro-*N*-phenylethan-1-imine 1y. Following general procedure A, on a 3 mmol scale. The product was purified by flash column chromatography on silica gel (height 18 cm, width 3.5 cm, eluent: petroleum ether + 3% Et$_3$N) as a yellow oil, yield = 31% (0.26 g). R$_f$ (petroleum ether/ethyl acetate = 91:9): 0.9. **^{1}H NMR** (400 MHz, CDCl$_3$) δ 7.29 – 7.10 (m, 6H), 7.05 (t, J = 7.6 Hz, 1H), 6.72 (d, J = 8.0 Hz, 2H). **^{19}F NMR** (376 MHz, CDCl$_3$) δ - 70.4 (s). **^{13}C NMR** (101 MHz, CDCl$_3$) δ 155.8 (q, J = 34.3 Hz), 146.8, 136.6, 130.1, 128.95, 128.92, 128.2, 125.6, 120.3, 119.7 (q, J = 279.8 Hz). **IR** (KBr, cm^{-1}) ν: 3410, 3073, 1662, 1592, 1488, 1329, 1232, 1195, 1136, 971, 832, 745, 693. **HRMS** (ESI) calcd for $C_{14}H_{10}ClF_3N^+$ *m/z* 284.0448 [M+H]$^+$, Found 284.0453.

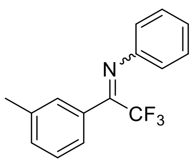

1z

2,2,2-trifluoro-*N*-phenyl-1-(m-tolyl)ethan-1-imine 1z. Following general procedure C, on a 15 mmol scale. The product was purified by flash column chromatography on silica gel (height 20 cm, width 3.5 cm, eluent: petroleum ether + 3% Et$_3$N) as an orange oil, overall yield = 8% (0.33 g). R$_f$ (petroleum ether): 0.7. **^{1}H NMR** (400 MHz, CDCl$_3$) δ 7.11 – 6.98 (m, 4H), 6.97 – 6.82 (m, 3H), 6.64 (d, J = 5.2 Hz, 2H), 2.12 (s, 3H). **^{19}F NMR** (376 MHz, CDCl$_3$) δ - 70.4 (s). **^{13}C NMR** (101 MHz, CDCl$_3$) δ 157.2 (q, J = 34.3 Hz), 147.2, 138.3, 131.0, 129.9, 128.9, 128.7, 128.3, 125.8, 125.2, 120.5, 119.9 (q, J = 280.8 Hz), 21.2. **IR** (KBr, cm^{-1}) ν: 3449, 3065, 2926, 1664, 1593, 1485, 1329, 1185, 1138, 1015, 984, 769, 696. **HRMS** (ESI) calcd for $C_{15}H_{13}F_3N^+$ *m/z* 264.0995 [M+H]$^+$, Found 264.0998.

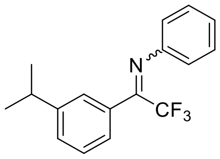

1aa

2,2,2-trifluoro-1-(3-isopropylphenyl)-*N*-phenylethan-1-imine 1aa. Following general procedure B, on a 10 mmol scale. The product was purified by flash column chromatography on silica gel (height 18 cm, width 3.5 cm, eluent: petroleum ether + 3% Et_3N) as an orange oil, overall yield = 69% (2.01 g). R_f (petroleum ether): 0.6. **^{1}H NMR** (400 MHz, $CDCl_3$) δ 7.26 – 7.08 (m, 5H), 7.01 (t, J = 7.6 Hz, 1H), 6.97 (s, 1H), 6.72 (d, J = 7.6 Hz, 2H), 2.80 – 2.68 (m, 1H), 1.06 (s, 3H), 1.04 (s, 3H). **^{19}F NMR** (376 MHz, $CDCl_3$) δ - 70.2 (s). **^{13}C NMR** (101 MHz, $CDCl_3$) δ 157.4 (q, J = 34.3 Hz), 149.1, 147.4, 129.8, 128.7, 128.5, 128.4, 127.2, 125.9, 125.1, 120.4, 119.9 (q, J = 280.8 Hz), 33.8, 23.5. **IR** (KBr, cm^{-1}) ν: 3409, 3069, 2964, 1660, 1593, 1479, 1328, 1231, 1185, 1135, 996, 899, 805, 696. **HRMS** (ESI) calcd for $C_{17}H_{17}F_3N^+$ *m/z* 292.1308 $[M+H]^+$, Found 292.1317.

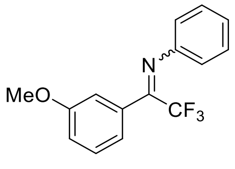

1ab

2,2,2-trifluoro-1-(3-methoxyphenyl)-*N*-phenylethan-1-imine 1ab. Following general procedure C, on a 15 mmol scale. The product was purified by flash column chromatography on silica gel (height 20 cm, width 3.5 cm, eluent: petroleum ether + 3% Et_3N) as a yellow oil, overall yield = 16% (0.69 g). R_f (petroleum ether): 0.3. **^{1}H NMR** (400 MHz, $CDCl_3$) δ 7.22 (t, J = 7.6 Hz, 3H), 7.06 (t, J = 7.6 Hz, 1H), 6.92 – 6.70 (m, 5H), 3.64 (s, 3H). **^{19}F NMR** (376 MHz, $CDCl_3$) δ - 70.3 (s). **^{13}C NMR** (101 MHz, $CDCl_3$) δ 159.3, 156.5 (q, J = 33.3 Hz), 147.1, 131.0, 129.6, 128.8, 125.3, 120.8, 120.3, 119.8 (q, J = 280.8 Hz), 116.1, 114.1, 55.0. **IR** (KBr, cm^{-1}) ν: 3474, 3071, 2954, 2841, 1666, 1592, 1483, 1330, 1256, 1136, 1044, 992, 842, 776, 696. **HRMS** (ESI) calcd for $C_{15}H_{13}F_3NO^+$ *m/z* 280.0944 $[M+H]^+$, Found 280.0950.

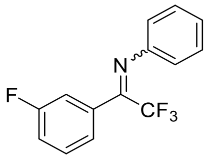

1ac

2,2,2-trifluoro-1-(3-fluorophenyl)-*N*-phenylethan-1-imine 1ac. Following general procedure C, on a 15 mmol scale, 2 equiv of aniline and 10 mol% of *p*-toluenesulfonic

acid monohydrate were used. The product was purified by flash column chromatography on silica gel (height 18 cm, width 3.5 cm, eluent: petroleum ether + 3% Et_3N) as an orange oil, overall yield = 41% (1.64 g). R_f (petroleum ether): 0.6. **^{1}H NMR** (400 MHz, $CDCl_3$) δ 7.28 – 7.15 (m, 3H), 7.09 – 6.91 (m, 4H), 6.74 (d, J = 7.2 Hz, 2H). **^{19}F NMR** (376 MHz, $CDCl_3$) δ - 70.6, - 111.5 (s) (s). **^{13}C NMR** (101 MHz, $CDCl_3$) δ 162.3 (d, J = 249.5 Hz), 155.5 (q, J = 34.3 Hz), 146.7, 131.8 (d, J = 8.1 Hz), 130.4 (d, J = 9.1 Hz), 128.9, 125.7, 124.6 (d, J = 3.0 Hz), 120.4, 119.6 (q, J = 280.8 Hz), 117.5 (d, J = 21.2 Hz), 115.9 (d, J = 23.2 Hz). **IR** (KBr, cm^{-1}) ν: 3441, 3074, 2926, 1668, 1588, 1487, 1440, 1332, 1251, 1138, 1000, 870, 778, 696. **HRMS** (ESI) calcd for $C_{14}H_{10}F_4N^+$ *m/z* 268.0744 $[M+H]^+$, Found 268.0754.

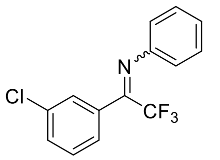

1ad

1-(3-chlorophenyl)-2,2,2-trifluoro-*N*-phenylethan-1-imine 1ad. Following general procedure C, on a 10 mmol scale, 2 equiv of aniline and 5 mol% of *p*-toluenesulfonic acid monohydrate were used. The product was purified by flash column chromatography on silica gel (height 20 cm, width 3.5 cm, eluent: petroleum ether + 3% Et_3N) as a yellow oil, overall yield = 7% (0.21 g). R_f (petroleum ether): 0.6. **^{1}H NMR** (400 MHz, $CDCl_3$) δ 7.29 (d, J = 8.0 Hz, 1H), 7.25 (s, 1H), 7.22 – 7.11 (m, 3H), 7.04 (t, J = 7.6 Hz, 2H), 6.73 (d, J = 8.0 Hz, 2H). **^{19}F NMR** (376 MHz, $CDCl_3$) δ - 70.5 (s). **^{13}C NMR** (101 MHz, $CDCl_3$) δ 155.3 (q, J = 34.3 Hz), 146.6, 134.7, 131.6, 130.4, 129.8, 128.9, 128.5, 126.9, 125.7, 120.4, 119.6 (q, J = 280.8 Hz). **IR** (KBr, cm^{-1}) ν: 3396, 3071, 2927, 1666, 1578, 1481, 1329, 1196, 1137, 988, 796, 692. **HRMS** (ESI) calcd for $C_{14}H_{10}ClF_3N^+$ *m/z* 284.0448 $[M+H]^+$, Found 284.0456.

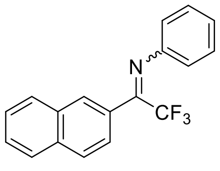

1ae

2,2,2-trifluoro-1-(naphthalen-2-yl)-*N*-phenylethan-1-imine 1ae. Following general procedure B, on a 10 mmol scale. The product was purified by flash column chromatography on silica gel (height 20 cm, width 3.5 cm, eluent: petroleum ether + 3% Et_3N) as a yellow oil, overall yield = 34% (1.01 g). R_f (petroleum ether): 0.5. **^{1}H NMR** (400 MHz, $CDCl_3$) δ 7.87 (s, 1H), 7.80 (d, J = 6.8 Hz, 2H), 7.72 (d, J = 8.4 Hz, 1H), 7.58 – 7.48 (m, 2H), 7.18 (t, J = 7.6 Hz, 3H), 7.03 (t, J = 7.6 Hz, 1H), 6.82 (d, J = 8.0 Hz, 2H). **^{19}F NMR** (376 MHz, $CDCl_3$) δ - 70.1 (s). **^{13}C NMR** (101 MHz, $CDCl_3$) δ

156.8 (q, J = 33.3 Hz), 147.1, 133.5, 132.5, 129.2, 128.9, 128.6, 128.3, 127.8, 127.7, 127.4, 126.8, 125.4, 125.1, 120.7, 120.0 (q, J = 279.8 Hz). **IR** (KBr, cm^{-1}) ν: 3292, 3064, 2928, 2859, 1948, 1659, 1588, 1491, 1320, 1128, 992, 819, 759. **HRMS** (MALDI) calcd for $C_{18}H_{13}F_3N^+$ *m/z* 300.0995 $[M+H]^+$, Found 300.0999.

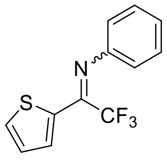

1af

2,2,2-trifluoro-*N*-phenyl-1-(thiophen-2-yl)ethan-1-imine 1af. Following general procedure A, on a 6 mmol scale, 3 equiv of aniline and 10 mol% of *p*-toluenesulfonic acid monohydrate were used. The product was purified by flash column chromatography on silica gel (height 20 cm, width 3.5 cm, eluent: petroleum ether + 3% Et_3N) as a yellow oil, yield = 16% (0.25 g). R_f (petroleum ether): 0.5. **^{1}H NMR** (400 MHz, $CDCl_3$) δ 7.57 – 7.52 (m, 1H), 7.48 – 7.35 (m, 3H), 7.21 (t, J = 7.6 Hz, 1H), 7.01 (dd, J = 5.2, 4.0 Hz, 1H), 6.82 (d, J = 7.2 Hz, 2H). **^{19}F NMR** (376 MHz, $CDCl_3$) δ – 68.6 (s). **^{13}C NMR** (101 MHz, $CDCl_3$) δ 148.8 (q, J = 34.3 Hz), 148.2, 133.6 (q, J = 3.0 Hz), 132.6, 129.7, 129.2, 126.6, 125.2, 119.8 (q, J = 280.8 Hz), 118.3. **IR** (KBr, cm^{-1}) ν: 3395, 3091, 1640, 1594, 1420, 1324, 1201, 1141, 1073, 919, 720. **HRMS** (ESI) calcd for $C_{12}H_9F_3NS^+$ *m/z* 256.0402 $[M+H]^+$, Found 256.0414.

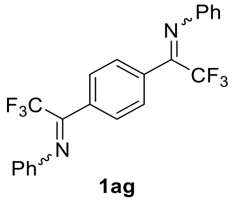

1ag

2,2,2-trifluoro-*N*-phenyl-1-(thiophen-2-yl)ethan-1-imine 1ag. Following procedure E, on a 2 mmol scale. The product was purified by flash column chromatography on silica gel (height 20 cm, width 3.5 cm, eluent: petroleum ether + 3% Et_3N) as a yellow solid, yield = 34% (0.29 g). R_f (petroleum ether): 0.5. **^{1}H NMR** (400 MHz, $CDCl_3$) δ 7.22 – 7.11 (m, 8H), 7.07 (t, J = 7.6 Hz, 2H), 6.64 (d, J = 7.6 Hz, 4H). **^{19}F NMR** (376 MHz, $CDCl_3$) δ – 70.5 (s). **^{13}C NMR** (101 MHz, $CDCl_3$) δ 155.8 (q, J = 34.3 Hz), 146.5, 131.8, 128.9, 128.8, 125.7, 120.5, 119.6 (q, J = 279.8 Hz). **IR** (KBr, cm^{-1}) ν: 3419, 3072, 2924, 1664, 1486, 1328, 1195, 1121, 967, 774, 703. **HRMS** (ESI) calcd for $C_{22}H_{15}F_6N_2^+$ *m/z* 421.1134 $[M+H]^+$, Found 421.1139.

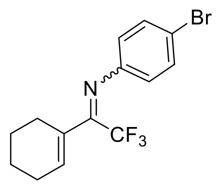

1ah

***N*-(4-bromophenyl)-1-(cyclohex-1-en-1-yl)-2,2,2-trifluoroethan-1-imine 1ah.** Following general procedure D, on a 14 mmol scale. The product was purified by flash column chromatography on silica gel (height 18 cm, width 3.5 cm, eluent: petroleum ether + 5% Et_3N) as a yellow oil, overall yield = 16% (0.74 g). R_f (petroleum ether): 0.6. **^{1}H NMR** (400 MHz, $CDCl_3$) δ 7.43 (d, J = 8.8 Hz, 2H), 6.77 (d, J = 8.4 Hz, 2H), 5.96 (s, 1H), 2.05 (s, 2H), 1.82 (s, 2H), 1.49 (s, 4H). **^{19}F NMR** (376 MHz, $CDCl_3$) δ – 71.3 (s). **^{13}C NMR** (101 MHz, $CDCl_3$) δ 159.8 (q, J = 33.3 Hz), 146.7, 135.0, 131.8, 129.3, 121.5, 119.7 (q, J = 280.8 Hz), 118.5, 26.3, 25.0, 21.7, 21.0. **IR** (KBr, cm^{-1}) ν: 3437, 2936, 2865, 1657, 1408, 1319, 1228, 1185, 1137, 1071, 982, 922, 832, 720. **HRMS** (ESI) calcd for $C_{14}H_{14}BrF_3N^+$ *m/z* 332.0256 $[M+H]^+$, Found 332.0261.

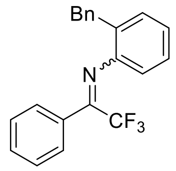

1ai

***N*-(2-benzylphenyl)-2,2,2-trifluoro-1-phenylethan-1-imine 1ai.** Following general procedure A, on a 10 mmol scale. The product was purified by flash column chromatography on silica gel (height 16 cm, width 4.5 cm, eluent: petroleum ether + 3% Et_3N) as a yellow oil, yield = 94% (3.18 g). R_f (petroleum ether): 0.2. **^{1}H NMR** (400 MHz, $CDCl_3$) δ 7.27 – 7.18 (m, 6H), 7.17 – 7.05 (m, 3H), 7.00 (t, J = 7.2 Hz, 1H), 6.88 (t, J = 7.6 Hz, 1H), 6.65 (d, J = 7.2 Hz, 2H), 6.24 (d, J = 7.6 Hz, 1H), 4.01 (s, 2H). **^{19}F NMR** (376 MHz, $CDCl_3$) δ – 70.3 (s). **^{13}C NMR** (101 MHz, $CDCl_3$) δ 156.3 (q, J = 34.3 Hz), 145.6, 140.1, 133.5, 130.1, 130.0, 129.8, 129.3, 128.4, 128.2, 128.1, 126.6, 126.1, 125.7, 119.9 (q, J = 279.8 Hz), 119.1, 38.5. **IR** (KBr, cm^{-1}) ν: 3442, 3032, 2922, 1907, 1660, 1592, 1486, 1442, 1327, 1193, 1139, 964, 737, 695, 612. **HRMS** (ESI) calcd for $C_{21}H_{17}F_3N^+$ *m/z* 340.1308 $[M+H]^+$, Found 340.1310.

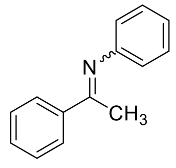

1aj

***N*,1-diphenylethan-1-imine 1aj**. Following general procedure A, on a 10 mmol scale. The product was purified by flash column chromatography on silica gel (height 20 cm, width 3.5 cm, eluent: petroleum ether + 3% Et_3N) as a yellow solid, yield = 50% (0.98 g). R_f (petroleum ether/ethyl acetate = 98:2): 0.3. **^{1}H NMR** (400 MHz, $CDCl_3$) δ 8.00 (d, *J* = 5.6 Hz, 2H), 7.58 – 7.42 (m, 3H), 7.37 (t, *J* = 7.6 Hz, 2H), 7.11 (t, *J* = 7.2 Hz, 1H), 6.82 (d, *J* = 7.6 Hz, 2H), 2.25 (s, 3H). **^{13}C NMR** (101 MHz, $CDCl_3$) δ 165.4, 151.7, 139.5, 130.4, 128.9, 128.3, 127.1, 123.2, 119.3, 17.3. **IR** (KBr, cm^{-1}) ν: 3458, 3370, 3036, 2925, 1682, 1610, 1496, 1444, 1362, 1269, 1175, 1077, 1026, 959, 757, 691. **HRMS** (ESI) calcd for $C_{14}H_{14}N^+$ *m/z* 196.1121 $[M+H]^+$, Found 196.1127.

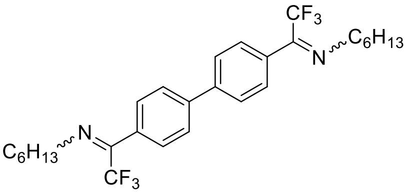

1ak

1,1'-([1,1'-biphenyl]-4,4'-diyl)bis(2,2,2-trifluoro-*N*-hexylethan-1-imine) 1ak. Following general procedure F, on a 3 mmol scale. The product was purified by flash column chromatography on silica gel (height 20 cm, width 3.5 cm, eluent: petroleum ether/ethyl acetate, gradient: 100:0 to 98:2 + 3% Et_3N) as a colorless oil, yield = 61% (0.94 g). R_f (petroleum ether/ethyl acetate = 98:2): 0.2. **^{1}H NMR** (400 MHz, $CDCl_3$) *δ* 7.71 (d, *J* = 8.0 Hz, 4H), 7.35 (d, *J* = 8.4 Hz, 4H), 3.44 (t, *J* = 6.4 Hz, 4H), 1.73 – 1.63 (m, 4H), 1.34 – 1.19 (m, 12H), 0.86 (t, *J* = 6.4 Hz, 6H). **^{19}F NMR** (376 MHz, $CDCl_3$) *δ* – 71.4 (s). **^{13}C NMR** (101 MHz, $CDCl_3$) *δ* 157.5 (q, *J* = 34.3 Hz), 141.6, 130.0, 128.4, 127.5, 119.7 (q, *J* = 279.8 Hz), 53.5, 31.4, 30.2, 26.9, 22.5, 14.0. **IR** (KBr, cm^{-1}) ν: 3314, 3040, 2931, 2862, 1919, 1666, 1609, 1460, 1335, 1198, 1124, 1005, 956, 825, 736, 684. **HRMS** (ESI) calcd for $C_{28}H_{35}F_6N_2^+$ *m/z* 513.2699 $[M+H]^+$, Found 513.2703.

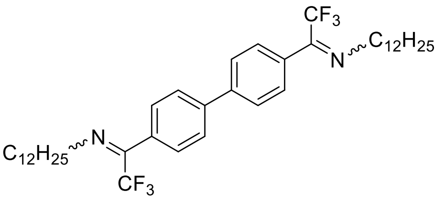

1al

1,1'-([1,1'-biphenyl]-4,4'-diyl)bis(*N*-dodecyl-2,2,2-trifluoroethan-1-imine) 1al. Following general procedure F, on a 3 mmol scale. The product was purified by flash column chromatography on silica gel (height 20 cm, width 3.5 cm, eluent: petroleum ether/ethyl acetate, gradient: 100:0 to 99:1 + 3% Et_3N) as a white solid, yield = 83% (1.69 g). R_f (petroleum ether/ethyl acetate = 98:2): 0.3. **^{1}H NMR** (400 MHz, $CDCl_3$) *δ* 7.71 (d, *J* = 8.4 Hz, 4H), 7.35 (d, *J* = 8.4 Hz, 4H), 3.49 – 3.39 (m, 4H), 1.73 – 1.63 (m,

4H), 1.30 – 1.19 (m, 36H), 0.87 (t, *J* = 6.8 Hz, 6H). **^{19}F NMR** (376 MHz, $CDCl_3$) *δ* – 71.4 (s). **^{13}C NMR** (101 MHz, $CDCl_3$) *δ* 157.5 (q, *J* = 34.3 Hz), 141.6, 130.0, 128.4, 127.5, 119.7 (q, *J* = 279.8 Hz), 53.5, 31.9, 30.2, 29.62, 29.61, 29.57, 29.5, 29.32, 29.27, 27.3, 22.7, 14.1. **IR** (KBr, cm^{-1}) ν: 3434, 2923, 2853, 1669, 1467, 1330, 1195, 1123, 1041, 995, 819, 732, 679. **HRMS** (ESI) calcd for $C_{40}H_{59}F_6N_2^+$ *m/z* 681.4577 $[M+H]^+$, Found 681.4581.

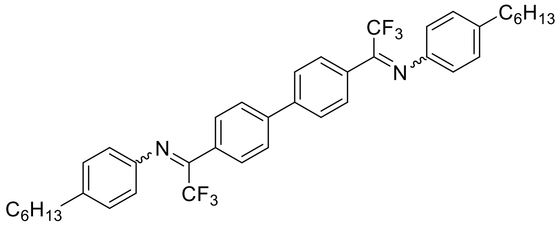

1am

1,1'-([1,1'-biphenyl]-4,4'-diyl)bis(2,2,2-trifluoro-*N*-(4-hexylphenyl)ethan-1-imine) 1am. Following general procedure F, on a 3 mmol scale. The product was purified by flash column chromatography on silica gel (height 20 cm, width 3.5 cm, eluent: petroleum ether + 3% Et_3N) as an orange solid, yield = 92% (1.83 g). R*f* (petroleum ether/ethyl acetate = 98:2): 0.4. **^{1}H NMR** (400 MHz, $CDCl_3$) *δ* 7.49 (d, *J* = 8.4 Hz, 4H), 7.31 (d, *J* = 8.0 Hz, 4H), 7.01 (d, *J* = 8.0 Hz, 4H), 6.70 (d, *J* = 8.4 Hz, 4H), 2.51 (t, *J* = 7.6 Hz, 4H), 1.61 – 1.48 (m, 4H), 1.32 – 1.21 (m, 12H), 0.85 (t, *J* = 6.8 Hz, 6H). **^{19}F NMR** (376 MHz, $CDCl_3$) *δ* – 70.2 (s). **^{13}C NMR** (101 MHz, $CDCl_3$) *δ* 155.7 (q, *J* = 34.3 Hz), 144.5, 141.2, 140.5, 129.8, 129.4, 128.8, 127.1, 120.8, 119.9 (q, *J* = 279.8 Hz), 35.3, 31.6, 31.1, 28.8, 22.6, 14.0. **IR** (KBr, cm^{-1}) ν: 3431, 3038, 2928, 2860, 1670, 1502, 1331, 1237, 1196, 1136, 1022, 969, 831, 736. **HRMS** (ESI) calcd for $C_{40}H_{43}F_6N_2^+$ *m/z* 665.3325 $[M+H]^+$, Found 665.3331.

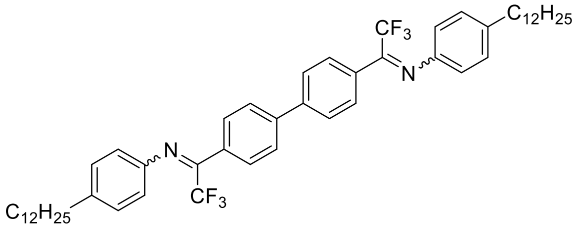

1an

1,1'-([1,1'-biphenyl]-4,4'-diyl)bis((4-dodecylphenyl)-2,2,2-trifluoroethan-1-imine) 1an. Following general procedure F, on a 3 mmol scale. The product was purified by flash column chromatography on silica gel (height 20 cm, width 3.5 cm, eluent: petroleum ether + 3% Et_3N) as an orange oil, yield = 95% (2.37 g). R*f* (petroleum ether/ethyl acetate = 98:2): 0.5. **^{1}H NMR** (400 MHz, $CDCl_3$) *δ* 7.50 (d, *J* = 8.0 Hz, 4H), 7.31 (d, *J* = 8.4 Hz, 4H), 7.02 (d, *J* = 8.4 Hz, 4H), 6.71 (d, *J* = 8.0 Hz, 4H), 2.52

(t, J = 8.0 Hz, 4H), 1.61 – 1.50 (m, 4H), 1.31 – 1.21 (m, 36H), 0.89 (t, J = 6.8 Hz, 6H). **^{19}F NMR** (376 MHz, $CDCl_3$) δ – 70.2 (s). **^{13}C NMR** (101 MHz, $CDCl_3$) δ 155.6 (q, J = 34.3 Hz), 144.5, 141.2, 140.5, 129.8, 129.4, 128.8, 127.1, 120.9, 119.9 (q, J = 279.8 Hz), 35.3, 31.9, 31.2, 29.7, 29.63, 29.59, 29.4, 29.3, 29.2, 22.7, 14.1. one carbon was overlapped. **IR** (KBr, cm^{-1}) ν: 3289, 3034, 2927, 1906, 1790, 1658, 1607, 1501, 1456, 1328, 1192, 1022, 967, 909, 830, 734, 620. **HRMS** (ESI) calcd for $C_{52}H_{67}F_6N_2^+$ *m/z* 833.5203 $[M+H]^+$, Found 833.5207.

General procedure G: synthesis of derivatives 3a-3be (Scheme S7)

An oven-dried 25 mL schlenk tube equipped with a stirring bar was transferred into a glovebox (through standard glovebox operation), where $Re_2(CO)_{10}$ (19.6 mg, 0.03 mmol, 0.1 equiv) was added. The tube was then removed from the glovebox and placed under Ar. Then the ketimine **1** (0.3 mmol, 1 equiv), isocyanate **2** (0.6 mmol, 2 equiv), and o-xylene (or PhCl) (3 mL) were added subsequently to the test tube under Ar. The resulting reaction mixture was then stirred at 150 °C (or indicated temperature) for 60 h (or indicated time). After reaction completed (by TLC monitoring), the mixture was cooled down to room temperature and concentrated under vacuum. The residue was then purified by flash column chromatography on silica gel to give the desired product **3a-3be**. Note that, in the case of solid α-CF_3 ketimine, these were added in the tube before the solvent.

Purification and characterization of derivatives 3a-3be

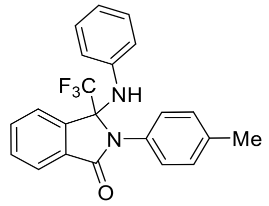

3a

3-(phenylamino)-2-(p-tolyl)-3-(trifluoromethyl)isoindolin-1-one 3a. Starting from **1a** and *p*-tolyl isocyanate (Cas: 622-58-2), o-xylene as solvent, 60 h. The product was purified by flash column chromatography on silica gel (height 16 cm, width 1.5 cm, eluent: petroleum ether/ethyl acetate, gradient: 99:1 to 95:5) as a white solid, yield = 82% (93.6 mg). R_f (petroleum ether/ethyl acetate = 91:9): 0.3. **^{1}H NMR** (400 MHz, $CDCl_3$) δ 8.06 (d, J = 7.2 Hz, 1H), 7.71 – 7.56 (m, 3H), 7.20 (d, J = 8.0 Hz, 2H), 7.06 (t, J = 8.0 Hz, 4H), 6.83 (t, J = 7.6 Hz, 1H), 6.34 (d, J = 7.2 Hz, 2H), 4.87 (s, 1H), 2.37 (s, 3H). **^{19}F NMR** (376 MHz, $CDCl_3$) δ -77.5 (s). **^{13}C NMR** (101 MHz, $CDCl_3$) δ 167.4, 141.7, 138.5, 138.3, 132.9, 132.8, 131.3, 131.1, 129.9, 129.3, 128.8, 124.7, 124.6, 123.3

(q, J = 288.9 Hz), 120.7, 116.4, 80.7 (q, J = 29.3 Hz), 21.1. **IR** (KBr, cm^{-1}) ν: 3356, 3058, 2924, 1919, 1705, 1606, 1507, 1361, 1257, 1180, 1065, 975, 812, 720. **HRMS** (ESI) calcd for $C_{22}H_{18}F_3N_2O^+$ *m/z* 383.1366 $[M+H]^+$, Found 383.1373.

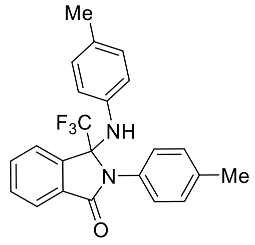

3b

2-(p-tolyl)-3-(p-tolylamino)-3-(trifluoromethyl)isoindolin-1-one 3b. Starting from **1b** and *p*-tolyl isocyanate (Cas: 622-58-2), PhCl as solvent, 48 h. The product was purified by flash column chromatography on silica gel (height 16 cm, width 1.5 cm, eluent: petroleum ether/ethyl acetate, gradient: 99:1 to 97:3) as a white solid, yield = 81% (95.8 mg). R_f (petroleum ether/ethyl acetate = 91:9): 0.3. **^{1}H NMR** (400 MHz, $CDCl_3$) δ 8.05 (d, J = 7.2 Hz, 1H), 7.70 – 7.55 (m, 3H), 7.19 (d, J = 7.6 Hz, 2H), 7.06 (d, J = 6.8 Hz, 2H), 6.87 (d, J = 8.0 Hz, 2H), 6.25 (d, J = 8.0 Hz, 2H), 4.71 (s, 1H), 2.37 (s, 3H), 2.20 (s, 3H). **^{19}F NMR** (376 MHz, $CDCl_3$) δ -77.5 (s). **^{13}C NMR** (101 MHz, $CDCl_3$) δ 167.4, 139.1, 138.5, 138.4, 132.9, 132.8, 131.4, 131.1, 130.2, 129.9, 129.8, 128.8, 124.7, 124.6, 123.4 (q, J = 288.9 Hz), 116.6, 80.9 (q, J = 29.3 Hz), 21.1, 20.3. **IR** (KBr, cm^{-1}) ν: 3322, 3070, 2923, 1700, 1614, 1517, 1465, 1370, 1261, 1180, 1053, 903, 813, 724. **HRMS** (ESI) calcd for $C_{23}H_{20}F_3N_2O^+$ *m/z* 397.1522 $[M+H]^+$, Found 397.1526.

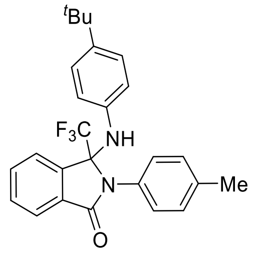

3c

3-((4-(tert-butyl)phenyl)amino)-2-(p-tolyl)-3-(trifluoromethyl)isoindolin-1-one 3c. Starting from **1c** and *p*-tolyl isocyanate (Cas: 622-58-2), o-xylene as solvent, 60 h. The product was purified by flash column chromatography on silica gel (height 16 cm, width 1.5 cm, eluent: petroleum ether/ethyl acetate, gradient: 99:1 to 97:3) as a white solid, yield = 70% (92.7 mg). R_f (petroleum ether/ethyl acetate = 91:9): 0.4. **^{1}H NMR** (400 MHz, $CDCl_3$) δ 8.06 (d, J = 7.2 Hz, 1H), 7.73 – 7.55 (m, 3H), 7.19 (d, J = 8.0 Hz, 2H), 7.12 – 6.97 (m, 4H), 6.25 (d, J = 8.8 Hz, 2H), 4.75 (s, 1H), 2.37 (s, 3H), 1.23 (s,

9H). **¹⁹F NMR** (376 MHz, $CDCl_3$) δ -77.5 (s). **¹³C NMR** (101 MHz, $CDCl_3$) δ 167.5, 143.5, 139.0, 138.5, 138.4, 132.8, 132.8, 131.4, 131.1, 129.9, 128.8, 126.1, 124.8, 124.6, 123.4 (q, J = 288.9 Hz), 116.1, 80.8 (q, J = 29.3 Hz), 33.9, 31.3, 21.1. **IR** (KBr, cm^{-1}) ν: 3338, 3044, 2961, 1712, 1614, 1522, 1468, 1365, 1263, 1185, 1053, 973, 820, 727. **HRMS** (ESI) calcd for $C_{26}H_{26}F_3N_2O^+$ *m/z* 439.1992 $[M+H]^+$, Found 439.1997.

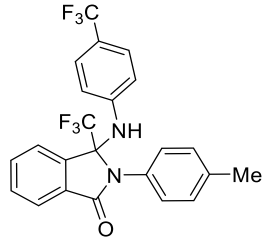

3d

2-(p-tolyl)-3-(trifluoromethyl)-3-((4-(trifluoromethyl)phenyl)amino)isoindolin-1-one 3d. Starting from **1d** and *p*-tolyl isocyanate (Cas: 622-58-2), o-xylene as solvent, 160 °C, 60 h. The product was purified by flash column chromatography on silica gel (height 16 cm, width 1.5 cm, eluent: petroleum ether/ethyl acetate, gradient: 99:1 to 95:5) as a white solid, yield = 65% (87.6 mg). R_f (petroleum ether/ethyl acetate = 91:9): 0.3. **¹H NMR** (400 MHz, $CDCl_3$) δ 8.05 (d, J = 7.6 Hz, 1H), 7.77 – 7.52 (m, 3H), 7.30 (d, J = 8.4 Hz, 2H), 7.20 (d, J = 8.0 Hz, 2H), 7.00 (d, J = 8.0 Hz, 2H), 6.36 (d, J = 8.4 Hz, 2H), 5.19 (s, 1H), 2.36 (s, 3H). **¹⁹F NMR** (376 MHz, $CDCl_3$) δ -62.3 (s), -77.5 (s). **¹³C NMR** (101 MHz, $CDCl_3$) δ 167.3, 144.7, 138.9, 137.7, 133.2, 132.7, 131.5, 131.1, 130.1, 128.8, 126.6 (q, J = 3.0 Hz), 124.9, 124.6, 124.2 (q, J = 272.7 Hz), 123.2 (q, J = 289.9 Hz), 122.6 (q, J = 33.3 Hz), 115.7, 80.3 (q, J = 29.3 Hz), 21.1. **IR** (KBr, cm^{-1}) ν: 3327, 3069, 2928, 1702, 1619, 1518, 1369, 1326, 1267, 1181, 1127, 1064, 843, 724. **HRMS** (ESI) calcd for $C_{23}H_{17}F_6N_2O^+$ *m/z* 451.1240 $[M+H]^+$, Found 451.1240.

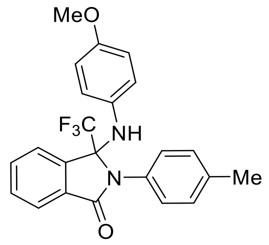

3e

3-((4-methoxyphenyl)amino)-2-(p-tolyl)-3-(trifluoromethyl)isoindolin-1-one 3e. Starting from **1e** and *p*-tolyl isocyanate (Cas: 622-58-2), o-xylene as solvent, 60 h. The product was purified by flash column chromatography on silica gel (height 16 cm, width 1.5 cm, eluent: petroleum ether/ethyl acetate, gradient: 99:1 to 95:5) as a white solid, yield = 75% (92.2 mg). R_f (petroleum ether/ethyl acetate = 91:9): 0.3. **¹H NMR** (400 MHz, $CDCl_3$) δ 8.03 (d, J = 7.6 Hz, 1H), 7.71 – 7.54 (m, 3H), 7.20 (d, J = 8.0 Hz,

2H), 7.06 (d, J = 8.0 Hz, 2H), 6.62 (d, J = 8.8 Hz, 2H), 6.32 (d, J = 8.8 Hz, 2H), 4.59 (s, 1H), 3.68 (s, 3H), 2.37 (s, 3H). **^{19}F NMR** (376 MHz, $CDCl_3$) δ -77.5 (s). **^{13}C NMR** (101 MHz, $CDCl_3$) δ 167.4, 154.2, 138.6, 138.3, 135.0, 133.0, 132.8, 131.4, 131.1, 130.0, 128.8, 124.8, 124.6, 123.4 (q, J = 289.9 Hz), 118.4, 114.6, 80.8 (q, J = 29.3 Hz), 55.4, 21.2. **IR** (KBr, cm^{-1}) ν: 3357, 3069, 2947, 1707, 1513, 1466, 1369, 1254, 1180, 1045, 825, 721. **HRMS** (ESI) calcd for $C_{23}H_{20}F_3N_2O_2^+$ *m/z* 413.1471 [M+H]$^+$, Found 413.1478.

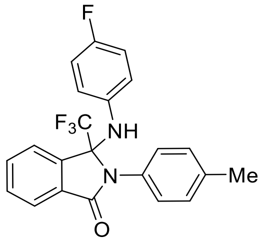

3f

3-((4-fluorophenyl)amino)-2-(p-tolyl)-3-(trifluoromethyl)isoindolin-1-one 3f. Starting from **1f** and *p*-tolyl isocyanate (Cas: 622-58-2), PhCl as solvent, 48 h. The product was purified by flash column chromatography on silica gel (height 16 cm, width 1.5 cm, eluent: petroleum ether/ethyl acetate, gradient: 99:1 to 96:4) as a white solid, yield = 81% (97.7 mg). R$_f$ (petroleum ether/ethyl acetate = 91:9): 0.3. **^{1}H NMR** (400 MHz, $CDCl_3$) δ 8.03 (d, J = 7.2 Hz, 1H), 7.73 – 7.51 (m, 3H), 7.20 (d, J = 8.0 Hz, 2H), 7.04 (d, J = 8.0 Hz, 2H), 6.90 – 6.67 (m, 2H), 6.39 – 6.17 (m, 2H), 4.81 (s, 1H), 2.37 (s, 3H). **^{19}F NMR** (376 MHz, $CDCl_3$) δ -77.5 (s), -123.4 (s). **^{13}C NMR** (101 MHz, $CDCl_3$) δ 167.4, 157.4 (d, J = 241.4 Hz), 156.2, 138.7 (d, J = 2.0 Hz), 138.0, 137.7, 132.9, 132.9, 131.3, 131.2, 130.0, 128.7, 124.7, 123.3 (q, J = 289.9 Hz), 117.9 (d, J = 8.1 Hz), 115.4 (d, J = 22.2 Hz), 80.8 (q, J = 29.3 Hz), 21.1. **IR** (KBr, cm^{-1}) ν: 3358, 3061, 2929, 1705, 1613, 1513, 1362, 1259, 1224, 1179, 1055, 977, 824, 773, 723. **HRMS** (ESI) calcd for $C_{22}H_{17}F_4N_2O^+$ *m/z* 401.1272 [M+H]$^+$, Found 401.1275.

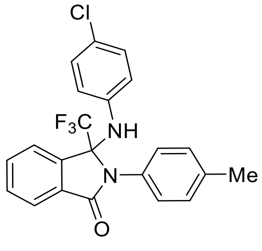

3g

3-((4-chlorophenyl)amino)-2-(p-tolyl)-3-(trifluoromethyl)isoindolin-1-one 3g. Starting from **1g** and *p*-tolyl isocyanate (Cas: 622-58-2), PhCl as solvent, 48 h. The product was purified by flash column chromatography on silica gel (height 16 cm, width 1.5 cm, eluent: petroleum ether/ethyl acetate, gradient: 99:1 to 95:5) as a white

solid, yield = 82% (102.9 mg). R_f (petroleum ether/ethyl acetate = 91:9): 0.3. **^{1}H NMR** (400 MHz, $CDCl_3$) δ 8.02 (d, J = 6.8 Hz, 1H), 7.73 – 7.49 (m, 3H), 7.20 (d, J = 8.0 Hz, 2H), 7.02 (dd, J = 12.0, 8.4 Hz, 4H), 6.26 (d, J = 8.4 Hz, 2H), 5.01 (s, 1H), 2.37 (s, 3H). **^{19}F NMR** (376 MHz, $CDCl_3$) δ -77.4 (s). **^{13}C NMR** (101 MHz, $CDCl_3$) δ 167.3, 140.3, 138.7, 137.9, 133.0, 132.7, 131.3, 131.2, 130.0, 129.2, 128.7, 125.7, 124.7, 124.6, 123.1 (q, J = 289.9 Hz), 117.6, 80.6 (q, J = 30.3 Hz), 21.1. **IR** (KBr, cm^{-1}) ν: 3353, 3043, 2925, 1706, 1606, 1503, 1361, 1259, 1182, 1058, 976, 815, 725. **HRMS** (ESI) calcd for $C_{22}H_{17}ClF_3N_2O^+$ *m/z* 417.0976 $[M+H]^+$, Found 417.0980.

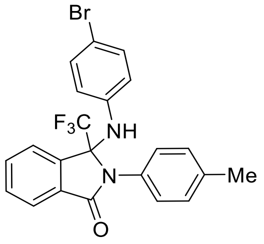

3h

3-((4-bromophenyl)amino)-2-(p-tolyl)-3-(trifluoromethyl)isoindolin-1-one 3h. Starting from **1h** and *p*-tolyl isocyanate (Cas: 622-58-2), o-xylene as solvent, 60 h. The product was purified by flash column chromatography on silica gel (height 16 cm, width 1.5 cm, eluent: petroleum ether/ethyl acetate, gradient: 99:1 to 95:5) as a white solid, yield = 66% (90.8 mg). R_f (petroleum ether/ethyl acetate = 91:9): 0.3. **^{1}H NMR** (400 MHz, $CDCl_3$) δ 8.03 (d, J = 7.2 Hz, 1H), 7.74 – 7.52 (m, 3H), 7.17 (dd, J = 20.4, 8.8 Hz, 4H), 7.02 (d, J = 8.4 Hz, 2H), 6.21 (d, J = 8.8 Hz, 2H), 4.92 (s, 1H), 2.37 (s, 3H). **^{19}F NMR** (376 MHz, $CDCl_3$) δ -76.9 (s). **^{13}C NMR** (101 MHz, $CDCl_3$) δ 167.3, 140.8, 138.7, 137.8, 133.0, 132.7, 132.2, 131.3, 131.2, 130.0, 128.8, 124.8, 124.6, 123.2 (q, J = 289.9 Hz), 118.0, 113.1, 80.5 (q, J = 29.3 Hz), 21.1. **IR** (KBr, cm^{-1}) ν: 3330, 3046, 2926, 1702, 1599, 1501, 1366, 1261, 1180, 1065, 971, 817, 723. **HRMS** (ESI) calcd for $C_{22}H_{17}BrF_3N_2O^+$ *m/z* 463.0450 $[M+H]^+$, Found 463.0457.

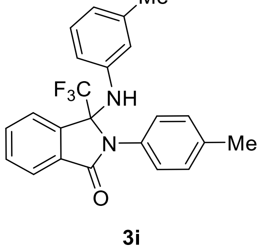

3i

2-(p-tolyl)-3-(m-tolylamino)-3-(trifluoromethyl)isoindolin-1-one 3i. Starting from **1i** and *p*-tolyl isocyanate (Cas: 622-58-2), PhCl as solvent, 48 h. The product was purified by flash column chromatography on silica gel (height 16 cm, width 1.5 cm, eluent: petroleum ether/ethyl acetate, gradient: 99:1 to 95:5) as a white solid, yield =

94% (111.3 mg). R_f (petroleum ether/ethyl acetate = 91:9): 0.3. **^{1}H NMR** (400 MHz, $CDCl_3$) δ 8.06 (d, J = 7.6 Hz, 1H), 7.71 – 7.59 (m, 3H), 7.20 (d, J = 8.4 Hz, 2H), 7.08 (d, J = 8.0 Hz, 2H), 6.92 (t, J = 8.0 Hz, 1H), 6.66 (d, J = 7.2 Hz, 1H), 6.24 (s, 1H), 6.06 (d, J = 8.0 Hz, 1H), 4.81 (s, 1H), 2.38 (s, 3H), 2.15 (s, 3H). **^{19}F NMR** (376 MHz, $CDCl_3$) δ -77.6 (s). **^{13}C NMR** (101 MHz, $CDCl_3$) δ 167.4, 141.6, 139.1, 138.5, 138.4, 138.3, 132.8, 131.4, 131.0, 129.9, 129.1, 128.8, 124.7, 124.5, 123.3 (q, J = 289.9 Hz), 121.6, 117.4, 113.2, 80.7 (q, J = 29.3 Hz), 21.4, 21.1. **IR** (KBr, cm^{-1}) ν: 3375, 2975, 2889, 1706, 1373, 1267, 1186, 1050, 881, 720. **HRMS** (ESI) calcd for $C_{23}H_{20}F_3N_2O^+$ *m/z* 397.1522 $[M+H]^+$, Found 397.1527.

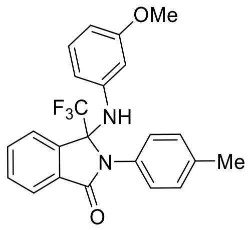

3j

3-((3-methoxyphenyl)amino)-2-(p-tolyl)-3-(trifluoromethyl)isoindolin-1-one 3j. Starting from **1j** and *p*-tolyl isocyanate (Cas: 622-58-2), o-xylene as solvent, 60 h. The product was purified by flash column chromatography on silica gel (height 20 cm, width 1.5 cm, eluent: petroleum ether/ethyl acetate, gradient: 99:1 to 97:3) as a white solid, yield = 73% (90.5 mg). R_f (petroleum ether/ethyl acetate = 91:9): 0.2. **^{1}H NMR** (400 MHz, $CDCl_3$) δ 8.05 (d, J = 6.8 Hz, 1H), 7.72 – 7.59 (m, 3H), 7.20 (d, J = 8.4 Hz, 2H), 7.05 (d, J = 8.0 Hz, 2H), 6.96 (t, J = 8.0 Hz, 1H), 6.38 (dd, J = 8.4, 2.0 Hz, 1H), 5.94 (dd, J = 8.4, 2.0 Hz, 1H), 5.84 (t, J = 2.0 Hz, 1H), 4.82 (s, 1H), 3.53 (s, 3H), 2.37 (s, 3H). **^{19}F NMR** (376 MHz, $CDCl_3$) δ -77.5 (s). **^{13}C NMR** (101 MHz, $CDCl_3$) δ 167.3, 160.4, 142.9, 138.6, 138.4, 133.0, 132.7, 131.3, 131.2, 130.1, 130.0, 128.8, 124.7, 124.6, 123.3 (q, J = 289.9 Hz), 108.9, 106.5, 102.1, 80.6 (q, J = 29.3 Hz), 54.8, 21.1. **IR** (KBr, cm^{-1}) ν: 3347, 3047, 2946, 1707, 1609, 1507, 1357, 1268, 1175, 1052, 722. **HRMS** (ESI) calcd for $C_{23}H_{20}F_3N_2O_2^+$ *m/z* 413.1471 $[M+H]^+$, Found 413.1474.

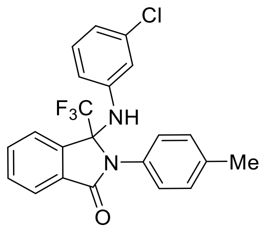

3k

3-((3-chlorophenyl)amino)-2-(p-tolyl)-3-(trifluoromethyl)isoindolin-1-one 3k. Starting from **1k** and *p*-tolyl isocyanate (Cas: 622-58-2), PhCl as solvent, 48 h. The

product was purified by flash column chromatography on silica gel (height 16 cm, width 1.5 cm, eluent: petroleum ether/ethyl acetate, gradient: 99:1 to 97:3) as a white solid, yield = 73% (91.8 mg). R*f* (petroleum ether/ethyl acetate = 91:9): 0.3. **^{1}H NMR** (400 MHz, $CDCl_3$) δ 8.05 (d, J = 7.2 Hz, 1H), 7.76 – 7.54 (m, 3H), 7.20 (d, J = 8.0 Hz, 2H), 7.02 (d, J = 8.0 Hz, 2H), 6.93 (t, J = 8.0 Hz, 1H), 6.80 (d, J = 8.0 Hz, 1H), 6.41 (s, 1H), 6.10 (dd, J = 8.0, 2.0 Hz, 1H), 4.90 (s, 1H), 2.37 (s, 3H). **^{19}F NMR** (376 MHz, $CDCl_3$) δ -77.5 (s). **^{13}C NMR** (101 MHz, $CDCl_3$) δ 167.3, 142.9, 138.8, 137.8, 135.0, 133.0, 132.7, 131.4, 131.2, 130.3, 130.0, 128.8, 124.8, 124.6, 123.2 (q, J = 288.9 Hz), 120.9, 116.7, 114.1, 80.4 (q, J = 29.3 Hz), 21.2. **IR** (KBr, cm^{-1}) ν: 3314, 3068, 1702, 1598, 1475, 1373, 1261, 1184, 1058, 729. **HRMS** (ESI) calcd for $C_{22}H_{17}ClF_3N_2O^+$ *m/z* 417.0976 $[M+H]^+$, Found 417.0981.

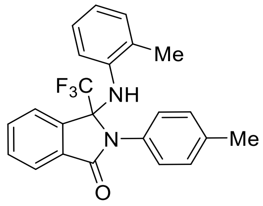

3l

2-(p-tolyl)-3-(o-tolylamino)-3-(trifluoromethyl)isoindolin-1-one 3l. Starting from **1l** and *p*-tolyl isocyanate (Cas: 622-58-2), o-xylene as solvent, 60 h. The product was purified by flash column chromatography on silica gel (height 16 cm, width 1.5 cm, eluent: petroleum ether/ethyl acetate, gradient: 99:1 to 98:2) as a white solid, yield = 54% (64.5 mg). R*f* (petroleum ether/ethyl acetate = 91:9): 0.4. **^{1}H NMR** (400 MHz, $CDCl_3$) δ 8.07 (dd, J = 7.6, 2.0 Hz, 1H), 7.72 – 7.51 (m, 3H), 7.22 – 7.07 (m, 3H), 7.04 – 6.93 (m, 2H), 6.84 – 6.70 (t, J = 7.6 Hz, 2H), 5.99 – 5.85 (m, 1H), 4.52 (s, 1H), 2.37 (s, 3H), 2.22 (s, 3H). **^{19}F NMR** (376 MHz, $CDCl_3$) δ -77.7 (s). **^{13}C NMR** (101 MHz, $CDCl_3$) δ 167.4, 139.8, 138.5, 138.3, 132.87, 132.85, 131.3, 131.1, 130.6, 130.0, 128.6, 127.0, 125.1, 124.7, 124.6, 123.5 (q, J = 289.9 Hz), 120.8, 115.0, 80.8 (q, J = 29.3 Hz), 21.2, 17.5. **IR** (KBr, cm^{-1}) ν: 3343, 3029, 2925, 1706, 1606, 1520, 1469, 1360, 1260, 1183, 1052, 975, 808, 718. **HRMS** (ESI) calcd for $C_{23}H_{20}F_3N_2O^+$ *m/z* 397.1522 $[M+H]^+$, Found 397.1526.

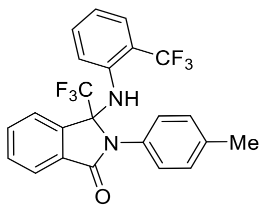

3m

2-(p-tolyl)-3-(trifluoromethyl)-3-((2-(trifluoromethyl)phenyl)amino)isoindolin-1-one 3m. Starting from **1m** and *p*-tolyl isocyanate (Cas: 622-58-2), PhCl as solvent, 48

h. The product was purified by flash column chromatography on silica gel (height 16 cm, width 1.5 cm, eluent: petroleum ether/ethyl acetate, gradient: 99:1 to 97:3) as a white solid, yield = 22% (29.9 mg). R*f* (petroleum ether/ethyl acetate = 91:9): 0.2. **^{1}H NMR** (400 MHz, $CDCl_3$) δ 8.06 (d, *J* = 7.6 Hz, 1H), 7.69 – 7.47 (m, 4H), 7.21 (d, *J* = 8.4 Hz, 2H), 7.08 (t, *J* = 8.4 Hz, 3H), 6.91 (t, *J* = 7.6 Hz, 1H), 6.20 (d, *J* = 8.4 Hz, 1H), 5.30 (s, 1H), 2.37 (s, 3H). **^{19}F NMR** (376 MHz, $CDCl_3$) δ -62.4 (s), -78.0 (s). **^{13}C NMR** (101 MHz, $CDCl_3$) δ 167.1, 139.8, 138.8, 137.4, 133.1, 133.0, 132.9, 131.4, 131.2, 130.1, 128.4, 126.9 (q, *J* = 5.1 Hz), 124.8, 124.7, 124.4 (q, *J* = 273.7 Hz), 123.2 (q, *J* = 288.9 Hz), 120.6, 118.2 (q, *J* = 29.3 Hz), 117.3, 80.5 (q, *J* = 29.3 Hz), 21.2. **IR** (KBr, cm^{-1}) ν: 3468, 3046, 2921, 1729, 1605, 1517, 1475, 1363, 1312, 1267, 1173, 1133, 1106, 1032, 974, 764, 722, 517. **HRMS** (ESI) calcd for $C_{23}H_{17}F_6N_2O^+$ *m/z* 451.1240 $[M+H]^+$, Found 451.1242.

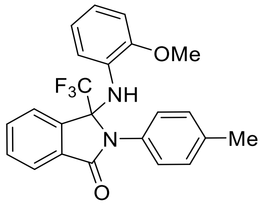

3n

3-((2-methoxyphenyl)amino)-2-(p-tolyl)-3-(trifluoromethyl)isoindolin-1-one 3n. Starting from **1n** and *p*-tolyl isocyanate (Cas: 622-58-2), PhCl as solvent, 48 h. The product was purified by flash column chromatography on silica gel (height 16 cm, width 1.5 cm, eluent: petroleum ether/ethyl acetate, gradient: 99:1 to 96:4) as a white solid, yield = 74% (91.0 mg). R*f* (petroleum ether/ethyl acetate = 91:9): 0.3. **^{1}H NMR** (400 MHz, $CDCl_3$) δ 8.08 (d, *J* = 6.4 Hz, 1H), 7.76 – 7.56 (m, 3H), 7.18 (d, *J* = 7.6 Hz, 2H), 7.00 (d, *J* = 4.8 Hz, 2H), 6.92 – 6.70 (m, 2H), 6.53 (t, *J* = 7.6 Hz, 1H), 5.81 (t, *J* = 6.4 Hz, 1H), 5.55 (s, 1H), 3.87 (s, 3H), 2.37 (s, 3H). **^{19}F NMR** (376 MHz, $CDCl_3$) δ -77.4 (s). **^{13}C NMR** (101 MHz, $CDCl_3$) δ 167.5, 147.6, 138.8, 138.3, 132.9, 132.7, 131.5, 131.4, 131.1, 129.8, 128.7, 124.6, 123.4 (q, *J* = 288.9 Hz), 121.0, 120.11, 120.08, 113.9, 110.1, 80.7 (q, *J* = 29.3 Hz), 55.7, 21.1. **IR** (KBr, cm^{-1}) ν: 3375, 3036, 2931, 1710, 1603, 1522, 1362, 1179, 1025, 975, 735. **HRMS** (ESI) calcd for $C_{23}H_{20}F_3N_2O_2^+$ *m/z* 413.1471 $[M+H]^+$, Found 413.1476.

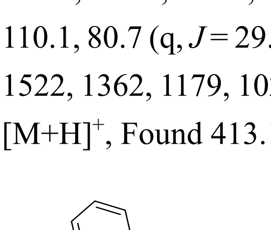

3o

3-((2-bromophenyl)amino)-2-(p-tolyl)-3-(trifluoromethyl)isoindolin-1-one 3o.

Starting from **1o** and *p*-tolyl isocyanate (Cas: 622-58-2), PhCl as solvent, 48 h. The product was purified by flash column chromatography on silica gel (height 16 cm, width 1.5 cm, eluent: petroleum ether/ethyl acetate, gradient: 99:1 to 97:3) as a white solid, yield = 57% (78.3 mg). R_f (petroleum ether/ethyl acetate = 91:9): 0.3. **^{1}H NMR** (400 MHz, $CDCl_3$) δ 8.07 (d, J = 9.2 Hz, 1H), 7.80 – 7.45 (m, 4H), 7.21 – 7.11 (m, 2H), 7.02 – 6.55 (m, 4H), 6.06 – 5.83 (m, 1H), 5.49 (s, 1H), 2.37 (s, 3H). **^{19}F NMR** (376 MHz, $CDCl_3$) δ -77.5 (s). **^{13}C NMR** (101 MHz, $CDCl_3$) δ 167.3, 139.2, 138.6, 137.9, 133.1, 132.9, 132.7, 131.3, 131.1, 130.0, 128.4, 124.7, 124.5, 123.2 (q, J = 289.9 Hz), 121.6, 115.4, 112.4, 80.7 (q, J = 29.3 Hz), 21.2, one carbon is overlapped. **IR** (KBr, cm^{-1}) ν: 3391, 3063, 1720, 1599, 1519, 1466, 1362, 1261, 1184, 1020, 734. **HRMS** (ESI) calcd for $C_{22}H_{17}BrF_3N_2O^+$ *m/z* 461.0471 $[M+H]^+$, Found 461.0480.

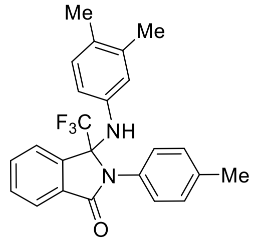

3p

3-((3,4-dimethylphenyl)amino)-2-(p-tolyl)-3-(trifluoromethyl)isoindolin-1-one 3p. Starting from **1p** and *p*-tolyl isocyanate (Cas: 622-58-2), o-xylene as solvent, 60 h. The product was purified by flash column chromatography on silica gel (height 16 cm, width 1.5 cm, eluent: petroleum ether/ethyl acetate, gradient: 99:1 to 98:2) as a white solid, yield = 77% (95.0 mg). R_f (petroleum ether/ethyl acetate = 91:9): 0.4. **^{1}H NMR** (400 MHz, $CDCl_3$) δ 8.06 (d, J = 7.2 Hz, 1H), 7.79 – 7.49 (m, 3H), 7.31 – 6.93 (m, 4H), 6.78 (d, J = 6.8 Hz, 1H), 6.24 (s, 1H), 5.98 (s, 1H), 4.68 (s, 1H), 2.37 (s, 3H), 2.11 (s, 3H), 2.06 (s, 3H). **^{19}F NMR** (376 MHz, $CDCl_3$) δ -77.6 (s). **^{13}C NMR** (101 MHz, $CDCl_3$) δ 167.4, 139.4, 138.4, 137.5, 132.9, 132.8, 131.5, 131.4, 131.0, 130.2, 129.9, 128.9, 128.8, 124.8, 124.5, 123.4 (q, J = 288.9 Hz), 118.4, 113.7, 80.8 (q, J = 28.3 Hz), 21.1, 19.8, 18.6. **IR** (KBr, cm^{-1}) ν: 3348, 3052, 2965, 1696, 1618, 1516, 1366, 1265, 1179, 1052, 804, 718. **HRMS** (ESI) calcd for $C_{24}H_{22}F_3N_2O^+$ *m/z* 411.1679 $[M+H]^+$, Found 411.1681.

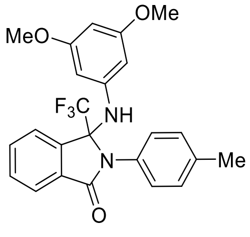

3q

3-((3,5-dimethoxyphenyl)amino)-2-(p-tolyl)-3-(trifluoromethyl)isoindolin-1-one 3q. Starting from **1q** and *p*-tolyl isocyanate (Cas: 622-58-2), o-xylene as solvent, 60 h. The product was purified by flash column chromatography on silica gel (height 18 cm, width 1.5 cm, eluent: petroleum ether/ethyl acetate, gradient: 99:1 to 95:5) as a white solid, yield = 68% (90.6 mg). R*f* (petroleum ether/ethyl acetate = 91:9): 0.4. **^{1}H NMR** (400 MHz, $CDCl_3$) *δ* 8.09 – 8.01 (m, 1H), 7.73 – 7.60 (m, 3H), 7.20 (d, *J* = 8.4 Hz, 2H), 7.05 (d, *J* = 8.4 Hz, 2H), 5.95 (t, *J* = 2.4 Hz, 1H), 5.46 (d, *J* = 2.0 Hz, 2H), 4.71 (s, 1H), 3.52 (s, 6H), 2.37 (s, 3H). **^{19}F NMR** (376 MHz, $CDCl_3$) *δ* -77.5 (s). **^{13}C NMR** (101 MHz, $CDCl_3$) *δ* 167.2, 161.4, 143.5, 138.6, 138.5, 133.0, 132.7, 131.3, 131.2, 130.0, 128.8, 124.8, 124.5, 123.3 (q, *J* = 288.9 Hz), 94.9, 93.3, 80.6 (q, *J* = 29.3 Hz), 54.9, 21.1. **IR** (KBr, cm^{-1}) ν: 3322, 3105, 2946, 1706, 1612, 1459, 1368, 1210, 1150, 1062, 815, 731. **HRMS** (ESI) calcd for $C_{24}H_{22}F_3N_2O_3^+$ *m/z* 443.1577 $[M+H]^+$, Found 443.1585.

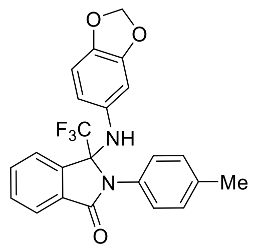

3r

3-(benzo[d][1,3]dioxol-5-ylamino)-2-(p-tolyl)-3-(trifluoromethyl)isoindolin-1-one 3r. Starting from **1r** and *p*-tolyl isocyanate (Cas: 622-58-2), o-xylene as solvent, 60 h. The product was purified by flash column chromatography on silica gel (height 16 cm, width 1.5 cm, eluent: petroleum ether/ethyl acetate, gradient: 99:1 to 95:5) as a white solid, yield = 73% (93.3 mg). R*f* (petroleum ether/ethyl acetate = 91:9): 0.2. **^{1}H NMR** (400 MHz, $CDCl_3$) *δ* 8.02 (d, *J* = 6.4 Hz, 1H), 7.70 – 7.51 (m, 3H), 7.21 (d, *J* = 8.0 Hz, 2H), 7.13 – 7.03 (m, 2H), 6.49 (d, *J* = 8.4 Hz, 1H), 5.97 (s, 1H), 5.86 – 5.74 (m, 3H), 4.66 (s, 1H), 2.37 (s, 3H). **^{19}F NMR** (376 MHz, $CDCl_3$) *δ* -76.9 (s). **^{13}C NMR** (101 MHz, $CDCl_3$) *δ* 167.4, 148.3, 142.1, 138.6, 138.2, 136.4, 132.9, 131.4, 131.2, 130.0, 128.8, 124.8, 124.7, 123.3 (q, *J* = 288.9 Hz), 109.4, 108.4, 101.0, 99.7, 81.0 (q, *J* = 29.3 Hz), 21.2, one C is overlapped. **IR** (KBr, cm^{-1}) ν: 3323, 3093, 2891, 1699, 1620, 1502, 1370, 1189, 1041, 916, 813, 724. **HRMS** (ESI) calcd for $C_{23}H_{18}F_3N_2O_3^+$ *m/z* 427.1264 $[M+H]^+$, Found 427.1271.

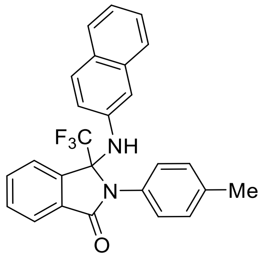

3s

3-(naphthalen-2-ylamino)-2-(p-tolyl)-3-(trifluoromethyl)isoindolin-1-one 3s. Starting from **1s** and *p*-tolyl isocyanate (Cas: 622-58-2), o-xylene as solvent, 60 h. The product was purified by flash column chromatography on silica gel (height 16 cm, width 1.5 cm, eluent: petroleum ether/ethyl acetate, gradient: 99:1 to 98:2) as a white solid, yield = 71% (92.5 mg). R_f (petroleum ether/ethyl acetate = 91:9): 0.3. **^{1}H NMR** (400 MHz, $CDCl_3$) δ 8.16 (d, J = 7.6 Hz, 1H), 7.77 – 7.58 (m, 5H), 7.39 – 7.26 (m, 3H), 7.20 (d, J = 8.4 Hz, 2H), 7.12 (d, J = 8.4 Hz, 2H), 6.84 (dd, J = 9.2, 2.4 Hz, 1H), 6.39 (d, J = 2.4 Hz, 1H), 5.08 (s, 1H), 2.38 (s, 3H). **^{19}F NMR** (376 MHz, $CDCl_3$) δ -77.5 (s). **^{13}C NMR** (101 MHz, $CDCl_3$) δ 167.4, 139.1, 138.6, 138.1, 134.0, 133.0, 132.9, 131.3, 131.2, 130.0, 129.3, 128.8, 128.6, 127.4, 126.7, 126.5, 124.7, 124.7, 123.8, 123.3 (q, J = 288.9 Hz), 118.8, 110.7, 80.7 (q, J = 29.3 Hz), 21.1. **IR** (KBr, cm^{-1}) ν: 3334, 3055, 2925, 1709, 1633, 1517, 1364, 1266, 1184, 1055, 826, 720. **HRMS** (ESI) calcd for $C_{26}H_{20}F_3N_2O^+$ *m/z* 433.1522 $[M+H]^+$, Found 433.1532.

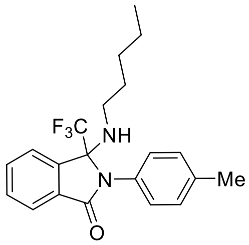

3t

3-(pentylamino)-2-(p-tolyl)-3-(trifluoromethyl)isoindolin-1-one 3t. Starting from **1t** and *p*-tolyl isocyanate (Cas: 622-58-2), o-xylene as solvent, 60 h. The product was purified by flash column chromatography on silica gel (height 20 cm, width 1.5 cm, eluent: petroleum ether/ethyl acetate, gradient: 99:1 to 95:5) as a white solid, yield = 74% (83.1 mg). R_f (petroleum ether/ethyl acetate = 91:9): 0.4. **^{1}H NMR** (400 MHz, $CDCl_3$) δ 7.95 (d, J = 7.2 Hz, 1H), 7.73 – 7.59 (m, 3H), 7.31 (d, J = 8.4 Hz, 2H), 7.13 (d, J = 8.0 Hz, 2H), 2.60 – 2.50 (m, 1H), 2.41 (s, 3H), 2.25 – 2.10 (m, 2H), 1.56 – 1.37 (m, 2H), 1.33 – 1.18 (m, 4H), 0.87 (t, J = 7.2 Hz, 3H). **^{19}F NMR** (376 MHz, $CDCl_3$) δ -76.7 (s). **^{13}C NMR** (101 MHz, $CDCl_3$) δ 168.0, 138.7, 138.3, 132.8, 132.5, 131.5, 130.8, 130.3, 128.7, 124.7, 124.3, 123.5 (q, J = 287.9 Hz), 83.6 (q, J = 29.3 Hz), 41.6, 29.5, 29.2, 22.4, 21.2, 13.9. **IR** (KBr, cm^{-1}) ν: 3341, 2930, 2864, 1703, 1512, 1466,

1361, 1282, 1176, 1034, 726. **HRMS** (ESI) calcd for $C_{21}H_{24}F_3N_2O^+$ *m/z* 377.1835 $[M+H]^+$, Found 377.1842.

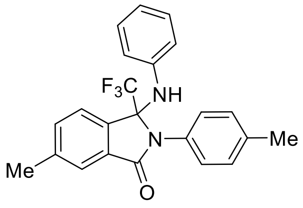

3u

6-methyl-3-(phenylamino)-2-(p-tolyl)-3-(trifluoromethyl)isoindolin-1-one 3u. Starting from **1u** and *p*-tolyl isocyanate (Cas: 622-58-2), PhCl as solvent, 48 h. The product was purified by flash column chromatography on silica gel (height 16 cm, width 1.5 cm, eluent: petroleum ether/ethyl acetate, gradient: 99:1 to 97:3) as a white solid, yield = 89% (105.8 mg). R_f (petroleum ether/ethyl acetate = 91:9): 0.3. **^{1}H NMR** (400 MHz, $CDCl_3$) δ 7.87 (s, 1H), 7.49 (d, J = 7.6 Hz, 1H), 7.42 (d, J = 8.4 Hz, 1H), 7.18 (d, J = 8.4 Hz, 2H), 7.13 – 6.97 (m, 4H), 6.83 (t, J = 7.6 Hz, 1H), 6.33 (d, J = 7.6 Hz, 2H), 4.78 (s, 1H), 2.52 (s, 3H), 2.36 (s, 3H). **^{19}F NMR** (376 MHz, $CDCl_3$) δ -77.7 (s). **^{13}C NMR** (101 MHz, $CDCl_3$) δ 167.6, 141.8, 141.7, 138.5, 135.4, 133.8, 132.9, 131.4, 129.9, 129.3, 128.8, 125.0, 124.4, 123.4 (q, J = 288.9 Hz), 120.7, 116.4, 80.5 (q, J = 29.3 Hz), 21.5, 21.1. **IR** (KBr, cm^{-1}) ν: 3337, 3055, 2924, 1703, 1609, 1507, 1364, 1265, 1186, 1061, 742. **HRMS** (ESI) calcd for $C_{23}H_{20}F_3N_2O^+$ *m/z* 397.1522 $[M+H]^+$, Found 397.1530. Contaminated with trace inseparable impurity.

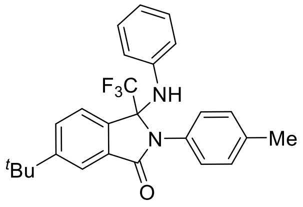

3v

6-(tert-butyl)-3-(phenylamino)-2-(p-tolyl)-3-(trifluoromethyl)isoindolin-1-one 3v. Starting from **1v** and *p*-tolyl isocyanate (Cas: 622-58-2), o-xylene as solvent, 140 °C, 60 h. The product was purified by flash column chromatography on silica gel (height 16 cm, width 1.5 cm, eluent: petroleum ether/ethyl acetate, gradient: 99:1 to 98:2) as a white solid, yield = 71% (93.7 mg). R_f (petroleum ether/ethyl acetate = 91:9): 0.5. **^{1}H NMR** (400 MHz, $CDCl_3$) δ 8.08 (d, J = 2.0 Hz, 1H), 7.64 (dd, J = 8.0, 2.0 Hz, 1H), 7.51 (d, J = 8.0 Hz, 1H), 7.18 (d, J = 8.4 Hz, 2H), 7.10 – 6.98 (m, 4H), 6.82 (t, J = 7.6 Hz, 1H), 6.31 (d, J = 8.0 Hz, 2H), 4.71 (s, 1H), 2.36 (s, 3H), 1.40 (s, 9H). **^{19}F NMR** (376 MHz, $CDCl_3$) δ -77.6 (s). **^{13}C NMR** (101 MHz, $CDCl_3$) δ 167.9, 155.1, 141.8, 138.5, 135.4, 132.6, 131.5, 130.3, 129.9, 129.3, 128.9, 124.3, 123.4 (q, J = 288.9 Hz), 121.6, 120.7, 116.4, 80.5 (q, J = 29.3 Hz), 35.3, 31.3, 21.2. **IR** (KBr, cm^{-1}) ν: 3309,

3035, 2963, 1698, 1604, 1508, 1359, 1262, 1176, 1038, 977, 819, 744. **HRMS** (ESI) calcd for $C_{26}H_{26}F_3N_2O^+$ *m/z* 439.1992 $[M+H]^+$, Found 439.1989.

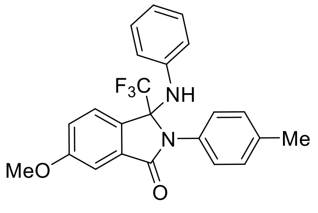

3w

6-methoxy-3-(phenylamino)-2-(p-tolyl)-3-(trifluoromethyl)isoindolin-1-one 3w. Starting from **1w** and *p*-tolyl isocyanate (Cas: 622-58-2), o-xylene as solvent, 130 °C, 60 h. The product was purified by flash column chromatography on silica gel (height 16 cm, width 1.5 cm, eluent: petroleum ether/ethyl acetate, gradient: 99:1 to 95:5) as a white solid, yield = 84% (103.8 mg). R_f (petroleum ether/ethyl acetate = 91:9): 0.3. **^{1}H NMR** (400 MHz, $CDCl_3$) δ 7.53 (d, J = 2.4 Hz, 1H), 7.48 (d, J = 8.4 Hz, 1H), 7.20 – 6.97 (m, 7H), 6.83 (t, J = 7.6 Hz, 1H), 6.32 (d, J = 8.0 Hz, 2H), 4.69 (s, 1H), 3.93 (s, 3H), 2.36 (s, 3H). **^{19}F NMR** (376 MHz, $CDCl_3$) δ -77.7 (s). **^{13}C NMR** (101 MHz, $CDCl_3$) δ 167.4, 162.2, 141.8, 138.6, 134.4, 131.4, 130.0, 129.9, 129.3, 128.8, 125.7, 123.3 (q, J = 288.9 Hz), 120.7, 120.6, 116.5, 107.9, 80.4 (q, J = 29.3 Hz), 55.8, 21.1. **IR** (KBr, cm^{-1}) ν: 3346, 3048, 2926, 1711, 1609, 1503, 1364, 1261, 1178, 1132, 1082, 972, 827, 740. **HRMS** (ESI) calcd for $C_{23}H_{20}F_3N_2O_2^+$ *m/z* 413.1471 $[M+H]^+$, Found 413.1476.

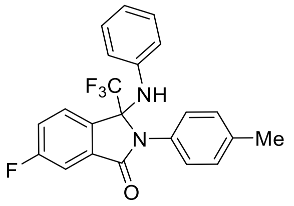

3x

6-fluoro-3-(phenylamino)-2-(p-tolyl)-3-(trifluoromethyl)isoindolin-1-one 3x. Starting from **1x** and *p*-tolyl isocyanate (Cas: 622-58-2), PhCl as solvent, 48 h. The product was purified by flash column chromatography on silica gel (height 16 cm, width 1.5 cm, eluent: petroleum ether/ethyl acetate, gradient: 99:1 to 96:4) as a white solid, yield = 75% (90.4 mg). R_f (petroleum ether/ethyl acetate = 91:9): 0.4. **^{1}H NMR** (400 MHz, $CDCl_3$) δ 7.79 – 7.64 (m, 1H), 7.62 – 7.52 (m, 1H), 7.36 – 7.27 (m, 1H), 7.20 (d, J = 7.2 Hz, 2H), 7.13 – 6.98 (m, 4H), 6.86 (t, J = 7.6 Hz, 1H), 6.35 (t, J = 8.4 Hz, 2H), 4.93 (s, 1H), 2.37 (s, 3H). **^{19}F NMR** (376 MHz, $CDCl_3$) δ -77.5 (s), -108.3 (s). **^{13}C NMR** (101 MHz, $CDCl_3$) δ 166.1, 164.5 (d, J = 253.5 Hz), 141.5, 138.7, 135.2 (d, J = 9.1 Hz), 133.7, 131.1, 130.0, 129.4, 128.6, 126.6 (d, J = 8.1 Hz), 123.2 (q, J = 289.9 Hz), 121.0, 120.3 (d, J = 23.2 Hz), 116.5, 111.7 (d, J = 24.2 Hz), 80.5 (q, J = 29.3 Hz),

21.07. **IR** (KBr, cm^{-1}) ν: 3440, 3349, 3066, 1713, 1609, 1502, 1363, 1261, 1177, 1117, 728. **HRMS** (ESI) calcd for $C_{22}H_{17}F_4N_2O^+$ *m/z* 401.1272 $[M+H]^+$, Found 401.1274.

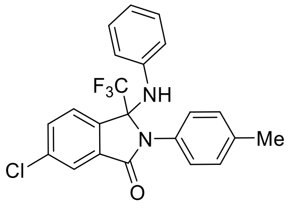

3y

6-chloro-3-(phenylamino)-2-(p-tolyl)-3-(trifluoromethyl)isoindolin-1-one 3y. Starting from **1y** and *p*-tolyl isocyanate (Cas: 622-58-2), PhCl as solvent, 48 h. The product was purified by flash column chromatography on silica gel (height 16 cm, width 1.5 cm, eluent: petroleum ether/ethyl acetate, gradient: 99:1 to 95:5) as a white solid, yield = 74% (92.5 mg). R_f (petroleum ether/ethyl acetate = 91:9): 0.4. **^{1}H NMR** (400 MHz, $CDCl_3$) δ 8.02 (d, *J* = 1.2 Hz, 1H), 7.65 – 7.47 (m, 2H), 7.19 (d, *J* = 8.0 Hz, 2H), 7.14 – 6.97 (m, 4H), 6.86 (t, *J* = 7.6 Hz, 1H), 6.35 (d, *J* = 8.0 Hz, 2H), 4.84 (s, 1H), 2.37 (s, 3H). **^{19}F NMR** (376 MHz, $CDCl_3$) δ -77.5 (s). **^{13}C NMR** (101 MHz, $CDCl_3$) δ 166.0, 141.4, 138.8, 137.7, 136.5, 134.6, 133.1, 131.0, 130.0, 129.4, 128.7, 126.0, 124.9, 123.1 (q, *J* = 289.9 Hz), 121.1, 116.5, 80.6 (q, *J* = 29.3 Hz), 21.1. **IR** (KBr, cm^{-1}) ν: 3320, 3072, 2925, 1706, 1606, 1508, 1429, 1360, 1268, 1186, 1062, 972, 744. **HRMS** (ESI) calcd for $C_{22}H_{17}ClF_3N_2O^+$ *m/z* 417.0976 $[M+H]^+$, Found 417.0988.

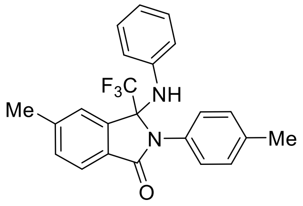

3z1

5-methyl-3-(phenylamino)-2-(p-tolyl)-3-(trifluoromethyl)isoindolin-1-one 3z1. Starting from **1z** and *p*-tolyl isocyanate (Cas: 622-58-2), o-xylene as solvent, 60 h. The product was purified by flash column chromatography on silica gel (height 16 cm, width 1.5 cm, eluent: petroleum ether/ethyl acetate, gradient: 99:1 to 95:5) as a white solid, yield = 61% (73.1 mg). R_f (petroleum ether/ethyl acetate = 91:9): 0.3. **^{1}H NMR** (400 MHz, $CDCl_3$) δ 7.94 (d, *J* = 8.0 Hz, 1H), 7.46 (d, *J* = 7.6 Hz, 1H), 7.42 (s, 1H), 7.16 (d, *J* = 8.4 Hz, 2H), 7.12 – 7.03 (m, 2H), 6.99 (d, *J* = 8.4 Hz, 2H), 6.83 (t, *J* = 7.6 Hz, 1H), 6.31 (d, *J* = 8.0 Hz, 2H), 4.71 (s, 1H), 2.44 (s, 3H), 2.35 (s, 3H). **^{19}F NMR** (376 MHz, $CDCl_3$) δ -77.6 (s). **^{13}C NMR** (101 MHz, $CDCl_3$) δ 167.6, 143.9, 141.8, 138.7, 138.4, 132.1, 131.5, 130.2, 129.9, 129.3, 128.9, 125.0, 124.5, 123.4 (q, *J* = 288.9 Hz), 120.6, 116.3, 80.5 (q, *J* = 29.3 Hz), 21.9, 21.1. **IR** (KBr, cm^{-1}) ν: 3443, 3312, 3077, 2924, 1703, 1611, 1505, 1367, 1265, 1178, 1066, 905, 745. **HRMS** (ESI) calcd for

$C_{23}H_{20}F_3N_2O^+$ *m/z* 397.1522 [M+H]$^+$, Found 397.1531.

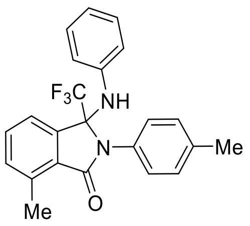

3z2

7-methyl-3-(phenylamino)-2-(p-tolyl)-3-(trifluoromethyl)isoindolin-1-one 3z2. Starting from **1z** and *p*-tolyl isocyanate (Cas: 622-58-2), o-xylene as solvent, 60 h . The product was purified by flash column chromatography on silica gel (height 16 cm, width 1.5 cm, eluent: petroleum ether/ethyl acetate, gradient: 99:1 to 98:2) as a white solid, yield = 17% (19.8 mg). R_f (petroleum ether/ethyl acetate = 91:9): 0.5. **^{1}H NMR** (300 MHz, $CDCl_3$) δ 77.51 – 7.37 (m, 3H), 7.17 (d, *J* = 8.1 Hz, 2H), 7.11 – 6.94 (m, 4H), 6.82 (t, *J* = 7.2 Hz, 1H), 6.32 (d, *J* = 7.8 Hz, 2H), 4.70 (s, 1H), 2.82 (s, 3H), 2.35 (s, 3H). **^{19}F NMR** (376 MHz, $CDCl_3$) δ -77.2 (s). **^{13}C NMR** (101 MHz, $CDCl_3$) δ 168.3, 141.8, 139.0, 138.7, 138.4, 133.4, 132.3, 131.5, 129.9, 129.7, 129.3, 129.0, 125.4, 123.4 (q, *J* = 289.9 Hz), 120.7, 116.4, 79.9 (q, *J* = 29.3 Hz), 21.2, 17.5. **IR** (KBr, cm^{-1}) ν: 3347, 3053, 2923, 1697, 1604, 1508, 1358, 1262, 1170, 1024, 735, 688. **HRMS** (ESI) calcd for $C_{23}H_{20}F_3N_2O^+$ *m/z* 397.1522 [M+H]$^+$, Found 397.1533.

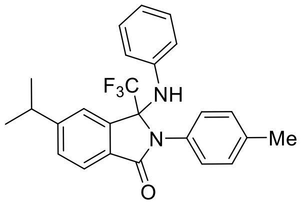

3aa

5-isopropyl-3-(phenylamino)-2-(p-tolyl)-3-(trifluoromethyl)isoindolin-1-one 3aa. Starting from **1aa** and *p*-tolyl isocyanate (Cas: 622-58-2), o-xylene as solvent, 60 h . The product was purified by flash column chromatography on silica gel (height 16 cm, width 1.5 cm, eluent: petroleum ether/ethyl acetate, gradient: 99:1 to 98:2) as a white solid, yield = 58% (49.4 mg, 0.2 mmol). R_f (petroleum ether/ethyl acetate = 91:9): 0.5. **^{1}H NMR** (400 MHz, $CDCl_3$) δ 7.97 (d, *J* = 8.0 Hz, 1H), 7.53 (dd, *J* = 7.6, 1.2 Hz, 1H), 7.45 (s, 1H), 7.18 (d, *J* = 8.0 Hz, 2H), 7.09 – 6.99 (m, 4H), 6.82 (t, *J* = 7.6 Hz, 1H), 6.30 (d, *J* = 8.0 Hz, 2H), 4.77 (s, 1H), 3.07 – 2.92 (m, 1H), 2.36 (s, 3H), 1.26 – 1.16 (m, 6H). **^{19}F NMR** (376 MHz, $CDCl_3$) δ -77.6 (s). **^{13}C NMR** (101 MHz, $CDCl_3$) δ 167.6, 154.8, 141.8, 138.6, 138.4, 131.5, 130.5, 129.9, 129.4, 129.2, 128.9, 124.6, 123.4 (q, *J* = 288.9 Hz), 122.8, 120.7, 116.5, 80.6 (q, *J* = 30.3 Hz), 34.5, 29.6, 23.7, 21.1. **IR** (KBr,

cm^{-1}) ν: 3368, 2967, 2924, 1703, 1610, 1508, 1360, 1261, 1176, 1139, 1051, 749. **HRMS** (ESI) calcd for $C_{25}H_{24}F_3N_2O^+$ *m/z* 425.1835 [M+H]$^+$, Found 425.1844.

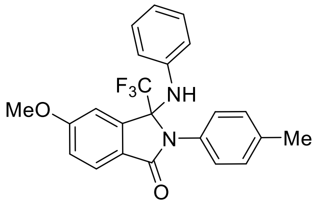

3ab

5-methoxy-3-(phenylamino)-2-(p-tolyl)-3-(trifluoromethyl)isoindolin-1-one 3ab. Starting from **1ab** and *p*-tolyl isocyanate (Cas: 622-58-2), o-xylene as solvent, 60 h. The product was purified by flash column chromatography on silica gel (height 18 cm, width 1.5 cm, eluent: petroleum ether/ethyl acetate, gradient: 99:1 to 91:9) as a white solid, yield = 85% (105.5 mg). R*f* (petroleum ether/ethyl acetate = 91:9): 0.2. **^{1}H NMR** (400 MHz, $CDCl_3$) *δ* 7.96 (d, *J* = 8.4 Hz, 1H), 7.21 – 7.12 (m, 3H), 7.12 – 7.03 (m, 3H), 7.00 (d, *J* = 7.6 Hz, 2H), 6.84 (t, *J* = 7.2 Hz, 1H), 6.35 (d, *J* = 8.4 Hz, 2H), 4.75 (s, 1H), 3.84 (s, 3H), 2.35 (s, 3H). **^{19}F NMR** (376 MHz, $CDCl_3$) *δ* -77.7 (s). **^{13}C NMR** (101 MHz, $CDCl_3$) *δ* 167.3, 163.6, 141.7, 140.7, 138.3, 131.5, 129.8, 129.3, 128.9, 126.0, 125.0, 123.3 (q, *J* = 288.9 Hz), 120.7, 117.3, 116.3, 109.7, 80.2 (q, *J* = 29.3 Hz), 55.7, 21.1. **IR** (KBr, cm^{-1}) ν: 3348, 3060, 2933, 1702, 1607, 1504, 1362, 1262, 1180, 1070, 724. **HRMS** (ESI) calcd for $C_{23}H_{20}F_3N_2O_2^+$ *m/z* 413.1471 [M+H]$^+$, Found 413.1474.

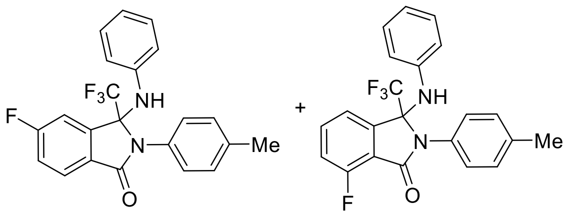

3ac

5-fluoro-3-(phenylamino)-2-(p-tolyl)-3-(trifluoromethyl)isoindolin-1-one 3ac. Starting from **1ac** and *p*-tolyl isocyanate (Cas: 622-58-2), o-xylene as solvent, 60 h. The product was purified by flash column chromatography on silica gel (height 18 cm, width 1.5 cm, eluent: petroleum ether/ethyl acetate, gradient: 99:1 to 95:5) as a white solid, yield = 85% (102.0 mg, **mixture of isomer, ratio = 1:0.20**). R*f* (petroleum ether/ethyl acetate = 91:9): 0.3. **^{1}H NMR** (400 MHz, $CDCl_3$) *δ* 8.03 (dd, *J* = 8.0, 4.8 Hz, 1H), 7.62 – 7.55 (m, 0.22H), 7.43 – 7.28 (m, 2.45H), 7.19 (d, *J* = 8.0 Hz, 2.41H), 7.14 – 7.06 (m, 2.45H), 7.03 (dd, *J* = 8.4, 2.0 Hz, 2.37H), 6.86 (t, *J* = 7.6 Hz, 1.20H), 6.42 – 6.31 (m, 2.38H), 4.83 (s, 1.19H), 2.36 (s, 3.60H). **^{19}F NMR** (376 MHz, $CDCl_3$) *δ* -77.5 (s, major), -77.6 (s, minor), -104.5 (s, major), -115.2 (s, minor). **^{13}C NMR** (101 MHz, $CDCl_3$, major) *δ* 166.3, 165.6 (d, *J* = 255.5 Hz), 141.4, 141.0 (d, *J* = 10.1 Hz), 138.7, 131.1, 130.0, 129.4, 128.7, 126.8 (d, *J* = 24.2 Hz), 123.1 (q, *J* = 288.9 Hz), 121.1,

118.9 (d, J = 255.5 Hz), 116.4, 112.6, 112.3, 80.2 (q, J = 30.3 Hz), 21.1. **IR** (KBr, cm^{-1}) ν: 3350, 3069, 2925, 1720, 1609, 1492, 1360, 1261, 1175, 1071, 987, 794, 743. **HRMS** (ESI) calcd for $C_{22}H_{17}F_4N_2O^+$ *m/z* 401.1272 $[M+H]^+$, Found 401.1276.

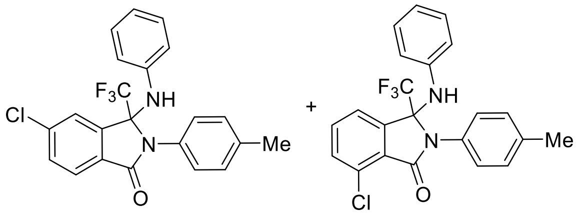

3ad

5-chloro-3-(phenylamino)-2-(p-tolyl)-3-(trifluoromethyl)isoindolin-1-one 3ad. Starting from **1ad** and *p*-tolyl isocyanate (Cas: 622-58-2), o-xylene as solvent, 60 h. The product was purified by flash column chromatography on silica gel (height 18 cm, width 1.5 cm, eluent: petroleum ether/ethyl acetate, gradient: 99:1 to 97:3) as a white solid, yield = 57% (71.0 mg, **mixture of isomer, ratio = 1:0.24**). R_f (petroleum ether/ethyl acetate = 91:9): 0.3. **^{1}H NMR** (400 MHz, $CDCl_3$) δ 7.95 (d, J = 8.0 Hz, 1H), 7.65 – 7.55 (m, 2.20H), 7.52 – 7.47 (m, 0.42H), 7.20 – 6.96 (m, 7.74H), 6.85 (t, J = 7.2 Hz, 1.24H), 6.33 (d, J = 7.2 Hz, 2.46H), 4.91 – 4.72 (m, 1.24H), 2.34 (s, 3.70H). **^{19}F NMR** (376 MHz, $CDCl_3$) δ -77.47 (s, major), -77.54 (s, minor). **^{13}C NMR** (101 MHz, $CDCl_3$, major) δ 166.4, 141.4, 140.1, 139.5, 138.8, 131.8, 131.3, 131.0, 130.0, 129.5, 128.7, 125.8, 125.0, 123.1 (q, J = 288.9 Hz), 121.1, 116.3, 80.3 (q, J = 29.3 Hz), 21.1. **^{13}C NMR** (101 MHz, $CDCl_3$, minor) δ 164.9, 141.4, 140.7, 140.1, 138.6, 133.5, 133.0, 132.6, 129.9, 129.4, 128.8, 125.2, 123.2, 121.0, 116.4, 29.6, two carbon were overlapped. **IR** (KBr, cm^{-1}) ν: 3338, 3068, 2923, 1705, 1607, 1507, 1366, 1260, 1174, 1061, 745. **HRMS** (ESI) calcd for $C_{22}H_{17}ClF_3N_2O^+$ *m/z* 417.0976 $[M+H]^+$, Found 417.0986.

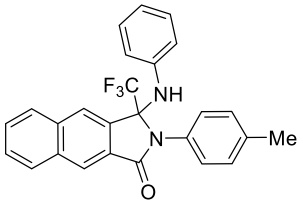

3ae

3-(phenylamino)-2-(p-tolyl)-3-(trifluoromethyl)-2,3-dihydro-1H-benzo[f]isoindol-1-one 3ae. Starting from **1ae** and *p*-tolyl isocyanate (Cas: 622-58-2), o-xylene as solvent, 60 h. The product was purified by flash column chromatography on silica gel (height 16 cm, width 1.5 cm, eluent: petroleum ether/ethyl acetate, gradient: 99:1 to 97:3) as a white solid, yield = 80% (103.8 mg). R_f (petroleum ether/ethyl acetate = 91:9): 0.2. **^{1}H NMR** (400 MHz, $CDCl_3$) δ 8.62 (s, 1H), 8.11 (t, J = 4.4 Hz, 2H), 7.95 – 7.86 (m, 1H), 7.70 – 7.57 (m, 2H), 7.21 (d, J = 8.4 Hz, 2H), 7.12 – 6.98 (m, 4H), 6.80 (t, J

= 7.6 Hz, 1H), 6.36 (d, J = 8.4 Hz, 2H), 4.94 (s, 1H), 2.38 (s, 3H). **^{19}F NMR** (376 MHz, $CDCl_3$) δ -77.8 (s). **^{13}C NMR** (101 MHz, $CDCl_3$) δ 167.5, 141.6, 138.6, 135.3, 134.2, 132.8, 131.5, 129.9, 129.7, 129.3, 129.2, 129.0, 128.8, 128.4, 127.8, 125.6, 124.8, 123.5 (q, J = 289.9 Hz), 120.6, 116.3, 80.7 (q, J = 29.3 Hz), 21.1. **IR** (KBr, cm^{-1}) ν: 3337, 3054, 2924, 1710, 1604, 1508, 1363, 1263, 1174, 1061, 961, 903, 743. **HRMS** (ESI) calcd for $C_{26}H_{20}F_3N_2O^+$ m/z 433.1522 $[M+H]^+$, Found 433.1527.

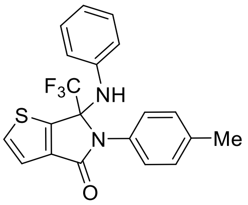

3af

6-(phenylamino)-5-(p-tolyl)-6-(trifluoromethyl)-5,6-dihydro-4H-thieno[2,3-c]pyrrol-4-one 3af. Starting from **1af** and *p*-tolyl isocyanate (Cas: 622-58-2), o-xylene as solvent, 110 °C, 60 h. The product was purified by flash column chromatography on silica gel (height 16 cm, width 1.5 cm, eluent: petroleum ether/ethyl acetate, gradient: 99:1 to 97:3) as a pale solid, yield = 24% (28.3 mg). R_f (petroleum ether/ethyl acetate = 91:9): 0.3. **^{1}H NMR** (400 MHz, $CDCl_3$) δ 7.60 (d, J = 4.8 Hz, 1H), 7.43 (d, J = 5.2 Hz, 1H), 7.20 – 7.09 (m, 4H), 7.03 (d, J = 8.0 Hz, 2H), 6.89 (t, J = 7.6 Hz, 1H), 6.45 (d, J = 7.6 Hz, 2H), 4.66 (s, 1H), 2.36 (s, 3H). **^{19}F NMR** (376 MHz, $CDCl_3$) δ -78.1 (s). **^{13}C NMR** (101 MHz, $CDCl_3$) δ 163.7, 147.1, 142.7, 141.5, 138.8, 133.9, 131.4, 130.0, 129.5, 129.2, 122.7 (q, J = 288.9 Hz), 121.4, 121.1, 116.6, 79.8 (q, J = 30.3 Hz), 21.2. **IR** (KBr, cm^{-1}) ν: 3419, 3086, 2925, 1705, 1609, 1507, 1397, 1337, 1256, 1187, 1060, 961, 723. **HRMS** (ESI) calcd for $C_{20}H_{16}F_3N_2OS^+$ m/z 389.0930 $[M+H]^+$, Found 389.0929.

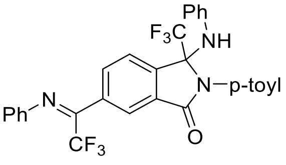

3ag

3-(phenylamino)-2-(p-tolyl)-6-(2,2,2-trifluoro-1-(phenylimino)ethyl)-3-(trifluoromethyl)isoindolin-1-one 3ag. Starting from **1ag** and *p*-tolyl isocyanate (Cas: 622-58-2), on a 0.15 mmol scale, o-xylene as solvent, 160 °C, 72 h. The product was purified by flash column chromatography on silica gel (height 16 cm, width 1.5 cm, eluent: petroleum ether/ethyl acetate, gradient: 99:1 to 97:3) as a white solid, yield = 72% (59.9 mg). R_f (petroleum ether/ethyl acetate = 91:9): 0.2. **^{1}H NMR** (400 MHz, $CDCl_3$) δ 7.93 (s, 1H), 7.53 (d, J = 7.6 Hz, 1H), 7.39 (d, J = 9.2 Hz, 1H), 7.23 – 7.14 (m, 4H), 7.11 – 6.94 (m, 5H), 6.86 (t, J = 7.6 Hz, 1H), 6.73 (d, J = 7.2 Hz, 2H), 6.19 (t,

J = 8.0 Hz, 2H), 4.69 (s, 1H), 2.35 (s, 3H). **^{19}F NMR** (376 MHz, $CDCl_3$) δ -70.8 (s), -77.4 (s). **^{13}C NMR** (101 MHz, $CDCl_3$) δ 166.1, 155.8 (q, *J* = 35.4 Hz), 146.3, 141.2, 139.9, 139.0, 133.53, 133.48, 133.1, 130.8, 130.1, 129.4, 128.9, 128.7, 126.0, 125.2, 124.9, 123.0 (q, *J* = 289.9 Hz), 121.2, 120.5, 119.5 (q, *J* = 279.8 Hz), 116.4, 80.6 (q, *J* = 29.3 Hz), 21.2. **IR** (KBr, cm^{-1}) ν: 3345, 3068, 1707, 1606, 1505, 1436, 1367, 1326, 1269, 1187, 1143, 1051, 985, 737, 686. **HRMS** (ESI) calcd for $C_{30}H_{22}F_6N_3O^+$ *m/z* 554.1662 [M+H]$^+$, Found 554.1671.

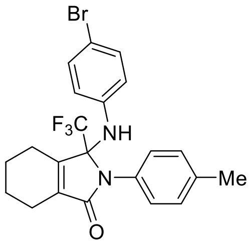

3ah

3-((4-bromophenyl)amino)-2-(p-tolyl)-3-(trifluoromethyl)-2,3,4,5,6,7-hexahydro-1H-isoindol-1-one 3ah. Starting from **1ah** and *p*-tolyl isocyanate (Cas: 622-58-2), on a 0.2 mmol scale, o-xylene as solvent, 60 h. The product was purified by flash column chromatography on silica gel (height 18 cm, width 1.5 cm, eluent: petroleum ether/ethyl acetate, gradient: 99:1 to 95:5) as a white solid, yield = 55% (50.9 mg). R_f (petroleum ether/ethyl acetate = 91:9): 0.2. **^{1}H NMR** (400 MHz, $CDCl_3$) δ 7.32 (d, *J* = 8.8 Hz, 2H), 7.14 (d, *J* = 8.0 Hz, 2H), 6.95 (d, *J* = 8.0 Hz, 2H), 6.57 (d, *J* = 8.8 Hz, 2H), 4.56 (s, 1H), 2.55 – 2.35 (m, 2H), 2.33 (s, 3H), 2.26 – 2.19 (s, 1H), 2.12 – 2.01 (m, 1H), 1.91 – 1.79 (m, 2H), 1.74 – 1.63 (m, 1H), 1.52 – 1.40 (m, 1H). **^{19}F NMR** (376 MHz, $CDCl_3$) δ -76.8 (s). **^{13}C NMR** (101 MHz, $CDCl_3$) δ 169.5, 148.6, 141.5, 139.3, 138.2, 132.4, 131.5, 129.9, 128.6, 123.0 (q, *J* = 290.9 Hz), 117.6, 113.2, 81.7 (q, *J* = 29.3 Hz), 22.6, 22.0, 21.4, 21.1, 20.8. **IR** (KBr, cm^{-1}) ν: 3451, 2929, 1698, 1602, 1501, 1399, 1353, 1259, 1167, 1068, 961, 815, 731. **HRMS** (ESI) calcd for $C_{22}H_{21}BrF_3N_2O^+$ *m/z* 465.0784 [M+H]$^+$, Found 465.0788.

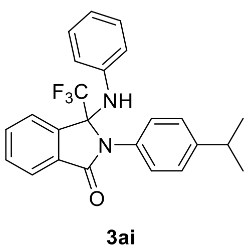

3ai

2-(4-isopropylphenyl)-3-(phenylamino)-3-(trifluoromethyl)isoindolin-1-one 3ai. Starting from **1a** and 4-isopropylphenyl isocyanate (Cas: 31027-31-3), o-xylene as solvent, 60 h. The product was purified by flash column chromatography on silica gel (height 16 cm, width 1.5 cm, eluent: petroleum ether/ethyl acetate, gradient: 99:1 to

98:2) as a pale yellow solid, yield = 75% (92.6 mg). R*f* (petroleum ether/ethyl acetate = 91:9): 0.3. **¹H NMR** (400 MHz, $CDCl_3$) *δ* 8.06 (d, *J* = 7.6 Hz, 1H), 7.71 – 7.56 (m, 3H), 7.24 (d, *J* = 8.4 Hz, 2H), 7.13 – 7.01 (m, 4H), 6.83 (t, *J* = 7.6 Hz, 1H), 6.34 (d, *J* = 8.0 Hz, 2H), 4.84 (s, 1H), 2.98 – 2.86 (m, 1H), 1.26 (d, *J* = 67.2 Hz, 6H). **¹⁹F NMR** (376 MHz, $CDCl_3$) *δ* -77.6 (s). **¹³C NMR** (101 MHz, $CDCl_3$) *δ* 167.4, 149.2, 141.7, 138.3, 132.9, 131.5, 131.1, 129.3, 128.7, 127.3, 124.70, 124.68, 124.66, 123.3 (q, *J* = 289.9 Hz), 120.8, 116.4, 80.7 (q, *J* = 29.3 Hz), 33.8, 23.79, 23.77. **IR** (KBr, cm^{-1}) ν: 3312, 3048, 2960, 2877, 1699, 1604, 1509, 1367, 1318, 1260, 1140, 1048, 827, 756. **HRMS** (ESI) calcd for $C_{24}H_{22}F_3N_2O^+$ *m/z* 411.1679 $[M+H]^+$, Found 411.1687.

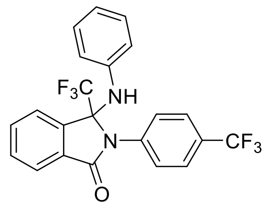

3aj

3-(phenylamino)-3-(trifluoromethyl)-2-(4-(trifluoromethyl)phenyl)isoindolin-1-one 3aj. Starting from **1a** and 4-(trifluoromethyl)phenyl isocyanate (Cas: 1548-13-6), PhCl as solvent, 48 h. The product was purified by flash column chromatography on silica gel (height 16 cm, width 1.5 cm, eluent: petroleum ether/ethyl acetate, gradient: 99:1 to 97:3) as a white solid, yield = 91% (119.2 mg). R*f* (petroleum ether/ethyl acetate = 91:9): 0.4. **¹H NMR** (400 MHz, $CDCl_3$) *δ* 8.05 (d, *J* = 6.8 Hz, 1H), 7.76 – 7.53 (m, 5H), 7.35 (d, *J* = 8.0 Hz, 2H), 7.08 (t, *J* = 8.4 Hz, 2H), 6.85 (t, *J* = 7.6 Hz, 1H), 6.37 (d, *J* = 8.0 Hz, 2H), 4.94 (s, 1H). **¹⁹F NMR** (376 MHz, $CDCl_3$) *δ* -63.2 (s), -77.6 (s). **¹³C NMR** (101 MHz, $CDCl_3$) *δ* 167.1, 141.3, 138.2, 137.7, 133.4, 132.2, 131.4, 130.4 (q, *J* = 32.3 Hz), 129.5, 129.2, 126.4 (q, *J* = 4.0 Hz), 124.8, 124.7, 123.8 (q, *J* = 273.7 Hz), 123.2 (q, *J* = 288.9 Hz), 121.2, 116.3, 81.1 (q, *J* = 29.3 Hz). **IR** (KBr, cm^{-1}) ν: 3320, 3067, 1710, 1608, 1507, 1362, 1324, 1265, 1172, 1127, 1074, 837, 724. **HRMS** (ESI) calcd for $C_{22}H_{15}F_6N_2O^+$ *m/z* 437.1083 $[M+H]^+$, Found 437.1090.

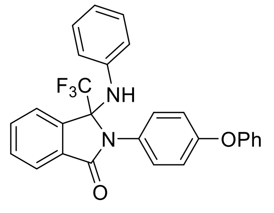

3ak

2-(4-phenoxyphenyl)-3-(phenylamino)-3-(trifluoromethyl)isoindolin-1-one 3ak. Starting from **1a** and 4-phenoxyphenyl isocyanate (Cas: 59377-19-4), PhCl as solvent, 48 h. The product was purified by flash column chromatography on silica gel (height 16 cm, width 1.5 cm, eluent: petroleum ether/ethyl acetate, gradient: 99:1 to 95:5) as a

white solid, yield = 74% (102.3 mg). R$_f$ (petroleum ether/ethyl acetate = 91:9): 0.3. **^{1}H NMR** (400 MHz, $CDCl_3$) δ 8.07 (d, J = 7.2 Hz, 1H), 7.78 – 7.55 (m, 3H), 7.35 (t, J = 8.0 Hz, 2H), 7.25 – 6.88 (m, 9H), 6.82 (t, J = 7.2 Hz, 1H), 6.36 (d, J = 8.0 Hz, 2H), 5.01 (s, 1H). **^{19}F NMR** (376 MHz, $CDCl_3$) δ -77.6 (s). **^{13}C NMR** (101 MHz, $CDCl_3$) δ 167.4, 157.6, 156.2, 141.6, 138.2, 133.0, 132.6, 131.1, 130.4, 129.8, 129.3, 128.5, 124.64, 124.60, 123.8, 123.3 (q, J = 288.9 Hz), 120.7, 119.6, 118.6, 116.2, 80.7 (q, J = 29.3 Hz). **IR** (KBr, cm^{-1}) ν: 3327, 3068, 1711, 1600, 1499, 1367, 1256, 1181, 1061, 834, 757. **HRMS** (ESI) calcd for $C_{27}H_{20}F_3N_2O_2^+$ *m/z* 461.1471 [M+H]$^+$, Found 461.1480.

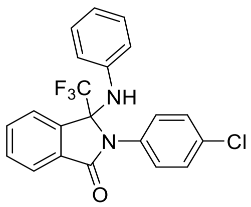

3al

2-(4-chlorophenyl)-3-(phenylamino)-3-(trifluoromethyl)isoindolin-1-one 3al. Starting from **1a** and 4-chlorophenyl isocyanate (Cas: 104-12-1), PhCl as solvent, 48 h. The product was purified by flash column chromatography on silica gel (height 16 cm, width 2.5 cm, eluent: petroleum ether/ethyl acetate, gradient: 99:1 to 95:5) as a white solid, yield = 92% (111.0 mg). R$_f$ (petroleum ether/ethyl acetate = 91:9): 0.6. **^{1}H NMR** (400 MHz, $CDCl_3$) δ 8.05 (d, J = 7.2 Hz, 1H), 7.75 – 7.52 (m, 3H), 7.36 (d, J = 8.4 Hz, 2H), 7.17 – 7.00 (m, 4H), 6.84 (t, J = 7.6 Hz, 1H), 6.33 (d, J = 8.0 Hz, 2H), 4.84 (s, 1H). **^{19}F NMR** (376 MHz, $CDCl_3$) δ -77.7 (s). **^{13}C NMR** (101 MHz, $CDCl_3$) δ 167.2, 141.4, 138.2, 134.5, 133.2, 132.7, 132.4, 131.3, 130.3, 129.54, 129.46, 124.8, 124.7, 123.2 (q, J = 288.9 Hz), 121.0, 116.3, 80.8 (q, J = 29.3 Hz). **IR** (KBr, cm^{-1}) ν: 3354, 3063, 2924, 1707, 1606, 1497, 1360, 1259, 1183, 1090, 975, 822, 720. **HRMS** (ESI) calcd for $C_{21}H_{15}ClF_3N_2O^+$ *m/z* 403.0820 [M+H]$^+$, Found 403.0825.

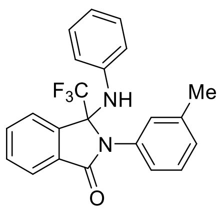

3am

3-(phenylamino)-2-(m-tolyl)-3-(trifluoromethyl)isoindolin-1-one 3am. Starting from **1a** and *m*-tolyl isocyanate (Cas: 621-29-4), PhCl as solvent, 48 h. The product was purified by flash column chromatography on silica gel (height 16 cm, width 1.5 cm, eluent: petroleum ether/ethyl acetate, gradient: 99:1 to 95:5) as a white solid,, yield = 68% (78.5 mg). R$_f$ (petroleum ether/ethyl acetate = 91:9): 0.3. **^{1}H NMR** (400 MHz,

$CDCl_3$) δ 8.03 (d, J = 7.6 Hz, 1H), 7.70 – 7.54 (m, 3H), 7.23 (t, J = 7.6 Hz, 1H), 7.16 (d, J = 7.6 Hz, 1H), 7.04 (t, J = 7.6 Hz, 2H), 6.96 (s, 1H), 6.92 (d, J = 8.0 Hz, 1H), 6.80 (t, J = 7.6 Hz, 1H), 6.30 (d, J = 7.6 Hz, 2H), 4.82 (s, 1H), 2.27 (s, 3H). **^{19}F NMR** (376 MHz, $CDCl_3$) δ -76.9 (s). **^{13}C NMR** (101 MHz, $CDCl_3$) δ 167.3, 141.7, 139.1, 138.3, 134.0, 132.9, 132.8, 131.2, 129.7, 129.4, 129.3, 129.0, 125.9, 124.71, 124.67, 123.3 (q, J = 289.9 Hz), 120.8, 116.4, 80.8 (q, J = 29.3 Hz), 21.2. **IR** (KBr, cm^{-1}) ν: 3329, 3143, 1703, 1603, 1496, 1369, 1322, 1261, 1186, 1062, 720. **HRMS** (ESI) calcd for $C_{22}H_{18}F_3N_2O^+$ *m/z* 383.1366 [M+H]$^+$, Found 383.1378.

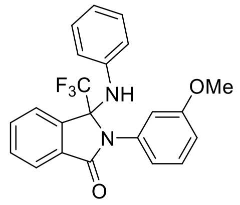

3an

2-(3-methoxyphenyl)-3-(phenylamino)-3-(trifluoromethyl)isoindolin-1-one 3an. Starting from **1a** and 3-methoxyphenyl isocyanate (Cas: 18908-07-1), o-xylene as solvent, 60 h. The product was purified by flash column chromatography on silica gel (height 16 cm, width 1.5 cm, eluent: petroleum ether/ethyl acetate, gradient: 99:1 to 95:5) as a white solid, yield = 61% (73.4 mg). R_f (petroleum ether/ethyl acetate = 91:9): 0.4. **^{1}H NMR** (400 MHz, $CDCl_3$) δ 8.06 (d, J = 7.6 Hz, 1H), 7.73 – 7.55 (m, 3H), 7.29 (t, J = 8.0 Hz, 1H), 7.06 (t, J = 8.0 Hz, 2H), 6.92 (dd, J = 8.4, 2.4 Hz, 1H), 6.85 – 6.75 (m, 2H), 6.64 (s, 1H), 6.32 (d, J = 8.0 Hz, 2H), 4.81 (s, 1H), 3.59 (s, 3H). **^{19}F NMR** (376 MHz, $CDCl_3$) δ -77.1 (s). **^{13}C NMR** (101 MHz, $CDCl_3$) δ 167.3, 160.1, 141.7, 138.3, 135.1, 133.0, 132.7, 131.3, 129.9, 129.4, 124.79, 124.76, 123.3 (q, J = 288.9 Hz), 121.3, 120.9, 116.3, 115.1, 114.1, 80.8 (q, J = 29.3 Hz), 55.0. **IR** (KBr, cm^{-1}) ν: 3439, 3328, 3087, 1702, 1604, 1498, 1370, 1261, 1180, 1044, 722. **HRMS** (ESI) calcd for $C_{22}H_{18}F_3N_2O_2^+$ *m/z* 399.1315 [M+H]$^+$, Found 399.1316.

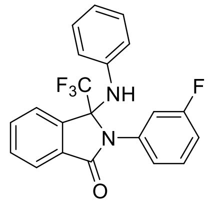

3ao

2-(3-fluorophenyl)-3-(phenylamino)-3-(trifluoromethyl)isoindolin-1-one 3ao. Starting from **1a** and 3-fluorophenyl isocyanate (Cas: 404-71-7), PhCl as solvent, 48 h. The product was purified by flash column chromatography on silica gel (height 16 cm, width 1.5 cm, eluent: petroleum ether/ethyl acetate, gradient: 99:1 to 97:3) as a white solid, yield = 90% (104.5 mg). R_f (petroleum ether/ethyl acetate = 91:9): 0.3. **^{1}H NMR**

(400 MHz, $CDCl_3$) δ 8.07 (d, *J* = 7.2 Hz, 1H), 7.74 – 7.58 (m, 3H), 7.40 – 7.32 (m, 1H), 7.14 – 7.03 (m, 3H), 7.00 – 6.78 (m, 3H), 6.33 (d, *J* = 8.0 Hz, 2H), 4.76 (s, 1H). **^{19}F NMR** (376 MHz, $CDCl_3$) δ -77.6 (s), -111.7 (s). **^{13}C NMR** (101 MHz, $CDCl_3$) δ 167.1, 162.8 (d, *J* = 248.5 Hz), 141.3, 138.2, 135.7 (d, *J* = 10.1 Hz), 133.2, 132.4, 131.3, 130.3 (d, *J* = 9.1 Hz), 129.5, 124.8, 124.7, 124.6 (d, *J* = 3.0 Hz), 123.2 (q, *J* = 289.9 Hz), 121.1, 116.4, 116.3 (d, *J* = 23.2 Hz), 115.6 (d, *J* = 21.2 Hz), 81.0 (q, *J* = 29.3 Hz). **IR** (KBr, cm^{-1}) ν: 3435, 3336, 1707, 1606, 1495, 1363, 1263, 1183, 1049, 756, 720. **HRMS** (ESI) calcd for $C_{21}H_{15}F_4N_2O^+$ *m/z* 387.1115 $[M+H]^+$, Found 387.1123.

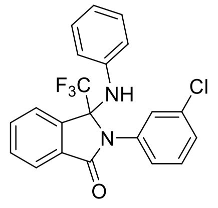

3ap

2-(3-chlorophenyl)-3-(phenylamino)-3-(trifluoromethyl)isoindolin-1-one 3ap. Starting from **1a** and 3-chlorophenyl isocyanate (Cas: 2909-38-8), PhCl as solvent, 48 h. The product was purified by flash column chromatography on silica gel (height 16 cm, width 2.5 cm, eluent: petroleum ether/ethyl acetate, gradient: 99:1 to 95:5) as a white solid, yield = 91% (109.5 mg). R_f (petroleum ether/ethyl acetate = 91:9): 0.3. **^{1}H NMR** (400 MHz, $CDCl_3$) δ 8.05 (d, *J* = 7.2 Hz, 1H), 7.74 – 7.55 (m, 3H), 7.41 – 7.27 (m, 2H), 7.20 (t, *J* = 1.6 Hz, 1H), 7.07 (t, *J* = 8.0 Hz, 3H), 6.84 (t, *J* = 7.6 Hz, 1H), 6.34 (d, *J* = 8.0 Hz, 2H), 4.88 (s, 1H). **^{19}F NMR** (376 MHz, $CDCl_3$) δ -77.6 (s). **^{13}C NMR** (101 MHz, $CDCl_3$) δ 167.1, 141.3, 138.2, 135.4, 134.7, 133.3, 132.3, 131.3, 130.2, 129.5, 129.2, 128.8, 127.5, 127.2, 124.8, 124.74, 124.73, 123.2 (q, *J* = 288.9 Hz), 121.1, 116.4, 81.0 (q, *J* = 29.3 Hz). **IR** (KBr, cm^{-1}) ν: 3331, 3144, 3093, 1703, 1603, 1486, 1366, 1323, 1260, 1186, 1048, 969, 899, 723. **HRMS** (ESI) calcd for $C_{21}H_{15}ClF_3N_2O^+$ *m/z* 403.0820 $[M+H]^+$, Found 403.0821.

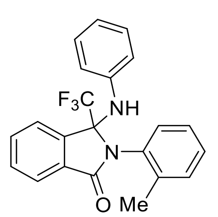

3aq

3-(phenylamino)-2-(o-tolyl)-3-(trifluoromethyl)isoindolin-1-one 3aq. Starting from **1a** and *o*-tolyl isocyanate (Cas: 614-68-6), o-xylene as solvent, 60 h. The product was purified by flash column chromatography on silica gel (height 20 cm, width 1.5 cm, eluent: petroleum ether/ethyl acetate, gradient: 99:1 to 97:3) as a white solid, yield = 36% (40.9 mg). R_f (petroleum ether/ethyl acetate = 91:9): 0.4. **^{1}H NMR** (400 MHz,

$CDCl_3$) δ 8.06 (d, J = 7.2 Hz, 1H), 7.73 – 7.50 (m, 3H), 7.36 (d, J = 7.6 Hz, 1H), 7.27 (t, J = 7.6 Hz, 1H), 7.06 (q, J = 8.0 Hz, 3H), 6.92 – 6.76 (m, 2H), 6.31 (d, J = 8.0 Hz, 2H), 4.79 (s, 1H), 2.35 (s, 3H). **^{19}F NMR** (376 MHz, $CDCl_3$) δ -77.2 (s). **^{13}C NMR** (101 MHz, $CDCl_3$) δ 166.4, 141.7, 138.4, 138.3, 133.0, 132.9, 132.6, 131.6, 131.2, 129.4, 128.6, 126.4, 124.8, 124.4, 123.6 (q, J = 288.9 Hz), 120.8, 116.6, 80.5 (q, J = 29.3 Hz), 18.1, one carbon is overlapped. **IR** (KBr, cm^{-1}) ν: 3440, 3334, 3089, 2929, 1702, 1608, 1497, 1362, 1265, 1182, 1045, 974, 758, 722. **HRMS** (ESI) calcd for $C_{22}H_{18}F_3N_2O^+$ *m/z* 383.1366 [M+H]$^+$, Found 383.1368.

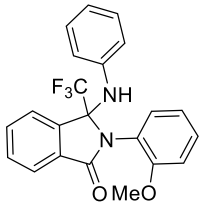

3ar

2-(2-methoxyphenyl)-3-(phenylamino)-3-(trifluoromethyl)isoindolin-1-one 3ar. Starting from **1a** and 2-methoxyphenyl isocyanate (Cas: 700-87-8), PhCl as solvent, 48 h. The product was purified by flash column chromatography on silica gel (height 16 cm, width 1.5 cm, eluent: petroleum ether/ethyl acetate, gradient: 99:1 to 95:5) as a white solid, yield = 81% (96.4 mg, **mixture of isomer, ratio = 1:0.30**). R_f (petroleum ether/ethyl acetate = 91:9): 0.1. **^{1}H NMR** (400 MHz, $CDCl_3$) δ 8.06 (d, J = 7.2 Hz, 1.30H), 7.74 – 7.51 (m, 3.90H), 7.45 – 7.30 (m, 1.60H), 7.13 – 6.64 (m, 7.50H), 6.41 – 6.08 (m, 2.60H), 4.74 (s, 1H), 4.60 (s, 0.30H), 3.85 (s, 3H), 3.02 (s, 0.90H). **^{19}F NMR** (376 MHz, $CDCl_3$) δ -77.1 (s, minor), -78.3 (s, major). **^{13}C NMR** (101 MHz, $CDCl_3$, major) δ 166.6, 157.1, 141.9, 138.8, 132.8, 132.7, 131.0, 130.2, 130.1, 129.4, 124.7, 124.3, 123.4 (q, J = 289.9 Hz), 122.8, 120.6, 120.4, 116.2, 112.3, 80.3 (q, J = 29.3 Hz), 55.8. **IR** (KBr, cm^{-1}) ν: 3309, 3072, 1706, 1604, 1501, 1370, 1266, 1177, 1042, 880, 755. **HRMS** (ESI) calcd for $C_{22}H_{18}F_3N_2O_2^+$ *m/z* 399.1315 [M+H]$^+$, Found 399.1318.

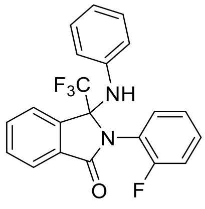

3as

2-(2-fluorophenyl)-3-(phenylamino)-3-(trifluoromethyl)isoindolin-1-one 3as. Starting from **1a** and 2-fluorophenyl isocyanate (Cas: 16744-98-2), o-xylene as solvent, 60 h. The product was purified by flash column chromatography on silica gel (height 16 cm, width 1.5 cm, eluent: petroleum ether/ethyl acetate, gradient: 99:1 to 98:2) as a

white solid, yield = 99% (115.0 mg). R$_f$ (petroleum ether/ethyl acetate = 91:9): 0.3. **^{1}H NMR** (400 MHz, CDCl$_3$) δ 8.07 (d, J = 7.2 Hz, 1H), 7.72 – 7.54 (m, 3H), 7.42 – 7.32 (m, 1H), 7.23 (t, J = 8.8 Hz, 1H), 7.12 – 6.90 (m, 4H), 6.83 (t, J = 7.6 Hz, 1H), 6.34 (d, J = 8.0 Hz, 2H), 4.78 (s, 1H). **^{19}F NMR** (376 MHz, CDCl$_3$) δ -78.6 (s), -117.4 (s). **^{13}C NMR** (101 MHz, CDCl$_3$) δ 166.5, 159.5 (d, J = 254.5 Hz), 141.5, 138.5, 133.1, 132.2, 131.2, 130.6, 130.5, 129.3, 124.8, 124.5, 124.4 (d, J = 3.0 Hz), 123.2 (q, J = 289.9 Hz), 121.8 (d, J = 13.1 Hz), 120.8, 116.7 (d, J = 20.2 Hz), 116.3, 80.5 (q, J = 29.3 Hz). **IR** (KBr, cm^{-1}) ν: 3336, 3085, 1712, 1606, 1502, 1367, 1264, 1182, 1048, 879, 758. **HRMS** (ESI) calcd for $C_{21}H_{15}F_4N_2O^+$ *m/z* 387.1115 [M+H]$^+$, Found 387.1122.

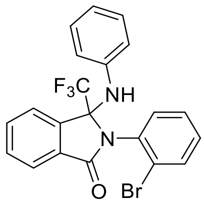

3at

2-(2-bromophenyl)-3-(phenylamino)-3-(trifluoromethyl)isoindolin-1-one 3at. Starting from **1a** and 2-bromophenyl isocyanate (Cas: 1592-00-3), PhCl as solvent, 48 h. The product was purified by flash column chromatography on silica gel (height 16 cm, width 1.5 cm, eluent: petroleum ether/ethyl acetate, gradient: 99:1 to 95:5) as a white solid, yield = 76% (101.6 mg). R$_f$ (petroleum ether/ethyl acetate = 91:9): 0.2. **^{1}H NMR** (400 MHz, CDCl$_3$) δ 8.07 (d, J = 7.2 Hz, 1H), 7.77 (dd, J = 7.6, 1.6 Hz, 1H), 7.70 – 7.50 (m, 3H), 7.25 – 7.12 (m, 2H), 7.11 – 6.90 (m, 3H), 6.82 (t, J = 7.6 Hz, 1H), 6.32 (d, J = 8.0 Hz, 2H), 4.88 (s, 1H). **^{19}F NMR** (376 MHz, CDCl$_3$) δ -76.7 (s). **^{13}C NMR** (101 MHz, CDCl$_3$) δ 165.8, 141.5, 138.3, 134.3, 133.7, 133.2, 132.2, 131.2, 130.6, 129.8, 129.5, 127.9, 124.9, 124.8, 124.2, 123.3 (q, J = 288.9 Hz), 120.8, 116.3, 80.6 (q, J = 30.3 Hz). **IR** (KBr, cm^{-1}) ν: 3306, 3071, 1709, 1605, 1477, 1364, 1267, 1180, 1067, 882, 758, 721. **HRMS** (ESI) calcd for $C_{21}H_{15}BrF_3N_2O^+$ *m/z* 447.0314 [M+H]$^+$, Found 447.0320.

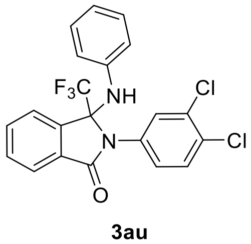

3au

2-(3,4-dichlorophenyl)-3-(phenylamino)-3-(trifluoromethyl)isoindolin-1-one 3au. Starting from **1a** and 3,4-dichlorophenyl isocyanate (Cas: 102-36-3), PhCl as solvent, 48 h. The product was purified by flash column chromatography on silica gel (height 16 cm, width 1.5 cm, eluent: petroleum ether/ethyl acetate, gradient: 99:1 to 97:3) as a

white solid, yield = 91% (119.7 mg). R*f* (petroleum ether/ethyl acetate = 91:9): 0.4. **^{1}H NMR** (400 MHz, $CDCl_3$) δ 8.03 (d, *J* = 7.2 Hz, 1H), 7.78 – 7.55 (m, 3H), 7.45 (d, *J* = 8.4 Hz, 1H), 7.30 (s, 1H), 7.17 – 6.96 (m, 3H), 6.85 (t, *J* = 7.6 Hz, 1H), 6.33 (d, *J* = 8.4 Hz, 2H), 4.94 (s, 1H). **^{19}F NMR** (376 MHz, $CDCl_3$) δ -77.5 (s). **^{13}C NMR** (101 MHz, $CDCl_3$) δ 167.0, 141.2, 138.2, 133.7, 133.4, 133.1, 132.9, 132.1, 131.4, 130.9, 130.9, 129.5, 128.3, 124.8, 124.7, 123.2 (q, *J* = 288.9 Hz), 121.2, 116.3, 80.98 (q, *J* = 29.3 Hz). **IR** (KBr, cm^{-1}) ν: 3340, 3061, 1713, 1603, 1472, 1359, 1260, 1186, 1049, 970, 893, 756, 716. **HRMS** (ESI) calcd for $C_{21}H_{14}Cl_2F_3N_2O^+$ *m/z* 437.0430 $[M+H]^+$, Found 437.0437.

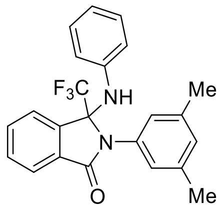

3av

2-(3,5-dimethylphenyl)-3-(phenylamino)-3-(trifluoromethyl)isoindolin-1-one 3av. Starting from **1a** and 3,5-dimethylphenyl isocyanate (Cas: 54132-75-1), PhCl as solvent, 48 h. The product was purified by flash column chromatography on silica gel (height 16 cm, width 1.5 cm, eluent: petroleum ether/ethyl acetate, gradient: 99:1 to 97:3) as a white solid, yield = 83% (98.5 mg). R*f* (petroleum ether/ethyl acetate = 91:9): 0.4. **^{1}H NMR** (400 MHz, $CDCl_3$) δ 8.06 (d, *J* = 7.6 Hz, 1H), 7.74 – 7.57 (m, 3H), 7.06 (t, *J* = 8.0 Hz, 2H), 7.00 (s, 1H), 6.83 (t, *J* = 7.6 Hz, 1H), 6.71 (s, 2H), 6.28 (d, *J* = 8.4 Hz, 2H), 4.71 (s, 1H), 2.23 (s, 6H). **^{19}F NMR** (376 MHz, $CDCl_3$) δ -77.5 (s). **^{13}C NMR** (101 MHz, $CDCl_3$) δ 167.5, 141.8, 138.8, 138.5, 133.8, 132.9, 132.8, 131.2, 130.4, 129.3, 126.8, 124.8, 124.7, 123.4 (q, *J* = 288.9 Hz), 120.9, 116.5, 80.8 (q, *J* = 30.3 Hz), 21.2. **IR** (KBr, cm^{-1}) ν: 3329, 3084, 2919, 1700, 1604, 1471, 1369, 1264, 1185, 1074, 836, 754, 722, 685. **HRMS** (ESI) calcd for $C_{23}H_{20}F_3N_2O^+$ *m/z* 397.1522 $[M+H]^+$, Found 397.1527.

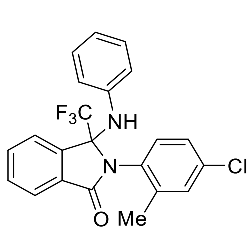

3aw

2-(4-chloro-2-methylphenyl)-3-(phenylamino)-3-(trifluoromethyl)isoindolin-1-one 3aw. Starting from **1a** and 4-chloro-1-isocyanato-2-methylbenzene (Cas: 37408-18-7), o-xylene as solvent, 60 h. The product was purified by flash column chromatography on silica gel (height 16 cm, width 1.5 cm, eluent: petroleum ether/ethyl

acetate, gradient: 99:1 to 97:3) as a white solid, yield = 74% (93.1 mg). R*f* (petroleum ether/ethyl acetate = 91:9): 0.4. **^{1}H NMR** (400 MHz, $CDCl_3$) δ 8.06 (d, J = 7.6 Hz, 1H), 7.72 – 7.52 (m, 3H), 7.35 (d, J = 2.4 Hz, 1H), 7.12 –6.95 (m, 3H), 6.87 – 6.75 (m, 2H), 6.29 (d, J = 7.6 Hz, 2H), 4.73 (s, 1H), 2.32 (s, 3H). **^{19}F NMR** (376 MHz, $CDCl_3$) δ -77.7 (s). **^{13}C NMR** (101 MHz, $CDCl_3$) δ 166.3, 141.5, 140.4, 138.2, 134.2, 133.1, 132.3, 131.6, 131.4, 131.3, 129.9, 129.5, 126.5, 124.7, 124.4, 123.5 (q, J = 288.9 Hz), 120.9, 116.4, 80.5 (q, J = 30.3 Hz), 18.0. **IR** (KBr, cm^{-1}) ν: 3326, 3060, 2973, 1706, 1605, 1490, 1361, 1266, 1176, 1089, 1041, 878, 723. **HRMS** (ESI) calcd for $C_{22}H_{17}ClF_3N_2O^+$ *m/z* 417.0976 [M+H]$^+$, Found 417.0982.

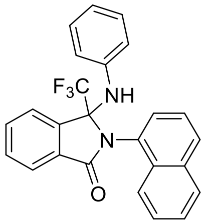

3ax

2-(naphthalen-1-yl)-3-(phenylamino)-3-(trifluoromethyl)isoindolin-1-one 3ax. Starting from **1a** and 1-naphthyl isocyanate (Cas: 86-84-0), o-xylene as solvent, 60 h. The product was purified by flash column chromatography on silica gel (height 16 cm, width 1.5 cm, eluent: petroleum ether/ethyl acetate, gradient: 99:1 to 95:5) as a white solid, yield = 68% (85.4 mg). R*f* (petroleum ether/ethyl acetate = 91:9): 0.4. **^{1}H NMR** (400 MHz, $CDCl_3$) δ 8.12 (d, J = 7.6 Hz, 1H), 8.05 – 7.80 (m, 3H), 7.75 – 7.49 (m, 5H), 7.33 (t, J = 8.0 Hz, 1H), 7.11 (dd, J = 14.4, 8.0 Hz, 3H), 6.87 (t, J = 7.6 Hz, 1H), 6.39 (d, J = 8.0 Hz, 2H), 4.93 (s, 1H). **^{19}F NMR** (376 MHz, $CDCl_3$) δ -76.7. **^{13}C NMR** (101 MHz, $CDCl_3$) δ 180.9, 167.2, 141.7, 138.4, 134.6, 133.1, 132.5, 131.3, 131.1, 130.9, 129.5, 128.4, 126.7, 126.6, 126.2, 125.1, 124.9, 124.6, 123.8, 123.5 (q, J = 288.9 Hz), 120.9, 116.6, 80.9 (q, J = 29.3 Hz). **IR** (KBr, cm^{-1}) ν: 3328, 3056, 2926, 1697, 1606, 1501, 1359, 1265, 1180, 1087, 872, 764. **HRMS** (ESI) calcd for $C_{25}H_{18}F_3N_2O^+$ *m/z* 419.1366 [M+H]$^+$, Found 419.1367.

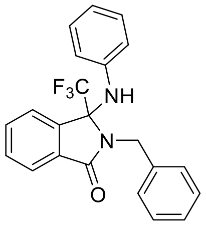

3ay

2-benzyl-3-(phenylamino)-3-(trifluoromethyl)isoindolin-1-one 3ay. Starting from **1a** and benzyl isocyanate (Cas: 3173-56-6), o-xylene as solvent, 60 h. The product was

purified by flash column chromatography on silica gel (height 16 cm, width 1.5 cm, eluent: petroleum ether/ethyl acetate, gradient: 99:1 to 95:5) as a white solid, yield = 89% (102.3 mg). R_f (petroleum ether/ethyl acetate = 91:9): 0.3. **^{1}H NMR** (400 MHz, $CDCl_3$) δ 8.03 (d, J = 7.6 Hz, 1H), 7.68 – 7.51 (m, 3H), 7.36 – 7.09 (m, 5H), 6.92 (t, J = 7.6 Hz, 2H), 6.74 (t, J = 7.2 Hz, 1H), 6.14 (d, J = 8.0 Hz, 2H), 4.90 (s, 1H), 4.78 (s, 2H). **^{19}F NMR** (376 MHz, $CDCl_3$) δ -77.6. **^{13}C NMR** (101 MHz, $CDCl_3$) δ 168.6, 141.4, 139.1, 136.4, 132.7, 132.6, 131.0, 128.9, 128.0, 127.8, 127.0, 124.3, 124.1, 123.5 (q, J = 289.9 Hz), 120.6, 116.2, 79.6 (q, J = 29.3 Hz), 43.4. **IR** (KBr, cm^{-1}) ν: 3325, 3062, 2931, 1697, 1605, 1500, 1389, 1265, 1182, 1085, 976, 735, 695. **HRMS** (ESI) calcd for $C_{22}H_{18}F_3N_2O^+$ *m/z* 383.1366 $[M+H]^+$, Found 383.1378.

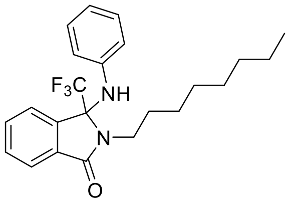

3az

2-octyl-3-(phenylamino)-3-(trifluoromethyl)isoindolin-1-one 3az. Starting from **1a** and 1-octyl isocyanate (Cas: 3158-26-7), o-xylene as solvent, 60 h. The product was purified by flash column chromatography on silica gel (height 16 cm, width 1.5 cm, eluent: petroleum ether/ethyl acetate, gradient: 99:1 to 95:5) as a white solid, yield = 86% (104.6 mg). R_f (petroleum ether/ethyl acetate = 91:9): 0.5. **^{1}H NMR** (400 MHz, $CDCl_3$) δ 7.92 (d, J = 7.2 Hz, 1H), 7.63 – 7.48 (m, 3H), 6.98 (t, J = 8.4 Hz, 2H), 6.76 (t, J = 7.2 Hz, 1H), 6.22 (d, J = 8.0 Hz, 2H), 4.74 (s, 1H), 3.47 – 3.31 (m, 2H), 1.77 – 1.52 (m, 2H), 1.36 – 1.13 (m, 10H), 0.86 (t, J = 6.8 Hz, 3H). **^{19}F NMR** (376 MHz, $CDCl_3$) δ -78.3. **^{13}C NMR** (101 MHz, $CDCl_3$) δ 168.2, 141.5, 139.1, 133.2, 132.4, 130.9, 129.2, 124.2, 123.9, 123.8 (q, J = 288.9 Hz), 120.9, 116.4, 79.4 (q, J = 30.3 Hz), 40.3, 31.8, 29.11, 29.10, 27.7, 27.3, 22.6, 14.1. **IR** (KBr, cm^{-1}) ν: 3322, 3074, 2929, 1695, 1605, 1499, 1379, 1321, 1259, 1165, 1079, 736. **HRMS** (ESI) calcd for $C_{23}H_{28}F_3N_2O^+$ *m/z* 405.2148 $[M+H]^+$, Found 405.2152.

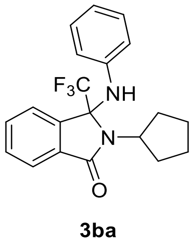

3ba

2-cyclopentyl-3-(phenylamino)-3-(trifluoromethyl)isoindolin-1-one 3ba. Starting from **1a** and cyclopentyl isocyanate (Cas: 4747-71-1), o-xylene as solvent, 60 h. The product was purified by flash column chromatography on silica gel (height 16 cm,

width 1.5 cm, eluent: petroleum ether/ethyl acetate, gradient: 99:1 to 98:2) as a white solid, yield = 61% (66.4 mg). R_f (petroleum ether/ethyl acetate = 91:9): 0.4. **^{1}H NMR** (400 MHz, $CDCl_3$) δ 7.90 (d, J = 7.6 Hz, 1H), 7.63 – 7.48 (m, 3H), 7.03 – 6.93 (m, 2H), 6.76 (t, J = 7.6 Hz, 1H), 6.27 (dd, J = 7.6, 1.6 Hz, 2H), 4.93 – 4.71 (m, 1H), 3.97 – 3.79 (m, 1H), 2.40 – 2.20 (m, 1H), 2.16 – 1.76 (m, 4H), 1.64 – 1.34 (m, 2H), 1.30 – 1.17 (m, 1H). **^{19}F NMR** (376 MHz, $CDCl_3$) δ -78.8 (s). **^{13}C NMR** (101 MHz, $CDCl_3$) δ 167.2, 141.6, 139.0, 133.8, 132.2, 130.9, 129.1, 124.1, 123.8 (q, J = 288.9 Hz), 123.6, 120.8, 116.2, 79.6 (q, J = 30.3 Hz), 53.4, 29.1, 28.5, 25.2, 25.1. **IR** (KBr, cm^{-1}) ν: 3319, 3062, 2955, 1687, 1608, 1502, 1357, 1258, 1166, 1078, 721. **HRMS** (ESI) calcd for $C_{20}H_{20}F_3N_2O^+$ *m/z* 361.1522 $[M+H]^+$, Found 361.1529

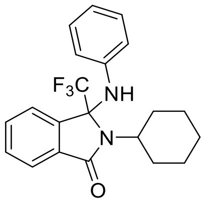

3bb

2-cyclohexyl-3-(phenylamino)-3-(trifluoromethyl)isoindolin-1-one 3bb. Starting from **1a** and cyclohexyl isocyanate (Cas: 3173-53-3), o-xylene as solvent, 60 h. The product was purified by flash column chromatography on silica gel (height 16 cm, width 1.5 cm, eluent: petroleum ether/ethyl acetate, gradient: 99:1 to 98:2) as a white solid, yield = 69% (77.1 mg). R_f (petroleum ether/ethyl acetate = 91:9): 0.5. **^{1}H NMR** (400 MHz, $CDCl_3$) δ 7.90 (d, J = 7.2 Hz, 1H), 7.65 – 7.40 (m, 3H), 6.98 (t, J = 7.6 Hz, 2H), 6.75 (t, J = 7.6 Hz, 1H), 6.25 (d, J = 8.4 Hz, 2H), 4.84 (s, 1H), 3.43 – 3.24 (m, 1H), 2.53 – 2.19 (m, 2H), 1.93 – 1.52 (m, 4H), 1.33 – 0.99 (m, 4H). **^{19}F NMR** (376 MHz, $CDCl_3$) δ -78.4 (s). **^{13}C NMR** (101 MHz, $CDCl_3$) δ 167.5, 141.8, 138.6, 133.9, 132.2, 130.9, 129.0, 124.2, 123.8 (q, J = 288.9 Hz), 123.7, 120.7, 116.2, 79.4 (q, J = 30.3 Hz), 53.6, 29.0, 28.8, 26.4, 26.4, 25.2. **IR** (KBr, cm^{-1}) ν: 3316, 3080, 2860, 1687, 1607, 1501, 1413, 1358, 1322, 1260, 1185, 1072, 980, 896, 721. **HRMS** (ESI) calcd for $C_{21}H_{22}F_3N_2O^+$ *m/z* 375.1679 $[M+H]^+$, Found 375.1684.

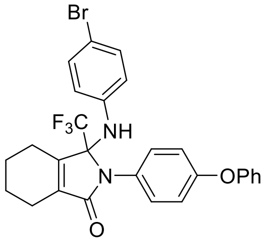

3bc

3-((4-bromophenyl)amino)-2-(4-phenoxyphenyl)-3-(trifluoromethyl)-2,3,4,5,6,7-hexahydro-1H-isoindol-1-one 3bc. Starting from **1ah** and 4-phenoxyphenyl

isocyanate (Cas: 59377-19-4), on a 0.2 mmol scale, o-xylene as solvent, 60 h. The product was purified by flash column chromatography on silica gel (height 18 cm, width 1.5 cm, eluent: petroleum ether/ethyl acetate, gradient: 99:1 to 95:5) as a white solid, yield = 49% (53.5 mg). R_f (petroleum ether/ethyl acetate = 91:9): 0.2. **^{1}H NMR** (400 MHz, $CDCl_3$) δ 7.40 – 7.28 (m, 4H), 7.12 (t, J = 7.2 Hz, 1H), 7.02 (d, J = 7.6 Hz, 4H), 6.93 (d, J = 8.8 Hz, 2H), 6.57 (d, J = 8.8 Hz, 2H), 4.59 (s, 1H), 2.55 – 2.20 (m, 3H), 2.14 – 1.99 (m, 1H), 1.93 – 1.79 (m, 2H), 1.76 – 1.60 (m, 1H), 1.54 – 1.40 (m, 1H). **^{19}F NMR** (376 MHz, $CDCl_3$) δ -76.8 (s). **^{13}C NMR** (101 MHz, $CDCl_3$) δ 169.5, 157.4, 156.3, 148.8, 141.4, 139.2, 132.5, 130.2, 129.8, 128.8, 123.9, 122.9 (q, J = 290.9 Hz), 119.6, 118.7, 117.5, 113.3, 81.7 (q, J = 29.3 Hz), 22.6, 22.0, 21.4, 20.8. **IR** (KBr, cm^{-1}) ν: 3444, 3313, 2934, 1696, 1595, 1497, 1398, 1254, 1234, 1165, 1120, 1064, 872, 822, 736, 698. **HRMS** (ESI) calcd for $C_{27}H_{23}BrF_3N_2O_2^+$ *m/z* 543.0890 $[M+H]^+$, Found 543.0897.

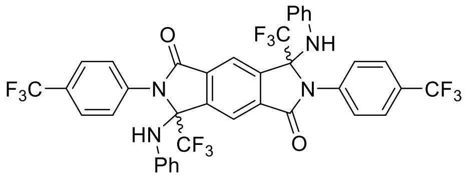

3bd

3,7-bis(phenylamino)-3,7-bis(trifluoromethyl)-2,6-bis(4-(trifluoromethyl)phenyl)-2,3,6,7-tetrahydropyrrolo[3,4-f]isoindole-1,5-dione 3bd. Starting from **1ag** and 4-(trifluoromethyl)phenyl isocyanate (Cas: 1548-13-6), on a 0.15 mmol scale, o-xylene as solvent, 160 °C, 72 h. The product was purified by flash column chromatography on silica gel (height 18 cm, width 1.5 cm, eluent: petroleum ether/ethyl acetate, gradient: 99:1 to 95:5) as a white solid, yield = 34% (40.1 mg). R_f (petroleum ether/ethyl acetate = 91:9): 0.2. **^{1}H NMR** (400 MHz, $CDCl_3$) δ 8.25 (d, J = 5.2 Hz, 2H), 7.68 (d, J = 7.2 Hz, 4H), 7.38 – 7.27 (m, 4H), 7.19 – 7.03 (m, 4H), 6.99 – 6.87 (m, 2H), 6.35 (dd, J = 22.4, 7.6 Hz, 4H), 4.86 (d, J = 5.6 Hz, 2H). **^{19}F NMR** (376 MHz, $CDCl_3$) δ -63.3 (s), -63.4 (s), -77.0 (s), -77.4 (s). **^{13}C NMR** (101 MHz, $CDCl_3$) δ 165.08, 165.06, 141.84, 141.79, 140.6, 140.5, 137.2, 137.1, 136.93, 136.92, 131.0 (q, J = 32.3 Hz), 131.0 (q, J = 33.3 Hz), 129.82, 129.79, 129.02, 129.00, 126.7 (q, J = 3.0 Hz), 123.6 (q, J = 273.7 Hz), 122.8 (q, J = 288.9 Hz), 122.0, 121.9, 121.7, 121.6, 116.4, 81.2 (q, J = 30.3 Hz), 81.1 (q, J = 29.3 Hz). **IR** (KBr, cm^{-1}) ν: 3370, 3064, 2927, 1720, 1612, 1509, 1328, 1260, 1175, 1064, 985, 825, 742. **HRMS** (ESI) calcd for $C_{38}H_{23}F_{12}N_4O_2^+$ *m/z* 795.1624 $[M+H]^+$, Found 795.1627.

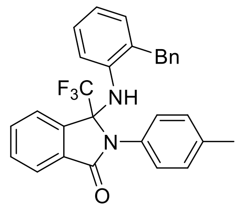

3be

3-((2-benzylphenyl)amino)-2-(p-tolyl)-3-(trifluoromethyl)isoindolin-1-one 3be. Starting from **1ai** and *p*-tolyl isocyanate (Cas: 622-58-2), on a 0.2 mmol scale, o-xylene as solvent, 150 °C, 60 h. The product was purified by flash column chromatography on silica gel (height 16 cm, width 1.5 cm, eluent: petroleum ether/ethyl acetate, gradient: 98:2 to 95:5) as a white solid, yield = 45% (42.7 mg). R_f (petroleum ether/ethyl acetate = 91:9): 0.2. **^{1}H NMR** (400 MHz, $CDCl_3$) δ 8.03 (d, J = 7.6 Hz, 1H), 7.62 (t, J = 7.6 Hz, 1H), 7.54 (t, J = 7.6 Hz, 1H), 7.34 (d, J = 7.6 Hz, 1H), 7.26 – 7.10 (m, 6H), 7.03 (d, J = 8.0 Hz, 2H), 6.91 – 6.73 (m, 4H), 5.97 (d, J = 7.6 Hz, 1H), 4.58 (s, 1H), 3.99 (s, 2H), 2.34 (s, 3H). **^{19}F NMR** (376 MHz, $CDCl_3$) δ -77.8 (s). **^{13}C NMR** (101 MHz, $CDCl_3$) δ 167.2, 139.9, 138.3, 137.99, 137.95, 133.0, 132.8, 131.2, 131.1, 131.0, 129.8, 128.8, 128.5, 128.3, 128.1, 127.7, 126.6, 124.6, 124.5, 123.2 (q, J = 289.9 Hz), 120.8, 116.0, 80.6 (q, J = 29.3 Hz), 39.0, 21.1. **IR** (KBr, cm^{-1}) ν: 3455, 3361, 2922, 1701, 1600, 1520, 1457, 1364, 1260, 1176, 1134, 1045, 811, 710. **HRMS** (ESI) calcd for $C_{29}H_{24}F_3N_2O^+$ *m/z* 473.1835 [M+H]$^+$, Found 473.1843.

Mechanistic studies

Procedure for the synthesis of the deuterated ketimine [D]$_5$-1a (Scheme S8)

To a solution of 1-bromobenzene-2,3,4,5,6-d$_5$ (2.63 mL, 25 mmol, 1 equiv) in dry Et_2O (70 mL) at -78 °C was slowly added *n*-BuLi (2.5 M in hexane, 1.1 equiv), and then reaction mixture was warmed to 0 °C and stirred at that temperature for 3 h. After that, the reaction mixture was cooled down to -60 °C and a solution of *N*-trifluoroacetylpiperidine (4.4 mL, 30 mmol, 1.2 equiv) in dry Et_2O (10 mL) was added in portions. The reaction mixture was allowed to stir at -60 °C for 3 h and then warmed to room temperature. The reaction mixture was then quenched by the addition of the saturated aqueous NH_4Cl (50 mL) and the organic layer was subsequently washed with saturated aqueous NH_4Cl (5 × 30 mL) and H_2O (3 × 30 mL). The combined organic layer was dried over Na_2SO_4, filtered and concentrated. The residue was dissolved in DCM and quickly passed through a short silica gel column to give the crude 2,2,2-trifluoro-1-(phenyl-d$_5$)ethan-1-one as a colorless oil (eluent: petroleum ether).

To a solution of the obtained 2,2,2-trifluoro-1-(phenyl-d$_5$)ethan-1-one (4.48 g, 25 mmol, 1 equiv) in toluene (90 mL) was added aniline (4.6 mL, 50 mmol, 2 equiv) followed by

p-toluenesulfonic acid monohydrate (0.95 g, 5 mmol, 20 mol%). The reaction mixture was heated at 140 °C for 48 h with removal of water *via* Dean-Stark trap. After cooling to room temperature, the reaction mixture was concentrated under vacuum, purification by column chromatography on silica gel (eluent: petroleum ether + 5% Et_3N) afforded **[D]$_5$-1a** (overall yield 20 %, 1.29 g) as yellow oil. **^{1}H NMR** (400 MHz, $CDCl_3$) δ 7.22 (t, J = 7.6 Hz, 2H), 7.07 (t, J = 7.6 Hz, 1H), 6.79 (d, J = 8.0 Hz, 2H). **^{19}F NMR** (376 MHz, $CDCl_3$) δ -70.5 (s). **HRMS** (ESI) calcd for $C_{14}H_6D_5F_3N^+$ *m/z* 255.1152 [M+H]$^+$, Found 255.1163.

Kinetic Isotope Effect (KIE) measurements (Scheme S9)

Five reactions were performed for different reaction time (1 h, 2 h, 3 h, 4 h, 5 h). An oven-dried 25 mL schlenk tube equipped with a stirring bar was transferred into a glovebox (through standard glovebox operation), where $Re_2(CO)_{10}$ (0.03 mmol, 19.6 mg, 0.1 equiv) was added. The tube was then removed from the glovebox and placed under Ar. Then the degassed o-xylene (3 mL), 2,2,2-trifluoro-*N*,1-diphenylethan-1-imine **1a** (74.8 mg, 0.3 mmol, 1 equiv) or 1-(cyclohexa-1,5-dien-1-yl-2,3,4,5,6-d$_5$)-2,2,2-trifluoro-*N*-phenylethan-1-imine **[D]$_5$-1a**, 1-isocyanato-4-methylbenzene **2a** (79.9 mg, 0.6 mmol, 2 equiv) were added under Ar. The resulting reaction mixture was then stirred at 150 ℃ for different reaction time. The yield of each reaction was determined by ^{19}F NMR analysis of the reaction mixture (Table S1). The parallel reactions provided a KIE value: k_H/k_D = 0.8

Control experiments

The reaction were carried out under different additives following general procedure (Scheme S10):

Reaction condition 1: **1a** (0.3 mmol), **2a** (0.6 mmol), $Re_2(CO)_{10}$ (0.03 mmol), in o-xylene, 150 °C, 60 h, under O_2 (1 atm). **3a** was not obtained.

Reaction condition 2: **1a** (0.3 mmol), **2a** (0.6 mmol), $Re_2(CO)_{10}$ (0.03 mmol), TEMPO (0.3 mmol), in o-xylene, 150 °C, 60 h, under Ar. **3a** was not obtained.

Reaction condition 3: **1a** (0.3 mmol), **2a** (0.6 mmol), $Re_2(CO)_{10}$ (0.03 mmol), in o-xylene, 150 °C, 60 h, under CO (1 atm). **3a** was not obtained.

Reaction condition 4: **1a** (0.3 mmol), **2a** (0.6 mmol), $Re_2(CO)_{10}$ (0.03 mmol), Et_3N (0.06 mmol), in o-xylene, 150 °C, 60 h, under Ar. **3a** was obtained in 85% yield.

Reaction condition 5: **1a** (0.3 mmol), **2a** (0.6 mmol), $Re_2(CO)_{10}$ (0.03 mmol), Na_2CO_3 (0.06 mmol), in o-xylene, 150 °C, 60 h, under Ar. **3a** was obtained in 68% yield.

Reaction condition 6: **1a** (0.3 mmol), **2a** (0.6 mmol), $Re_2(CO)_{10}$ (0.03 mmol), NaOAc (0.06 mmol), in o-xylene, 150 °C, 60 h, under Ar. **3a** was obtained in 67% yield.

The reaction was carried out use 1aj instead of 1a following general procedure G, 3bf was obtained (Scheme S11):

3-methylene-2-(p-tolyl)isoindolin-1-one 3bf. starting from **1aj** and **2a**, on a 0.2 mmol scale, o-xylene as solvent, 150 °C, 48 h. The product was purified by flash column chromatography on silica gel (height 20 cm, width 1.5 cm, eluent: petroleum ether/ethyl acetate, gradient: 98:2 to 97:3) as a white solid, yield = 80% (37.8 mg). R_f (petroleum ether/ethyl acetate = 91:9): 0.5. **^{1}H NMR** (400 MHz, $CDCl_3$) δ 7.91 (d, J = 7.6 Hz, 1H), 7.74 (d, J = 7.6 Hz, 1H), 7.61 (t, J = 7.6 Hz, 1H), 7.53 (t, J = 7.6 Hz, 1H), 7.34 – 7.21 (m, 4H), 5.20 (s, 1H), 4.77 (s, 1H), 2.41 (s, 3H). **^{13}C NMR** (101 MHz, $CDCl_3$) δ 166.6, 143.2, 137.9, 136.2, 132.1, 131.8, 129.9, 129.6, 128.9, 127.8, 123.4, 119.9, 90.2, 21.1. **IR** (KBr, cm^{-1}) ν: 3415, 3035, 2916, 1713, 1639, 1509, 1463, 1380, 1295, 1193, 1127, 1018, 821, 768, 695. **HRMS** (ESI) calcd for $C_{16}H_{14}NO^+$ *m/z* 236.1070 $[M+H]^+$, Found 236.1076.

Procedure for the gram synthesis of 3a (Scheme S12)

In a glove-box, an oven-dried 120 mL sealed tube equipped with a stirring bar was charged with $Re_2(CO)_{10}$ (234.9 mg, 0.036 mmol, 0.08 equiv), the tube was removed from the glove-box and degassed o-xylene (45 mL), 2,2,2-trifluoro-*N*,1-diphenylethan-1-imine **1a** (1.122 g, 4.5 mmol, 1 equiv), 1-isocyanato-4-methylbenzene **2a** (1.198 g, 9.0 mmol, 2 equiv) were added under Ar. The resulting reaction mixture was then stirred at 150 °C for 80 h. After reaction completed, the mixture was transferred into to a round-bottom flask with CH_2Cl_2 and concentrated under reduced vacuum. The residue was purified by flash column chromatography on silica gel (eluent: petroleum ether/ethyl acetate, gradient: 99:1 to 95:5) to afford the desired product **3a** (1.39 g, 81%) as a white solid.

Procedure for the synthesis of derivatives 4 (Scheme S13) (Comins and Hiebel, 2005)

To a solution of **3a** (76.5 mg, 0.2 mmol, 1 equiv) in anhydrous THF (5 mL) at 0 °C was slowly added the LiHMDS (1 M in THF, 1.6 mL, 8 equiv,). After stirred at 0 °C for 10 min, methane iodide (99.6 μL, 1.6 mmol, 8 equiv) was added and the reaction mixture was refluxed for 24 h. After cooling to room temperature, water (20 mL) was added to

the mixture which was further extracted with EtOAc (3 × 20 mL). The combined organic layers was dried over Na_2SO_4, filtered and concentrated under vacuum. The residue was purified by flash column chromatography on silica gel (height 16 cm, width 1.5 cm, eluent: petroleum ether/ethyl acetate, gradient: 99:1 to 97:3) to afford the product **4** as a white solid, yield = 86% (67.8 mg). R_f (petroleum ether/ethyl acetate = 91:9): 0.2.

3-(methyl(phenyl)amino)-2-(p-tolyl)-3-(trifluoromethyl)isoindolin-1-one 4. ^{1}H NMR (400 MHz, $CDCl_3$) δ 7.93 (d, J = 7.6 Hz, 1H), 7.52 – 7.35 (m, 4H), 7.30 (t, J = 8.0 Hz, 3H), 7.11 (t, J = 8.0 Hz, 2H), 6.95 (t, J = 6.8 Hz, 3H), 3.05 (s, 3H), 2.43 (s, 3H). **^{19}F NMR** (376 MHz, $CDCl_3$) δ -69.1 (s). **^{13}C NMR** (101 MHz, $CDCl_3$) δ 167.7, 148.6, 140.4, 138.4, 133.2, 132.5, 132.3, 130.2, 129.8, 129.1, 128.5, 124.51, 124.48, 124.0, 123.7, 123.5 (q, J = 289.9 Hz), 85.3 (q, J = 29.3 Hz), 39.4 (q, J = 3.0 Hz), 21.2. **IR** (KBr, cm^{-1}) ν: 3412, 3056, 2924, 2856, 1711, 1602, 1506, 1360, 1245, 1178, 1120, 1035, 952, 765, 712. **HRMS** (ESI) calcd for $C_{23}H_{20}F_3N_2O^+$ *m/z* 397.1522 $[M+H]^+$, Found 397.1526.

Procedure for the synthesis of derivatives 5 (Scheme S14)

In a glove-box, an oven-dried 25 mL sealed tube equipped with a stirring bar was charged with Cs_2CO_3 (97.7 mg, 0.3 mmol, 1.5 equiv), and the tube was removed from the glove-box. Then, the PIDA (128.8 mg, 0.4 mmol, 2 equiv), 3-imino-1-isoindolinones (76.5 mg, 0.2 mmol, 1 equiv) and TFE (3 mL) were added under Ar. The resulting reaction mixture was stirred at 70 °C for 2 h. Then, PIDA (0.2 mmol, 1 equiv) was added and the mixture was stirred at 70 °C for another 2 h. After that, an additional PIDA (0.2 mmol, 1 equiv) was added (the starting material was completely consumed monitored by TLC after 2 h). The reaction mixture was quenched with saturated aqueous NH_4Cl (10 mL), extracted with EtOAc (3 × 10 mL) and washed with brine (3 × 10 mL). The combined organic layers was dried over Na_2SO_4, filtered and concentrated under vacuum. The residue was purified by flash column chromatography on silica gel (height 16 cm, width 1.5 cm, eluent: petroleum ether/ethyl acetate, gradient: 99:1 to 93:7) afford the product **5** as a white solid, yield = 59% (68.3 mg). R_f (petroleum ether/ethyl acetate = 91:9): 0.2.

3-((4,4-bis(2,2,2-trifluoroethoxy)cyclohexa-2,5-dien-1-ylidene)amino)-2-(p-tolyl)-3-(trifluoromethyl)isoindolin-1-one 5. ^{1}H NMR (400 MHz, $CDCl_3$) δ 8.06 – 7.95 (m, 1H), 7.72 – 7.63 (m, 2H), 7.57 – 7.49 (m, 1H), 7.19 (d, J = 8.0 Hz, 2H), 7.00 (d, J = 8.4 Hz, 2H), 6.66 (dd, J = 10.4, 2.0 Hz, 1H), 6.45 (dd, J = 10.4, 2.8 Hz, 1H), 6.24 (dd, J = 10.8, 2.8 Hz, 1H), 6.02 (dd, J = 10.8, 2.0 Hz, 1H), 4.02 – 3.80 (m, 4H), 2.36 (s, 3H).

^{19}F NMR (376 MHz, $CDCl_3$) δ -74.6 (s), -77.5 (s). **^{13}C NMR** (101 MHz, $CDCl_3$) δ 168.0, 160.8, 142.1, 138.5, 136.5, 134.2, 133.8, 132.6, 131.1, 130.9, 129.9, 127.6, 124.8, 124.3, 123.8 (q, J = 287.9 Hz), 123.20 (q, J = 278.8 Hz), 123.18 (q, J = 278.8 Hz), 121.9, 93.4, 82.7 (q, J = 30.3 Hz), 60.7 (q, J = 36.4 Hz), 60.4 (q, J = 36.4 Hz), 21.1, one carbon was overlapped. **IR** (KBr, cm^{-1}) ν: 3417, 2926, 1733, 1599, 1517, 1468, 1350, 1285, 1171, 1006, 970, 820, 722. **HRMS** (ESI) calcd for $C_{26}H_{20}F_9N_2O_3^+$ *m/z* 579.1325 $[M+H]^+$, Found 579.1318.

Procedure for the synthesis of derivative 6 (Scheme S15)

An oven-dried 25 mL sealed tube equipped with a stirring bar was charged with **3a** (38.2 mg, 0.1 mmol, 1 equiv), CH_3CN (2 mL) and $BF_3{\cdot}Et_2O$ (42.6 mg, 0.3 mmol, 3 equiv) under Ar. The resulting reaction mixture was stirred at 80 °C for 24 h. After cooling down to room temperature, the volatiles were removed under vacuum. The residue was directly purified by flash column chromatography on silica gel (height 16 cm, width 1.5 cm, eluent: petroleum ether/ethyl acetate, gradient: 95:5 to 83:17) to afford the product **6** as a white solid, yield = 81% (24.8 mg). R_f (petroleum ether/ethyl acetate = 75:25): 0.2.

3-hydroxy-2-(p-tolyl)-3-(trifluoromethyl)isoindolin-1-one 6. ^{1}H NMR (400 MHz, $CDCl_3$) δ 7.76 (d, J = 7.6 Hz, 1H), 7.71 – 7.64 (m, 1H), 7.62 – 7.53 (m, 2H), 7.24 – 7.11 (m, 4H), 4.44 (s, 1H), 2.41 (s, 3H). **^{19}F NMR** (376 MHz, $CDCl_3$) δ -78.4 (s). **^{13}C NMR** (101 MHz, $CDCl_3$) δ 167.5, 139.9, 138.6, 133.1, 131.4, 131.3, 131.1, 129.9, 128.6, 124.1, 123.9, 122.7 (q, J = 288.9 Hz), 88.9 (q, J = 33.3 Hz), 21.2. **IR** (KBr, cm^{-1}) ν: 3261, 3055, 2927, 1695, 1614, 1516, 1469, 1375, 1259, 1183, 1078, 946, 883, 813, 700. **HRMS** (ESI) calcd for $C_{16}H_{13}F_3NO_2^+$ *m/z* 308.0893 $[M+H]^+$, Found 308.0896.

Procedure for the synthesis of derivative 10 (Scheme S16) (Trost and Debien, 2015)

To a solution of *Myrtenal* (3.8 mL, 25 mmol, 1 equiv) in THF (30 mL) was slowly added TBAF (1 M in THF, 12.5 mmol, 0.5 equiv) and $TMSCF_3$ (8.1 mL, 55 mmol, 2.2 equiv) at -40 °C under Ar. After addition completed, the reaction mixture was slowly warmed to room temperature and stirred at that temperature for 20 h. The pale yellow reaction mixture was quenched by the addition of HCl (2 M, 7 mL) and then separated. The aqueous layer was extracted with Et_2O (3 × 40 mL) and the combined organic layers was dried over Na_2SO_4, filtered, and concentrated under vacuum. The residue

was purified by flash column chromatography on silica gel (height 16 cm, width 4.5 cm, eluent: petroleum ether/ethyl acetate, gradient: 99:1 to 97:3) afford the product **7** as a yellow oil, yield = 51% (2.80 g). R_f (petroleum ether/ethyl acetate = 91:9): 0.5.

To a solution of DMP (1.02 g, 2.4 mmol, 1.2 equiv) in DCM (3 mL) was added the solution of alcohol **7** (440.0 mg, 2 mmol, 1 equiv) in DCM (3 mL) at 0 °C. The resulting reaction mixture was stirred at room temperature for 30 min. Then aqueous NaOH (0.5 M, 5 mL) was added to quench the reaction and the mixture was extracted with Et_2O (3 × 40 mL). The combined organic layer was dried over Na_2SO_4, filtered and concentrated under vacuum. The residue was purified by flash column chromatography on silica gel (height 16 cm, width 1.5 cm, eluent: petroleum ether) afford the product **8** as a pale yellow oil, yield = 73% (0.32 g). R_f (petroleum ether): 0.8.

The compound **9** was synthesized according to procedure A (on a 2 mmol scale, 2 equiv of 4-bromoaniline and 20 mol% of *p*-toluenesulfonic acid monohydrate were used). Compound **10** was synthesized according procedure G (on a 0.3 mmol scale, o-xylene, 60 h).

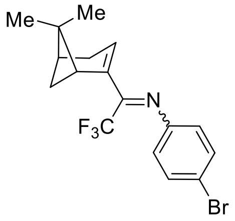

9

***N*-(4-bromophenyl)-1-((1R,5S)-6,6-dimethylbicyclo[3.1.1]hept-2-en-2-yl)-2,2,2-trifluoroethan-1-imine 9.** The product was purified by flash column chromatography on silica gel (height 16 cm, width 2.5 cm, eluent: petroleum ether) as a yellow oil, yield = 50% (0.37 g). R_f (petroleum ether): 0.6. **^{1}H NMR** (400 MHz, $CDCl_3$) δ 7.42 (d, *J* = 8.4 Hz, 2H), 6.71 (d, *J* = 8.8 Hz, 2H), 6.03 (s, 1H), 2.31 (t, *J* = 3.2 Hz, 2H), 2.24 – 2.16 (m, 1H), 2.03 – 1.98 (m, 1H), 1.94 (t, *J* = 5.2 Hz, 1H), 1.15 (s, 3H), 0.91 (d, *J* = 9.2 Hz, 1H), 0.77 (s, 3H). **^{19}F NMR** (376 MHz, $CDCl_3$) δ – 69.8 (s). **^{13}C NMR** (101 MHz, $CDCl_3$) δ 157.1 (q, *J* = 33.3 Hz), 147.2, 138.6, 133.3, 131.8, 121.5, 119.7 (q, *J* = 280.8 Hz), 118.2, 44.3, 39.5, 37.8, 32.2, 31.2, 25.9, 20.7. **IR** (KBr, cm^{-1}) ν: 2930, 1887, 1644, 1476, 1305, 1134, 1007, 943, 887, 827, 731, 521. **HRMS** (ESI) calcd for $C_{17}H_{18}B_rF_3N^+$ *m/z* 372.0569 $[M+H]^+$, Found 372.0580.

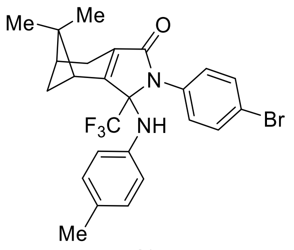

10

2-(4-bromophenyl)-5,5-dimethyl-3-(p-tolylamino)-3-(trifluoromethyl)-2,3,4,5,6,7-hexahydro-1H-4,6-methanoisoindol-1-one 10. o-xylene as solvent, 60 h. (height 18 cm, width 1.5 cm, eluent: petroleum ether/ethyl acetate, gradient: 99:1 to 97:3) as a white solid, yield = 55% (83.4 mg, **mixture of isomer, ratio = 1:0.4**). R_f (petroleum ether/ethyl acetate = 91:9): 0.3. **^{1}H NMR** (400 MHz, $CDCl_3$) δ 7.37 – 7.27 (m, 2.80H), 7.16 (d, J = 8.4 Hz, 2.80H), 7.10 – 7.01 (m, 2.80H), 6.80 – 6.65 (m, 2.82H), 4.49 (s, 1H), 4.45 (s, 0.40H), 2.73 – 2.39 (m, 5.81H), 2.34 (s, 4.22H), 2.32 – 2.24 (m, 1.34H), 1.40 – 1.14 (m, 5.63H), 0.92 – 0.81 (m, 4.29H). **^{19}F NMR** (376 MHz, $CDCl_3$) δ – 76.7, (s, major), – 76.9 (s, minor). **^{13}C NMR** (101 MHz, $CDCl_3$, major) δ 169.2, 160.5, 141.6, 138.1, 135.2, 132.2, 131.8, 129.9, 128.4, 122.8 (q, J = 288.9 Hz), 118.9, 113.8, 81.6 (q, J = 29.3 Hz), 41.1, 40.5, 40.4, 32.1, 26.1, 21.1, 20.7. **^{13}C NMR** (101 MHz, $CDCl_3$, minor) δ 169.2, 160.6, 141.2, 137.8, 135.3, 132.1, 132.0, 129.9, 128.0, 119.7, 114.1, 41.1, 40.2, 39.6, 32.1, 26.1, 21.4, 21.1, $\underline{C}F_3$ and $\underline{C}CF_3$ did not observed. **IR** (KBr, cm^{-1}) ν: 3331, 2927, 1702, 1596, 1502, 1387, 1344, 1258, 1164, 1063, 907, 815, 731. **HRMS** (ESI) calcd for $C_{25}H_{25}BrF_3N_2O^+$ m/z 505.1097 $[M+H]^+$, Found 505.1101.

Procedure for the synthesis of derivative 14 (Scheme S17) (Trost and Debien, 2015)

To a solution of *Perillaldehyde* (4.7 mL, 30 mmol, 1 equiv) in THF (60 mL) was slowly added TBAF (1 M in THF, 15 mmol, 0.5 equiv) and $TMSCF_3$ (9.8 mL, 66 mmol, 2.2 equiv) at -40 °C under Ar. After addition completed, the reaction mixture was slowly warmed to room temperature and stirred at that temperature for 20 h. The pale yellow reaction mixture was quenched by the addition of aqueous HCl (1 M, 30 mL) and then separated. The aqueous layer was extracted with Et_2O (3 × 40 mL) and the combined organic layers was dried over Na_2SO_4, filtered, and concentrated under vacuum. The residue was purified by flash column chromatography on silica gel (height 20 cm, width 1.5 cm, eluent: petroleum ether/ethyl acetate, gradient: 99:1 to 95:5) afford the product **11** as a yellow oil, yield = 68% (4.50 g). R_f (petroleum ether/ethyl acetate = 91:9): 0.3.

To a solution of DMP (5.1 g, 12 mmol, 1.2 equiv) in DCM (25 mL) was added the

solution of alcohol **11** (2.2 g, 10 mmol, 1 equiv) in DCM (25 mL) at 0 °C. The resulting reaction mixture was stirred at room temperature for 30 min. Then aqueous NaOH (0.5 M, 5 mL) was added to quench the reaction and the mixture was extracted with Et_2O (3 × 40 mL). The combined organic layer was dried over Na_2SO_4, filtered and concentrated under vacuum. The residue was purified by flash column chromatography on silica gel (height 16 cm, width 1.5 cm, eluent: petroleum ether) afford the product **12** as a colorless oil, yield = 81% (1.76 g). R_f (petroleum ether): 0.9.

The compound **13** was synthesized according to procedure A (on a 5 mmol scale, 2 equiv of 4-bromoaniline and 20% mmol of *p*-toluenesulfonic acid monohydrate were used). Compound **14** was synthesized according procedure G (on a 0.3 mmol scale, o-xylene, 60 h).

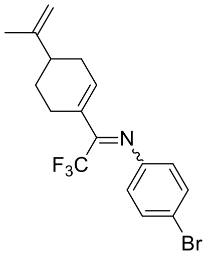

13

***N*-(4-bromophenyl)-2,2,2-trifluoro-1-(4-(prop-1-en-2-yl)cyclohex-1-en-1-yl)ethan-1-imine 13.** O-xylene as solvent, 60 h. The product was purified by flash column chromatography on silica gel (height 20 cm, width 3.5 cm, eluent: petroleum ether) as an orange oil, yield = 60% (1.11 g, **mixture of isomer, ratio = 1:0.15**). R_f (petroleum ether): 0.6. **^{1}H NMR** (400 MHz, $CDCl_3$) δ 7.44 (d, J = 8.4 Hz, 2H, major), 7.32 (d, J = 8.8 Hz, 0.30H, minor), 6.77 (d, J = 8.4 Hz, 2H, major), 6.64 (d, J = 8.8 Hz, 0.30H, minor), 6.00 (s, 1H), 4.72 (s, 1.15H), 4.63 (s, 1H), 2.30 – 2.16 (m, 1.16H), 2.12 – 1.82 (m, 4.30H), 1.69 (s, 3.35H), 1.38 – 1.19 (m, 2.56H). **^{19}F NMR** (376 MHz, $CDCl_3$) δ – 70.3 (s, minor), – 71.2 (s, major). **^{13}C NMR** (101 MHz, $CDCl_3$, major) δ 159.5 (q, J = 33.3 Hz), 148.2, 146.7, 134.5, 131.9, 129.0, 121.4, 119.6 (q, J = 280.9 Hz), 118.5, 109.4, 39.4, 30.4, 26.6, 26.5, 20.6. **^{13}C NMR** (101 MHz, $CDCl_3$, minor) δ 151.7, 146.3, 131.9, 128.7, 126.8, 122.3, 33.9, 29.7, 23.5, six carbon did not observed. **IR** (KBr, cm^{-1}) ν: 3080, 2930, 1700, 1646, 1478, 1443, 1323, 1191, 1140, 1067, 1009, 896, 829, 716. **HRMS** (ESI) calcd for $C_{17}H_{18}BrF_3N^+$ *m/z* 372.0569 $[M+H]^+$, Found 372.0570.

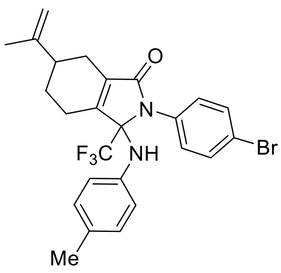

14

2-(4-bromophenyl)-6-(prop-1-en-2-yl)-3-(p-tolylamino)-3-(trifluoromethyl)-2,3,4,5,6,7-hexahydro-1H-isoindol-1-one 14. O-xylene as solvent, 60 h. (height 20 cm, width 1.5 cm, eluent: petroleum ether/ethyl acetate, gradient: 99:1 to 95:5) as a white solid, yield = 56% (85.0 mg, **mixture of isomer, ratio = 1:0.18**). R$_f$ (petroleum ether/ethyl acetate = 91:9): 0.3. **^{1}H NMR** (400 MHz, $CDCl_3$) δ 7.50 – 7.44 (m, 0.37H), 7.33 (dd, J = 8.8, 3.6 Hz, 2H), 7.23 – 7.07 (m, 2.73H), 7.04 – 6.88 (m, 2.34H), 6.58 (dd, J = 12.4, 8.8 Hz, 2H), 4.90 – 4.67 (m, 2.19H), 4.51 (d, J = 10.8 Hz, 1H), 3.12 – 3.02 (m, 0.18H), 2.73 – 2.60 (m, 1H), 2.39 – 2.23 (m, 6.12H), 2.07 – 1.92 (m, 1.24H), 1.82 – 1.74 (m, 3.33H), 1.39 – 1.28 (m, 2.48H). **^{19}F NMR** (376 MHz, $CDCl_3$) δ – 76.7 (s, major), – 76.8 (s, major), – 77.6 (s, minor). **^{13}C NMR** (101 MHz, $CDCl_3$) δ 169.3, 169.2, 148.4, 147.9, 147.7, 146.8, 141.5, 141.4, 139.4, 138.8, 138.3, 138.3, 132.5, 129.9, 129.9, 128.6, 128.5, 123.0 (q, J = 289.9 Hz), 117.6, 117.5, 110.2, 110.0, 81.6 (q, J = 29.3 Hz), 81.5 (q, J = 29.3 Hz), 40.7, 39.2, 27.3, 26.4, 26.3, 25.5, 21.1, 20.8. **IR** (KBr, cm^{-1}) ν: 3343, 3048, 2926, 1697, 1598, 1499, 1397, 1358, 1263, 1162, 1077, 889, 816, 733. **HRMS** (ESI) calcd for $C_{25}H_{25}BrF_3N_2O^+$ *m/z* 505.1097 [M+H]$^+$, Found 505.1100.

Procedure for the synthesis of derivatives 22 and 23 (Scheme S18)

(Furuya et al., 2009; Thompson et al., 2005; Hu et al., 2016)

To a solution of *Tocopherol* (4.5 g, 11.2 mmol, 1 equiv) in CH_2Cl_2 (58 mL) at 0 °C was added triethylamine (3.9 mL, 28 mmol, 2.5 equiv) and trifluoromethanesulfonic anhydride (2.5 mL, 14.6 mmol, 1.3 equiv). The resulting reaction mixture was stirred at 0 °C for 30 min before the addition of saturated aqueous $NaHCO_3$ (50 mL). Then reaction mixture was separated and the aqueous layer was extracted with CH_2Cl_2 (3 × 50 mL). The combined organic layer was washed with brine (80 mL), dried over Na_2SO_4, filtered, and concentrated under vacuum. The residue was purified by flash chromatography on silica gel to afford the product **15**, yield = 85% (5.1 g). R$_f$ (petroleum ether): 0.5.

The aryl triflate **15** (0.64 g, 1.2 mmol, 1 equiv) and $PdCl_2$(dppf) (88.0 mg, 0.12 mmol, 10 mol%) were dissolved in anhydrous dioxane (6 mL), followed by the addition of

Et_3N (0.5 mL, 3.6 mmol, 3 equiv) and pinacolborane (0.4 mL, 2.4 mmol, 2 equiv). The resulting reaction was heated at 100 °C for 4 h until the disappearance of the starting material. Then the reaction mixture was quenched with water (30 mL) and extracted with CH_2Cl_2 (3 × 20 mL). The combined organic layer was dried over Na_2SO_4, filtered and concentrated under vacuum. The residue was purified by flash chromatography on silica gel (height 16 cm, width 2.5 cm, eluent: petroleum ether) to afford the product **16** as a pale yellow oil, yield = 84% (514.0 mg). R_f (petroleum ether): 0.4.

An oven-dried 25 mL sealed tube equipped with a stirring bar was charged with $CuBr_2$ (134.0 mg, 0.6 mmol, 3 equiv), **16** (102.5 mg, 0.2 mmol, 1 equiv) and dry methanol (6 mL) under Ar. The resulting reaction mixture was heated at 90 °C under Ar for 72 h. Upon completion, the reaction mixture was cooled down to room temperature and concentrated under vacuum. The residue was purified by a short column chromatography (100 % hexanes) to afford the desired product **17** as a colorless oil, yield = 72% (67.3 mg). R_f (petroleum ether): 0.9.

To a solution of compound **17** (931.0 mg, 2 mmol, 1 equiv) in dry THF (30 mL) was slowly added *n*-BuLi (2.5 M in hexane, 1.6 mL, 2 equiv) at -78 °C. The mixture was then stirred for 1 hour at that temperature. After that, dry dimethylformamide (0.77 mL, 10 mmol, 5 equiv) was added into the reaction mixture at -78 °C. The resulting reaction mixture was stirred at -78 °C for further 12 h, which was quenched by the addition of water (40 mL). The reaction mixture was extracted with DCM (3 × 40 mL), dried over Na_2SO_4, filtered, concentrated to give the crude product **18** which was used directly for the synthesis of **19** according to procedure D, overall yield = 56% (541.2 mg).

The compound **20** and **21** was synthesized according procedure D. **22 a**nd **23** was synthesized according procedure G.

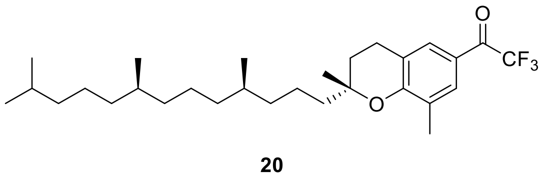

20

1-((*R*)-2,8-dimethyl-2-((4*R*,8*R*)-4,8,12-trimethyltridecyl)chroman-6-yl)-2,2,2-trifluoroethan-1-one 20. Starting from **19**, on a 0.91 mmol scale, The product was purified by flash column chromatography on silica gel (height 18 cm, width 2.5 cm, eluent: petroleum ether) as a pale yellow oil, yield = 53% (230.8 mg). R_f (petroleum ether): 0.5. **^{1}H NMR** (400 MHz, $CDCl_3$) δ 7.70 (d, *J* = 6.4 Hz, 2H), 2.89 – 2.71 (m, 2H), 2.21 (s, 3H), 1.93 – 1.76 (m, 2H), 1.67 – 1.57 (m, 2H), 1.54 – 1.21 (m, 16H), 1.17 – 1.02 (m, 6H), 0.92 – 0.78 (m, 12H). **^{19}F NMR** (376 MHz, $CDCl_3$) δ – 71.0 (s). **^{13}C NMR** (101 MHz, $CDCl_3$) δ 179.1 (q, *J* = 33.3 Hz), 159.2, 130.6, 127.3, 121.0, 120.9,

117.1 (q, J = 291.9 Hz), 78.4, 40.3, 39.4, 37.4, 37.4, 37.3, 37.3, 32.8, 32.6, 30.7, 28.0, 24.8, 24.4, 24.3, 22.7, 22.6, 22.1, 20.9, 19.7, 19.6, 16.1. **IR** (KBr, cm^{-1}) ν: 3450, 2931, 2857, 1703, 1598, 1473, 1352, 1280, 1196, 1142, 1015, 961, 852, 769, 712. **HRMS** (ESI) calcd for $C_{29}H_{46}BrF_3O_2^+$ *m/z* 483.3444 $[M+H]^+$, Found 483.3458.

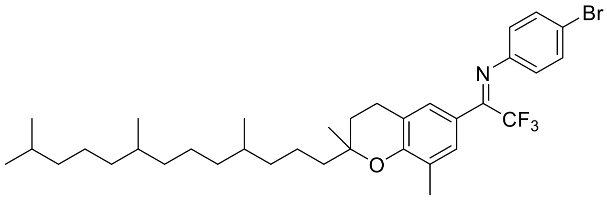

21

***N*-(4-bromophenyl)-1-(2,8-dimethyl-2-(4,8,12-trimethyltridecyl)chroman-6-yl)-2,2,2-trifluoroethan-1-imine 21.** On a 1.7 mmol scale, 2 equiv of 4-bromoaniline and 20 mol% of *p*-toluenesulfonic acid monohydrate were used. The product was purified by flash column chromatography on silica gel (height 25 cm, width 2.5 cm, eluent: petroleum ether + 5% Et_3N) as a yellow oil, yield = 29% (316.0 mg). R_f (petroleum ether): 0.4. **^{1}H NMR** (400 MHz, $CDCl_3$) δ 7.34 (d, J = 8.4 Hz, 2H), 6.79 (d, J = 6.4 Hz, 2H), 6.67 (d, J = 8.4 Hz, 2H), 2.69 – 2.54 (m, 2H), 2.04 (s, 3H), 1.82 – 1.71 (m, 2H), 1.56 – 1.52 (m, 2H), 1.41 – 1.33 (m, 4H), 1.31 – 1.21 (m, 12H), 1.51 – 1.05 (m, 6H), 0.88 – 0.83 (m, 12H). **^{19}F NMR** (376 MHz, $CDCl_3$) δ – 69.7 (s). **^{13}C NMR** (101 MHz, $CDCl_3$) δ 157.4 (q, J = 33.3 Hz), 154.3, 146.9, 131.9, 128.9, 128.1, 126.7, 122.3, 120.6, 120.0 (q, J = 280.8 Hz), 119.4, 118.1, 40.3, 39.4, 37.4, 37.4, 37.4, 37.3, 32.8, 32.7, 30.7, 28.0, 24.8, 24.4, 24.2, 22.7, 22.6, 22.1, 20.9, 19.7, 19.6, 16.0. **IR** (KBr, cm^{-1}) ν: 3435, 2931, 1703, 1600, 1475, 1350, 1278, 1190, 1141, 1018, 962, 830. **HRMS** (ESI) calcd for $C_{35}H_{50}BrF_3NO^+$ *m/z* 636.3022 $[M+H]^+$, Found 636.3029. Contaminated with trace inseparable impurity.

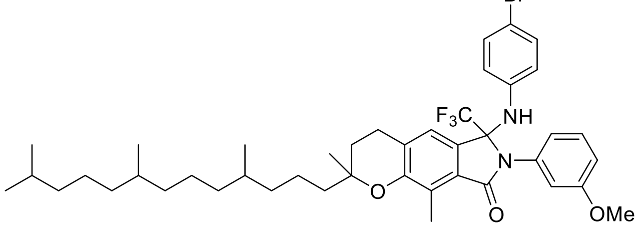

22

6-((4-bromophenyl)amino)-7-(3-methoxyphenyl)-2,9-dimethyl-6-(trifluoromethyl)-2-(4,8,12-trimethyltridecyl)-3,4,6,7-tetrahydropyrano[2,3-f]isoindol-8(2H)-one 22. On a 0.1 mmol scale, o-xylene, 140 °C, 60 h. The product was purified by flash column chromatography on silica gel (height 16 cm, width 1.5 cm, eluent: petroleum ether/ethyl acetate, gradient: 99:1 to 95:5) as a colorless oil, yield =

15% (12.0 mg, **mixture of isomer, ratio = 1:1**). R*f* (petroleum ether/ethyl acetate = 91:9): 0.2. **^{1}H NMR** (400 MHz, $CDCl_3$) δ 7.29 – 7.26 (m, 1H), 7.21 – 7.14 (m, 3H), 6.90 (dd, J = 8.4, 2.4 Hz, 1H), 6.70 (d, J = 8.0 Hz, 1H), 6.60 (s, 1H), 6.24 (d, J = 8.8 Hz, 2H), 4.71 (s, 1H), 3.64 (s, 3H), 3.48 – 3.16 (m, 2H), 2.21 (s, 3H), 1.96 – 1.79 (m, 2H), 1.69 – 1.59 (t, J = 14.7 Hz, 2H), 1.38 – 1.01 (m, 22H), 0.92 – 0.84 (m, 12H). **^{19}F NMR** (376 MHz, $CDCl_3$) δ – 77.87 (s), – 77.88 (s). **^{13}C NMR** (101 MHz, $CDCl_3$,) δ 168.4, 160.1, 154.7, 141.2, 135.4, 132.41, 132.37, 132.2, 129.8, 128.3, 127.4, 123.8, 123.4 (q, J = 288.9 Hz), 121.4, 120.81, 120.76, 118.0, 114.8, 114.4, 112.8, 79.6 (q, J = 29.3 Hz), 55.1, 40.5, 39.8, 39.4, 37.51, 37.46, 37.45, 37.40, 37.39, 37.30, 37.28, 32.8, 32.7, 30.1, 30.0, 29.7, 28.0, 24.8, 24.5, 24.4, 23.8, 22.7, 22.6, 21.0, 20.9, 19.74, 19.67, 18.6, 18.5, 17.22, 17.21. **IR** (KBr, cm^{-1}) ν: 3450, 2930, 1634, 1488, 1462, 1367, 1258, 1171, 1074, 812, 734. **HRMS** (ESI) calcd for $C_{43}H_{57}BrF_3N_2O_3^+$ *m/z* 785.3499 $[M+H]^+$, Found 785.3495.

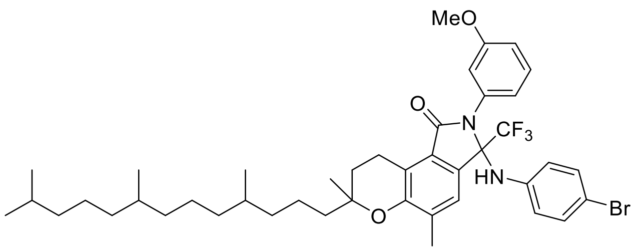

23

3-((4-bromophenyl)amino)-2-(3-methoxyphenyl)-5,7-dimethyl-3-(trifluoromethyl)-7-(4,8,12-trimethyltridecyl)-2,3,8,9-tetrahydropyrano[3,2-e]isoindol-1(7H)-one 23. On a 0.1 mmol scale, o-xylene, 140 °C, 60 h. The product was purified by flash column chromatography on silica gel (height 16 cm, width 1.5 cm, eluent: petroleum ether/ethyl acetate, gradient: 99:1 to 95:5) as a colorless oil, yield = 42% (33.0 mg, **mixture of isomer, ratio = 1:1**). R*f* (petroleum ether/ethyl acetate = 91:9): 0.2. **^{1}H NMR** (400 MHz, $CDCl_3$) δ 7.26 – 7.22 (m, 1H), 7.19 (d, J = 8.8 Hz, 2H), 7.09 (s, 1H), 6.90 (dd, J = 8.4, 2.4 Hz, 1H), 6.69 (d, J = 8.0 Hz, 1H), 6.59 (s, 1H), 6.23 (d, J = 8.4 Hz, 2H), 4.70 (s, 1H), 3.63 (s, 3H), 2.88 – 2.69 (m, 2H), 2.62 (s, 3H), 1.88 – 1.73 (m, 2H), 1.65 – 1.49 (m, 7H), 1.40 – 1.04 (m, 27H), 0.88 – 0.84 (m, 12H). **^{19}F NMR** (376 MHz, $CDCl_3$) δ – 77.96 (s), – 77.98 (s). **^{13}C NMR** (101 MHz, $CDCl_3$) δ 168.4, 160.1, 154.78, 154.76, 141.2, 135.4, 132.2, 129.8, 127.92, 127.86, 127.8, 127.6, 125.7, 125.6, 123.4 (q, J = 289.9 Hz), 122.6, 121.5, 117.9, 114.8, 114.4, 112.7, 79.2 (q, J = 30.3 Hz), 55.1, 40.7, 40.0, 39.4, 37.50, 37.48, 37.43, 37.41, 37.28, 37.27, 32.8, 32.7, 29.7, 28.0, 24.8, 24.45, 24.43, 23.9, 23.1, 22.7, 22.6, 21.04, 20.97, 19.7, 19.6. **IR** (KBr, cm^{-1}) ν: 3448, 2927, 1704, 1636, 1494, 1457, 1357, 1258, 1169, 1085, 733. **HRMS** (ESI) calcd for $C_{43}H_{57}BrF_3N_2O_3^+$ *m/z* 785.3499 $[M+H]^+$, Found 785.3508.

General procedure for the synthesis of polymers 24a-d (Scheme S19)

(Sueki et al., 2013)

An oven-dried 25 mL schlenk tube equipped with a stirring bar was transferred into a glovebox (through standard glovebox operation), where $Re_2(CO)_{10}$ (26.1 mg, 0.04 mmol, 0.1 equiv) was added. The tube was then removed from the glovebox and placed under Ar. Then the ketimine **1ai-1al** (0.4 mmol, 1 equiv), isocyanate **2v** (64.1 mg, 0.4 mmol, 1 equiv), and o-xylene (2 mL) were added subsequently to the test tube under Ar. The resulting reaction mixture was then stirred at 150 °C for 72 h. After reaction completed, the mixture was cooled down to room temperature and concentrated under vacuum. The residue was washed with petroleum ether/ethyl acetate (50:1, 100 mL), filtered and dried under vacuum to give the crude polyamides as black solid. Next, 100 mg of each crude polyamide was further purified by dialysis against CH_2Cl_2 using a benzoylated cellulose membrane (MWCO 500 g/mol) for 2 days. Finally, the solvent was removed under reduced pressure and the obtained product was dried in vacuum for 24 h.

24a, black solid, yield = 45% (64.0 mg). **IR** (KBr, cm^{-1}) ν: 3452, 2930, 1911, 1638, 1513, 1349, 1179, 645.
24b, black solid, yield = 34% (57.0 mg). **IR** (KBr, cm^{-1}) ν: 3425, 2926, 2856, 2015, 1902, 1719, 1512, 1467, 1349, 1291, 1181, 1050, 829, 730.
24c, black solid, yield = 49% (66.0 mg). **IR** (KBr, cm^{-1}) ν: 3428, 2926, 2858, 2024, 1907, 1720, 1619, 1513, 1344, 1260, 1179, 970, 826, 732.
24d, black solid, yield = 32% (55.0 mg). **IR** (KBr, cm^{-1}) ν: 3427, 2925, 2854, 2026, 1909, 1723, 1621, 1515, 1345, 1260, 1177, 970, 825, 728.

References

Elliott, D.C., Marti, A., Mauleón, P., Pfaltz, A. (2019). H_2 Activation by Non-Transition-Metal Systems: Hydrogenation of Aldimines and Ketimines with $LiN(SiMe_3)_2$. Chem. Eur. J. *25*, 1918-1922.

Dai, X., Cahard, D. (2014). Enantioselective Synthesis of α-Trifluoromethyl Arylmethylamines by Ruthenium-Catalyzed Transfer Hydrogenation Reaction. Adv. Synth. Catal. *356*, 1317-1328.

Strømgaard, K., Saito, D. R., Shindou, H., Ishii, S., Shimizu, T., Nakanishi, K. (2002). Ginkgolide Derivatives for Photolabeling Studies: Preparation and Pharmacological Evaluation. J. Med. Chem. *45*, 4038-4046.

Fujita, T., Takazawa, M., Sugiyama, K., Suzuki, N., Ichikawa, J. (2017). Domino C–F Bond Activation of the CF_3 Group: Synthesis of Fluorinated Dibenzo[*a*,*c*][7]annulenes from 2-(Trifluoromethyl)-1-alkenes and 2,2′-Diceriobiaryls. Org. Lett. *19*, 588-591.

Trost, B.M., Debien, L. (2015). Palladium-Catalyzed Trimethylenemethane Cycloaddition of Olefins Activated by the σ-Electron-Withdrawing Trifluoromethyl Group. J. Am. Chem. Soc. *137*, 11606-1609.

Chen, L.S., Chen, G.J., Tamborski, C. (1983). Regiospecific Synthesis of Aromatic Compounds via Organometallic Intermediates. J. Organomet. Chem. *251*, 139-148.

Abid, M., Savolainen, M., Landge, S., Hu, J., Prakash, G.K.S., Olah, G.A., Török, B. (2007). Synthesis of trifluoromethyl-imines by solid acid/superacid catalyzed microwave assisted approach. *J. Fluorine Chem. 128*, 587-594.

Henseler, A., Kato, M., Mori, K., Akiyama, T. (2011). Chiral Phosphoric Acid Catalyzed Transfer Hydrogenation: Facile Synthetic Access to Highly Optically Active Trifluoromethylated Amines. Angew. Chem. Int. Ed. *50*, 8180-8183.

Li, C.-L., Chen, M.-W., Zhang, X.-G. (2010). Synthesis of N-aryl trifluoromethylarylketoimines by palladium-catalyzed Suzuki coupling reaction of N-aryltrifluoroacetimidoyl chlorides with aryl boronic acids. *J. Fluorine Chem. 131*, 856-860.

Kiselyov, A.S. (1999). Unexpected behavior of imines derived from trifluoromethylaryl ketones under basic conditions: Convenient synthesis of 2-arylbenzimidazoles and 2-arylbenzoxazoles. Tetrahedron Lett. *40*, 4119-4122.

Patterson, S.E., Janda, L., Strekowski, L. (1992). A new synthesis of *N*-substituted-2-alkyl(or aryl)quinazolin-4-amines by amide base-mediated cyclization of carbox-imidamides derived from 2-(trifluoromethyl)benzenamine. J. Heterocyclic Chem. *29*, 703-706.

Wang, W.-Y., Feng, X., Hu, B.-L., Deng, C.-L., Zhang, X.-G. (2013). Synthesis of 6-(Trifluoromethyl)phenanthridines via Palladium-Catalyzed Tandem Suzuki/C–H Arylation Reactions. J. Org. Chem. *78*, 6025-6030.

Comins, D.L., Hiebel, A.-C. (2005). Asymmetric synthesis of 3,3-disubstituted isoindolinones. Tetrahedron Lett. *46*, 5639-5642.

Furuya, T., Strom, A. E., Ritter, T. (2009). Silver-Mediated Fluorination of Functionalized Aryl Stannanes. J. Am. Chem. Soc. *131*, 1662-1663.

Thompson, A.L.S., Kabalka, G.W., Akula, M.R., Huffman, J.W. (2005). The Conversion of Phenols to the Corresponding Aryl Halides Under Mild Conditions. Synthesis. *4*, 547-550.

Hu, P., Lee, S., Park, K.H., Das, S., Herng, T.S., Gonçalves, T.P., Huang, K.W., Ding, J., Kim, D., Wu, J. (2016). Octazethrene and Its Isomer with Different Diradical Characters and Chemical Reactivity: The Role of the Bridge Structure. J. Org. Chem.

81, 2911-2919.

Sueki, S., Guo, Y., Kanai, M., Kuninobu, Y. (2013). Rhenium-Catalyzed Synthesis of 3-Imino-1-isoindolinones by C-H Bond Activation: Application to the Synthesis of Polyimide Derivatives. Angew. Chem. Int. Ed. *52*, 11879-11883.
